# Genomic Comparison of the P-ATPase Gene Family in Four Cotton Species and Their Expression Patterns in *Gossypium hirsutum*

**DOI:** 10.3390/molecules23051092

**Published:** 2018-05-05

**Authors:** Wen Chen, Guo-Yang Si, Gang Zhao, Muhammad Abdullah, Ning Guo, Da-Hui Li, Xu Sun, Yong-Ping Cai, Yi Lin, Jun-Shan Gao

**Affiliations:** School of Life Sciences, Anhui Agricultural University, Hefei 230036, China; chenwen121314@163.com (W.C.); siguoyang007@163.com (G.-Y.S.); 2007zg2007@163.com (G.Z.); xfcypeng@126.com (M.A.); guoning@ahau.edu.cn (N.G.); ldh@ahau.edu.cn (D.-H.L.); sunxu@ahau.edu.cn (X.S.); swkx12@ahau.edu.cn (Y.-P.C.); linyi320722@163.com (Y.L.)

**Keywords:** brown cotton, evolution, P-ATPase, proanthocyanidins, qRT-PCR

## Abstract

Plant P-type H^+^-ATPase (P-ATPase) is a membrane protein existing in the plasma membrane that plays an important role in the transmembrane transport of plant cells. To understand the variety and quantity of P-ATPase proteins in different cotton species, we combined four databases from two diploid cotton species (*Gossypium raimondii* and *G. arboreum*) and two tetraploid cotton species (*G. hirsutum* and *G. barbadense*) to screen the P-ATPase gene family and resolved the evolutionary relationships between the former cotton species. We identified 53, 51, 99 and 98 *P-ATPase* genes from *G. arboretum, G. raimondii*, *G. barbadense* and *G. hirsutum*, respectively. The structural and phylogenetic analyses revealed that the gene structure was consistent between *P-ATPase* genes, with a close evolutionary relationship. The expression analysis of *P-ATPase* genes showed that many *P-ATPase* genes were highly expressed in various tissues and at different fiber developmental stages in *G. hirsutum*, suggesting that they have potential functions during growth and fiber development in cotton.

## 1. Introduction

H^+^-ATPase is a membrane protein that is widely present in the plant plasma membrane and various endomembranes, and plays an important role in cell metabolism. The representative H^+^-ATPases are currently divided into three types according to structure, namely the vacuolar-type H^+^-ATPases (V-ATPases), H^+^-pyrophosphatases (H^+^-PPases) and P type H^+^-ATPases (P-ATPase). The H^+^-PPases is located in vacuolar or endomembranes; the vacuole H^+^-ATPase is a V-type ATPase; and the plasma membrane H^+^-ATPase belongs to the P-type ATPase. As a P-type pump, P-ATPase can generate and maintain the electrochemical gradient of H^+^ on both sides of the cell membrane by decomposing intracellular ATP and providing energy for the transmembrane transport of various nutrients and ions [1,2]. In addition, the plant P-ATPase also participates in the regulation of various physiological processes, such as intracellular pH, cell elongation, stomatal opening and closing, and plant response to environmental stress [3,4].

To date, 10 subfamilies of P-ATPase have been identified according to their transport of various ions, heavy metals and possibly lipids: P_1B_ (heavy metal ion ATPases [HMA]), P_2A_ (endoplasmic reticulum Ca^2+^ ATPase [ECA]), P_2B_ (autoinhibited Ca^2+^ ATPase [ACA]), P_3A_ (autoinhibited H^+^ ATPase (AHA)), P_4_ (putative aminophospholipid ATPase [ALA]), P_5_ (unknown ATPase), P_1A_ (K^+^ ATPase), P_2C_ (Na^+^/K^+^ ATPase), P_2D_ (Na^+^ or Ca^2+^ ATPase), and P_3B_ (Mg^2+^ ATPase) [5,6]. Forty-six *P-ATPase* genes have been identified in *Arabidopsis* [7], and 43 *P-ATPase* genes have been identified in rice [8]. The gene numbers of five subfamilies (P_1B_, P_2A_, P_2B_, P_3A_, and P_4_) are high, but there are few P_5_ subfamily genes, while the genes of the P_2C_, P_2D_, P_1A_ and P_3B_ subfamilies were absent in *Arabidopsis* and rice [8].

Some studies have revealed that P-ATPase genes play an important role in primary metabolic and secondary metabolic processes in plants. For example, some genes of the P_3A_ subfamily can affect the transport of anthocyanins and proanthocyanidins (PAs) in plants, such as *TT13* in *Arabidopsis* [9] and *PH5* in petunia [10]. However, there are only a few detailed reports about P_3A_-ATPase in cotton, especially involving the transport of PAs, while another study reported that pigment formation in brown cotton fiber is related to the transport of PAs [11]. Cotton is the most important fiber crop in China with wide varieties. The whole genome sequencing of two diploid cotton species (*G. arboretum* and *G. raimondii*) and two tetraploid cotton species (*G. barbadense* and *G. hirsutum*) has been completed [12,13,14,15]. In this study, the *P-ATPase* genes of four cotton species were identified, screened and analyzed using bioinformatics, the evolutionary relationship of the P-ATPase family in cotton has been preliminarily discussed, and the expression pattern of the *P-ATPase* gene in upland cotton has been analyzed. To search for possible genes related to PA accumulation, fluorescent quantitative analysis of the *P_3A_-ATPase* subfamily of genes of brown cotton fibers was used, combined with the accumulation of PAs in brown cotton fibers. This study lays the foundation for further cloning and utilization of *P-ATPase* genes to cultivate new varieties of colored cotton.

## 2. Results

### 2.1. Identification of P-ATPase Genes in Cotton

We first queried the specific domains of *Arabidopsis AHA10* (PF00122, PF00702, and PF00690) on the Pfam website and used the amino acid sequences of these domains to search for candidate genes in the local databases of *G. arboretum*, *G. raimondii*, *G. barbadense* and *G. hirsutum*, which were previously established. We screened these candidate genes using the PFAM and SMART databases [16,17] and identified 301 *P-ATPase* genes with specific domains of *P-ATPase* genes in four cotton species. Fifty-one *P-ATPase* genes were found in *G. raimondii* and 53 *P-ATPase* genes were found in *G. arboretum*, similar to those in *Arabidopsis* and rice, containing 46 and 43 *P-ATPase* genes, respectively. However, 99 and 98 *P-ATPase* genes were found in the tetraploid cotton *G. barbadense* and *G. hirsutum*, respectively. These genes in cotton were classified and named according to the classifications and names of the *P-ATPase* genes in *Arabidopsis*.

In addition, the lengths and molecular weights of proteins encoded by the *P-ATPase* genes were analyzed. The characteristics of 301 *P-ATPase* genes in *G. raimondii*, *G. hirsutum*, *G. arboreum* and *G. barbadense* suggested that the lengths of all P-ATPase proteins differed largely, ranging from 800 to 1301 aa; the molecular weights of the P-ATPase proteins ranged from 90 to 140 kDa in *G. arboretum* and *G. raimondii*, and the lengths and molecular weights of most P-ATPase proteins in *G. barbadense* and *G. hirsutum* had a similar trend as those in *G. arboretum* and *G. raimondii*, but some proteins were different. For example, *GhHMA10* encodes 420 amino acids with a molecular weight of 44.9kDa, and *GbAHA23* encodes 1646 aa with a molecular weight of 183.48 kDa. The isoelectric points of all P-ATPase proteins were predicted; the minimum was 4.82, and the maximum was 8.81 (Table S1).

### 2.2. Chromosome Distribution of P-ATPase Genes

**Figure 1:**
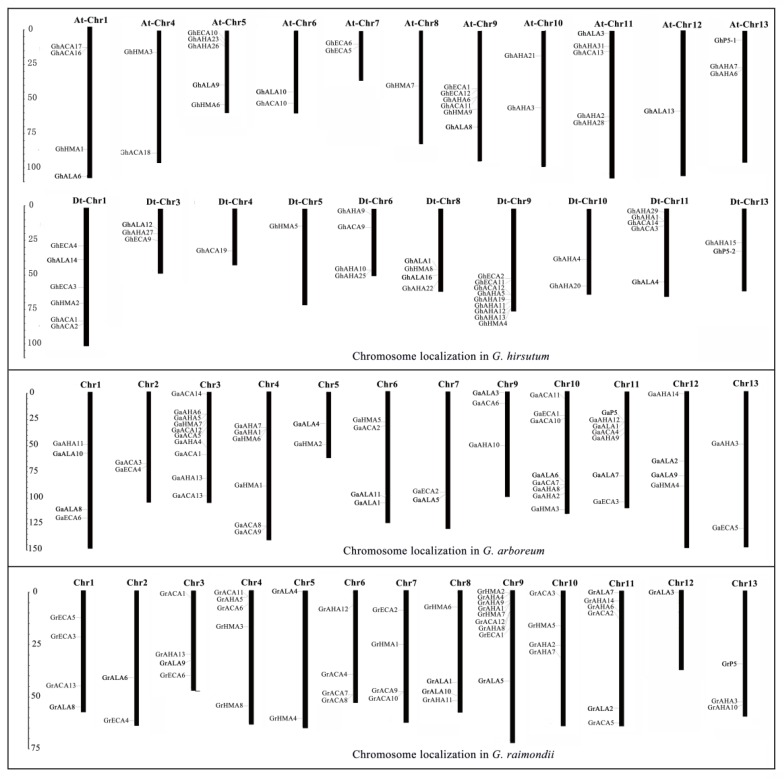
Chromosomal localization of *P-ATPase* genes in *G. hirsutum*, *G. arboretum*, and *G. raimondii*. Number of *P-ATPase* genes on each chromosome and location of each gene on the chromosome are shown on the map. Scale number is in megabases (Mb).

To better understand the distribution of *P-ATPase* genes on chromosomes, according to the genome database information of *G. raimondii*, *G. arboretum* and *G. hirsutum*, the chromosome localization was mapped. The results showed that the *P-ATPase* genes of *G. hirsutum* were tagged on 21 chromosomes, and the *P-ATPase* genes of *G. raimondii* and *G. arboretum* were tagged on 12 and 13 chromosomes (Figure 1). The *P_1B_-ATPase* genes of *G. hirsutum* were distributed in nine chromosomes (At1, Dt1, At4, At5, Dt5, At8, Dt8, At9 and Dt9), and each chromosome had only one gene, while the *P_1B_-ATPase* genes of *G. raimondii* and *G. arboretum* were all distributed in 6 chromosomes, with only one or two genes on each chromosome (Figure 1). The distributions of the *P_2A_-ATPase*, *P_2B_-ATPase*, and *P_3A_-ATPase* genes in *G. raimondii*, *G. arboretum* and *G. hirsutum* were similar to that of the *P_1B_-ATPase* gene, but a few chromosomes had different distributions. For instance, there were four *P_2B_-ATPase* genes on the 6^th^ chromosome of *G. raimondii*, 4 *P_2B_-ATPase* genes on the 3^rd^ chromosome of *G. arboretum*, and six *P_3A_-ATPase* genes on the Dt9 chromosome. The P_4_ subfamily genes in four cotton species are evenly distributed on chromosomes, with one or two genes per chromosome.

### 2.3. Phylogenetic Analysis of the P-ATPase Genes

**Figure 2:**
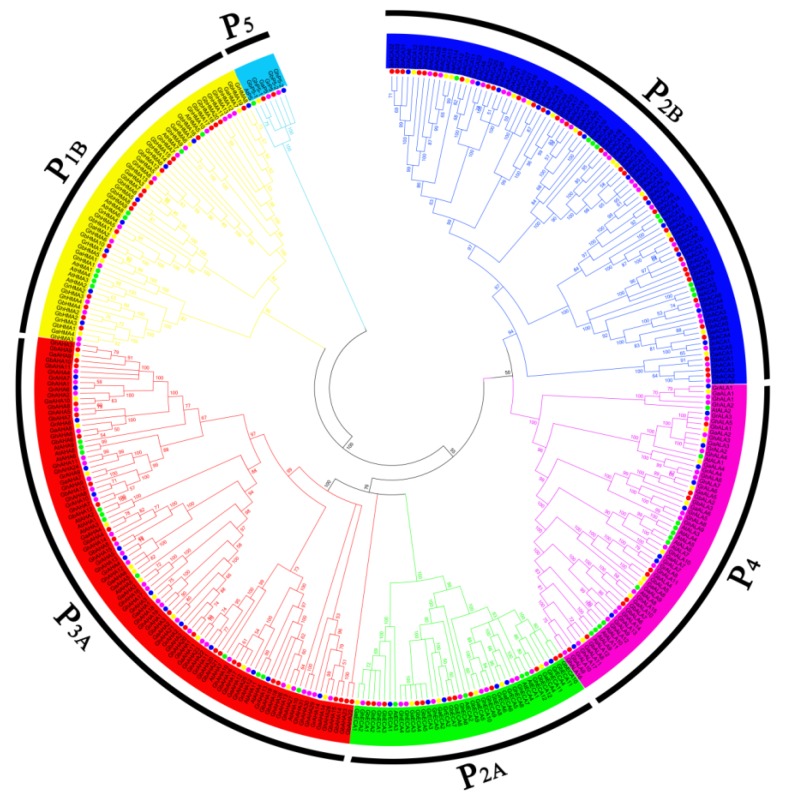
Phylogenetic tree of *P-ATPase* genes in various cottons and *Arabidopsis*, constructed by neighbor-joining (NJ) using MEGA 5 software (1000 bootstrap replicates). Different colors indicate different subfamilies of P-ATPase, the green, orange, blue, red and purple dots indicate *Arabidopsis*, *G. arboretum*, *G. raimondii*, *G. barbadense* and *G. hirsutum* respectively.

To determine the subfamily classification of *P-ATPase* genes in cotton species and the number of genes in each subfamily, a phylogenetic tree was constructed with the sequences of 51 P-ATPase proteins in *G. raimondii*, 53 P-ATPase proteins in *G. arboretum*, 98 P-ATPase proteins in *G. hirsutum*, 99 P-ATPase proteins in *G. barbadense* and 45 P-ATPase proteins in *Arabidopsis*. As shown in the result, all *P-ATPases* genes in each cotton species could be clustered into six subfamilies: P_1B_, P_2B_, P_2A_, P_3A,_ P_4_ and P_5_ (Figure 2). The gene numbers of each subfamily in *G. raimondii* and *G. arboretum* were very similar, and there were large numbers in the P_2B_ and P_3A_ subfamilies, which were approximately twice those in the P_1B,_ P_2A_ and P_4_ subfamilies. (Figure 2). In *G. hirsutum*, the gene numbers were approximately twice that of *G. raimondii* and *G. arboretum*, but the distribution of genes in each subfamily was similar to *G. raimondii*, with the most genes in the P_3A_ subfamily and the least in the P_2A_ subfamily. In *G. barbadense*, *P_2B_-ATPase* genes were most abundant, while *P_2A_-ATPase* genes were least abundant. Interestingly, the member of P_5_ subfamily was very few in the five plants, only one or two genes (Table S2).

### 2.4. Evolutionary Relationship Analysis of P-ATPase Genes

**Figure 3:**
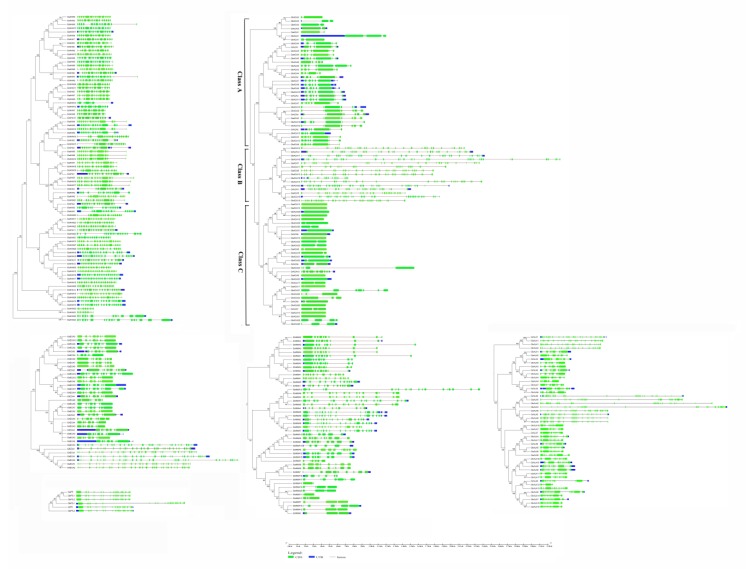
Phylogenetic tree and structure of *P-ATPase* genes in *G. arboretum*, *G. raimondii*, *G. hirsutum* and *G. barbadense*. Green boxes, blue boxes and gray lines represent exons, UTRs and introns, respectively.

To gain insight into the evolutionary relationship of *P-ATPase* genes in *G. arboretum*, *G. raimondii*, *G. barbadense* and *G. hirsutum*, a phylogenetic tree was constructed according to the protein sequences of the *P-ATPase* genes in four cotton species, and the *P-ATPase* gene structures were analyzed with their CDS sequences using the GSDS online tool [18]. The results showed that most *P-ATPase* genes contained introns and exons, and most *P-ATPase* genes in *G. raimondii* and *G. barbadense* also contained untranslated regions (UTRs). Almost all P_3A_ subfamily genes contained 10–20 introns, the P_1B_ subfamily genes contained 5–20 introns, and most P_2A_ subfamily genes contained 5–10 introns, but some genes contained more than 20 introns (Figure 3). Interestingly, the structures of the P_1B_ subfamily genes on different branches had significant differences. Most genes in class C did not contain introns or contained only one, and most genes in class A contained 5–7 introns, but all genes in class B contained more than 30 introns. In the phylogenetic tree, the gene structures of most paralogous gene pairs between *G. hirsutum* and *G. arboretum*/*G. raimondii* were very similar, which suggests that their genetic relationship is close, and these paralogous genes had high homology. However, the gene structures of paralogous gene pairs between *G. barbadense* and *G. arboretum*/*G. raimondii* were not close, which indicates that *G. hirsutum* has a closer genetic relationship to *G. arboretum*/*G. raimondii* than *G. barbadense*.

**Figure 4:**
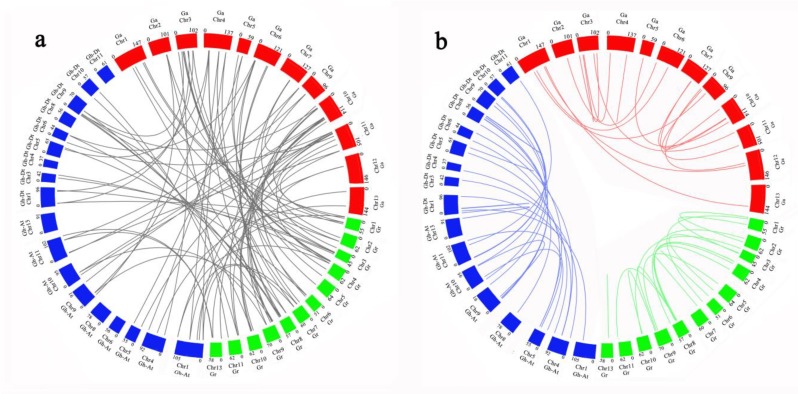
Collinearity analysis of P-ATPase genes in *G. hirsutum*, *G. arboreum*, and *G. raimondii*.; (**a**) Duplication between *G. hirsutum*, *G. arboreum*, and *G. raimondii*, (**b**) Duplication within *G. hirsutum*, *G. arboreum*, and *G. raimondii*. Red, blue and green rectangles represent chromosomes of three cotton species. Blue, orange and gray lines represent genetic duplication in three cotton species themselves, and green lines indicate genetic duplication between different cotton species.

To further analyze the evolutionary relationship of *P-ATPase* genes in *G. hirsutum*, *G. arboretum* and *G. raimondii*, the collinearity of *P-ATPase* genes was drawn, using Circos software, between different cotton species. Twenty-one pairs of orthologous genes were identified between *G. hirsutum* and *G. arboretum*, and 16 pairs of orthologous genes were identified between *G. hirsutum* and *G. raimondii*. The results showed significant collinear relationships between *G. hirsutum* and *G. arboretum* or *G. raimondii*, and *G. hirsutum* was more closely related to *G. arboretum* than *G. raimondii* (Figure 4 and Table S3). In addition, the remaining genes in *G. hirsutum* do not have duplicative relationships with those in *G. arboretum* and *G. raimondii*, indicating that independent duplications might occur in *G. hirsutum*.

### 2.5. Expression Patterns of P-ATPase Genes in G. hirsutum

**Figure 5:**
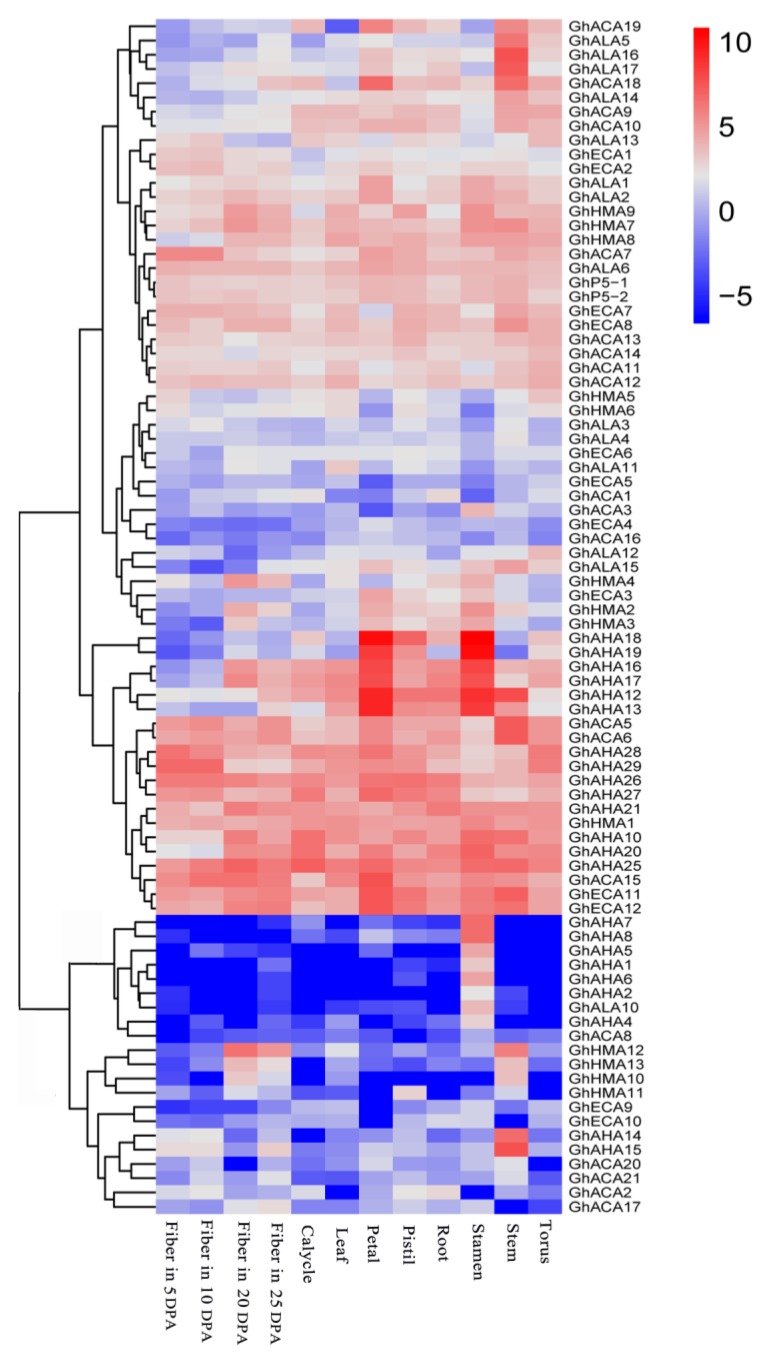
Expression patterns of *P-ATPase* genes in *G. hirsutum*. Different colors represent expression level; –5 and 10 indicates the expression level, different colors represent expression level; red indicates high expression, white indicates low expression, and blue indicates not expressing.

P-ATPase plays a variety of roles in plant cells. Thus, to realize the function of P-ATPase genes in *G. hirsutum*, the expression of *P-ATPase* genes in the different tissues (leaf, petal, pistil, root, stamen, stem and torus) and fibers was analyzed at various developmental stages (5 DPA,10 DPA, 20 DPA and 25 DPA). Among the 98 genes, 84 *P-ATPase* genes were at least expressed in one tissue or at one developmental stage of fiber because the value of log_2_^FPKM^ was more than 1. The heat map was plotted using R software. The results indicated that 84 *P-ATPase* genes might play a role at various growth stages of cotton, and the remaining 14 genes might be expressed in a special environment or were only pseudogenes. More than one-third of the 84 *P-ATPase* genes were widely expressed in the various tissues of cotton and at three developmental stages of cotton fiber, which suggested that these genes may participate in the whole growth and fiber development of cotton (Figure 5). In addition, some genes were highly expressed in the petal and pistil or stamen, suggesting that these genes may play a role in flower development. A few genes had higher expression at the three developmental stages of cotton fibers compared to other tissues, indicating that these genes are involved in the regulation of cotton fiber development. In the stem, leaf, and root, a few genes were highly expressed, showing that these genes may be involved in stress resistance. Interestingly, seven *P-ATPase* genes (*GhAHA1, 2, 4–8*) were only expressed in the stamen, suggesting that these genes are associated with a specific role in the development of the cotton stamen (Figure 5 and Table S4). A significantly different expression pattern in *P-ATPase* members in the same subfamily was observed, signifying that these genes performed functional differentiation, while a similar expression pattern of some genes from different subfamilies was observed, which showed that these genes may have a kind of synergy.

### 2.6. Accumulation of PAs and Expression Analysis of P_3A_-ATPase Genes in Brown Cotton.

**Figure 6:**
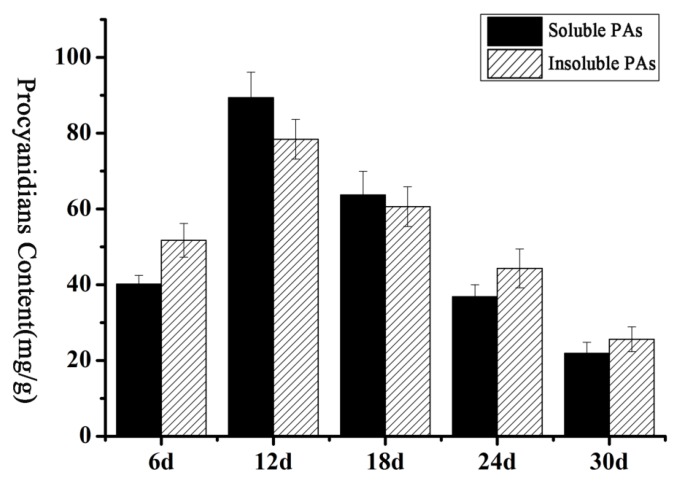
PA content at different development stages of brown cotton fibers. Abscissa indicates different days post anthesis of cotton fibers, and ordinate indicates PA content, the error bars indicate SE.

**Figure 7:**
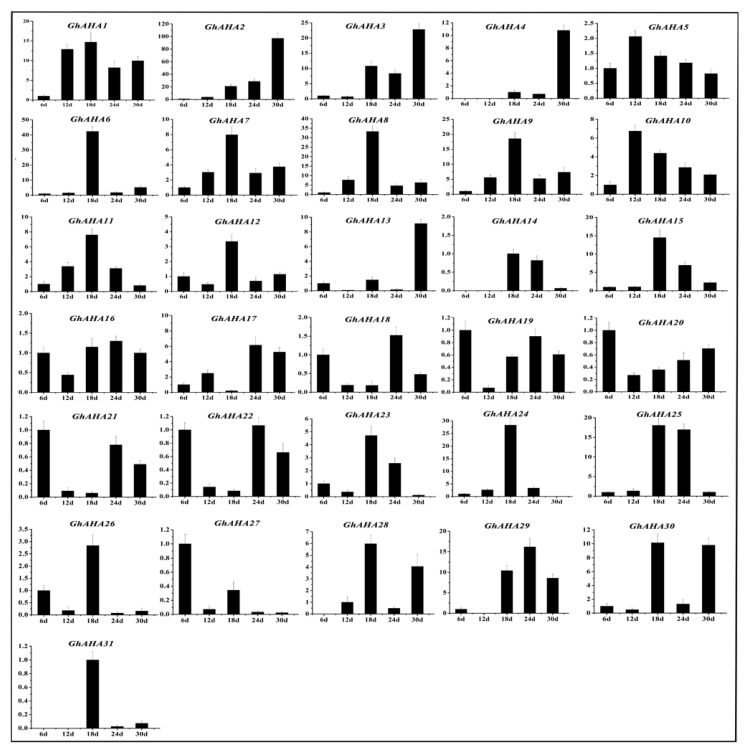
Relative expression levels of the *P_3A_-ATPase* genes at different development stages of brown cotton fiber. The relative expression level was calculated using the 2^−^^△△Ct^ method, the error bars indicate SE.

Some *P_3A_-ATPase* genes were found in *Arabidopsis* and petunia, such as *TT13* and *PH5* [9,10], which were related to the accumulation of PAs. Thus, we explored whether such genes are present in brown cotton. First, the contents of PAs at different development stages of brown cotton fiber were measured. As shown in Figure 6, the contents of PAs increased gradually with the development of fiber, reached the highest level at 12 DPA, and thereafter decreased gradually. The accumulation of PAs showed a trend that increased initially and then decreased gradually back to baseline. To find the genes in the P_3A_ subfamily associated with the accumulation of PAs in brown cotton fiber, primers were designed according to the sequences of *P_3A_-ATPase* genes, and qRT-PCR was performed with the fibers at 6, 12, 24, and 30 DPA in brown cotton, respectively. The results suggested that the relative expression levels of most *P_3A_-ATPase* genes were not consistent with the accumulation of PAs in brown cotton fibers. However, *GhAHA5*, *GhAHA 7*, *GhAHA 9*, *GhAHA10* and *GhAHA11* showed a relative expression tendency similar to the PAs’ accumulation (Figure 7). Therefore, we hypothesized that one or more of these five genes may be related to the accumulation of PAs.

## 3. Discussion

In recent years, many reports have described the structure and function P-ATPase genes [9,19,20], but a comprehensive study of their roles is still lacking in cotton. In this study, we identified and analyzed the P-ATPase gene family in *G. arboretum*, *G. raimondii*, *G. barbadense* and *G. hirsutum* at the genomic level. In all, 53, 51, 99 and 98 P-ATPase genes were identified in *G. arboretum*, *G. raimondii*, *G. barbadense* and *G. hirsutum*, respectively. The rich members and their high homology determined the diversity of expression and the specificity of function for the P-ATPase gene family. The homologous genes of various P-ATPases have been isolated from various plants. For example, *AtECA1-4* can be expressed in all tissues of *Arabidopsis*. These genes play a role in the entire growth process of *Arabidopsis*, such as transporting calcium or manganese ions [21]. In upland cotton, there were similar expression of P_2A_ genes, *GhECA1*, *2*, *7*, *8*, *11*, *12* were expressed in all tissues, they might play a similar function like *AtECA1-4*. In plants, some *P-ATPase* genes showed tissue-specific expression, suggesting they have specific function in a tissue. In *Arabidopsis*, the *AHA3* is only expressed in phloem cells [22]; the expression pattern of *AHA2*, *AHA9* and *AHA10* had well defined the interaction/role in root hair, anther and developing seeds, respectively [23,24]; In rice, *OsHMA3*, *OsHMA4* and *OsHMA5* have high expression in root [25,26,27]; In Hevea brasiliensis, *HbHA2* and *HbHA4* showed high transcript abundance in latex [28]; in tomato, *SlHA5, 6, 7,* show tissue-specific pattern, its transcripts can only be detected in flowers [29]; In *Arabidopsis*, *ALA10* is only expressed in guard cells and root epidermal cells [30]. These P-ATPase genes have been shown to play a role in specific tissues. The expression patterns of P-ATPase in upland cotton showed that some genes had obvious tissue-specific expression. For instance, *GhAHA1, 2, 4–8* were expressed in stamen *and GhAHA14*, *GhAHA15* had a specific expression in stem, suggesting these genes may play a role in stamen or stem, like P3A genes of *Arabidopsis* in different tissues. Some genes of other subfamilies also showed tissue-specific expression. In all, the tissue-specific expression indicates that the P-ATPase genes play multiple functions in upland cotton. Kutschera et al. (2004) [31] found that three P-ATPase genes form different subfamilies and could be expressed in the same type of cells at the same development stage. Interestingly, the similar expression patterns in upland cotton were found, *GhAHA2* and *GhALA10* were only expressed in stamens, *GhAHA21* and *GhHMA1* showed similar expression patterns in different tissues, suggesting these genes may have similar or synergistic effects in specific tissue. In addition, the expression of some P-ATPase genes in specific tissues was also influenced by different developmental stages. For example, tobacco pma1 is mainly expressed in the guard cells of seedling cotyledons and stems, whereas the pma1 gene in fruit is only expressed in the phloem, and pma6 is mainly expressed in the vegetative phase of trichomes [32]. In upland cotton, *GhHMA12* and *GhHMA13* showed a similar expression in developmental fiber, which had an expression level at 20 and 25 DPA, but little expression at 5 and 10 DPA (Figure 5). Upland cotton, as an important economic crop in the world, had complex gene regulation networks. Many P-ATPases have been shown to participate in regulating growth and physiological processes in different plants. The analysis of *P-ATPases* expression patterns in upland cotton showed that vast majority of *P-ATPases* had a certain function in cotton growth and development. Combining with the tissue-specific expression and functional analysis of *P-ATPases* in other plants, the potential functions of some *P-ATPases* in upland cotton could be inferred, such as regulating root and fiber development, and participating in some abiotic stresses response, etc.

Interestingly, in brown cotton fiber, the expression patterns of major *P_3A_-ATPase* genes by qRT-PCR were found to be inconsistent with the expression pattern of *P_3A_-ATPase* genes in upland cotton (Figure 5). The material used for fluorescence quantification experiments was brown cotton fiber, and white cotton was used for transcriptome analyses. We speculated that the differences in cotton varieties may lead to the changes in expression patterns of P_3A_ genes, and the difference in the environment may also be the cause of the changes.

In *Arabidopsis* and rice, the P-ATPase gene family can be divided into six subfamilies: P_1B_, P_2B_, P_2A_, P_3A_, P_4_, and P_5_. [8]. Phylogenetic analysis showed that the *P-ATPases* genes of four cotton species could be also clustered into the same six subfamilies. There was only one or two P_5_ members in four cotton species, which were similar with *Arabidopsis* and rice. Gilbert (1987) believed that there is a relationship between intron structure and gene evolution [33]. There are two different hypotheses about the origin of introns. One posits that the gene encoding the protein is initially discontinuous, namely that introns were present at the beginning, before the gene encodes the protein; and the other speculation is that the gene encoding the protein is continuous, and thus, there is no intron while the gene encodes the protein [34]. Cao et al. (2016) showed that the original genes had few introns, and with the ongoing replication of genes, intron number gradually increased [35]. The study also indicated that some introns were already present, and others were generated or increased with the insertion of transposons during gene duplication. By comparing the exon and intron distributions of *P-ATPase* genes in diploid and tetraploid cotton genotypes, most *P-ATPase* genes on the same branch were found to have a nearly identical structure, which suggested that the evolution of cotton species was highly conserved. In addition, the *P-ATPase* genes on different branches or the same branches in diploid and tetraploid cotton species were found to have some structural diversity, indicating that the structural diversity of the *P-ATPase* genes ensured that some new functional genes appeared in the evolution of the P-ATPase gene family.

In a previous study, it was suggested that proanthocyanidin is synthesized in the cytoplasm, transported into vacuoles by transporters, and is then accumulated in vacuoles [36]. The *P-ATPase* genes play important roles during this process. First, H^+^-ATPase decomposes ATP and releases energy to generate and maintain an electrochemical gradient of H^+^ on both sides of the cell membrane and to provide energy to transporters for the transmembrane transport of PAs. Second, H^+^-ATPase is used to adjust cell pH [3,4]. A relatively stable pH can maintain cell activity and affect the activity of H^+^-ATPase [37]. pH can affect the accumulation of PAs in the vacuole, whether it is too high or too low. Appelhagen et al. (2015) showed that *TT13* played an important role in the accumulation of PAs in the *Arabidopsis* seed coat [9]. When *TT13* is mutated, PAs will not accumulate in the vacuoles of the seed coat. Some researchers found that vacuole pH increased, and the petal color changed from red to purple color when the expression of *PH5* was reduced in petunia [19,20]. In the present study, it is speculated that *P_3A_-ATPase*, the homologous gene to *PH5*, may also play an important role in the PAs biosynthesis of brown cotton fiber. Xiao et al. (2007) analyzed the expression levels of five key genes (*CHI, F3H, DFR, ANS* and *ANR*) participating in PAs synthesis pathways at different developmental stages of brown cotton fiber and found that these genes have higher expression levels in the early stage of fiber development. After reaching the peak, the expression began to gradually decrease [38]. This result is consistent with the accumulation of PAs during the development of brown cotton fibers. We speculate that if there are one or more P_3A_ genes involved in the proanthocyanidin synthesis pathway, they might have an expression trend similar to the accumulation of PAs. Fluorescence quantitative analysis showed that the trend of relative expression of *GhAHA5*, *GhAHA7*, *GhAHA9*, *GhAHA10*, and *GhAHA11* in brown cotton fiber was similar to that for PAs accumulation (Figure 6 and Figure 7), especially the changes in expression levels of *GhAHA10* and *GhAHA11* were basically consistent with changes in the accumulation of PAs. Therefore, it was speculated that one or more of these genes may affect the accumulation of PAs in cotton fiber. Other P_3A_ genes may also have certain functions in the development of fibers, such as *GhAHA6* and *GhAHA24*, which had the highest expression at 18 DPA; they may play a role in the stage of fiber development.

## 4. Materials and Methods

### 4.1. Genomic Data Sources

The genome data for *Arabidopsis* were downloaded from The *Arabidopsis* Information Resource (http://www.arabidopsis.org/). The genome data for four cotton species were obtained from their genomes’ respective sequence websites: *Gossypium raimondii* from the Phytozome database (http://www.phytozome.net/); *Gossypium arboreum* and *Gossypium hirsutum* from the Cotton Genome Project (http://cgp.genomics.org.cn); and *Gossypium barbadense* from the Cotton Crop Databases (http://cotton.cropdb.org).

### 4.2. Sequence Identification of P-ATPase Genes

First, we used DNATOOLS software to establish four local databases with the whole genome amino acid sequences of *G. arboretum*, *G. raimondii*, *G. barbadense* and *G. hirsutum*. Then, we used the amino acid sequence of *Arabidopsis P-ATPase* gene (*AHA10*)-specific domains as a query sequence to preliminarily identify *P-ATPase* genes in four local databases using DNAtools software (*E*-value < 0.001) [39]. These candidate genes were examined to determine whether they contained the *P-ATPase* domains using the PFAM (http://pfam.wustl.edu/hmmsearch.shtml) [16] and SMART databases (http://smart.embl-heidelberg.de) [17].

### 4.3. Phylogenetic Analysis of P-ATPase Genes

These *P-ATPase* sequences were aligned using the ClustalW tool [40] of MEGA 5.0 software to remove repeat sequences, and the phylogenetic tree of *Arabidopsis* and four cotton species was constructed using the Neighbor-Joining method (Bootstrap = 1000) to analyze the evolutionary relationships of the P-ATPase gene family [41]. The naming and classification of *P-ATPase* genes in cotton refers to *Arabidopsis P-ATPase* genes [7].

### 4.4. Amino Acid Sequence Analysis of Cotton P-ATPase Genes 

To analyze the basic properties and characteristics of cotton *P-ATPase* genes, the data for isoelectric point, protein molecular weight and amino acid length were obtained using the ExPAsy (http://www.expasy.org) online tools and the Cotton Functional Genomics Database (https://cottonfgd.org/) [42]. The basic structure of P-ATPase genes was analyzed using the GSDS online tool (http://gsds.cbi.pku.edu.cn) [18].

### 4.5. Chromosome Localization and Gene Duplication of Cotton P-ATPase Genes

The genome annotation information for *G. arboretum*, *G. hirsutum* and *G. raimondii* was downloaded from the Phytozome database (http://www.phytozome.net/) and the Cotton Genome Project (http://cgp.genomics.org.cn). The syntenic information was obtained using OrthoMCL (http://orthomcl.org/orthomcl/) [43]. The chromosome localizations and syntenic blocks were mapped using MapInspect (http://mapinspect.software.informer.com) and Circos software [44].

### 4.6. Expression Patterns of P-ATPase in G. hirsutum

The RNA-seq data derived from the TM-1 transcriptome of the Cotton Functional Genomics Database (https://cottonfgd.org/) (Zhang et al., 2015) were used to analyze *P-ATPase* gene expression profiles in *G. hirsutum* [15], which included the expression analyses of *P-ATPase* genes in various tissues and fibers at different development stages. If the value of log_2_^FPKM^ was more than 1, we considered the gene expressed, and if the value was less than or equal to 1, we considered it not expressed [45]. R software was used to visualize the expression patterns with normalization method.

### 4.7. qRT-PCR of P_3A_-ATPase Genes in G. hirsutum

The PCR primers were designed according to the sequences of *P_3A_-ATPase* genes (Table S3). The RNA of brown cotton fibers at 6 days post-anthesis (DPA), 12 DPA, 18 DPA, 24 DPA and 30 DPA was extracted with an RNA extraction kit (TransGen Biotech, Beijing, China) and used to perform qRT-PCR. The total volume was 20 μL, including 10 μL of SYBR^®^Premix Ex Taq ™ II (2x), 2 μL of cDNA, and 0.8 μL of upstream and downstream primers. The reaction procedure was as follows: 50 °C for 2 min, 95 °C for 30 s, 40 cycles of 95 °C for 15s, 60 °C for 20 s and 68 °C for 20 s, and 4 °C for storage. The UBQ7 gene was used as an internal reference gene [46]. For each experiment, three biological replicates were executed, and the relative expression levels were calculated using the 2^−^^△△Ct^ method [47].

### 4.8. Determination of PA Content

According to methods from Ikegami [48], the soluble and insoluble PAs of brown cotton fibers at different developmental stages (6 DPA, 12 DPA, 18 DPA, 24 DPA, and 30 DPA) were extracted, and the content of PAs was determined by spectrophotometry according to the standard curve of catechins, which were used as controls [49]. For each experiment, three biological replicates were executed.

## 5. Conclusions

In the study, a total of 301 *P-ATPase* genes were identified according to the whole genome sequences of *G. arboretum*, *G. raimondii*, *G. barbadense* and *G. hirsutum* using TBlastN, PFAM, and ClustalW tools, and the isoelectric point and molecular weight of 301 genes were predicted. The phylogenetic and structural analyses of *P-ATPase* genes in four cotton species showed that most genes located in the same branch of the evolutionary tree had a similar structure, and the numbers of exons and introns contained in the genes were also similar. The expression pattern analysis of *P-ATPase* genes in *G. hirsutum* indicated that most genes participated in the growth and development of cotton. In addition, the qRT-PCR analyses suggested that some members of the P_3A_ subfamily in brown cotton might participate in the transport and accumulation of PAs. The function of *P_3A_-ATPase* genes in transporting the pigment of brown cotton fiber was initially explored, laying the foundation for selecting a functional *P-ATPase* gene and breeding a new brown cotton variety.

## Supplementary Materials

The following are available online. Table S1: Characteristics of 301 P-ATPase genes in *G. raimondii*, *G. hirsutum*, *G. arboreum* and *G. barbadense*. “At” and “Dt” represent Group A and Group D chromosomes in *G. hirsutum*, respectively, and “sca” indicates that there is no chromosomal location information for the gene. Table S2: Gene numbers of 5 P-ATPase subfamilies in *Arabidopsis*, *G. raimondii*, *G. hirsutum*, *G. arboreum* and *G. barbadense*. Table S3: Homologous analyses of *P-ATPase* genes between different cotton species. Table S4: The RNA-seq data of P-ATPases expression patterns in upland cotton. Table S5: Primers used in qRT-PCR.
