# Supplementary material for: Genomic Comparison of the P-ATPase Gene Family in Four Cotton Species and Their Expression Patterns in Gossypium hirsutum
Source: Molecules. 2018 May 5;23(5):1092. doi: 10.3390/molecules23051092 (PMC6102550; doi:10.3390/molecules23051092)
Supplement: Supplementary file 1 [file molecules-23-01092-s001.zip › Supplementary files/Protein sequences of four cotton species and Arabidopsis.docx]

>GbAHA14

MNEELDKPLLDPENFNREGIDLERLPLEEVFEQLRTSRAGLTSEDAEVRVHIFGQNKLEEKPENKFLKFLSFMWNPLSWVMEAAAVMAIVLANGGGEGPDWQDFVGIICLLILNSTISFIEENNAGNAAAALMARLAPKTKVLRDGQWQERDAAILVPGDIISIKLGDIIPADARLLEGDPLKIDQSALTGESLPVTKRTGDEVFSGSTCKHGEIEAVVIATGVHSFFGKAAHLVDSTEVVGHFQQVLTSIGNFCICSIAVGMVLEIIVMFPIQHRSYRDGINNLLVLLIGGIPIAMPTVLSVTLAIGSHRLSQQGAITKRMTAIEEMAGMDVLCSDKTGTLTLNRLTVDRNLVEVFSKNMDKDLIILLAARASRLENQDAIDAAIINMLADPKEARANIKEVHFLPFNPVDKCTAITYIDSDGNWYRASKGAPEQILNLCLEKDLIAGRVHAIIDKFAERGLRSLGVAFQEIPERTKESPGGPWTFCGLLPLFDPPRHDSAETIRRALNLGVDVKMITGDQLAIAKETGRRLGMGTNMYPSSSLLGREKDENEALPVDELIEKADGFAGVFPEHKYEIVKILQEKKHVVGMTGDGVNDAPALKKADIGIAVADATDAARSAADIVLTEPGLSVIISAVLTSRAIFQRMKNYTIYAVSITIRIVLGFVLLALIWEYDFPPFMVLIIAILNDGTIMTISKDRVRPSPTPDSWKLNEIFATGVVIGTYLALVTVLFYWIVIDTDFFETHFNVRSISDDTEQISSAVYLQVSIISQALIFVTRSRSWSFVERPGVLLMCAFMVAQLVATLIAVYAHISFADISGIGWGWAGVIWLYSLVFYVPLDIIKFTVRYALSGEAWNLLFDRKVSVLFLVSYLIGFKSFLEEAFHGIPVQQTAFTSKKDYGKDDRAAQWILSQRSLQGLMAADLDFNGRRSRSSLIADHARRRAEIARLGELHTLRGHVESVMRLKNLDLNAIKSAHTV

>GbAHA15

MNEELDKPLLDPENFNREGIDLERLPLEEVFEQLRTSRAGLTSEDAEVRVHIFGQNKLEEKPENKFLKFLSFMWNPLSWVMEAAAVMAIVLANGGGEGPDWQDFVGIICLLILNSTISFIEENNAGNAAAALMARLAPKTKVLRDGQWQERDAAILVPGDIISIKLGDIIPADARLLEGDPLKIDQSALTGESLPVTKRTGDEVFSGSTCKHGEIEAVVIATGVHSFFGKAAHLVDSTEVVGHFQQVLTSIGNFCICSIAVGMVLEIIVMFPIQHRSYRDGINNLLVLLIGGIPIAMPTVLSVTLAIGSHRLSQQGAITKRMTAIEEMAGMDVLCSDKTGTLTLNRLTVDRNLVEVFSKNMDKDLIILLAARASRLENQDAIDAAIINMLADPKEARANIKEVHFLPFNPVDKCTAITYIDSDGNWYRASKGAPEQILNLCLEKDLIAGRVHAIIDKFAERGLRSLGVAFQEIPERTKESPGGPWTFCGLLPLFDPPRHDSAETIRRALNLGVDVKMITGDQLAIAKETGRRLGMGTNMYPSSSLLGREKDENEALPVDELIEKADGFAGVFPEHKYEIVKILQEKKHVVGMTGDGVNDAPALKKADIGIAVADATDAARSAADIVLTEPGLSVIISAVLTSRAIFQRMKNYTIYAVSITIRIVLGFVLLALIWEYDFPPFMVLIIAILNDGTIMTISKDRVRPSPTPDSWKLNEIFATGVVIGTYLALVTVLFYWIVIDTDFFETHFNVRSISDDTEQISSAVYLQVSIISQALIFVTRSRSWSFVERPGVLLMCAFMVAQLVATLIAVYAHISFADISGIGWGWAGVIWLYSLVFYVPLDIIKFTVRYALSGEAWNLLFDRKTAFTSKKDYGKDDRAAQWILSQRSLQGLMAADLDFNGRRSRSSLIADHARRRAEIARLGELHTLRGHVESVMRLKNLDLNAIKSAHTV

>GbAHA20

KKETDYHDFVGILALLLINSTISFIEENNAGNAAAALMARLAPKAKVLRDGKWNEEDASVLVPGDIISIKLGDIIPADARLLQGDPLKIDQSALTGESLPVTKHPGDGVYSGSTCKQGEIEAVVIATGVHTFFGKAAHLVESTTHVGHFQQVLTSIGNFCICSIAIGMLAELIVIYGAQRRSYRTGIDNLLVILIGGIPIAMPTVLSVTMAIGSHRLSQQGAITKRMTAIEEMAGMDVLCSDKTGTLTLNKLTVDKNLIEVFVNNVDKDTVILMAARASRLENQDAIDTAIVAMLADPKEARAGITEVHFLPFNPTDKRTALTYVDEAGKMHRVSKGAPEQILNLAYNKSEIGKKVHSIIDKYAERGLRSLAVARQEVPAGTKDSPGGPWEFVGLLPLFDPPRHDSAETIRRALDLGVSVKMITGDQLAIGKETGRRLGMGTNMYPSSSLLGDHKNEEIGAFSIDELIENADGFAGVFPEHKFEIVKRLQAKKHIVGMTGDGVNDAPALKKADIGIAVADSTDAARSASDIVLTEPGLSVIISAVLTSRAIFQRMKNYTIYAVSITIRIVLGFMLLTLIWRLNFPPFMVLIIAILNDGTIMTISKDRVKPSPLPDSWKLKEIFATGIVLGSYLALMTLIFFWAAYETDFFPDKFGVRSLSKKSFDLTQESERRKVNALLSSAVYLQVSTISQALIFVTRSRGWSFTERPGLLLVTAFIIAQLVATVISAQATWSFAGIRAVGWGWCGVIWIYNILTYFLLDPIKFAVRYALSGKAWDLVLNQRTAFSTQKDFGKEAREAAWAAEQRTLHGLQSISDAKITEKHNFRDISVMAEEARRRAEIARLREIHTLKGKVESFAKLRGLDIDVNPHYTLLTYCVLIANQFMVWSMAMEHTSTLQVECLNWNREVVLVSSSDVQYYWAPLTCLVQNFGSFCNCLLSESIQVTAVRHLPDNPAYSDDERLDSAAMSLTAESEEEDGVTDHHLLTIPNSNVAFLKEKTVWLTREL

>GbAHA21

MWNPLSWVMEAAAVMAIALAHGGKKETDYHDFVGILALLLINSTISFIEENNAGNAAAALMARLAPKAKVLRDGKWNEEDASVLVPGDIISIKLGDIIPADARLLQGDPLKIDQSALTGESLPVTKHPGDGVYSGSTCKQGEIEAVVIATGVHTFFGKAAHLVESTTHVGHFQQVLTSIGNFCICSIAIGMLAELIVIYGAQRRSYRTGIDNLLVILIGGIPIAMPTVLSVTMAIGSHRLSQQGAITKRMTAIEEMAGMDVLCSDKTGTLTLNKLTVDKNLIEVFVNNVDKDTVILMAARASRLENQDAIDTAIVAMLADPKEARAGITEVHFLPFNPTDKRTALTYVDEAGKMHRVSKGAPEQILNLAYNKSEIGKKVHSIIDKYAERGLRSLAVARQEVPAGTKDSPGGPWEFVGLLPLFDPPRHDSAETIRRALDLGVSVKMITGDQLAIGKETGRRLGMGTNMYPSSSLLGDHKNEEIGAFSIDELIENADGFAGVFPEHKFEIVKRLQAKKHIVGMTGDGVNDAPALKKADIGIAVADSTDAARSASDIVLTEPGLSVIISAVLTSRAIFQRMKNYTIYAVSITIRIVLGFMLLTLIWRLNFPPFMVLIIAILNDGTIMTISKDRVKPSPVPDSWKLKEIFATGIVLGSYLALMTVIFFWAAYETDFFPDKFGVRSLSKKSFDLKQESERRKVNALLSSAVYLQVSTISQALIFVTRSRGWSFTERPGLLLVTAFIIAQLVATVISAQATWSFAGIRAVGWGWCGVIWIYNILTYFLLDPIKFAVRYALSGKAWDLVLNQRTAFSTQKDFGKEAREAAWAAEQRTLHGLQSTSDAKITEKHNFRDISVMAEEARRRAEIARLREIHTLKGKVESFAKLRGLDIDVNPHYTV

>GbAHA8

MAETSVGLEDIKNETIDLERIPVHEVFQKLKCTRDGLSSEEGQKRLHIFGPNKLEEKQESKVLKFLGFMWNPLSWVMEIAAIMAIALANGGGKPPDWQDFIGIVALLLINSTISFIEENNAGNAAAALMAGLAPKTKVLRDGKWSEQEASILVPGDIISIKLGDIVPADARLLEGDPLKIDQSALTGESLPVNKNAGDEVFSGSTVKQGELEAVVIATGVHTFFGKAAHLVDSTNNVGHFQQVLTAIGNFCICSIGVGMLIEIVVMYPIQQRRYRDGIDNLLVLLIGGIPIAMPTVLSVTMAIGSHRLSQQGAITKRMTAIEEMAGMDVLCSDKTGTLTLNKLTVDKSLVEVFTNDVDKDMVILLAARASRVENQDAIDACIVGMLGDPKEARAGITEVHFFPFNPVDKRTAMTYIEADGSWHRVSKGAPEQIIDLCNLRDDVKRRAHDIIANFADRGLRSLAVARQTVKEKNKDAQGEAWEFVGILPLFDPPRHDSAETICRALNLGVNVKMITGDQLAIGKETGRRLGMGTNMYPSSALLGQNKGDTIDTIGVDELIEKADGFAGVFPEHKYEIVKRLQQRNHICGMTGDGVNDAPALKKADIGIAVDDATDAARGASDIVLTEPGLSVIVSAVLTSRAIFQRMKNYTIYAVSITIRIVLGFMLIALIWKFDFSPFMVLIIAILNDGTIMTISKDRVKPSPMPDSWKLKEIFATGIVLGTYLACMTVVFFWAANDSNFFQDKFGVRSIRHNQDELTAAIYLQVSIVSQALIFVTRSRSWSFIERPGLLLVVAFILAQLIATLLAVYANWGFARIKGIGWGWAGVIWLYSLVFYVPLDVLKFLIRYSLSGKAWDNLLQSKTAFTTKKDYGKGEREAQWAMAQRTLHGLTPAEMTQLYNEETNYRELSEIAEQARKRAEVARLRELHTLKGHVESVVKLKGLDIDTIQQHYTV

>GbAHA18

MGDKNEVLEAVLKETVDLENIPIEEVFENLRCSREGLTTEAAEERLTIFGHNKLEEKKESKFLKFLGFMWNPLSWVMEAAAIMAIALANGGGKPPDWQDFVGIITLLVINSTISFIEENNAGNAAAALMARLAPKAKVLRDGRWNEQDAAILVPGDIISIKLGDIIPADARLLEGDPLKIDQSALTGESLPVTKGPGDGIYSGSTCKQGEVEAVVIATGVHTFFGKAAHLVDTTNQVGHFQKVLTAIGNFCICSIAVGMVIEIIVMYPIQDRDYRPGIDNLLVLLIGGIPIAMPTVLSVTMAIGSHRLSQQGAITKRMTAIEEMAGMDVLCSDKTGTLTLNKLTVDKNLIEVFAKGVDADTVVLMAARASRTENQDAIDSAIVGMLADPKEARAGIREVHFLPFNPTDKRTALTYIDSDGKMHRVSKGAPEQILHLAHNKTDIERRVHAVIDKFAERGLRSLAVAYQEVPDGRKESPGGPWQFIGLMPLFDPPRHDSAETIRRALNLGVNVKMITGDQLAIGKETGRRLGMGTNMYPSSALLGQDKDESIAALPVDELIEKADGFAGVFPEHKYEIVKRLQARKHICGMTGDGVNDAPALKKADIGIAVADATDAARSASDIVLTEPGLSVIISAVLTSRAIFQRMKNYTIYAVSITIRIVLGFMLLALIWKFDFPPFMVLIIAILNDGTIMTISKDRVKPSPLPDSWKLAEIFTTGIVLGSYLAVMTVIFFWAAYKTNFFPRVFGVATLEKTAHDDIKKLASAVYLQVSIISQALIFVTRSRSWSFVERPGLLLLAAFVIAQLIATLIAVYANWSFAAIEGIGWGWAGVIWLYNIIFYIPLDFIKFFIRYALSGRAWDLVIEQRIAFTRQKDFGKEQRELQWAHAQRTLHGLQAPDTKMFTERTHFTELNQMAEEAKRRAEIARLRELHTLKGHVESVVRLKNLDIDTIQQAYTV

>GbAHA9

MWNPLSWVMEFAAIMAIALANGGGKPPDWQDFIGIVSLLFINSTISFIEENNAGNAAAALMAGLAPKTKVLRDGKWNEQEAAILVPGDIISIKLGDIVPADARLLEGDSLKIDQSALTGESLPVNKHSGDEVFSGSTVKQGEIEAVVIATGVHTFFGKAAHLVDSTNNVGHFQQVLTAIGNFCICSIGVGMLIEIVVMYPIQRRKYRDGIDNLLVLLIGGIPIAMPTVLSVTMAIGSHRLSQQGAITKRMTAIEEMAGMDVLCSDKTGTLTLNKLTVDKSMIEVFMDNIDKEMVLLLAARASRVENQDAIDACIVGMLGDPKEARAGVTEVHFFPFNPVDKRTAMTYIEADGSWHRVSKGAPEQIIELCNLRNDAKRRAHDIITKFADRGLRSLAVAKQKVPEKTKDGQGDPWQFVGLLPLFDPPRHDSAETIRGALSLGVNVKMITGDQLAIGKETGCRLGMGTNMYPSSVLFGENKGDALDTIGVDELIEKADGFAGVFPEHKYEIVKRLQQRKHICGMTGDGVNDAPALKKADTGIAVDDATDAARSASDIRMKNYTIYAVSITIRIVLGFMLLALIWKFDFSPFMVLIIAILNDGTIMTISKDRVKPSPMPDSWKLKEIFCTGIVLGTYLACMTVVFFWAANDSNFFSDKFGVRSIRHNQDELTAAVYLQVSIVSQALIFVTRSRSWSFIERPGFLLVIAFILAQLVATVIAVYANWGFARIKGIGWGWAGVIWIYSVVFYFPLDVIKFLIRYAMSGKAWNNLLQNKTAFTTKKDYGKGEREAQWALAQRTLHGLTPPEITEKSNYIELSEIAEQARKRAEVARLRELHTLKGHVESVVKLKGLDIDTIQQHYTV

>GbAHA10

MADKSISLEEIKNETVDLERIPVEEVFQQLKCTRNGLTSEEGQKRLQIFGPNKLEEKKAKENKVLKFLGFMWNPLSWVMEFAAIMAIALANGGGKPPDWQDFIGIVSLLFINSTISFIEENNAGNAAAALMAGLAPKTKVLRDGKWNEQEAAILVPGDIISIKLGDIVPADARLLEGDSLKIDQSALTGESLPVNKHSGDEVFSGSTVKQGEIEAVVIATGVHTFFGKAAHLVDSTNNVGHFQQVLTAIGNFCICSIGVGMLIEIVVMYPIQRRKYRDGIDNLLVLLIGGIPIAMPTVLSVTMAIGSHRLSQQGAITKRMTAIEEMAGMDVLCSDKTGTLTLNKLTVDKSMIEVFMDNIDKEMVLLLAARASRVENQDAIDACIVGMLGDPKEARAGVTEVHFFPFNPVDKRTAMTYIEADGSWHRVSKGAPEQIIELCNLRNDAKRRAHDIITKFADRGLRSLAVAKQKVPEKTKDGQGDPWQFVGLLPLFDPPRHDSAETIRGALSLGVNVKMITGDQLAIGKETGRRLGMGTNMYPSSVLLGENKGDALDTIGVDELIEKADGFAGVFPEHKYEIVKRLQQRKHICGMTGDGVNDAPALKKADIGIAVDDATDAARSASDIVLTEPGLSVIVSAVLTSRAIFQRMKNYTIYAVSITIRIVLGFMLLALIWKFDFSPFMVLIIAILNDGTIMTISKDRVKPSPMPDSWKLKEIFGTGIVLGTYLACMTVVFFWAANDSNFFSDKFGVRSIRHNQDELTAAVYLQVSIVSQALIFVTRSRSWSFIERPGFLLVIAFILAQLVATVIAVYANWGFARIKGIGWGWAGVIWIYSVVFYFPLDVIKFLIRYAMSGKAWNNLLQNKTAFTTKKDYGKGEREAQWALAQRTLHGLTPPEMTEKSNYIELSEIAEQARKRAEVARLRELHTLKGHVESVVKLKGLDIDTIQQHYTV

>GbAHA19

MGDKNEVLEAVLKETVDLENIPIEEVFENLRCSRGGLTTEAAEERLTIFGHNKLEEKKESKFLKFLGFMWNPLSWVMEAAAIMAIALANGGGKPPDWQDFVGIITLLVINSTISFIEENNAGNAAAALMARLAPKAKVLRDGRWNEQDAAILVPGDIISIKLGDIIPADARLLEGDPLKIDQSALTGESLPVTKGPGDGIYSGSTCKQGEIEAVVIATGVHTFFGKAAHLVDTTNQVGHFQKVLTAIGNFCICSIAVGMVIEIIVMYPIQDRDYRPGIDNLLVLLIGGIPIAMPTVLSVTMAIGSHRLSQQGAITKRMTAIEEMAGMDVLCSDKTGTLTLNKLTVDKNLIEVFAKGVDADTVVLMAARASRTENQDAIDSAIVGMLADPKEARAGIREVHFLPFNPTDKRTALTYIDSDGKMHRVSKGAPEQILHLAHNKTDIERRVHAVIDKFAERGLRSLAVAYQEVPDGRKESPGGPWQFIGLMPLFDPPRHDSAETIRRALNLGVNVKMITGDQLAIGKETGRRLGMGTNMYPSSALLGQDKDESIAALPVDELIEKADGFAGVFPEHKYEIVKRLQARKHICGMTGDGVNDAPALKKADIGIAVADATDAARSASDIVLTEPGLSVIISAVLTSRAIFQRMKNYTIYAVSITIRIVLGFMLLALIWKFDFPPFMVLIIAILNDGTIMTISKDRVKPSPLPDSWKLAEIFTTGIVLGSYLAVMTVIFFWAAYKTNFFPRVFGVATLEKTAHDDIKKLASAVYLQVSIISQALIFVTRSRSWSFVERPGLLLLAAFVIAQLIATLIAVYANWSFAAIEGIGWGWAGVIWLYNIIFYIPLDFIKFFIRYALSGRAWDLVIEQRIAFTRQKDFGKEQRELQWAHAQRTLHGLQAPDTKMFTERTHFTELNQMAEEAKRRAEIARLRELHTLKGHVESVVRLKNLDIDTIQQAYTV

>GbECA4

MEKKPFPAWSWSVEQCLKEYNVKLDKGLSSYQVEKQREKYGWNELSKEKGKPLLRLVLEQFDDMLVKILLVAAYISFILAYMHGSESEESGFEAYVEPFVIVLILVLNAIVGVWQETNAEKALDALKEMQCESGKVLRDGYIVPDLPARELVPGDIVELQVGDKVPADMRIAALKTSTLRLEQSALTGEAMPVLKGTSPIFPEECELQAKENMVFAGTTVVNGSCVCIVVCTGMNTEIGKIQKQIHEASLEESDTPLKKKLDEFGSRLTTAIGLVCLIVWLINCKNFLSWDMVDGWPANLRFSFEKCTYYFKIAVALAVAAIPEGLPAVITTSLALGTRKMAQKNAIVRKLPSVETLGCTTVICSDKTGTLTTNQMSVAEFFTLGGKTTTSRIFHVEGTTYDPKDGGIVDWTCYNMDANLQVMAEICAVCNDAGIFCDGRLFRATGLPTEAALKVLVEKMGVPDAKMRNEIHDSQLAANYLIDRSTIKLGCWEWRTKRSKRLATLELDTLRKSMSVIVREPTGHNRLLVKGAVESLVERSTHVQLADGSLVPMDESCSQLLLSRNSEMSSKGLRCLGLAYKDDLGEFSDYYSENNPAHKKLLDPASYSSIESDLVFVGVVGLRDPPRDEVDKAIEDCKGAGIRVIVITGDNKSTAEAICHEIKLFSDGEDVRGKSFTGKEFMALSPSQQIETLSKPGGKVFSRAEPRHKQEIVRMLKEMGEIVAMTGDGVNDAPALKLADIGIAMGITGTEVAKEASDMVLADDNFSTIVSAVAEGRSIYNNMKAFIRYMISSNVGEVISIFLTAALGLPECMIPVQLLWVNLVTDGPPATALGFNPPDVGIMRKPPRKSDDTLIDSWVLFRYLTIGSYVGVATVGIFILWYTQASFMGINLVSDGHTLIELSQLRNWGECSTWSNFSVAPYTVGDGHLITFSNPCDYFTIGKVKAMTLSLSVLVAIEMFNSLNALSEDSSLLTLPPWRNPWLLVAMSVSFGLHCLVLYVPFLANMFGVVPLSLNEWFLVILVSIPVILIDETLKFFGRSRRHRVKEKTA

>GbAHA4

MAGISLEEIKNETVDLEKIPIEEVFEQLKCTREGLSSDEGVNRIQIFGPNKLEEKKESKILKFLGFMWNPLSWVMEAAAIMAIALANGEGKPPDWQDFVGIVCLLVINSTISFIEENNAGNAAAALMAGLAPKTKVLRDGKWTEQEAAILVPGDIISIKLGDIIPADARLLEGDPLKVDQSALTGESLPVTKNPGDEVFSGSTCKQGEIEAVVIATGVHTFFGKAAHLVDSTNQVGHFQKVLTAIGNFCICSIAIGMLVEIVVMYPIQHRKYRDGIDNLLVLLIGGIPIAMPTVLSVTMAIGSHRLSQQGAITKRMTAIEEMAGMDVLCSDKTGTLTLNKLSVDKNLIEVFVKDADKEHVVLLAARASRTENQDAIDAAIVGMLADPKEARAGIREVHFLPFNPVDKRTALTYIDSNGNWHRASKGAPEQILALCNAKEDLKKRVHSIIDKFADRGLRSLAVARQQVPEKTKESAGTPWQFVGLLPLFDPPRHDSAETIRQALHLGVNVKMITGDQLAIAKETGRRLGMGTNMYPSASLLGQDKDASIAALPVEELIEKADGFAGVFPEHKYEIVKKLQERKHICGMTGDGVNDAPALKKADIGIAVADATDAARSASDIVLTEPGLSVIISAVLTSRAIFQRMKNYTIYAVSITIRIVFGFLFIALIWKFDFSPFMVLIIAILNDGTIMTISKDRVKPSPLPDSWKLKEIFATGIVLGGYLALMTVIFFWAMHDTDFFSDKFGVRSLREREHEMMGALYLQVSIVSQALIFVTRSRSWSYAERPGLLLVTAFIIAQLVATLIAVYANWGFARIKGIGWGWAGVIWLYSIVFYVPLDIMKFAIRYILSGKAWLNLLENKTAFTTKKDYGKEEREAQWALAQRTLHGLQPPETSNLFNDKNSYRELSEIAEQAKRRAEVARLRELHTLKGHVESVVKLKGLDIDTIQQHYTV

>GbECA5

MEKKPFPAWSWSVEQCLKEYNVKLDKGLSSYQVEKQREKYGWNELSKEKGKPLLRLVLEQFDDMLVKILLVAAYISFILAYMHGSESEESGFEAYVEPFVIVLILVLNAIVGVWQETNAEKALEALKEMQCESGKVLRDGYIVPDLPARELVPGDIVELQVGDKVPADMRIAALKTSTLRLEQSALTGEAMPVLKGTSPIFPEECELQAKENMVFAGTTVVNGSCVCIVVCTGMNTEIGKIQKQIHEASLEESDTPLKKKLDEFGSRLTTAIGLVCLIVWLINCKNFLSWDMVDGWPANLRFSFEKCTYYFKIAVALAVAAIPEGLPAVITTSLALGTKKMAQKNAIVRKLPSVETLGCTTVICSDKTGTLTTNQMSVAEFFTLGGKTTTSRIFHVEGTTYDPKDGGIVDWTCYNMDANLQVMAEICAVCNDAGIFCDGRLFRATGLPTEAALKVLVEKMGVPDAKMRNEIHDSQLAANYLIDRSTIKLGCWEWRTKRSKRLATLELDTLRKSMSVIVREPTGHNRLLVKGAVESLVERSTHVQLADGSLVPMDESCSQLLLSRNSEMSSKGLRCLGLAYKDDLGEFSDYYSENHPAHKKLLDPASYSSIESDLVFVGVVGLRDPPRDEVDKAIEDCKGAGIRVIVITGDNKSTAEAICHEIKLFSDGEDVRGKSFTGKEFMALSPSQQIETLSKPGGKVFSRAEPRHKQEIVRMLKEMGEIVAMTGDGVNDAPALKLADIGIAMGITGTEVAKEASDMVLADDNFSTIVSAVAEGRSIYNNMKAFIRYMISSNVGEVISIFLTAALGLPECMIPVQLLWVNLVTDGPPATALGFNPPDVGIMWKPPRKSDDALIDSWVLFRYLTIGSYVGVATVGIFILWYTQASFMGINLVSDGHTLIELSQLRNWGECSTWSNFCVAPYMVGDGHLITFSNPCDYFTIGKVKAMTLSLSVLVAIEMFNSLNALSEDSSLLTLPPWRNTWLLVAMSVSFGLHCLILYVPFLANMFGVVPLSLNEWFLVILVSIPVVLIDETLKFFGRSRRHRVKEKTA

>GbAHA5

MWNPLSWVMEAAAIMAIALANGEGKPPDWQDFVGIVCLLVINSTISFIEENNAGNAAAALMAGLAPKTKVLRDGKWTEQEAAILVPGDIISIKLGDIIPADARLLEGDPLKVDQSALTGESLPVTKNPGDEVFSGSTCKQGEIEAVVIATGVHTFFGKAAHLVDSTNQVGHFQKVLTAIGNFCICSIAIGMLVEIVVMYPIQHRKYRDGIDNLLVLLIGGIPIAMPTVLSVTMAIGSHRLSQQGAITKRMTAIEEMAGMDVLCSDKTGTLTLNKLSVDKNLIEVFVKDADKEHVVLLAARASRTENQDAIDAAIVGMLADPKEARAGIREVHFLPFNPVDKRTALTYIDSNGNWHRASKGAPEQILALCNAKEDLKKRVHSIIDKFADRGLRSLAVARQQVPEKTKESAGTPWQFVGLLPLFDPPRHDSAETIRQALHLGVNVKMITGDQLAIAKETGRRLGMGTNMYPSASLLGQDKDASIAALPVEELIEKADGFAGVFPEHKYEIVKKLQERKHICGMTGDGVNDAPALKKADIGIAVADATDAARSASDIVLTEPGLSVIISAVLTSRAIFQRMKNYTIYAVSITIRIVFGFLFIALIWKFDFSPFMVLIIAILNDGTIMTISKDRVKPSPLPDSWKLKEIFATGIVLGGYLALMTVIFFWAMHDTDFFSDKFGVRSLREREHEMMGALYLQVSIVSQALIFVTRSRSWSYAERPGLLLVTAFIIAQLVATLIAVYANWGFARIKGIGWGWAGVIWLYSIVFYVPLDIMKFAIRYILSGKAWLNLLENKTAFTTKKDYGKEEREAQWALAQRTLHGLQPPETSNLFNDKNSYRELSEIAEQAKRRAEVARLRELHTLKGHVESVVKLKGLDIDTIQQHYTV

>GbAHA11

MADKSISLEEIKNETVDLERIPVEEVFQQLKCTRNGLTSEEGQKRLQIFGPNKLEEKKENKLLKFLGFMWNPLSWVMEFAAIMAIALANGGGKPPDWQDFIGIVSLLFINSTISFIEENNAGNAAAALMAGLAPKTKVLRDGKWNEQEAAILVPGDIISIKLGDIVPADARLLEGDALKIDQSALTGESLPVNKHSGDEVFSGSTVKQGEIEAVVIATGVHTFFGKAAHLVDSTNNVGHFQQVLTAIGNFCICSIGVGMLIEIVVMYPIQRRKYRDGIDNLLVLLIGGIPIAMPTVLSVTMAIGSHRLSQQGAITKRMTAIEEMAGMDVLCSDKTGTLTLNKLTVDKSMIEVFMDNIDKEMVLLLAARASRVENQDAIDACIVGMLGDPKEARAGVTEVHFFPFNPVDKRTAMTYIEADGSWHRASKGAPEQIIELCNLRNDAKRRAHDIITKFADRGLRSLAVAKQKVPEKTKDGQGDPWQFVGLLPLFDPPRHDSAETIRRALSLGVNVKMITGDQLAIGKETGRRLGMGTNMYPSSVLLGENKGDALDTIGVDELIEKADGFAGVFPEHKYEIVKRLQQRKHICGMTGDGVNDAPALKKADIGIAVDDATDAARSASDIVLTEPGLSVIVSAVLTSRAIFQRMKNYTIYAVSITIRIVLGFMLLALIWKFDFSPFMVLIIAILNDGTIMTISKDRVKPSPMPDSWKLKEIFGTGIVLGTYLACMTVVFFWAANDSNFFSDKFGVRSIRHNQDELTAAVYLQVSIVSQALIFVTRSRSWSFIERPGFLLVIAFILAQLVATVIAVYANWGFARIKGIGWGWAGVIWIYSVVFYFPLDVIKFLIRYAMSGKAWNNLLQNKTAFTTKKDYGKGEREAQWALAQRTLHGLTPPEMTEKSNYIELSEIAEQARKRAEVARLRELHTLKGHVESVVKLKGLDIDTIQQHYTV

>GbAHA1

MGGEKGISLEEIKNESVDLERIPIEEVFEQLKCTREGLSTEEGNNRLQVFGPNKLEEKKESKVLKFLGFMWNPLSWVMEAAAIMAIALANGDGRPPDWQDFVGIIVLLVINSTISFIEENNAGNAAAALMANLAPKTKVLRDGRWSEQEAAILVPGDIITIKLGDIVPADARLLEGDPLKIDQSALTGESLPVTKNPSDEVFSGSTCKQGEIEAVVIATGVHTFFGKAAHLVDSTNQVGHFQKVLTAIGNFCICSIAVGIIVELIVMYPIQHRKYRDGIDNMLVLLIGGIPIAMPTVLSVTMAIGSHRLSQQGAITKRMTAIEEMAGMDVLCSDKTGTLTLNKLTVDRNLIEVFTKGVEKEHVILYAARASRTENQDAIDAAIVGMLADPKEARAGVREIHFLPFNPVDKRTALTYIDNDGNWHRASKGAPEQIIDLCKCKDDVRKKVHSVIDKFAERGLRSLAVARQEIPEKTKESPGSPWQFIGLLPLFDPPRHDSAETIRRALNLGVNVKMITGDQLAIAKETGRRLGMGTNMYPSSSLLGQDKDASIAALPIDELIEKADGFAGVFPEHKYEIVKRLQERKHICGMTGDGVNDAPALKKADIGIAVADATDAARSASDIVLTEPGLSVIISAVLTSRAIFQRMKNYTIYAVSITIRIVFGFMFIALIWKFDFAPFMVLIIAILNDGTIMTISKDRVKPSPQPDSWKLKEIFSTGIVLGGYLALMTVIFFWAMKDTNFFSNTFNVRSLRHSIDGEREMMAALYLQVSIVSQALIFVTRSRSWSYFERPGLLLVSAFLVAQLVATLIAVYAEWGFARIKGMGWGWAGVIWLYSVVTFIPLDFIKFAIRYVLSGKAWDNLLENKTAFTTKKDYGKEEREAQWAAAQRTLHGLQPPETTSIFNERSSYRELSEIAEQAKRRAEVARLRELNTLKGHVESVVKLKGLDIDNIQQHYTV

>GbAHA2

ERIPIEEVFEQLKCTREGLSTEEGNNRLQVFGPNKLEEKKESKVLKFLGFMWNPLSWVMEAAAIMAIALANGDGRPPDWQDFVGIIVLLVINSTISFIEENNAGNAAAALMANLAPKTKVLRDGRWSEQEAAILVPGDIITIKLGDIVPADARLLEGDPLKIDQSALTGESLPVTKNPSDEVFSGSTCKQGEIEAVVIATGVHTFFGKAAHLVDSTNQVGHFQKVLTAIGNFCICSIAVGIIVELIVMYPIQHRKYRDGIDNMLVLLIGGIPIAMPTVLSVTMAIGSHRLSQQGAITKRMTAIEEMAGMDVLCSDKTGTLTLNKLTVDRNLIEVFTKGVEKEHVILYAARASRTENQDAIDAAIVGMLADPKEARAGVREIHFLPFNPVDKRTALTYIDNDGNWHRASKGAPEQIIDLCKCKDDVRKKVHSVIDKFAERGLRSLAVARQEIPEKTKESPGSPWQFIGLLPLFDPPRHDSAETIRRALNLGVNVKMITGDQLAIAKETGRRLGMGTNMYPSSSLLGQDKDASIAALPIDELIEKADGFAGVFPEHKYEIVKRLQERKHICGMTGDGVNDAPALKKADIGIAVADATDAARSASDIVLTEPGLSVIISAVLTSRAIFQRMKNYTIYAVSITIRIVFGFMFIALIWKFDFAPFMVLIIAILNDGTIMTISKDRVKPSPQPDSWKLKEIFSTGIVLGGYLALMTVIFFWAMKDTNFFSNTFNVRSLRHSIDGEREMMAALYLQVSIVSQALIFVTRSRSWSYFERPGLLLVSAFLVAQLVATLIAVYAEWGFARIKGMGWGWAGVIWLYSVVTFIPLDFIKFAIRYVLSGKAWDNLLENKTAFTTKKDYGKEEREAQWAAAQRTLHGLQPPETTSIFNERSSYRELSEIAEQAKRRAEVARLRELNTLKGHVESVVKLKGLDIDNIQQHYTV

>GbAHA3

ESKVLKFLGFMWNPLSWVMEAAAIMAIALANGDGRPPDWQDFVGIIVLLVINSTISFIEENNAGNAAAALMANLAPKTKVLRDGRWSEQEAAILVPGDIITIKLGDIVPADARLLEGDPLKIDQSALTGESLPVTKNPSDEVFSGSTCKQGEIEAVVIATGVHTFFGKAAHLVDSTNQVGHFQKVLTAIGNFCICSIAVGIIVELIVMYPIQHRKYRDGIDNMLVLLIGGIPIAMPTVLSVTMAIGSHRLSQQGAITKRMTAIEEMAGMDVLCSDKTGTLTLNKLTVDRNLIEVFTKGVEKEHVILYAARASRTENQDAIDAAIVGMLADPKEARAGVREIHFLPFNPVDKRTALTYIDSDGNWHRASKGAPEQIIDLCKCKDDVRKKVHSVIDKFAERGLRSLAVARQEIPEKTKESPGSPWQFIGLLPLFDPPRHDSAETIRRALNLGVNVKMITGDQLAIAKETGRRLGMGTNMYPSSSLLGQDKDASIAALPTDELIEKADGFAGVFPEHKYEIVKRLQERKHICGMTGDGVNDAPALKKADIGIAVADATDAARSASDIVLTEPGLSVIISAVLTSRAIFQRMKNYTIYAVSITIRIVFGFMFIALIWKFDFAPFMVLIIAILNDGTIMTISKDRVKPSPQPDSWKLKEIFSTGIVLGGYLALMTVIFFWAMKDTNFFSNTFNVRSLRHNNIDGEKEMMAALYLQVSIVSQALIFVTRSRSWSYFERPGLLLVSAFLVAQLVATLIAVYADWGFARIKGMGWGWAGVIWLYSVVTYIPLDLIKFAIRYVLSGKAWDNLLENKTAFTTKKDYGKEEREAQWAAAQRTLHGLQPPETTSIFNERSSYRELSEIAEQAKRRAEVARLRELNTLKGHVESVVKLKGLDIDNIQQHYTV

>GbECA6

MEERAFPAWSWSVEHCLKEYDVRLDKGLSSYKVEKQREKYGWNELAKEKGKPLWRLVLEQFDDMLVKILLVAAFISFLLAYMHGSESEESGFEAYVEPFVIVLILVLNAIVGVWQETNAEKALEALKEMQCESGKVLRDGFLVPDLPARELVPGDIVELQVGDKVPADMRIAALKTSTLRLEQSALTGEAMPVLKGTSPIFQKECELQAKENIVFAGTTVVNGCCVCIVVCTGMNTEIGKIQRQIHEASLEDSDTPLKKKLDEFGSRLTTAIGIVCLIVWLINYKNFLSYDMVDGWPANFRFSFEKCTYYFKIAVALAVAAIPEGLPAVITTSLALGTRKMAQKNAIVRKLPSVETLGCTTVICSDKTGTLTTNQMSVAEFFTLGGKTTTSRIFHVKGTTYDPKDGGIVDWTCYNMDANLQVMAEICAVCNDAGIFCDGRLFRATGLPTEAALKVLAEKMGVPDAKMRSKIRDSELVANYLIDRSTVKLGCCEWWIKRSKRVATLEFDRVRKSSSIIVREAAGQNRLLAKGAVESLLERSTHVQLADGSLAPMDEPCRQLLLSRQTEMSSKGLRCLGLAYKEDLGEFSDYYSENHPAHKKLLDPACYCSIENDLVFVGVVGLRDPPRDEVHKAIEDCKRAGIRVMVITGDNKSTAEAICREIKLFSDGEDLRGKSFTGKEFMALSPSQQIETLSKPGGKVFSRAEPRHKQEIVRMLKEMGEIVAMTGDGVNDAPALKLADIGIAMGITGTEVAKEASDMVLADDNFSTIVSAVAEECMIPVQLLWVNLVTDGPPATALGFNPPDIGIMRKPPRRSDDALINSWVLFRYLIIGSYVGIATVGIFILWYTRASFMGINLVSDGHTLVELSQLRNWGECSTWSNFTVAPYMVGGGQLISFSNPCDYFTAGKVKAMTLSLSVLVAIEMFNSLNALSEDSSLLMLPPWRNPWLLAAMSVSFGLHCLILYVPFLADIFAVAPLSLNEWFLVILVSVPVILIDEILKFVGRGQRYRVKEKTA

>GbAHA6

MVDIDDTLQEIKNENVDLERIPVEEVFLQLKCTKEGLTTEEGLKRLQIFGPNKLEEKSESKVLKFLGFMWNPLSWVMEIAAIMAIALANGGGKPPDWQDFVGITVLLIINSTISFIEENNAGNAAAALMAGLAPKTKVLRDGKWCEQEAAILVPGDIISIKLGDIIPADARLLEGDALKVDQSALTGESLPVNKNPGDGVYSGSTVKQGELEAVVIATGVHTFFGKAAHLVDSTNNVGHFQKVLTAIGNFCICSIAVGMLVEIVVMYPIQRRKYRDGIDNLLVLLIGGIPIAMPTVLSVTMAIGSHRLSQQGAITKRMTAIEEMAGMDVLCSDKTGTLTLNKLTVDKSMVEVFTKDVDKEMLLLLAARASRVENQDAIDACIVGMLGDPKEAREGITEVHFFPFNPVDKRTAMTYIESDGSWHRVSKGAPEQIISLCDLRDDVKKKAHDIIDKFADRGLRSLGVARQTVPEKTKDSPGSPWEFVGLLPLFDPPRHDSAETIRRALHLGVNVKMITGDQLAIGKETGRRLGMGTNMYPSSALLGHNKDEKVETIDVDELIEKADGFAGVFPEHKYEIVKRLQERNHICGMTGDGVNDAPALKKADIGIAVDDATDAARSASDIVLTEPGLSVIVSAVLTSRAIFQRMKNYTIYAVSITIRIVLGFMLLALIWKFDFSPFMVLIIAILNDGTIMTISKDRVKPSPMPDSWKLNEIFATGIVLGTYLACMTVVFFWAANDSDFFSDKFGVKSIRYSQDELTAAVYLQVSIVSQALIFVTRSRSWSFIERPGLLLVVAFILAQLVATVLAVYANWGFARIKGIGWPWAGVIWLYSIVFYIPLDVLKFLIRYALSGKAWDNLLQNKTAFTTKKDYGRGEREAQWATAQRTLHGLQAPGAEEILNEKSSYRELSEIAEQAKKRAEVARLRELHTLKGHVDSVVKLKGLDIETINQNYTV

>GbAHA7

MVDIDDTLQEIKNENVDLEIKNENVDLERIPVEEVFLQLKCTKEGLTTEEGLKRLQIFGPNKLEEKSESKVLKFLGFMWNPLSWVMEIAAIMAIALANGGGKPPDWQDFVGITVLLIINSTISFIEENNAGNAAAALMAGLAPKTKVLRDGKWCEQEAAILVPGDIISIKLGDIIPADARLLEGDALKVDQSALTGESLPVNKNPGDGVYSGSTVKQGELEAVVIATGVHTFFGKAAHLVDSTNNVGHFQKVLTAIGNFCICSIAVGMLVEIVVMYPIQRRKYRDGIDNLLVLLIGGIPIAMPTVLSVTMAIGSHRLSQQGAITKRMTAIEEMAGMDVLCSDKTGTLTLNKLTVDKSMVEVFTKDVDKEMLLLLAARASRVENQDAIDACIVGMLGDPKEAREGITEVHFFPFNPVDKRTAMTYIESDGSWHRVSKGAPEQIISLCDLRDDVKKKAHDIIDKFADRGLRSLGVARQTVPEKTKDSPGSPWEFVGLLPLFDPPRHDSAETIRRALHLGVNVKMITGDQLAIGKETGRRLGMGTNMYPSSALLGHNKDEKVDTIDVDELIEKADGFAGVFPEHKYEIVKRLQERNHICGMTGDGVNDAPALKKADIGIAVDDATDAARSASDIVLTEPGLSVIVSAVLTSRAIFQRMKNYTIYAVSITIRIVLGFMLLALIWKFDFSPFMVLIIAILNDGTIMTISKDRVKPSPMPDSWKLKEIFATGIVLGTYLACMTVVFFWAANDSDFFSDKFGVKSIRYSQDELTAAVYLQVSIVSQALIFVTRSRSWSFIERPGLLLVVAFILAQLVATVLAVYANWGFARIKGIGWPWAGVIWLYSIVFYIPLDVLKFLIRYALSGKAWDNLLQNKTAFTTKKDYGRGEREAQWATAQRTLHGLQAPGAEEILNEKSSYRELSEIAEQAKKRAEVARLRELHTLKGHVDSVVKLKGLDIETINQNYTV

>GbAHA16

MENKDETMDAVLKEAVDLENVPLEEVFQTLRCNRDGLTTEAAEQRLSIFGYNKLEEKQESKILKFLGFMWNPLSWVMEAAAIMAIALANGGGKPPDWQDFVGIITLLIINSTISFIEENNAGNAAAALMARLAPKAKVFRDGKWSEEEASILVPGDIISIKLGDIIPADARLLDGDPLKIDQSSLTGESLPVTKGPGDSIYSGSTCKQGEIEAVVIATGVHTFFGKAAHLVDSTNQQGHFQKVLTAIGNFCICSIAVGMITEIIVMYPIQDRDYRPGIDNLLVLLIGGIPIAMPTVLSVTMAIGSHRLSLQGAITKRMTAIEEMAGMDVLCSDKTGTLTLNKLSVDKNLIEIFAKGVDPDTVVLMAARASRLENQDAIDAAIVGMLADPKEARAGIQEVHFLPFNPTDKRTALTYIDNQGRMHRVSKGAPEQILNLAHNKSELERRVHAVIDKFAERGLRSLAVAYQEVPEGRKESSGGPWQFIGLMPLFDPPRHDSADTIRRALNLGVNVKMITGDQLAIAKETGRRLGMGTNMYPSSALLGQNKEESIAALPVDELIEKADGFAGVFPEHKYEIVKRLQARKHICGMTGDGVNDAPALKKADIGIAVADATDAARSASDIVLTEPGLSVIISAVLTSRAIFQRMKNYTIYAVSITIRIVLGFMLLALIWKFDFPPFMVLIIAILNDGTIMTISKDRVKPSPLPDSWKLAEIFATGIILGGYLAMMTVIFFWAAYKTDFFPSTFGVKSLQKTDRKDIKMLASAVYLQVSIISQALIFVTRARSWSFLERPGFLLVLAFVVAQLIATLIAVYANWGFAAIEGIGWGWAGVIWLYNLIFYIPLDFIKFFIRYALSGKAWDLVIEQRIAFTRKKDFGKEERELKWAHAQRTLHGLQPPDTKMFGDRTSYNELNQMAEEAKRRAEIARLRELTTLKGHVESVVRLKGLDIDTIQQAYTV

>GbECA7

MEERAFPAWSWSVEHCLKEYDVRLDKGLSSYKVEKQREKYGWNELAKEKGKPLWRLVLEQFDDMLVKILLVAAFISFLLAYMHGSESEESGFEAYVEPFVIVLILVLNAIVGVWQETNAEKALEALKEMQCESGKVLRDGFLVPDLPARELVPGDIVELQVGDKVPADMRIAALKTSTLRLEQSALTGEAMPVLKGTSPIFQKECELQAKENMVFAGTTVVNGCCVCIVVCTGMNTEIGKIQKQIHEASLEDSDTPLKKKLDEFGSRLTTAIGIVCLIVWLINYKNFLSYDMVDGWPANFRFSFEKCTYYFKIAVALAVAAIPEGLPAVITTSLALGTRKMAQKNAIVRKLPIVETLGCTTVICSDKTGTLTTNQMSVAEFFTLGTTYDPKDGGIVDWTCYNMDANLQVMAEICAVCNDAGIFCDGRLFRATGLPTEAALKVLAEKMGVPDAKMRNKIRDSELVANYLIDRSTVKLGCCEWWIKRSKRVATLEFDRVRKSSSIIVREAAGQSRLLAKGAVESLLERSTHVQLADGSLAPMDEPCRQLLLSRQTEMSSKGLRCLGLAYKEDLGEFSDYYSENHPAHKKLLDPACYCSIESDLVFVGAVGLRDPPRDEVHKAIEDCKRAGIRVMVITGDNKSTAEAICREIKLFSDGEDLRGKSFTGKEFMALSPSQQIETLSKPGGKVFSRAEPRHKQEIVRMLKEMGEIVAMTGDGVNDAPALKLADIGIAMGITGTEVAKEASDMVLADDNFSTIVSAVAEECMIPVQLLWVNLVTDGPPATALGFNPPDIGIMRKPPRRSDDALINSWVLFRYLIIGSYVGIATVGIFILWYTRASFMGINLVSDGHTLVELSQLRNWGECSTWSNFTVAPYMVGGGQLISFSNPCDYFTAGKVKAMTLSLSVLVAIEMFNSLNALSEDSSLLMLPPWRNPWLLVAMSVSFGLHCLILYVPFLADIFAVAPLSLNEWFLVILVSVPVILIDEILKFVGRGQRYRVKEKTA

>GbAHA17

MENKDETLDAVLKEAVDLENVPLEEVFQTLRCNRDGLTTEAAEQRLSIFGYNKLEEKQESKILKFLGFMWNPLSWVMEAAAIMAIALANGGGKPPDWQDFVGIITLLIINSTISFIEENNAGNAAAALMARLAPKAKVFRDGKWSEEEASILVPGDVISIKLGDIIPADARLLDGDPLKIDQSSLTGESLPVTKGPGDSIYSGSTCKQGEIEAVVIATGVHTFFGKAAHLVDSTNQQGHFQKVLTAIGNFCICSIAVGMITEIIVMYPIQDREYRPGIDNLLVLLIGGIPIAMPTVLSVTMAIGSHRLSLQGAITKRMTAIEEMAGMDVLCSDKTGTLTLNKLSVDKNLIEIFAKGVDPDTVVLMAARAARLENQDAIDAAIVGMLADPKEARAGIQEVHFLPFNPTDKRTALTYIDNQGRMHRVSKGAPEQILNLAHNKSELERRVHAVIDKFAERGLRSLAVAYQEVPDGRKESSGGPWQFIGLMPLFDPPRHDSADTIRRALNLGVNVKMITGDQLAIAKETGRRLGMGTNMYPSSSLLGQNKEESIAALPVDELIEKADGFAGVFPEHKYEIVKRLQARKHICGMTGDGVNDAPALKKADIGIAVADATDAARSASDIVLTEPGLSVIISAVLTSRAIFQRMKNYTIYAVSITIRIVLGFMLLALIWKFDFPPFMVLIIAILNDGTIMTISKDRVKPSPLPDSWKLAEIFATGIILGGYLAMMTVIFFWAAYKTDFFPSTFGVKSLQKTDRKDIKMLASAVYLQVSIISQALIFVTRARSWSFLERPGFLLVLAFVVAQLIATLIAVYANWGFAAIEGIGWGWAGVIWLYNLIFYIPLDFIKFFIRYALSGKAWDLVIEQRIAFTRKKDFGKEERELKWAHAQRTLHGLQPPDTKMFGDRTSYNELNQMAEEAKRRAEIARLRELTTLKGHVESVVRLKGLDIDTIQQAYTV

>GbECA10

MGKGGENYGKREFVNSKPTDPDVFPAWAKDIRECEKHYDVSRKSGLSTAEVENRRRIYGNNELEKHEGQSIWSLIMEQFNDTLVRILLLAAVVSFVLAWYDGDEGGEMEITAFVEPLVIFLILIVNAIVGVWQENNAEKALEALKEIQSEQATVIRDGIKIPSLPAKELVPGDIVELKVGDKVPADMRVLELVSSTLRVEQGSLTGESEAVNKTNKSVSEDADIQGKRSMVFAGTTVVNGNCFCLVTQIGMETEIGKVHTQIHVASQSEEDTPLKKKLNEFGEVLTMIIGVICLFVWLINVKYFLSWEYIDGWPRNFKFSFEKCTYYFEIAVALAVAAIPEGLPAVITTCLALGTRKMAQKNALVRKLPSVETLGCTTVICSDKTGTLTTNQMAVAKLVAMGGHASSLRSFRVDGTTYNPSDGKIHGWPSGGMDANLETIAKISAICNDAGVTHSDNKYVAHGMPTEAAIKVLVEKMGLPKGLYSGGAAGNDVLRCCQWWNEYEHRIATLEFDRDRKSMGVIVKSKSGRRSLLVKGAVENLLERSSKMQLLDGSVVPLDQNSRILVSNALQDMSSGALRCLGFAYKDELPEFETYDGSDDHPAHALLLDPSNYPSIESNLTFVGLVGLRDPPRQEVHQAIEDCKAAGIRVMVITGDNKNTAEAICREIGVFGPMEDISSKSLTGKEFMALSDKKAHLRQSGGLLFSRAEPRHKQEIVRLLKEDGEVVAMTGDGVNDAPALKLADIGIAMGIAGTEVAKEASDMVLADDNFSTIVSAVGEGRSIYDNMKAFIRYMISSNIGEVASIFLTAALGIPEGLIPVQLLWVNLVTDGPPATALGFNPPDKDIMKKPPRRSDDSLITAWILFRYLVIGLYVGVATVGIFIIWYTHGSFLGIDLSGDGHTLVTYPQLANWAQCSSWKNFTVSPFTAGNQVFSFENNPCDYFQGGKVKAMTLSLSVLVAIEMFNSLNALSEDGSLLTMPPWVNPWLLLAMSVSFGLHFLILYVPFLAQVFGIVPLSFNEWLLVLAVAFPVILIDEVLKFVGRLGRRMRSSSQRPLKPKTE

>GbECA12

MGKGGENYGKREFVNSKPTDPDVFPAWAKDIRECEKHYDVSRKSGLSTAEVENRRRIYGNNELEKHEGQSIWSLIMEQFNDTLVRILLLAAVVSFVLAWYDGDEGGEMEITAFVEPLVIFLILIVNAIVGVWQENNAEKALEALKEIQSEQATVIRDGIKIPSLPAKELVPGDIVELKVGDKVPADMRVLELVSSTLRVEQGSLTGESEAVNKTNKSVSEDADIQGKRSMVFAGTTVVNGNCFCLVTQIGMETEIGKVHTQIHVASQSEEDTPLKKKLNEFGEVLTMIIGVICLFVWLINVKYFLSWEYIDGWPRNFKFSFEKCTYYFEIAVALAVAAIPEGLPAVITTCLALGTRKMAQKNALVRKLPSVETLGCTTVICSDKTGTLTTNQMAVAKLVAMGGHASSLRSFRVDGTTYNPSDGKIHDWPSGGMDANLETIAKISAICNDAGVTHSDNKYVAHGMPTEAAIKVLVEKMGLPKGSYSGGAAGDDVLRCCQWWNEYEHRIATLEFDRDRKSMGVIVKSKSGRRSLLVKGAVENLLERSSKMQLLDGSVVPLDQNSRILVSNALQDMSSGALRCLGFAYKDELPEFETYDGSDDHPAHALLLDPSNYSSIESNLTFVGLVGLRDPPRQEVHQAIEDCKAAGIRVMVITGDNKNTAEAICHEIGVFGPMEDISSKSLTGKEFMALSDKKAHLRQSGGLLFSRAEPRHKQEIVRLLKEDGEVVAMTGDGVNDAPALKLADIGIAMGIAGTEVAKEASDMVLADDNFSTIVSAVGEGRSIYDNMKAFIRYMISSNIGEVASIFLTAALGIPEGLIPVQLLWVNLVTDGPPATALGFNPPDKDIMKKPPRRSDDSLITAWILFRYLVAGLNLCLNLRQLLAWWNEYEHRIATLEFDRDRKSMGVIVKSKSGRRSLLVKGAVENLLERSSKMQLLDGSVVPLDQNSRILVSNALQDMSSGALRCLGFAYKDELPEFETYDGSDDHPAHALLLDPSNYPSIESNLTFVGLVGLRDPPRQEVHQAIEDCKAAGIRVMVITGDNKNTAEAICHEIGVFGPMEDISSKSLTGKEFMALSDKKAHLRQSGGLLFSRAEPRHKQEIVRLLKEDGEVVAMTGDGVNDAPALKLADIGIAMGIAGTEVAKEASDMVLADDNFSTIVSAVGEGRSIYDNMKAFIRYMISSNIGEVASIFLTAALGIPEGLIPVQLLWVNLVTDGPPATALGFNPPDKDIMKKPPRRSDDSLITAWILFRYLVIGLYVGVATVGVFIIWYTHSSFLCIDLSGDGHTLVTYPQLANWAQCSSWKNFTVSPFTAGNQVFYFENNPCDYFQGGKVKAMTLSLSVLVAIEMFNSLNALSEDGSLLTMPPWVNPWLLLAMSVSFGLHFLILYVPFLAQVFGIVPLSFNEWLLVLAVAFPVILIDEVLKFVGRLGRRMRSSSQRPLKPKTE

>GbAHA12

MASDGDISLEQIKNESVDLENIPVEEVFKLLKCTKEGLTTAEGETRLSIFGHNKLEEKKDNKVLKFLGFMWNPLSWVMEAAAIVAIVLANGGGKPPDWPDFIGIVTLLLINSTISFIEENSAGNAAAALMAGLAPKTKVLRDGKWSEQEASILVPGDIISVKLGDIIPADARLLEGDPLKIDQAALTGESLPVTKNPGDLVFSGSTCKQGELDAIVIATGIHSFFGKAAHLVDNTNNVGHFQQVLTSIGNFCIFSIGIGMVIEIIVMYPIQHRNYRDGIDNLLVLLIGGIPIAMPTVLSVTMAIGSHRLSEQGAITKRMTAIEEMAGMDILCSDKTGTLTLNKLTIDKNLVEVFINDMDVDTLVLLAARASRVENQDAIDACIVGMLSDPKQAREGITELHFLPFNPVDKRTAITYTDRKGEWHRCSKGAPEEIIDLCGLTGGLRKKALSIIDGYANRGLRSLGVARQTIPEKTKESPGGPWEFVGLLPLFDPPRHDSAETIKRALELGISVKMITGDQLAIGKETGRRLGMGTDMYPSSALLGECGDEDIAAIPIDDLIEKADGFAGVFPEHKYEIVGRLQERKHICGMTGDGVNDAPALKKADIGIAVADSTDAARGASDIVLTEPGLSVIVSAVLTSRSIFQRMKNYTIYAVSITIRIVLGFMLVALIWKFDFSPFMVLVIAILNDGTIMTISKDRVRPSPKPDTWKLDEIFATGVVLGTYMAIVTVFFFWLVHDTEFFTRTFGVKPINDNEDALTTALYLQVSTVSQALIFVTRSRSWSFIELPGPLLLIAFILAQLVATLLAVYANWGFARIQGIGWEWAGAIWVFSIITYIPLDVLKFIIRYALTGDAWGDVVQSKTAFKGESEVQLTTNYRPLQGVSSPPETWSNDELVPVV

>GbECA8

MGRGGENYGKRENAGAASSKQETFPAWARDVKQCEEKYQVNRELGLPSAEVEKRRQIYGLNELLKHEPTSIFQLLLEQFTDTLVRILLAAAIISFVLAWYDGEGRGEKEITAFVEPLVIFLILIVNAIVGIWQESSAEKALEALKEIQSEHADVIRDGKKVSSLPAKELVPGDIVELRVGDKVPADMRVLSLISSTVRVEQGSLTGESEAVSKTVKVVPENSDIQGKKCMVFAGTTMVNGNCICLVTEIGMNTEIGKVHSQIHEASQSDTDTPLKKKLNEFGEVLTLIIGIICILVWLINVKYFLSWEYVDGWPTNFKFSFEKCTYYFEIAVALAVAAIPEGLPAVITTCLALGTRKMAQKNALVRKLPSVETLGCTTVICSDKTGTLTTNQMAVSKLVAIGSRSGTLRSFDVEGTTYDPFDGKILGWPVDGMDANLEMIAKISAVCNDAGVEQSGSHYVAIGMPTEAALKVLVEKMELPEKYASSSAPGDPRRCCQVWNKMVQRIATLEFDRDRKSMGVIINSSSGNKSLLVKGAVENLLERSSFIQLLDGSTVELDKYSKDLILQVLREMSTDALRCLGFAYKEELPEFATYNSDEDHPAHQLLLNPSNYSSIESNLIFVGLVGLRDPPRKEVRQAIEDCKAAGIRVMVITGDNKNTAEAICREIGVFGYREDITSRSLTGKEFMDHPDQRNHLRQNGGLLFSRAEPRHKQEIVRLLKQDGEVVAMTGDGVNDAPALKLADIGVAMGITGTEVAKEASDMVLADDNFSTIVAAVGEGRSIYDNMKAFIRYMISSNIGEVASIFLTAALGIPEGMIPVQLLWVNLVTDGPPATALGFNPQDTDIMKKPPRRSNDSLITAWILFRYLVIGSYVGLATVGVFIIWYTHNTFMGIDLSGDGHSLVTYSQLSNWDKCPSWVNFTASPFTAGPQVFNFDTNPCDYFRSGKIKASTLSLSVLVSIEMFNSLNALSEDGSLLTMPPWVNPWLLLAMSISFGLHFLILYVPFLAQVFGIVPLSHNEWLLVLAVAFPVILIDEVLKCIGRCTTGPRYSPATISIKHKAE

>GbECA9

MGRGGENYGKRENAGAASFKQETFPAWARDVKQCEEKYQVNRELGLSSAEVEKRRQIYGLNELLKHKPTSIFQLLLEQFNDTLVRILLAAAIISFVLAWYDGEEGGEKEITAFVEPLVIFLILIVNAFVGIWQESSAEKALEALKEIQSEHADVIRDGKKVSSLPAKELVPGDIVELRVGDKVPADMRVLNMISSTVRVEQGSLTGESEAVSKTVKVVPENSDIQGKKCMVFAGTTMVNGNCICLVTEIGMNTEIGKVHSQIHEASQSDTDTPLKKKLNEFGEVLTMIIGMICILVWLINVKYFLSWEYVDGWPTNFKFSFEKCTYYFEIAVALAVAAIPEGLPAVITTCLALGTRKMAQKNALVRKLPSVETLGCTTVICSDKTGTLTTNQMAVSKLVAIGSRSGTLRSFDVEGTTYDPFDGKILGWPVDGMDSNLEMIAKISAVCNDAGVEQSGRHYVAIGMPTEAALKVLVEKMELPEKYASSSAPGDPQRCCHVWNKMVQRIATLEFDRDRKSMGVIINSSSGNKSLLVKGAVENLLERSSFIQLLDGSTVELDKYSKDLILQVLREMSTDALRCLGFAYKEELPEFATYNGDEDHPAHQLLLNPSNYSSIESNLIFVGLVGLRDPPRKEVRQAIEDCKAAGIRVMVITGDNKNTAEAICREIGVFGYREDITSRSLTGKEFMDHPDQRNHLRQNGGLLFSRAEPRHKQEIVRLLKQDGEVVAMTGDGVNDAPALKLADIGVAMGITGTEVAKEASDMVLADDNFSTIVAAVGEGRSIYDNMKAFIRSLLLWFL

>GbHMA1

MVANKNLQKSYFDVLGLCCSSEVPLIENILKSLEGVKEVSVIVPTRTVIVLHDNLLLSQLQIVKALNQARLEANVRAHGEIKYQKKWPSPFAVVCGLLLLLSFLKYVYHPLQWLAVGAVVIGIYPVLFKGFAAITHFRIDINILILIAVIGSVAMKDYTEAGTIVFLFTTAEWLESRASHKASAVMSSLMRITPQKAVIAENGEEVDADEVKLNTLLAVKAGEVIPIDGIVVDGRCEVDEKSLTGESLPVTKEKDSTVWAGTINLNGYISVKTTAVAEDCVVAKMAKLVEEAQNSKSNTQRSIDKCAQFYTPVIIVVSAAIAVIPAALRVQNLHHWFHLALVVLVSACPCALILSTPVASFCALTKAATSGLLVKGGDYLETLSKIRITAFDKTGTLTRGEFIVTDFQPLSQDISLDTLLYWVSSIESKSSHPMAAALVDYGRSHSIEPNPETVEDYQNFPGEGIYGRVDGRDIYIGSLKISVRAHGTVPTLEGNMMKGKTIGFVYSGATPAGIFSLSDACRTGVPEAVEELKSMRIKVAMLTGDNQSAAIHVQEQLGNRLDVVHAELLPEDKARIIKEFKKEGATAMIGDGVNDAPALATADIGISMGISGSALATETGHVVLMSNDIRKIPKAIKLARKAHRKVIENVILSISTKTAILALAFAGHPLVWAAVLADVGTCLLVICNSMLLLRGKHKDGRKCCKSSAAAHTNKHGCKASHCDSSHKHQDASLDKKVQKACEPPTCSSERCASRCHSGLFKTDSPSNSRESDKCEDLGRTHDGSVIREAKYCDQGSCHLVNHKIEAQNLPRKCCSGRGSLDLGKEANALHGAKQCHQGHLHQYSSSTPEEEQRETKNDHCHSTHCRENHVEIHGNNLTAFGNLVEHRCLESLNQRAHLDSHEPTHTAIDITMNPDEVHGCANVEKRELGGCCKSYMKECCGKHKHGRFRPGLTDIITE

>GbHMA2

MVANKNLQKSYFDVLGLCCSSEVPLIENILKSLEGVKEVSVIVPTRTVIVLHDNLLLSQLQIVKALNQARLEANVRAHGEIKYQKKWPSPFAVVCGLLLLLSFLKYVYHPLQWLAVGAVVIGIYPVLFKGFAAITHFRIDINILILIAVIGSVAMKDYTEAGTIVFLFTIAEWLESRASHKASAVMSSLMSITPQKAVIAENGEEVDADEVKLNTLLAVKAGEAIPIDGIVVDGRCEVDEKSLTGESLPVTKEKDSTVWAGTINLNGYISVKTTAVAKDCVVAKMAKLVEEAQNSKSNTQRFIDKCAQFYTPAIIIVSAAIAVIPTALRVKNIHHWFHLALVVLVSACPCSLILSTPVASFCALTKAATSGLLVKGGDYLETLSKIRITAFDKTGTLTRGEFIVTDFQPLSQDISLDTLLYWVSSIESKSSHPMAAALVDYGRSHSVEPNPETVEDYQNFPGEGIYGRVDGRDIYIGSKKISVRAHGTVPTLEGNMMKGKTIGFVYSGATPAGIFSLSDACRTGVPEAVDELKSMRIKAAMLTGDNQAVAIHVQEQLGNRLDVVHAELLPEDKARIIKEFKKEGATAMIGDGVNDAPALATADIGISMGISGSALATETGHVVLMSNDIRKIPKAIKLAKKAHRKVIENVILSISTKTAILALAFAGHPLVWAAVLADVGTCLLVICNSMLLLRGKHKDGRKCCKSSAAAHTNKHGSKASHCDSFHKHQDASLDKKVQKACEPPTCCSERCASRCHSGLFKTDSSSNSRGSDKCEDSRRTHDGFVIREAKYCNQRSCHLVNHKIEAQNLPRKCCSGHGSLDLGKEANALYGAKQCHQGHLHQYSSSTPKKEQQGIKNDHCHSTYCGENHVDIHGNNLTAFENLVEHRCLESLNQTGHLDSHEPTHTAIDITMNPDEVHGCTSVEKRELGGCCKSYMKECCGKHGRFRPGLTEIITE

>GbHMA3

MAANKKKLQKSYFDVLGLCCSSEVPLIENILKPLEGVKQVSVIVPTRTVIVVHDNLLVSQLQIVKALNQARLEANVRAHGEIKYQKKWPSPFAMACGLLLLISFFKYVYRPLQWVAVGAVVIGICPILLKGYAAITNFRLDINILMLIAVIGSIAMKDYTEAATIVFLFTIAEWLESRASYKATAVMSSLMSMSPQKAVIAESGEEVDVDEVKLNTVLAVKAGEVIPIDGIVVDGNCEVDEKTLTGESLPVSKQKDSTVWAGTINLNGYISVKTTAVAEDCVVAKMAKLVEEAQNSKSTTQRFIDKCAQFYTPAIIVVSVAIAVIPAAFRVHNLRHWFHLALVVLVSACPCALILSTPVASFCALTKAATSGLLVKGGDYLEILSNIKITAFDKTGTLTRGEFVVTNFRSLCQDISFNSLLYWVSSIESKSSHPMAAALIEYGRSHSIEPKPETVEDYQNFPGEGIYGRIDGRDIYIGSRKVSVRAHGTAPNVEGNMMEGKTIGYVFCGATPAGIFSLSDACRTGAAEAVNELKSMGIKTAMLTGDNQAAAIHVQEQLRNSLDVIHADLLPQDKARIVEEFKKEGPTAMLGDGINDAPALATADIGISMGISGSALATETGHVILMSNDIRKIPKAIRLARKAHRKVIQNVILSISTKVAILALAFAGHPLVWAAVLADVGTCLLVIFNSMLLLHGTHKHAGKCSKSSAASHKDKQGCNTSHCHSSHNHEHSSIDKKVQKACEPQKCSSRSCASRCQSNPSNSDASSNSCGSNKCTESTGTREMKHCDQGSCNIVNHKIEAHNLPSKCCSSHGKLGEKHCHHSSNQQGTKADHCHSTCGGNHTDRQTLGTFVEHSCLESPKPEAHPYSNKCFTDYWESPHTAIDIPMNTSHETAQACMSVEKREMGGCCKSYMKECCGKHGHFRSGLTEIVTE

>GbHMA4

MAANKKKLQKSYFDVLGLCCSSEVPLIENILKPLEGVKQVSVIVPTRTVIVVHDNLLVSQLQIVKALNQARLEANVRAHGEIKYQKKWPSPFAMACGLLLLISFFKYVYRPLQWVTVGAVVIGICPILLKGYAAITNFRLDINILMLIAVIGSIAMKDYTEAATIVFLFTVAEWLESRASHKATAVMSSLMSMSPQKAVIAESGEEVDVDEVKLNTVLAVKAGEVIPIDGIVVDGNCEVDEKTLTGESLPVSKQKDSTVWAGTINLNGYISVKTTAVAEDCVVAKMAKLVEEAQNSKSTTQRFIDKCAQFYTPAIIVVSVAIAVIPAAFRVHNLRHWFHLALVVLVSACPCALILSTPVASFCALTKAATSGLLVKGGDYLEILSNIKITAFDKTGTLTRGEFVVTNFRSLCQDISFNSLLYWVSSIESKSSHPMAAALIEYGRSHSIEPKPETVEDYQNFPGEGIYGRIDGRDIYIGSRKVSVRAHGTAPNVEGNMMEGKTIGYVFCGATPAGIFSLSDACRTGAAEAVNELKSMGIKTAMLTGDNQAAAINVQEQLGNRLDVIHADLLPQDKARIVEEFKKEGPTAMIGDGINDAPALATADIGISMGISGSALATETGHVILMSNDIRKIPKAIRLARKAHRKVIQNVILSIITKAAILALAFAGHPLVWAAVLADVGTCLLVIFNSMLLLHGTHKHAGKCCKSSAASHKDKQGCKTSHCHTSHNHEHASIDKKVQKACEPQKCSSRSCASRCQSNPSNSDASSNSCGSNKCRESKGTREMKHCDQGSCNIVNHKIEAHNLPSKCCSSHGKLGEKHCHHSSNQQGTKADHCHSTCGGNHTDRQTLGTFVEHSCLESPKPEAHPYSNKCFTDYRESPHTAIDIPLYTSHETAQACTSVEKREMGGCCKSYMKECCGKHGHFRSGLTEIIAE

>GbECA11

MGKGGENYGKREFVNSKPTDPDVFPAWAKDIHECEKHYDVSQKSGLSTAEVENRRRIYGNNELEKHEGQSIWSLIMEQFNDTLVRILLLAAVVSFVLAWYDGDEGGEMEITAFVEPLVIFLILIVNAIVGVWQENNAEKALEALKEIQSEQATVIRDGIKIPSLPAKELVPGDIVELKVGDKVPADMRVLELVSSTLRVEQGSLTGESEAVNKTNKSVSEDADIQGSLTGESEAVNKTNKSVSEDADIQGKRSMVFAGTTVVNGNCFCLVTQIGMETEIGKVHTQIHVASQSEEDTPLKKKLNEFGEVLTMIIGVICLFVWLINVKYFLSWEYIDGWPRNFKFSFEKCTYYFEIAVALAVAAIPEGLPAVITTCLALGTRKMAQKNALVRKLPSVETLGCTTVICSDKTGTLTTNQMAVAKLVAMGGHASSLRSFRVDGTTYNPSDGKIHGWPSGGMDANLETIAKISAICNDAGVTHSDNKYVAHGMPTEAAIKVLVEKMGLPKGLYSGGAAGNDVLRCCQWWNEYEHRIATLEFDRDRKSMGVIVKSKSGRRSLLVKGAVENLLERSSKMQLLDGSVVPLDQNSRILVSNALQDMSSGALRCLGFAYKDELPEFETYDGSDDHPAHALLLDPSNYPSIESNLTFVGLVGLRDPPRQEVHQAIEDCKAAGIRVMVITGDNKNTAEAICREIGVFGPMEDISSKSLTGKEFMALSDKKAHLRQSGGLLFSRAEPRHKQEIVRLLKEDGEVVAMTGDGVNDAPALKLADIGIAMGIAGTEVAKEASDMVLADDNFSTIVSAVGEGRSIYDNMKAFIRYMISSNIGEVASIFLTAALGIPEGLIPVQLLWVNLVTDGPPATALGFNPPDKDIMKKPPRRSDDSLITAWILFRYLVIGLYVGVATVGIFIIWYTHGSFLGIDLSGDGHTLVTYPQLANWAQCSSWKNFTVSPFTAGNQVFSFENNPCDYFQGGKVKAMTLSLSVLVAIEMFNSLNALSEDGSLLTMPPWVNPWLLLAMSVSFGLHFLILYVPFLAQVFGIVPLSFNEWLLVLAVAFPVILIDEVLKFVGRLGRRMRSSSQRPLKPKTE

>GbECA1

MEDAYARSVSEVLDFFGVDSSKGLTDFQVSQHARLYGKNVLPEEERTPFWKLVFKQFDDLLVKILIAAALVSFLLALINGETGLIAFLEPSVILMILAANAAVGVITETNAEKALEELRAYQADIATVLRNGCFSILPATELVPGDIVEVSVGCKIPADMRMIEMLSGQLRVDQAILTGESSSVEKDLESTIATNAVYQDKTNILFSGTVVVAGRARAVVIGVGANTAMGSIRDSMLRTDDEATPLKKKLDEFGTFLAKVIAGICVLVWIVNIGHFRDPAHGGFLRGAIHYFKIAVALAVAAIPEGLPAVVTTCLALGTKRMARLNAIVRSLPSVETLGCTTVICSDKTGTLTTNMMSVSKICVVHSIKNGPEVAEFGVSGTTYAPEGFIFDNTGVQLEFPAQLPCLLHIAMCSALCNESLLQYNPDKGNYEKIGESTEVALRVLAEKVGLPGFDSMPSALNMLSKHERASYCNHYWENQFKKVSVLEFSRDRKMMSVLCNHKQMEIMFSKGAPESIISRCTNILCNNDGSTIPMDATLRAELDSRFNSFAGKETLRCLALALKIMPMGQQILSFDDEKDLTFIGLVGMLDPPREEVRNAMISCMTAGIRVIVVTGDNKSTAESVCRKIGAFDHLVDYVGHSYTAAEFEELPGTQQTMALQRMALLTRVEPSHKRMLVEALQNQNEVVAMTGDGVNDAPALKKADIGVAMGSGTAVAKSASDMVLADDNFATIVAAVAEGRAIYNNTKQFIRYMISSNIGEVVCIFVAVVLGIPDTLAPVQLLWVNLVTDGLPATAIGFNKPDSDVMKAKPRKVSEAVVSGWLFFRYLVIGAYVGLATVAGFIWWFIYSETGPKLPYTELMNFDTCPTRETTYPCSIFEDRHPSTVAMTVLVVVEMFNALNNLSENQSLLVIPPWSNLWLVASIILTMLLHILILYVPPLSTLFSVTSLSWNEWAVILYLSFPVIIIDEVLKFFSRNSHGIRFNFRFRRYDALPKKELRDK

>GbECA3

MLKFANYYFFTLLNTGTPFWKLVFKQFDDLLVKILIAAALVSFLLALINGETGLIAFLEPSVILMILAANAAVGVITETNAEKALEELRAYQADIATVLRNGCFSILPATELVPGDIVEVSVGCKIPADMRMIEMLSGQLRVDQAILTGESSSVEKDLESTIATNAVYQDKTNILFSGTVVVAGRARAVVIGVGANTAMGSIRDSMLRTDDEATPLKKKLDEFGTFLAKVIAGICVLVWIVNIGHFRDPAHGGFLRGAIHYFKIAVALAVAAIPEGLPAVVTTCLALGTKRMARLNAIVRSLPSVETLGCTTVICSDKTGTLTTNMMSVSKICVVHSIKNGPEVAEFGVSGTTYAPEGFIFDNTGVQLEFPAQLPCLLHIAMCSALCNESLLQYNPDKGNYEKIGESTEVALRVLAEKVGLPGFDSMPSALNMLSKHERASYCNHYWENQFKKVSVLEFSRDRKMMSVLCNHKQMEIMFSKGAPESIISRCTNILCNNDGSTIPMDATLRAELDSRFNSFAGKETLRCLALALKIMPMGQQILSFDDEKDLTFIGLVGMLDPPREEVRNAMISCMTAGIRVIVVTGDNKSTAESVCRKIGAFDHLVDYVGHSYTAAEFEELPGTQQTMALQRMALLTRVEPSHKRMLVEALQNQNEVVAMTGDGVNDAPALKKADIGVAMGSGTAVAKSASDMVLADDNFATIVAAVAEGRAIYNNTKQFIRYMISSNIGEVVCIFVAVVLGIPDTLAPVQLLWVNLVTDGLPATAIGFNKPDSDVMKAKPRKVSEAVVSGWLFFRYLVIGAYVGLATVAGFIWWFIYSETGPKLPYTELMNFDTCPTRETTYPCSIFEDRHPSTVAMTVLVVVEMFNALNNLSENQSLLVIPPWSNLWLVASIILTMLLHILILYVPPLSTLFSVTSLSWNEWAVILYLSFPVIIIDEVLKFFSRNSHGIRFNFRFRRYDALPKKELRDK

>GbECA2

MEDAYARSVSEVLDFFGVDSSKGLTDFQVSQHARLYGKNVLPEEERTPFWKLVFKQFDDLLVKILIAAALVSFLLALINGETGLIAFLEPSVILMILAANAAVGVITETNAEKALEELRAYQADIATVLRNGCFSILPATELVPGDIVEVSVGCKIPADMRMIEMLSGQLRVDQAILTGESSSVEKDLESTIATNAVYQDKTNILFSGTVVVAGRARAVVIGVGANTAMGSIRDSMLRTDDETTPLKKKLDEFGTFLAKVIAGICVLVWIVNIGHFRDPAHGGFLRGAIHYFKIAVALAVAAIPEGLPAVVTTCLALGTKRMARLNAIVRSLPSVETLGCTTVICSDKTGTLTTNMMSVSKICVVHSIKNGPEVAEFGVSGTTYAPEGFIFDNTGVQLEFPAQLPCLLHIAMCSALCNESLLQYNPDKGNYEKIGESTEVALRVLAEKVGLPGFDSMPSALNMLSKHERASYCNHYWENQFKKVSVLEFSRDRKMMSVLCNHKQMEIMFSKGAPESIISRCTNILCNNDGSTIPIDATLRAELDSRFNSFAGKETLRCLALALKIMPMGQQTLSFDDEKDLTFIGLVGMLDPPREEVRNAMISCMTAGIRVIVVTGDNKSTAESVCRKIGAFDHLVDYVGHSYTAAEFEELPGTQQTMALQRMALLTRVEPSHKRMLVEALQNQNEVVAMTGDGVNDAPALKKADIGVAMGSGTAVAKSASDMVLADDNFATIVAAVAEGRAIYNNTKQFIRYMISSNIGEVVCIFVAAVLGIPDTLAPVQLLWVNLVTDGLPATAIGFNKPDSDVMKAKPRKVSEAVVSGWLFFRYLVIGAYVGLATVAGFIWWFIYSETGPKLPYTELMNFDTCPTRETTYPCSIFEDRHPSTVAMTVLVVVEMFNALNNLSENQSLLVIPPWSNLWLVASIILTMLLHILILYVPPLSTLFSVTSLSWNEWAVILYLSFPVIIIDEVLKFFSRNSHGIRFNFRFRRYDALPKKELRDK

>GbHMA10

MESALSVTVPTLALFSIPRALNRHFYYNNCSLIARCIRSRLFPQGRGVTLLASRSYSSPLRSLCAAPVPQRLHRRLECVASSAAYFGAAGGGGVYGGGDGSGSGSGGGGGDGGEGTGDGDLKAKLGAGAVDEPSALSPDIIILDVGGMTCGGCAASVKRILENQPQVSSASVNLTTETAIVWPVSEAKVVPNWQKELGEALARQLTSCGFNSNLRDSGRDNFFKVFERKMDEKRSRLRESGRELAVSWALCAVCLIGHVAHFLGAKASWMHAFHSTGFHLSLSLFTLLGPGRQLIFEGVKNLFKGAPNMNTLVGLGALSSFAVSSLAVLIPKLGWRAFFEEPVMLIAFVLLGRNLEQRAKIKATSDMTGLLSSLPSQARLMVDDSIVEVPCSSLSVGDQIVVLPGDRVPADGIVRAGRSTIDESSFTGEPMPVTKEPGSQVAAGSINLNGTLTIEVRRPGGETAMGDIVRLVEEAQSREAPVQRLADKVAGHFTYGVMALSAATFMFWNLFGARIIPASIYQGSTVSLALQLSCSVLVVACPCALGLATPTAMLVGTSLGATRGLLLRGGNILEKFSMVNVIIFDKTGTLTIGRPVVTKVVTPSGMDHSDSRQHFNGSWSEDDVLKLAAAVESNTIHPVGKAIVEAAQAVKSPNIKVVDGTFVEEPGSGAVAVINDKTVSVGTLEWVQRHGVGDSLLLETDEELRNKSVVYVGVNNKLAGLIYFEDQIREDARHVVDSLYRQGISVYMLSGDKRSTAEYVASIVGIPKDKVLSQVKPDEKRKFVSELQENQNVVAMVGDGINDAAALASAHIGVAMGGGVGAASEVSSIVLMGNRLSQLLDALALSQLTMKTVNFQAQLTVTVLQFSGNIHVHYICLDSDDVLSIYLLAVIFMQRAFLQVVDGTFVEEPGSGAVAVINDKTVSVGTLEWVQRHGVGDSLLLETDEELRNKSVVYVGVNNKLAGLIYFEDQIREDARHVVDSLYRQGISVYMLSGDKRSTAEYVASIVGIPKDKVLSQVKPDEKRKFVSELQENQNVVAMVGDGINDAAALASAHIGVAMGGGVGAASEVSSIVLMGNRLSQLLDALALSQLTMKTVKQNLWWAFAYNIVGIPIAAGTLLPLTGTMLTPSIAGALMGLSSIGVVTNSLLLRFRFSLQQQQAYRSSLQPPPPPPPYAAVDINNDLAMDHSKAKLKKPDSIT

>GbHMA11

MESALSVTVPTLALFSIPRALNRHFFYNNSSLIARCIRSRLFPQGRGVTLLASRSYSSPLRSLCSASVPQRLHRRLECVASSAAYFGAAGGGGVYGGGDGSGSGSGGGGGDGGEGTGDGDLKAKLGAGAVDEPSALSPDIIILDVGGMTCGGCAASVKRILENQPQVSSASVNLTTETAIVWPVSEAKVVPNWQKELGEALARQLTSCGFNSNLRVPQRLHRRLECVASSAAYFGAAGGGGVYGGGDGSGSGGGGGDGGEGTGDGDLKAKLGAGAVDEPSALSPDIIILDVGGMTCGGCAASVKRILENQPQVSSASVNLTTETAIVWPVSEAKVVPNWQKELGEALARQLTSCGFNSNLRDSGRDNFFKLFELKMDEKRSRLKESGRELAVSWALCAVCLIGHVAHFLGAKASWMHAFHSTGFHLSLSLFTLLGPGRQIIFEGVKNLFKGAPNMNTLVGLGALSSFAVSSLAVLIPKLGWRAFFEEPVMLIAFVLLGRNLEQRAKIKATSDMTGLLSSLPSQARLMVDDSIVEVPCSSLSVGDQIVVLPGDRVPADGIVRAGRSTIDESSFTGEPMPVTKEPGSQVAAGSINLNGTLTIEVRRPGGETAMGDIVRLVEEAQSREAPVQRLADKVAGHFTYGVMALSAATFMFWNLFGARIIPASIYQGSAVSLALQLSCSVLVVACPCALGLATPTAMLVGTSLGATRGLLLRGGNILEKFSMVNVIIFDKTGTLTIGRPVVTKVVTPSRMDHSDSRQHFDGSWSEDDVLKLAAAVESNTIHPVGKAIVEAAQAVKSPNIKVVDGTFVEEPGSGAVAVIDDKTVSVGTLEWVQRHGVGDSLLLETDEELRNKSVVYVGVNNKLAGLIYFEDQIREDARHVVDSLHRQGISVYMLSGDKRSTAEYVASIVGIPKDKVLSQVKPDEKRKFVSELQENQNVVAMVGDGINDAAALASAHIGVAMGGGVGAASEVSSIVLMGNRLSQLLDALALSQLTMKTVKKNLWWAFAYNIVGIPIAAGTLLPLTGTMLTPSIAGALMGLSSIGVVTNSLLLRFRFSLQQQQAYRSSLQPPPHAAMDINNDLAKDHSRAKLKKPDSIT

>GbHMA19

MATKLLALACIRKESYGDLSPRPHYPSMPKYPKGVTAQETSLQGSEAKAMFSVMGMTCSACAGSVEKAVKRLPGIKEAVVDVLNNKAQVMFYPSFVNEESIREAIEDAGFQAALIQDETDDKSVQVCRIRINGMTCTSCSSTLENALQAVPGVQKVQVALATEEAQIHHDPKIITYNQLMEQIEETGFGAVLVSTGEDMSKINLRIDGVRTVNSMRMLENSLQALPGVQAVQTSPELKKIAVSYKPDMTGPRNFIKVIDSTGSSRRFKATIYPEGEGAGRESHRKEEIKQYFRSFLWSLIFTTPVFLTSMVFMYIPGIKHGLDTKVVNMLTIGEVIRWVLSTPVQFIIGRRFYTGSYKALRHGSANMDVLIALGTNAAYFYSVYTVIRAASSPDFEGTDFFETSAMLISFILLGKYLEVLAKGKTSEAIAKLMNLAPETAILLSLDEEGNVISEEETDSRLIQKNDIIKIIPGAKVASDGFVLWGQSHINESMITGEARPVAKRKGDTVIGGTVNENGVLHIKATKVGSESALAQIVRLVESAQMAKAPVQKFADRISKYFVPLVIMLSFSTWLAWFLAGKLHGYPESWIPSSMDSFELALQFGISVMVIACPCALGLATPTAVMVGTGVGASLGVLIKGGQALEGAHKVNCIVFDKTGTLTVGKPVVVNTRLLKNMVLHEFYELVAATEVNSEHPLAKAIIEYAKKFREDEENPAWPEARDFVSITGHGVKAIVRNKEVIVGNKSLMLENNIVIPVDAQDMLTETESMAQTGILVSIDGEVTGVLAISDPVKPGAQEVISILKSMNVRSIMVTGDNWGTASSIASQIGIETVVAEAKPEQKAEKVKELQAEGYAVAMVGDGINDSPALVAADVGMAIGAGTDIAIEAADIVLMKSNLEDVITAIHLSRKTFSRIRLNYIWALGYNILGIPIAAGALFPSTGFRLPPWIAGAAMAASSVSVVCCSLLLKNYERPKKLENLEIGGIQIE

>GbACA31

MSSSDECKLYDCSTSLLNVTAPSGFTVAQRRWRIAYIRIYSSRVMLSLADKIISQRATQLPSMTSQQFDHYVAEFDHKINQKRLVKTVKEKDLVSLNHLGGVDGVVDALCTNSEHGIRDDEQEVIKRQEMFGFNKYHKPPPKGLLYFVLEAFKDTTILILLVCAALSLGFGIKEHGAEEGWYEGGSIFVAVFLVIVVSALSNFRQETQFDKLSKISNNIKVEVVRSGRRQQISIFDLVAGDVVFLKIGDQIPADGLFLDGHSLQVDESSMTGESDHVEVDACRNPFLSSGSKVVDGYARMLVVSVGMDTAWGEMMSSITSDKNERTPLQARLDKLTSSIGKVGLVVAFLVLAVLLIRYFTGNTKDDNGQTEYRGSETDVDDILNAVVRIIAAAVTIVVVAIPEGLPLAVTLTLAYSMKRMMADQAMVRKLSACETMGSATIICTDKTGTLTLNQMKVTQFWLGQESVEEDLAKKIAPSVLELFYQGVGLNTTGSVCKPVSGSLPEFSGSPTEKAILSWGVLGLDMNIEKLKQQYSILHVETFNSEKKRSGVSVQKKADEMVHIHWKGAAEMIVAMCSQYYESNGTIRSMNEDGRDRIETIIQSMAASSLRCIAFAHKQVLKGETEDGDDQSRKTNRGIKEDGLTLLGIVGLKDPCRPGVKKAVQACQSAGVSIKMITGDNMFTAKAIATECGILGPDYQQGSGEVVEGIEFRNYAPDERMEKVEKIRVMARSSPFDKLLMVQCLKQKGHVVAVTGDGTNDAPALKEADIGLSMGIQGTEVAKESSDIVILDDNFSSVATVLRWGRCVYNNIQKFIQFQLTVNVAALVINFIAAVSAGEVPLTTVQLLWVNLIMDTLGALALATDRPTNELMEKPPVGRTEPLITNIMWRNLLAQALYQIAILLILQFRGESIVNVPETVKDTLIFNTFVLCQVFNEFNARKLEKQNVFEGILKNRLFLGIIGVTIVLQVVMVEFLKKFADTEQLKLWQWGVCILLAAFSWPIAWFVKLIPVSNTPFFSYLKRLRIIYTIVKRPINHQKP

>GbHMA14

MSPGSRDLQLTSQAAGVWRSTYPSSVRAVDPDDMEEGTRLLDSYETGDYKLESIEEGSMRRIQVTVTGMTCAACSNSVEAALKNINGVLRASVALLQNRADVVFDPTLVKDEDIKNAIEDAGFEAEILPEPSNVGTKPRGVLVGQFTIGGMTCAACVNSVEGILRDLPGVSRAVVALATSLGEVEYDPTVISKDDIVNAIEDAGFEASLVQSSEQDKIILGVAGVFNELDVQLIEGILSSLKGVRQFRFDRSSGELEVLFDPEVVSSRSLVDGIEGGSKGKFKLHVMNPYARMTTKDEETSIMFQLFTSSLFLSIPVFLIRVVCPHIPLLDAFLLWRCGPFLMGDWLKWALVSVVQFVIGKRFYVAAGRALRNGSTNMDVLVALGTSASYFYSVGALLYGAITGFWSPTYFETSSMLITFVLLGKYLECLAKGKTSDAIKKLVELAPATALLVVKDNGGNIIGEREVDALLVQPGDILKVLPGAKLPADGIVVWGSSYVNESMVTGESVPVSKEVDSPVIGGTINLHGALHIKATKIGSEAVLSQIISLVETAQMSKAPIQKFADFVASIFVPTVVTLSLITLLGWYVGGAAGAYPEQWLPENGNYFVFALMFSISVVVIACPCALGLATPTAVMVATGVGASNGVLIKGGDALERAQKVQYVIFDKTGTLTQGKAKVTTVKVFSEMDRGEFLTLVASAEASSEHPLAKAIVEYARHFHFFDENSLTEDAEYSSKGSPISAWLLDVAEFSAVPGRGIQCFIDGKQVLVGNRKLLTESGVSISAHVEQFVVDLEERARTGILAAYDGNVIGVLGVADPLKREAAVVVQGLQKMGVRPVMVTGDNWRTAQAVAREVGIRDVRAEVMPAGKAEVVRSFQKDGSIVAMVGDGINDSPALAAADVGMAIGAGTDIAIEAADYVLMRNNLEDVITAIDLSRKTFSRIRWNYVFAMAYNVVAIPIAAGVLYPSLGIKLPPWAAGACMALSSVSVVCSSLLLRRYKKPRLTTILEITVE

>GbACA28

MYDCGTLLFKVTTSGFTTAQKRWRIAYASIYSVRVMLSIAKEIISKRGIEQPSIISDLHPYVALDVEPSSSPHWGEKFSSSSFAPKIDRKRLVETVKEKDLVSLHQLGGVEGIAAALGTNPEKGIRDDDRDVVKRQEMFGTNTYHKPPPKGLLYFVLDAFKDTTILILLVCAALSLGFGIKEHGAAEGWYEGGSIFVAVFLVIVVSALSNFRQETQFDKLSKISNNIKVEVVRGGRRRQVSIFDLVVGDVVFLKIGDQIPADGLFLDGYSLQVDESSMTGESDHMEVDATRNPFLFSGSKVADGYGQMLVASVGIDTTWGEMMSSITSDKNERTPLQERLDRLTSSIGKVGLAVAFLVLVVLLIRYFTGNTEDDNGNTEYIGSKTSVDDILNAVVRIVSAAVTIVVVAIPEGLPLAVTLTLAYSMKRMMADQAMVRKLSACETMGSATIICTDKTGTLTLNQMKVTQFWLGQESIKEDHSNIIDHAVLELFYQGVGLNTTGSVCKPVSGSLPEFCGSPTEKAILSWAALGLDLDMEKLKQKYSILHVETFNSEKKRSGVSVRRKTDETLHVHWKGAAEIIVAMCSDYYESNGGIRSMDEDQRSRIETIIQSMAASSLRCIAFAHKQVSQKEMECVDDSEKTHQRIKEDGLTLLGIVGLKDPCRPGVKKAVEACKSAGVDIKMITGDNIFTAKAIAAECGILGADYNEESGQAIEGIGFRNYTPEERMEKIGKIKVMARSSPFDKLLMVQCLKQKGDVVAVTGDGTNDALALKEADIGLSMGIQGTEVAKESSDIVILDDNFSSVATVLRWGRCVYNNIQKFIQFQLTVNVAALVINFIAAVSAGEVPLTAVQLLWVNLIMDTLGALALATDRPTKELMKKPPVGRTEPLITNVMWRNLLAQAVYQIAILLILQFRGESMFNVAKRVKDTLIFNTFVLCQVFNEFNARKLEKQNVFQGILQNRLFLGIVGITIILQVVMVEFLKKFADTERLELWQWGVCILFAAFSWPIAWVVKLIPVSDKPFFSYLKRSKSSSQLNKSSTTRNLQSAGMELEVQ

>GbHMA12

MSPGSRDLQLTSQAAGVWRSTYPSSVRADDPDDMEEGTRLLDSYETGDYKLESIEEGSMRRIQVTVTGMTCAACSNSVEAALKSINGVLRASVALLQNRADVVFDPNLVKDEDIKNAIEDAGFEAEILLEPSNVGTKPRGVLVGQFSIGGMTCAACVNSVEGILRDLPGVSRAVVALATSLGEVEYDPTVISKDDIVNAIEDAGFEASLVQSSEQDKIILGVAGVFNELDVQLIEGILSSLKGVRQFRFDRSSGELEVLFDPEVVSSRSLVDGIEGGSKGKFKLHVMNPYARMTTKDEETSIMFQLFTSSLFLSIPVFLIRVVCPHIPLLDAFLLWRCGPFLMGDWLKWALVSVVQFVIGKRFYVAAGRALRNGSTNMDVLVALGTSASYFYSVGALLYGAITGFWSPTYFETSSMLITFVLLGKYLECLAKGKTSDAIKKLVELAPATALLVVKDNGGNIIGEREVDALLIQPGDILKVLPGAKLPADGVVVWGSSYVNESMVTGESVPVSKEVDSPVIGGTINLHGALHIKATKIGSEAVLSQIISLVETAQMSKAPIQKFADFVASIFVPTVVTLSLITLLGWYAGGAAGAYPQQWLPENGNYFVFALMFSISVVVIACPCALGLATPTAVMVATGVGASNGVLIKGGDALERAQKVQYVIFDKTGTLTQGKAKVTTVKVFSEMDRGEFLTLVASAEASSEHPLAKAIVEYARHFHFFDENSLTEDAQYSSKESPISAWRLDVAEFSAVPGRGIQCFIDGKRVLVGNRKLLTESGVSISAHVEQFVVDLEESARTGILAAYDGNVIGVLGVADPLKREAAVVVQGLQKMGVRPVMVTGDNWRTAQAVAREVGIRDVRAEVMPAGKAEVVRSFQKDGSIVAMVGDGINDSPALAAADVGMAIGAGTDIAIEAADYVLMRNNLEDVITAIDLSRKTFSRIRWNYVFAMAYNVVAIPIAAGVLYPSLGIKLPPWAAGACMALSSVSVVCSSLLLRRYKKPRLTTILEITVE

>GbHMA15

MSPGSRDLQLTSQAAGVWRSTYPSSVRADDPDDMEEGTRLLDSYETGDYKLESIEEGSMRRIQVTVTGMTCAACSNSVEAALKSINGVLRASVALLQNRADVVFDPNLVKIPVFLIRVVCPHIPLLDAFLLWRCGPFLMGDWLKWALVSVVQFVIGKRFYVAAGRALRNGSTNMDVLVALGTSASYFYSVGALLYGAITGFWSPTYFETSSMLITFVLLGKYLECLAKGKTSDAIKKLVELAPATALLVVKDNGGNIIGEREVDALLIQPGDILKVLPGAKLPADGVVVWGSSYVNESMVTGESVPVSKEVDSPVIGGTINLHGALHIKATKIGSEAVLSQIISLVETAQMSKAPIQKFADFVASIFVPTVVTLSLITLLGWYAGGAAGAYPQQWLPENGNYFVFALMFSISVVVIACPCALGLATPTAVMVATGVGASNGVLIKGGDALERAQKVQYVIFDKTGTLTQGKAKVTTVKVFSEMDRGEFLTLVASAEASSEHPLAKAIVEYARHFHFFDENSLTEDAQYSSKESPISAWRLDVAEFSAVPGRGIQCFIDGKRVLVGNRKLLTESGVSISAHVEQFVVDLEESARTGILAAYDGNVIGVLGVADPLKREAAVVVQGLQKMGVRPVMVTGDNWRTAQAVAREVGIRDVRAEVMPAGKAEVVRSFQKDGSIVAMVGDGINDSPALAAADVGMAIGAGTDIAIEAADYVLMRNNLEDVITAIDLSRKTFSRIRWNYVFAMAYNVVAIPIAAGVLYPSLGIKLPPWAAGACMALSSVSVVCSSLLLRRYKKPRLTTILEITVE

>GbHMA13

MSPGSRDLQLTSQAAGVWRSTYPSSVRADDPDDMEEGTRLLDSYETGDYKLESIEEGSMRRIQVTVTGMTCAACSNSVEAALKSINGVLRASVALLQNRADVVFDPNLVKDEDIKNAIEDAGFEAEILLEPSNVGTKPRGVLVGQFSIGGMTCAACVNSVEGILRDLPGVSRAVVALATSLGEVEYDPTVISKDDIVNAIEDAGFEASLVQSSEQDKIILGVAGVFNELDVQLIEGILSSLKGVRQFRFDRSSGELEVLFDPEVVSSRSLVDGIEGGSKGKFKLHVMNPYARMTTKDEETSIMFQLFTSSLFLSIPVFLIRVVCPHIPLLDAFLLWRCGPFLMGDWLKWALVSVVQFVIGKRFYVAAGRALRNGSTNMDVLVALGTSASYFYSVGALLYGAITGFWSPTYFETSSMLITFVLLGKYLECLAKGKTSDAIKKLVELAPATALLVVKDNGGNIIGEREVDALLIQPGDILKVLPGAKLPADGVVVWGSSYVNESMVTGESVPVSKEVDSPVIGGTINLHGALHIKATKIGSEAVLSQIISLVETAQMSKAPIQKFADFVASIFVPTVVTLSLITLLGWYAGGAAGAYPQQWLPENGNYFVFALMFSISVVVIACPCALGLATPTAVMVATGVGASNGVLIKGGDALERAQKVQYVIFDKTGTLTQGKAKVTTVKVFSEMDRGEFLTLVASAEASSEHPLAKAIVEYARHFHFFDENSLTEDAQYSSKESPISAWRLDVAEFSAVPGRGIQCFIDGKRVLVGNRKLLTESGVSISAHVEQFVVDLEESARTGILAAYDGNVIGVLGVADPLKREAAVVVQGLQKMGVRPVMVTGDNWRTAQAVAREVCTHTYIHTCIHAYMH

>GbACA29

MSSSDGNQMYDCGTLLFKVTTSGFTTAQKRWRIAYASIYSVRVMLSLAKEIISKRGIEQPSIISDLHPYVALDVEPSSSPHWGEKFSSSSFAPKIDRKRLVETVKEKDLVSLHQLGGVEGIAAALGTNPGKGIRDDDREVVKRQEMFGTNTYHKPPPKGLLYFVLDAFKDTTILILLVCAALSLGFGIKEHGAAEGWYEGGSIFVAVFLVIVVSALSNFRQETQFDKLSKISNNIKVEVVRGGRRRQVSIFDLVVGDVVFLKIGDQIPADGLFLDGYSLQVDESSMTGESDHMEVDVTRNPFLFSGSKVADGYGQMLVASVGIDTTWGEMMSSITSDKNERTPLQERLDRLTSSIGKVGLAVAFLVLVVLLIRYFTGNTEDDNGNTEYIGSKTSVDDILNAVVRIVSAAVTIVVVAIPEGLPLAVTLTLAYSMKRMMADQAMVRKLSACETMGSATIICTDKTGTLTVNQMKVTQFWLGQESIKEDHSNIIDHTVLELFYQGVGLNTTGSVCKPVSGSLPEFSGSPTEKAILSWAVLGLDLDMEKLKQKYSILHVETFNSEKKRSGVSVRRKTDETLHVHWKGAAEIIVAMCSDYYESNGGIRSMDEDQRSRIETIIQSMAASSLRCIAFAHKQVSQKEMERVDDSEKTHQRIKEDGLTLLGIVGLKDPCRPGVKKAVEACKSAGVDIKMITGDNIFTAKAIAAECGILGADYNEESGQAIEGIEFRNYTPEERMEKIGKIKVMARSSPFDKLLMVQCLKQKGDVVAVTGDGTNDALALKEADIGLSMGIQGTEVAKESSDIVILDDNFSSVATVLRWGRCVYNNIQKFIQFQLTVNIAALVINFIAAVSAGEVPLTAVQLLWVNLIMDTLGALALATDRPTKELMKKPPVGRTEPLITNVMWRNLLAQAVYQIAILLILQFREGKGHTDFQYFRSLPSFQRIQRKEVGEAKRLPGHSSEQIVSGNRGDNHHSSGGYGGISKEFCRYREIETMAVGALYLIRSFLMANCMGCEAHTCFRQTLLQLSQEIKTTLLGIVGLKDPCRPGVKKAVEACKSAGVDIKMITGDNIFTAKAIAAECGILGADYNEESGQAIEGIEQETFNLQAWN

>GbACA21

MPLKVKTEISPVCSFSLLINVLRLRKPSEPTMHRQVEPSKSSVRRWRVAVTAISVTRFLVGLTKKVVENNAELLRSLSFVTIDVEGSGDERVPILDVDPQGLAKMVKDKSFQSLNDQYGGVKQVATLLQTDFKKGIPGDDNDLALRTKVFGANKYQKQPAKSFFSFVLEAFKDTIIIILLVCAVLSLGFGIKQHGLKEGWYDGGSIIVAVVLVVVVSAVSNYRQSKQFEELSHETNDIRVQVVRNGRYQPVSIFELVVGDIVSLKTGDQIPADGLFVEGHSLKVDESSMTGESDHVEVNEKKNPFLLSGTKVTDGHGYMLVTAVGTNTAWGEMMSSIRRDLNEETPLQARLSKLTSYIGNIGLSVAVLVLLVLLIRYFTGHTKAENGRSAFNGSRTKFDDVMNSVVGIIAAAVTIVVVAIPEGLPLAVTLTLAYSMKRMMREHAMVRKLSACETMGSATIICTDKTGTLTLNEMKVTEFWLGKEPIDNSMSSEIAPNVLQLLSEGVGLNTTGTVYKPKPTSVPEIYGSPTEKAILSWALDDMGLNIDESKQSCEIIHVEAFNSEKKRSGVLIRRSNNKRVLATHWKGAAEMLLAMCSCYYDKKGVSKFMNEDEREHIGMVIESMAAKSLRCIAFATSDVTVTDGNEENHTKLEERGLTWLGLVGLKDPCRPGVKQAVESCQNAGVSIKMITGDNMHTARAIAFECGILNSESSLHNEAVVEGVQFRNYSEEERRQKIETIRVMARSSPFDKLLMVQCLKQKGHVVAVTGDGTNDAPALKEADIGLSMGIQGTEVAKESSDIVILDDNFTSVATVLRWGRCVYNNIQKFIQFQLTVNVAALVINFIAAVSSGDVPLTAVQLLWVNLIMDTLGALALATEQPTNDLMDKRPVGRTELLITKVMWRNLIAQALYQVTILLILQFKGKSIFGVSEEVKDTLIFNTFVLCQIFNEFNARNMDKKNIFKGIHKNRLFLAIIGITLVLQAIMVEFLQRFANTERLSWEQWGACIGIAALTWPIGWIVKCIPVDKKVQTRSSAAS

>GbACA14

MEELLKGFEVPPKNSSEAALRRWRKLVTIVRNPRRRFRMIANLEKRSEAEQQKLKIKEKIRVALIVQKAALQFIDAAGPLDYKITDEVRQANFGIEPDELASIVHGHDIKRLKSHGGVDGIAEKVTVSLDEGVLSENVSTRQRIYGFNQYTEKPPRTFWMFVWDALQDLTLIILMICAVVSIGVGLATEGWPKGMYDGAGILLSIILVVLVTAISDYRQSLQFRDLDREKKKISVQVTRDGRRQQVSIYDLVVGDVVHLGIGDQVPADGLFISGYSMQIDESSLSGETDLVDIYEQKPFLLSGTKVRDGSAKMLVTAVGMRTEWGKLMETLNEGGDDETPLQVKLNGVATIIGKIGLTFAVLTFLVLTVRFLIEKALHNEFTKWSSTDALTLLDYFAIAVTIIVVAVPEGLPLAVTLSLAFAMKQLMDERALVRHLSACETMGSASCICTDKTGTLTTNHMVVNKIWICEKISNIGDNENKNIDELEIHESVFSIFLRSIFLNSSAEVVKDENGKNSILGTPTETALLEFGLLLSADHDAYRRRFKILKVEPFNSDRKKMSVLVALPEGRIQAFCKGAPEIVLRMCEKVVDSSGEVVLLSEERVRDITEAINGFASDALRTLCVAVKDVGETFNENGIPDSGYTLIAVFGIKDPVRPGVKEAVQTCLAAGITVRMVTGDNINTAKAIAKECGILTAEENAIEGPEFSSKSPDEMKDIIPNIQVMARSKPSDKLNFVTNLRNMFGEVVAVTGDGTNDAPALRQSDIGLAMGIAGTEVAKENADVIVMDDNFATIVNVAKWGRAVYINIQKFVQFQLTDLPLSQLYNCFGLI

>GbACA12

MEDYLRKNFAVEPKRPSEEALRRWRSAVALVKNRSRRFRMVADLAKRAEADRRRKIIQVFNFFFNYITICSGNCPNSFFTYEKIRVALYVQKAALNFIDAGKQAERKLPEDVREAGFHIGADELASIVRSHDMSSFEENGGVEGLAKKVSVSLTNGVVPTDISFRKNIYGNNKFDEKPARSFWTFVWEALHDLTLIILIVCAVVSIGVGVATEGWPGGLYDGLGIVLCIFLVVFVTAISDYKQSLQFKDLDKEKKNILVQVTREGCRQKISIYDLVVGDIVHLSIGDQVPADGVLISGYSLSIDESSLSGESEPVKVTQERPFLLSGTKVQDGSGKMLVTTVGMRTEWGRLMVTLSEGGVDETPLQVKLNGVATVIGKIGLVFAVLTFLVLAIRFMVTKAQLGEIEKWVMSDVLSLLNFFAVAVTIIVVAVPEGLPLAVTLSLAFAMKKLMSDKALVRHLSACETMGSATCICTDKTGTLTTNHMVVDKIWTCGRTISIAGDNKREDVLRSSIAGEVLDLLLQSIFQNTGAEVVKGKDGKNNILGSPTETAILEFGLLLGGEFKKHRKESTILKVEPFNSEKKRMSVLVSLSNGGENRAFCKGASEIILESCNKVINVDGKAEHLSKEQKKFITDVINGFACEALRTLCLAFKDVKDTSDVDSDSIPQENYTLIAVIGIKDPVRPGVRQAVETCLSAGIKVRIVTGDNINTAKAIARECGILTENGLAIEGPEFRDMSPRQMEETIPKLQVLARSLPLDKHKLVTYLRMEFKEVVAVTGDGTNDAPALHEADIGLAMGIAGTEVAKENADVIIMDDNFATIQNVARWGRAVYINIQKFVQFQLTVNIVALMLNFVSACISGSAPLTAVQLLWVNMIMDTLGALALATEPPHEGLMKRPPIGRDVAFITRVMWRNIIGQTIYQLIVLAILKFDGKRLLKISGSNATAILNTLIFNSFVFCQVFNEINSRDMEKINVFRGFFDSWLFIMVMVCTVGFQSIIVELLGTVADTVPLSWELWLTSILLGAGSLIVAVILKCIPVENCKEASTTKHHDGYEPLPTGPDMA

>GbACA13

MSSFEEHGGVEGLAKKVSVSLTNGVVPTDISFRQNIYGNNKFDEKPARSFWMFVWEALHDLTLIILIVCAVVSIGVGVATEGWPGGLYDGLGIVLCIFLVVFVTAISDYKQSLQFKDLDKEKKNILVQVTREGCRQKISIYDLVVGDIVHLSIGDQVPADGVLISGYSLSIDESSLSGESEPVKVTQERPFLLSGTKVQDGSGKMLVTTVGMRTEWGRLMVTLSEGGVDETPLQVKLNGVATVIGKIGLVFAVLTFLVLTIRFMVTKAQLGEIEKWGMSDVLVLLNFFAVAVTIIVVAVPEGLPLAVTLSLAFAMKKLMSDKALVRHLSACETMGSATCICTDKTGTLTTNHMVVDKIWTCGRTISIAGDNKREDVLRSSIAGEVLDLLLQSIFQNTGAEVVKGKDGKNNILGSPTETAILEFGLLLGGEFKKYRKESTILKVEPFNSEKKRMSVLVSLSNGGENRAFCKGASEIILESCNKVINVDGKAEHLSKEQKKYITDVINGFACEALRTLCLAFKDVKDTSDVHSDSIPQENYTLIAVIGIKDPVRPGVRQAVETCLSAGIKVRIVTGDNINTAKAIARECGILTENGLAIEGPEFRDMSPRQMEETIPKLQVLARSLPLDKHKLVTYLRKEFKEVVAVTGDGTNDAPALHEADIGLAMGIAGTEVAKENADVIIMDDNFATIQNVARWGRAVYINIQKFVQFQLTVNIVALMLNFVSACISGSAPLTAVQLLWVNMIMDTLGALALATEPPHEGLMKRPPIGRDVAFITRVMWRNIIGQTIYQLIVLAILKFDGERLLKISGSNATAILNTLIFNSFVFCQVLPLNRYILFITF

>GbACA22

MSLRLRKPTEPTMHRQVEPSKSSVRRWRVAVTAISVTRFLVGLTKKVAEKNAVLLRSLSFVTIDVEGSGDERVPILDVDPQGLAKMVKDKSFQSLNDQYGGVKQVATLLQTDFKTGIPGDDNDLALRTKVFGANKYQKQPAKSFFSFVLEAFKDTIIIILLVCAVLSLGFGIKQHGLKEGWYDGGSIIVAVVLVVVVSAVSNYRQSKQFEELSHETNDIRVQVVRNGRYQPVSIFELVVGDIVSLKTGDQIPADGLFVEGHSLKVDESSMTGESDHVEVNEKKNPFLLSGTKVTDGHGYMLVTAVGTNTAWGEMMSSIRRDLNEETPLQVRLSKLTSYIGNIGLSVAVLVLLVLLIRYFTGHTKAENGRSAFNGSRTKFDDVMNSVVGIIAAAVTIVVVAIPEGLPLAVTLTLAYSMKRMMRDHAMVRKLSACETMGSATIICTDKTGTLTLNEMKVTEFWLGKEPIDNSMSSEIAPNVLQLLSEGVGLNTTGTVYKPEPTSVPEIYGSPTEKAILSWALDDMGLNIDESKQSCEIIHVEAFNSEKKRSGVLIRRSNNKKVLATNWKGAAEMLLAMCSCYYDKKGVSKFMNEDERAHIGMVIESMAAKSLRCIAFATSDVTVTDGNEENHTKLEETGLTWLGLVGLKDPCRPGVKQAVESCKKAGVSIKMITGDNMHTARAIAFECGILNSESSLHNEAVVEGVQFRNYSEEERRQKIETIRVMARSSPFDKLLMVQCLKQKGHVVAVTGDGTNDAPALKEADIGLSMGIQGTEVAKESSDIVILDDNFTSVATVLRWGRCVYNNIQKFIQFQLTVNVAALVINFIAAVSSGDVPLTAVQLLWVNLIMDTLGALALATEQPTNDLMDKRPVGRTELLITKVMWRNLIAQALYQVAILLILQFKGKSIFGVSEEVKDTLIFNTFVLCQIFNEFNARNMDKKNIFKGIHKNRLFLAIIGITLVLQAIMVEFLQRFANTERLSWEQWGACIGIAALTWPIGWIVKCIPVDKKVQTRSSAAS

>GbACA15

MEELLKDFEVPPKNSSEAALRRWRKLVTIVRNPRRRFRMIANLEKRSEAEQQKLKIKEKIRVALIVQKAALQFIDAAGPPDYKITDEVRQAKFGIEPDELASIVHGHDIKRLKSHGGVDGIAEKVTVSLDEGVCSENVSTRQRIYGFNRYTEKPPRNFWMFVWDALQDLTLIILMICAVVSIGVGLATEGWPKGMYDGAGILLSIILVGLVTAISDYRQSLQFRDLDREKKKISVQVTRDGRRQQVSIYDLVVGDVVHLGIGDQVPADGLFISGYSVQIDESSLSGETDLVDIYEQKPFLLSGTKVRDGSAKMLVTAVGMRTEWGKLMETLNEGGDDETPLQVKLNGVATIIGKIGLTFAVLTFLVLTVRFLIEKALHNEFTKWSSTDALTLLDYFAIAVTIIVVAVPEGLPLAVTLSLAFAMKQLMDERALVRHLSACETMGSASCICTDKTGTLTTNHMVVNKIWICEKISNIGDNENKNIDELEIHESVFSILLRSIFLNSSAEVVKDENGKNSILGTPTETALLEFGLLLSADLDAYRRQFKILKVEPFNSDRKKMSVLVALPEGRIQAFCKGAPEIVLRMCEKVVDSSGEVVLLSEERVRDITEAINGFASDALRTLCVAVKDVGETFNENGIPDSGYTLIAVFGIKDPVRPGVKEAVQTCLAAGITVRMVTGDNINTAKAIAKECGILTAEENAIEGPEFSSKSPDEMKDIIPNIQVMARSKPSDKLNFVINLRNMFGEVVAVTGDGTNDAPALRQSDIGLAMGIAGTEVAKENADVIVMDDNFATIVNVAKWGRSVYINIQKFVQFQLTVNVVALIINFVSACISGSAPLTAVQLLWVNMIMDTLGALALATEPPNDALMKRPPVPRGASFITKPMWRNIIGQSIYQLIVLGVLNFDGKQLLKLTGSDATTVLNTVIFNSFVFCQVFNEINSREIEKINIFRGMFSSWIFLGVMASTVAFQVVIVEFLGTFASTVPLSWQLWLLCILIGSVSLIVGVIVKCIPVERAAVKPKHHDGYDALPSGPELA

>GbHMA6

MAMAGDLLRLTVLNRPKLSLGNGAKLKADRFGLLKRCPRGRFHCQPRSTPGFVLFSSLETRLESEESSIQPVGQKLKDPSVLLDVNGMMCGGCVSRVKLVISSDERVESVVVNLLTETAAIKLKREVMERETAESVAESIAQRVSECGFMAKMRVSGTGIAENMRKWQEMLKKKEELLVKSRNRVAFAWTLVALCCGAHASHILHSLGIHFGHGSFLEILHNSYVKGGLALTALLGPGRDLLVDGLLAFKKGSPNMNSLVSLLNPGLKWDASFFDEPVMLLGFVLLGRSLEEKARIRASSDMNELLSLISTRSRLVITSSDTDSSADSVLSSDAICIEVPSDDIRVGDSVLVLPGETIPVDGKVLTGRSVVDESMLTGESLPVFKEKGLMVSAGTINWDGPLRIEATSTGSNSTIAKIVRMVEDAQGQEAPVQRLADAIAGPFVYSIMTLSAATFAFWYYAGSHIFPDVLLNDIAGPDGDSLLLSLKLAVDVLVVSCPCALGLATPTAILVGTSLGARQGLLIRGGDVLERLANVDRIAFDKTGTLTEGKPTVSSVSSFTYDESEILQIAAAVERTATHPIAKAIVKKAELLNLLLPETRGQLVEPGFGTLAEVNGCLVAVGKLEWVNERFQIKASPSDLMALEHAVMRQSSSPSNYSKTAIYVGREGEGVIGAIGMSDSLRFDAESTVSRLQRKGIKTILISGDREEAVATIAKTVGIDHEFVNASLTPQQKSRVISTLQTAGHHIAMVGDGINDAPSLAIADVGIALQTEAQETAASDAASIILLGNRLSQVVDALDLAQATMAKVYQNLSWAVAYNIVAIPIAAGVLLPQYELAMTPSFSGGLMALSSIFVVTNSLLLRLHGSEKSWKNSIAKISQMPAGPG

>GbHMA7

MAMAGDLLRLTVLNRPKLSLGNGAKLKADRFGLLKRCPRGRFHCQPRSTPGFVLFSSLETRLESEESSIQPVGQKLKDPSVLLDVNGMMCGGCVSRVKLVISSDERVESVVVNLLTETAAIKLKREVMERETAESVAESIAQRVSECGFMAKMRVSGTGIAENMRKWQEMLKKKEELLVKSRNRVAFAWTLVALCCGAHASHILHSLGIHFGHGSFLEILHNSYVKGGLALTALLGPGRDLLVDGLLAFKKGSPNMNSLVGFGSIVAFIISAVSLLNPGLKWDASFFDEPVMLLGFVLLGRSLEEKARIRASSDMNELLSLISTRSRLVITSSDTDSSADSVLSSDAICIEVPSDDIRVGDSVLVLPGETIPVDGKVLTGRSVVDESMLTGESLPVFKEKGLMVSAGTINWDGPLRIEATSTGSNSTIAKIVRMVEDAQGQEAPVQRLADAIAGPFVYSIMTLSAATFAFWYYAGSHIFPDVLLNDIAGPDGDSLLLSLKLAVDVLVVSCPCALGLATPTAILVGTSLGARQGLLIRGGDVLERLANVDRIAFDKTGTLTEGKPTVSSVSSFTYDESEILQIAAAVERTATHPIAKAIVKKAELLNLLLPETRGQLVEPGFGTLAEVNGCLVAVGKLEWVNERFQIKASPSDLMALEHAVMRQSSSPSNYSKTAIYVGREGEGVIGAIGMSDSLRFDAESTVSRLQRKGIKTILISGDREEAVATIAKTVGIDHEFVNASLTPQQKSRVISTLQTAGHHIAMVGDGINDAPSLAIADVGIALQTEAQETAASDAASIILLGNRLSQVVDALDLAQATMAKVYQNLSWAVAYNIVAIPIAAGVLLPQYELAMTPSFSGGLMALSSIFVVTNSLLLRLHGSEKSWKNSIAKISQMPAGPG

>GbHMA9

MAMAGDLLRLSVLNRPKLSFSNGAKLKADRFGLLKRCPRGRFHCQPRSTPGFVLFSSLETRLESEESSILPVGQKLKDPSVLLDVNGMMCGGCVSRVKSVISSDERVESVVVNLLTETAAIKLKREVMERETVESVAESIAQRVSECGFMAKMRVSGTGIAENMRKWQEMLKKKEELLVKSRNRVAFAWTLVALCCGAHASHILHSLGIHFGHGSFLEILHNSYVKGGLALTALLGPGRDLLVDGLLAFKKGSPNMNSLVGFGSIVAFIISAVSLLNPGLEWDASFFDEPVMLLGFVLLGRSLEEKARIRASSDMNELLSLISTRSRLVITSSDTDSSADSVLSSDAICIEVPSDDIRVGDSVLVLPGETIPVDGKVLTGRSVVDESMLTGESLPVFKEKGLMVSAGTINWDGPLRIGATSTGSNSTIAKIVRMVEDAQGQEAPVQRLADAIAGPFVYSIMTLSAATFAFWYYAGSHIFPDVLLNDIAGPDGDPLLLSLKLAVDVLVVSCPCALGLATPTAILVGTSLGARQGLLIRGGDVLERLANVDRIAFDKTGTLTEGKPTVSSVSSFTYDESEILQIAAAVERTAIHPIAKAIVKKAELLNLVLPETRGQLVEPGFGTLAEVNGRLVAVGKLEWVNERFQIKAIPSDLMALEHAVMRQSSSPSNYSKTAIYVGREGEGVIGAIGMSDSLRFDAESTVSRLQRKGIKTILISGDREEAVATIAKTVGIEHEFVNASLTPQQKSRVISTLQTAGHHIAMVGDGINDAPSLALADVGIALQTEAQETAASDAASIILLGNRLSQVCSSLTKIVVSCNKLDELVEYKPFSKVVCAFV

>GbHMA8

MAMAGDLLRLSVLNRPKLSFSNGAKLKADRFGLLKRCPRGRFHCQPRSTPGFVLFSSLETRLESEESSILPVGQKLKDPSVLLDVNGMMCGGCVSRVKSVISSDERVESVVVNLLTETAAIKLKREVMERETVESVAESIAQRVSECGFMAKMRVSGTGIAENMRKWQEMLKKKEELLVKSRNRVAFAWTLVALCCGAHASHILHSLGIHFGHGSFLEILHNSYVKGGLALTALLGPGRDLLVDGLLAFKKGSPNMNSLVGFGSIVAFIISAVSLLNPGLEWDASFFDEPVMLLGFVLLGRSLEEKARIRASSDMNELLSLISTRSRLVITSSDTDSSADSVLSSDAICIEVPSDDIRVGDSVLVLPGETIPVDGKVLTGRSVVDESMLTGESLPVFKEKGLMVSAGTINWDGPLRIGATSTGSNSTIAKIVRMVEDAQGQEAPVQRLADAIAGPFVYSIMTLSAATFAFWYYAGSHIFPDVLLNDIAGPDGDPLLLSLKLAVDVLVVSCPCALGLATPTAILVGTSLGARQGLLIRGGDVLERLANVDRIAFDKTGTLTEGKPTVSSVSSFTYDESEILQIAAAVERTAIHPIAKAIVKKAELLNLVLPETRGQLVEPGFGTLAEVNGRLVAVGKLEWVNERFQIKAIPSDLMALEHAVMRQSSSPSNYSKTAIYVGREGEGVIGAIGMSDSLRFDAESTVSRLQRKGIKTILISGDREEAVATIAKTVGIEHEFVNASLTPQQKSRVISTLQTAGHHIAMVGDGINDAPSLALADVGIALQTEAQETAASDAASIILLGNRLSQVVDALDLAQATMAKVYQNLSWAVAYNIVAIPIAAGVLLPQFDLAMTPSFSGGLMALSSIFVVTNSLLLRLHGSEKSWKNSIAKISQMPAGPG

>GbACA32

MSSSDECKLYDCSTSLLTVTAPGGFTVAQRRWRIAYITIYSARVMLSLADKIISQRATQLPSMTSQQFHHYVTEFDHYVALDIEHKINQKRLVKTVKEKDLVSLNHLGGVDGVVDALCTNSEHGIRDDEQEVIKRQEMFGFNKYHKPPPKGLLYFVLEAFKDTTILILLVCATLSLGFGIKEHGTEEGWYEGGSIFVAVFLVIVVSALSNFRQETQFDKLSKISNNIKVEVVRTGRRQQISIFDLVAGDVVFLKIGDQIPADGLFLDGHSLQVDESSMTGESDHVEVDACRNPFLSSGSKVVDGYARMLVASVGMDTAWGEMMSSITSDKNERTPLQARLDKLTSSIGKVGLAVAFLVLAVLLIRYFTGNTKDDNGQTDNDRGRGYPEGLPLAVTLTLAYSMKRMMADQAMVRKLSACETMGSATIICTDKTGTLTLNQMKVTQFWLGQESVEEDLAKEIAPSVLELFYQGVGLNTTGSVCKPVSGSLPEFSGSPTEKAILSWAVLGLGMDMEKLKQQYSILHVETFNSEKKRSGVSVQRKADEMVDIHWKGAAEMIVAMCSQYYESNGTIRSMNEDGRDRIETIIQSMAASSLRCIAFAHKQVLKGETEDGDDQSRKTNRGIKEDGLTLLGIVGLKDPCRPGVKKAVQACQSAGVSIKMITGDNMFTAKAIATECGILGPDYQQGSGEVVEGIEFRNYAPDERMEKVEKIRVMARSSPFDKLLMVQCLKQKGHVVAVTGDGTNDAPALKEADIGLSMGIQGTEVAKESSDIVILDDNFSSVATVLRWGRCVYNNIQKFIQFQLTVNVAALVINFIAAVSAGEVPLTTVQLLWVNLIMDTLGALALATDRPTNELMEKPPVGRTEPLITNIMWRNLLAQALYQIAILLILQFRGESIVNVPETVKDTLIFNTFVLCQVFNEFNARKLEKQNVFEGILKNRLFLGIIGVTIVLQVVMVEFLKKFADTEQLKLWQWGVCILLAAFSWPIAWFVKLIPVSNTPFFSYLKRLRIIYTIVKRPINHQKP

>GbACA30

MTSQQFHHYVTEFDHYVALDIEHKINQKRLVKTVKEKDLVSLNHLGGVDGVVDALCTNSEHGIRDDEQEVIKRQEMFGFNKYHKPPPKGLLYFVLEAFKDTTILILLVCATLSLGFGIKEHGTEEGWYEGGSIFVAVFLVIVVSALSNFRQETQFDKLSKISNNIKVEVVRTGRRQQISIFDLVAGDVVFLKIGDQIPADGLFLDGHSLQVDESSMTGESDHVEVDACRNPFLSSGSKVVDGYARMLVASVGMDTAWGEMMSSITSDKNERTPLQARLDKLTSSIGKVGLAVAFLVLAVLLIRYFTGNTKDDNGQTDNDRGRGYPEGLPLAVTLTLAYSMKRMMADQAMVRKLSACETMGSATIICTDKTGTLTLNQMKVTQFWLGQESVEEDLAKEIAPSVLELFYQGVGLNTTGSVCKPVSGSLPEFSGSPTEKAILSWAVLGLGMDMEKLKQQYSILHVETFNSEKKRSGVSVQRKADEMVDIHWKGAAEMIVAMCSQYYESNGIIRSMSEDGRERIETIIQSMAASSLRCIAFAHKQVLKGETEDGDDQSGKTNRRLKEDGLTLLGIVGLKDPCRPGVKKAVQSCQSAGVGVKMITGDNIFTAKAIATECGILGPDYQQGSGEVVEGIEFRNYAPDERMEKVEKIRVMARSSPFDKLLMVQCLKQKGHVVAVTGDGTNDAPALKEADIGLSMGIQGTE

>GbACA23

MSLRHRNTGYLEPAMSDGEDTPIVKCQHRRWRSVFAAIYSARIFVSLYKKIINKKQILRSLSYIALDVHDSDSSDDRLPFLGVDQKTLTEVVREKSLETLSKLGGVKQIAASLETDKKDGISANEADLAHRVDVFGANRYQKPPKKSFFSFVYEAFKDTTIIILLVCAVLSLGFGIKQHGITDGGYDGGSIVIAVFLVVAVSAVSNFKQNRQFEKLSKESSDIKVEVVRDGRRQFISVFEVVVGDVVCLKIGDQIPADGLFLDGHSLKVDESSMTGESDHVEINGSNNPFVLSGTKVTNGFGSMLVTSVGMNTAWGEMMSSINRELDEETPLQARLNKLTSAIGKIGLAVAVLVLAVLLIRYFTGNTKDDQGNKEYIRGKTKFDSMMNSVVEIISAAITIVVVAIPEGLPLAVTLTLAYSMKQMMAEHAMVRKLSACETMGSATTICTDKTGTLTLNEMKVMEFWLGKELMGSISSEIAPNVHKLLQQAVALNTTGTVYKPNSRSLPEISGSPTEKAILSWAVSDLGMNLDDPKQNYELIQVEAFNSEKKRSGVLIRRKSENGGATQVHWKGAAEMILAMCSQYYDRSGVVKAIDEEERVEMGKVIEDMAAKSLRCIAFAHTQNPKDNERVLQESGLILLGLVGLKDPCRPGVRKAVEACINAGVNIKIITGDNIFTAKAIATECGILQPNEDLREAVIEGVQFRNYSPEERMAKINKICVMARSSPFDKLLMVQCLKQNGHVVAVTGDGTNDAPALKEADIGLSMGIQGTEVAKESSDIIILDDNFTSVVTVLRWGRCVFNNIQKFIQFQLTVNIAALVINFIAAVSSGEIPLTAVQLLWVNLIMDTFGALALATERPTNDLMTKPPVGRSKPLISNIMWRNLIAQALYQVAVLLTLQFRGKFIFDVDKKVKNTLIFNTFVLCQVFNEFNARKLEKKNIFQGLHKNKLFLGIIAITIILQVVMVEFLKRFANTQRLNWGQWGTCIGIAALSWPLGWLVKWIPA

>GbHMA16

DDDGNVVSEVAISTQLIQRNDIIKIIPGEKVPVDGIVIDGQSYVNESMITGEAQPIAKKPGDKVIGGTMNENGCLLVKATHVGSETALSQIVQLVEAAQLARAPIQKIADRISRFFVPAIVLTAFITWLGWLIPGVIGIYPKHWIPKGMDKFELALQFGISVLVVACPCALGLATPTAVMVATGKGASLGVLIKGGNALEKAHKVKAIVFDKTGTLTVGKPEVVNVMLFSSVSMEDFCDVAIAAEANSQHPIAKAFLEHARKLRQKMESNRQSNNQHVTEAKDFEVHPGTGVSGKVGDKMVLVGNKRLMQTYNVTVGPEIEGYISEHEQQARTCVLVSIDGKIAGAFAVTDPVKPEAKNVILYLHSMGISSIMVTGDNWATATAIAKEVGIEKVIAEMDPIGKADRIKDLQMRGLTVAMVGDGINDSPALVAADVGMAIGAGTDVAIEAADIVLIKSNLEDVVTAIDLSRKTISRIWLNYVWALGYNILGVPVAAGILYPFTGVRLPPWLAGACMAASSLSVVCSSLLLQSYRKSWVFQDTKRGHSHCSKST

>GbACA10

MESYLNENFGDVKPKNSSEEALERWRKLCWIVKNRKRRFRFTANLSKRFEAEAIRRSNQEKFRVAVLVSQAALQFIHGLNLSSEYDAPEEVKAAGFQICADELGSIVEGHDVKKLKIHGGVEDIAAKLSTSIVNGIPTSEHLVNERKRIYGINKFTETPPRGFWVFVWEALQDTTLMILAVCALVSLAVGITVEGWPKGAYDGLGIVLSILLVVFVTATSDYRQSLQFRDLDKEKKKITVQVTRDGLRQKISIFDLLPGDIVHLAIGDQVPADGLFISGFSVLINESSLTGESEPVSVNSRNPFLLSGTKVQDGSCKMLVTTVGMRTQWGKLMATLSEGGDDETPLQVKLNGVATIIGKIGLFFAVVTFAVLVQGLFSRKLQDGTQWIWSGDDAMEMLEFFAIAVTIVVVAVPEGLPLAVTLSLAFAMKKMMNDKALVRHLAACETMGSSTSICSDKTGTLTTNHMTVVKTCFCGEIKEVSTSDKNNHFRSAVPESAVKILIESIFNNTGGEVVNNKENKIEILGTPTETALLEFGLLLGGDFQAERKASKIVKVEPFNSAKKRMGVVIEFPEGGLRVHCKGASEIILAACDKVISSNGDVLPLDEPTTNHLKNTIEQFASGALRTLCLAYMDVGTNFSVDSSLPLQGYTCIGIVGIKDPVRPGVKESVAICKSAGITVRMVTGDNINTAKAIAREIGILTDDGIAIEGPVFREKSEEELYELIPKIQVMARSSPMDKHTLVKHLRTSLGEVVAVTGDGTNDAPALHEADIGLAMGIAGTEVAKESADVIILDDNFSTIVTVAKWGRSVYINIQKFVQFQLTVNVVALIVNFASACLTGNAPLTAVQLLWVNMIMDTLGALALATEPPNDDLMKRLPVGRKGNFISNVMWRNILGQSLYQFVIIWILQTRGKAAFHLDGSDSDLILNTLIFNSFVFCQVFNEISSREMEKINVLKGLLKNHVFVAVISCTIIFQIVIVEFLGTFASTSPLTVQQWFVSVCLGFLGMPIAAALKLIPVGSN

>GbACA7

MILAVCALVSLAVGITVEGWPKGAYDGLGIVLSILLVVFVTATSDYRQSLQFRDLDKEKKKITVQVTRDGLRQKISIFDLLPGDIVHLAIGDQVPADGLFISGFSVLINESSLTGESEPVSVNSRNPFLLSGTKVQDGSCKMLVTTVGMRTQWGKLMATLSEGGDDETPLQVKLNGVATIIGKIGLFFAVVTFAVLVQGLFSRKLQDGTQWIWSGDDAMEMLEFFAIAVTIVVVAVPEGLPLAVTLSLAFAMKKMMNDKALVRHLAACETMGSSTSICSDKTGTLTTNHMTVVKTCFCGEIKEVSTSDKNNHFRSAVPESAVKILIESIFNNTGGEVVNNKENKIEILGTPTETALLEFGLLLGGDFQAERKASKIVKVEPFNSAKKRMGVVIEFPEGGLRVHCKGASEIILAACDKVISSNGDVLPLDEPTTNHLKNTIEQFASGALRTLCLAYMDVGTNFSVDSSLPLQGYTCIGIVGIKDPVRPGVKESVAICKSAGITVRMVTGDNINTAKAIAREIGILTDDGIAIEGPVFREKSEEELYELIPKIQVMARSSPMDKHTLVKHLRTSLGEVVAVTGDGTNDAPALHEADIGLAMGIAGTEVAKESADVIILDDNFSTIVTVAKWGRSVYINIQKFVQFQLTVNVVALIVNFASACLTGNAPLTAVQLLWVNMIMDTLGALALATEPPNDDLMKRLPVGRKGNFISNVMWRNILGQSLYQFVIIWILQTRGKAAFHLDGSDSDLILNTLIFNSFVFCQVFNEISSREMEKINVLKGLLKNHVFVAVISCTIIFQIVIVEFLGTFASTSPLTVQQWFVSVCLGFLGMPIAAALKLIPVGSN

>GbACA8

MILAVCALVSLAVGITVEGWPKGAYDGLGIVLSILLVVFVTATSDYRQSLQFRDLDKEKKKITVQVTRDGLRQKISIFDLLPGDIVHLAIGDQVPADGLFISGFSVLINESSLTGESEPVSVNSRNPFLLSGTKVQDGSCKMLVTTVGMRTQWGKLMATLSEGGDDETPLQVKLNGVATIIGKIGLFFAVVTFAVLVQGLFSRKLQDGTQWIWSGDDAMEMLEFFAIAVTIVVVAVPEGLPLAVTLSLAFAMKKMMNDKALVRHLAACETMGSSTSICSDKTGTLTTNHMTVVKTCFCGEIKEVSTSDKNNHFRSAVPESAVKILIESIFNNTGGEVVNNKENKIEILGTPTETALLEFGLLLGGDFQAERKASKIVKVEPFNSAKKRMGVVIEFPEGGLRVHCKGASEIILAACDKVISSNGDVLPLDEPTTNHLKNTIEQFASGALRTLCLAYMDVGTNFSVDSSLPLQGYTCIGIVGIKDPVRPGVKESVAICKSAGITVRMVTGDNINTAKAIAREIGILTDDGIAIEGPVFREKSEEELYELIPKIQVMARSSPMDKHTLVKHLRTSLGEVVAVTGDGTNDAPALHEADIGLAMGIAGTEVAKESADVIILDDNFSTIVTVAKWGRSVYINIQKFVQFQLTVNVVALIVNFASACLTGNAPLTAVQLLWVNMIMDTLGALALATEPPNDDLMKRLPVGRKGNFISNVMWRNILGQSLYQFVIIWILQTRGKAAFHLDGSDSDLILNTLIFNSFVFCQVFNEISSREMEKINVLKGLLKNHVFVAVISCTIIFQIVIVEFLGTFASTSPLTVQQWFVSVCLGFLGMPIAAALKLIPVGSN

>GbACA24

MTIKTEKKNDGINFQEKVPLECCQSGHQCSPKVYSLPSKYQMKLQGTFFVTEREVLTLLSYRFNMSLRHRNTGYPEPAMSDGEDTPIVKCQHRRWRSVFAAIYSARIFVSLYKKIINKKQILRSLSYIALDVHDSDSSDDRLPSLSVDQKTLTEVVREKSLGTLSKLGGVKQIAASLETDEKDGISTNEADLAHRVDVFGANRYQKPPKKSFFSFVYEAFKDTTIIILLVCAVLSLGFGIKQHGITDGGIKVEVVRDGRRQFISVFEVVVGDVVFLKIGDQIPADGLFLDGHSLKVDESSMTGESDHVEINGSNNPFVLSGTKVTNGFGSMLATSVGMNTQWGKMMSSINSELDEETPLQARLNKLTSAIGKIGLAVAVLVIAVLLIRYFTGNTKDDQGNKEYIRGKTKFDSMMNSVVEIISAAITIVVVAIPEGLPLAVTLTLAYSMKQMMADHAMVRKLSACETMGSATTICTDKTGTLTLNEMKVTEFWLGKELMGSISSEIAPNVHKLLQQAVALNTTGTVYKPNSRSLPEISGSPTEKAILSWAVSDLGMNLDDPKQNYELIQVEAFNSEKKRSGVLIRRKSENGGATQVHWKGAAEMILAMCSQYYDRSGAVKAIDEEERVEMRKIIEDMATKSLRCIAFAHTKYPIDNERVLQESGLILLGLVGLKDPCRPGVRTAVEACINAGVNIKMITGDNIFTAKAIATECGILQPNEDMSEAVIEGVQFRNYSPEERMAKINRICVMARSSPFDKLLIVRCLKQNGHVVAVTGDGTNDAPALKEADIGLSMGIQGTEVAKESSDIIILDDNFTSVVTVLRWGRCVFNNIQKFIQFQLTVNIAALVINFIAAISSGEVPLTAVQLLWVNLIMDTFGSLALATERPTNDLMTKPPVGRSKPLISNIMWRNLIAQALYQVAVLLTLQFRGKFIFDVDEKVKNTLIFNTFVLCQVFNEFNARKLEKKNIFQGLHKNKLFLGIIAITIILQVVMVEFLKRFANTQRLNSGQWGTCIGIAALSWPLGWLVKWIPA

>GbHMA17

MATKLLALACIRKESYGDLSPRPHYPSMPKYPKGVTSQETSLQGSEAKAMFSVMGMTCSACAGSVEKAIKRLPGIKEAVVDVLNNKAQVMFYPSFVNGWTESFATALLIVSDAKRCYLMTKRAFVRPFEDAGFQAALIQDETDDKSVQVCRIRINGMTCTSCSSTLENALQAVPGVQKVQVALATEEAQIHHDPKIITYNQFMEKIEEAGFGAVLVSTGEDMSKINLRIDGVRTVNSMRMLENSLPSLPGVQANDIIKIIPGAKVASDGFVLWGQSHINESMITGEARPVAKRKGDTVIGGTVNENGVLHIKATKVGSESALAQIVRLVESAQMAKAPVQKFADRISKYFVPLVIMLSFSTWLAWFLAGKLHGYPESWIPSSMDSFELALQFGISVMVIACPCALGLATPTAVMVGTGVGASLGVLIKDGQALEGAHKVNCIVFDKTGTLTVGKPVVVNTRLLKNMVLHEFYELVAATEVNSEHPLTKAIIEYAKKFREDEENPAWPEARDFVSITGHGVKAIVRNKEVIVGNKSLMLENNIVIPVDAQDMLTETESMAQTGILVSIDGEVKGVLAISDPVKPGAQEVISILKSMNVRSIMVTGDNWRTARDGINDSPALLAADVGMAIGAGTDIAIEAADIVLMKMKPGAQEVISILKSMNVRSIMVTGDNWGTASSIASQIGIETVVAEAKPEQKAEKVKELQAEGYAVAMVGDGINDSPALVAADVGMAIGAGTDIAIEAADIVLMKSNLDDVITAIHLSRKTFSRIRLNYIWALGYNILGIPIAAGALFPSTGFRLPPWIAGAAMAASSVSVVCCSLLLKNYERPKKLENLEIGGIQIE

>GbACA11

MESYLNENFGDVKPKNSSEEALERWRKLCWIVKNRKRRFRFTANLSKRFEAEAIRRSNQEKFRVAVLVSQAALQFIHGLNLSSEYDAPEEVKAAGFQICADELGSIVEGHDVKKLKIHGGVEDIAAKLSTSIVNGIPTSEHLVNKRKRIYGINKFTETPPRGFWVFVWEALQDTTLMILAVCALVSLAVGITVEGWPKGAYDGLGIVLSILLVVFVTATSDYRQSLQFRDLDKEKKKITVQVTRDGLRQKISIFDLLPGDIVHLAIGDQVPADGLFISGFSVLINESSLTGESEPVSANARNPFLLSGTKVQDGSCKMLVTTVGMRTQWGKLMATLSEGGDDETPLQVKLNGVATIIGKIGLFFAVVTFAVLVQGLFSRKLQDGTQWIWSGDDAMEMLEFFAIAVTIVVVAVPEGLPLAVTLSLAFAMKKMMNDKALVRHLAACETVGSSTSICSDKTGTLTTNHMTVVKTCFCGETKEVSTSNKSNHFRSAVPESAVKILIESIFNNTGGEVVNNKENKIEILGTPTETALLEFGLLLGGDFQAERKASKIVKVEPFNSAKKRMGVVIEFPEGGLRVHCKGASEIILAACDKVISSNGDVVPLDEPTTNHLKNTIEQFASEALRTLCLAYMDVGTDFSVDSSLPLQGYTCIGIVGIKDPVRPGVKESVAICKSAGITVRMVTGDNINTAKAIAREIGILTDDGIAIEGPVFREKSEEELYELIPKIQVMARSSPMDKHTLVKHLRTSLGEVVAVTGDGTNDAPALHEADIGLAMGIAGTEVAKESADVIILDDNFSTIVTVAKWGRSVYINIQKFVQFQLTVNVVALIVNFTSACLTGNAPLTAVQLLWVNMIMDTLGALALATEPPNDDLMKRSPVGRKGNFISNVMWRNILGQSLYQFVIIWFLQTRGKAAFHLDGPDSDLILNTLIFNSFVFCQVFNEISSREMEKINVLKGILKNHVFVAVLSCTIIFQIVIVEFLGTFASTSPLTVQQWFVSVCLGFLGMPIAAALKLIPVGSN

>GbACA9

MILAVCALVSLAVGITVEGWPKGAYDGLGIVLSILLVVFVTATSDYRQSLQFRDLDKEKKKITVQVTRDGLRQKISIFDLLPGDIVHLAIGDQVPADGLFISGFSVLINESSLTGESEPVSANARNPFLLSGTKVQDGSCKMLVTTVGMRTQWGKLMATLSEGGDDETPLQVKLNGVATIIGKIGLFFAVVTFAVLVQGLFSRKLQDGTQWIWSGDDAMEMLEFFAIAVTIVVVAVPEGLPLAVTLSLAFAMKKMMNDKALVRHLAACETVGSSTSICSDKTGTLTTNHMTVVKTCFCGETKEVSTSNKSNHFRSAVPESAVKILIESIFNNTGGEVVNNKENKIEILGTPTETALLEFGLLLGGDFQAERKASKIVKVEPFNSAKKRMGVVIEFPEGGLRVHCKGASEIILAACDKVISSNGDVVPLDEPTTNHLKNTIEQFASEALRTLCLAYMDVGTDFSVDSSLPLQGYTCIGIVGIKDPVRPGVKESVAICKSAGITVRMVTGDNINTAKAIAREIGILTDDGIAIEGPVFREKSEEELYELIPKIQVMARSSPMDKHTLVKHLRTSLGEVVAVTGDGTNDAPALHEADIGLAMGIAGTEVAKESADVIILDDNFSTIVTVAKWGRSVYINIQKFVQFQLTVNVVALIVNFTSACLTGNAPLTAVQLLWVNMIMDTLGALALATEPPNDDLMKRSPVGRKGNFISNVMWRNILGQSLYQFVIIWFLQTRGKAAFHLDGPDSDLILNTLIFNSFVFCQVFNEISSREMEKINVLKGILKNHVFVAVLSCTIIFQIVIVEFLGTFASTSPLTVQQWFVSVCLGFLGMPIAAALKLIPVGSN

>GbACA6

MDSYLNQNFDLKSKHSSDEALEKWRTVVGFVKNPKRRFRFTANLSKRYEAAAMRRTNQEKLRIAVLVSKAAFQFISGVQPSDYVVPEQVTAAGFQLCADELGSIVEGHDVKKLKFHGGVSGIAEKLSTSTNTGLSSDAALLSKRQEIYGINKFAEPEAKGFWVFVWEALQDMTLMILGACAIVSLVVGIAMEGWPAGAHDGLGIVASILLVVFVTATSDYRQSLQFKDLDKEKKKITIQVTRNDCRQKLSIYDLLPGDIVHLNIGDQVPADGLFVSGFSVLIDESSLTGESEPVVVNDKNPFMLSGTKLQDGSCKMLVTTVGMRTQWGKLMATLSEGGDDETPLQVKLNGVATIIGKVGLFFAVVTFAVMVQGLLRSKLQEGTIWSWSGDEALKLLEFFAVAVTIVVVAVPEGLPLAVTLSLAFAMKKMMNDKALVRHLAACETMGSATGICSDKTGTLTTNRMTVVKSCICMSVKEINSTNKASFCSEIHESAIKLLLQSIFMNTGGEIVTSKDGRREILGTPTETALLEFGLSLGGDPQTERQASKTVKVEPFNSTKKRMGVVLELPGGGLRAHTKGVQPSDYVVPEQVTAAGFQLCADELGSIVEGHDVKKLKFHGGVSGIAEKLSTSTNTGLSSDAALLSKRQEIYGINKFAEPEAKGFWVFVWEALQDMTLMILGACAIVSLVVGIAMEGWPAGAHDGLGIVASILLVVFVTATSDYRQSLQFKDLDKEKKKITIQVTRNACRQKMSIYDLLPGDIVHLNIGDQVPADGLFVSGFSVLIDESSLTGESEPVVVNDKNPFMLSGTKLQDGSCKMMVTSVGMRTQWGKLMATLSEGGDDETPLQVKLNGVATIIGKVGLFFAVVTFAVMVQGLLRSKLQEGTIWSWSGDEALKLLEFFAVAVTIVVVAVPEGLPLAVTLSLAFAMKKMMNDKALVRHLAACETMGSATGICSDKTGTLTTNRMTVVKSCICMNVKEINSTNKASFCSEIHESAIKLLLQSIFMNTGGEIVTSKDGRREILGTPTETALLEFGLSLGGDPQTERQASKTVKVEPFNSTKKRMGVVLELPGGGLRAHTKGASEIVLAGCDKVIDSNGEVVPLDEKSINHLNATIIEFANEALRTLCLAYLELKNGFSSDNAIPVSGYTCLGIVGIKDPVRPGVKESVAICRTAGITVRMVTGDNINTAKAIARECGILTDDGIAIEGPDFREKSQEELLKLIPKIQVIARSSPMDKHTLVKQLRTTFDEVVAVTGDGTNDAPALHEADIGLAMGIAGTEVAKESADVIILDDNFSTILTVAKWGRSVYINIQKFVQFQLTVNIVALIVNFSSACLTGSAPLTAVQLLWVNMIMDTLGALALATEPPTDELMKRAPVGRKGNFISNVMWRNIFGQSFYQLMVIWYLQARGKAMFELDGPDSTLKLNTLIFNSFVFCQVFNEISSRNMEEIDVLSGILNNSVFVAVLGCTAVFQIIIIEFLGTFASTTPLTYSQWGLSVVIGFFSMPIAAALKLVPV

>GbACA27

MTTIFEPNLICSEHTIQVPPSLSTSDKAWHSIFASKKFSSLLTKSPTPKEEAKVVRRSPSHVSVNVMQENPLYQIDQRTLIELVKEKKLDRLQEFGGVNGVASNFGTDTQVGISGGADDVARLRNTFGSNTYNKPPTKGFFHFVIEALKEPAVMILLGCAALSLGFKIKAHGHKDSWYEVGSNFAAVFLPIVFSAISNYMQNRQFKELSRTNNKILVDVVRGGQRQQIRMFDVVVGDIVCLKMGDQVPADGLFLDGHSLQVDESSMTGETEHTEVNSSQNPFLLSWTKVAHGDARMLVTSVGSNTTWGQISRETNEQTPLQARLNKLSLSIAKVGLAVAFPVLLVLLTSDIINAIVGIATTAISTVAEGLPLVVTLTLAYSMKKIVADQAMVRKLSACETMGSTTTICTNKTGTLTLNRMEVTKFWLGQESMEEGASSISPFVVDLIHQGVALNTTGSFYRASPGTEYEFSGSPTEKAILSWAVVELKMDVEKTKKSCAVLQVEAFISQRRRSGVLIERNDDDTVHVHWKGAAEMILAMCSSYNDASGVVKDLDDGERTKFEEIIQGMAASSLRCIAFAHKQVPEEDYYNASGVEYQNLKEQKKLKEDSLALLGLVGIKDPFRPGVKKAVEDCQYAGVNIKMITGDNVFTARAIATECGILKPGQDLSSGAVVEVEKIQVMARSSPFDKLLMVQCLKQKGHVVAFTGDGTDDAPALKEADIGLSMGIQGTEAAKESSDIVILDDSFASVATVLRWGRCVYTNIQKFIQFLLTVNVSALCINFIAAVSTGEVPLTTVQLLWVNLYMDTLGALALATELPTKELLEKPPVGRTEPLITNIMWRNLLAQALYQIAVFNNFNARKLEKKNIFDDIHKNKMFIGIIGVTIVLQVVMVEFLKRFTDTESHWGWHKCCTTTPAHREADICLFMGIQGTEVAKESSNIVILEDDFASVATVLRWGEDERMEKVEKIQVMARSTSSDKLLMVQGLKQKGQVVAVTGDGTNDAPALKEADIGLSMGIQGTEVAKESSDIIILDDNFASVTMVLWWGKCVYTNIQKFIQFLLTVNVSALCINFIAAVSTGEVPLTTVQLLWGNLYMNTLAALSLVTERPTKELMAKPPISRTELLITNIMWRKVVSSSIPNSTLQFNGESILRVTH

>GbACA33

MSSSDECKLYDCSTSLLTVTAPGGFTVAQRRWRIAYITIYSARVMLSLADKIISQRATQLPSMTSQQFHHYVTEFDHYVALDIEHKINQKRLVKTVKEKDLVSLNHLGGVDGVVDALCTNSEHGIRDDEQEVIKRQEMFGFNKYHKPPPKGLLYFVLEAFKDTTILILLVCATLSLGFGIKEHGAEEGWYEGGSIFVAVFLVIVVSALSNFRQETQFDKLSKISNNIKVEVVRSGRRQQISIFDLVAGDVVFLKIGDQIPADGLFLDGHSLQVDESSMTGESDHVEVDACRNPFLSSGSKVVDGYARMLVASVGMDTAWGEMMSSITSDKNERTPLQARLDKLTSSIGKVGLAVAFLVLAVLLIRYFTGNTKDDNGQTEYRGSETDVDDILNAVVRIVAAAVTIVVVAIQKMKVTQFWLGQESVEEDLAKKIAPSVLELFYQGVGLNTTGSVCKPVSGSLPEFSGSPTEKAILSWAVLGLGMDMEKLKQQYSILHVETFNSEKKRSGVSVQKKADEMVHIHWKGAAEMIVAMCSQYYESNGTIRSMNEDGRDRIETIIQSMAASSLRCIAFAHKQVLKGETEDGDDQSRKTNRGIKEDGLTLLGIVGLKDPCRPGVKKAVQACQSAGVSIKMITGDNMFTAKAIATECGILGPDYQQGSGEVVEGIEFRNYAPDERMEKVEKIRVMARSSPFDKLLMVQCLKQKGHVVAVTGDGTNDAPALKEADIGLSMGIQGTEVAKESSDIVILDDNFSSVATVLRWGRCVYNNIQKFIQFQLTVNVAALVINFIAAVSAGEVPLTTVQLLWVNLIMDTLGALALATDRPTNELMEKPPVGRTEPLITNIMWRNLLAQALYQIAILLILQFRGESIVNVPETVKDTLIFNTFVLCQVFNEFNARKLEKQNVFEGILKNRLFLGIIGVTIVLQVVMVEFLKKFADTEQLKLWQWGVCILLAAFSWPIAWFVKLIPVSNTPFFSYLKRLRIIYTIVKRPINHQKP

>GbACA4

METFWNESFDLKPKHSSEEALEKWRKVVGFVKNPKRRFRFTANLSKRYEAAAMRRSNHEKLRIAVLVSKAALQFISGVKPSESDYVVPEEVKAAGFELCAEELGSIVEGQDVKKLKIHGGVDGIAEKLSTSTTDGLSSDSVLLNKRQEVYGINKFAEAEAKGFLVFVWEALQDMTLMILGVCALVSLIVGIAMEGWPKGAHDGLGIVASILLVVFVTATSDYRQSLQFKDLDKEKKKITIQVTRNACRQKMSIYDLLPGDIVHLNIGDQVPADGLFVSGFSVLIDGSSLTGESEPVMVNADNPFMLSGTKLQDGSCKMMVTSVGMRTQWGKLMATLSEGGDDETPLQVKLNGVATIIGKVGLFFAVVTFAVMVQGLFMSKLQEGTIWSWSGDEALKLLEFFAVAVTIVVVAVPEGLPLAVTLSLAFAMKKMMNDKALVRHLAACETMGSATNICSDKTGTLTTNHMTVVKSCICMDVREVGNNNKASLCSEIPESAVKLLLQSIFTNTGGEIVINKDGKREILGTPTETALLEFGLSLGGDSRAERLASKLVKVEPFNSTKKRMGVILELPEGGLRAHTKGASEIVLAGCDKVINSNGEVVPLDVESINHLNATINQFANEALRTLCLAYMELENDFSPDNAIPLSGYTCIGIVGIKDPVRPGVKESVAICRSAGITVRMVTGDNINTAKAIARECGILTDDGIAIEGPDFREKSQEEMLALIPKIQVMARSSPMDKHTLVRQLRSIDEVIAVTGDGTNDAPALHEADIGLAMGISGTEVAKESADVIILDDNFSTIVTVAKWGRSVYINIQKFVQFQLTVNIVALIVNFSSACLTGTAPLTAVQLLWVNMIMDTLGALALATEPPTDELMKRAPVGKKGNFISNVMWRNILGQSFYQFMVIWYLQVKGKGMFSLEGPDSNLTLNTIIFNSFVFCQVFNEISSRNMEEINVFKGILNNYVFVAVLGCTAVFQVIIIEFLGTFASTTPLTCLQWFVSVFIGFLGMPVAAALKTIPV

>GbACA16

MMGDSTLTSDKFCLNLVRILTWGHKLKSQVQAALVLNASRRFRYTLDLRKQEEKEQRKRMIRAHAQVIRAALLFKLAGEKEIVSGTPVTLPGAAGDFAVGLEQLASMTRDHKLSALQQYGGVKGLSDLLKTNLETGIYGDEVDLLNRKTAFGSNTYPRKKGRSFWQAALVLNASRRFRYTLDLRKQEEKEQRKRMIRAHAQVIRAALLFKLAGEKEIVSGTPVTLPGAAGDFAVGLEQLASMTRDHKLSALQQYGGVKGLSDLLKTNLETGIYGDEVDLLNRKTAFGSNTYPRKKGRSFWRFLWEAWQDLTLIILIVAASVSLGLGIKTEGLKEGWYDGGSIFLAVLLVIVVTATSDYRQSLQFQNLNEEKRNIQLEVLRGGRTVKVSIYDVVVGDVVPLKIGDQVPADGVLVSGHSLAIDESSMTGESKIVHKDKKEPFLMSGCKVADGVGTMLVTGVGINTEWGLLMASISEDTGEETPLQVRLNGVATFIGIVGLSVAVSVLVVLLARYFTGHTEDPDGTKQFIKGRTNFDDAFNDVVKIFTIAVTIVVVAVPEGLPLAVTLTLAYSMRKMMADKALVRRLSACETMGSATTICSDKTGTLTLNEMTVVEAFVGKKKINPPSDSSQLPASVVSLLNEGVAQNSTGNVFVPKDGGNIEISGSPTEKAILSWAVKLGMKFDIIRSDSKILHVFPFNSEKKRGGVALQGADSEVRIHWKGAAEIVLTSCSGYIDSNGCLQSINEDKEFFKAAIDEMAVNSLRCVALAYRLCEKEKVPTDEEGFNGWILPEDNLILLAIVGIKDPCRPGVKDAVKICMDAGVKVRMVTGDNIQTAKAIALECGILSSAEDATEPTIIEGRVFRELSDKEREQIAKKITVMGRSSPSDKLLLVQALRKGGDVVAVTGDGTNDAPALHEADIGLSMGIQGTEVAKESSDIIILDDNFASVVKVVRWGRSVYANIQKFIQFQLTVNVAALVINVVAAISSGDVPLNSVQLLWVNLIMDTLGALALATEPPTDNLMHRSPVGRREPLITNIMWRNLLLQASYQVTVLLVLNFAGLTILHLKDDRDREHAYDVKNTLIFNAFVMCQIFNEFNARKPEEINCFKGVTKNYLFMGIIGFTFVLQIIIIEFLGKFTTTVRLNWKLWLVSLGIGIISWPLAIVGKLIPVPKTPVSGYFTKAFRRCRTDRNA

>GbACA5

MGTFWNENFDLKPKHSSEEALEKWRKVVGFVKNPKRRFRFTANLSKRYEAAAMRRSNHEKLRIAVLVSKAALQFISGVKPSESDYVVPEEVKAAGFELCAEELGSIVENQDVKKLKIHGGVDGIAEKLSTSTTDGLSSDSGLLNKRQEVYGINKFAEAEAKGFLVFVWEALQDMTLMILGVCALVSLIVGIAMEGWPKGAHDGLGIVASILLVVFVTATSDYRQSLQFKDLDKEKKKITIQVTRNACRQKMSIYDLLPGDIVHLNIGDQVPADGLFVSGFSVLIDGSSLTGESEPVIVNVDNPFMLSGTKLQDGSCKMMVTSVGMRTQWGKLMATLSEGGDDETPLQVKLNGVATIIGKVGLFFAVVTFAVMVQGLFTSKLQEGTIWSWSGDEALKLLEFFAVAVTIVVVAVPEGLPLAVTLSLAFAMKKMMNDKALVRHLAACETMGSATNICSDKTGTLTTNHMTVVKSCICMDVREVGNNNKASLCSEIPESAVKLLLQSIFTNTGGEIVINKDGKREILGTPTETALLEFGLSLGGDSQAERLASKLVKVEPFNSTKKRMGVVLELPEGGLRAHTKGASEIVLAGCDKVINCNGEVIPLDAESINHLNATINQFANEALRTLCLAYMELENGFSPDNAIPVSGYTCIGIVGIKDPVRPGVKESVAICRAAGITVRMVTGDNINTAKAIARECGILTDDGIAIEGPDFREKSQEEMLALIPKIQVMARSSPMDKHTLVRQLRSIDEVVAVTGDGTNDAPALHEADIGLAMGISGTEVAKESADVIILDDNFSTIVTVAKWGRSVYINIQKFVQFQLTVNIVALIVNFSSACLTGTAPLTAVQLLWVNMIMDTLGALALATEPPTDELMKRAPVGKKGNFISNVMWRNILGQSFYQFMVIWYLQVKGKGMFSLDGPDSDLKLNTIIFNSFVFCQVFNEISSRNMEEINVFKGILNNYVFVAVLGCTAVFQVIIIEFLGTFASTTPLTCLQWFVSVVIGFLGMPIAAALKTIPV

>GbACA17

MLLQAALLFKLAGEKGIGTPVTLPGAAGDFAVGLEQLASMTRDHKLSALQQYGGVKGLSDLLKTNLETGIYGDEVDLLNRKTAFGSNTYPRKKGRSFWQAALVLNASRRFRYTLDLRKQEEKEQRKRMIRAHAQVIRAALLFKLAGEKGIVSGTPVTLPGAAGDFAVGLEQLASMTRDHKLSALQQYGGVKGLSDLLKTNLETGIYGDEVDLLNRKTAFGSNTYPRKKGRSFWRFLWEAWQDLTLIILIVAASVSLGLGIKTEGLKEGWYDGGSIFLAVLLVIVVTATSDYRQSLQFQNLNEEKRNIQLEVVRGGRIVKVSIYDVVVGDVVPLKIGDQVPADGVLVNGHSLAIDESSMTGESKIVYKDKNEPFLMSGCKVADGVGTMLVTGVGINTEWGLLMASISEDTGEETPLQVRLNGVATFIGIVGLSVAVSVLVVLLARYFTGHTEDPDGTKQFIKGRTNFDDAFNDVVKIFTIAVTIVVVAVPEGLPLAVTLTLAYSMRKMMADKALVRRLSACETMGSATTICSDKTGTLTLNEMTVVEAFVGKKKINPPSDSSQLPASVVSLLNEGVAQNSTGSVFVPKDGGNIEISGSPTEKAILSWAVKLGMKFDIIRSDSKILHVFPFNSEKKRGGVALQGADSEVRIHWKGAAEIVLTSCSGYIDSNSCLQSIKEDKEFFKAAIDEMAVNSLRCVALAYRLCEKEKVPTDEEGFNGWILPEENLVLLAIVGIKDPCRPGVKDAVKICMDAGVKVRMVTGDNIQTAKAIALECGILSSAEDAAEPTIIEGRVFRELSDKEREQISKKITVMGRSSPSDKLLLVQALRKGGDVVAVTGDGTNDAPALHEADIGLSMGIQGTEVAKESSDIIILDDNFASVVKVVRWGRSVYANIQKFIQFQLTVNVAALVINVVAAISSGDVPLNSVQLLWVNLIMDTLGALALATEPPTDNLMHRSPVGRREPLITNIMWRNLLLQASYQVTVLLVLNFMGLTILHLKDDHDREHAYDVKNTLIFNAFVMCQIFNEFNARKPEEINCFKGVTKNYLFMGIIGFTFVLQIIIIEFLGKFTKTVRLNWKLWLVSLGIGIISWPLAIVGKLIPVPKTPVSGYFTKAFRRCRTDGNA

>GbHMA5

METHSISLTNFSPLTRPLRPSRLRRLNSFHFKPLFFSPLSTRYKSLFLPLNSHTIRIRCVANHGHHHHHEHDLDHGHDHDHDHHHHHHHHHHGSGQLNGPQKAVIGFAKAIRWMDLANFLREHLHLCCCATALFIAAAAFPYLVPKPAVKPLQNSFLVLAFPLVGVSAALDAITDIAGGKVNIHVLMALAAFASVFMGNALEGGLLLAMFNLAHIAEEFFTSRSMIDVKELKENYPDSALVLNLDDDNLPNVSDLSYRSIPVHDVEVGSYILVTTGEAVPVDCEVFHGSATITIEHLTGEIKPLEAKAGDRIPGGARNLDGRMIVKVLKTWKESTLSRIVQLTEEAQLNKPKLQRWLDEFGEQYSKVVVVLSVAIAVLGPFLFKWPFISTAVCRGSIYRALGLMVAASPCALAVAPLAYATAVSSCARKGILLKGGQVLDALASCHTVAFDKTGTLTTGGLMFKAIEPIYGHIIGNKKTNFTSCCVPNCEVEALAVAAAMEKGTTHPIGRAVVDHSIGKDLPSVSVESFEYFPGKGLIATLNSAESGTRGGKMLKASLGSIEFITSLCKSEVESRKIRAAVNASSYGTDFVHAALSVDEKVTLIHLEDRPRPGVLDVISELKDKAKVRVMMLTGDHKLSAWRVANAVGINEVYCSLKPEDKLNHVKRISGDMGGGLIMVGEGINDAPALAAATVGIVLAHRASATAIAVADVLLLQDNISGVPFSIAKARQTTSLVKQNVALALTCIILASLPSVLGFLPLWLTVLLHEGGTLLVCLNSIRALNDPSWSWGQDLRNLIGKLKSKLAFLRHNATSSTIQTAPL

>GbACA19

MSSIFKGSPYRRPNDLEAGSSRSAHSDDEDHESCADPFDITSTKNAPIDRLRRWRQAALVLNASRRFRYTLDLKKEEEKKQILRKIRAHAQAIRAAYLFKQAGEQVNGTTTPHPTPGSDFAFGPEQLASVTRDHNFNALQEYGGVNGLAESLKTNLQKGIPGDDSDLLKRRNAFGSNTYPRKKGRSFWRFVWEACQDLTLMILVVAAVASLALGIKTEGPKEGWYDGGSIAFAVFLVIIVTAISDYRQSLQFQKLDEEKRNIHLEVVRGGRRVEISIYDIVVGDIIPLNIGDQVPADGILISGHSLAIDESSMTGESDIVQKDAKQPFLMSGCKVADGSGTMLVTGVGINTEWGLLMANLSEDTGEETPLQVRLNGVATFIGFVGLSVAFAVLVVLLVRYFTGHTEDSNGRQQFVAGKTSVGNAIDGAIKIVTVAVTIVVVAVPEGLPLAVTLTLAYSMKKMMADKALVRRLSACETMGSATTICSDKTGTLTLNQMTVVEAYAGGRKNDPPESRSELPDTLVSLLIEGIAVNANGSVFTPEGGGDVEVSGSPTEKAILNWGIKLGMDFDAVRSGSSIVHVFPFNSEKKRGGVAIRLPDSKVHIHWKGAAEIVLAACTRYLDTNGEAVAMDEEKVAFFEKAIETMAAGSLRCVAIAYRSYESEKVPTNEEELAKWALPEDDLVLLAVVGIKDPCRPSVKKSVQLCQKAGVKVRMVTGDNLKTARAIALECGILSSDAPESSLIEGKVFRSLSDSEREEVAEKISVMGRSSPNDKLLLVQALRRKGHVVAVTGDGTNDAPALHEADIGLAMGIQGTEVAKESSDIIILDDNFASVVKVVRWGRSVYANIQKFIQFQLTVNVAALVINVVAAVSSGDVPLNAVQLLWVNLIMDTLGALALATEPPTDHLMHRPPVGRREPLITNIMWRNLLIQAIYQVTVLLVLNFDGKKILHLEHESKEHAKRVKNTLIFNAFVLCQIFNEFNARKPEEVNIFRGLSKNYLFIGIVAITIILQAIIVEFLGKFAKTEKLSWQLWLVSIGIGFISWPLAILGKFIPVPETPAIYQVTVLLVLNFDGKKILHLEHESKEHAKRVKNTLIFNAFVLCQIFNEFNARKPEEVNIFRGLSKNYLFIGIVAITIILQAIIVEFLGKFAKTEKLSWQLWLVSIGIGFISWPLAILGKFIPVPETPVSRVFSRMFYRRRNQSSQKHEDSNATSIRN

>GbACA20

MSSIFKGSPYRRPNDLEAGSSRSAHSDDEDHESFADPFDITSTKNAPIDRLRRWRQAALVLNASRRFRYTLDLKKEEEKKQILRKIRAHAQAIRAAYLFKQAGEQVNGTTTPHPTPGSDFAFGPEQLASVTRDHNFNALQEYGGVNGLAESLKTNLEKGIPGDDSDLLKRRNAFGSNTYPRKKGRSFWRFVWEACQDLTLMILVVAAVASLALGIKTEGPKEGWYDGGSIAFAVFLVIIVTAISDYRQSLQFQKLDEEKRNIHLEVVRGGRRVEISIYDIVVGDVVPLNIGDQVPADGILISGHSLAIDESSMTGESDIVQKDAKQPFLMSGCKVADGSGTMLVTGVGINTEWGLLMANLSEDTGEETPLQVRLNGVATFIGFVGLSVAFAVLVVLLVRYFTGHTEDSNGRQQFVAGKTSVGNAIDGAIKIVTVAVTIVVVAVPEGLPLAVTLTLAYSMKKMMADKALVRRLSACETMGSATTICSDKTGTLTLNQMTVVEAYAGGRKNDPPESRSELTDTLVSLLIEGIAVNANGSVFTSEGGGDVEVSGSPTEKAILIWGIKLGMDFDAVRSGSSIVHVFPFNSEKKRGGVAIRLPDSKVHIHWKGAAEIVLAACTWYLDTNGEAVAMDEEKVAFFEKAIETMAAGSLRCVAIAYRSYESEKVPTNEEELAKWALPEDDLVLLAIVGIKDPCRPSVKDSVQLCQKAGVKVRMVTGDNLKTARAIALECGILSSDAPESSLIEGKVFRSLSASEREEVAEKISVMGRSSPNDKLLLVQALRRKGHVVAVTGDGTNDAPALHEADIGLAMGIQGTEVAKESSDIIILDDNFASVVKVVRWGRSVYANIQKFIQFQLTVNVAALVINVVAAVSSGDVPLNAVQLLWVNLIMDTLGALALATEPPTDHLMHRPPVGRREPLITNIMWRNLLIQAIYQVTVLLVLNFDGKKILNLEHESKEHANRVKNTLIFNAFVLSQIFNEFNARKPDEVNIFRGLSKNYLFIGIVAITIILQADIGLAMGIQGTEVAKESSDIIILDDNFASVVKVVRWGRSVYANIQKFIQFQLTVNVAALVINVVAAVSSGDVPLNAVQLLWVNLIMDTLGALALATEPPTDHLMHRPPVGRREPLITNIMWRNLLIQAIYQVTVLLVLNFDGKKILNLEHESKEHAKRVKNTLIFNAFVLCQIFNEFNARKPEEVNIFRGLSKNYLFIGIVAITIILQAIIVEFLGKFAKTEKLSWQLWLVSIGIGFISWPLATLGKFIPVPETPVSRVFSRMFYRRRNQSSQKHEDSNATSIRN

>GbAHA22

MERFKFLRFFSFRTQLDFLPCSNPVRENLINGATTDTNGEHRDGFFSNLVDGFLRRFKSGKKIDGSSKTEEEEKVYSWLYALARSEIYMAFEYVRSTERGLSFTEAEKRLKENGPNTPLEYTFPSSWHLLWSAFFHPFNIILIVLSALSYITSDNPNGCIMLILVFISVSLRFYQEYGSSKAAMQLSEYVRSPVKVQRCAGRVVQTELIVQVDQRDVVPGDIVIFEPGDLFPGDVRLLTSKHLVVSQSSLTGESWPTEKTADAREDRSTPLLELKNICFMGTNVVSGTGTGLVVSTGSKTYISAVFSTIGKHKPADSFEKGIRHISYVLVGVMLLVVTIMILVEYFTFYDLSESTLFGISVACALTPQMLPLIINTSLAKGALAMARERCIVKSLSAIRDMGSIFTEAEKRLKENGPNTPLEYTFPSSWHLLWSAFFHPFNIILIVLSALSYITSDNPNGCIMLILVFISVSLRFYQEYGSSKAAMQLSEYVRSPVKVQRCAGRVVQTELIVQVDQRDVVPGDIVIFEPGDLFPGDVRLLTSKHLVVSQSSLTGESWPTEKTADAREDRSTPLLELKNICFMGTNVVSGTGTGLVVSTGSKTYISAVFSTIGKHKPADSFEKGIRHISYVLVGVMLLVVTIMILVEYFTFYDLSESTLFGISVACALTPQMLPLIINTSLAKGALAMAMERCIVKSLSAIRDMGSMDILCIDKTGTLTMNRAIMVNHLDSWGAPREKVLHFAFLNSYFKSDQKYPLDDAILAFVYTNGYRFQPSKWRKTDEIPFDFMRRRVSVILETESNPEVRNIQPFYRFIITKGALEDVLKVCSYVENVDMGEITAVSAEHHHRILNVVEELRNEGLRIIGVAIKNLQQTKISKQNMENEDDVESDMVFLGVIAFFDPPKDSAKEALWRLAEKGVKAKVLTGDSLSLAIKICKEVGIRTTHVTTGPELEQLNQEAFHEKVKKATVLARLTPSQKLRVVQSLQSVGNHVVGFLGDGINDSLALDAANVGISVDSAASVAKDLADIILLEKDLNVLVAGVEQGRLTFGNTMKYIKLSVIANLGSVISLFIVTLALRFEPLTPIQLLVQNFLYSVGQIAIPWDRMEEDYVKIPQKWSVKSLPMFILWNGPVCTLCDVATLLFVWFYYSSGGKLDESFFRSAYFIQGLLMQTLIFHLIRTEKIPFIMEIASWPVLASTVVISAIGIAIPFSIIGHFMGFTDLPLSYFGFLVVLFVGYFTVGQIVKRVYIMVYKKWL

>GbAHA23

MERFKFLRFFSFRTQHDFLPSSNPVRENLINGANTDTNGEHRDGFFSNLVYGFLRRFKSGKKIDGGSKTEEEEKVYSWLYALARSEIYMAFEYVRSTERGLSFTEAEKRLKENGPNTPLEYTFPSSWHLLWSAFFHPFNIILIVLSALSYITSDNPNGCIMLILVFISVSLRFYQEYGSSKAAMQLSEYVRSPVKVQRCAGRVVQTELIVQVDQRDVVPGDIVIFEPGDLFPGDVRLLTSKHLVVSQSSLTGESWPTEKTADAREDRSTPLLELKNICFMGTNVVSGTGTGLVVSTGSKTYISAVFSTIGKHKPADSFEKGIRHISYVLVGVMLLVVTIMILVEYFTFYDLSESTLFGISVACALTPQMLPLIINTSLAKGALAMARERCIVKSLSAIRDMGSITQHDFLPSSNPVRENLINGANTDTNGEHRDGFFSNLVDGFLRRFKSGKKIDGGSKTEEEEKVYSWLYALARSEIYMAFEYVRSTERGLSFTEAEKRLKENGPNTPLEYTFPSSWHLLWSAFFHPFNIILIVLSALSYITSDNPNGCIMLILVFISVSLRFYQEYGSSKAAMQLSEYVRSPVKVQRCAGRVVQTELIVQVDQRDVVPGDIVIFEPGDLFPGDVRLLTSKHLVVSQSSLTGESWPTEKTADAREDRSTPLLELKNICFMGTNVVSGTGTGLVVSTGSKTYISAVFSTIGKHKPADSFEKGIRHISYVLVGVMLLVVTIMILVEYFTFYDLSESTLFGISVACALTPQMLPLIINTSLAKGALAMARERCIVKSLSAIRDMGSIFTEAEKRLKENGPNTPLEYTFPSSWHLLWSAFFHPFNIILIVLSALSYITSDNPNGCIMLILVFISVSLRFYQEYGSSKAAMQLSEYVRSPVKVQRCAGRVVQTELIVQVDQRDVVPGDIVIFEPGDLFPGDVRLLTSKHLVVSQSSLTGESWPTEKTADAREDRSTPLLELKNICFMGTNVVSGTGTGLVVSTGSKTYISAVFSTIGKHKPADSFEKGIQHISYVLVGVMLLVVTIMILVEYFTFYDLSESTLFGISVACALTPQMLPLIINTSLAKGALAMAMERCIVKSLSAIRDMGSMDILCIDKTGTLTMNRAIMVNHLDSWGAPREKVLHFAFLNSYFKSDQKYPLDDAILAFVYTNGYRFQPSKWRKTDEIPFDFMRRRVSVILETESNPEGRNIQPFYRFIITKGALEDVLKVCSYVENVDMGEITAVSAEHHHRILNVVEELRNEGLRIIGVAIKNLQQTKISKQNMENEDDVESDMVFLGVIAFFDPPKDSAKEALWRLAEKGVKAKVLTGDSLSLAIKICKEVGIRTTHVTTGPELEQLNQEAFHEKVKKATVLARLTPSQKLRVVQSLQSVGNHVVGFLGDGINDSLALDAANVGISVDSAASVAKDLADIILLEKDLNVLVAGVEQGRLTFGNTMKYIKLSVIANLGSVVSLFIVTLVLRFEPLTPIQLLVQNFLYSVGQIAIPWDRMEEDYVKIPQKWSVKSLPMFILWNGPVCTLCDVATLLFVWFYYSSGGKLDESFFRSAYFIQGLLMQTLIFHLIRTEKIPFIMEIASWPVLASTVVISAIGIAIPFSIIGDFMGFTDLPLSYFGFLVVLFVGYFTVGQIVKRIYIVVYKKWL

>GbACA25

MQENSPFRIDQPTLIELVKEKKIEKLRKHGGVDGVASGPGTDTQVGVSGSAEDIERRREAFGSNTYKKPPTKGFFHFVVEAFKDLTIMILLGCAALSLGFGIKEHGLKDGWYDGGSIFVAVFLVIAVSAVSNYRQDRQFDKLSKVSNNIQVDVVRGGRRQQISIFDIVVGDIVCLKIGDQVPADGLFIDGHSLQIDESSMTGESDHVEVNGNQNPFLLSGTKVADGYARMLVTSVGMNTTWGQMMCQISRDTNDETPLQARLNKLTSSIGKVGLAVAFLVLVVLLVRYFTGHTTDENGNREFNGSKTKKNSPFRIDQPTLIELVKEKKIEKLRKHGGVDGVASGPGTDTQVGVSGSAEDIERRREAFGSNTYKKPPTKGFFHFVVEAFKDLTIMILLGCAALSLGFGIKEHGLKDGWYDGGSIFVAVFLVIAVSAVSNYRQDRQFDKLSKVSNNIQVDVVRGGRRQQISIFDIVVGDIVCLKIGDQVPADGLFIDGHSLQIDESSMTGESDHVEVNGNQNPFLLSGTKVADGYARMLVTSVGMNTTWGQMMCQISRDTNDETPLQARLNKLTSSIGKVGLAVAFLVLVVLLVRYFTGHTTDENGNREFNGSKTKTIATECGILKPGQDLSSGAVVEGEEFRNYTPQERMEKVEKIQVMARSSPFDKLLMVQCLKQKGHVVAVTGDGTNDAPALKEADIGLSMGIQGTEVAKESSDIVILDDNFASVATVLRWGRCVYTNIQKFIQFQLTVNVAALCINFVAAVSAGEVPLTAVQLLWVNLIMDTLGALALATERPTKELMEKPPVGRTEPLITNIMWRNLLAQALYQIAVLLTLQFSVFNEFNARKLEKKNVFEGIHKNKLFIGIIGVTILLQVAMVEFLKRFADTERLTWGQWGACIAVAAVSWPLGWVVKCLPVPQKPIFSYLKWRK

>GbACA26

MSTKFSSLITKTATANDEARVVHRSPSHVSLAVMQENSPFRIDQPTLIELVKEKKVEKLRKHGGVDGVASGLGTDTQVGVSGSAEDIERRHEAFGSNTYKKPPTKGFFHFVVEAFKDLTIMILLGCAALSLGFGIKEHGLKDGWYDGGSIFVAVFLVIGVSAVSNYRQNRQFDKLSKVSNNIQVDVVRGGRRQQISIFDIVVGDIVCLKIGDQVPADGLFIDGHSLQIDESSMTGESDHVEVNGNQNPFLLSGTKVADGYARMLVTSVGMNTTWGQMMCQISRDTNDETPLQARLNKLTSSIGKVGLAVAFLVLVVLLVRYFTGHTTDENGNREFNGSKTKTIATECGILKPGQDLSSGAVVEGEEFRNYTPQERMEKVEKIQVMARSSPFDKLLMVQCLKQKGHVVAVTGDGTNDAPALKEADIGLSMGIQGTEVAKESSDIVILDDNFASVATVLRWGRCVYTNIQKFIQFQLTVNVAALCINFVAAVSAGEVPLTAVQLLWVNLIMDTLGALALATERPTKELMEKPPVGRTEPLITNIMWRNLLAQALYQVAILLTLQFSGESILGVTEKVNDTLIFNIFVFCQVFNEFNARKLEKKNVFEGIHKNKLFIGIIGVTILLQVVMVEFLKRFADTERLSWGQWGACIAVAAVSWPLGWVVKCLPVPQKPIFSYLKWWK

>GbACA18

MSGTSSGNGLLHLNDVEAGLSKDNADLDHHLDPDAGTSDPFDIAHTKSASPETLKRWRQAALVLNASRRFRYTLDLKKEEEKEQRKRMIRAHAQVIRAALLFKLAGENQIVSGAPVASPSAGDDYKIGLEQLASMTRDHKLSALEQYGGVKGLSGLLRTNLEKGIDEDEADLLNRRNAFGSNTYPRKKGRSFWMFLWEAWQDLTLIILIIAAAVSLGLGIKTEGLTEGWYDGGSIFFAVFLVILVTATSDYRQSLQFQNLNEEKRNIQLEVVRGGRTVKVSIYDLVVGDLVPLKIGDQVPADGVLIAGHSLAIDESSMTGESKIVHKNQNDPFLMSGCKVADGFGTMLVTGVGINTEWGLLMASISEDTGEETPLQVRLNGVATFIGVVGLSVAVSVLAILLARYFTGNTEDPDGATQFIKGRTKFDDAFNDVVKIF

>GbHMA20

MHKEGELWGFVTKASLSIDAQIPKRGYGTRNELAGIRGESYVFRMGMTCSACAGSVEKAIKRLPGIKEAVVDVLNNKAQVMFYPSFEESIREAIEDARFQAALIQDETDDKSVQVCLIRINGMTCTSCSSTLENALQAVPGVQKVQVALATEEAQIHHDPKIITCNQLMEKIEETGFGAVLVSTGEDMSKINLRIDGVRTVNSMRMLENSLQALPGVQAVQISPELKKIAVSYKPDMTGRRNFIKVIDSTGSSRRFKATIYSEGEGGGRESHRKKEIKQYFRSFLWSLIFTTPVFLTSMIFMYIPGIKHGLDTKVVNMLTIGEVIRWVLSTPVQFIIGRRFYTGSYKALRHGSANMDVLIALGTNAAYFYSVYSVIRAASPDFEGTDFFETSAMLISFILLGKYLEVLAKGKTSEAIAKLMNLALETAMLLSLDEEGNVIGEEEIDSRLIQKNDIIKILPGAKVASDGFVLWGQSHINESMITGEIVRLVESAQMAKAPVQKFADRISKYFVPLVIMLSFSTWLAWFLAGKLHGYPESWIPSSMDSFELALQFGISVMVIACPCALGLATPTAVMVGTGVGASLGVLIKGGQALEGAHKVNCIVFDKTGTLTVGKPVVVNTRLLKNMVLHEFYELVAATEANSEHPLAKAIIQYAKFREDEENPAWPEARDFVSITRHGVKAIVRDKEIIVGNKSLMLENNIVIPVDAQDMLTETESMAQTGILVSIDGEVTGVLAISDPVKPGAQEVISILKSMNVRSIMVTGDNWGTASSIASQIGIETVVAETKPEQKAEKVKELQAEGYAVAMVGDGINDSPALVAADVGMEIGAGTDIAIEAADIVLMKSNLEDVITAIYLSRKTFSRIRLNYIWAFGYNMLGIPIATGSLFPPTGFRLPPWIAGAAMAASSVSVVCCSLL

>GbHMA18

MATKLLASSCVRKESSGDLSPRPHYPSIPKYPKGVTAQETSLQGSEAKAMLSVMGMTCSACAGSVEKAIKRPPGIKEAVEESIREAIEDAGFQAALIQDETDYKSVQAAPGVQKVQVALATEEAQIHHDPKIITCNQLMEKIEETGFGAVLVSTGEDMSKINLRIDGVRTVNSMRVLENSLQALPGVQAVQISPELKKIAVSYKPDMTGPRNFIKVIDSTGSSRRFKATIYSEGEGGGRESHRKEEIKQYFRSFLWSLIFTTPVFLTSMVFMYIPGIKHGLDTKVVNMLTIGEVIRWVLSTPVQFIIGRRFYTGSYKALRHGSANMDVSIALGTNTTYFYSVYTVIRAASSPDFEGTDFFETSAMLISFILLGKYLEVLAKGKTLEAIAKLMNLAPETAMLLSLDEEGNMIGEEEIDSRMIQKNDIIKILPGAKVASDGFVLWGQSHINESLITGEARPVAKRKGNTVIGGTVNENGVLHIKATKVGSESDHAFVFNVACMVFSWKVPWYPESWIPSSMDSFELALQFGISVMVIACPCALGLVTPTAVMVGTGVSASLGVLIEGGQALEGAHKVNCIVFDKRGTLTVEKPVVVNTRLLKNVVLHEFYELDATTEANSEHPLAKAIIEYAKKFREDEENPAWLEARDFVSITGHGVKAIVRDKEIIVGNKSLMLENNIVIPVDAQDMLTETESMAQTGILVSIDGEVTGVLAISDPVKPGGQKVISILKSMNVRSIMVTGDNWGTASSIASQIGIETVVAETKPEQKAEKVKELQAEGYAVAKVGDGINDLPALEAADVGMAIGAGTDIAIEAADIVLMKSNLEDVITAIHLSKKTFSHICLNYIWAFGYNMLGTPIAAESLFPPTRFRLPLWIAGAAMVASSVSVVCC

>GbACA1

MFAGASNIGGCNEHGDEDTLSVSLLNTTANSSRKNWTRLYNLFLVVGSRIKSGREELSIEEDGSVPKSSSNSGSADCCTIDMVPTEIKEFFIEEDGSVPKSSSTSGSADCSTNDMFAPKIEVFPKEDGSVPSIIFLRKAAQIVRDKDLTSLEEEGGVRRVKSRLQSILAHEIDRSFEEQATAVIVTGTTFDAKVFVVSFVEASKTPTIFPLLVFAALSFTIEMMEAEPKHGWHDGVAILVAVFMLLIFRSVANYRRARRLQKRKKFHVSVLKDGQPKTITISTLVVGHILCLKKGDFVPADGLFVSENGLKLDDKLNPNINRDDNPFLLAGSKIMEGEGHMLVTSVGDNSVLPTVDPDEKSLMEDQIDKTNAYMEYVGLSISLLVSALVLINLLARKMDKNSNIMQEMKGDVSAYRVIKIFARVFLNPRGKVQIFTGVLTVMVTSLQHGMPVVITISLSYWKKKMASGDANVQSLSSCGTIGIVSVICFDENTVVACKEVMGSIVGALKEEEVGCKLMSKDELATAQVMAHEIGILNPDLRDMAIENKDLHELAATAEGMNKIAVIGSCKLKDKIRILQRLKQEGHVVAFIGGMATNDDLALKAADVGITICECSTKMARENSEIVISSRNSLRSLIRCLKMGKCAYGNVRTFTEIQLTATLASLLVTLVTTSILDESPITGIHMLWVNSIIYILGGLMMVMESYGHQELMNNQPARRMKSLLTKTMWRNVAIRAASDACHLLVLQFIGQAILQINKDVVKTMLPKEDGSVPRSADDIRINIPEGVDGNANIIFLRKAAQIVRDKDLTSLEEEGGVRRVKSRLQSILAQEIDRSFEEQATAVIVTGTTFDAKVFVVSFVEASKTPTIFLLLVFAALSFTIEMMEAEPKHGWHDGVAILVAVFMLLIFRSVANYRRARRLQKRNKFHVSVLKDGQPKTITISTLAVGHILCLQKGDFVPADGLFVSENGLKLDDKLNPNINRDDNPFLLAGSKIMEGEGHMLVTSVGDNSVLPTVDPDEKSLMEDQIDKTNAYMEYVGLSISLLVSALVLINLLARKMDKNSNIMQEMKGDVSAYRVIKIFARVFLNPRGKVQIFTGVLTVMVTSLQHGMPVVITISLSYWKKKMASGDANVQSLSSCGTIGIVSVICFDENTVVACKEVMGSIVGALKEEEVGCKLMSKDELATAQVMAHEIGILNPDLRDMAIENKDLHELAATAEGMNKIAVIGSCKLKDKIRILQRLKQEGHVVAFIGGMATNDDLALKAADVGITICECSTKMARENSEIVISSRNSLRSLIRCLKMGKCAYGNVRTFTEIQLTATLASLLVTLVTTSILDESPITGIHMLWVNSIICILGGLMMVMESYGHQELMNNQTVRRMKSLLTKTMWRNVAIRAASDACHLLVLQFIGQAILQINKDVVKTMVFNGFILCQVLDLFISTIIIFARNEEVSVSVVRFMCSSHWFLMASGGVMAMQVVVVELLQSLAEYERLNVMQWGFCFIYAAWLCGTGLTVKLIADSASEVLWSSRSSGSLFGYLRLRRSSLRLFVIPFSVCVVASFSYCYVNPDIA

>GbAHA13

MASDGDISLEQIKNEIVDLENIPVEEVFKLLKCTKEGLTTAEGETRLSIFGHNKLEEKKDNKVLKFLGFMWNPLSWVMEAAAIMAIVLANGGGKPPDWPDFVGIVTLLLINSTISFIEENSAGNAAAALMAGLAPKTKVYYTYIYVCELDAIVIATGIHSFFGKAAHLVDNTNNVGHFQQVLTSIGNFCIFSIGIGMVIEIIVMYPIQHRNYRDGIDNLLVLLIGGIPIAMPTVLSVTMAIGSHRLSEQGAITKRMTAIEEMAGMDILCSDKTGTLTLNKLTVDKNLVEVFINDMDVDTLVLLAARASRVENQDAIDACIVGMLSDPKQAREGITELHFLPFNPVDKRTAITYTDSKGEWHRCSKGAPEEIIDLCGLTGGLRKKALSIIDGYANRGLRSLGVARQTIPEKTKESPGGPWEFVGLLPLFDPPRHDSAETIKRALELGISVKMITGDQLAIGKETGRRLGMGTDMYPSSALLGQCGDEDIAAIPIDDLIEKADGFAGVFP

>GbACA3

MISLIFLFRAGASNIGGCNEHGDEDTLSVSLLNTTANSSRKNWTRLYNLFLVVGSRIKPEREEVSIEEDGSVPKSSSNSGSADCCTIDMVPTEIEEFFIEEDGSVPKSSSTSGSADCSTNDMFAPKIEVFPKEDGSVPRSADDIRINIPEGVDGNANIIFLRKAAQIVRDKDLTSLEEEGGVRRVKSRLQSILAQEIDRSFEEQATAVIVTGTTFDAKVFVVSFVEASKTPTIFLLLVFAALSFTIEMMEAEPKHGWHDGVAILVAVFMLLIFRSVANYRRARRLQKRNKFHVSVLKDGQPKTITISTLAVGHILCLQKGDFVPADGLFVSENGLKLDDKLNPNINRDDNPFLLAGSKIMEGEGHMLVTSVGDKTVLAIVDPDEKSLMEDQIDKTNAYMEYVGLSISLLVSALVLINLLARKMDKNSNIMQEMKGDVSAYRVIKIFARVFLNPRGKVQIFTGVLTVMVTSLQHGMPVVITISLSYWKKKMASGDANVQSLSSCGTIGIVSVMCFDENTVVACKEVMESIVGTLKEEEVGCKLMSKDELPTAQAMAREIGIQNPDLRDMAIENKDLHELAATAEGMNKIAVIGSCKLEDKIRILQRLKQEGHVVAFIGGMATNDDLALKAADVGITICECSTKMARENSEIVISSRNSLRSLIRCLKMGKCAYGNVQTFNQIQLTAILASLLVTLVTTSILDESPITGIHMLWVNSIICILGGLMMVMESYGHQELMNNQTVRRMKSLLTKTMWRNVAIRAASDACHLLLLQFIGQAILQINKDVVKTMVFNGFILCQVLDLFISTIIIFARNEEVSVSVVRFMCSRHWFLMASGGVMAMQVVVVELLQSLAEYERLNVMQWGFCFIYAAWMCGTGLTVKLIADSASEGLWSSRSSGSQFGYLRLR

>GbACA2

MSAGASNIGGCNEHRDEDTLSVSLLNTTANSSRKNWTRLYNLFLVVGSRIKPEREEVSIEEDGSVPKSSSNSGSADCCTIDMVPTEIEEFFIEEDGSVPKSSSTSGSADCSTNDMFAPKIEVFPKEDGSVPRSADDIRINIPEGVDGNANIIFLRKAAQIVRDKDLTSLEEEGGVRRVKSRLQSILAQEIDRSFEEQATAVIVTSTTFDAKVFVVSFVEASKTPTIFLLLVFAALSFTIEMMEAEPKHGWHDGVAILVAVFMLLIFRSVANYRRARRLQKRNKFHVSVFAGASNIGGCNEHGDEDTLSVSLLNTTANSSRKNWTRLYNLFLVVGSRIKPEREEVSIEEDGSVPKSSSNSGSADCCTIDMVPTEIEEFFIEEDGSVPKSSSTSGSADCSTNDMFAPKIEVFPKEDGSVPRSADDIRINIPEGVDGNANIIFLRKAAQIVRDKDLTSLEEEGGVRRVKSRLQSILAQEIDRSFEEQATAVIVTSTTFDAKVFVVSFVEASKTPTIFLLLVFAALSFTIEMMEAEPKHGWHDGVAILVAVFMLLIFRSVANYRRARRLQKRNKFHVSVLKDGQPKTITISTLAVGHILCLQKGDFVPADGLFVSENDLKLADILNPNINRDDNPFLLAGSKIMEGEGHMLVTSVGDKTVLAIVDPDEKSLMEGQIDKTNACMEYVGLSISLLVSALVLINLVVRKMDKNSNTMPEIKGDVSAYRVIKIFARVFLNPRGKVQILTGVLTVMVTSLQHGIPVVITISLWYWKKKMSSGDANVQNLSSCGTIGIVSVMCFDENTVVACKEVMESIVGTLKEEEVGCKLMSKDELPTAQAMAREIGIQNPDLRDMAIENKDLHELAATAEGMNKIAVIGSCKLEDKIRILQRLKQEGHVVAFIGGMATNDDLALKAADVGITICECSTKMARENSEIVISSRNSLRSLIRCLKMGKCAYGNVQTFNQIQLTAILASLLVTLVTTSILDESPITGIHMLWVNSIICILGGLMMVMESYGHQELMNNQTVRRMKSLLTKTMWRNVAIRAASDACHLLLLQFIGQAILQINKDVVKTMVFNGFILCQVLDLFISTIIIFARNEEVSVSVVRFMCSRHWFLMASGGVMAMQVVVVELLQSLAEYERLNVMQWGFCFIYAAWMCGTGLTVKLIADSASEGLWSSRSSGSQFGYLRLRRSNIRLFVIPFSACVVASFSYYYVNPDNA

>GbALA1

MDSKTPVENLYSIEPALSSSSRSNFSARSKSSGGSSIREVNFSDVGPKPVRYGSQGAESDTYSMSQKEINDEDARLVHINDPVNTNERFDFARNSIRTAKYSILTFLPRNLFEQFHRVAYVYFLVIAVLNQLPQLAVFGRTASVLPLAFVLLVTAVKDAYEDYRRHRADRIENNRLALVLVNNEFQQKKWKNIQVGEIIKIQANETIPCDMVLLSTSEPTGVAYVQTINLDGESNLKTRYAKQETLQKIPEKENVSGLIKCEKPNRNIYGFQANMEVDGKRVSLGPSNIILRGCELKNTTWAVGVAVYAGSETKAMLNNSGAPSKRSRLETHMNLEIIFLSLFLVALCTVVSVCAAVWLRRHRDELDYLPFYRRKDFSEDEEKNYNYYGWGLEIFFTFLMSVIVFQIMIPISLYISMELVRVGQAYFMIRDAEMYDESSNTRFQCRALNINEDLGQIKYVFSDKTGTLTENKMEFQCASIWGVDYNGGKATSQDQKDGYFVQADGQVLRPKMVVKTDPELLQFVRNGKETKEGSYVHDFFLALAACNTIVPIIVDTPDPTLKLIDYQGESPDEQALVYAAAAYGFMLIERTSGHIVIDIQGERKRFNVLGLHEFDSDRKRMSVILGFPNQSVKVFVKGADTTMFSVIDRSLNTSIIRATEGHLQSYSSIGLRTLVIGMRELSTSEFEEWHSAFEVASTALMGRARLLRKIASNIESNLCILGASGIEDKLQQGVPEAIESLRTAGIKVWVLTGDKQETAISIGYSSKLLTSKMTQVIVNSNSKESCRKSLEDAIIMSKKLTTMSGTTNETGRTLGSGSTPVALIIDGTSLVHILDSELEERLFELACNCSVVLCCRVAPLQKAGIVSLVKKRTSDMTLAIGDGANDVSMIQMADVGVGISGQEGRQAVMASDFAMGQFRFLVPLLFVHGHWNYQRMGYMILYNFYRNAVFVLVLFWYVLFTGFTLTTAINEWSSVLYSVIYTSVPTIVVGILDKDLSRLTLLKHPQLYGAGHRDECYNKTLFWITMLDTLYQSVIVFFIPLLAYWGSTIDASSIGDLWTLAVVILVNLHLAMDVIQWNWITHAAIWGSIIATFICVIIIDAIPSLVGYWAIFEIAKTRLFWCCLLAIIVTALIPRFVVKVLYQFYAPCDVQIAREAEKFWAQNQSAAVEVEMSPILDHPRR

>GbALA2

MSGWDNIRSSTRSQQGRSHSLNQREPSRTVTLGRVQPQAPAFRTIYCNDRDANFAHRYRGNSVSTTKYNFFTFLPKGLYEQFRRVANLYFLMVSILSATPYSPVHPVTNVVPLSLVLLVSLIKEAFEDWKRFQNDMAINSTPVDVLQDQRWESIPWKKLQVGDIIRVKQDGFFPADMLLLASTNADGVCYIETANLDGETNLKIRKALERTWDYVTPEKACEFKGEVLCEQPNNSLYTFTGNLVIDNQTLPLSPNQILLRGCSLKNTEFVVGVVIFTGHETKVMMNSMNVPSKRSTLERKLDKLILTLFGTLFTMCLIGAIGSGVFIDRKYYFLGLSKSVEDQFNPNRRFLVVLLTMLTLLTLYSTIIPISLYVSIEMVKFIQSTQFINKDLNMYHAETDTPALARTSNLNEELGQVEYIFSDKTGTLTRNLMEFFKCTIGGEIYGTGMTEIERGVAERKGIKVQEVPTSINSVREKGFNFDDVRLMRGAWRNEPNPEACKEFFRCLAICHTVLPEGDESPEKIKYQAASPDEAALVLAAKHFGFFFYRRTPTMIYVRESHVERMGKIQDVSYEILNVLEFNSTRKRQSVVCRYPDGRLVLYCKGADTVIYERLVGGSDDLKKVTREHLEIFGSAGLRTLCLAYKDLAPDVYESWNEKFIQAKSSLRDRERKLDEVAELIEKDLILIGATAIEDKLQEGVPDCIETLSRAGIKIWVLTGDKMETAINIAYACNLLNNEMKQFIISSETDAIREVEERGDQVEIARFIKEEVKKQLKKCLDEAQQYFHSVSGPKLALIIDGKCLMYALDPSLRIMLLNLSLNCSSVVCCRVSPLQKAQVTSLVKKGARKITLSIGDGANDVSMIQAAHIGVGISGLEGMQAVMASDFAIAQFRFLKDLLLVHGRWSYIRLCKVVTYFFYKNLTFTLTQFWFTFYTGFSGQRFYDDWFQSLYNVIFTALPVIIVGLFDKDVSSSLSKRYPELYKEGIKNMFFKWRVVAIWAFFAVYQSLVFYYFVIVSSSTSQGSSGKMFGLWDVSTMAFTCVVVTVNLRLIMICNSITRWHYISVGGSIVAWFLFIFLYSGIMTPYDRQENIFWVIYVLMSTFYFYITLLLVPVAALLGDFLYLGVQRWFFPYDYQIVQEIHKDEADDSGRTDLLGIYNQLTPDEARSYALSQLPRELSKHTGFAFDSPGYESFFASQLGVYAPQKAWDVARRASM

>GbALA3

MSGWDNIRSSTRSQQGRSHSLNQREPSRTVTLGRGNSVSTTKYNFFTFLPKGLYEQFRRVANLYFLMVSILSATPYSPVHPVTNVVPLSLVLLVSLIKEAFEDWKRFQNDMAINSTPVDVLQDQRWESIPWKKLQVGDIIRVKQDGFFPADMLLLASTNADGVCYIETANLDGETNLKIRKALERTWDYVTPEKACEFKGEVQCEQPNNSLYTFTGNLVIDNQTLPLSPNQILLRGCSLKNTEFVVGVVIFTGHETKVMMNSMNVPSKRSTLERKLDKLILTLFGTLFTMCLIGAIGSGVFIDRKYYFLGLSKSVEDQFNPNRRFLVVLLTMLTLLTLYSTIIPISLYVSIEMVKFIQSTQFINKDLNMYHAETDTPALARTSNLNEELGQVEYIFSDKTGTLTRNLMEFFKCTIGGEIYGTGMTEIERGVAERKGIKVQEVPTSINSVREKGFNFDDVRLMRGAWRNEPNPDACKEFFRCLAICHTVLPEGDESPEKIKYQAASPDEAALVLAAKHFGFFFYRRTPTMIYVRESHVERMGKIQDVSYEILNVLEFNSTRKRQSVVCRYPDGRLVLYCKGADTVIYERLVGGSDDLKKVTREHLEKFGSAGLRTLCLAYKDLAPDVYESWNEKFIQAKSSLRDRERKLDEVAELVEKDLILIGATAIEDKLQEGVPDCIETLSRAGIKMWVLTGDKMETAINIAYACNLLNNEMKQFIISSETDAIREVEERGDQVEIARFIKEGVKKQLKKCLDEAQQYFHSVSGPKLALIIDGKCLMYALDPSLRIMLLNLSLNCSSVVCCRVSPLQKAQVTSLVKKGARKITLSIGDGANDVSMIQAAHIGVGISGLEGMQAVMASDFAIAQFRFLKDLLLVHGRWSYIRLCKVVTYFFYKNLTFTLTQFWFTFYTGFSGQRFYDDWFQSLYNVIFTALPVIIVGLFDKDVSSSLSKRYPELYKEGIKNMFFKWRVVAIWAFFAVYQSLVFYYFVIVSSSTSQGSSGKMFGLWDVSTMAFTCVVVTVNLRLIMICNSITRWHYISVGGSIVAWFLFIFLYSGIMTPYDRQENIFWVIYVLMSTFYFYITLLLVPVAALLGDFLYLGVQRWFFPYDYQIVQEIHKDEADDSGRTDLLGIYNQLTPDEARSYALSQLPRELSKHTGFAFDSPGYESFFASQLGVYAPQKAWDVARRASMRSKPKPKPNKIN

>GbALA4

MARVRKRNIHLSKSCSFSCCRRPSQPSSTDEHDRIGQKGYSRVVYCNEPDCEEQIRLKYRGNYVSTTKYTAANFIPKSLFEQFRRVANIYFLVVACVSFSPLAPYSAPSVLVPLIVVIGATMAKEGIEDYRRRQQDVEANNRTVEVYDRNSSFNETKWKNLRVGDIVKVHKDEYFPADLLLLSSDYEDGVCYVETMNLDGETNLKSKHPLEVTFFIRDVETVKGFRAVIKCEDPNEHLYKFVGTLYYECQQYALSPQQILLRGTKLKGPDYINGVVIFTGHDTKVMQNAMDPPSKRTRIERRMDKIIYVLFSALILVSFIGSLLFGIETKKDGGDYGRWYLRPDITTVFFDPRRPSVAAFLHFLTGLMLYGYLIPISLYVSIEICKVLQSIFINQDQAMYDEETKRSAHARTSNLNEELGQVFTILSDKTGTLTCNKMEFVKCSIAGTAYGCGMTEVEIALARKRGETLDEQRDIDTVESREPVKGFNFRDGRIMNRKWVHEPYGDYIEKFFRVLALCHTAVPEVLDPGKIFYEAESPDEAAFVIAAREVGFQFVKRNQTSIQLRELDRSSGEIVDRVYELLHVLEFSSTRRRMSVIVRNPERQLLLLAKGADSVIFERLSEQGRMFEAETKEHIERYSEAGLRTLAVAYRELDDDEYKRWEQEYVKAKTSVSANRDDLLDVMAELIERDLILLGATAIEDKLQKGVPDCIDKLAQAGINIWVLTGDKKGTAINIGYACSLLRHGMKEILVILENPGIAAVERDTEDFAKASEIVEKQIDEGISQVTGGSSTQFGLIIDGQSLIFALDEKLIMRFMELAMKCATVICCRSSPKQKAIVTRWVKSVTGRTTLAIGDGANDVGMIQEADIGVGITGVEGMQAAMSSDFSIAQFRFLERLLLVHGHWCYRRITMMICYFFYKNITFGFTLFWFEAYSSFSAQPAYNDWYMSSYNVLFTSLPVIALGVFDQDFSARHCLEYPSLYKEGIDDVLFRWTHILGWMFNGVLSSVIIFFLTANSITGQAFRKDGQVADYSVLGLTMYTCVVLAVNCQMALCINYFTWIQHLFIWGSITLWFKFLLVYGSIPPTLSTTAYKVFIEACAPSIQYWLTIILVVIATLLPWFSYRAFQTRFRSMVRDCIQT

>GbALA5

MARVRKRNIHLSKSCSFSCCRRPSQPSSTDEHDRIGQKGYSRVVYCNEPDCEEQIRLKYRGNYVSTTKYTAANFIPKSLFEQFRRVANIYFLVVACVSFSPLAPYSAPSVLVPLIVVIGATMAKEGIEDYRRRQQDVEANNRTVEVYDGNSSFNETKWKNLRVGDIVKVHKDEYFPADLLLLSSEYEDGVCYVETMNLDGETNTKSKHPLEVTSFIRDVETVKGFRAVIKCEDPNEHLYSFVGTLYYECQQYALSPQQILLRGTKLKNTDYINGVVIFTGHDTKVMQNAMDPPSKRTRIERRMDRIIYVLFSALILVSFVGSLLFGIETKKDGGDYGRWYLRPDITTVFFDPRRPSVAAFLHFLTGLMLYGYLIPISLYVSIEICKVLQSIFINQDQAMYDEETKRSAHARTSNLNEELGQVFTILSDKTGTLTCNSMEFVKCSIAGTAYGRGMTEVEIALARKRGETLDEQRDIDTVESREPVKGFNFRDGRIMNRKWVNEPNRDYIEKFFRVLALCHTAVPEVLDPGKIFYEAESPDEAAFVIAAREVGFQFFKRNQTSIQLRELDRSSGEIVDRVYELLHVLEFSSARRRMSVIVRNPERQLLLLAKGADSVIFERLSEEGRMFEAETKEHIERYSEAGLRTLAVAYRELDDDQYKRWEQEYVKAKTSVSADRDDLLDEKADMIERDLILLGATAIEDKLQKGVPDCIDKLAQAGIHIWVLTGDKKGTAINIGYACSLLRHGMKEILVILEKPGIEAIERDKEEFAKAYKIVEKQIDEGISQVTGESNTQFGLIIDGKSLIFALDKNLIIRFMELAMKCATVICCRSSPKQKAIVTRWVKSKTGRTTLAIGDGANDVGMIQEADIGVGITGVEGMQAAMSSDFSIAQFHFLERLLLVHGHWCYRRITMMICYFFYKNITFGFTLFWFEAYSSFSAQPAYNDWYMSSYNVLFTSLPVIALGVFEQDFSARHCLKYPSLYKEGIDNVLFRWIHILGWMFNGVLSSVIIFFLTTNSITGQAFRKDGQVADYSILGLTMYTCVVLAVNCQMALSINYFTWIQHLFIWGSITLWFKFLLVYGSIPPTLSTTAYKVFIEACAPSIRYWLTIILVVIATLLPLFSYRAFQTRFQPMVHDSIQTRRP

>GbALA6

MTGGRRRKQHFSRIHAFSCGKASFRGDHSLIGGPGFSRVVYCNDPECFEASLRNYAGNYVRSTKYTLATFFPKSLFEQFRRVANFYFLICAILSFTPLSPYSAVSNVLPLVVVIGATMGKEAVEDWRRKKQDTEVNNRKVKMHQSDGIFEPTKWMDLKVGDIVKVEKDEFFPADLILLSSSYEEAICYVETMNLDGETNLKLKGASDVTSSLHDDASFQDFKATIRCEDPNANLYSFVGSLELGDEQYPLSPQQLLLRDSKLRNTDYIFGVVIFTGRDTKVIQNSTEPPSKRSKIEKRMDNIVYFLFAVLVGLSIIGSIFFGIETREDLENGKMRRWYLRPDDTTIYYNPKRAAVAAILQFLTALMLYSYLIPISLYVSIEIVKVLQSIFINQDLHMYHEETDKPAHARTSNLNEELGQVDTILSDKTGTLTCNSMEFIKCSIAGTSYGHGITEVERALAWRKGSPLAREVPEINGQVEEFKKEKPSVKGFNFVDERIMNGNWLKEPHADVIQKFLRLLAICHTAIPEVDEETGRISYEAESPDEAAFVVAARELGFEFYERTQTSISLYEFDLSGKKVERSYKLLNILEFSSSRKRMSVILQNEEGKLLLLCKGADSVMFERLAKNGQEFAEQAKEHIEEYADAGLRTLVLAYREINEEEYVEFNEKFTEAKNIVSADREEMIEEVAESIERDLILLGATAVEDKLQNGVPECIDKLAQAGIKIWVLTGDKMETAINIGFACSLLRQGMKQIIINSDTPEDKALEKSGDKTAAAADTEVNNRKVKMHQSDGIFEPTKWMDLKVGDIVKVEKDEFFPADLILLSSSYEEAICYVETMNLDGETNLKLKGASDVTSSLHDDASFQDFKATIRCEDPNANLYSFVGSLELGDEQYPLSPQQLLLRDSKLRNTDYIFGVVIFTGRDTKVIQNSTEPPSKRSKIEKRMDNIVYFLFAVLVGLSIIGSIFFGIETREDLENGKMRRWYLRPDDTTIYYNPKRAAVAAILQFLTALMLYSYLIPISLYVSIEIVKVLQSIFINQDLHMYHEETDKPAHARTSNLNEELGQVDTILSDKTGTLTCNSMEFIKCSIAGTSYGHGITEVERALVWRKGSPLAREVPEINGQVEEFKKEKPLVKGFNFVDERIMNSNWLNEPHADVIQKFLRLLAICHTAIPEVDEETGRISYEAESPDEAAFVVAARELGFEFYERTQTSISLYEFDLSGKKVKRSYKLLNILEFSSSRKRMSVILQNEEGKLLLLCKGADSVMFERLAKNGIEFAEQTKEHIEEYADAGLRTLVLAYREINEEEYVEFNEKFMEAKNIVSADREEMIEEVAESIERDLILLGATAVEDKLQNGVPECIDKLAQAGIKIWVLTGDKMETAINIGFACSLLRQGMKQIIINSDTPENKALEKSGDKTAAAAAYKASVLQQIAEGRQLLTSSNENSEALALIVDGKSLTYALEDDVKDAFLELTIGCASVICCRSSPKQKALVTRLVKTKTGSTTLAIGDGANDVGMLQEADIGVGISGVEGMQAVMSSDIAIAQFRFLERLLLVHGHWCYRRISSMICYFFYKNIVFGFTLFFYEIYASFTGQAVYNDWFLSFYNVFFTSLPVIALGVFDQDVSSRLCLKFPLLYQEGIQNVLFSWLRIVAWAFNGVLSATVIFFFCIRATQHQAFRKGGEVVGLEILGTTMYTCVVWVVNCQMTLSISYFTYIQHLFIWGSIILWYIFLMAYGAMATSISTTAYKVFIESCAPAGMYWLLTLLVLISSLLPYFIYSAIQVRFFPSYHQMIQWIRSDGQSDDPEYCHMVRQRSLRPTTVGYTARLEAKSRSSRKGGEDHQ

>GbALA7

MTGGRRRKQHFSRIHAFSCGKASFKGDHSLIGGPGFSRVVYCNDPECFEASLRNYAGNYVRSTKYTLATFFPKSLFEQFRRVANFYFLICAILSFTPLSPYSAVSNVLPLVVVIGATMGKEAVEDWRRKKQDTEVNNRKVKMHQSDGIFEPTKWMDLKVGDIVKVEKDEFFPADLILLSSSYEEAICYVETMNLDGETNLKLKGASDVTSSLHDDASFQDFKATIRCEDPNANLYSFVGSLELGDEQYPLSPQQLLLRDSKLRNTDYIFGVVIFTGRDTKVIQNSTEPPSKRSKIEKRMDNIVYFLFAVLVGLSIIGSIFFGIETREDLENGKMRRWYLRPDDTTIYYNPKRAAVAAILQFLTALMLYSYLIPISLYVSIEIVKVLQSIFINQDLHMYHEETDKPAHARTSNLNEELGQVDTILSDKTGTLTCNSMEFIKCSIAGTSYGHGITEVERALAWRKGSPLAREVPEINDQVEEFKKEKPSVKGFNFVDERIMNGNWLKEPHADVIQKFLRLLAICHTAIPEVDEETGRISYEAESPDEAAFVVAARELGFEFYERTQTSISLYEFDLSGKKVERSYKLLNILEFSSSRKRMSVILQNEEGKLLLLCKGADSVMFERLAKNGQEFAEQAKEHIEEYADAGLRTLVLAYREINEEEYVEFNEKFTEAKNIVSADREEMIEEVAESIERDLILLGATAVEDKLQNGVPECIDKLAQAGIKIWVLTGDKMETAINIGFACSLLRQGMKQIIINSDTPEDKALEKSGDKTAAAAAYKASVLQQIAQGRLLLTSSNENSEALALIVDGKSLTYALEDDVKDAFLELAIGCASVICCRSSPKQKALVTRLVKTKTGSTTLAIGDGANDVGMLQEADIGVGISGVEGMQAVMSSDIAIAQFRFLERLLLVHGHWCYRRISSMICYFFYKNIVFGFTLFFYEIYASFSGQAVYNDWFLSFYNVFFTSLPVIALGVFDQDVSSRLCLKFPLLYQEGIQNVLFSWLRIIAWAFNGVLSATVIFFFCIRAVQHQAFRKGGEVVGLEILGTAMYTCVVWVVNCQMALSVSYFTYIQHLFIWGSIILWYIFLMAYGAMDPSISTTAYKVFIESCAPAGMYWLLTLLVLISSLLPYFIYSAIQVRFFPSYHQMIQWIRSDGQSDDPEYCHMVRQRSLRPTTVGYTARLEAKSRSSRKGGEDHQ

>GbALA8

MSGGRRRKVLMSRIYGIACGKASFKEDHSQIGGPGFSRVVYCNEPNSLEAGTRNYSDNYVSTTKYTIATFLPKSLFEQFRRVANFFFLVTGILSFTAIAPYSALSAIVPLIIVIGATMIKEGVEDWRRQQQDIEVNNRKVKVHQGDGNFHHTEWKNLRVGDIVKVEKDEFFPTDLILLASSYEDAVCYVETMNLDGETNLKLKQALEVTSSLHNDYNFRDFKAIVKCEDPNANLYSFVGTMEFEEQQHPLSPQQLLLRDSKLRNTDYIYGAVVFTGHDTKVMQNATDPPSKRSKIEKTMDRVIYLMFFIVFIMGFVGSIFFGIATENDYEGGRIKRRWYLRPDNAEIFFDPERAPVAAIYHFLTALLLYSYFIPISLYVSIEIVKVLQSIFINQDSHMYYEEADKPAHARTSNLNEELGQVDTILSDKTGTLTCNSMEFIKCSIAGTAYGRGVTEVERAIYRKKGSPVVHEPNGLNHIEDSADVNPAIKGFNFKDERIMNGNTGHDTKVMQNATDPPSKRSKIEKTMDRVIYLMFFIVFIMGFIGSIFFGIATENDYEGGRIKRRWYLRPDNAEIFFDPERAPVAAIYHFLTALLLYSYFIPISLYVSIEIVKVLQSIFINQDSHMYYEEADKPAHARTSNLNEELGQVDTILSDKTGTLTCNSMEFIKCSIAGTAYGRGVTEVERAIYRKKGSPVVHEPNGLNHIEDSADVNPAIKGFNFKDERIMNGNWVNEPRADVIQKFFRLLAICHTAIPEVDEENGNISYEAESPDEAAFVIAARVLGFEFHNRTQTSISLHELDPVSGKRVNRLFKLLNVLEFDSTRKRMSVIVRDEEGKLLLLCKGADSVMFERLAKGGRDFEEDTREHMNEYADAGLRTLVLAYRELSENEYEVFNEKMTEAKNSVSADRETLIDEVAEMIERDLILLGATAVEDKLQNGVPDCIDKLAQAGIKLWVLTGDKMETAINIGYACSLLRQGMKQIIINIDTPEIQSLEKTGDKDAVIKASRKSVMEQIVSGKAQVSALSAISEAFALIIDGKSLAYALEDDMKNIFLELAIGCASVICCRSSPKQKALVTRLVKLGTGKTTLAIGDGANDVGMLQEADIGIGISGVEGMQAVMSSDVAIAQFRYLERLLLVHGHWCYRRISSMICYFFYKNIAFGFTIFLYEAYTSFSAQPAYNDWYLTLFNVFFSSLPVIAMGVFDQDVSARFCLQFPLLYQEGVQNVLFSWRRIVSWMFNGFYSAIIIFFFCSRALEQQAFNDEGKIASKDILGGTMYTCIVWVVNLQMALSISYFTLIQHIVIWGTIAFWYVFQLAYGALPASFSTDAYRVFVEALAPAPSYWFITLFVVIATLTPYFLYSAIQMRFFPMYHEMIQWIRHEGLSDDPLYCEMVRQRSIRPTTVGFTARRHHYTAGETHVQIQYLHGRILQRDNASILI

>GbALA9

MSGGRRRKVLMSRIYGVACGKASFKEDHSQIGGPGFSRIVYCNEPNSLEAGTRNYSDNYVSTTKYTVATFLPKSLFEQFRRVANFFFLVTGILSFTAIAPYSALSAIVPLIIVIGATMIKEGVEDWRRQQQDIEVNNRKVKVHQGDGNFHHTEWKNLRVGDIVKVEKDEFFPTDLILLASSYEDAVCYVETMNLDGETNLKLKQALEVTSSLHDDYNFRDFKAIVKCEDPNANLYSFVGTMEFEEQQHPLSPQQLLLRDSKLRNTDYIYGAVVFTGHDTKVMQNATDPPSKRSKIEKTMDRVIYLMFFIVFIMGFVGSIFFGIATENDYEGGRIKRRWYLRPDNAEIFFDPERAPVAAIYHFLTALLLYSYFIPISLYVSIEIVKVLQSIFINQDSHMYYEEADKPAHARTSNLNEELGQVDTILSDKTGTLTCNSMEFIKCSIAGTAYGRGVTEVERAIYRKKGSPVVHEPNGLNHIEDSADANPAIKGFNFKDERIMNGNWVNEPRADVIQKFFRLLAICHTAIPEVDEENGNISYEAESPDEAAFVIAARVLGFEFHNRTQTSISLHELDPVSGKRVNRLYKLLNVLEFDSTRKRMSVIVRDEEGKLLLLCKGADSVMFERLAKGGRDFEEDTREHMNEYADAGLRTLVLAYRELSENEYEVFNEKMTEAKNSVSADRETLIDGVAEMIERDLILLGATAVEDKLQNGVPDCIDKLAQAGIKLWVLTGDKMETAINIGYACSLLRQGMKQIIINLDTPEIQSLEKTGDKDAVIKASRKSVMEQIVSGKSQVSALSAISEAFALIIDGKSLAYALEDDMKNIFLELAIGCASVICCRSSPKQKALVTRLVKLGTGKTTLAIGDGANDVGMLQEADIGIGISGVEGMQAVMSSDVAIAQFRYLERLLLVHGHWCYRRISSMICYFFYKNIAFGFTIFLYEAYTSFSAQPAYNDWYLTLFNVFFSSLPVIAMGVFDQDVSARFCLKFPLLYQEGVQNVLFSWRRIVSWMFNGFYSAIIIFFFCSRALEQQAFNDEGKIASKDILGGTMYTCIVWVVNLQMALSISYFTLIQHIVIWGTIAFWYVFQLAYGALPASFSTDAYRVFVEALAPAPSYWFITLFVVIATLTPYFLYSAIQMRFFPMYHEMIQWIRHEGLSDDPLYCEMVRQRSIRPTTVGFTARRAASRRQ

>GbP51

MSRFHVGGKVVDKVDLLRKKHAAWRLDVWPFAMLYLLWLTMVVPSIDFVDAAIVLGGLAVTHILVLLFTTWSVDFKCFVQYSKVNNIRLADVCKVTPAKFCGSKEVVPLHIRKQATSSSSAKDVEEIYFDFRKQCFIYSKEEDTFCKLPYPTKETFGYYLKCSGHGSDAKVLAATEKWGRNVFEYPQPTFQKLMKEHCMEPFFVFQVFCVGLWCLDEYWYYSLFTLFMLFMFESTMAKSRLKTLSELRRVRVDSQTLMVHRCGKWVKLSGTDLLPGDVVSIGRSSGQNEEDKSVPADMLILAGSAIVNEAILTGESTPQWKVSIAGRGIEEKLSAKRDKNHMLFGGTKILQHTADKSFPVRTPDGGCLAVVLRTGFETSQGKLMRTILFSTERVTANSWESGLFILFLVVFAIIAAGYVLKKGLEDPTRSKYKLFLSCSLIITSVIPPELPMELSIAVNTSLIALARRGIFCTEPFRIPFAGKVDICCFDKTGTLTSDDMEFSGVVGLNDSSELESDMTKVPSRTVEILASCHALVFVDNKLVGDPLEKAALKGIDWSYKSDEKAIPKKGSGNPVQIAQRHHFASHLKRMAVVVRVQEDFFSFVKGAPETIQDRLIDLPPTYVETYKKYTRQGSRVLALAYKSLPDMTVSEARSMERDTVECGLTFAGFAGAPETIQDRLIDLPPTYVETYKKYTRQGSRVLALAYKSLPDMTVSEARSMERDTVECGLTFAGFAVFNCPIRADSLTVLSELKNSSHDLVMITGDQALTACHVAGQVNIVSKPALILVAVKNSKGYEWVSPDETERIPYSENEVEALSETHDLCIGGDCFEMLQQTSAVLRVIPFVKVFARVAPEQKELIMTTFKTVGRLTLMCGDGTNDVGALKQAHVGVALLNAVPPTKSESSSGTSKDENTKSLKSKKSKPTVEATGNSEASSKGKVVPRSESSNNATSNRHLNAAEKHRQKLKKMMNELNEEGDGRSAPIVKLGDASMASPFTAKHASVAPTTDIIRQGRSTLVTTLQMFKILGLNCLATAYVLSVMYLDGVKLGDVQATISGVFTAAFFLFISHARPLPTLSAARPHPNIFCSYVFLSLMGQFAMHLLFLISSVKEAEKHMPEECIEPESEFHPNLVNTVSYMVSMMLQVATFAVNYMGHPFNQSIPENKPFLYALGAAAGFFVVITSDLFRDLNDWLSLVPLPVGLRDKLLLWALLMFLWCYAWERLLRWAFPGKIPAWRKRQRVAAASSEKKLV

>GbP52

MSRFHVDGKVVDKVDLLRKKHAAWRLDVWPFAMLYLLWLTMVVPSIDFVDAAIVLGGLAVTHILVLLFTTWSVDFKCFVQYSKVNNIRLADVCKVTPAKFCGSKEVVPLHIRKQIASSSSAKDVEEIYFDFRKQCFIYSEEEDTFCKLPYPTKETFGYYLKCSGHGSDAKVLAATEKWGRNVFEYPQPTFQKLMKEHCMEPFFVFQVFCVGLWCLDEYWYYSLFTLFMLFMFESTMAKSRLKTLSELRRVRVDSQTLMVHRCGKWVKLSGTDLLPGDVVSIGRSSGQNEEDKSVPADMLILAGSAIVNEAILTGESTPQWKVSIAGRGIEEKLSAKRDKNHMLFGGTKILQHTADKSFPLRTPDGGCLAVVLRTGFETSQGKLMRTILFSTERVTANSWESGLFILFLVVFAIIAAGYVLKKGLEDPTRSKYKLFLSCSLIITSVIPPELPMELSIAVNTSLIALARRGIFCTEPFRIPFAGKVDICCFDKTGTLTSDDMEFSGVVGLNDSSELESDMTKVPSRTVEILASCHALVFVDNKLVGDPLEKAALKGIDWSYKSDEKAIPKKGSGNPVQIVQRHHFASHLKRMAVVVRVQEDFFAFVKGAPETIQDRLIDLPPTYVETYKKYTRQGSRVLALAYKSLPDMTVSEARSMERDTVECGLTFAGFAVFNCPIRADSSTVLSELKNSSHDLVMITGDQALTACHVAGQVNIVSKPALILVAVKNSKGYEWVSPDETERIPYSENEVEALSETHDLCIGGDCFEMLQQTSAVLRVIPFVKVFARVAPEQKELIMTTFKTVGRLTLMCGDGTNDVGALKQAHVGVALLNAVPPTKSESSSGTSKDENTKALKSKKSKPTVEATGNSEASSKGKVVPRSESSNNATSNRHLNAAEKHRQKLKKMMNELNEEGDGRSAPIVKLGDASMASPFTAKHASVAPTTDIIRQGRSTLVTTLQMFKILGLNCLATAYVLSVMYLDGVKLGDVQATISGVFTAAFFLFISHARPLPTLSAARPHPNIFCSYVFLSLMGQFAMHLLFLISSVKEAEKHMPEECIEPESEFHPNLVNTVSYMVSMMLQVATFAVNYMGHPFNQSIPENKPFLYALGAAAGFFVVITSDLFRDLNDWLSLVPLPVGLRDKLLLWALLMFLCCYAWERLLRWAFPGKIPAWRKRQRVAAASSEKKLV

>AtAHA1

MSGLEDIKNETVDLEKIPIEEVFQQLKCTREGLTTQEGEDRIVIFGPNKLEEKKESKILKFLGFMWNPLSWVMEAAALMAIALANGDNRPPDWQDFVGIICLLVINSTISFIEENNAGNAAAALMAGLAPKTKVLRDGKWSEQEAAILVPGDIVSIKLGDIIPADARLLEGDPLKVDQSALTGESLPVTKHPGQEVFSGSTCKQGEIEAVVIATGVHTFFGKAAHLVDSTNQVGHFQKVLTSIGNFCICSIAIGIAIEIVVMYPIQHRKYRDGIDNLLVLLIGGIPIAMPTVLSVTMAIGSHRLSQQGAITKRMTAIEEMAGMDVLCSDKTGTLTLNKLSVDKNLVEVFCKGVEKDQVLLFAAMASRVENQDAIDAAMVGMLADPKEARAGIREVHFLPFNPVDKRTALTYIDSDGNWHRVSKGAPEQILDLANARPDLRKKVLSCIDKYAERGLRSLAVARQVVPEKTKESPGGPWEFVGLLPLFDPPRHDSAETIRRALNLGVNVKMITGDQLAIGKETGRRLGMGTNMYPSAALLGTDKDSNIASIPVEELIEKADGFAGVFPEHKYEIVKKLQERKHIVGMTGDGVNDAPALKKADIGIAVADATDAARGASDIVLTEPGLSVIISAVLTSRAIFQRMKNYTIYAVSITIRIVFGFMLIALIWEFDFSAFMVLIIAILNDGTIMTISKDRVKPSPTPDSWKLKEIFATGIVLGGYQAIMSVIFFWAAHKTDFFSDKFGVRSIRDNNDELMGAVYLQVSIISQALIFVTRSRSWSFVERPGALLMIAFVIAQLVATLIAVYADWTFAKVKGIGWGWAGVIWIYSIVTYFPQDILKFAIRYILSGKAWASLFDNRTAFTTKKDYGIGEREAQWAQAQRTLHGLQPKEDVNIFPEKGSYRELSEIAEQAKRRAEIARLRELHTLKGHVESVAKLKGLDIDTAGHHYTV

>AtAHA2

MSSLEDIKNETVDLEKIPIEEVFQQLKCSREGLTTQEGEDRIQIFGPNKLEEKKESKLLKFLGFMWNPLSWVMEMAAIMAIALANGDGRPPDWQDFVGIICLLVINSTISFIEENNAGNAAAALMAGLAPKTKVLRDGKWSEQEAAILVPGDIVSIKLGDIIPADARLLEGDPLKVDQSALTGESLPVTKHPGQEVFSGSTCKQGEIEAVVIATGVHTFFGKAAHLVDSTNQVGHFQKVLTAIGNFCICSIAIGMVIEIIVMYPIQRRKYRDGIDNLLVLLIGGIPIAMPTVLSVTMAIGSHRLSQQGAITKRMTAIEEMAGMDVLCSDKTGTLTLNKLSVDKNLVEVFCKGVEKDQVLLFAAMASRVENQDAIDAAMVGMLADPKEARAGIREVHFLPFNPVDKRTALTYIDGSGNWHRVSKGAPEQILELAKASNDLSKKVLSIIDKYAERGLRSLAVARQVVPEKTKESPGAPWEFVGLLPLFDPPRHDSAETIRRALNLGVNVKMITGDQLAIGKETGRRLGMGTNMYPSSALLGTHKDANLASIPVEELIEKADGFAGVFPGYNLLIYCLDYKPHYMFIAKVVMLVLSFVFFIAEHKYEIVKKLQERKHIVGMTGDGVNDAPALKKADIGIAVADATDAARGASDIVLTEPGLSVIISAVLTSRAIFQRMKNYTIYAVSITIRIVFGFMLIALIWEFDFSAFMVLIIAILNDGTIMTISKDRVKPSPTPDSWKLKEIFATGVVLGGYQAIMTVIFFWAAHKTDFFSDTFGVRSIRDNNHELMGAVYLQVSIISQALIFVTRSRSWSFVERPGALLMIAFLIAQLIATLIAVYANWEFAKIRGIGWGWAGVIWLYSIVTYFPLDVFKFAIRYILSGKAWLNLFENKTAFTMKKDYGKEEREAQWALAQRTLHGLQPKEAVNIFPEKGSYRELSEIAEQAKRRAEIARLRELHTLKGHVESVVKLKGLDIETPSHYTV

>AtAHA3

MASGLEDIVNENVDLEKIPIEEVFQQLKCSREGLSGAEGENRLQIFGPNKLEEKKESKLLKFLGFMWNPLSWVMEAAAIMAIALANGGGKPPDWQDFVGIVCLLVINSTISFVEENNAGNAAAALMAGLAPKTKVLRDGKWSEQEASILVPGDIVSIKLGDIIPADARLLEGDPLKVDQSALTGESLPATKGPGEEVFSGSTCKQGEIEAVVIATGVHTFFGKAAHLVDSTNQVGHFQKVLTAIGNFCICSIAVGIAIEIVVMYPIQRRHYRDGIDNLLVLLIGGIPIAMPTVLSVTMAIGSHKLSQQGAITKRMTAIEEMAGMDVLCSDKTGTLTLNKLSVDKNLIEVYCKGVEKDEVLLFAARASRVENQDAIDAAMVGMLADPKEARAGIREIHFLPFNPVDKRTALTFIDSNGNWHRVSKGAPEQILDLCNARADLRKRVHSTIDKYAERGLRSLAVSRQTVPEKTKESSGSPWEFVGVLPLFDPPRHDSAETIRRALDLGVNVKMITGDQLAIAKETGRRLGMGSNMYPSSSLLGKHKDEAMAHIPVEDLIEKADGFAGVFPEHKYEIVKKLQERKHICGMTGDGVNDAPALKKADIGIAVADATDAARGASDIVLTEPGLSVIISAVLTSRAIFQRMKNYTIYAVSITIRIVFGFMLIALIWKFDFSPFMVLIIAILNDGTIMTISKDRVKPSPTPDSWKLKEIFATGVVLGGYMAIMTVVFFWAAYKTDFFPRTFHVRDLRGSEHEMMSALYLQVSIVSQALIFVTRSRSWSFTERPGYFLLIAFWVAQLIATAIAVYGNWEFARIKGIGWGWAGVIWLYSIVFYFPLDIMKFAIRYILAGTAWKNIIDNRTAFTTKQNYGIEEREAQWAHAQRTLHGLQNTETANVVPERGGYRELSEIANQAKRRAEIARLRELHTLKGHVESVVKLKGLDIETAGHYTV

>AtAHA4

MTTTVEDNREVLEAVLKEAVDLENVPIEEVFENLRCSKEGLTTQAADERLALFGHNKLEEKKESKFLKFLGFMWNPLSWVMEAAAIMAIALANGGGKPPDWQDFVGIITLLVINSTISFIEENNAGNAAAALMARLAPKAKVLRDGRWGEQDAAILVPGDIISIKLGDIVPADARLLEGDPLKIDQSALTGESLPVTKSSGDGVYSGSTCKQGEIEAVVIATGVHTFFGKAAHLVDTTNQIGHFQQVLTAIGNFCICSIAVGMLIEIVVMYPIQHRAYRPGIDNLLVLLIGGIPIAMPTVLSVTMAIGSHRLSQQGAITKRMTAIEEMAGMDVLCSDKTGTLTLNKLTVDKNLIEVFMKGVDADTVVLMAARASRLENQDAIDAAIVGMLADPKDARAGIQEVHFLPFNPTDKRTALTYIDNEGNTHRVSKGAPEQILNLAHNKSEIERRVHAVIDKFAERGLRSLAVAYQDVPEGRKDSAGGPWQFVGLMPLFDPPRHDSAETIRRALNLGVSVKMITGDQLAIGKETGRRLGMGTNMYPSSALLGQNKDESIVALPVDELIEKADGFAGVFPEHKYEIVKRLQARKHICGMTGDGVNDAPALKKADIGIAVADATDAARSASDIVLTEPGLSVIISAVLTSRAIFQRMKNYTIYAVSITIRIVLGFMLLALIWQFDFPPFMVLIIAILNDGTIMTISKDRVKPSPLPDSWKLSEIFATGVVFGSYMAMMTVIFFWVSYKTDFFPRTFGVATLEKTAHDDFRKLASAIYLQVSIISQALIFVTRSRSWSFVERPGIFLMIAFILAQLVATLIAVYANWSFAAIEGIGWGWAGVIWLYNIIFYIPLDFIKFFIRYALSGRAWDLVIEQRVAFTRQKDFGKEQRELQWAHAQRTLHGLQAPDTKMFTDRTHVSELNQMAEEAKRRAEIARLRELHTLKGHVESVVRLKGLDIETIQQAYTV

>AtAHA5

MSELDHIKNESVDLVRIPMEEVFEELKCTKQGLTANEASHRLDVFGPNKLEEKKESKLLKFLGFMWNPLSWVMEVAALMAIALANGGGRPPDWQDFVGIVCLLLINSTISFIEENNAGNAAAALMAGLAPKTKVLRDNQWSEQEASILVPGDVISIKLGDIIPADARLLDGDPLKIDQSSLTGESIPVTKNPSDEVFSGSICKQGEIEAIVIATGVHTFFGKAAHLVDNTNQIGHFQKVLTSIGNFCICSIALGIIVELLVMYPIQRRRYRDGIDNLLVLLIGGIPIAMPSVLSVTMATGSHRLFQQGAITKRMTAIEEMAGMDVLCCDKTGTLTLNKLTVDKNLVEVFAKGVGKEHVFLLAARASRIENQDAIDAAIVGMLADPKEARAGVREVHFFPFNPVDKRTALTYVDSDGNWHRASKGAPEQILNLCNCKEDVRRKVHGVIDKFAERGLRSLAVARQEVLEKKKDAPGGPWQLVGLLPLFDPPRHDSAETIRRALNLGVNVKMITGDQLAIGKETGRRLGMGTNMYPSSALLGQVKDSSLGALPVDELIEKADGFAGVFPEHKYEIVHRLQQRNHICGMTGDGVNDAPALKKADIGIAVVDATDAARGASDIVLTEPGLSVIISAVLTSRAIFQRMKNYTIYAVSITIRIVFGFMFIALIWQFDFSPFMVLIIAILNDGTIMTISKDRMKPSPQPDSWKLRDIFSTGVVLGGYQALMTVVFFWVMKDSDFFSNYFGVRPLSQRPEQMMAALYLQVSIISQALIFVTRSRSWSYAECPGLLLLGAFVIAQLVATFIAVYANWSFARIEGAGWGWAGVIWLYSFLTYIPLDLLKFGIRYVLSGKAWLNLLENKTAFTTKKDYGKEEREAQWAAAQRTLHGLQPAEKNNIFNEKNSYSELSQIAEQAKRRAEVVRLREINTLKGHVESVVKLKGLDIDTIQQHYTV

>AtAHA6

MAADISWDEIKKENVDLEKIPVDEVFQQLKCSREGLSSEEGRNRLQIFGANKLEEKVENKFLKFLGFMWNPLSWVMEAAAIMAIVLANGGGRPPDWQDFVGITCLLIINSTISFIEENNAGNAAAALMANLAPKTKVLRDGRWGEQEAAILVPGDLISIKLGDIVPADARLLEGDPLKIDQSALTGESLPATKHQGDEVFSGSTCKQGEIEAVVIATGVHTFFGKAAHLVDSTNNVGHFQKVLTAIGNFCICSIGIGMLIEIIIMYPIQHRKYRDGIDNLLVLLIGGIPIAMPTVLSVTMAIGSHRLSQQGAITKRMTAIEEMAGMDVLCSDKTGTLTLNKLTVDKNLIEVFSKDVDKDYVILLSARASRVENQDAIDTSIVNMLGDPKEARAGITEVHFLPFNPVEKRTAITYIDTNGEWHRCSKGAPEQIIELCDLKGETKRRAHEIIDKFAERGLRSLGVARQRVPEKDKESAGTPWEFVGLLPLFDPPRHDSAETIRRALDLGVNVKMITGDQLAIGKETGRRLGMGTNMYPSSSLLENKDDTTGGVPVDELIEKADGFAGVFPEHKYEIVRKLQERKHIVGMTGDGVNDAPALKKADIGIAVDDATDAARSASDIVLTEPGLSVIVSAVLTSRAIFQRMKNYTIYAVSITIRIVLGFMLVALIWEFDFSPFMVLIIAILNDGTIMTISKDRVKPSPIPDSWKLKEIFATGVVLGTYMALVTVVFFWLAHDTTFFSDKFGVRSLQGKDEELIAVLYLQVSIISQALIFVTRSRSWSFVERPGLLLLIAFFVAQLIATLIATYAHWEFARIKGCGWGWCGVIWIYSIVTYIPLDILKFITRYTLSGKAWNNMIENRTAFTTKKDYGRGEREAQWALAQRTLHGLKPPESMFEDTATYTELSEIAEQAKKRAEVARLREVHTLKGHVESVVKLKGLDIDNLNQHYTV

>AtAHA7

MTDIEALKAITTESIDLENVPVEEVFQHLKCTKEGLTSNEVQERLTLFGYNKLEEKKESKILKFLGFMWNPLSWVMEAAALMAIGLAHGGGKPADYHDFVGIVVLLLINSTISFVEENNAGNAAAALMAQLAPKAKAVRDGKWNEIDAAELVPGDIVSIKLGDIIPADARLLEGDPLKIDQATLTGESLPVTKNPGASVYSGSTCKQGEIEAVVIATGVHTFFGKAAHLVDSTTHVGHFQKVLTAIGNFCICSIAVGMAIEIVVIYGLQKRGYRVGIDNLLVLLIGGIPIAMPTVLSVTMAIGAHRLAQQGAITKRMTAIEEMAGMDVLCSDKTGTLTLNKLSVDKNLIEVFKRGIDRDMAVLMAARAARLENQDAIDTAIVSMLSDPKEARAGIKELHFLPFSPANRRTALTYLDGEGKMHRVSKGAPEEILDMAHNKLEIKEKVHATIDKFAERGLRSLGLAYQEVPDGDVKGEGGPWDFVALLPLFDPPRHDSAQTIERALHLGVSVKMITGDQLAIAKETGRRLGMGTNMYPSSSLLSDNNTEGVSVDELIENADGFAGVFPEHKYEIVKRLQSRKHICGMTGDGVNDAPALKKADIGIAVDDATDAARGASDIVLTEPGLSVIISAVLTSRAIFQRMKNYTIYAVSITIRIVMGFMLLCVFWEFDFPPFMVLVIAILNDGTIMTISKDRVKPSPTPDCWKLKEIFATGVVLGAYLAIMTVVFFWAAYETNFFHNIFHVRNFNQHHFKMKDKKVAAHLNEQMASAVYLQVSTISQALIFVTRSRSWSFVERPGFLLVIAFLIAQLVASVISAMANWPFAGIRSIGWGWTGVIWIFNIVTYMLLDPIKFLVRYALSGKSWDRMVEGRTALTGKKNFGQEERMAAWATEKRTQHGLETGQKPVYERNSATELNNMAEEAKRRAEIARMRELQTLKGKVESAAKLKGYDLEDPNSNNYTI

>AtAHA8

MATEFSWDEIKKENVDLERIPVEEVFEQLKCSKEGLSSDEGAKRLEIFGANKLEEKSENKFLKFLGFMWNPLSWVMESAAIMAIVLANGGGKAPDWQDFIGIMVLLIINSTISFIEENNAGNAAAALMANLAPKTKVLRDGKWGEQEASILVPGDLISIKLGDIVPADARLLEGDPLKIDQSALTGESLPTTKHPGDEVFSGSTCKQGEIEAVVIATGVHTFFGKAAHLVDSTNNVGHFQKVLTSIGNFCICSIGLGMLIEILIMYPIQHRTYRDGIDNLLVLLIGGIPIAMPTVLSVTMAIGSHRLSQQGAITKRMTAIEEMAGMDVLCSDKTGTLTLNKLSVDKSLIEVFPKNMDSDSVVLMAARASRIENQDAIDASIVGMLGDPKEARAGITEVHFLPFNPVDKRTAITYIDESGDWHRSSKGAPEQIIELCNLQGETKRKAHEVIDGFAERGLRSLGVAQQTVPEKTKESDGSPWEFVGLLPLFDPPRHDSAETIRRALELGVNVKMITGDQLAIGIETGRRLGMGTNMYPSTSLLGNSKDESLVGIPIDELIEKADGFAGVFPEHKYEIVKKLQERKHICGMTGDGVNDAPALKKADIGIAVADATDAARSASDIVLTEPGLSVIISAVLTSRAIFQRMKNYTIYAVSITIRIVLGFMLVALIWRFDFAPFMVLIIAILNDGTIMTISKDRVKPSPVPDSWKLNEIFATGVVLGTYMALTTVLFFWLAHDTDFFSKTFGVRSIQGNEEELMAALYLQVSIISQALIFVTRSRSWSFVERPGFLLLIAFVIAQLVATLIAVYANWGFARIVGCGWGWAGGIWVYSIITYIPLDILKFIIRYALTGKAWDNMINQKTAFTTKKDYGKGEREAQWALAQRTLHGLPPPEAMFNDNKNELSEIAEQAKRRAEVARLRELHTLKGHVESVVKLKGLDIDTIQQHYTV

>AtAHA9

MAGNKDSSWDDIKNEGIDLEKIPIEEVLTQLRCTREGLTSDEGQTRLEIFGPNKLEEKKENKVLKFLGFMWNPLSWVMELAAIMAIALANGGGRPPDWQDFVGITVLLIINSTISFIEENNAGNAAAALMAGLAPKTKVLRDGKWSEQEAAILVPGDIISIKLGDIVPADGRLLDGDPLKIDQSALTGESLPVTKHPGQEVYSGSTCKQGELEAVVIATGVHTFFGKAAHLVDSTNQEGHFQKVLTAIGNFCICSIAIGMLIEIVVMYPIQKRAYRDGIDNLLVLLIGGIPIAMPTVLSVTMAIGSHRLSQQGAITKRMTAIEEMAGMDVLCSDKTGTLTLNKLTVDKSMVEVFVKDLDKDQLLVNAARASRVENQDAIDACIVGMLGDPREAREGITEVHFFPFNPVDKRTAITYIDANGNWHRVSKGAPEQIIELCNLREDASKRAHDIIDKFADRGLRSLAVGRQTVSEKDKNSPGEPWQFLGLLPLFDPPRHDSAETIRRALDLGVNVKMITGDQLAIGKETGRRLGMGTNMYPSSALLGQDKDESIASLPVDELIEKADGFAGVFPEHKYEIVKRLQEMKHICGMTGDGVNDAPALKRADIGIAVADATDAARSASDIVLTEPGLSVIVSAVLTSRAIFQRMKNYTIYAVSITIRIVMGFMLLALIWKFDFSPFMVLIVAILNDGTIMTISKDRVKPSPLPDSWKLKEIFATGVVLGTYLAVMTVVFFWAAESTDFFSAKFGVRSISGNPHELTAAVYLQVSIVSQALIFVTRSRSWSYVERPGFWLISAFFMAQLIATLIAVYANWNFARIRGIGWGWAGVIWLYSIVFYIPLDILKFIIRYSLSGRAWDNVIENKTAFTSKKDYGKGEREAQWAQAQRTLHGLQPAQTSDMFNDKSTYRELSEIADQAKRRAEVARLRERHTLKGHVESVVKQKGLDIEAIQQHYTL

>AtAHA10

MAEDLDKPLLDPDTFNRKGIDLGILPLEEVFEYLRTSPQGLLSGDAEERLKIFGPNRLEEKQENRFVKFLGFMWNPLSWVMEAAALMAIALANSQSLGPDWEDFTGIVCLLLINATISFFEENNAGNAAAALMARLALKTRVLRDGQWQEQDASILVPGDIISIKLGDIIPADARLLEGDPLKIDQSVLTGESLPVTKKKGEQVFSGSTCKQGEIEAVVIATGSTTFFGKTARLVDSTDVTGHFQQVLTSIGNFCICSIAVGMVLEIIIMFPVQHRSYRIGINNLLVLLIGGIPIAMPTVLSVTLAIGSHRLSQQGAITKRMTAIEEMAGMDVLCCDKTGTLTLNSLTVDKNLIEVFVDYMDKDTILLLAGRASRLENQDAIDAAIVSMLADPREARANIREIHFLPFNPVDKRTAITYIDSDGKWYRATKGAPEQVLNLCQQKNEIAQRVYAIIDRFAEKGLRSLAVAYQEIPEKSNNSPGGPWRFCGLLPLFDPPRHDSGETILRALSLGVCVKMITGDQLAIAKETGRRLGMGTNMYPSSSLLGHNNDEHEAIPVDELIEMADGFAGVFPEHKYEIVKILQEMKHVVGMTGDGVNDAPALKKADIGIAVADATDAARSSADIVLTDPGLSVIISAVLTSRAIFQRMRNYTVYAVSITIRIVLGFTLLALIWEYDFPPFMVLIIAILNDGTIMTISKDRVRPSPTPESWKLNQIFATGIVIGTYLALVTVLFYWIIVSTTFFEKHFHVKSIANNSEQVSSAMYLQVSIISQALIFVTRSRGWSFFERPGTLLIFAFILAQLAATLIAVYANISFAKITGIGWRWAGVIWLYSLIFYIPLDVIKFVFHYALSGEAWNLVLDRKTAFTYKKDYGKDDGSPNVTISQRSRSAEELRGSRSRASWIAEQTRRRAEIARLLEVHSVSRHLESVIKLKQIDQRMIRAAHTV

>AtAHA11

MGDKEEVLEAVLKETVDLENVPIEEVFESLRCSREGLTTEAADERLALFGHNKLEEKKESKFLKFLGFMWNPLSWVMEAAAIMAIALANGGGKPPDWQDFVGIITLLVINSTISFIEENNAGNAAAALMARLAPKAKVLRDGRWGEQDAAILVPGDIISIKLGDIVPADARLLEGDPLKIDQSSLTGESLPVTKGPGDGVYSGSTCKQGELEAVVIATGVHTFFGKAAHLVDTTNHVGHFQQVLTAIGNFCICSIAVGMIIEIVVMYPIQHRAYRPGIDNLLVLLIGGIPIAMPTVLSVTMAIGSHRLSQQGAITKRMTAIEEMAGMDVLCSDKTGTLTLNKLTVDKNLIEVFTKGVDADTVVLMAAQASRLENQDAIDAAIVGMLADPKEARAGVREVHFLPFNPTDKRTALTYIDSDGKMHRVSKGAPEQILNLAHNRAEIERRVHAVIDKFAERGLRSLAVAYQEVPEGTKESAGGPWQFMGLMPLFDPPRHDSAETIRRALNLGVNVKMITGDQLAIGKETGRRLGMGTNMYPSSALLGQHKDESIGALPIDDLIEKADGFAGVFPEHKYEIVKRLQARKHICGMTGDGVNDAPALKKADIGIAVADATDAARSASDIVLTEPGLSVIISAVLTSRAIFQRMKNYTIYAVSITIRIVLGFMLLALIWKFDFPPFMVLIIAILNDGTIMTISKDRVKPSPLPDSWKLSEIFATGVVFGSYMAMMTVIFFWAAYKTDFFPRTFGVSTLEKTAHDDFRKLASAIYLQVSIISQALIFVTRSRSWSYVERPGMLLVVAFILAQLVATLIAVYANWSFAAIEGIGWGWAGVIWLYNIVFYIPLDIIKFLIRYALSGRAWDLVIEQRVAFTRQKDFGKEQRELQWAHAQRTLHGLQAPDAKMFPERTHFNELSQMAEEAKRRAEIARLRELHTLKGHVESVVRLKGLDIETIQQAYTV

>AtHMA1

MEPATLTRSSSLTRFPYRRGLSTLRLARVNSFSILPPKTLLRQKPLRISASLNLPPRSIRLRAVEDHHHDHHHDDEQDHHNHHHHHHQHGCCSVELKAESKPQKMLFGFAKAIGWVRLANYLREHLHLCCSAAAMFLAAAVCPYLAPEPYIKSLQNAFMIVGFPLVGVSASLDALMDIAGGKVNIHVLMALAAFASVFMGNALEGGLLLAMFNLAHIAEEFFTSRSMVDVKELKESNPDSALLIEVHNGNVPNISDLSYKSVPVHSVEVGSYVLVGTGEIVPVDCEVYQGSATITIEHLTGEVKPLEAKAGDRVPGGARNLDGRMIVKATKAWNDSTLNKIVQLTEEAHSNKPKLQRWLDEFGENYSKVVVVLSLAIAFLGPFLFKWPFLSTAACRGSVYRALGLMVAASPCALAVAPLAYATAISSCARKGILLKGAQVLDALASCHTIAFDKTGTLTTGGLTCKAIEPIYGHQGGTNSSVITCCIPNCEKEALAVAAAMEKGTTHPIGRAVVDHSVGKDLPSIFVESFEYFPGRGLTATVNGVKTVAEESRLRKASLGSIEFITSLFKSEDESKQIKDAVNASSYGKDFVHAALSVDQKVTLIHLEDQPRPGVSGVIAELKSWARLRVMMLTGDHDSSAWRVANAVGITEVYCNLKPEDKLNHVKNIAREAGGGLIMVGEGINDAPALAAATVGIVLAQRASATAIAVADILLLRDNITGVPFCVAKSRQTTSLVKQNVALALTSIFLAALPSVLGFVPLWLTVLLHEGGTLLVCLNSVRGLNDPSWSWKQDIVHLINKLRSQEPTSSSSNSLSSAH

>AtHMA2

MASKKMTKSYFDVLGICCTSEVPLIENILNSMDGVKEFSVIVPSRTVIVVHDTLILSQFQIVKALNQAQLEANVRVTGETNFKNKWPSPFAVVSGILLLLSFFKYLYSPFRWLAVAAVVAGIYPILAKAVASLARFRIDINILVVVTVGATIGMQDYTEAAVVVFLFTIAEWLQSRASYKASAVMQSLMSLAPQKAVIAETGEEVEVDELKTNTVIAVKAGETIPIDGVVVDGNCEVDEKTLTGEAFPVPKLKDSTVWAGTINLNGYITVNTTALAEDCVVAKMAKLVEEAQNSKTETQRFIDKCSKYYTPAIILISICFVAIPFALKVHNLKHWVHLALVVLVSACPCGLILSTPVATFCALTKAATSGLLIKGADYLETLAKIKIVAFDKTGTITRGEFIVMDFQSLSEDISLQSLLYWVSSTESKSSHPMAAAVVDYARSVSVEPKPEAVEDYQNFPGEGIYGKIDGKEVYIGNKRIASRAGCLSVPDIDVDTKGGKTIGYVYVGETLAGVFNLSDACRSGVAQAMKELKSLGIKIAMLTGDNHAAAMHAQEQLGNAMDIVRAELLPEDKSEIIKQLKREEGPTAMVGDGLNDAPALATADIGISMGVSGSALATETGNIILMSNDIRRIPQAIKLAKRAKRKVVENVVISITMKGAILALAFAGHPLIWAAVLADVGTCLLVILNSMLLLSDKHKTGNKCYRESSSSSVLIAEKLEGDAAGDMEAGLLPKISDKHCKPGCCGTKTQEKAMKPAKASSDHSHSGCCETKQKDNVTVVKKSCCAEPVDLGHGHDSGCCGDKSQQPHQHEVQVQQSCHNKPSGLDSGCCGGKSQQPHQHELQQSCHDKPSGLDIGTGPKHEGSSTLVNLEGDAKEELKVLVNGFCSSPADLAITSLKVKSDSHCKSNCSSRERCHHGSNCCRSYAKESCSHDHHHTRAHGVGTLKEIVIE

>AtHMA3

MAEGEESKKMNLQTSYFDVVGICCSSEVSIVGNVLRQVDGVKEFSVIVPSRTVIVVHDTFLISPLQIVKALNQARLEASVRPYGETSLKSQWPSPFAIVSGVLLVLSFFKYFYSPLEWLAIVAVVAGVFPILAKAVASVTRFRLDINALTLIAVIATLCMQDFTEAATIVFLFSVADWLESSAAHKASIVMSSLMSLAPRKAVIADTGLEVDVDEVGINTVVSVKAGESIPIDGVVVDGSCDVDEKTLTGESFPVSKQRESTVMAATINLNGYIKVKTTALARDCVVAKMTKLVEEAQKSQTKTQRFIDKCSRYYTPAVVVSAACFAVIPVLLKVQDLSHWFHLALVVLVSGCPCGLILSTPVATFCALTKAATSGFLIKTGDCLETLAKIKIVAFDKTGTITKAEFMVSDFRSLSPSINLHKLLYWVSSIECKSSHPMAAALIDYARSVSVEPKPDIVENFQNFPGEGVYGRIDGQDIYIGNKRIAQRAGCLTDNVPDIEATMKRGKTIGYIYMGAKLTGSFNLLDGCRYGVAQALKELKS

>AtHMA4

MALQNKEEEKKKVKKLQKSYFDVLGICCTSEVPIIENILKSLDGVKEYSVIVPSRTVIVVHDSLLISPFQIAKALNEARLEANVRVNGETSFKNKWPSPFAVVSGLLLLLSFLKFVYSPLRWLAVAAVAAGIYPILAKAFASIKRPRIDINILVIITVIATLAMQDFMEAAAVVFLFTISDWLETRASYKATSVMQSLMSLAPQKAIIAETGEEVEVDEVKVDTVVAVKAGETIPIDGIVVDGNCEVDEKTLTGEAFPVPKQRDSTVWAGTINLNGYICVKTTSLAGDCVVAKMAKLVEEAQSSKTKSQRLIDKCSQYYTPAIILVSACVAIVPVIMKVHNLKHWFHLALVVLVSGCPCGLILSTPVATFCALTKAATSGLLIKSADYLDTLSKIKIVAFDKTGTITRGEFIVIDFKSLSRDINLRSLLYWVSSVESKSSHPMAATIVDYAKSVSVEPRPEEVEDYQNFPGEGIYGKIDGNDIFIGNKKIASRAGCSTVPEIEVDTKGGKTVGYVYVGERLAGFFNLSDACRSGVSQAMAELKSLGIKTAMLTGDNQAAAMHAQEQLGNVLDVVHGDLLPEDKSRIIQEFKKEGPTAMVGDGVNDAPALATADIGISMGISGSALATQTGNIILMSNDIRRIPQAVKLARRARRKVVENVCLSIILKAGILALAFAGHPLIWAAVLVDVGTCLLVIFNSMLLLREKKKIGNKKCYRASTSKLNGRKLEGDDDYVVDLEAGLLTKSGNGQCKSSCCGDKKNQENVVMMKPSSKTSSDHSHPGCCGDKKEEKVKPLVKDGCCSEKTRKSEGDMVSLSSCKKSSHVKHDLKMKGGSGCCASKNEKGKEVVAKSCCEKPKQQVESVGDCKSGHCEKKKQAEDIVVPVQIIGHALTHVEIELQTKETCKTSCCDSKEKVKETGLLLSSENTPYLEKGVLIKDEGNCKSGSENMGTVKQSCHEKGCSDEKQTGEITLASEEETDDQDCSSGCCVNEGTVKQSFDEKKHSVLVEKEGLDMETGFCCDAKLVCCGNTEGEVKEQCRLEIKKEEHCKSGCCGEEIQTGEITLVSEEETESTNCSTGCCVDKEEVTQTCHEKPASLVVSGLEVKKDEHCESSHRAVKVETCCKVKIPEACASKCRDRAKRHSGKSCCRSYAKELCSHRHHHHHHHHHHHVSA

>AtHMA5

MATKLLSLTCIRKERFSERYPLVRKHLTRSRDGGGGSSSETAAFEIDDPISRAVFQVLGMTCSACAGSVEKAIKRLPGIHDAVIDALNNRAQILFYPNSVDVETIRETIEDAGFEASLIENEANERSRQVCRIRINGMTCTSCSSTIERVLQSVNGVQRAHVALAIEEAEIHYDPRLSSYDRLLEEIENAGFEAVLISTGEDVSKIDLKIDGELTDESMKVIERSLEALPGVQSVEISHGTDKISVLYKPDVTGPRNFIQVIESTVFGHSGHIKATIFSEGGVGRESQKQGEIKQYYKSFLWSLVFTVPVFLTAMVFMYIPGIKDLLMFKVINMLTVGEIIRCVLATPVQFVIGWRFYTGSYKALRRGSANMDVLIALGTNAAYFYSLYTVLRAATSPDFKGVDFFETSAMLISFIILGKYLEVMAKGKTSQAIAKLMNLAPDTAILLSLDKEGNVTGEEEIDGRLIQKNDVIKIVPGAKVASDGYVIWGQSHVNESMITGEARPVAKRKGDTVIGGTLNENGVLHVKVTRVGSESALAQIVRLVESAQLAKAPVQKLADRISKFFVPLVIFLSFSTWLAWFLAGKLHWYPESWIPSSMDSFELALQFGISVMVIACPCALGLATPTAVMVGTGVGASQGVLIKGGQALERAHKVNCIVFDKTGTLTMGKPVVVKTKLLKNMVLREFYELVAATEVNSEHPLAKAIVEYAKKFRDDEENPAWPEACDFVSITGKGVKATVKGREIMVGNKNLMNDHKVIIPDDAEELLADSEDMAQTGILVSINSELIGVLSVSDPLKPSAREAISILKSMNIKSIMVTGDNWGTANSIAREVGIDSVIAEAKPEQKAEKVKELQAAGHVVAMVGDGINDSPALVAADVGMAIGAGTDIAIEAADIVLMKSNLEDVITAIDLSRKTFSRIRLNYVWALGYNLMGIPIAAGVLFPGTRFRLPPWIAGAAMAASSVSVVCCSLLLKNYKRPKKLDHLEIREIQVERV

>AtHMA6

MESTLSAFSTVKATAMARSSGGPSLPLLTISKALNRHFTGARHLHPLLLARCSPSVRRLGGFHGSRFTSSNSALRSLGAAVLPVIRHRLECLSSSSPSFRSISSGGGSGFGGYNGGSGGGGGGGSESGDSKSKLGANASDGVSVPSSDIIILDVGGMTCGGCSASVKKILESQPQVASASVNLTTETAIVWPVPEAKSVPDWQKSLGETLANHLTNCGFQSTPRDLVTENFFKVFETKTKDKQARLKESGRELAVSWALCAVCLVGHLTHFLGVNAPWIHAIHSTGFHVSLCLITLLGPGRKLVLDGIKSLLKGSPNMNTLVGLGALSSFSVSSLAAMIPKLGWKTFFEEPVMLIAFVLLGRNLEQRAKIKATSDMTGLLSVLPSKARLLLDGDLQNSTVEVPCNSLSVGDLVVILPGDRVPADGVVKSGRSTIDESSFTGEPLPVTKESGSQVAAGSINLNGTLTVEVHRSGGETAVGDIIRLVEEAQSREAPVQQLVDKVAGRFTYGVMALSAATFTFWNLFGAHVLPSALHNGSPMSLALQLSCSVLVVACPCALGLATPTAMLVGTSLGARRGLLLRGGDILEKFSLVDTVVFDKTGTLTKGHPVVTEVIIPENPRHNLNDTWSEVEVLMLAAAVESNTTHPVGKAIVKAARARNCQTMKAEDGTFTEEPGSGAVAIVNNKRVTVGTLEWVKRHGATGNSLLALEEHEINNQSVVYIGVDNTLAAVIRFEDKVREDAAQVVENLTRQGIDVYMLSGDKRNAANYVASVVGINHERVIAGVKPAEKKNFINELQKNKKIVAMVGDGINDAAALASSNVGVAMGGGAGAASEVSPVVLMGNRLTQLLDAMELSRQTMKTVKQNLWWAFGYNIVGIPIAAGVLLPLTGTMLTPSMAGALMGVSSLGVMTNSLLLRYRFFSNRNDKNVKPEPKEGTKQPHENTRWKQSS

>AtHMA7

MAPSRRDLQLTPVTGGSSSQISDMEEVGLLDSYHNEANADDILTKIEEGRDVSGLRKIQVGVTGMTCAACSNSVEAALMNVNGVFKASVALLQNRADVVFDPNLVKEEDIKEAIEDAGFEAEILAEEQTQATLVGQFTIGGMTCAACVNSVEGILRDLPGVKRAVVALSTSLGEVEYDPNVINKDDIVNAIEDAGFEGSLVQSNQQDKLVLRVDGILNELDAQVLEGILTRLNGVRQFRLDRISGELEVVFDPEVVSSRSLVDGIEEDGFGKFKLRVMSPYERLSSKDTGEASNMFRRFISSLVLSIPLFFIQVICPHIALFDALLVWRCGPFMMGDWLKWALVSVIQFVIGKRFYVAAWRALRNGSTNMDVLVALGTSASYFYSVGALLYGAVTGFWSPTYFDASAMLITFVLLGKYLESLAKGKTSDAMKKLVQLTPATAILLTEGKGGKLVGEREIDALLIQPGDTLKVHPGAKIPADGVVVWGSSYVNESMVTGESVPVSKEVDSPVIGGTINMHGALHMKATKVGSDAVLSQIISLVETAQMSKAPIQKFADYVASIFVPVVITLALFTLVGWSIGGAVGAYPDEWLPENGTHFVFSLMFSISVVVIACPCALGLATPTAVMVATGVGATNGVLIKGGDALEKAHKVKYVIFDKTGTLTQGKATVTTTKVFSEMDRGEFLTLVASAEASSEHPLAKAIVAYARHFHFFDESTEDGETNNKDLQNSGWLLDTSDFSALPGKGIQCLVNEKMILVGNRKLMSENAINIPDHVEKFVEDLEESGKTGVIVAYNGKLVGVMGIADPLKREAALVVEGLLRMGVRPIMVTGDNWRTARAVAKEVGIEDVRAEVMPAGKADVIRSLQKDGSTVAMVGDGINDSPALAAADVGMAIGAGTDVAIEAADYVLMRNNLEDVITAIDLSRKTLTRIRLNYVFAMAYNVVSIPIAAGVFFPVLRVQLPPWAAGACMALSSVSVVCSSLLLRRYKKPRLTTVLKITTE

>AtHMA8

MASNLLRFPLPPPSSLHIRPSKFLVNRCFPRLRRSRIRRHCSRPFFLVSNSVEISTQSFESTESSIESVKSITSDTPILLDVSGMMCGGCVARVKSVLMSDDRVASAVVNMLTETAAVKFKPEVEVTADTAESLAKRLTESGFEAKRRVSGMGVAENVKKWKEMVSKKEDLLVKSRNRVAFAWTLVALCCGSHTSHILHSLGIHIAHGGIWDLLHNSYVKGGLAVGALLGPGRELLFDGIKAFGKRSPNMNSLVGLGSMAAFSISLISLVNPELEWDASFFDEPVMLLGFVLLGRSLEERAKLQASTDMNELLSLISTQSRLVITSSDNNTPVDSVLSSDSICINVSVDDIRVGDSLLVLPGETFPVDGSVLAGRSVVDESMLTGESLPVFKEEGCSVSAGTINWDGPLRIKASSTGSNSTISKIVRMVEDAQGNAAPVQRLADAIAGPFVYTIMSLSAMTFAFWYYVGSHIFPDVLLNDIAGPDGDALALSLKLAVDVLVVSCPCALGLATPTAILIGTSLGAKRGYLIRGGDVLERLASIDCVALDKTGTLTEGRPVVSGVASLGYEEQEVLKMAAAVEKTATHPIAKAIVNEAESLNLKTPETRGQLTEPGFGTLAEIDGRFVAVGSLEWVSDRFLKKNDSSDMVKLESLLDHKLSNTSSTSRYSKTVVYVGREGEGIIGAIAISDCLRQDAEFTVARLQEKGIKTVLLSGDREGAVATVAKNVGIKSESTNYSLSPEKKFEFISNLQSSGHRVAMVGDGINDAPSLAQADVGIALKIEAQENAASNAASVILVRNKLSHVVDALSLAQATMSKVYQNLAWAIAYNVISIPIAAGVLLPQYDFAMTPSLSGGLMALSSIFVVSNSLLLQLHKSETSKNSL

>AtECA1

MGKGSEDLVKKESLNSTPVNSDTFPAWAKDVAECEEHFVVSREKGLSSDEVLKRHQIYGLNELEKPEGTSIFKLILEQFNDTLVRILLAAAVISFVLAFFDGDEGGEMGITAFVEPLVIFLILIVNAIVGIWQETNAEKALEALKEIQSQQATVMRDGTKVSSLPAKELVPGDIVELRVGDKVPADMRVVALISSTLRVEQGSLTGESEAVSKTTKHVDENADIQGKKCMVFAGTTVVNGNCICLVTDTGMNTEIGRVHSQIQEAAQHEEDTPLKKKLNEFGEVLTMIIGLICALVWLINVKYFLSWEYVDGWPRNFKFSFEKCTYYFEIAVALAVAAIPEGLPAVITTCLALGTRKMAQKNALVRKLPSVETLGCTTVICSDKTGTLTTNQMAVSKLVAMGSRIGTLRSFNVEGTSFDPRDGKIEDWPMGRMDANLQMIAKIAAICNDANVEQSDQQFVSRGMPTEAALKVLVEKMGFPEGLNEASSDGDVLRCCRLWSELEQRIATLEFDRDRKSMGVMVDSSSGNKLLLVKGAVENVLERSTHIQLLDGSKRELDQYSRDLILQSLRDMSLSALRCLGFAYSDVPSDFATYDGSEDHPAHQQLLNPSNYSSIESNLIFVGFVGLRDPPRKEVRQAIADCRTAGIRVMVITGDNKSTAEAICREIGVFEADEDISSRSLTGIEFMDVQDQKNHLRQTGGLLFSRAEPKHKQEIVRLLKEDGEVVAMTGDGVNDAPALKLADIGVAMGISGTEVAKEASDMVLADDNFSTIVAAVGEGRSIYNNMKAFIRYMISSNIGEVASIFLTAALGIPEGMIPVQLLWVNLVTDGPPATALGFNPPDKDIMKKPPRRSDDSLITAWILFRYMVIGLYVGVATVGVFIIWYTHSSFMGIDLSQDGHSLVSYSQLAHWGQCSSWEGFKVSPFTAGSQTFSFDSNPCDYFQQGKIKASTLSLSVLVAIEMFNSLNALSEDGSLVTMPPWVNPWLLLAMAVSFGLHFVILYVPFLAQVFGIVPLSLNEWLLVLAVSLPVILIDEVLKFVGRCTSGYRYSPRTLSTKQKEE

>AtECA2

MEEEKSFSAWSWSVEQCLKEYKTRLDKGLTSEDVQIRRQKYGFNELAKEKGKPLWHLVLEQFDDTLVKILLGAAFISFVLAFLGEEHGSGSGFEAFVEPFVIVLILILNAVVGVWQESNAEKALEALKEMQCESAKVLRDGNVLPNLPARELVPGDIVELNVGDKVPADMRVSGLKTSTLRVEQSSLTGEAMPVLKGANLVVMDDCELQGKENMVFAGTTVVNGSCVCIVTSIGMDTEIGKIQRQIHEASLEESETPLKKKLDEFGSRLTTAICIVCVLVWMINYKNFVSWDVVDGYKPVNIKFSFEKCTYYFKIAVALAVAAIPEGLPAVITTCLALGTRKMAQKNAIVRKLPSVETLGCTTVICSDKTGTLTTNQMSATEFFTLGGKTTTTRVFSVSGTTYDPKDGGIVDWGCNNMDANLQAVAEICSICNDAGVFYEGKLFRATGLPTEAALKVLVEKMGIPEKKNSENIEEVTNFSDNGSSVKLACCDWWNKRSKKVATLEFDRVRKSMSVIVSEPNGQNRLLVKGAAESILERSSFAQLADGSLVALDESSREVILKKHSEMTSKGLRCLGLAYKDELGEFSDYSSEEHPSHKKLLDPSSYSNIETNLIFVGVVGLRDPPREEVGRAIEDCRDAGIRVMVITGDNKSTAEAICCEIRLFSENEDLSQSSFTGKEFMSLPASRRSEILSKSGGKVFSRAEPRHKQEIVRMLKEMGEIVAMTGDGVNDAPALKLADIGIAMGITGTEVAKEASDMVLADDNFSTIVSAVAEGRSIYNNMKAFIRYMISSNVGEVISIFLTAALGIPECMIPVQLLWVNLVTDGPPATALGFNPADIDIMKKPPRKSDDCLIDSWVLIRYLVIGSYVGVATVGIFVLWYTQASFLGISLISDGHTLVSFTQLQNWSECSSWGTNFTATPYTVAGGLRTIAFENNPCDYFTLGKVKPMTLSLTVLVAIEMFNSLNALSEDNSLLTMPPWRNPWLLVAMTVSFALHCVILYVPFLANVFGIVPLSFREWFVVILVSFPVILIDEALKFIGRCRRTRIKKKIKTM

>AtECA3

MEDAYARSVSEVLDFFGVDPTKGLSDSQVVHHSRLYGRNVLPEEKRTPFWKLVLKQFDDLLVKILIVAAIVSFVLALANGETGLTAFLEPFVILLILAANAAVGVITETNAEKALEELRAYQANIATVLRNGCFSILPATELVPGDIVEVTVGCKIPADLRMIEMSSNTFRVDQAILTGESCSVEKDVDCTLTTNAVYQDKKNILFSGTDVVAGRGRAVVIGVGSNTAMGSIHDSMLQTDDEATPLKKKLDEFGSFLAKVIAGICVLVWVVNIGHFSDPSHGGFFKGAIHYFKIAVALAVAAIPEGLPAVVTTCLALGTKKMARLNAIVRSLPSVETLGCTTVICSDKTGTLTTNMMSVSKICVVQSAEHGPMINEFTVSGTTYAPEGTVFDSNGMQLDLPAQSPCLHHLAMCSSLCNDSILQYNPDKDSYEKIGESTEVALRVLAEKVGLPGFDSMPSALNMLSKHERASYCNHYWENQFKKVYVLEFTRDRKMMSVLCSHKQMDVMFSKGAPESIIARCNKILCNGDGSVVPLTAAGRAELESRFYSFGDETLRCLALAFKTVPHGQQTISYDNENDLTFIGLVGMLDPPREEVRDAMLACMTAGIRVIVVTGDNKSTAESLCRKIGAFDNLVDFSGMSYTASEFERLPAVQQTLALRRMTLFSRVEPSHKRMLVEALQKQNEVVAMTGDGVNDAPALKKADIGIAMGSGTAVAKSASDMVLADDNFASIVAAVAEGRAIYNNTKQFIRYMISSNIGEVVCIFVAAVLGIPDTLAPVQLLWVNLVTDGLPATAIGFNKQDSDVMKAKPRKVGEAVVTGWLFFRYLVIGVYVGLATVAGFIWWFVYSDGGPKLTYSELMNFETCALRETTYPCSIFEDRHPSTVAMTVLVVVEMFNALNNLSENQSLLVITPRSNLWLVGSIILTMLLHVLILYVHPLAVLFSVTPLSWAEWTAVLYLSFPVIIIDELLKFLSRNTGMRFRFRLRKADLLPKDRRDK

>AtECA4

MGKGGEDCGNKQTNSSELVKSDTFPAWGKDVSECEEKFGVSREKGLSTDEVLKRHQIYGLNELEKPEGTSIFKLILEQFNDTLVRILLAAAVISFVLAFFDGDEGGEMGITAFVEPLVIFLILIVNAIVGIWQETNAEKALEALKEIQSQQATVMRDGTKVSSLPAKELVPGDIVELRVGDKVPADMRVVALISSTLRVEQGSLTGESEAVSKTTKHVDENADIQGKKCMVFAGTTVVNGNCICLVTDTGMNTEIGRVHSQIQEAAQHEEDTPLKKKLNEFGEVLTMIIGLICALVWLINVKYFLSWEYVDGWPRNFKFSFEKCTYYFEIAVALAVAAIPEGLPAVITTCLALGTRKMAQKNALVRKLPSVETLGCTTVICSDKTGTLTTNQMAVSKLVAMGSRIGTLRSFNVEGTSFDPRDGKIEDWPTGRMDANLQMIAKIAAICNDANVEKSDQQFVSRGMPTEAALKVLVEKMGFPEGLNEASSDGNVLRCCRLWSELEQRIATLEFDRDRKSMGVMVDSSSGKKLLLVKGAVENVLERSTHIQLLDGSTRELDQYSRDLILQSLHDMSLSALRCLGFAYSDVPSDFATYDGSEDHPAHQQLLNPSNYSSIESNLVFVGFVGLRDPPRKEVRQAIADCRTAGIRVMVITGDNKSTAEAICREIGVFEADEDISSRSLTGKEFMDVKDQKNHLRQTGGLLFSRAEPKHKQEIVRLLKEDGEVVAMTGDGVNDAPALKLADIGVAMGISGTEVAKEASDLVLADDNFSTIVAAVGEGRSIYNNMKAFIRYMISSNIGEVASIFLTAALGIPEGMIPVQLLWVNLVTDGPPATALGFNPPDKDIMKKPPRRSDDSLITAWILFRYMVIGLYVGVATVGVFIIWYTHNSFMGIDLSQDGHSLVSYSQLAHWGQCSSWEGFKVSPFTAGSQTFSFDSNPCDYFQQGKIKASTLSLSVLVAIEMFNSLNALSEDGSLVTMPPWVNPWLLLAMAVSFGLHFVILYVPFLAQVFGIVPLSLNEWLLVLAVSLPVILIDEVLKFVGRCTSGYRYSPRTPSAKQKEE

>AtACA1

MESYLNENFGDVKPKNSSDEALQRWRKLCWIVKNPKRRFRFTANLSKRSEAEAIRRSNQEKFRVAVLVSQAALQFINSLKLSSEYTLPEEVRKAGFEICPDELGSIVEGHDLKKLKIHGGTEGLTEKLSTSIASGISTSEDLLSVRKEIYGINQFTESPSRGFWLFVWEALQDTTLMILAACAFVSLIVGILMEGWPIGAHDGLGIVASILLVVFVTATSDYRQSLQFKDLDAEKKKIVVQVTRDKLRQKISIYDLLPGDVVHLGIGDQIPADGLFISGFSVLINESSLTGESEPVSVSVEHPFLLSGTKVQDGSCKMLVTTVGMRTQWGKLMATLSEGGDDETPLQVKLNGVATIIGKIGLFFAVITFAVLVQGLANQKRLDNSHWIWTADELMAMLEYFAVAVTIVVVAVPEGLPLAVTLSLAFAMKKMMNDKALVRNLAACETMGSATTICSDKTGTLTTNHMTVVKACICEQAKEVNGPDAAMKFASGIPESAVKLLLQSIFTNTGGEIVVGKGNKTEILGTPTETALLEFGLSLGGDFQEVRQASNVVKVEPFNSTKKRMGVVIELPERHFRAHCKGASEIVLDSCDKYINKDGEVVPLDEKSTSHLKNIIEEFASEALRTLCLAYFEIGDEFSLEAPIPSGGYTCIGIVGIKDPVRPGVKESVAICKSAGITVRMVTGDNLTTAKAIARECGILTDDGIAIEGPEFREKSDEELLKLIPKLQVMARSSPMDKHTLVRLLRTMFQEVVAVTGDGTNDAPALHEADIGLAMGISGTEVAKESADVIILDDNFSTIVTVAKWGRSVYINIQKFVQFQLTVNVVALIVNFLSACLTGNAPLTAVQLLWVNMIMDTLGALALATEPPQDDLMKRSPVGRKGNFISNVMWRNILGQSLYQLVIIWCLQTKGKTMFGLDGPDSDLTLNTLIFNIFVFCQVFNEISSREMEKIDVFKGILKNYVFVAVLTCTVVFQVIIIELLGTFADTTPLNLGQWLVSIILGFLGMPVAAALKMIPVGSH

>AtACA2

MESYLNENFDVKAKHSSEEVLEKWRNLCGVVKNPKRRFRFTANLSKRYEAAAMRRTNQEKLRIAVLVSKAAFQFISGVSPSDYTVPEDVKAAGFEICADELGSIVESHDVKKLKFHGGVDGLAGKLKASPTDGLSTEAAQLSQRQELFGINKFAESEMRGFWVFVWEALQDMTLMILGVCAFVSLIVGIATEGWPKGSHDGLGIAASILLVVFVTATSDYRQSLQFRDLDKEKKKITVQVTRNGFRQKLSIYDLLPGDIVHLAIGDQVPADGLFLSGFSVVIDESSLTGESEPVMVNAQNPFLMSGTKVQDGSCKMMITTVGMRTQWGKLMATLTEGGDDETPLQVKLNGVATIIGKIGLFFAVVTFAVLVQGMFMRKLSTGTHWVWSGDEALELLEYFAIAVTIVVVAVPEGLPLAVTLSLAFAMKKMMNDKALVRHLAACETMGSATTICSDKTGTLTTNHMTVVKSCICMNVQDVANKGSSLQSEIPESAVKLLIQSIFNNTGGEVVVNKHGKTELLGTPTETAILELGLSLGGKFQEERKSYKVIKVEPFNSTKKRMGVVIELPEGGRMRAHTKGASEIVLAACDKVVNSSGEVVPLDEESIKYLNVTINEFANEALRTLCLAYMDIEGGFSPDDAIPASGFTCVGIVGIKDPVRPGVKESVELCRRAGITVRMVTGDNINTAKAIARECGILTDDGIAIEGPVFREKNQEELLELIPKIQVMARSSPMDKHTLVKQLRTTFDEVVAVTGDGTNDAPALHEADIGLAMGIAGTEVAKESADVIILDDNFSTIVTVAKWGRSVYINIQKFVQFQLTVNVVALVVNFSSACLTGSAPLTAVQLLWVNMIMDTLGALALATEPPNDELMKRLPVGRRGNFITNAMWRNILGQAVYQFIVIWILQAKGKAMFGLDGPDSTLMLNTLIFNCFVFCQVFNEISSREMEEIDVFKGILDNYVFVVVIGATVFFQIIIIEFLGTFASTTPLTITQWIFSIFIGFLGMPIAAGLKTIPV

>AtACA4

MSNLLRDFEVEAKNPSLEARQRWRSSVSIVKNRTRRFRNIRDLDKLADYENKKHQIQEKIRVAFFVQKAALHFIDAAARPEYKLTDEVKKAGFSIEADELASMVRKNDTKSLAQKGGVEELAKKVSVSLSEGIRSSEVPIREKIFGENRYTEKPARSFLMFVWEALHDITLIILMVCAVVSIGVGVATEGFPRGMYDGTGILLSILLVVMVTAISDYKQSLQFRDLDREKKKIIVQVTRDGSRQEISIHDLVVGDVVHLSIGDQVPADGIFISGYNLEIDESSLSGESEPSHVNKEKPFLLSGTKVQNGSAKMLVTTVGMRTEWGKLMETLVDGGEDETPLQVKLNGVATIIGKIGLSFAVLTFVVLCIRFVLDKATSGSFTNWSSEDALTLLDYFAISVTIIVVAVPEGLPLAVTLSLAFAMKKLMSDRALVRHLAACETMGSSTCICTDKTGTLTTNHMVVNKVWICDKVQERQEGSKESFELELSEEVQSTLLQGIFQNTGSEVVKDKDGNTQILGSPTERAILEFGLLLGGDFNTQRKEHKILKIEPFNSDKKKMSVLIALPGGGARAFCKGASEIVLKMCENVVDSNGESVPLTEERITSISDIIEGFASEALRTLCLVYKDLDEAPSGELPDGGYTMVAVVGIKDPVRPGVREAVQTCQAAGITVRMVTGDNISTAKAIAKECGIYTEGGLAIEGSEFRDLSPHEMRAIIPKIQVMARSLPLDKHTLVSNLRKIGEVVAVTGDGTNDAPALHEADIGLAMGIAGTEVAKENADVIIMDDNFKTIVNVARWGRAVYINIQKFVQFQLTVNVVALIINFVSACITGSAPLTAVQLLWVNMIMDTLGALALATEPPNEGLMKRAPIARTASFITKTMWRNIAGQSVYQLIVLGILNFAGKSLLKLDGPDSTAVLNTVIFNSFVFCQVFNEINSREIEKINVFKGMFNSWVFTWVMTVTVVFQVIIVEFLGAFASTVPLSWQHWLLSILIGSLNMIVAVILKCVPVESRHHHDGYDLLPSGPSSSNS

>AtACA7

MESYLNSNFDVKAKHSSEEVLEKWRNLCSVVKNPKRRFRFTANLSKRYEAAAMRRTNQEKLRIAVLVSKAAFQFISGVSPSDYKVPEEVKAAGFDICADELGSIVEGHDVKKLKFHGGVDGLSGKLKACPNAGLSTGEPEQLSKRQELFGINKFAESELRSFWVFVWEALQDMTLMILGVCAFVSLIVGIATEGWPQGSHDGLGIVASILLVVFVTATSDYRQSLQFRDLDKEKKKITVQVTRNGFRQKMSIYDLLPGDVVHLAIGDQVPADGLFLSGFSVVIDESSLTGESEPVMVTAQNPFLLSGTKVQDGSCKMLVTTVGMRTQWGKLMATLSEGGDDETPLQVKLNGVATIIGKIGLSFAIVTFAVLVQGMFMRKLSLGPHWWWSGDDALELLEYFAIAVTIVVVAVPEGLPLAVTLSLAFAMKKMMNDKALVRHLAACETMGSATTICSDKTGTLTTNHMTVVKSCICMNVQDVASKSSSLQSDIPEAALKLLLQLIFNNTGGEVVVNERGKTEILGTPTETAILELGLSLGGKFQEERQSNKVIKVEPFNSTKKRMGVVIELPEGGRIRAHTKGASEIVLAACDKVINSSGEVVPLDDESIKFLNVTIDEFANEALRTLCLAYMDIESGFSADEGIPEKGFTCIGIVGIKDPVRPGVRESVELCRRAGIMVRMVTGDNINTAKAIARECGILTDDGIAIEGPVFREKNQEEMLELIPKIQVMARSSPMDKHTLVKQLRTTFDEVVAVTGDGTNDAPALHEADIGLAMGIAGTEVAKEIADVIILDDNFSTIVTVAKWGRSVYINIQKFVQFQLTVNVVALIVNFSSACLTGSAPLTAVQLLWVNMIMDTLGALALATEPPNNELMKRMPVGRRGNFITNAMWRNILGQAVYQFIIIWILQAKGKSMFGLVGSDSTLVLNTLIFNCFVFCQVFNEVSSREMEEIDVFKGILDNYVFVVVIGATVFFQIIIIEFLGTFASTTPLTIVQWFFSIFVGFLGMPIAAGLKKIPV

>AtACA8

MTSLLKSSPGRRRGGDVESGKSEHADSDSDTFYIPSKNASIERLQQWRKAALVLNASRRFRYTLDLKKEQETREMRQKIRSHAHALLAANRFMDMGRESGVEKTTGPATPAGDFGITPEQLVIMSKDHNSGALEQYGGTQGLANLLKTNPEKGISGDDDDLLKRKTIYGSNTYPRKKGKGFLRFLWDACHDLTLIILMVAAVASLALGIKTEGIKEGWYDGGSIAFAVILVIVVTAVSDYKQSLQFQNLNDEKRNIHLEVLRGGRRVEISIYDIVVGDVIPLNIGNQVPADGVLISGHSLALDESSMTGESKIVNKDANKDPFLMSGCKVADGNGSMLVTGVGVNTEWGLLMASISEDNGEETPLQVRLNGVATFIGSIGLAVAAAVLVILLTRYFTGHTKDNNGGPQFVKGKTKVGHVIDDVVKVLTVAVTIVVVAVPEGLPLAVTLTLAYSMRKMMADKALVRRLSACETMGSATTICSDKTGTLTLNQMTVVESYAGGKKTDTEQLPATITSLVVEGISQNTTGSIFVPEGGGDLEYSGSPTEKAILGWGVKLGMNFETARSQSSILHAFPFNSEKKRGGVAVKTADGEVHVHWKGASEIVLASCRSYIDEDGNVAPMTDDKASFFKNGINDMAGRTLRCVALAFRTYEAEKVPTGEELSKWVLPEDDLILLAIVGIKDPCRPGVKDSVVLCQNAGVKVRMVTGDNVQTARAIALECGILSSDADLSEPTLIEGKSFREMTDAERDKISDKISVMGRSSPNDKLLLVQSLRRQGHVVAVTGDGTNDAPALHEADIGLAMGIAGTEVAKESSDIIILDDNFASVVKVVRWGRSVYANIQKFIQFQLTVNVAALVINVVAAISSGDVPLTAVQLLWVNLIMDTLGALALATEPPTDHLMGRPPVGRKEPLITNIMWRNLLIQAIYQVSVLLTLNFRGISILGLEHEVHEHATRVKNTIIFNAFVLCQAFNEFNARKPDEKNIFKGVIKNRLFMGIIVITLVLQVIIVEFLGKFASTTKLNWKQWLICVGIGVISWPLALVGKFIPVPAAPISNKLKVLKFWGKKKNSSGEGSL

>AtACA9

MSTSSSNGLLLTSMSGRHDDMEAGSAKTEEHSDHEELQHDPDDPFDIDNTKNASVESLRRWRQAALVLNASRRFRYTLDLNKEEHYDNRRRMIRAHAQVIRAALLFKLAGEQQIAFGSSTPAASTGNFDIDLEKLVSMTRNQNMSNLQQYGGVKGVAEKLKSNMEQGINEDEKEVIDRKNAFGSNTYPKKKGKNFFMFLWEAWQDLTLIILIIAAVTSLALGIKTEGLKEGWLDGGSIAFAVLLVIVVTAVSDYRQSLQFQNLNDEKRNIQLEVMRGGRTVKISIYDVVVGDVIPLRIGDQVPADGVLISGHSLAIDESSMTGESKIVHKDQKSPFLMSGCKVADGVGNMLVTGVGINTEWGLLMASISEDTGEETPLQVRLNGLATFIGIVGLSVALVVLVALLVRYFTGTTQDTNGATQFIKGTTSISDIVDDCVKIFTIAVTIVVVAVPEGLPLAVTLTLAYSMRKMMADKALVRRLSACETMGSATTICSDKTGTLTLNQMTVVETYAGGSKMDVADNPSGLHPKLVALISEGVAQNTTGNIFHPKDGGEVEISGSPTEKAILSWAYKLGMKFDTIRSESAIIHAFPFNSEKKRGGVAVLRGDSEVFIHWKGAAEIVLACCTQYMDSNGTLQSIESQKEFFRVAIDSMAKNSLRCVAIACRTQELNQVPKEQEDLDKWALPEDELILLAIVGIKDPCRPGVREAVRICTSAGVKVRMVTGDNLQTAKAIALECGILSSDTEAVEPTIIEGKVFRELSEKEREQVAKKITVMGRSSPNDKLLLVQALRKNGDVVAVTGDGTNDAPALHEADIGLSMGISGTEVAKESSDIIILDDNFASVVKVVRWGRSVYANIQKFIQFQLTVNVAALIINVVAAMSSGDVPLKAVQLLWVNLIMDTLGALALATEPPTDHLMHRTPVGRREPLITNIMWRNLLVQSFYQVAVLLVLNFAGLSILGLNHENHAHAVEVKNTMIFNAFVMCQIFNEFNARKPDEMNVFRGVNKNPLFVAIVGVTFILQIIIVTFLGKFAHTVRLGWQLWLASIIIGLVSWPLAIVGKLIPVPKTPMSVYFKKPFRKYKASRNA

>AtACA10

MSGQFNNSPRGEDKDVEAGTSSFTEYEDSPFDIASTKNAPVERLRRWRQAALVLNASRRFRYTLDLKREEDKKQMLRKMRAHAQAIRAAHLFKAAASRVTGIASPLPTPGGGDFGIGQEQIVSISRDQNIGALQELGGVRGLSDLLKTNLEKGIHGDDDDILKRKSAFGSNTYPQKKGRSFWRFVWEASQDLTLIILIVAAVASLALGIKTEGIEKGWYDGISIAFAVLLVIVVTATSDYRQSLQFQNLNEEKRNIRLEVTRDGRRVEISIYDIVVGDVIPLNIGDQVPADGVLVAGHSLAVDESSMTGESKIVQKNSTKHPFLMSGCKVADGNGTMLVTGVGVNTEWGLLMASVSEDNGGETPLQVRLNGVATFIGIVGLTVAGVVLFVLVVRYFTGHTKNEQGGPQFIGGKTKFEHVLDDLVEIFTVAVTIVVVAVPEGLPLAVTLTLAYSMRKMMADKALVRRLSACETMGSATTICSDKTGTLTLNEMTVVECYAGLQKMDSPDSSSKLPSAFTSILVEGIAHNTTGSVFRSESGEIQVSGSPTERAILNWAIKLGMDFDALKSESSAVQFFPFNSEKKRGGVAVKSPDSSVHIHWKGAAEIVLGSCTHYMDESESFVDMSEDKMGGLKDAIDDMAARSLRCVAIAFRTFEADKIPTDEEQLSRWELPEDDLILLAIVGIKDPCRPGVKNSVLLCQQAGVKVRMVTGDNIQTAKAIALECGILASDSDASEPNLIEGKVFRSYSEEERDRICEEISVMGRSSPNDKLLLVQSLKRRGHVVAVTGDGTNDAPALHEADIGLAMGIQGTEVAKEKSDIIILDDNFESVVKVVRWGRSVYANIQKFIQFQLTVNVAALVINVVAAISAGEVPLTAVQLLWVNLIMDTLGALALATEPPTDHLMDRAPVGRREPLITNIMWRNLFIQAMYQVTVLLILNFRGISILHLKSKPNAERVKNTVIFNAFVICQVFNEFNARKPDEINIFRGVLRNHLFVGIISITIVLQVVIVEFLGTFASTTKLDWEMWLVCIGIGSISWPLAVIGKLIPVPETPVSQYFRINRWRRNSSG

>AtACA11

MSNLLKDFEVASKNPSLEARQRWRSSVGLVKNRARRFRMISNLDKLAENEKKRCQIQEKIRVVFYVQKAAFQFIDAGARPEYKLTDEVKKAGFYVEADELASMVRNHDTKSLTKIGGPEGIAQKVSVSLAEGVRSSELHIREKIYGENRYTEKPARSFLTFVWEALQDITLIILMVCAVVSIGVGVATEGFPKGMYDGTGILLSIILVVMVTAISDYKQSLQFRDLDREKKKIIIQVTRDGSRQEVSIHDLVVGDVVHLSIGDQVPADGIFISGYNLEIDESSLSGESEPSHVNKEKPFLLSGTKVQNGSAKMLVTTVGMRTEWGKLMDTLSEGGEDETPLQVKLNGVATIIGKIGLGFAVLTFVVLCIRFVVEKATAGSITEWSSEDALTLLDYFAIAVTIIVVAVPEGLPLAVTLSLAFAMKQLMSDRALVRHLAACETMGSSTCICTDKTGTLTTNHMVVNKVWICENIKERQEENFQLNLSEQVKNILIQAIFQNTGSEVVKDKEGKTQILGSPTERAILEFGLLLGGDVDTQRREHKILKIEPFNSDKKKMSVLTSHSGGKVRAFCKGASEIVLKMCEKVVDSNGESVPLSEEKIASISDVIEGFASEALRTLCLVYTDLDEAPRGDLPNGGYTLVAVVGIKDPVRPGVREAVQTCQAAGITVRMVTGDNISTAKAIAKECGILTAGGVAIEGSDFRNLPPHEMRAILPKIQVMARSLPLDKHTLVNNLRKMGEVVAVTGDGTNDAPALHEADIGLAMGIAGTEVAKENADVIIMDDNFATIVNVAKWGRAVYINIQKFVQFQLTVNVVALIINFVSACITGSAPLTAVQLLWVNMIMDTLGALALATEPPNEGLMKRQPIGRTASFITRAMWRNIIGQSIYQLIVLGILNFAGKQILNLNGPDSTIVLNTIIFNSFVFCQVFNEVNSREIEKINVFEGMFKSWVFVAVMTATVGFQVIIVEFLGAFASTVPLSWQHWLLCILIGSVSMILAVGLKCIPVESNRHHDGYELLPSGPSDSA

>AtACA12

MRDLKEYDYSALLLNLTTSSLNKAQRRWRFAYAAIYSMRAMLSLVKEIVPARIDPKTSDASLSLSYTALESGEGAKINSMPLSYVPAIDQEQLVEIMKGKDLPGIQALGGVEGVAASLRTNPTKGIHGNEQEVSRRRDLFGSNTYHKPPPKGLLFFVYEAFKDLTILILLVCAIFSLGFGIKEHGIKEGWYEGGSIFVAVFLVIVVSALSNFRQERQFDKLSKISNNIKVEVLRDSRRQHISIFDVVVGDVVFLKIGDQIPADGLFLEGHSLQVDESSMTGESDHLEVDHKDNPFLFSGTKIVDGFAQMLVVSVGMSTTWGQTMSSINQDSSERTPLQVRLDTLTSTIGKIGLTVAALVLVVLLVRYFTGNTEKEGKREYNGSKTPVDTVVNSVVRIVAAAVTIVVVAIPEGLPLAVTLTLAYSMKRMMSDQAMVRKLSACETMGSATVICTDKTGTLTLNEMKVTKFWLGQESIHEDSTKMISPDVLDLLYQGTGLNTTGSVCVSDSGSTPEFSGSPTEKALLSWTVLNLGMDMESVKQKHEVLRVETFSSAKKRSGVLVRRKSDNTVHVHWKGAAEMVLAMCSHYYTSTGSVDLMDSTAKSRIQAIIQGMAASSLRCIAFAHKIASNDSVLEEDGLTLMGIVGLKDPCRPGVSKAVETCKLAGVTIKMITGDNVFTAKAIAFECGILDHNDKDEEDAVVEGVQFRNYTDEERMQKVDKIRVMARSSPSDKLLMVKCLRLKGHVVAVTGDGTNDAPALKEADIGLSMGIQGTEVAKESSDIVILDDNFASVATVLKWGRCVYNNIQKFIQFQLTVNVAALVINFIAAISAGEVPLTAVQLLWVNLIMDTLGALALATERPTNELLKRKPVGRTEALITNVMWRNLLVQSLYQIAVLLILQFKGMSIFSVRKEVKDTLIFNTFVLCQVFNEFNAREMEKKNVFKGLHRNRLFIGIIAITIVLQVIMVEFLKKFADTVRLNGWQWGTCIALASLSWPIGFFTKFIPVSETPFLSYFKNPRSLFKGSRSPSLKKP

>AtALA1

MDPRKSIDKPPHHDPILGVSSRWSVSSKDNKEVTFGDLGSKRIRHGSAGADSEMLSMSQKEIKDEDARLIYINDPDRTNERFEFTGNSIKTAKYSVFTFLPRNLFEQFHRVAYIYFLVIAVLNQLPQLAVFGRGASIMPLAFVLLVSAIKDAYEDFRRHRSDRVENNRLALVFEDHQFREKKWKHIRVGEVIKVQSNQTLPCDMVLLATSDPTGVVYVQTTNLDGESNLKTRYAKQETLLKAADMESFNGFIKCEKPNRNIYGFQANMEIDGRRLSLGPSNIILRGCELKNTAWALGVVVYAGGETKAMLNNSGAPSKRSRLETRMNLEIILLSLFLIVLCTIAAATAAVWLRTHRDDLDTILFYRRKDYSERPGGKNYKYYGWGWEIFFTFFMAVIVYQIMIPISLYISMELVRIGQAYFMTNDDQMYDESSDSSFQCRALNINEDLGQIKYLFSDKTGTLTDNKMEFQCACIEGVDYSDREPADSEHPGYSIEVDGIILKPKMRVRVDPVLLQLTKTGKATEEAKRANEFFLSLAACNTIVPIVSNTSDPNVKLVDYQGESPDEQALVYAAAAYGFLLIERTSGHIVINVRGETQRFNVLGLHEFDSDRKRMSVILGCPDMSVKLFVKGADSSMFGVMDESYGGVIHETKIQLHAYSSDGLRTLVVGMRELNDSEFEQWHSSFEAASTALIGRAGLLRKVAGNIETNLRIVGATAIEDKLQRGVPEAIESLRIAGIKVWVLTGDKQETAISIGFSSRLLTRNMRQIVINSNSLDSCRRSLEEANASIASNDESDNVALIIDGTSLIYVLDNDLEDVLFQVACKCSAILCCRVAPFQKAGIVALVKNRTSDMTLAIGDGANDVSMIQMADVGVGISGQEGRQAVMASDFAMGQFRFLVPLLLVHGHWNYQRMGYMILYNFYRNAVFVLILFWYVLFTCYTLTTAITEWSSVLYSVIYTAIPTIIIGILDKDLGRQTLLDHPQLYGVGQRAEGYSTTLFWYTMIDTIWQSAAIFFIPMFAYWGSTIDTSSLGDLWTIAAVVVVNLHLAMDVIRWNWITHAAIWGSIVAACICVIVIDVIPTLPGYWAIFQVGKTWMFWFCLLAIVVTSLLPRFAIKFLVEYYRPSDVRIAREAEKLGTFRESQPVGVEMNLIQDPPRR

>AtALA2

MNLEFCFLQAYSILRDFRRNDKLIDVFEFLPDLDRFVYINDDEASKELCCDNRISNRKYTLWNFLPKNLWEQFSRFMNQYFLLIACLQLWSLITPVNPASTWGPLIFIFAVSASKEAWDDYHRYLSDKKANEKEVWIVKQGIKKHIQAQDIQVGNIVWLRENDEVPCDLVLLGTSDPQGVCYVETAALDGETDLKTRVIPSACVGIDLELLHKMKGVIECPVPDKDIRRFDANMRLFPPFIDNDVCSLTIKNTLLQSCYLRNTEWACGVSVYTGNQTKLGMSRGIAEPKLTAMDAMIDKLTGAIFVFQIVVVLVLGIAGNVWKDTEARKQWYVQYPEEAPWYELLVIPLRFELLCSIMIPISIKVSLDLVKGLYAKFIEWDVEMIDQETGTASYAANTAISEDLGQVEYILTDKTGTLTDNKMIFRRCCIGGIFYGNENGDALKDAQLLNAITSGSTDVIRFLTVMAICNTVLPVQSKAGDIVYKAQSQDEDALVIAASKLHMVFVGKNANLLEIRFNGSVIRYEVLEILEFTSDRKRMSVVVKDCQNGKIILLSKGADEAILPYARAGQQTRTIGDAVEHYSQLGLRTLCLAWRELEENEYLEWSVKFKEASSLLVDREWRIAEVCQRLEHDLYILGVTAIEDRLQDGVPETIETLRKAGINFWMLTGDKQNTAIQIALSCNFISPEPKGQLLMIDGKTEEDVSRSLERVLLTMRITASEPKDVAFVIDGWALEIALKHHRKDFVELAILSRTAICCRVTPSQKAQLVEILKSCDYRTLAIGDGGNDVRMIQQADIGVGISGREGLQAARAADYSIGRFRFLKRLILVHGRYSYNRTAFLSQYSFYKSLLICFIQIFFSFISGVSGTSLFNSVSLMAYNVFYTSVPVLVSVIDKDLSEASVMQHPQILFYCQAGRLLNPSTFAGWFGRSLFHAIIVFVITIHAYAYEKSEMEELGMVALSGCIWLQAFVVAQETNSFTVLQHLSIWGNLVGFYAINFLFSAIPSSGMYTIMFRLCSQPSYWITMFLIVGAGMGPIFALKYFRYTYRPSKINILQQAERMGGPILTLGNIETQPRTIEKDLSPISITQPKNRSPVYEPLLSDSPNATRRSFGPGTPFEFFQSQSRLSSSSGYTRNCKDN

>AtALA3

MVRSGSFSVDSSATHQRTPSRTVTLGHIQPQAPTYRTVYCNDRESNQPVRFKGNSISTTKYNVFTFLPKGLFEQFRRIANIYFLGISCLSMTPISPVSPITNVAPLSMVLLVSLIKEAFEDWKRFQNDMSINNSTVEILQDQQWVSIPWRKLQVGDIVKIKKDGFFPADILFMSSTNSDGICYVETANLDGETNLKIRKALERTWDYLVPEKAYEFKGEIQCEQPNNSLYTFTGNLVVQKQTLPLSPDQLLLRGCSLRNTEYIVGAVVFTGHETKVMMNAMNAPSKRSTLEKKLDKLIITIFCVLVTMCLIGAIGCSIVTDREDKYLGLHNSDWEYRNGLMIGFFTFFTLVTLFSSIIPISLYVSIEMIKFIQSTQFINRDLNMYHAETNTPASARTSNLNEELGQVEYIFSDKTGTLTRNLMEFFKCSIGGVSYGCGVTEIEKGIAQRHGLKVQEEQRSTGAIREKGFNFDDPRLMRGAWRNEPNPDLCKELFRCLAICHTVLPEGDESPEKIVYQAASPDEAALVTAAKNFGFFFYRRTPTMVYVRESHVEKMGKIQDVAYEILNVLEFNSTRKRQSVVCRFPDGRLVLYCKGADNVIFERLANGMDDVRKVTREHLEHFGSSGLRTLCLAYKDLNPETYDSWNEKFIQAKSALRDREKKLDEVAELIEKDLILIGSTAIEDKLQEGVPTCIETLSRAGIKIWVLTGDKMETAINIAYACNLINNEMKQFVISSETDAIREAEERGDQVEIARVIKEEVKRELKKSLEEAQHSLHTVAGPKLSLVIDGKCLMYALDPSLRVMLLSLSLNCTSVVCCRVSPLQKAQVTSLVRKGAQKITLSIGDGANDVSMIQAAHVGIGISGMEGMQAVMASDFAIAQFRFLTDLLLVHGRWSYLRICKVVMYFFYKNLTFTLTQFWFTFRTGFSGQRFYDDWFQSLFNVVFTALPVIVLGLFEKDVSASLSKRYPELYREGIRNSFFKWRVVAVWATSAVYQSLVCYLFVTTSSFGAVNSSGKVFGLWDVSTMVFTCLVIAVNVRILLMSNSITRWHYITVGGSILAWLVFAFVYCGIMTPHDRNENVYFVIYVLMSTFYFYFTLLLVPIVSLLGDFIFQGVERWFFPYDYQIVQEIHRHESDASKADQLEVENELTPQEARSYAISQLPRELSKHTGFAFDSPGYESFFASQLGIYAPQKAWDVARRASMRSRPKVPKK

>AtALA4

MARGRIRSKLRLSHIYTFGCLRPSADEGQDPHPIQGPGFSRTVYCNQPHMHKKKPLKYRSNYVSTTRYNLITFFPKCLYEQFHRAANFYFLVAAILSVFPLSPFNKWSMIAPLVFVVGLSMLKEALEDWSRFMQDVKINASKVYVHKSDGEFRRRKWKKISVGDIVKVEKDGFFPADLLLLSSSYEDGICYVETMNLDGETNLKVKRSLEVTLSLDDYDSFKDFTGIIRCEDPNPSLYTFVGNLEYERQIFPLDPSQILLRDSKLRNTPYVYGVVVFTGHDTKVMQNSTKSPSKRSRIEKTMDYIIYTLLVLLILISCISSSGFAWETKFHMPKWWYLRPEEPENLTNPSNPVYAGFVHLITALLLYGYLIPISLYVSIEVVKVLQASFINKDLHMYDSESGVPAHARTSNLNEELGQVDTILSDKTGTLTCNQMDFLKCSIAGTSYGVRSSEVEVAAAQQMAVDLDEHGEVSSRTSTPRAQARDIEVESSITPRIPIKGFGFEDIRLMDGNWLREPHTDDILLFFRILAICHTAIPELNEETGKYTYEAESPDEASFLTAASEFGFVFFKRTQSSVYVHERLSHSGQTIEREYKVLNLLDFTSKRKRMSVVVRDEEGQILLLCKGADSIIFERLAKNGKVYLGPTTKHLNEYGEAGLRTLALSYRKLDEEEYSAWNAEFHKAKTSIGSDRDELLERISDMIEKDLILVGATAVEDKLQKGVPQCIDKLAQAGLKLWVLTGDKMETAINIGYSCSLLRQGMKQICITVVNSEGASQDAKAVKDNILNQITKAVQMVKLEKDPHAAFALIIDGKTLTYALEDEMKYQFLALAVDCASVICCRVSPKQKALVTRLVKEGTGKITLAIGDGANDVGMIQEADIGVGISGVEGMQAVMASDFSIAQFRFLERLLVVHGHWCYKRIAQMICYFFYKNIAFGLTLFYFEAFTGFSGQSVYNDYYLLLFNVVLTSLPVIALGVFEQDVSSEICLQFPALYQQGKKNLFFDWYRILGWMGNGVYSSLVIFFLNIGIIYEQAFRVSGQTADMDAVGTTMFTCIIWAVNVQIALTVSHFTWIQHVLIWGSIGLWYLFVALYGMMPPSLSGNIYRILVEILAPAPIYWIATFLVTVTTVLPYFAHISFQRFLHPLDHHIIQEIKYYKRDVEDRRMWTRERTKAREKTKIGFTARVDAKIRHLRSKLNKKQSNMSQFSTQDTMSPRSV

>AtALA5

MARGRIRSKLRLSLLYTFGCLRPATLEGQDSQPIQGPGFSRTVFCNQPHMHKKKPLRYRSNYVSTTRYNLITFFPKSLYEQFHRAANLYFLVAAILSVFPLSPFNKWSMIAPLVFVVGLSMLKEALEDWRRFMQDVKINARKTCVHKSDGVFRQRKWKKVSVGDIVKVEKDEFFPADLLLLSSSYEDGICYVETMNLDGETNLKVKRSLEVSLPLDDDESFKNFMATIRCEDPNPNLYTFVGNLEFERQTFPLDPSQILLRDSKLRNTTYVYGVVVFTGFDTKVMQNSTKSPSKRSRIERTMDYIIYTLLVLLILISCISSSGFAWETEFHMPKMWYLRPGEPIDFTNPINPIYAGVVHLITALLLYGYLIPISLYVSIEVVKVWQASFINQDLHMYDDESGVPANARTSNLNEELGQVHTILSDKTGTLTCNQMDFLKCSIAGTSYGVRSSEVEVAAAKQMAVDLEEHGEISSTPQSQTKVYGTWDSSRTQEIEVEGDNNYNTPRAPIKGFGFEDNRLMNGNWLRESQPNDILQFFRILAICHTAIPELNEETGKYTYEAESPDEASFLAAAREFGFEFFKRTQSSVFIRERFSGSGQIIEREYKVLNLLEFTSKRKRMTVIVRDEEGQILLLCKGADSIIFERLAKNGKTYLGPTTRHLTEYGEAGLRTLALAYRKLDEDEYAAWNSEFLKAKTSIGSDRDELLETGADMIEKELILIGATAVEDKLQKGVPQCIDKLAQAGLKLWVLTGDKMETAINIGFACSLLRQGMRQICITSMNSEGGSQDSKRVVKENILNQLTKAVQMVKLEKDPHAAFALIIDGKTLTYALEDDMKYQFLALAVDCASVICCRVSPKQKALVVRLVKEGTGKTTLAIGDGANDVGMIQEADIGVGISGVEGMQAVMASDFSIAQFRFLERLLVVHGHWCYKRIAQMICYFFYKNIAFGLTLFYFEAFTGFSGQSVYNDYYLLLFNVVLTSLPVIALGVFEQDVSSEICLQFPALYQQGTKNLFFDWSRILGWMCNGVYASLVIFFLNIGIIYSQAFRDNGQTADMDAVGTTMFTCIIWAANVQIALTMSHFTWIQHVLIWGSIGMWYLFVAIYSMMPPSYSGNIYRILDEILAPAPIYWMATLLVTVAAVLPYVAHIAFQRFLNPLDHHIIQEIKYYGRDIEDARLWTRERTKAREKTKIGFTARVDAKIRHLRSKLNKKQSNLSHFSAQDAMSPRSL

>AtALA6

MARRRIRSRIRKSHFYTFRCLRPKTLDDQGPHVINGPGYTRIVHCNQPHLHLATKLIRYRSNYVSTTRYNLLTFLPKCLYEQFHRVANFYFLVAAILSVFPLSPFNKWSMIAPLVFVVGLSMGKEALEDWRRFMQDVEVNSRKASVHKGSGDFGRRTWKRIRVGDIVRVEKDEFFPADLLLLSSSYEDGICYVETMNLDGETNLKVKRCLDATLALEKDESFQNFSGTIKCEDPNPNLYTFVGNLECDGQVYPLDPNQILLRDSKLRNTAYVYGVVVFTGHDTKVMQNSTKSPSKRSRIEKRMDYIIYTLFALLLTVSFISSLGFAVMTKLLMAEWWYLRPDKPESLTNPTNPLYAWVVHLITALLLYGYLIPISLYVSIEVVKVLQAHFINQDLQLYDSESGTPAQARTSNLNEELGQVDTILSDKTGTLTCNQMDFLKCSIAGTSYGVRASEVELAAAKQMAMDLEEKGEEVANLSMNKGRTQRYAKLASKTSSDFELETVVTASDEKDQKQNTGVKGFSFEDNRLMNENWLNEPNSDDILMFFRILAVCHTAIPEVDEDTGMCTYEAESPDEVAFLVASREFGFEFTKRTQSSVFIAERFSSSGQPVDREYKILNLLDFTSKRKRMSAIVRDEEGQILLLCKGADSIIFERLSKSGKEYLGATSKHLNVYGEAGLRTLALGYRKLDETEYAAWNSEFHKAKTSVGADRDEMLEKVSDMMEKELILVGATAVEDKLQKGVPQCIDNLAQAGLKIWVLTGDKMETAINIGYACSLLRQGMKQISISLTNVEESSQNSEAAAKESILMQITNASQMIKIEKDPHAAFALIIDGKTLTYALKDDVKYQFLALAVDCASVICCRVSPKQKALVTRLAKEGTGKTTLAIGDGANDVGMIQEADIGVGISGVEGMQAVMASDFSIAQFRFLERLLVVHGHWCYKRIAQMICYFFYKNITFGLTLFYFECFTGFSGQSIYNDSYLLLFNVVLTSLPVISLGVFEQDVPSDVCLQFPALYQQGPKNLFFDWYRILGWMGNGVYASIVIFTLNLGIFHVQSFRSDGQTADMNAMGTAMFTCIIWAVNVQIALTMSHFTWIQHVMIWGSIGAWYVFLALYGMLPVKLSGNIFHMLVEILAPAPIFWLTSLLVIAATTLPYLFHISYQRSVNPLDHHIIQEIKHFRIDVEDERMWKREKSKAREKTKIGFTARVDAKIRQLRGRLQRKHSVLSVMSGTSSNDTPSSNSQ

>AtALA7

MGRRRIRSRIRKSHFYTFKCLRPKTLEDQGPHIINGPGYTRIVHCNQPHLHLAKVLRYTSNYVSTTRYNLITFLPKCLYEQFHRVANFYFLVAAILSVFPLSPFNKWSMIAPLIFVVGLSMGKEALEDWRRFMQDVKVNSRKATVHRGDGDFGRRKWKKLRVGDVVKVEKDQFFPADLLLLSSSYEDGICYVETMNLDGETNLKVKRCLDVTLPLERDDTFQSFSGTIKCEDPNPNLYTFVGNLEYDGQVYPLDPSQILLRDSKLRNTSYVYGVVVFTGHDTKVMQNSTKSPSKRSRIEKRMDYIIYTLFALLVLVSFISSLGFAVMTKMHMGDWWYLRPDKPERLTNPRNPFHAWVVHLITAVLLYGYLIPISLYVSIELVKVLQATFINQDLQMYDSESGTPAQARTSNLNEELGQVDTILSDKTGTLTCNQMDFLKCSIAGTSYGVRASEVELAAAKQMAIDLDEEQGEEVTHLPRTRGRMHGYAKMPSKTSSDIELETVITATDEGDQTQSTGIKGFSFEDQRLMGGNWLNEPNSDDILMFLRILAVCHTAIPEVDEDTGKCTYEAESPDEVAFLVAAGEFGFEFTKRTQSSVFISERHSGQPVEREYKVLNVLDFTSKRKRMSVIVRDEKGQILLLCKGADSIIFERLSKNGKNYLEATSKHLNGYGEAGLRTLALSYRKLDETEYSIWNSEFHKAKTSVGADRDEMLEKVSDMMEKELILVGATAVEDKLQKGVPQCIDKLAQAGLKIWVLTGDKMETAINIGYACSLLRQGMKQIYIALRNEEGSSQDPEAAARENILMQIINASQMIKLEKDPHAAFALIIDGKTLTYALEDDIKYQFLALAVDCASVICCRVSPKQKALVTRLAKEGTGKTTLAIGDGANDVGMIQEADIGVGISGVEGMQAVMASDFSIAQFRFLERLLVVHGHWCYKRIAQMICYFFYKNITFGLTLFYFEAFTGFSGQAIYNDSYLLLFNVILTSLPVIALGVFEQDVSSEVCLQFPALYQQGPKNLFFDWYRIIGWMANGVYASVVIFSLNIGIFHVQSFCSGGQTADMDAMGTAMFTCIIWAVNVQIALTMSHFTWIQHVLIWGSIVTWYIFLALFGMLPPKVSGNIFHMLSETLAPAPIFWLTSLLVIAATTLPYLAYISFQRSLNPLDHHIIQEIKHFRIDVQDECMWTRERSKAREKTKIGVTARVDAKIRQLRGRLQRKHSILSVMSGLSGVSASTDTTSTTQHS

>AtALA8

MAGERRKGMKFSKLYSFKCFKPFSREDHSQIGSRGYSRVVFCNDPDNPEALQLNYRGNYVSTTKYTAANFIPKSLFEQFRRVANIYFLVVAFVSFSPLAPYTAPSVLAPLLIVIGATMVKEGVEDLRRRKQDVEANNRKVEVLGKTGTFVETKWKNLRVGDLVKVHKDEYFPADLLLLSSSYEDGICYVETMNLDGETNLKLKHALEITSDEESIKNFRGMIKCEDPNEHLYSFVGTLYFEGKQYPLSPQQILLRDSKLKNTDYVYGVVVFTGHDTKVMQNATDPPSKRSKIEKKMDQIIYILFSILIVIAFTGSVFFGIATRRDMSDNGKLRRWYLRPDHTTVFYDPRRAVAAAFFHFLTALMLYGYLIPISLYVSIEVVKVLQSIFINQDQEMYHEETDRPARARTSNLNEELGQVDTILSDKTGTLTCNSMEFVKCSIAGTAYGRGMTEVEVALRKQKGLMTQEEVGDNESLSIKEQKAVKGFNFWDERIVDGQWINQPNAELIQKFFRVLAICHTAIPDVNSDTGEITYEAESPDEAAFVIASRELGFEFFSRSQTSISLHEIDHMTGEKVDRVYELLHVLEFSSSRKRMSVIVRNPENRLLLLSKGADSVMFKRLAKHGRQNERETKEHIKKYAEAGLRTLVITYREIDEDEYIVWEEEFLNAKTLVTEDRDALIDAAADKIEKDLILLGSTAVEDKLQKGVPDCIEKLSQAGVKIWVLTGDKTETAINIGYACSLLREGMKQILVTLDSSDIEALEKQGDKEAVAKASFQSIKKQLREGMSQTAAVTDNSAKENSEMFGLVIDGKSLTYALDSKLEKEFLELAIRCNSVICCRSSPKQKALVTRLVKNGTGRTTLAIGDGANDVGMLQEADIGVGISGAEGMQAVMASDFAIAQFRFLERLLLVHGHWCYRRITLMICYFFYKNLAFGFTLFWYEAYASFSGKPAYNDWYMSCYNVFFTSLPVIALGVFDQDVSARLCLKYPLLYQEGVQNVLFSWERILGWMLNGVISSMIIFFLTINTMATQAFRKDGQVVDYSVLGVTMYSSVVWTVNCQMAISINYFTWIQHCFIWGSIGVWYLFLVIYGSLPPTFSTTAFQVFVETSAPSPIYWLVLFLVVFSALLPYFTYRAFQIKFRPMYHDIIVEQRRTERTETAPNAVLGELPVQVEFTLHHLRANLSRRDSWN

>AtALA9

MVGGGTKRRRRRLQLSKLYTLTCAQACFKQDHSQIGGPGFSRVVYCNEPDSPEADSRNYSDNYVRTTKYTLATFLPKSLFEQFRRVANFYFLVTGVLAFTPLAPYTASSAIVPLLFVIGATMVKEGVEDWRRQKQDNEVNNRKVKVHRGDGSFDAKEWKTLSIGDIVKVEKNEFFPADLVLLSSSYEDAICYVETMNLDGETNLKVKQGLEVTSSLRDEFNFKGFEAFVKCEDPNANLYSFVGTMELKGAKYPLSPQQLLLRDSKLRNTDFIFGAVIFTGHDTKVIQNSTDPPSKRSMIEKKMDKIIYLMFFMVITMAFIGSVIFGVTTRDDLKDGVMKRWYLRPDSSSIFFDPKRAPVAAIYHFLTAVMLYSYFIPISLYVSIEIVKVLQSIFINQDIHMYYEEADKPARARTSNLNEELGQVDTILSDKTGTLTCNSMEFIKCSVAGTAYGRGVTEVEMAMGRRKGGPLVFQSDENDIDMEYSKEAITEESTVKGFNFRDERIMNGNWVTETHADVIQKFFRLLAVCHTVIPEVDEDTEKISYEAESPDEAAFVIAARELGFEFFNRTQTTISVRELDLVSGKRVERLYKVLNVLEFNSTRKRMSVIVQEEDGKLLLLCKGADNVMFERLSKNGREFEEETRDHVNEYADAGLRTLILAYRELDEKEYKVFNERISEAKSSVSADRESLIEEVTEKIEKDLILLGATAVEDKLQNGVPDCIDKLAQAGIKIWVLTGDKMETAINIGFACSLLRQDMKQIIINLETPEIQSLEKTGEKDVIAKASKENVLSQIINGKTQLKYSGGNAFALIIDGKSLAYALDDDIKHIFLELAVSCASVICCRSSPKQKALVTRLVKSGNGKTTLAIGDGANDVGMLQEADIGVGISGVEGMQAVMSSDIAIAQFRYLERLLLVHGHWCYRRISTMICYFFYKNITFGFTLFLYETYTTFSSTPAYNDWFLSLYNVFFSSLPVIALGVFDQDVSARYCLKFPLLYQEGVQNVLFSWRRILGWMFNGFYSAVIIFFLCKSSLQSQAFNHDGKTPGREILGGTMYTCIVWVVNLQMALAISYFTLIQHIVIWSSIVVWYFFITVYGELPSRISTGAYKVFVEALAPSLSYWLITLFVVVATLMPYFIYSALQMSFFPMYHGMIQWLRYEGQCNDPEYCDIVRQRSIRPTTVGFTARLEAKKRSVRISEPAS

>AtALA10

MAGPSRRRRRLHLSKIYSYTCGKSSFQEDHSNIGGPGFSRVVYCNEPGSPAAERRNYAGNYVRSTKYTVASFFPKSLFEQFRRVANFYFLVTGILSLTDLSPYGAVSALLPLALVISATMVKEGIEDWRRKQQDIEVNNRKVKVHDGNGIFRQEEWRNLRVGDIVRVEKDEFFPADLLLLSSSYEDSVCYVETMNLDGETNLKVKQGLEATSSLLNQDSDFKDFRGVVRCEDPNVNLYVFVGTLALEEERFPLSIQQILLRDSKLRNTEYVYGAVVFTGHDTKVIQNSTDPPSKRSRIERTMDKIIYLMFGLVFLMSFVGSIIFGVETREDKVKNGRTERWYLKPDDADIFFDPERAPMAAIYHFFTATMLYSYFIPISLYVSIEIVKVLQSIFINRDIHMYYEETDKPAQARTSNLNEELGMVDTILSDKTGTLTCNSMEFIKCSIAGKAYGRGITEVERAMAVRSGGSPLVNEDLDVVVDQSGPKVKGFNFEDERVMNGNWVRQPEAAVLQKFFRLLAVCHTAIPETDEESGNVSYEAESPDEAAFVVAAREFGFEFFNRTQNGISFRELDLVSGEKVERVYRLLNVLEFNSTRKRMSVIVRDDDGKLLLLSKGADNVMFERLAKNGRQFEAKTQEHVNQYADAGLRTLVLAYREVDENEYIEFNKSFNEAKASVSEDREALIDEITDKMERDLILLGATAVEDKLQNGVPECIDKLAQAGIKIWVLTGDKMETAINIGFASSLLRQEMKQIIINLETPQIKSLEKSGGKDEIELASRESVVMQLQEGKALLAASGASSEAFALIIDGKSLTYALEDEIKKMFLDLATSCASVICCRSSPKQKALVTRLVKSGTGKTTLAIGDGANDVGMLQEADIGVGISGVEGMQAVMSSDIAIAQFRYLERLLLVHGHWCYSRIASMICYFFYKNITFGVTVFLYEAYTSFSGQPAYNDWFLSLFNVFFSSLPVIALGVFDQDVSARFCYKFPLLYQEGVQNILFSWKRIIGWMFNGFISALAIFFLCKESLKHQLFDPDGKTAGREILGGTMYTCVVWVVNLQMALSISYFTWVQHIVIWGSIAFWYIFLMIYGAMTPSFSTDAYMVFLEALAPAPSYWLTTLFVMIFALIPYFVYKSVQMRFFPKYHQMIQWIRYEGHSNDPEFVEMVRQRSIRPTTVGYTARRAASVRRSARFHDQIYKDLVGV

>AtALA11

MTKCRRRRLHLSNIYAFKGRKSNFQEDHSHIGGPGFSRVVYCNEPNSPAAERRNYVGNYVRSTKYTLASFIPKSLFEQFRRVANFYFLVTGVLSLTALSPYSPISALLPLTFVIAASMVKEAIEDWGRKKQDIEMNNRKVKVHDGNGIFRREGWRDLKVGNIVRVEKDEFFPADLLLLSSSYEDSICYVETMNLDGETNLKVKQGLEATSSALHEDSDFKELKAVVKCEDPNADLYTFVGTLHFEEQRLPLSITQLLLRDSKLRNTEYIYGVVVFTGHDTKVIQNSTDPPSKRSRIERKMDKIIYLMFGVVFLMSFIGSIVFGIETREDRVRNGGRTERWYLRPDNADIFFDPDRAPMAAVYHFFTAVMLYSYFIPISLYVSIEIVKVLQSLFINNDILMYYEENDKPAHARTSNLNEELGMVDTILSDKTGTLTCNSMEFIKCSIAGTAYGRGITEVERSMAMRSNGSSLVGDDLDVVVDQSGPKIKGFNFLDERVMKGNWVKQRDAAVLQKFFRLLAVCHTAIPETDEATGSVSYEAESPDEAAFVVAAREFGFEFFSRTQNGISFRELDLASGKTVERVYRLLNVLEFNSARKRMSVIVRDEDGRLLLLSKGADNVMFERLAKNGRKFEEKTREHVNEYADAGLRTLILAYREVDENEYIEFSKNFNEAKNSVTADRESLIDEITEQMERDLILLGATAVEDKLQNGVPDCIDKLAQAGIKIWVLTGDKMETAINIGFACSLLRQEMKQIIINLETPHIKALEKAGEKDAIEHASRESVVNQMEEGKALLTASSSASSHEAFALIIDGKSLTYALEDDFKKKFLDLATGCASVICCRSSPKQKALVTRLVKSGTGKTTLAIGDGANDVGMLQEADIGVGISGVEGMQAVMSSDIAIAQFRYLERLLLVHGHWCYSRISSMICYFFYKNITFGVTVFLYEAYTSFSAQPAYNDWFLSLFNVFFSSLPVIALGVFDQDVSARYCYKFPLLYQEGVQNLLFSWKRIIGWMFNGVFTALAIFFLCKESLKHQLYNPNGKTAGREILGGTMYTCVVWVVNLQMALAISYFTWLQHIVIWGSVAFWYIFLMIYGAITPSFSTDAYKVFIEALAPAPSYWLTTLFVMFFALIPFFVFKSVQMRFFPGYHQMIQWIRYEGHSNDPEFVEMVRQRSIRPTTVGFTARRAASVRRSGRFHDQLNKNFIAF

>AtALA12

MATVSGRRRKRKIQLSKLFTLTGAKACFKPDHSKIGRSGFSRVVFCNQPDSPEAESRNYCDNYVRTTKYTLATFLPKSLFEQFRRVANFYFLVVGILSFTPLAPYTAVSAIVPLTFVILATMFKEGVEDWRRKQQDIEVNNRKVRVHRGNGNFDLREWKTLRVGDILKVEKNEFFPADLVLLSSSYEDAVCYVETMNLDGETNLKLKQGLEVTLSLREELNFRDFEAFIKCEDPNANLYSFVGTMDLKGEKYPLSPQQLLLRGSKLRNTDYIYGVVIFTGPDTKVVQNSTDPPSKRSMIERKMDKIIYLMFLMVFSLAFFGSVLFGIWTRDDFQNGVMERWYLKPDDSSIFFDPKRAPMAAIYHFLTALMLNSYFIPISLYVSIEIVKVLQSIFINQDIHMYYEEADKPAHARTSNLNEELGQVGTILSDKTGTLTCNSMEFIKCSIAGTAYGRGVTEVEMAMDKRKGSALVNQSNGNSTEDAVAAEPAVKGFNFRDERIMDGNWVTETHADVIQKFFQLLAVCHTVIPEVDEDTGKISYEAESPDEAAFVIAARELGFEFFTRTQTTISVRELDLVTGERVERLYSVLNVLEFSSSKKRMSVIVQDQDGKLLLLCKGADSVMFERLSESGRKYEKETRDHVNEYADAGLRTLILAYRELDENEYEVFTERISEAKNSVSADREALIDEVTEKIEKNLVLLGATAVEDKLQNGVPDCINKLAQAGIKIWVLTGDKMETAINIGFACSLLRRDMKQIIINLETPEIQQLEKSGEKDAIAALKENVLHQITSGKAQLKASGGNAKAFALIIDGKSLAYALEEDMKGIFLELAIGCASVICCRSSPKQKALVTRLVKTGSGQTTLAIGDGANDVGMLQEADIGVGISGVEGMQAVMSSDIAIAQFRYLERLLLVHGHWCYRRISKMICYFFYKNITFGFTLFLYEAYTSFSATPAYNDWYLSLYSVFFTSLPVICLGIFDQDVSAPFCLKFPVLYQEGVQNLLFSWRRILSWMFHGFCSAIIIFFLCKTSLESQAFNHEGKTAGRDILGGTMYTCVVWVVSLQMVLTISYFTLIQHVVVWGSVVIWYLFLMVYGSLPIRMSTDAYMVFLEALAPAPSYWITTLFVVLSTMMPYFIFSAIQMRFFPMSHGTVQLLRYEDQCSNSGNFEMGRQGSVRPTLVMRSHQPES

>AtP5

MSSFRVGGKVVEKVDLCRKKQLVWRLDVWPFAILYTVWLTTIVPSIDFSDACIALGGLSAFHILVLLFTTWSVDFKCFVQFSKVNSIDQADACKVTPAKFSGSKEVVPLHFRSQMTDSASSGDMEEIFFDFRKQRFIYSKELGAFSKLPYPTKETFGHYLKCTGHGTEAKIATATEKWGRNVFDYPQPTFQKLMKENCMEPFFVFQVFCVGLWCLDEFWYYSVFTLFMLFMFESTMAKSRLKTLTDLRSVRVDSQTVMVYRSGKWVKLLGTDLLPGDVVSIGRPSTQTGGEDKTVPADMLLLVGSAIVNEAILTGESTPQWKVPIVGQRSDEKLSIKRNKNHVLFGGTKILQHSPDKSFSLKTPDGGCLAVVLRTGFETSQGKLMRTILFSTERVTANSWESGLFILFLVVFAVIAAGYVLVKGLEDPTRSKYKLLLGCSLIITSVIPPELPMELSIAVNTSLLALVRRGIFCTEPFRIPFAGKVDLCCFDKTGTLTSDDMEFRGVGGLSNCEEAETDMSKVPVRTLEILASCHALVFVENKLVGDPLEKAALKGIDWSYKADEKALPRRGNGNSVQIMQRYHFASHLKRMSVIVRIQEEYLAFVKGAPETIQERLVDVPAQYIETYKRYTRQGSRVLALAYKRLPDMMVSEARDMDRDAVESDLTFAGFAVFNCPIRPDSAPVLLELKNSSHDLVMITGDQALTACHVAGQVHIVSNPVLILGRSGSGNEYKWVSPDEKEIIPYSEKEIETLAETHDLCIGGDSIEMLQATSAVLRVIPFVKVFARVAPQQKELILTTFKAVGRGTLMCGDGTNDVGALKQAHVGVALLNNKLPLSPSDSSKDDKSKSKKSKLPLEPASKTITQNGEGSSKGKIPPQNRHLTAAELQRQKLKKIMDDLNNDEGDGRSAPLVKLGDASMASPFTAKHASVAPVTDIIRQGRSTLVTTLQMFKILGLNCLATAYVLSVMYLDGVKLGDVQATISGVLTAAFFLFISHARPLQTLSAERPHPSVFSVYLFLSLIGQFAVHLTFLVYSVKEAEKHMPEECIEPDASFHPNLVNTVSYMVSMMLQVATFAVNYMGHPFNQSIRENKPFFYALIAGAGFFTVIASDLFRDLNDSLKLVPLPQGLRDKLLIWASLMFIICYSWERLLRWAFPGKISSWKHKQRAVTANLEKKKKV

>GaAHA11

MDTEKKAVALEAINKEIVDLENIPIEEVLEKLKCTKEGLTTDEVQQRLEL

FGYNKLEEKKENKILKFLGFMWNPLSWVMEAAAVMAIALAHGGKKETDYH

DFVGILALLLINSTISFIEENNAGNAAAALMARLAPKAKVLRDGKWNEED

ASVLVPGDIISIKLGDIIPADARLLQGDPLKIDQSALTGESLPVTKHPGD

GVYSGSTCKQGEIEAVVIATGVHTFFGKAAHLVESTTHVGHFQQVLTSIG

NFCICSIAIGMLAELIVIYGAQRRSYRTGIDNLLVILIGGIPIAMPTVLS

VTMAIGSHRLSQQGAITKRMTAIEEMAGMDVLCSDKTGTLTLNKLTVDKN

LIEVFVNNVDKDTVILMAARASRLENQDAIDTAIVAMLADPKEARAGITE

VHFLPFNPTDKRTALTYIDEAGKMHRVSKGAPEQILNLAYNKSEIGKKVH

SIIDKYAERGLRSLAVARQEVPAGTKDSPGGPWEFVGLLPLFDPPRHDSA

ETIRRALDLGVSVKMITGDQLAIGKETGRRLGMGTNMYPSSSLLGDHKNE

EIGAFSIDELIENADGFAGVFPEHKFEIVKRLQAKKHIVGMTGDGVNDAP

ALKKADIGIAVADSTDAARSASDIVLTEPGLSVIISAVLTSRAIFQRMKN

YTIYAVSITIRIVLGFMLLTLIWRLNFPPFMVLIIAILNDGTIMTISKDR

VKPSPVPDSWKLKEIFATGIVLGSYLALMTVIFFWAAYETDFFPDKFGVR

SLSKKSFDLKQESERRKVNALLSSAVYLQVSTISQALIFVTRSRGWSFTE

RPGLLLVTAFIIAQLVATVISAQATWSFAGIRAVGWGWCGVIWIYNILTY

FLLDPIKFAVRYALSGKAWDLVLNQRTAFSTQKDFGKEAREAAWAAEQRT

LHGLQSTSDAKITEKHNFRDISVMAEEARRRAEIARLREIHTLKGKVESF

AKLRGLDIDVNPHYTV

>GaAHA9

MADKSISLEEIKNETVDLVSISFPRIQPERIPVEEVFQQLKCTRNGLTSE

EGQKRLQIFGPNKLEEKKAKENKVLKFLGFMWNPLSWVMEFAAIMAIALA

NGGGKPPDWQDFIGIVSLLFINSTISFIEENNAGNAAAALMAGLAPKTKV

LRDGKWNEQEAAILVPGDIISIKLGDIVPADARLLEGDSLKIDQSALTGE

SLPVNKHSGDEVFSGSTVKQGEIEAVVIATGVHTFFGKAAHLVDSTNNVG

HFQQVLTAIGNFCICSIGVGMLIEIVVMYPIQRRKYRDGIDNLLVLLIGG

IPIAMPTVLSVTMAIGSHRLSQQGAITKRMTAIEEMAGMDVLCSDKTGTL

TLNKLTVDKSMIEVFMDNIDKEMVLLLAARASRVENQDAIDACIVGMLGD

PKEARAGVTEVHFFPFNPVDKRTAMTYIEADGSWHRVSKGAPEQIIELCN

LRNDAKRRAHDIITKFADRGLRSLAVAKQKVPEKTKDGQGDPWQFVGLLP

LFDPPRHDSAETIRRALSLGVNVKMITGDQLAIGKETGRRLGMGTNMYPS

SVLLGENKGDALDTIGVDELIEKADGFAGVFPEHKYEIVKRLQQRKHICG

MTGDGVNDAPALKKADTGIAVDDATDAARSASDIVLTEPGLSVIVSAVLT

SRAIFQRMKNYTIYAVSITIRIVLGFMLLALIWKFDFSPFMVLIIAILND

GTIMTISKDRVKPSPMPDSWKLKEIFCTGIVLGTYLACMTLVFFWAANDS

NFFSDKFGVRSIRHNQDELTAAVYLQVSIVSQALIFVTRSRSWSFIERPG

FLLVIAFILAQLVATVIAVYANWGFARIKGIGWGWAGVIWIYSVVFYFPL

DVIKFLIRYAMSGKAWNNLLQNKTAFTTKKDYGKGEREAQWALAQRTLHG

LTPPEMTEKSNYIELSEIAEQARKRAEVARLRELHTLKGHVESVVKLKGL

DIDTIQQHYTV

>GaAHA10

MAETSVGLEDIKNETIDLERIPVHEVFQKLKCTRDGLSSEEGQKRLHIFG

PNKLEEKQESKVLKFLGFMWNPLSWVMEIAAIMAIALANGGGKPPDWQDF

IGIVALLLINSTISFIEENNAGNAAAALMAGLAPKTKVLRDGKWSEQEAA

ILVPGDIISIKLGDIVPADARLLEGDPLKIDQSALTGESLPVNKNAGDEV

FSGSTVKQGELEAIVIATGVHTFFGKAAHLVDSTNNVGHFQQVLTAIGNF

CICSIGVGMLIEIVVMYPIQQRRYRDGIDNLLVLLIGGIPIAMPTVLSVT

MAIGSHRLSQQGAITKRMTAIEEMAGMDVLCSDKTGTLTLNKLTVDKSLV

EVFTNDVDKDMVILLAARASRVENQDAIDACIVGMLGDPKEARAGITEVH

FFPFNPVDKRTAMTYIKADGSWHRVSKGAPEQIIDLCNLRDDVKRRAHDI

IANFADRGLRSLAVARQTVKEKNKDAQGEAWEFVGILPLFDPPRHDSAET

IRRALNLGVNVKMITGDQLAIGKETGRRLGMGTNMYPSSALLGQNKGDTI

DTIGVDELIEKADGFAGVFPEHKYEIVKRLQQRNHICGMTGDGVNDAPAL

KKADIGIAVDDATDAARGASDIVLTEPGLSVIVSAVLTSRAIFQRMKNYT

IYAVSITVRIVLGFMLIALIWKFDFSPFMVLIIAILNDGTIMTISKDRVK

PSPMPDSWKLKEIFATGIVLGTYLACMTVVFFWAANDSNFFQDKFGVRSI

RHNQDELTAAIYLQVSIVSQALIFVTRSRSWSFIERPGLLLVVAFILAQL

IATLLAVYANWGFARIKGIGWGWAGVIWLYSLVFYVPLDVLKFLIRYSLS

GKAWDNLLQSKTAFTTKKDYGKGEREAQWAMAQRTLHGLTPPEMTQLYNE

ETNYRELSEIAEQARKRAEVARLRELHTLKGHVESVVKLKGLDIDTIQQH

YTV

>GaAHA13

MGDKNEVLEAVLKETVDLENIPIEEVFENLRCSREGLTTEAAEERLTIFG

HNKLEEKKESKFLKFLGFMWNPLSWVMEAAAIMAIALANGGGKPPDWQDF

VGIITLLVINSTISFIEENNAGNAAAALMARLAPKAKVLRDGRWNEQDAA

ILVPGDIISIKLGDIIPADARLLEGDPLKIDQSALTGESLPVTKGPGDGI

YSGSTCKQGEIEAVVIATGVHTFFGKAAHLVDTTNQVGHFQKVLTAIGNF

CICSIAVGMVIEIIVMYPIQDRDYRPGIDNLLVLLIGGIPIAMPTVLSVT

MAIGSHRLSQQGAITKRMTAIEEMAGMDVLCSDKTGTLTLNKLTVDKNLI

EVFAKGVDADTVVLMAARASRTENQDAIDSAIVGMLADPKEARAGIREVH

FLPFNPTDKRTALTYIDSDGKMHRVSKGAPEQILHLAHNKTDIERRVHAV

IDKFAERGLRSLAVAYQEVPDGRKESPGGPWQFIGLMPLFDPPRHDSAET

IRRALNLGVNVKMITGGFLLQLNVVIDFSGRGNGAMKKASEDIYRDQLAI

GKETGRRLGMGTNMYPSSALLGQDKDESIAALPVDELIEKADGFAGVFPE

HKYEIVKRLQARKHICGMTGDGVNDAPALKKADIGIAVADATDAARSASD

IVLTEPGLSVIISAVLTSRAIFQRMKNYTIYAVSITIRIVLGFMLLALIW

KFDFPPFMVLIIAILNDGTIMTISKDRVKPSPLPDSWKLAEIFTTGIVLG

SYLAVMTVIFFWAAYKTNFFPRVFGVATLEKTAHDDIKKLASAVYLQVSI

ISQALIFVTRSRSWSFVERPGLLLLAAFVIAQLIATLIAVYANWSFAAIE

GIGWGWAGVIWLYNIIFYIPLDFIKFFIRYALSGRAWDLVIEQRIAFTRQ

KDFGKEQRELQWAHAQRTLHGLQAPDTKMFTERTHFTELNQMAEEAKRRA

EIARLRELHTLKGHVESVVRLKNLDIDTIQQAYTV

>GaAHA4

MAGISLEEIKNETVDLEKIPIEEVFEQLKCTREGLSSDEGVNRIQIFGPN

KLEEKKESKILKFLGFMWNPLSWVMEAAAIMAIALANGEGKPPDWQDFVG

IVCLLVINSTISFIEENNAGNAAAALMAGLAPKTKVLRDGKWTEQEAAIL

VPGDIISIKLGDIIPADARLLEGDPLKVDQSALTGESLPVTKNPGDEVFS

GSTCKQGEIEAVVIATGVHTFFGKAAHLVDSTNQVGHFQKVLTAIGNFCI

CSIAIGMLVEIVVMYPIQHRKYRDGIDNLLVLLIGGIPIAMPTVLSVTMA

IGSHRLSQQGAITKRMTAIEEMAGMDVLCSDKTGTLTLNKLSVDKNLIEV

FVKDADKEHVVLLAARASRTENQDAIDAAIVGMLADPKEARAGIREVHFL

PFNPVDKRTALTYIDSNGNWHRASKGAPEQILALCNAKEDLKKRVHSIID

KFADRGLRSLAVARQQVPEKTKESAGTPWQFVGLLPLFDPPRHDSAETIR

QALHLGVNVKMITGDQLAIAKETGRRLGMGTNMYPSASLLGQDKDASIAA

LPVEELIEKADGFAGVFPEHKYEIVKKLQERKHICGMTGDGVNDAPALKK

ADIGIAVADATDAARSASDIVLTEPGLSVIISAVLTSRAIFQRMKNYTIY

AVSITIRIVFGFLFIALIWKFDFSPFMVLIIAILNDGTIMTISKDRVKPS

PLPDSWKLKEIFATGIVLGGYLALMTVIFFWAMHDTDFFSDKFGVRSLRE

REHEMMGALYLQVSIVSQALIFVTRSRSWSYAERPGLLLVTAFIIAQLVA

TLIAVYANWGFARIKGIGWGWAGVIWLYSIVFYVPLDIMKFAIRYILSGK

AWLNLLENKTAFTTKKDYGKEEREAQWALAQRTLHGLQPPETSNLFNDKN

SYRELSEIAEQAKRRAEVARLRELHTLKGHVESVVKLKGLDIDTIQQHYT

V

>GaAHA1

MGNDKGISLEEIKNESVDLERIPIEEVFEQLKCTRGGLTSEEGTNRLQVF

GPNKLEEKKESKFLKFLGFMWNPLSWVMEAAALMAIALANGDGRPPDWQD

FVGIIALLLINSTISFIEENNAGNAAAALMANLAPKTKVLRDGRWSEQDA

AILVPGDIITIKLGDIVPADARLLEGDPLKIDQSALTGESLPVTKNPSDE

VFSGSTCKQGEIEAVVIATGVHTFFGKAAHLVDSTNQVGHFQKVLTAIGN

FCICSIAVGIIIEIIVMYPIQHRKYRDGIDNLLVLLIGGIPIAMPTVLSV

TMAIGSHRLSQQGAITKRMTAIEEMAGMDVLCSDKTGTLTLNKLTVDRNL

IEVFAKGVEKEHVILYAARASRTENQDAIDAAIVGMLADPKEARAGIQEI

HFLPFNPVDKRTALTYIDAAGNWHRASKGAPEQIITLCNCKEDVKKKVHA

VIDKFAERGLRSLAVARQDVPEKSKDGPGAPWQLIGLLPLFDPPRHDSAE

TIRRALNLGVNVKMITGDQLAIAKETGRRLGMGTNMYPSSSLLGQDKDAS

IASLPVDELIEKADGFAGVFPEHKYEIVKRLQERKHICGMTGDGVNDAPA

LKKADIGIAVADATDAARSASDIVLTEPGLSVIISAVLTSRAIFQRMKNY

TIYAVSITIRIVFGFMFIALIWKFDFAPFMVLIIAILNDGTIMTISKDRV

KPSPQPDSWKLKEIFSTGIVLGGYLALMTVVFFWVMKDTDFFPNMFNVRS

LADSPEEMMAALYLQVSIVSQALIFVTRSRSWSYVERPGLLLLTAFVIAQ

LVATLIAVYANWGFARIKGMGWGWAGVIWLYSVVTYIPLDLIKFAIRYVL

SGKAWDNLLENKTAFTTKKDYGKEEREAQWAAAQRTLHGLQPPETSNLFN

EKSSYRELSEIAEQAKRRAEVARLRELNTLKGHVESVVKLKGLDIDTIQQ

HYTV

>GaAHA5

MGAISLEEIKNETVDLEKIPIEEVFEQLKCSREGLSSEEGANRLQIFGPN

KLEEKKESKILKFLGFMWNPLSWVMEAAAIMAIALANGDGKPPDWQDFVG

IVCLLVINSTISFIEENNAGNAAAALMAGLAPKTKVLRDGKWSEQEAAIL

VPGDIISIKLGDIIPADARLLEGDPLKIDQSALTGESLPVTKNPGDEVFS

GSTCKQGEIEAVVIATGVHTFFGKAAHLVDSTNNVGHFQKVLTAIGNFCI

CSIAIGMLVEIIVMYPIQHRKYRDGIDNLLVLLIGGIPIAMPTVLSVTMA

IGSHRLSQQGAITKRMTAIEEMAGMDVLCSDKTGTLTLNKLSVDRNLIEV

FVKDVDKEHVVLLAARASRTENQDAIDAAIVGMLADPKEARAGIREVHFL

PFNPVDKRTALTYIDSNGNWHRASKGAPEQILTLCNAKEDVKKKVHAIID

KFADRGLRSLGVARQLVPEKSKDGAGTPWQFVGLLPLFDPPRHDSAETIR

RALNLGVNVKMITGDQLAIAKETGRRLGMGTNMYPSASLLGQDKDASIAA

LPVEELIEKADGFAGVFPEHKYEIVRKLQERKHICGMTGDGVNDAPALKK

ADIGIAVADATDAARSASDIVLTEPGLSVIISAVLTSRAIFQRMKNYTIY

AVSITIRIVFGFMLIALIWKFDFSPFMVLIIAILNDGTIMTISKDRVKPS

PLPDSWKLKEIFATGIVLGGYLALMTVVFFWIMHDTDFFSEKFGVRSLRK

RDDQMMGALYLQVSIVSQALIFVTRSRSWSYFERPGLLLVTAFIIAQLVA

TVIAVYANWGFARIQGIGWGWAGVIWLYSVVFYIPLDLMKFAIRYILSGK

AWLNLLENKTAFTTKKDYGKEEREAQWALAQRTLHGLQPPEASNLFNDKS

SYRELSEIAEQAKRRAEVARLRELHTLKGHVESVVKLKGLDIDTIQQHYT

V

>GaAHA6

MGAISLEEIKNETVDLEKIPIEEVFEQLKCSREGLSSEEGANRLQIFGPN

KLEEKKESKILKFLGFMWNPLSWVMEAAAIMAIALANGDGKPPDWQDFVG

IVCLLVINSTISFIEENNAGNAAAALMAGLAPKTKVLRDGKWSEQEAAIL

VPGDIISIKLGDIIPADARLLEGDPLKIDQSALTGESLPVTKNPGDEVFS

GSTCKQGEIEAVVIATGVHTFFGKAAHLVDSTNNVGHFQKVLTAIGNFCI

CSIAIGMLVEIIVMYPIQHRKYRDGIDNLLVLLIGGIPIAMPTVLSVTMA

IGSHRLSQQGAITKRMTAIEEMAGMDVLCSDKTGTLTLNKLSVDRNLIEV

FVKDVDKEHVVLLAARASRTENQDAIDAAIVGMLADPKEARAGIREVHFL

PFNPVDKRTALTYIDSNGNWHRASKGAPEQILTLCNAKEDVKKKVHAIID

KFADRGLRSLGVARQLVPEKSKDGAGTPWQFVGLLPLFDPPRHDSAETIR

RALNLGVNVKMITGDQLAIAKETGRRLGMGTNMYPSASLLGQDKDASIAA

LPVEELIEKADGFAGVFPEHKYEIVRKLQERKHICGMTGDGVNDAPALKK

ADIGIAVADATDAARSASDIVLTEPGLSVIISAVLTSRAIFQRMKNYTIY

AVSITIRIVFGFMLIALIWKFDFSPFMVLIIAILNDGTIMTISKDRVKPS

PLPDSWKLKEIFATGIVLGGYLALMTVVFFWIMHDTDFFSEKFGVRSLRK

RDDQMMGALYLQVSIVSQALIFVTRSRSWSYFERPGLLLVTAFIIAQLVA

TVIAVYANWGFARIQGIGWGWAGVIWLYSVVFYIPLDLMKFAIRYILSGK

AWLNLLENKTAFTTKKDYGKEEREAQWALAQRTLHGLQPPEASNLFNDKS

SYRELSEIAEQAKRRAEVARLRELHTLKGHVESVVKLKGLDIDTIQQHYT

V

>GaAHA14

MVTVAPRFRVSLNHHERGARQTVAGSREFQPGGDRFGEFNERLPLEEVFE

QLRTSRAGLTSEDAEVRVHIFGQNKLEEKPENKFLKFLSFMWNPLSWVME

AAAVMAIVLANGGGEGPDWQDFVGIICLLIINSTISFIEENNAGNAAAAL

MARLAPKTKVLRDGQWQERDAAILVPGDIISIKLGDIIPADARLLEGDPL

KIDQATLTGESLPVTKRTGDEVFSGSTCKHGEIEAVVIATGVHSFFGKAA

HLVDSTEVVGHFQQVLTSIGNFCICSIAVGMVLEIIVMFPIQHRSYRDGI

NNLLVLLIGGIPIAMPTVLSVTLAIGSHRLSQQGAITKRMTAIEEMAGMD

VLCSDKTGTLTLNRLTVDRNLVEVFSKNMDKDLIVLLAARASRLENQDAI

DAAIINMLADPKEARANIKEVHFLPFNPVDKRTAITYIDSDGNWYRASKG

APEQLSGATNLSSYSSSSSTVYQILNLCLEKDLIAGRVHAIIDKFAERGL

RSLGVAFQEIPERTKESPGGPWTFCGLLPLFDPPRHDSAETIRRALNLGV

DVKMITGDQLAIAKETGRRLGMGTNMYPSSSLLGREKDENEALPVDELIE

KADGFAGVFPEHKYEIVKILQEKKHVVGMTGDGVNDAPALKKADIGIAVA

DATDAARSAADIVLTEPGLSVIISAVLTSRAIFQRMKNYTIYAVSITIRI

VLGFVLLALIWEYDFPPFMVLIIAILNDGTIMTISKDRVRPSPTPDSWKL

NEIFATGVVIGTYLALVTVLFYWIVIDTDFFETHFNVRSISDDTEQISSA

VYLQVSIISQALIFVTRSRSWSFVERPGVLLMCAFVVAQLVATLIAVYAH

ISFADISGIGWGWAGVIWLYSLVFYVPLDIIKFTVRYALSGEAWNLLFDR

KVSVLFLVSYLIGFKSFLEEAFHGIPVQQTAFTSKKDYGKDDRAAQWILS

QRSLQGLMAADLDFNGRRSRSSLIADQARRRAEIARLGELHTLRGHVESV

MRLKNLDLNAIKSAHTV

>GaAHA2

MGGEKGISLEEIKNESVDLERIPIEEVFEQLKCSREGLSTEEGNNRLQVF

GPNKLEEKKESKVLKFLGFMWNPLSWVMEAAAIMAIALANGDGRPPDWQD

FVGIIVLLVINSTISFIEENNAGNAAAALMANLAPKTKVLRDGRWSEQEA

AILVPGDIITIKLGDIVPADARLLEGDPLKIDQSALTGESLPVTKNPSDE

VFSGSTCKQGEIEAVVIATGVHTFFGKAAHLVDSTNQVGHFQKVLTAIGN

FCICSIAVGIIVELIVMYPIQHRKYRDGIDNMLVLLIGGIPIAMPTVLSV

TMAIGSHRLSQQGAITKRMTAIEEMAGMDVLCSDKTGTLTLNKLTVDRNL

IEVFTKGVEKEHVILYAARASRTENQDAIDAAIVGMLADPKEARAGVREI

HFLPFNPVDKRTALTYIDSDGNWHRASKGAPEQIIDLCKCKDDVRKKVHS

VIDKFAERGLRSLAVARQEIPEKTKESPGSPWQFIGLLPLFDPPRHDSAE

TIRRALNLGVNVKMITGDQLAIAKETGRRLGMGTNMYPSSSLLGQDKDAS

IAALPIDELIEKADGFAGVFPEHKYEIVKRLQERKHICGMTGDGVNDAPA

LKKADIGIAVADATDAARSASDIVLTEPGLSVIISAVLTSRAIFQRMKNY

TIYAVSITIRIVFGFMFIALIWKFDFAPFMVLIIAILNDGTIMTISKDRV

KPSPQPDSWKLKEIFSTGIVLGGYLALMTVIFFWAMKDTNFFSNTFNVRS

LRHNNIDGEKEMMAALYLQVSIVSQALIFVTRSRSWSYFERPGLLLVSAF

LVAQLVATLIAVYADWGFARIKGMGWGWAGVIWLYSVVTYIPLDLIKFAI

RYVLSGKAWDNLLENKTAFTTKKDYGKEEREAQWAAAQRTLHGLQPPETT

SIFNERSSYRELSEIAEQAKRRAEVARLRELNTLKGHVESVVKLKGLDID

NIQQHYTV

>GaAHA3

MDRLSGSHSGYTFCYYRWRATIKAPPPPFHMAGNKGISLDEIKNESVDLE

RIPIEEVFEQLKCTRAGLTTQEGNNRLQVFGPNKLEEKKESKFLKFLGFM

WNPLSWVMEAAAVMAIALANGGGRPPDWQDFVGIIVLLVINSTISFIEEN

NAGNAAAALMANLAPKTKVLRDGRWSEQEAAILVPGDIITIKLGDIVPAD

ARLLEGDPLKIDQSALTGESLPVTKNPSDEVFSGSTCKQGEIEAVVIATG

VHTFFGKAAHLVDSTNQVGHFQQVLTAIGNFCICSIAVGIVIELIVMYPI

QRRKYRDGIDNLLVLLIGGIPIAMPTVLSVTMAIGSHRLSQQGAITKRMT

AIEEMAGMDVLCSDKTGTLTLNKLTVDRNLIEVFAKGVEKEQVLLYAARA

SRTENQDAIDAAIVGMLADPKEARAGIREVHFLPFNPVDKRTALTYIDSD

GKWHRASKGAPEQIITLCNCKADVRNKVHAVIDKFAERGLRSLAVARQEV

PEKTKESPGAPWQLVGLLPLFDPPRHDSAETITRALNLGVNVKMITGDQL

AIAKETGRRLGMGTNMYPSSSLLGQDKDSSVSALPVDELIEKADGFAGVF

PEHKYEIVKRLQERKHICGMTGDGVNDAPALKKADIGIAVADATDAARSA

SDIVLTEPGLSVIISAVLTSRAIFQRMKNYTIYAVSITIRIVFGFLFIAL

IWKFDFAPFMVLIIAILNDGTIMTISKDRVKPSPQPDSWKLKEIFSTGIV

LGGYLALMTVLFFWAMKDTDFFSDKFHVRSLRDRPEQMMAALYLQVSIVS

QALIFVTRSRSWSFVERPGLLLVTAFIAAQLVATLIAVYANWRFAKIQGM

GWGWAAVIWLYSLVTYIPLDLIKFAIRYVLSGKAWDNLLENKTAFTTKKD

YGKEEREAQWAAAQRTLHGLQPPETSNVFNERSSYRELSEIAEQAKRRAE

VARLRELNTLKGHVESVVKLKGLDIDTIQQHYTV

>GaECA2

MEERAFPAWSWSVEHCLKEYDVRLDKGLSSYKVEKRREKYGWNELAKEKG

KPLWRLVLEQFDDMLVKILLVAAFISFLLAYMHGSESEESGFEAYVEPFV

IVLILVLNAIVGVWQETNAEKALEALKEMQCESGKVLRDGFLVPDLPARE

LVPGDIVELQVGDKVPADMRIAALKTSTLRLEQSALTGEAMPVLKGTSPI

FQKECELQAKENMVFAGTTVVNGCCVCIVVCTGMNTEIGKIQKQIHEASL

EESDTPLKKKLDEFGSRLTTAIGIVCLIVWLINYKNFLSYDMVEGWPANV

RFSFEKCTYYFKIAVALAVAAIPEGLPAVITTSLALGTRKMAQKNAIVRK

LPSVETLGCTTVICSDKTGTLTTNQMSVAEFFTLGGKTTTSRIFHVKGTT

YDPKDGGIVDWTCYNMDANLQVMAEICAVCNDAGIFCDGRLFRATGLPTE

AALKVLAEKMGVPDAKMRNKIRDSELVANYLIDRSTVKLGCCEWWIKRSK

RVATLEFDRVRKSSSIIVREAAGQNRLLAKGAVESLLERSTHVQLADGSL

APMDGPCRQLLLSRQTEMSSKGLRCLGLAYKEDLGEFSDYYSENHPAHKK

LLDPACYCSIESDLVFVGAVGLRDPPRDEVHKAIEDCKRAGIRVMVITGD

NKSTAEAICREIKLFSDGEDLRGKSFTGKDFMALSPSQQIETLSKPGGKV

FSRAEPRHKQEIVRMLKEMGEIVAMTGDGVNDAPALKLADIGIAMGITGT

EVAKEASDMVLADDNFSTIVSAVAEGRSIYNNMKAFIRQYMISSNVGEVI

SIFLTAALGLPECMIPVQLLWVNLVTDGPPATALGFNPPDIGIMRKPPRR

SDDALINSWVLFRYLIIGSYVGIATVGIFILWYTRASFMGINLVSDGHTL

VELSQLRNWGECSTWSNFTVAPYMVGGGQLISFSNPCDYFTAGKVKAMTM

SLSVLVAIEMFNSLNALSEDSSLLTLPPWRNPWLLAAMSVSFGLHCLILY

VPFFADIFAVAPLSLNEWFLVILVSVPVILIDEILKFVGRGQRYRVKEKT

A

>GaECA3

MEKKPFPAWSWSVEQCLKEYNVKLDKGLSSYQVEKQREKYGWNELSKEKG

KPLLRLVLEQFDDILVKILLVAAYISFILAYMHGSESEESGFEAYVEPFV

IVLILVLNAIVGVWQETNAEKALDALKEMQCESGKVVRDGYIVPDLPARE

LVPGDIVELQVGDKVPADMRIAALKTSTLRLEQSALTGEAMPVLKGTSPI

FPEECELQAKENMVFAGTTVVNGSCVCIVVCTGMNTEIGKIQKQIHEASL

EESDTPLKKKLDEFGSRLTTAIGLVCLIVWLINCKNFLSWDMVDGWPANL

RFSFEKCTYYFKIAVALAVAAIPEGLPAVITTSLALGTKKMAQKNAIVRK

LPSVETLGCTTVICSDKTGTLTTNQMSVAEFFTLGGKTTTSRIFHVEGTT

YDPKDGGIVDWTCYNMDANLQVMAEICAVCNDAGIFCDGRLFRATGLPTE

PALKVLVEKMGVPDAKMRNEIHDSQLAANYLIDRSTIKLGCWEWWTKRSK

RLATLELDTLRKSMSVIVREPTGHNRLLVKGAVESLVERSTHVQLADGSL

VPMDESCSQLLLSRNSEMSSKGLRCLGLAYKDDLGEFSDYYSENNPAHKK

LLDPASYSSIESDLVFVGVVGLRDPPRDEVDKAIEDCKGAGIRVIVITGD

NKSTAEAICHEIKLFSDGEDVRGKSFTGKEFMALSPSQQIETLSKPGGKV

FSRAEPRHKQEIVRMLKEMGEIVAMTGDGVNDAPALKLADIGIAMGITGT

EVAKEASDMVLADDNFSTIVSAVAEGRSIYNNMKAFIRYMISSNVGEVIS

IFLTAALGLPECMIPVQLLWVNLVTDGPPATALGFNPPDVGIMRKPPRKS

DDTLIDSWVLFRYLTIGSYVGVATVGIFILWYTQASFMGINLVSDGHTLI

ELSQLRNWGECSTWSNFSVAPYTVGDGHLITFSNPCDYFTIGKVKAMTLS

LSVLVAIEMFNSLNALSEDSSLLTLPPGRNPWLLVAMSVSFGLHCLVLYV

PFLANMFGVVPLSLNEWFLVILVSIPVILIDETLKFFGRSRRHRVKEKTA

>GaAHA8

MVDIDDTLQEIKNENVDLERIPVEEVFLQLKCTKEGLTTEEGLKRLQIFG

PNKLEEKSESKVLKFLGFMWNPLSWVMEIAAIMAIALANGGGKPPDWQDF

VGITVLLIINSTISFIEENNAGNAAAALMAGLAPKTKVLRDGKWCEQEAA

ILVPGDIISIKLGDIIPADARLLEGDALKVDQSALTGESLPVNKNPGDGV

YSGSTVKQGELEAVVIATGVHTFFGKAAHLVDSTNNVGHFQKVLTAIGNF

CICSIAVGMLVEIVVMYPIQRRKYRDGIDNLLVLLIGGIPIAMPTVLSVT

MAIGSHRLSQQGAITKRMTAIEEMAGMDVLCSDKTGTLTLNKLTVDKSMV

EVFTKDVDKEMLLLLAARASRVENQDAIDACIVGMLGDPKEAREGITEVH

FFPFNPVDKRTAMTYIESDGSWHRVSKGAPEQIISLCDLRDDVKKKAHDI

IDKFADRGLRSLGVARQTVPEKTKDSPGSPWEFVGLLPLFDPPRHDSAET

IRRALHLGVNVKMITGDQLAIGKETGRRLGMGTNMYPSSALLGHNKDEKV

ETIDVDELIEKADGFAGVFPEHKYEIVKRLQERNHICGMTGDGVNDAPAL

KKADIGIAVDDATDAARSASDIVLTEPGLSVIVSAVLTSRAIFQRMKNYT

IYAVSITIRIVLGFMLLALIWKFDFSPFMVLIIAILNDGTIMTISKDRVK

PSPMPDSWKLKEIFATGIVLGTYLACMTVVFFWAANDSDFFSDKFGVKSI

RYSQDELTAAVYLQVSIVSQALIFVTRSRSWSFIERPGLLLVVAFILAQL

VATVLAVYANWGFARIKGIGWPWAGVIWLYSIVFYIPLDVLKFLIRYALS

GKAWDNLLQNKTAFTTKKDYGREEREAQWATAQRTLHGLQAPGAEEILNE

KSSYRELSEIAEQAKKRAEVARLRELHTLKGHVDSVVKLKGLDIETINQN

YTV

>GaAHA12

MENKDETLDAVLKEAVDLENVPLEEVFQTLRCNRDGLTTEAAEQRLSIFG

YNKLEEKQESKILKFLGFMWNPLSWVMEAAAIMAIALANGGGKPPDWQDF

VGIITLLIINSTISFIEENNAGNAAAALMARLAPKAKVFRDGKWSEEEAS

ILVPGDIISIKLGDIIPADARLLDGDPLKIDQSSLTGESLPVTKGPGDSI

YSGSTCKQGEIEAVVIATGVHTFFGKAAHLVDSTNQQGHFQKVLTAIGNF

CICSIAVGMITEIIVMYPIQDREYRPGIDNLLVLLIGGIPIAMPTVLSVT

MAIGSHRLSLQGAITKRMTAIEEMAGMDVLCSDKTGTLTLNKLSVDKNLI

EIFAKGVDPDTVVLMAARASRLENQDAIDAAIVGMLADPKEARAGIQEVH

FLPFNPTDKRTALTYIDNQGRMHRVSKGAPEQILNLAHNKSELERRVHAV

IDKFAERGLRSLAVAYQEVPDGRKESSGGPWQFIGLMPLFDPPRHDSADT

IRRALNLGVNVKMITGDQLAIAKETGRRLGMGTNMYPSSSLLGQNKEESI

AALPVDELIEKADGFAGVFPEHKYEIVKRLQARKHICGMTGDGVNDAPAL

KKADIGIAVADATDAARSASDIVLTEPGLSVIISAVLTSRAIFQRMKNYT

IYAVSITIRIVLGFMLLALIWKFDFPPFMVLIIAILNDGTIMTISKDRVK

PSPLPDSWKLAEIFATGIILGGYLAMMTVIFFWAAYKTDFFPRTFGVKSL

QKTDRKDIKMLASAVYLQVSIISQALIFVTRARSWSFLERPGLLLVLAFV

VAQLIATLIAVYANWGFAAIEGIGWGWAGVIWLYNLIFYIPLDFIKFFIR

YALSGKAWDLVIEQRIAFTRKKDFGKEERELKWAHAQRTLHGLQPPDTKM

FGDRTSYNELNQMAEEAKRRAEIARLRELTTLKGHVESVVRLKGLDIDTI

QQAYTV

>GaECA4

MGKGGENYGKREFVNSKPTDPDVFPAWAKDIRECEKHYDVSRKSGLSTAE

VENRRKIYGNNELEKHEGQSIWSLIMEQFNDTLVRILLLAAVVSFVLAWY

DGDEGGEMEITAFVEPLVIFLILIVNAIVGVWQENNAEKALEALKEIQSE

QATVIRDGIKIPSLPAKELVPGDIVELKVGDKVPADMRVLELVSSTLRVE

QGSLTGESEAVNKTNKSVSEDADIQGKRSMVFAGTTVVNGNCFCLVTQIG

METEIGKVHTQIHVASQSEEDTPLKKKLNEFGEVLTMIIGVICIFVWLIN

VKYFLSWEYIDGWPRNFKFSFEKCTYYFEIAVALAVAAIPEGLPAVITTC

LALGTRKMAQKNALVRKLPSVETLGCTTVICSDKTGTLTTNQMAVAKLVA

MGGHASSLRSFRVDGTTYNPSDGKIHDWPSGGMDANLETIAKISAICNDA

GVTHSDNKYVAHGMPTEAAIKVLVEKMGLPKGLYSGGAAGDDVLRCCQWW

NEYEHRIATLEFDRDRKSMGVIVKSKSGRSSLLVKGAVENLLERSSKMQL

LDGSVVPLDQNSRILVSNALQDMSSGALRCLGFAYKDELPEFETYDGSDD

HPAHALLLDPSNYSSIESNLTFVGLVGLRDPPRQEVHQAIEDCKAAGIRV

MVITGDNKNTAEAICHEIGVFGPMEDISSKSLTGKEFMALSDKKAHLRQS

GGLLFSRAEPRHKQEIVRLLKEDGEVVAMTGDGVNDAPALKLADIGIAMG

IAGTEVAKEASDMVLADDNFSTIVSAVGEGRSIYDNMKAFIRYMISSNIG

EVASIFLTAALGIPEGLIPVQLLWVNLVTDGPPATALGFNPPDKDIMKKP

PRRSDDSLITAWILFRYLVIGLYVGVATVGVFIIWYTHRSFLGIDLSGDG

HTLVTYPQLANWAQCSSWKNFTVSPFTAGNQVFDFENNPCDYFQGGKVKA

MTLSLSVLVAIEMFNSLNALSEDGSLLTMPPWVNPWLLLAMSVSFGLHFL

ILYVPFLAQVFGIVPLSFNEWLLVLAVAFPVILIDEVLKFVGRLGRRMRS

SSQRPLKPKTE

>GaECA5

MGRGGENYGKRENAGAASFKQETFPAWARDVKQCEEKYQVNRELGLSSAE

VEKRRQIYGLNELLKHKPTSIFQLLLEQFNDTLVRILLAAAIISFVLAWY

DGEGGGEKEITAFVEPLVIFLILIVNAFVGIWQESSAEKALEALKEIQSE

HADVIRDGKKVSSLPAKELVPGDIVELRVGDKVPADMRVLNMISSTVRVE

QGSLTGESEAVSKTVKVVPENSDIQGKKCMVFAGTTMVNGNCICLVTEIG

MNTEIGKVHSQIHEASQSDTDTPLKKKLNEFGEVLTMIIGMICILVWLIN

VKYFLSWEYVDGWPTNFKFSFEKCTYYFEIAVALAVAAIPEGLPAVITTC

LALGTRKMAQKNALVRKLPSVETLGCTTVICSDKTGTLTTNQMAVSKLVA

IGSRSGTLRSFDVEGTTYDPFDGKILGWPVDGMDSNLEMIAKISAVCNDA

GVEQSGRHYVAIGMPTEAALKVLVEKMELPEKYASSSVPGDPRRCCHVWN

KMVQRIATLEFDRDRKSMGVIINSSSGNKSLLVKGAVENLLERSSFIQLL

DGSTVELDKYSKDLILQVLREMSTDALRCLGFAYKEELPEFATYNGDEDH

PAHQLLLNPSNYSSIESNLIFVGLVGLRDPPRKEVRQAIEDCKAAGIRVM

VITGDNKNTAEAICREIGVFGYREDITSRSLTGKEFMDHPDQRNHLRQNG

GLLFSRAEPRHKQEIVRLLKQDGEVVAMTGDGVNDAPALKLADIGVAMGI

TGTEVAKEASDMVLADDNFSTIVAAVGEGRSIYDNMKAFIRYMISSNIGE

VASIFLTAALGIPEGMIPVQLLWVNLVTDGPPATALGFNPQDTDIMKKPP

RRSNDSLITAWILFRYLVIGSYVGLATVGVFIIWYTHNTFMGIDLSGDGH

SLVTYSQLSNWDKCPSWVNFTASPFTAGPQVFNFDTNPCDYFRSGKIKAS

TLSLSVLVSIEMFNSLNALSEDGSLLTMPPWVNPWLLLAMSISFGLHFLI

LYVPFLAQVFGIVPLSLNEWLLVLAVAFPVILIDEVLKCIGRCTTGPRYS

PATKSIKHKAE

>GaHMA4

MVANKNLQKSYFDVLGLCCSSEVPLIENILKSLEGVKEVSVIVPTRTVIV

LHDNLLLSQLQIVKALNQARLEANVRAHGEIKYQKKWPSPFAVVCGLLLL

LSFLKYVYHPLQWLAVGAVVIGIYPVLFKGFAAITHFRIDINILILIAVI

GSVAMKDYTEAGTIVFLFTTAEWLESRASHKASAVMSSLMRITPQKAVIA

ENGEEVDADEVKLNTLLAVKAGEVIPIDGIVVDGRCEVDEKSLTGESLPV

TKEKDSTVWAGTINLNGYISVKTTAVAEDCVVAKMAKLVEEAQNSKSNTQ

RFIDKCAQFYTPVIIVVSAAIAVIPAALRVQNLHHWFHLALVVLVSACPC

ALILSTPVASFCALTKAATSGLLVKGGDYLETLSKIRITAFDKTGTLTRG

EFIVTDFQSLSQDISLDTLLYWVSSIESKSSHPMAAALVDYGRSHSIEPN

PETVEDYQNFPGEGIYGRVDGRDIYIGSLKISVRAHGTVPTLEGNMMKGK

TIGFVYSGATPAGIFSLSDACRTGVPEAVEELKSMRIKVAMLTGDNQSAA

IHVQEQLGNRLDVVHAELLPEDKARIIKEFKKEGATAMIGDGVNDAPALA

TADIGISMGISGSALATETGHVVLMSNDIRKIPKAIKLARKAHRKVIENV

ILSISTKTAILALAFAGHPLVWAAVLADVGTCLLVICNSMLLLRGKHKDG

RKCCKSSAAAHTNKHGCKASHCDSSHKHQDASLDKKVQKACEPPTCSSER

CASRCHSGLFKTDSPSNSRGSDKCEDLGRTHDGSVIREAKYCDQGSCHLV

NHKIEAQNLPRKCCSGRGSLDLGKEANALHGAKQCHQGHLHQYSSSTPEE

EQRETKNDHCHSTHCRENHVEIHGNNLTAFGNLVEHRCLESLNQRAHLDS

HEPTHTAIDITMNPDEVHGCANVEKRELGGCCKSYMKECCGKHKHGRFRP

GLTDIITE

>GaECA6

MGRGGEDYGKREKVSAASSKVENFPAWAKDVKQCEENFQTNRELGLSSAE

VEKRREIYGWNELEKHEGTSIFQLILEQFNDTLVRILLLAAIISFVLAWL

DGDEGGEKEITAFVEPLVIFLILIVNAIVGIWQESNAEKALEALKEIQSE

QANVVRDGKKVSNLPAKELVPGDIVELRVGDKVPADMRVLTLISSTVRVE

QGSLTGESEAVSKTAKVVPENTDIQGKKCMVFAGTTVVNGNCICMVTQIG

MNTEIGKVHSQIHEASQSDDDTPLKKKLNEFGEALTMIIGVVCALVWLIN

VKYFLSWEYVDGWASNFKFSFEKCTYYFEIAVALAVAAIPEGLPAVITTC

LALGTRKMAQKNALVRKLPSVETLGCTTVICSDKTGTLTTNQMAVSKLIA

IGSRPAILRAFDVEGTTYNPFDGKIRGWAAGEMDANLQMIAKICAVCNDA

GVEQSGSHYVATGMPTEAALKVLVEKMGLPEENGSSSDHWDHQRCCQAWN

KMEQRIATLEFDRDRKSMGVIVNSSTGQKALLVKGAVENLLERSSFMQLC

DGSIIELDQYSKDLILQSLHEMSTDALRCLGFAYKEEPFEFTTYNGDEDH

PAHQLLLNPSNYSSIESKLIFSGLVGLRHLDKPMKDPPRKEVRQAIEDCK

AAGIRVMVITGDNKNTAEAICREIGVFESSEDISSRSLTGKDFMDHPNQK

NHLRQSGGLLFSRAEPRHKQEIVRLLKEDGEVVAMTGDGVNDAPALKLAD

IGVAMGIAGTEVAKEASDMVLADDNFSTIVAAVGEGRSIYNNMKAFIRYM

ISSNIGEVASIFLTSALGIPEGMIPVQLLWVNLVTDGPPATALGFNPPDK

DIMKKPPRRSDDSLITAWILFRYLVIGLYVGIATVGVFIIWYTHHSFLGI

DLSGDGHSLVTYSRLANWAKCDSWEGFSVSPFTAGSQVFKFDSDPCDYFQ

SGKIKASTLSLSVLVAIEMFNSLNALSEDGSLLTMPPWVNPWLLLAMSVS

FGLHFLILYVPFLARVFGIVPLSTNEWLLVIAVAFPVILIDEVLKFIGRR

TTRLHYPAVPKSSKQKAE

>GaAHA7

MASNGDISLEQIKNETVDLERIPVEEVFQQLKCTREGLTSEEGEKRLQIF

GPNKLEEKEESKFLKFLGFMWNPLSWVMEAAAIMAIVLANGGGKPPDWPD

FIGIVSLLFINSTISFIEKNNAGNAAAALMAGLAPKTKVLRDGKWGEQEA

TILVPGDVINVKLGDIIPADARLLEGDALKFDQSALTGESLPVTKNPGDE

VFSGSTCKQGEIEAVVIATGVHTFFRKAAHLVDNTNNVGHFQKVLAAIGN

FCICSIAVGMLIEILVMYPIQHRRYRDGIDNLLVLLIGGIPIAMPTILSV

TMAIGSHSLSQQGAITKRMTAIEEMAGMDVLCSDKTGTLTLNKLTVDKSL

IEVFINDMDADTLVLIAARASRVENQDAIDASIVGMLGDFKEARAGITEV

HFLPFNPVDKRTAITYIDRNGEWHRCSKGAPEQVIELCELTGGLRQKAHH

VIDSFADRGLRSLGVARQTIPEKTKECAGGPWEFVGLLPLFDPPRHDSAE

TIRQTLDLGVNVKMITGDQLAIGKETGRRLGMGTNMYPSSSLLGQCKDEA

IATIPVDELIETADGFIGVFPEHKYEIVKRLQELKHICGMTGDGVNDAPA

LKKADIGIAVADATDAARSASDIVLTEPGLSVIVSAVLTSRAIFQRMKNY

TIYAVSITIRIVMGFMLIALIWEFDFSPFMVLIIAILNDGTIMTISKDRV

KPSPTPDSWKLKEIFITGVVLGAYMAIVSVIFFWLVHDTLFFTEKFGVKP

INENANELTSALYLQVSIISQALIFVTRSRSWSFVELPGLLLLGAFLAAQ

LVATCIAVYANWGFARIEGIGWEWTGVIWPHSQQRRIMNYGKEAREAQWA

ANQRTLHGLSPPETSWTNEKDHHELSEIAEQAKKRAEIARLRKLHTLKGH

VESVVKLKGIDIDTIQQHYSV

>GaHMA2

MESALSVTVPTLALFSIPRALNRHFYYNNCSLIARCIRSRLFPQGRGVTL

LASRSYSSPLRSLCAAPVPQRLHRRLECVASSAAYFGAAGGGGVYGGGDG

SGSGSGGGGGDGGEGTGDGDLKAKLGAGAVDEPSALSPDIIILDVGGMTC

GGCAASVKRILENQPQVSSASVNLTTETAIVWPVSEAKVVPNWQKELGEA

LARQLTSCGFNSNLRDSGRDNFFKVFERKMDEKRSRLRESGRELAVSWAL

CAVCLIGHVAHFLGAKASWMHAFHSTGFHLSLSLFTLLGPGRQLIFEGVK

NLFKGAPNMNTLVGLGALSSFAVSSLAVLIPKLGWRAFFEEPVMLIAFVL

LGRNLEQRAKIKATSDMTGLLSSLPSQARLMVDDSIVEVPCSSLSVGDQI

VVLPGDRVPADGIVRAGRSTIDESSFTGEPMPVTKEPGSQVAAGSINLNG

TLTIEVRRPGGETAMGDIVRLVEEAQSREAPVQRLADKVAGHFTYGVMAL

SAATFMFWNLFGARIIPASIYQGSTVSLALQLSCSVLVVACPCALGLATP

TAMLVGTSLGATRGLLLRGGNILEKFSMVNVIIFDKTGTLTIGRPVVTKV

VTPSGMDHSDSRQHFDGSWSEDDVLKLAAAVESNTIHPVGKAIVEAAQAV

KSPNIKVVDGTFVEEPGSGAVAVINDKTVSVGTLEWVQRHGVGDSLLLET

DEELRNKSVVYVGVNNKLAGLIYFEDQIREDARHVVDSLYRQGISVYMLS

GDKRSTAEYVASIVGIPKDKVLSQVKPDEKRKFVSELQENQNVVAMVGDG

INDAAALASAHIGVAMGGGVGAASEVSSIVLMGNRLSQLLDALALSQLTM

KTVKQNLWWAFAYNIVGIPIAAGTLLPLTGTMLTPSIAGALMGLSSIGVV

TNSLLLRFRFSLQQQQAYRSSLQPPPPPPPPPYAAVDINNDLAMDHSKAK

LKKPDSIT

>GaHMA7

MATKLLALACIRKESYGDLSPRPHYPSMPKYPKGVTAQETSLQGSEAKAM

FSVMGMTCSACAGSVEKAVKRLPGIKEAVVDVLNNKAQVMFYPSFVNEES

IREAIEDAGFQAALIQDETDDKSVQVCRIRINGMTCTSCSPTLENALQAV

PGVQKVQVALATEEAQIHHDPKIITYNQLMEKIEETGFGAVLVSTGEDMS

KINLRIDGVRTVNSMRMLENSLQALPGVQAVQTSPELKKIAVSYKPDMTG

PRNFIKVIDSTGSSRRFKATIYPEGEGAGRESHRKEEIKQYFRSFLWSLI

FTTPVFLTSMIFMYIPGIKHGLDTKVVNMLTIGEVIRWVLSTPVQFIIGR

RFYTGSYKALRHGSANMDVLIALGTNAAYFYSVYTVIRAASSPDFEGTDF

FETSAMLISFILLGKYLEVLAKGKTSEAIAKLMNLAPETAILLSLDEEGN

VISEEEIDSRLIQKNDIIKIIPGAKVASDGFVLWGQSHINESMITGEARP

VAKRKGDTVIGGTVNENGVLHIKATKVGSESALAQIVRLVESAQMAKAPV

QKFADRISKYFVPLVIMLSFSTWLAWFLAGKLHGYPESWIPSSMDSFELA

LQFGISVMVIACPCALGLATPTAVMVGTGVGASLGVLIKGGQALEGAHKV

NCIVFDKTGTLTVGKPVVVNTRLLKNVVLHEFYELVAATEANSEHPLAKA

IIEYAKKFREDEENPAWPEARDFVSKTGHGVKAIVRNKEVIVGNKSLMLE

NNIVIPVDAQDMLTETESMAQTGILVSIDSEVTGVLAISDPVKPGAQEVI

SILKSMNVRSIMVTGDNWGTASSIASQIGIETVVAEAKPEQKAEKVKELQ

AEGYAVAMVGDGINDSPALVAADVGMAIGAGTDIAIEAADIVLMKSNLED

VITAIHLSRKTFSRIRLNYIWALGYNILGIPIAAGALFPSTGFRLPPWIA

GAAMAASSVSVVCCSLLLKNYERPKKLENLEIGGIQIE

>GaACA13

MSSSGGNQMYDCGTLLFKVTTSGFTTAQKRWRIAYASIYSVRVMLSLAKE

IISKRGIEQPSIISDLHPYVALDVEPSSSPHWGEKFSSSSFAPKIDRKRL

VETLVETVKEKDLVSLHQLGGVEGIAAALGTNPEKGIRDDDRDVVKRQEM

FGTNTYHKPPPKGLLYFVLDAFKDTTILILLVCAALSLGFGIKEHGAAEG

WYEGGSIFVAVFLVIVVSALSNFRQETQFDKLSKISNNIKVEVVRGGRRR

QVSIFDLVVGDVVFLKIGDQIPADGLFLDGYSLQVDESSMTGESDHMEVD

ATRNPFLFSGSKVADGYGQMLVASVGMDTTWGEMMSSITSDKNERTPLQE

RLDRLTSSIGKVGLAVAFLVLVVLLIRYFTGNTEDDNGNTEYIGSKTSVD

DILNAVVRIVSAAVTIVVVAIPEGLPLAVTLTLAYSMKRMMADQAMVRKL

SACETMGSATIICTDKTGTLTLNQMKVTQFWLGQESIKEDHSNIIDHAVL

ELFYQGVGLNTTGSVCKPVSGSLPEFCGSPTEKAILSWAVLGLDLDMEKL

KQKYSILHVETFNSEKKRSGVSVRRKTDETLHVHWKGAAEIIVAMCSDYY

ESNGGIRSMDEDQRSRIETIIQSMAASSLRCIAFAHKQVSQKEMECVDDS

EKTHQRIKEDGLTLLGIVGLKDPCRPGVKKAVEACKSAGVDIKMITGDNI

FTAKAIAAECGILGADYNEESGQAIEGIEFRNYTPEERMEKIGKIKVMAR

SSPFDKLLMVQCLKQKGDVVAATGDGTNDALALKEADIGLSMGIQGTEVA

KESSDIVILDDNFSSVATVLRWGRCVYNNIQKFIQFQLTVNVAALVINFI

AAVSAGEVPLTAVQLLWVNLIMDTLGALALATDRPTKELMKKPPVGRTEP

LITNVMWRNLLAQAVYQIAILLILQFRGESMFNVPKRVKDTLIFNTFVLC

QVFNEFNARKLEKQNVFQGILQNRLFLGIVGITIILQVVMVEFLKKFADT

ERLELWQWGVCILFAAFSWPIAWVVKLIPVSDKPFFSYLKRSKSSSQLNK

SSTTRNLQSAGMELEVQ

>GaACA14

MSSSGGNQMYDCGTLLFKVTTSGFTTAQKRWRIAYASIYSVRVMLSLAKE

IISKRGIEQPSIISDLHPYVALDVEPSSSPHWGEKFSSSSFAPKIDRKRL

VETVKEKDLVSLHQLGGVEGIAAALGTNPEKGIRDDDRDVVKRQEMFGTN

TYHKPPPKGLLYFVLDAFKDTTILILLVCAALSLGFGIKEHGAAEGWYEG

GSIFVAVFLVIVVSALSNFRQETQFDKLSKISNNIKVEVVRGGRRRQVSI

FDLVVGDVVFLKIGDQIPADGLFLDGYSLQVDESSMTGESDHMEVDATRN

PFLFSGSKVADGYGQMLVASVGMDTTWGEMMSSITSDKNERTPLQERLDR

LTSSIGKVGLAVAFLVLVVLLIRYFTGNTEDDNGNTEYIGSKTSVDDILN

AVVRIVSAAVTIVVVAIPEGLPLAVTLTLAYSMKRMMADQAMVRKLSACE

TMGSATIICTDKTGTLTLNQMKVTQFWLGQESIKEDHSNIIDHAVLELFY

QGVGLNTTGSVCKPVSGSLPEFCGSPTEKAILSWAVLGLDLDMEKLKQKY

SILHVETFNSEKKRSGVSVRRKTDETLHVHWKGAAEIIVAMCSDYYESNG

GIRSMDEDQRSRIETIIQSMAASSLRCIAFAHKQVSQKEMECVDDSEKTH

QRIKEDGLTLLGIVGLKDPCRPGVKKAVEACKSAGVDIKMITGDNIFTAK

AIAAECGILGADYNEESGQAIEGIEFRNYTPEERMEKIGKIKVMARSSPF

DKLLMVQCLKQKGDVVAATGDGTNDALALKEADIGLSMGIQGTEVAKESS

DIVILDDNFSSVATVLRWGRCVYNNIQKFIQFQLTVNVAALVINFIAAVS

AGEVPLTAVQLLWVNLIMDTLGALALATDRPTKELMKKPPVGRTEPLITN

VMWRNLLAQAVYQIAILLILQFRGESMFNVPKRVKDTLIFNTFVLCQVFN

EFNARKLEKQNVFQGILQNRLFLGIVGITIILQVVMVEFLKKFADTERLE

LWQWGVCILFAAFSWPIAWVVKLIPVSDKPFFSYLKRSKSSSQLNKSSTT

RNLQSAGMELEVQ

>GaACA12

MSSSDECKLYDCSTSLLTVKAPGGFTVAQRRWRIAYITIYSARVMLSLAD

KIISQRATQLPSMTSQQYVTEFDHYVALDIEHKINQKRLVKTVKEKDLVS

LNHLGGVDGVVDALCTNSEHGIRDDEQEVIKRQEMFGFNKYHKPPPKGLL

YFVLEAFKDTTILILLVCATLSLGFGIKEHGAEEGWYEGGSIFVAVFLVI

VVSALSNFRQETQFDKLSKISNNIKVEVVRSGRRQQISIFDLVAGDVVFL

KIGDQIPADGLFLDGHSLQVDESSMTGESDHVEVDACRNPFLSSGSKVVD

GYARMLVASVGMDTAWGEMMSSITSDKNERTPLQARLDKLTSSIGKVGLA

VAFLVLAVLLIRYFTGNTKDDNGQTEYRGSQTDVDDILNAVVRIVAAAVT

IVVVAIPEGLPLAVTLTLAYSMKRMMADQAMVRKLSACETMGSATIICTD

KTGTLTLNQMKVTQFWLGQESVEEDLAKEIAPSVLELFYQGVGLNTTGSV

CKPVSGSLPEFSGSPTEKAILSWAVLGLGMDMEKLKQQYIILHVETFNSE

KKRSGVSVQRKADEMVDIHWKGAAEMIVAMCSQYYESNGIIRSMSEDGRE

RIETIIQSMAASSLRCIAFAHKQVLKGETEDGDDQSGKTNRRLKEDGLTL

LGIVGLKDPCRPGVKKAVQACQSAGVGVKMITGDNIFTAKAIATECGILG

PDYQQGSGEVVEGTEFRNYAPDERMEKVEKIRVMARSSPFDKLLMVQCLK

QKGHVVAVTGDGTNDAPALKEADIGLSMGIQGTEVAKESSDIVILDDNFS

SVATVLRWGRCVYNNIQKFIQFQLTVNVAALVINFIAAVSAGEVPLTTVQ

LLWVNLIMDTLGALALATDRPTNELMEKPPVGRTEPLITNIMWRNLLAQA

LYQIAILLILQFRGESIVNVPETVKDTLIFNTFVLCQVFNEFNARKLEKQ

NVFEGILKNRLFLGIIGVTIVLQVVMVEFLKKFADTEQLKLWQWGVCILL

AAFSWPIAWFVKLIPVSNTPFFSYLKRSRTIFKRPINHQKP

>GaHMA5

MSPGSRDLQLTSQAAGVWRSTYPSSVRADDPDDMEEGTRLLDSYETGDYK

LESIEEGSMRRIQVTVTGMTCAACSNSVEAALKSINGVLRASVALLQNRA

DVVFDPNLVKDEDIKNAIEDAGFEAEILPEPSNVGTKPRGVLVGQFTIGG

MTCAACVNSVEGILRDLPGVSRAVVALATSLGEVEYDPTVISKDDIVNAI

EDAGFEASLVQSSEQDKIILGVAGVFNELDVQLIEGILSSLKGVRQFRFD

RSSGELEVLFDPEVVSSRSLVDGIEGGSKGKFKLHVMNPYARMTTKDEET

SIMFQLFTSSLFLSIPVFLIRVVCPHIPLLDAFLLWRCGPFLMGDWLKWA

LVSVVQFVIGKRFYVAAGRALRNGSTNMDVLVALGTSASYFYSVGALLYG

AITGFWSPTYFETSSMLITFVLLGKYLECLAKGKTSDAIKKLVELAPATA

LLVVKDNGGNIIGEREVDALLIQPGDILKVLPGAKLPADGVVVWGSSYVN

ESMVTGESVPVSKEVDSPVIGGTINLHGALHIKATKIGSEAVLSQIISLV

ETAQMSKAPIQKFADFVASIFVPTVVTLSLITLLGWYAGGAAGAYPQQWL

PENGNYFVFALMFSISVVVIACPCALGLATPTAVMVATGVGASNGVLIKG

GDALERAQKVQYVIFDKTGTLTQGKAKVTTVKVFSEMDRGEFLTLVASAE

ASSEHPLAKAIVEYARHFHFFDENSLTEDAQYSSKESPISAWLLDVAEFS

AVPGRGIQCFIDGKRVLVGNRKLLTESGVSISAHVEQFVVDLEESARTGI

LAAYDGNVIGVLGVADPLKREAAVVVQGLQKMGVGPVMVTGDNWRTAQAV

AREVGIRDVRAEVMPAGKAEVVRSFQKDGSIVAMVGDGINDSPALAAADV

GMAIGAGTDIAIEAADYVLMRNNLEDVITAIDLSRKTFSRIRWNYVFAMA

YNVVAIPIAAGVLYPSLGIKLPPWAAGACMALSSVSVVCSSLLLRRYKKP

RLTTILEITVE

>GaACA2

MEELLKDFEVPPKNSSEAALRRWRKLVTIVRNPRRRFRMIANLEKRSEAE

QQKLKIKEKIRVALIVQKAALQFIDAAGPPDYKITDEVRQAKFGIEPDEL

ASIVHGHDIKRLKSHGGVDGIAEKVTVSLDEGVCSENVSTRQRIYGFNRY

TEKPPRNFWMFVWDALQDLTLIILMICAVVSIGVGLATEGWPKGMYDGAG

ILLSIILVVLVTAISDYRQSLQFRDLDREKKKISVQVTRDGRRQQVSIYD

LVVGDVVHLGIGDQVPADGLFISGYSVQIDESSLSGETDPVDIYEQKPFL

LSGTKVRDGSAKMLVTAVGMRTEWGKLMETLNEGGEDETPLQVKLNGVAT

IIGKIGLTFAVLTFLVLTVRFLIEKALHNEFTKWSSTDALTLLDYFAIAV

TIIVVAVPEGLPLAVTLSLAFAMKQLMDERALVRHLSACETMGSASCICT

DKTGTLTTNHMVVNKIWICEKIRNIGGNENKSIDELEIHESVFSILLRSI

FLNSSAEVVKDENGKNSILGTPTETALLEFGLLLSADLDAYRRQFKILKV

EPFNSDRKKMSVLVALPEGRIQAFCKGAPEIVLRMCEKVVDSSGEVVLLS

EERVRDITEAINGFASDALRTLCVAVKDVGETFNENGIPDSGYTLIAVFG

IKDPVRPGVKEAVQTCLAAGITVRMVTGDNINTAKAIAKECGILTAEENA

IEGPEFSSKSPDEMKDIIPNIQVMARSKPSDKLNFVINLRNMFGEVVAVT

GDGTNDAPALRQSDIGLAMGIAGTEVAKENADVIVMDDNFATIVNVAKWG

RAVYINIQKFVQFQLTVNVVALIINFVSACISGSAPLTAVQLLWVNMIMD

TLGALALATEPPNDALMKRPPVPRGASFITKPMWRNIIGQSIYQLIVLGV

LNFDGKQLLKLTGSDATTVLNTVIFNSFVFCQVFNEINSREIEKINILRG

MFSSWIFLGVMASTVAFQVVIVEFLGTFASTVPLSWQLWLLCILIGSVSL

IVGVIVKCIPVERAAVKPKHHDGYDALPSGPELA

>GaHMA6

MNINVGDNKMELNGRDDLKRPLLEPSDSVCVTIPEPVDKLEKKRTVMFKI

GNIKCASCVTSIESVLGEINGVESVSVSPIHGYAAIEYVPKLVNPKIIKE

TIEDAGFPVKEFSEQQIAVCRLRIKGMACTSCSESLERALKFLDGVKKAV

VGLALEEAKVHFDPNVTDSDRIIEAIEDAGFGADLISSGNEANKVHLKLE

GVSSVEDMNTIKSYLESAIGVNHVEMDLEEKMATVNYDPDFTGPRSIIEA

VQEVAHGSYKASLYIPPRQRETEQHHEINNYRNQFLLSCLFSVPLFIFSM

VLPMLPPFGDWLEYKIYNMFTVGLLLRWVLCTPVQFIVGRRFYKGSYHAL

RLKSANMDVLVAMGTNAAYFYSVYVAIKSLSSDTFKGQDFFETSAMLISF

ILLGKYLEVLAKGKTSDALAKLTDLAPDSACLLILDDDGNVVSEVAISTQ

LIQRNDIIKIIPGEKVPVDGIVTDGQSYVNESMITGEAQPIAKKPGDKVI

GGTMNENGCLLVNATHVGSETALSQIVQLVEAAQLARAPIQKIADRISRF

FVPAIVLTAFITWLGWLIPGVIGIYPKHWIPKGMDKFELALQFGISVLVV

ACPCALGLATPTAVMVATGKGASLGVLIKGGNALEKAHKVKAIVFDKTGT

LTVGKPEVVNVMLFSSVSMEDFCDVAIAAEANSQHPIAKAFLEHARKLRQ

KIESNRQSNNQHVTEAKDFEVHPGTGVSGKVGDKMVLVGNKRLMQTYNVT

VGPEIEGYISEHEQQARTCVLVSIDGKIAGAFAVTDPVKPEANNVILYLR

SMGISSIMVTGDNWATATAIAKEVGIEKVIAETDPIGKADRIKDLQMRGL

TVAMVGDGINDSPALVAADVGMAIGAGTDVAIEAADIVLIKSNLEDVVTA

IDLSRKTISRIWLNYVWALGYNILGVPVAAGILYPFTGVRLPPWLAGACM

AASSLSVVCSSLLLQSYRKSWVFQDTKIGHSHCSKST

>GaACA8

MSLRLRKPSEPTMHRQVEPSKSSVRRWRVAVTAISVTRFLVGLTKKVVEK

NAELLRSLSFVTIDVEGSGDERVPILDVDPQGLAKMVKDKSFQSLNDQYG

GVKQVATLLQTDFKKGIPGDDNDLALRTKVFGANKYQKQPAKSFFSFVLE

AFKDTIIIILLVCAVLSLGFGIKQHGLKEGWYDGGSIIVAVVLVVVVSAV

SNYRQSKQFEELSHETNDIRVQVVRNGRYQPVSIFELVVGDIVSLKTGDQ

IPADGLFVEGHSLKVDESSMTGESDHVEVNEKKNPFLLSGTKVTDGHGYM

LVTAVGMNTAWGEMMSSIRRDLNEETPLQARLSKLTSYIGNIGLSVAVLV

LLVLLIRYFTGHTKAENGRSAFNGSKTKFDDVMNSVVGIIAAAVTIVVVA

IPEGLPLAVTLTLAYSMKRMMRDHAMVRKLSACETMGSATIICTDKTGTL

TLNEMKVTEFWLGKEPIDNSMSSEIAPNVLQLLSEGVGLNTTGTVYKPKP

TSVPEIYGSPTEKAILSWALNDMGLNIDESKQSCEIIHVEAFNSEKKRSG

VLIRRSNNKRVLATHWKGAAEMLLAMCSCYYDKKGVSKFMNEDEREHIGM

VIESMAAKSLRCIAFATLDVTVTDGNEENHTKLEERGLTWLGLVGLKDPC

RPGVKQAVESCQNAGVSIKMITGDNMHTARAIAFECGILNSESSLHNEAV

VEGVQFRNYSEEERMQKIETIRVLARSSPFDKLLMVQCLKQKGHVVAVTG

DGTNDAPALKEADIGLSMGIQGTEVAKESSDIVILDDNFTSVATVLRWGR

CVYNNIQKFIQFQLTVNVAALVINFIAAVSSGDVPLTAVQLLWVNLIMDT

LGALALATEQPTNDLMDKRPVGRTEPLITKVMWRNLTAQALYQVAILLIL

QFKGKSIFGVSEEVKDTLIFNTFVLCQIFNEFNARNMDKKNIFKGIHKNR

LFLAIIGITLVLQAIMVEFLQRFANTERLSWEQWGACIGIAALTWPIGWI

VKCIPVDKKVQTRSSAAS

>GaACA3

MEDYLRKNFAVEPKRPSEEALRRWRSAVALVKNRSRRFRMVADLAKRAEA

DRRRKIIQEKIRVALYVQKAALNFIDAGKQAERKLPEDVREAGFHIGADE

LASIVRSHDMSSFEEHGGVEGLAKKVSVSLTNGVVPTDISFRKNIYGNNK

FDEKPARSFWMFVWEALHDLTLIILIVCAVVSIGVGVATEGWPGGLYDGL

GIVLCIFLVVFVTAISDYKQSLQFKDLDKEKKNILVQVTREGCRQKISIY

DLVVGDIVHLSIGDQVPADGVLISGYSLSIDESSLSGESEPVKVTQERPF

LLSGTKVQDGSGKMLVTTVGMRTEWGRLMVTLSEGGVDETPLQVKLNGVA

TVIGKIGLVFAVLTFLVLAIRFMVTKAQLGEIEKWGMSDVLSLLNFFAVA

VTIIVVAVPEGLPLAVTLSLAFAMKKLMSDKALVRHLSACETMGSATCIC

TDKTGTLTTNHMVVDKIWTCGRTISIAGDNKRKDVLRSSIAGEVLDLLLQ

SIFQNTGAEVVKGKDGKNNILGSPTETAILEFGLLLGGEFKKQRKESTIL

KVEPFNSEKKRMSVLVSLSNGGENRAFCKGASEIILESCNKVINVDGKAE

HLSKEQKKFITDVINGFACEALRTLCLAFKDVKDTSDVDSDSIPQENYTL

IAVIGIKDPVRPGVRQAVETCLSAGIKVRIVTGDNINTAKAIARECGILT

ENGLAIEGPEFRDMSPRQMEETIPKLQVLARSLPLDKHKLVTYLRMEFKE

VVAVTGDGTNDAPALHEADIGLAMGIAGTEVAKENADVIIMDDNFATIQN

VARWGRAVYINIQKFVQFQLTVNIVALMLNFVSACISGSAPLTAVQLLWV

NMIMDTLGALALATEPPHEGLMKRPPIGRDVAFISRVMWRNIIGQTIYQL

IVLAILKFDGKRLLKISGSNATAILNTLIFNSFVFCQVFNEINSRDMEKI

NVFRGFFDSWLFIMVMVCTVGFQSIIVELLGTVADTVPLSWELWLTSILL

GAGSLIVAVILKCIPVENWREASTTKHHDGYEPLPTGPDMA

>GaHMA3

MAMAGDLLRLTVLNRPKLSLGNGAKLKADRFGLLKRCPRGRFHCQPRSTP

GFVLFSSLETRLESEESSIQPVGQKLKDPSVLLDVNGMMCGGCVSRVKSV

ISSDERVESVVVNLLTETAAIKLKREVMERETAESVAESIAQRVSECGFM

AKMRVSGTGIAENMRKWQEMLKKKEELLVKSRNRVAFAWTLVALCCGAHA

SHILHSLGIHFGHGSFLEILHNSYVKGGLALTALLGPGRDLLVDGLLAFK

KGSPNMNSLVGFGSIVAFIISAVSLLNPGLKWDASFFDEPVMLLGFVLLG

RSLEEKARIRASSDMNELLSLISTRSRLVITSSDTDSSADSVLSSDAICI

EVPSDDIRVGDSVLVLPGETIPVDGKVLTGRSVVDESMLTGESLPVFKEK

GLMVSAGTINWDGPLRIEATSTGSNSTIAKIVRMVEDAQGQEAPVQRLAD

AIAGPFVYSIMTLSAATFAFWYYAGSHIFPDVLLNDIAGPDGDSLLLSLK

LAVDVLVVSCPCALGLATPTAILVGTSLGARQGLLIRGGDVLERLANTGT

LTEGKPTVSSVSSFTYDESEILQIAAAVERTATHPIAKAIVKKAELLNLL

LPETRGQLVEPGFGTLAEVNGCLVAVGKLEWVNERFQIKASPSDLMALEH

AVMRQSSSPSNYSKTAIYVGREGEGVIGAIGMSDSLRFDAKSTVSRLQRK

GIKTILISGDREEAVATIAKTVGIVHEFVNASLTPQQKSRVISTLQTAGH

HIAMVGDGINDAPSLAIADVGIALQTEAQETAASDAASIILLGNRLSQVV

DALDLAQATMAKVYQNLSWAVAYNIVAIPIAAGVLLPQYELAMTPSFSGG

LMALSSIFVVTNSLLLRLHGSEKSWKNSIAKISQMPAGP

>GaACA9

MSLRHRNTGYLEPAMSDGEDTPIVKCQHRRWRSVFAAIYSARIFVSLYKK

IINKKQILRSLSYIALDVHDSDSSDDRLPSLGVDQKTLTEVVREKSLETL

SKLGGVKQIAASLETDKKDGISANEADLAHRVNVFGANRYQKPPKKSFFS

FVYEAFKDTTIIILLVCAVLSLGFGIKQHGITDGGYDGGSIVIAVFLVVA

VSAVSNFKQNRQFEKLSKESSDIKVEVVRDGRRQFISVFEVVVGDVVCLK

IGDQIPADGLFLDGHSLKVDESSMTGESDHVEINGSNNPFVLSGTKVTNG

FGSMLVTSVGMNTAWGEMMSSINRELDEETPLQARLNKLTSAIGKIGLAV

AVLVLAVLLIRYFTGNTKDDQGNKEYIRGKTKFDSMMNSVVEIISAAITI

VVVAIPEGLPLAVTLTLAYSMKQMMADHAMVRKLSACETMGSATTICTDK

TGTLTLNEMKVMEFWLGKELMGSISSEIAPNVHKLLQQAVALNTTGTVYK

PNSRSLPEISGSPTEKAILSWAVSDLGMNLDDPKQNYELIQVEAFNSEKK

RSGVLIRRKSESGGATQVHWKGAAEMILAMCSQYYDRSGVVKAIDEEERV

EMGKVIEDMAAKSLRCIAFAHTQNPKDNERVLQESGLILLGLVGLKDPCR

PGVRKAVEACINAGVNIKIITGDNIFTAKAIATECGILQPNEDLREAVIE

GVQFRNYSPEERMAKINKICVMARSSPFDKLLMVQCLKQNGHVVAVTGDG

TNDAPALKEADIGLSMGIQGTEVAKESSDIIILDDNFTSVVTVLRWGRCV

FNNIQKFIQFQLTVNIAALVINFIAAVSSGEIPLTAVQLLWVNLIMDTFG

ALALATERPTNDLMTKPPVGRSKPLISNIMWRNLIAQALYQVAVLLTLQF

RGKFIFDVDKKVNNTLIFNTFVLCQVFNEFNARKLEKKNIFQGLHKNKLF

LGIIAITIILQVVMVEFLKRFANTQRLNWGQWGTCIGIAALSWPLGWLVK

WIPA

>GaECA1

MEDAYARSVSEVLDFFGVDSSKGLTDFQVSQHARLYGKNGTPFWKLVFKQ

FDDLLVKILIAAALVSFLLALINGETGLIAFLEPSVILMILAANAAVGVI

TETNAEKALEELRAYQADIATVLRNVGCKIPADMRMIEMLSGQLRVDQAI

LTGESSSVEKDLESTIATNAVYQDKTNILFSGTVVVAGRARAVVIGVGAN

TAMGSIRDSMLRTDDEATPLKKKLDEFGTFLAKVIAGICVLVWIVNIGHF

RDPAHGGFLRGAIHYFKIAVALAVAAIPEGLPAVVTTCLALGTKRMARLN

AIVRSLPSVETLGCTTVICSDKTGTLTTNMMSVSKICVVHSIKNGPEVAE

FGVSGTTYAPEGFIFDNTGVQLEFPAQLPCLLHIAMCSALCNESLLQYNP

DKGNYEKIGESTEVALRVLAEKVGLPGFDSMPSALNMLSKHKRASYCNHY

WENQFKKVSVLEFSRDRKMMSVLCNHKQMEIMFSKGAPESIISRCTNILC

NNDGSTIPIDATLRAELDSRFNSFDLRRVSSLFSFAGKETLRCLALALKI

MPMGQQTLSFDDEKDLTFIGLVGMLDPPREEVRNAMISCMTAGIRVIVVT

GDNKSTAESVCRKIGAFDHLVDYVGHSYTAAEFEELPGTQQTMALQRMAL

LTRVEPSHKRMLVEALQNQNEVVAMTGDGVNDAPALKKADIGIAMGSGTA

VAKSASDMVLADDNFATIVAAVAEGRAIYNNTKQFIRYMISSNIGEVVCI

FVAAVLGIPDTLAPVQLLWVNLVTDGLPATAIGFNKPDSDVMKAKPRKVS

EAVVSGWLFFRYLVIGAYVGLATVAGFIWWFIYSETGPKLPYTELMNFDT

CPTRETTYPCSIFEDRHPSTVAMTVLVVVEMFNALNNLSENQSLLVIPPW

SNLWLVASIILTMLLHILILYVPPLSTLFSVTSLSWNEWAVILYLSFPVI

IIDEVLKFFSRNSHGNAYCIRFNFRFRRYDALPKKELRDK

>GaACA10

MTTILQSNLLCFEYTIQVPSATFTKSRKKWHSLFATIYCSRKFSSLITKT

ATANDEARVVHRSPSHVSLAVMQENSPFRIDQPTLIELVKEKKIEKLRKH

GGVDGVASGLGTDTQVGVSGSAEDIERRHEAFGSNTYKKPPTKGFFHFVV

EAFKDLTIMILLGCAALSLGFGIKEHGLKDGWYDGGSIFVAVFLVIGVSA

VSNYRQNRQFDKLSKVSNNIQVDVVRGGRRQQISIFDIVVGDIVCLKIGD

QVPADGLFTDGHSLQIDESSMTGESDHVEVNGSQNPFLLSGTKVADGYAR

MLVTSVGMNTTWGQMMCQISRDTNDETPLQARLNKLTSSIGKVGLAVAFL

VLVVLLVRYFTGHTTDENGNREFNGSKTKSDDIINAVVGIVAAAVTIVVV

AIPEGLPLAVTLTLAYSMKRMMADQAMVRKLSACETMGSATTICTDKTGT

LTLNRMKVTKFWLGQESMEEGASSISPFVVDLIHQGVALNTTGSVYRASP

GTEYEFSGSPTEKAILSWAVVELKMDMEKTKKCCAVLQVEAFNSQKKRSG

VLIGRNDDDTVHVHWKGAAEMILALCSSYYDASGVEKDLDDDERMKFEQI

IQGMAASSLRCIAFAHKQVPEEEYQNLKEQKKLKEDNLTLLGLVGIKDPC

RPGVKKAVEDCQYAGVNIKMITGDNVFTARAIATECGILKPGQDLSSGAV

VEGEEFRNYTPQERMEKVEKIQVMARSSPFDKLLMVQCLKQKGHVVAVTG

DGTNDAPALKEADIGLSMGIQGTEVAKESSDIVILDDNFASVATVLRWGR

CVYTNIQKFIQFQLTVNVAALCINFVAAVSAGEVPLTAVQLLWVNLIMDT

LGALALATERPTKELMEKPPVGRTEPLITNIMWRNLLAQALYQVAILLTL

QFSGESILGVTEKVNDTLIFNIFVFCQVFNEFNARKLEKKNVFEGIHKNK

LFIGIIGVTILLQVVMVEFLKRFADTERLSWGQWGACIAVAAVSWPLGWV

VKCLPVPQKPIFSYLKWWK

>GaACA4

MGTFWNENFDLKPKHSSEEALEKWRKVVGFVKNPKRRFRFTANLSKRYEA

AAMRRSNHEKLRIAVLVSKAALQFISGVKPSESDYVVPEEVKAAGFELCA

EELGSIVENQDVKKLKIHGGVDGIAEKLSTSTTDGLSSDSGLLNKRQEVY

GINKFAEAEAKGFLVFVWEALQDMTLMILGVCALVSLIVGIAMEGWPKGA

HDGLGIVASILLVVFVTATSDYRQSLQFKDLDKEKKKITIQVTRNACRQK

MSIYDLLPGDIVHLNIGDQVPADGLFVSGFSVLIDGSSLTGESEPVIVNV

DNPFMLSGTKLQDGSCKMMVTSVGMRTQWGKLMATLSEGGDDETPLQVKL

NGVATIIGKVGLFFAVVTFAVMVQGLFTSKLQEGTIWSWSGDEALKLLEF

FAVAVTIVVVAVPEGLPLAVTLSLAFAMKKMMNDKALVRHLAACETMGSA

TNICSDKTGTLTTNHMTVVKSCICMDVREVGNNNKASLCSEIPESAVKLL

LQSIFTNTGGEIVINKDGKREILGTPTETALLEFGLSLGGDSQAERLASK

LVKVEPFNSTKKRMGVILELPEGGLRAHTKGASEIVLAGCDKVINSNGEV

IPLDAESINHLNATINQFANEALRTLCLAYMELENGFSPDNAIPVSGYTC

IGIVGIKDPVRPGVKESVAICRAAGITVRMVTGDNINTAKAIARECGILT

DDGIAIEGPDFREKSQEEMLALIPKIQVMARSSPMDKHTLVRQLRSIDEV

VAVTGDGTNDAPALHEADIGLAMGISGTEVAKESADVIILDDNFSTIVTV

AKWGRSVYINIQKFVQFQLTVNIVALIVNFSSACLTGTAPLTAVQLLWVN

MIMDTLGALALATEPPTDELMKRAPVGKKGNFISNVMWRNILGQSFYQFM

VIWYLQVKGKGMFSLDGPDSDLKLNTIIFNSFVFCQVFNEISSRNMEEIN

VFKGILNNYVFVAVLGCTAVFQVIIIEFLGTFASTTPLTCLQWFVSVFIG

FLGMPIAAALKTIPV

>GaHMA1

METHSISLTNFSPLTRPLRPSRLGRLNSFHFKPLFFSPLSTRYKSLFLPL

NSHTIRIRCVANHEHHHHHEHDHDHDHDHHHHHHHGSGLLNGPQKAVIGF

AKAIRWMDLANFLREHLHLCCCATALFIAAAACPYLVPKPAVKPLQNSFL

VLAFPLVGVSAALDAITDIAGGKVNIHVLMALAAFASVFMGNALEGGLLL

AMFNLAHIAEEFFTSRSMIDVKELKENYPDSALVLNLDDDNLPNVSDLSY

QSIPVHDVEVGSYILVTTGEAVPVDCEVFHGSATITIEHLTGEIKPLEAK

AGDRIPGGARNLDGRMIVKVLKTWKESTLSRIVQLTEEAQLNKPKLQRWL

DEFGEQYSKVVVVLSVAIAVLGPFLFKWPFISTAVCRGSIYRALGLMVAA

SPCALAVAPLAYATAVSSCARKGILLKGGQVLDALASCHTVAFDKTGTLT

TGGLMFKAIEPIYGHIIGNMKTNLTSCCVPSCEVEALAVAAAMEKGTTHP

IGRAVVDHSIGKDLPSVSVESFEYFPGKGLIATLNSAESGTRGGKMLKAS

LGSIEFITSLCKSEVESRKITAAVNASSFGTDFVHAALSVDEKVTLIHLE

DRPRPGVLDVISELKDKAKVRVMMLTGDHKLSAWRVANAVGINEVYCSLK

PEDKLNHVKRISGDMGGGLIMVGEGINDAPALAAATVGIVLAHRASATAI

AVADVLLLQDNISGVPFSIAKARQTTSLVKQNVALALTCIILASLPSVLG

FLPLWLTVLLHEGGTLLVCLNSIRALNDPSWSWGQDLRNLIGKLKSKLAL

LRHNATSSPIQTAPL

>GaACA11

MTTIFEPNLICSEHTIQVPPSLSTSDKAWHSIFASKKFSSLLTKSPTPKE

EAKVVRRSPSHVSVNVMQENPLYQIDQRTLIELVKEKKLDRLQEFGGVNG

VASNFGTDTQVGISGGADDVARLRNTFGSNTYNKPPTKGFFHFVIEALKE

PAVLILLGCAALSLGFKIKAHGHKDSWYEVGSNFAAVFLPIVVSAISNYM

QNRQFKELSRTNNKILVDVVRGGQRQQIRMFDVVVGDIVCLKMGDQVPAD

GLFLDGHSLQVDESSMTGETEHTEVNSSQNPFLLSWTKVAHGDARMLVTS

VGSNTTWGQISRETNEQTPLQARLNKLSSSIAKVGLAVAFPVLLVLLVRY

FTGHTKDEKGNREFHRSKAKTSDIINSIVGIATTAITTVAEGLPLVVTLT

LAYSMKKMVADQAMVRKLSACETMGSTTTICTNKTGTLTLNRMEVTKFWL

GQESMEEGASSISPFVVDLIHQGVALNTTGSFYRASPGTEYEFSGSPTEK

AILSWAVVELKMDVEKTKKSCAVLQVEAFISQRRRSGVLIERNDDDTVHV

HWKGAAEMILAMCSSYYDASGVVKDLDDGERTKFEEIIQGMAASSLRCIA

FAHKQVPEEEYQNLKEQKKLKEDSLALLGLVGIKDPCRPGVKKAVEDCQY

AGVNIKMITGDNVFTARAIAIECGILKPGQDLSSGAVVEGEEFRKYTLQE

RMEKVEKIQVMARSSPCDKLLMVQCLKQKGHVVAFTGDGTDDAPALKEAD

IGLSMGIQGTEAAKESSDIVILDDSFASVATVLRWGRCVYTNIQKFIQFL

LTVNVSALCINFIAAVSTGEVPLTTVQLLWVNLYMDTLGALALATELPTK

ELLEKPPVGRTEPLITNIMWRNLLAQALYQIAVLLTLQFNGESIFGVTKK

VNHTLIFNTFMLCQVFNNFNARKLEKKNVFDDIHKNKMFIGIIGVTIVLQ

VVMVEFLKRFTDTERLNWGQWGACIAIAAASCPLGWAVKCLPVPKKPIFS

YQKW

>GaACA5

MSSIFKGSPYRRPNDLEAGSSRSAHSDDEDHESCADPFDITSTKNAPIDR

LRRWRQAALVLNASRRFRYTLDLKKEEEKKQILRKIRAHAQAIRAAYLFK

QAGEQVNGTTTPHPTPGSDFAFGPEQLASVTRDHNFNALQEYGGVNGLAE

SLKTNLQKGIPGDDSDLLKRRNAFGSNTYPRKKGRSFWRFVWEACQDLTL

MILVVAAVASLALGIKTEGPKEGWYDGGSIAFAVFLVIIVTAISDYRQSL

QFQKLDEEKRNIHLEVVRGGRRVEISIYDIVVGDIIPLNIGDQVPADGIL

ISGHSLAIDESSMTGESDIVQKDAKQPFLMSGCKVADGSGTMLVTGVGIN

TEWGLLMANLSEDTGEETPLQVRLNGVATFIGFVGLSVAFAVLVVLLVRY

FTGHTEDSNGRQQFVAGKTSVGNAIDGAIKIVTVAVTIVVVAVPEGLPLA

VTLTLAYSMKKMMADKALVRRLSACETMGSATTICSDKTGTLTLNQATYA

GGRKNDPPERRSELPDTLVSLLIEGIAGGGDVEVSGSPTEKAILNWGIKL

GMDFDAVRSGSSIVHVFPFNSEKKRGGVAIRLLSLRSLFEKFLGRLVTTA

AQASNSNPIFISKPDSKVHIHWKGAAEIVLAACTRYLDTNGEAVAMDEEK

MAFFEKAIETMAAGSLRCVAIAYRSYDSEKVPTNEEELAKWALPEDDLVL

LAVVGIKDPCRPSVKKSVQLCQKAGVKVRMVTGDNLKTARAIALECGILS

SDAPESSLIEGKVFRSLSDSEREEVAEKISVMGRSSPNDKLLLVQALRRK

GHVVAVTGDGTNDAPALHEADIGLAMGIQGTEVAKESSDIIILDDNFASV

VKVVRWGRSVYANIQKFIQFQLTVNVAALVINVVAAVSSGDVPLNAVQLL

WVNLIMDTLGALALATEPPTDHLMHRPPVGRREPLITNIMWRNLLIQAIY

QVTVLLVLNFDGKKILHLEHESKEHANRVKNTLIFNAFVLCQIFNEFNAR

KPEEVNIFRGLSKNYLFIGIVAITIILQAIIVEFLGKFAKTEKLSWQLWL

VSIGIGFISWPLAILGKFIPVPETPVSRVFSRMFYRRRNQNVSGSQKHED

SNATSIRN

>GaACA6

MSGTSSGNGLLHLNDVEAGLSKDNADLDHHLDPDAGTSDPFDIAHTKNAA

PETLKRWRQAALVLNASRRFRYTLDLKKEEEKEQRKRMIRAHAQVIRAAL

LFKLAGENQIVSSAPVASPSAGDDYKIGLEHLASMTRDHKLSALEQYGGV

KGLSGLLRTNLEKGIDEDEADLLNRRNVFGSNTYPRKKGRSFWMFLWEAW

QDLTLIILIIAAAVSLGLGIKTEGLKEGWYDGGSIFFAVFLVILVTATSD

YRQSLQFQNLNEEKRNIQLEVVRGGRTVKVSIYDLVVGDVVPLKIGDQVP

ADGVLIAGHSLAIDESSMTGESKIVHKNQNDPFLMSGCKVADGFGTMLVT

GVGINTEWGLLMASISEDTGEETPLQVRLNGVATFIGIVGLSVAVSVLAI

LLARYFTGNTEDPDGATQFIKGRTKFDDAFNDVVKIFTIAVTIVVVAVPE

GLPLAVTLTLAYSMRKMMADKALANTDTQVRRLSACETMGSATTICSDKT

GTLTLNEMTVVEAFVGKKKINPPADSSQLHLSVVSLLSEGVAQSTMGNVF

VSKDGGDVEISGSPTEKAILSWAIELGMKFDAIRSESTILHVFPFNSEKK

RGGVALRRDRNKDMNGRNICCPGLVDRSDAEVHIHWKGAAEIVLAACSGY

LDSNGCLQSMNEDKEFFEAAIDEMAANCLRCIALAYRLCEQEKVPSNEES

FDDWVLPEDNLVLLAIVGIKDPCRPGVKDAVKICTDAGVKVRMVTGDNIQ

TAQAIALECGILSSAQDVTEPTIIEGRVFRALTEIEREQVARKIMVMGRS

SPNDKLLLVQALRKGGDVVAVTGDGTNDAPALHEADIGLSMGIQGTEVAK

ESSDIIILDDNFASVVKVVRWGRSVYANIQKFIQFQLTVNVAALVINVAA

AISSGDVPLNSVQLLWVNLIMDTLGALALATEPPTDNLMHRSPVGRREPL

ITNIMWRNLLIQALYQVTVLLVLNFRGMTILQLEDDGNREHAYKVKNSLI

FNAFVMCQIFNEFNARKPEEVNCFKGVTKNYLFMGIIGFTFILQIIIIEF

LGKFTSTVRLDWQLWLVSLGIGIISWPLAIVGKLIPVPKTPVASYFIKPF

QQCKRSRDA

>GaACA7

MMGDSTLTSDKFCLNLVRILTWGHKLKSQVVIFVLFLADDLLQAALVLNA

SRRFRYTLDLRKQEEKEQRKRMIRAHAQVIRAALLFKLAGEKEIVSGTPV

TLPGAAGDFAVGLEQLASMTRDHKLSALQQYGGVKGLSDLLKTNLETGIY

GDEVDLLNRKTAFGSNTYPRKKGRSFWRFLWEAWQDLTLIILIVAASVSL

GLGIKTEGLKEGWYDGGSIFLAVLLVIVVTATSDYRQSLQFQNLNEEKRN

IQLEVLRGGRTVKVSIYDVVVGDVVPLKIGDQVPADGVLVTGHSLAIDES

SMTGESKIVHKDKKEPFLMSGCKVADGVGTMLVTGVGINTEWGLLMASIS

EDTGEETPLQVRLNGVATFIGIVGLSVAVSVLVVLLARYFTGHTEDPDGT

KQFIKGRTNFDDAFNGVVKIFTIAVTIVVVAVPEGLPLAVTLTRDLQVRR

LSACETMGSATTICSDKTGTLTLNEMTVVEAFVGKKKINPPSDSSQLPAS

VVSLLNEGVAQNSTGNVFVPKADSEVRIHWKGAAEIVLTSCSGYIDSNGC

LQSINEDKEFFKAAIDEMAVNSLRCVALAYRLCEKEKVPTDEEGFNGWIL

PEDNLVLLAIVGIKDPCRPGVKDAVKICMDAGVKVRMVTGDNIQTAKAIA

LECGILSSAEDATEPTIIEGRVFRELSDKEREQIAKKITVMGRSSPSDKL

LLVQALRKGGDVVAVTGDGTNDAPALHEADIGLSMGIQGTEVAKESSDII

ILDDNFASVVKVVRWGRSVYANIQKFIQFQLTVNVAALVINVVAAISSGD

VPLNSVQLLWVNLIMDTLGALALATEPPTDNLMHRSPVGRREPLITNIMW

RNLLLQIFNEFNARKPEEINCFKGVTKNYLFMGIIGFTFVLQIIIIEFLG

KFTTTVRLNWKLWLVSLGIGIISWPLAIVGKLIPVPKTPVSGYFTKAFRR

CRTDRNA

>GaACA1

MFAGASNIGGCNEHGDEDTLSVSLLNTTANSSRKNWTRLYNFIIFLRKAA

QIVRDKDLTSLEEEGGVRRVKSRLQSILAHEIDRSFEEQATAVIVTGTTF

DAKFFVVSFVEASKTPTIFPLLVFAALSFTIEMMEAEPKHGWHDGVAILV

AVFMLLIFRSVANYRRARRLQKRKKFHVSVLKDGQPKTITISTLVVGHIL

CLKKGDFVPADGLFVSENGLKLDDKLNPNINRDDNPFLLAGSKIMEGEGH

MLVTSVGDNSVLPTVDPDEKSLMEDQIDKTNAYMEYVGLSISLLVSALVL

INLLARKMDKNSNIMPEMKGDVSAYRVIKIFARVFLNPRGKVQIFTGVLT

VMVTSLQHGMPVVITISLSYWKKKMASGDANVQSLSSCGTIGIVSVICFD

ENTVVACKEVMGSIVGALKEEEVGCKLMSKDELATAQVMAHEIGILNPDL

RDMAIENKDLHELAATAEGMNKIAVIGSCKLKDKIRILQRLKQEGHVVAF

IGGMATNDDLALKAADVGITICECSTKMARENSEIVISSRNSLRSLIRCL

KMGKCAYGNVRTFTEIQLTATLASLLVTLVTTSILDESPITGIHMLWVNS

IICILGGLMMVMESYGHQELMNNQPARRMKSLLTKTMWRNVAIRAASDAC

HLLVLQFIGQAILQINKDVVKTMVFNGFILCQVLDLFISTIIIFARNEEV

SVSVVRFMCSSHWFLMASGGVMAMQVVVVELLQSLAEYERLNVMQWGFCF

IYAAWLCGTGLTVKLIADSASEVLWSSRSSGSLFGYLRLRRSSLRLFVIP

FSVCVVASFSYCYVNPDIA

>GaALA1

MKRYVYINDNESSHELYCDNRISNRKYTVLNFLPKNLWEQFSRFMNQYFL

FIACLQLWSLITPVNPASTWGPLIFIFAVSASKEAWDDYNRYLSDKKANE

KLVWVVRQGIRKQIQAQDIHVGNIVWLRENDEVPCDLVLIGTSDPQGLCY

VETAALDGETDLKTRVIPSACMGIDFELLHKIKAGVIECPNPDKDITRFD

ANLRLFPPFIDNDVCPLTIKNTILQSCYLRNTEWACGVAVYTGRNETKLG

MSRGIPEPKLTAMDAMIDKLTGAIFVFQIVVVMVLGIAGNVWKDTEARKQ

WYVQYPIEGPWYELLVIPLRFELLCSIMIPISIKVSLDLVKSLYAKFIDW

DAEMIDYETGIPSHATNTAISEDLGQVEYIMTDKTGTLTENRMIFRRCCI

SGVFYGNESGDALKDTKLLNAVAGGSPDVVQFLTVMAICNTVVPIKSKTG

AISYKAQSQDEDALVNAAAQLHMVYANKNANILEIRFNGSVIKYEVLEIL

EFTSDRKRMSVVVKDCQTGKIVLLSKGADEAILPYAFVGQQTRTFIEAVE

QYAQLGLRTLCLAFRELREDEYQEWSLMFKEASSTLVDREWRIAEVCQRL

EHDFEVLGVTAIEDRLQDGVPETIETLRKAGINFWMLTGDKQNTAIQIAL

SCNFISPEPKGQLLLIDGKTEDEVCRSLERVLLTMRITSSEPKDVAFVVD

GWALEIALKHYRKAFTELAILSRTAICCRVTPSQKAQLVELLKSCDYRTL

AIGDGGNDVRMIQQADIGVGISGREGLQAARAADYSIGKFRFLKRLILVH

GRYSYNRTAFLSQYSFYKSLVICFIQIFFSFISGVSGTSLFNSVSLMAYN

VFYTSVPVLVSVLDKDLSEGTVMQHPQILFYCQAGRLLNPSTFAGWFGRS

LFHAIVVFVITIHAYAYEKSEMEELSMVALSGCIWLQAFVVALETNSFTI

LQHLAIWGNLVAFYVINWIFSAIPASGMYTIMFRLCRQPSYWITMSLIVA

AGMGPVLALKYFRYTYRPSKINTLQQAERMGGPILTLGNIEPHPRPMEKE

VVSPLQISQPKNRNPVYEPLLSDSPNSSRRSLGSGAPFDFFQSQSRLSSS

YSRNCKDN

>GaALA2

MDSKTPVENLCCIEPALSSSSRRSNFSARSKSLGGSSIREVNFSDVGPKP

VRYGSQGAESETYSMSQKEINDEDARLVHINDPVNTNERFKFAGNSIRTA

KYSILTFLPRNLFEQFHRVAYVYFLVIAVLNQLPQLAVFGRTASILPLAF

VLLVTAVKDAYEDYRRHRADRIENNRLALVLVNNEFQQKKWKNIQVGEII

KIQANETIPCDMVLLSTSEPTGVAYVQTTNLDGESNLKTRYAKQETLQKI

PEKENVSGLIKCEKPNRNIYGFQANMEVDGKQVSLGPSNIILRGCELKNT

TWAVGVAVYAGSETKAMLNNSGAPSKRSRLETHMNLEIIFLSLFLVALCT

VVSVCAAVWLRRHRDELDYLPFYRRKDFSEDEEKNYNYYGWGLEIFFTFL

MSVIVFQIMIPISLYISMELVRVGQAYFMIRDAEMYDESSNTRFQCRALN

INEDLGQIKYVFSDKTGTLTENKMEFQCASIWGVDYNGGKATSQDQKDGY

FVQADGQVLRPKMVVKTDPELLQYVRNGKETKEGSYVHDFFLALAACNTI

VPIIVDTPDPTLKLIDYQGESPDEQALVYAAAAYGFMLIERTSGHIVIDI

QGERKRFNVLGLHEFDRDRKRMSVILGFPNQSVKVFVKGADTTMFSVIDR

SLNTSIIRATEAHLQSYSSIGLRTLVIGMRELSTSEFEEWHSAFEVASTA

LMGRARLLRKIASNIESNLCILGASGIEDKLQQGVPEAIESLRTAGIKVW

VLTGDKQETAISIGYSSKLLTSKMTQVIVNSNSKESCRKSLEDAIIMSKK

LTTTSGTTNETGRTLGTGSTPVALIIDGTSLVYILDSELEERLFELACNC

SVVLCCRVAPLQKAGIISLVKKRTSDMTLAIGDGANDVSMIQMADVGVGI

SGQEGRQAVMASDFAMGQFRFLVPLLFVHGHWNYQRMGYMILYNFYRNAV

FVLVLFWYVLFTCFTLTTAINEWSSVLYSVIYTSVPTIVVGILDKDLSRL

TLLKHPQLYGAGHRDECYNKTLFWITMLDTLYQSVVVFFIPLLAYWGSTI

DAASIGDLWTLAVVILVNLHLAMDVIHWNWITHAAIWGSIIATFICVIVI

DAIPSLVGYWAIFEIAKTRLFWFCLLAIIVTALIPRFVVKVLYQFYAPCD

VQIAREAEKFWAQSQSAAVEVEMSPILDHPRR

>GaALA3

MDSRTSFEDFYSIESAFSSSSRRSNFSVQSKASGGNSIREVNFGDLGTKP

VRYGSHGADSETYSISMSQKEINDEDARLVHINDPVQTNERFEFSGNSIR

TGKYSILTFLPRNLFEQFHRVAYIYFLLIAVLNQLPQLAVFGRGASILPL

AFVLLVTAVKDAYEDYRRHRSDRIENNRLASVLVDDQFQEKKWKNIQVGE

IIKIYANETIPCDMVLLSTSDPTGVAYVQTINLDGESNLKTRYAKQETLM

KIPENDEVIGLIKCEKPNRNIYGFQANMEVDGKQLSLGPSNIILRGCELK

NTAWAVGVAVYTGRETKAMLNSSGAPSKRSRLETHMNLEIIFLSLFLIAL

CTVVSICAAVWLRHHRKELDYLPFYRRKEFSDGEEENYNYYGWGLEICFT

FLMSVIVFQIMIPISLYISMELVRVGQAYFMIRDTRMYDESSNSRFQCRA

LNINEDLGQIKYVFSDKTGTLTENKMEFQCASIWGVDYSGGNAISLDQND

GYFVKVDGKVLRPKMKVKTDPELLQFARNGKETQEGSHVYDFFLALAACN

TIVPLIVDTPDPTVKLIDYQGESPDEQALVYAAASYGFMLIERTSGHIVI

DIQGERQRFNVFGLHEFDSDRKRMSVILGFPDRYVKVFVKGADTSMFSVI

DRSMDMKVIRTTEAHLHSYSSLGLRTLVVGMRELSTSEFKQWHSTFEAAS

TALMGRASLLRKVANNIENNLHILGASGIEDKLQQGVPEAIESLRTAGIK

VWVLTGDKQETAISIGYSSKLLTSKMTQIIINSKSMESCRKSLEDAIIMS

KKLTTTSAISGTTNNTGGTSGAGSTPIALITDGTSLVYILDSELEERLFQ

LSCNCSVVLCCRVAPLQKAGIVSLVKKRTADMTLAIGDGANDVSMIQMAD

VGVGISGQEGRQAVMASDFAMGQFRFLVPLLLVHGHWNYQRMGYMILYNF

YRNAVFVLVLFWYVLFTSFTLTTAITEWSSVLYSVIYTALPTIVVGILDK

DLSRRTLLKYPQLYRAGQKQECYNKKLFWITMIDTFWQSAVTFFIPLLAY

WESTIDASSIGDLWTLAVVILVNLHLAMDVNRWNWLTHAAIWGSIIATFI

CVIVIDALPFLVGYWAIFEIAKTGLFWLCLLAIIVAALIPHFVVKALYQL

YAPCDVQIAREAEKFRTLCESGAVEIEMNSILEVPRR

>GaALA4

LYSADCLSRQQFSLECPTTDSGNQVSWGSMELHNNDNTYTSFDISRSSSQ

AQENLSKSRRIRNKSVDFDDNLPYSENPRLIYINDPRRTNDKYEFTGNEI

RTSKYTLITFLPKNLFIQFHRVAYLYFLAIAALNQLPPLAVFGRTVSLFP

LLFVLCVTAIKDGYEDWRRHRSDRNENNREALVLQVGEFQRKKWKKIRAG

EVVKIHADETIPCDMVLLGTSDPSGLAYIQTMNLDGESNLKTRYARQETA

SSIFEGCNVSGLIRCEQPNRNIYEFTANMEFNGHKFPLSQSNIVLRGCQL

KNTDWIIGVVVYAGQETKAMLNSAVSPSKRSKLEGYMNRETFWLSIFLLV

MCSVVAVGMGLWLHRHKDELDTLPYYRKTYIREGRENGKTYRYYGIPMET

FFSFLSSVIVFQIMIPISLYITMELVRLGQSYFMIEDKHMYCSNSGSRFQ

CRSLNINEDLGQIRYVFSDKTGTLTENKMEFRKASVYGKDYRSSNLTDDS

LQDNSITDAAVRSRWKLKSEISVDSELMDLLHKDLAGDERIAAHLFFLTL

AACNTVIPIVSQDASSSHGSSDSSGEVKTIDYQGESPDEQALVSAASAYG

YTLHERTSGHIVIDINGNKLRLDVLGLHEFDSVRKRMSVVIRFPDNTVKV

LVKGADSTMFSILADTEKVDQIRQATRSHLTEYSSEGLRTLAVAARDLTD

AELEQWQCRYEDASTSLIDRAAKLRQTAALVECNLKLLGATAIEDKLQDG

VPEAIESLRQAGIKVWVLTGDKQETAISIGLSCKLLTADMQQIIINGNSE

EECRNLLTDAMTRHGVQPANRKKQNSTRRKNSENGYLEIPDDTKSSNVLQ

QHSGKEEPDVCAPLALIIDGNSLVYILEKDLQSELFDIATSCKVVLCCRV

APLQKAGIVDLIKSRTDDMTLAIGDGANDVSMIQMADVGVGICGQEGRQA

VMASDFAMGQFRFLKRLLLVHGHWNYQRVGYLVLYNFYRNAVFVLMLFWY

VPFFVVKSELFEFLGCIGQKKGGTY

>GaALA5

MSGWDNIRSSTRSQQGRSHSLNQREPSRTVTLGRVQPQAPAFRTIYCNDR

DANFAHRYRGNSVSTTKYNFFTFLPKGLYEQFRRVANLYFLMVSILSATP

YSPVHPVTNVVPLSLVLLVSLIKEAFEDWKRFQNDMAINSTPVDVLQDQR

WESIPWKKLQVGDIIRVKQDGFFPADMLLLASTNADGVCYIETANLDGET

NLKIRKALERTWDYVTPEKACEFKGEVQCEQPNNSLYTFTGNLVIDNQTL

PLSPNQILLRGCSLKNTEFVVGVVIFTGHETKVRYKSLTLSLIRIDAILW

EFGMSLQVMMNSMNVPSKRSTLERKLDKLILTLFGTLFTMCLVGAIGSGV

FIDRKYYFLGLSKSVEDQFNPNRRFLVVLLTMLTLLTLYSTIIPISLYVS

IEMVKFIQSTQFINKDLNMYHAETDTPALARTSNLNEELGQVEYIFSDKT

GTLTRNLMEFFKCTIGGEIYGTGMTEIERGVAERKGIKVQEVPTSINSVR

EKGFNFDDVRLMRGAWRNEPNPDACKEFFRCLAICHTVLPEGDESPEKIK

YQAASPDEAALVLAAKHFGFFFYRRTPTMIYVRESHVERMGKIQDVSYEI

LNVLEFNSTRKRQSVVCRYPDGRLVLYCKGADTVIYERLVGGSDDLKKVT

REHLENFGSAGLRTLCLAYKDLAPDVYESWNEKFIQAKSSLRDRERKLDE

VCLSMWYHFLLEETKYIFFSFVIMIFLSSLHLKKYIDSCIICFDFYFLFI

YYFHNNAVMILFTEILALISELIEKDLILIGATAIEDKLQEGVPDCIETL

RAGIKMWVLTGDKMETAINIAYACNLLNNEMKQFIISSETDAIREVEERG

DQVEIARFIKEEVKKQLKKCLDEAQQYFHSVSGPKLALIIDGKCLMYALD

PSLRIMLLNLSLNCSSVVCCRVSPLQKAQVTSLVKKGARKITLSIGDGAN

DVSMIQAAHIGVGISGLEGMQAVMASDFAIAQFRFLKDLLLVHGRWSYIR

LCKVVTYFFYKNLTFTLTQFWFTFYTGFSGQRFYDDWFQSLYNVIFTALP

VIIVGLFDKVPQFHLFLRILFPCYKLDLFKTLLDVQDVSSSLSKRYPELY

KEGIKNMFFKWRVVAIWAFFAVYQSLVFYYFVTVSSSTSQGSSGKMFGLW

DVSTMAFTCVVVTVNLRLIMICNSITRWHYISVGGSIVAWFLFIFLYSGI

MTPYDRQENIFWVIYVLMSTFYFYITLLLVPVAALLGDFLYLGVQRWFFP

YDYQIVQEIHKDEADDSGRTDLLGIDNQLTPDEARSYALSQLPRELSKHT

GFAFDSPGYESFFASQLGVYAPQKAWDVARRASMRSKPK

>GaALA6

MSGWGTPSRTVTLGRVQPQAPALRTIYCNHREANFAHRYKGNSISTTKYN

VFTFLPKGLYEQVILLEFYRTETGIWLCDSLGISYDNFLLFRRVANLYFL

MVSILSATPYSPVHPITNMVPLSLVLLFSLIKEAFEDWQRFQNDMTINNT

LVDVLQAQGWESLQWKKLQVGDIIRVKQDGFFPADLLLLASTNVDGICYI

ETANLDGETNLKIRKALERTWDYVTPEKACEFKGEVQCEQPNNSLYTFTG

NLVMDNQTMPLSPNQILLRGCSLKNTDYIVGTVIFTGHETKVMMNSMNVP

SKRSTLERKLDKLILALFSTLFSMCLLGAIGSGIFIDRKYYYLGLSQSVE

DQFNPSKRFLVIILTMLTLLTLYSTIIPISLYVSIETIKFIQSSQFINKD

LNMYHAESNTPASARTSNLNEELGQVEYIFSDKTGTLTRNLMEFFKCSIG

GEMYGTGMTEIEMGVAERKGIKVQEAKTSTNSLREKGFNFDDVRLMQGAW

RNEPNHDACKEFFRCLAICHTVLPEGEETPEKIRYQAASPDEAALVLAAK

NFGFFFYRRTPTMIYVRESHVEKMGKTQDVSYEILNVLEFNSTRKRQSVV

CRYRDGRLVLYCKGADTVIYERLGTGNGDLNKVTRVHLEQFGSAGLRTLC

LAYRDLAPDLYESWNEKFITAKSSLRDREKRLDEVAELIEKELILIGATA

IEDKLQEGVPNCIQTLSRAGIKIWVLTGDKIETAINIAYACNLLNNEMKQ

FIISSETDAIREVEERGDQTEIARFMKEEVKKQLKQFLDEAPQYFCSPSG

PKLALVIDGKCLMYALEPSLRIMLLTLSLNCSSVVCCRVSPLQKAQVTSL

VKKGARKITLSIGDGANDVSMIQAAHIGIGISGLEGMQAVMASDFAIAQF

RFLEDLLLVHGRWSYIRLCKVVMYFFYKNLAFTLTQFWFTFGTGFSGQRF

YDDWFQSLYNVIFTALPVVIVGLFDQDISSSLSKKYPELYKEGDNMFWVI

YVLMSTSYFYVTLLLVPVAALLGDFLYLGVQRWFFPYDYQIVQESHKDDA

DSINKPDSQETKEHLTPDEARSNEISQLPKEVSKHSGFAFDSPGYESFFA

TQFGKYVPPKAWDVARRASMKSKSKPK

>GaALA7

MAKGKIRAKIKRSQLYTFACGRKSTQEAVPFVEGAGCSRTVHCNQPHMHR

KRPLKYRSNYISTTKYNFLSFLPKSLYEQFHRVANLYFLAAAVLSLTPLS

PFSAVSMIAPLAFVVGLSMAKEGLEDWRRFMQDMNVNSRKVKFHKEGGVF

EFKQWQQVEVGDVVRVEKDEFFPADLLLLSSSYEDGICYVETMNLDGETN

LKVKRAMDVTLSLEEDEDFKNFTGLIKCEDPNPSLYTFVGNLDYDRQTYS

LEPAQVLLRDSKLRNTGFVYGVVIFTGFDTKVMQNSTKSPSKRSRIEKKM

DYIIYVLFSLLLVISLISSLGFALKTKFHMPNWWYMRPDDTEQFYNPKEP

FISGLSHLVTALILYGYLIPISLYVSIEVVKVLQASFINQDLQMYDDDTG

IPAQARTSNLNEELGQVDTILSDKTGTLTCNQMDFLKCSIAGTAYGVRAS

EVERAAAQQMANDLEQDGRQSSVSSKRSKQDIELETVVTSKDGKDNKPPI

KFFSFEDDRMTEGNWRKEPNVDMIILFCRILAVCHTAIPELNEETGTYTY

EAESPDEGAFLVAAREFGFEFFKRTQTSVFVRERYIGSGQTTEREYKILN

LLEFTSKRKRMTVIVRDEEGQIILMCKGADSIIFDRLAKKGKTYLEDTTK

HLNIYGEAGLRTLALAYRKLEESEYSAWNNEFQKAKTSIGADRDTNLEKV

AEMMEKELTLVGATAVEDKLQNGVPQCIDKLAQAGLKLWVLTGDKMETAI

NIGFACSLLREGMKQICITTLDSESKEAIKENILTQVNNGSQMIKLEKDP

YAAFALIIDGKTLAYALEEDMKLLFLGLAVDCASVICCRVSPKQKALVTR

LVKEGTGKVTLGIGDGANDVGMIQEADIGVGISGVEGMQAVMASDFSVSQ

FRFLERLLVVHGHWCYKRIAQMVCYFFYKNIAFGLTLFYFEAFTGFSGQS

VYDDWYMILFNVVLTSLPVISLGVFEQDVSSDVCLEFPTLYQQGPRNLFF

DWYRILGWMGNGLYSSLVIFSLNIVIFYDQAYRVEGQTTDMSILGTIMFT

CIICALNFQVSLIMTHYTWMQHVLIWFSIVAWFIFLFIYGMLPPKISHSA

YQILTEALASAPVYWITTLLVTIACTLPYLAHMSLQRCFNPMDHHVIQEI

KYLKKDVSDQVMWRREKTKAREKTKIGFTARVDALIRSLRGKLNKKTHSI

ESNKSYPPS

>GaALA8

MARVRKRNIHLSKSCSFSCCRRPSQPSSTDEHDRIGQKGYSRVVYCNEPD

CEEQIRLKYRGNYVSTTKYTAANFIPKSLFEQFRRVANIYFLVVACVSFS

PLAPYSAPSVLVPLIVVIGATMAKEGIEDYRRRQQDVEANNRTVEVYDGN

SSFNETKWKNLRVGDIVKVHKDEYFPADLLLLSSEYEDGVCYVETMNLDG

ETNTKSKHPLEVTSFIRDVETVKGFRAVIKCEDPNEHLYSFVGTLYYECQ

QYALSPQQILLRGTKLKNTDYINGVVIFTGHDTKVMQNAMDPPSKRTRIE

RRMDRIIYVLFSALILVSFVGSLLFGIETKKDGGDYGRWYLRPDITTVFF

DPRRPSVAAFLHFLTGLMLYGYLIPISLYVSIEICKVLQSIFINQDQAMY

DEETKRSAHARTSNLNEELGQVCTILSDKTGTLTCNSMEFVKCSIAGTAY

GRGMTEVEIALARKRGETLDEQRDIDTVESREPVKGFNFRDGRIMNRKWV

NEPNRDYIEKFFRVLALCHTAVPEVLDPGKIFYEAESPDEAAFVIAAREV

GFQFFKRNQTSIQLRELDRSSGEIVDRVYELLHVLEFSSARRRMSVIVRN

PERQLLLLAKGADSVIFERLSEEGRMFEAETKEHIERYSEAGLRTLAVAY

RELDDDEYKRWEQEYVKAKTSVSADRDDLLDEKADMIERGLILLGATAIE

DKLQKGVPDCIDKLAQAGIHIWVLTGDKKGTAINIGYACSLLRHGMKEIL

VILEKPGIEAIERDKEEFAKAYKIVEKQIDEGISQVTGESNTQFGLIIDG

KSLIFALDKNLIIRFMELAMKCATVICCRSSPKQKAIVTRWVKSKTGRTT

LAIGDGANDVGMIQEADIGVGITGVEGMQAAMSSDFSIAQFHFLERLLLV

HGHWCYRRITMMICYFFYKNITFGFTLFWFEAYSSFSAQPAYNDWYMSSY

NVLFTSLPVIALGVFEQDFSARHCLKYPSLYKEGIDNVLFRWIHILGWMF

NGVLSSVIIFFLTTNSITGQAFRKDGQVADYSILGLTMYTCVVLAVNCQM

ALSINYFTWIQHLFIWGSITLWFKFLLVYGSIPPTLSTTAYKVFIEACAP

SIRYWLTIILVVIATLLPLFSYRAFQTRFQPMVHDSIQTRRP

>GaALA9

MARIRKRSIQLSKLCSFACCRSSSQDEHAQIGQKGYSRVVYCNEPDSQEQ

IRLRYRGNYVSTTKYTAVNFIPKSLFEQFRRVANIYFLVVACVSFSPLAP

YSAPSIILPLVVVIGATMAKEGVEDWRRNLQDIEANNRKVEVYDKSSCSF

RQTKWKDLRVGDLVKVCKDEYFPADLLLLSSSYEDVVCYVETMNLDGETN

LKLKHALNVTSFYSSAESLKEFRAVIKCEDPNEHLYSFIGTLYFDCQQYS

LSPQQILLRDSKLKNTEYIFGVVIFTGHDTKVMQNATDPPSKRTKIERRM

DRIIYVLFSTLILVSFIGSLFFGIETNNDLSGGNYTRWYLRPDKTTVFFD

PRRPAVAAFLHCLTGLMLYGYLIPISLYVSIEICKVLQSIFINQDQAMYD

EEMDRPAHARTSNLNEELGQVHTILSDKTGTLTCNSMEFVKCSIAGTLYG

RGMTEVEIALARKRGEQLTEQAPIDASESKKSIKGFNFRDERIMDGKWVS

EPQSDVIQKFFRVLATCHTAVPEVMGPDEIMYEAESPDEAAFVIAAREVG

FEFFARNQTSIKLRELDPTSGIRVDRVYELLHVLEFSSARKRMSVIVKNP

ENQLLLLAKGADSVIFERLSKEGRAYEERTKEHIEKYSEAGLRTMAVAYR

ELDNDEYKTWEQEFLKAKTSVSADRDVLMDELACQIERDLILLGATAVED

KLQKGVPGCIDKLARAGIGIWVLTGDKRGTAINIGYACSLLRHGMKQIVI

TLELPEILAQEKEGDKEAIAEASLNSIKLQILKAKSQVTEESSTEFGLII

DGKSLTYAMDKNLVNSFMDLAMRCATVICCRSSPKQKAVITRLVKSVTGR

TTLAIGDGANDVGMLQEADIGVGISGVEGMQAVMASDFAIAQFRFLERLL

LVHGHWCYRRISMMICYFFYKNITFGFTLFWFEAYTSFSGQPAYNDWYMS

CYNVFFTSLPVIALGVFDQDVSARLCLKHPLLYQEGVRNILFNWPRILGW

MFNGLLSSIIIYFLTTNSITGPAFRKDGQVADYSVLGVTMYTCVVWTVNC

QMALSINYFTWIQHLFIWGSIAFWYIFLVVYGSIPPTLSTTAYKVLVEAC

APSILYWLTTLLVVIAALLPLLSYRAFQIRFRPMEHDRIQIQRRRPESTE

YDATQRRLSESSEARTSSELPSEVRIRIDNLKASSKRKN

>GaALA10

MTGGRRRKQHFSRIHAFSCGKASFKGDHSLIGGPGFSRVVYCNDPECFEA

SLRNYAGNSVRSTKYTLATFFPKSLFEQFRRVANFYFLICAILSFTPLSP

YSAVSNVLPLVVVIGATMGKEAVEDWRRKKQDTEVNNRKVKMHQSDGIFE

PTKWMDLKVGDIVKVEKDEFFPADLILLSSSYEEAICYVETMNLDGETNL

KLKQASDVTSSLHDDASFQDFKATIRCEDPNANLYSFVGSLELGDEQYPL

SPQQLLLRDSKLRNTDYIFGVVIFTGRDTKVIQNSTEPPSKRSKIEKRMD

NIVYFLFAVLVGLSIIGSIFFGIETREDLENGKMRRWYLRPDDTTIYYNP

KRAAVAAILQFLTALMLYSYLIPISLYVSIEIVKVLQSIFINQDLHMYHE

ETDKPAHARTSNLNEELGQVDTILSDKTGTLTCNSMEFIKCSIAGTSYGH

GITEVERALAWRKGSPLAREVPEINDQVEEFKKEKPSVKGFNFVDERIMN

GNWLKEPHADVIQKFLRLLAICHTAIPEVDEETGRISYEAESPDEAAFVV

AARELGFEFYERTQTSISLYEFDLSGKKVERSYKLLNILEFSSSRKRMSV

ILQNEEGKLLLLCKGADSVMFERLAKNGQEFAEQTKEHIEEYADAGLRTL

VLAYREINEEEYVEFNEKFTEAKNIVSADREEMIEEVAESIERDLILLGA

TAVEDKLQNGVPECIDKLAQAGIKIWVLTGDKMETAINIGFACSLLRQGM

KQIIINSDTPEDKALEKSGDKTAAAAAYKASVLQQIAEGRLLLTSSNENS

EALALIVDGKSLTYALEDDVKDAFLELAIGCASVICCRSSPKQKALVTRL

VKTKTRSTTLAIGDGANDVGMLQEADIGVGISGVEGMQAVMSSDIAIAQF

RFLERLLLVHGHWCYRRISSMICYFFYKNIVFGFTLFFYEIYASFSGQAV

YNDWFLSFYNVFFTSLPVIALGVFDQDVSSRLCLKFPLLYQEGIQNVLFS

WLRIIAWAFNGVLSATVIFFFCIRAVQHQAFRKGGEVVGLEILGTAMYTC

VVWVVNCQMALSVSYFTYIQHLFIWGSIILWYIFLMAYGAMDPSISTTAY

KVFIESCAPAGMYWLLTLLVLISSLLPYFIYSAIQVRFFPSYHQMIQWIR

SDGQSDDPEYCHMVRQRSLRPTTVGYTARLEAKSRSSRKGGEDHQ

>GaALA11

MSGGRRRKVLMSRIYGIACGKASFKEDHSQIGGPGFSRVVYCNEPNSLEA

GTRNYSDNYVSTTKYTIATFLPKSLFEQFRRVANFFFLVTGILSFTAIAP

YSALSAIVPLIIVIGATMIKEGVEDWRRQQQDIEVNNRKVKVHQGDGNFH

HTEWKNLRVGDIVKVEKDEFFPTDLILLASSYEDAVCYVETMNLDGETNL

KLKQALEVTSSLHDDYNFRDFKAIVKCEDPNANLYSFVGTMEFEEQQHPL

SPQQLLLRDSKLRNTDYIYGAVVFTGHDTKVMQNATDPPSKRSKIEKTMD

RVIYLMFFIVFIMGFIGSIFFGIATENDYEGGRIKRRWYLRPDNAEIFFD

PERAPVAAIYHFLTALLLYSYFIPISLYVSIEIVKVLQSIFINQDSHMYY

EEADKPAHARTSNLNEELGQVDTILSDKTGTLTCNSMEFIKCSIAGTAYG

RGVTEVERAIYRKKGSPVVHEPNGLNHIEDSADVNPAIKGFNFKDERIMN

GNWVNEPRADVIQKFFRLLAICHTAIPEVDEENGNISYEAESPDEAAFVI

AARVLGFEFHNRTQTSISLHELDPVSGKRVNRLFKLLNVLEFDSSRKRMS

VIVRDEEGKLLLLCKGADSVMFERLAKGGRDFEEDTREHMNEYADAGLRT

LVLAYRELSQNEYEVFNEKMTEAKNSVSADRETLIDEVAEMIERDLILLG

ATAVEDKLQNGVPDCIDKLAQAGIKLWVLTGDKMETAINIGYACSLLRQG

MKQIIINIDTPEIQSLEKTGDKDAVIKASRKSVMEQIVSGKAQVSALSAI

SEAFALIIDGKSLAYALEDDMKNSFLELAIGCASVICCRSSPKQKALVTR

LVKLGTGKTTLAIGDGANDVGMLQEADIGIGISGVEGMQAVMSSDVAIAQ

FRYLERLLLVHGHWCYRRISSMICYFFYKNIAFGFTIFLYEAYTSFSAQP

AYNDWYLTLFNVFFSSLPVIAMGVFDQDVSAWFCLKFPLLYQEGVQNVLF

SWRRIVSWMFNGFYSAIIIFFFCSRALEQQAFNDEGKTASKDILGGTMYT

CIVWVVNLQMALSISYFTLIQHIVIWGTIAFWYVFQLAYGALPASFSTDA

YRVFVEALAPAPSYWFITLFVVIATLTPYFLYSAIQMRFFPMYHEMIQWI

RHEGLSDDPLYCEMVRQRSIRPTTVGFTARRAASRRQ

>GaP5

MSRFHVGGKVVDKVDLLRKKHAAWRLDVWPFAMLYLLWLTMVVPSIDFVD

AAIVLGGLAVTHILVLLFTTWSVDFKCFVQYSKVNNIRLADVCKVTPAKF

CGSKEIASSSSAKDVEEIYFDFRKQCFIYSKEEDTFCKLPYPTKETFGYY

LKCSGHGSDAKVLAATEKWGRNVFEYPQPTFQKLMKEHCMEPFFVFQVFC

VGLWCLDEYWYYSLFTLFMLFMFESTMAKSRLKTLSELRRVRVDSQTLMV

HRCGKWVKLSGTDLLPGDVVSIGRSSGQNEEDKSVPADMLILAGSAIVNE

AILTGESTPQWKVSIAGRGIEEKLSAKRDKNHMLFGGTKILQHTADKSFP

LRTPDGGCLAVVLRTGFETSQGKLMRTILFSTERVTANSWESGLFILFLV

VFAIIAAGYVLKKGLEDPTRSKYKLFLSCSLIITSVIPPELPMELSIAVN

TSLIALARRGIFCTEPFRIPFAGKVDICCFDKTGTLTSDDMEFSGVVGLN

DSSELESDMTKVPSRTVEILASCHALVFVDNKLVGDPLEKAALKGIDWSY

KSDEKAIPKKGSGNPVQIVQRHHFASHLKRMAVVVRVQEDFFSFVKGAPE

TIQDRLIDLPPTYVETYKKYTRQGSRVLALAYKSLPDMTVSEARSMERDT

VECGLTFAGFAVFNCPIRADSSTVLSELKNSSHDLVMITGDQALTACHVA

GQVNIVSKPALILVAVKNSKGYEWVSPDETERIPYSENEVEALSETHDLC

IGGDCFEMLQQTSAVLRVIPFVKVFARVAPEQKELIMTTFKTVGRLTLMC

GDGTNDVGALKQAHVGVALLNAVPPTKSESSSGTSKDENTKSLKSKSKPT

VEATGNSEASSKGKVVPRSESSNNATSNRHLNAAEKHRQKLKKMMDELNE

EGDGRSAPIVKLGDASMASPFTAKHASVAPTTDIIRQGRSTLVTTLQMFK

ILGLNCLATAYVLSVMYLDGVKLGDVQATISGVFTAAFFLFISHARPLPT

LSAARPHPNIFCSYVFLSLMGQFAMHLLFLISSVKEAEKHMPEECIEPES

EFHPNLVNTVSYMVSMMLQVATFAVNYMGHPFNQSIPENKPFLYALGAAA

GFFVVITSDIFRDLNDWLSLVPLPVGLRDKLLIWALLMFLCCYAWERLLR

WAFPGKIPAWRKRQRVAAASSEKKLV

>GaAHA11

MDTEKKAVALEAINKEIVDLENIPIEEVLEKLKCTKEGLTTDEVQQRLELFGYNKLEEKKENKILKFLGFMWNPLSWVMEAAAVMAIALAHGGKKETDYHDFVGILALLLINSTISFIEENNAGNAAAALMARLAPKAKVLRDGKWNEEDASVLVPGDIISIKLGDIIPADARLLQGDPLKIDQSALTGESLPVTKHPGDGVYSGSTCKQGEIEAVVIATGVHTFFGKAAHLVESTTHVGHFQQVLTSIGNFCICSIAIGMLAELIVIYGAQRRSYRTGIDNLLVILIGGIPIAMPTVLSVTMAIGSHRLSQQGAITKRMTAIEEMAGMDVLCSDKTGTLTLNKLTVDKNLIEVFVNNVDKDTVILMAARASRLENQDAIDTAIVAMLADPKEARAGITEVHFLPFNPTDKRTALTYIDEAGKMHRVSKGAPEQILNLAYNKSEIGKKVHSIIDKYAERGLRSLAVARQEVPAGTKDSPGGPWEFVGLLPLFDPPRHDSAETIRRALDLGVSVKMITGDQLAIGKETGRRLGMGTNMYPSSSLLGDHKNEEIGAFSIDELIENADGFAGVFPEHKFEIVKRLQAKKHIVGMTGDGVNDAPALKKADIGIAVADSTDAARSASDIVLTEPGLSVIISAVLTSRAIFQRMKNYTIYAVSITIRIVLGFMLLTLIWRLNFPPFMVLIIAILNDGTIMTISKDRVKPSPVPDSWKLKEIFATGIVLGSYLALMTVIFFWAAYETDFFPDKFGVRSLSKKSFDLKQESERRKVNALLSSAVYLQVSTISQALIFVTRSRGWSFTERPGLLLVTAFIIAQLVATVISAQATWSFAGIRAVGWGWCGVIWIYNILTYFLLDPIKFAVRYALSGKAWDLVLNQRTAFSTQKDFGKEAREAAWAAEQRTLHGLQSTSDAKITEKHNFRDISVMAEEARRRAEIARLREIHTLKGKVESFAKLRGLDIDVNPHYTV

>GaAHA9

MADKSISLEEIKNETVDLVSISFPRIQPERIPVEEVFQQLKCTRNGLTSEEGQKRLQIFGPNKLEEKKAKENKVLKFLGFMWNPLSWVMEFAAIMAIALANGGGKPPDWQDFIGIVSLLFINSTISFIEENNAGNAAAALMAGLAPKTKVLRDGKWNEQEAAILVPGDIISIKLGDIVPADARLLEGDSLKIDQSALTGESLPVNKHSGDEVFSGSTVKQGEIEAVVIATGVHTFFGKAAHLVDSTNNVGHFQQVLTAIGNFCICSIGVGMLIEIVVMYPIQRRKYRDGIDNLLVLLIGGIPIAMPTVLSVTMAIGSHRLSQQGAITKRMTAIEEMAGMDVLCSDKTGTLTLNKLTVDKSMIEVFMDNIDKEMVLLLAARASRVENQDAIDACIVGMLGDPKEARAGVTEVHFFPFNPVDKRTAMTYIEADGSWHRVSKGAPEQIIELCNLRNDAKRRAHDIITKFADRGLRSLAVAKQKVPEKTKDGQGDPWQFVGLLPLFDPPRHDSAETIRRALSLGVNVKMITGDQLAIGKETGRRLGMGTNMYPSSVLLGENKGDALDTIGVDELIEKADGFAGVFPEHKYEIVKRLQQRKHICGMTGDGVNDAPALKKADTGIAVDDATDAARSASDIVLTEPGLSVIVSAVLTSRAIFQRMKNYTIYAVSITIRIVLGFMLLALIWKFDFSPFMVLIIAILNDGTIMTISKDRVKPSPMPDSWKLKEIFCTGIVLGTYLACMTLVFFWAANDSNFFSDKFGVRSIRHNQDELTAAVYLQVSIVSQALIFVTRSRSWSFIERPGFLLVIAFILAQLVATVIAVYANWGFARIKGIGWGWAGVIWIYSVVFYFPLDVIKFLIRYAMSGKAWNNLLQNKTAFTTKKDYGKGEREAQWALAQRTLHGLTPPEMTEKSNYIELSEIAEQARKRAEVARLRELHTLKGHVESVVKLKGLDIDTIQQHYTV

>GaAHA10

MAETSVGLEDIKNETIDLERIPVHEVFQKLKCTRDGLSSEEGQKRLHIFGPNKLEEKQESKVLKFLGFMWNPLSWVMEIAAIMAIALANGGGKPPDWQDFIGIVALLLINSTISFIEENNAGNAAAALMAGLAPKTKVLRDGKWSEQEAAILVPGDIISIKLGDIVPADARLLEGDPLKIDQSALTGESLPVNKNAGDEVFSGSTVKQGELEAIVIATGVHTFFGKAAHLVDSTNNVGHFQQVLTAIGNFCICSIGVGMLIEIVVMYPIQQRRYRDGIDNLLVLLIGGIPIAMPTVLSVTMAIGSHRLSQQGAITKRMTAIEEMAGMDVLCSDKTGTLTLNKLTVDKSLVEVFTNDVDKDMVILLAARASRVENQDAIDACIVGMLGDPKEARAGITEVHFFPFNPVDKRTAMTYIKADGSWHRVSKGAPEQIIDLCNLRDDVKRRAHDIIANFADRGLRSLAVARQTVKEKNKDAQGEAWEFVGILPLFDPPRHDSAETIRRALNLGVNVKMITGDQLAIGKETGRRLGMGTNMYPSSALLGQNKGDTIDTIGVDELIEKADGFAGVFPEHKYEIVKRLQQRNHICGMTGDGVNDAPALKKADIGIAVDDATDAARGASDIVLTEPGLSVIVSAVLTSRAIFQRMKNYTIYAVSITVRIVLGFMLIALIWKFDFSPFMVLIIAILNDGTIMTISKDRVKPSPMPDSWKLKEIFATGIVLGTYLACMTVVFFWAANDSNFFQDKFGVRSIRHNQDELTAAIYLQVSIVSQALIFVTRSRSWSFIERPGLLLVVAFILAQLIATLLAVYANWGFARIKGIGWGWAGVIWLYSLVFYVPLDVLKFLIRYSLSGKAWDNLLQSKTAFTTKKDYGKGEREAQWAMAQRTLHGLTPPEMTQLYNEETNYRELSEIAEQARKRAEVARLRELHTLKGHVESVVKLKGLDIDTIQQHYTV

>GaAHA13

MGDKNEVLEAVLKETVDLENIPIEEVFENLRCSREGLTTEAAEERLTIFGHNKLEEKKESKFLKFLGFMWNPLSWVMEAAAIMAIALANGGGKPPDWQDFVGIITLLVINSTISFIEENNAGNAAAALMARLAPKAKVLRDGRWNEQDAAILVPGDIISIKLGDIIPADARLLEGDPLKIDQSALTGESLPVTKGPGDGIYSGSTCKQGEIEAVVIATGVHTFFGKAAHLVDTTNQVGHFQKVLTAIGNFCICSIAVGMVIEIIVMYPIQDRDYRPGIDNLLVLLIGGIPIAMPTVLSVTMAIGSHRLSQQGAITKRMTAIEEMAGMDVLCSDKTGTLTLNKLTVDKNLIEVFAKGVDADTVVLMAARASRTENQDAIDSAIVGMLADPKEARAGIREVHFLPFNPTDKRTALTYIDSDGKMHRVSKGAPEQILHLAHNKTDIERRVHAVIDKFAERGLRSLAVAYQEVPDGRKESPGGPWQFIGLMPLFDPPRHDSAETIRRALNLGVNVKMITGGFLLQLNVVIDFSGRGNGAMKKASEDIYRDQLAIGKETGRRLGMGTNMYPSSALLGQDKDESIAALPVDELIEKADGFAGVFPEHKYEIVKRLQARKHICGMTGDGVNDAPALKKADIGIAVADATDAARSASDIVLTEPGLSVIISAVLTSRAIFQRMKNYTIYAVSITIRIVLGFMLLALIWKFDFPPFMVLIIAILNDGTIMTISKDRVKPSPLPDSWKLAEIFTTGIVLGSYLAVMTVIFFWAAYKTNFFPRVFGVATLEKTAHDDIKKLASAVYLQVSIISQALIFVTRSRSWSFVERPGLLLLAAFVIAQLIATLIAVYANWSFAAIEGIGWGWAGVIWLYNIIFYIPLDFIKFFIRYALSGRAWDLVIEQRIAFTRQKDFGKEQRELQWAHAQRTLHGLQAPDTKMFTERTHFTELNQMAEEAKRRAEIARLRELHTLKGHVESVVRLKNLDIDTIQQAYTV

>GaAHA4

MAGISLEEIKNETVDLEKIPIEEVFEQLKCTREGLSSDEGVNRIQIFGPNKLEEKKESKILKFLGFMWNPLSWVMEAAAIMAIALANGEGKPPDWQDFVGIVCLLVINSTISFIEENNAGNAAAALMAGLAPKTKVLRDGKWTEQEAAILVPGDIISIKLGDIIPADARLLEGDPLKVDQSALTGESLPVTKNPGDEVFSGSTCKQGEIEAVVIATGVHTFFGKAAHLVDSTNQVGHFQKVLTAIGNFCICSIAIGMLVEIVVMYPIQHRKYRDGIDNLLVLLIGGIPIAMPTVLSVTMAIGSHRLSQQGAITKRMTAIEEMAGMDVLCSDKTGTLTLNKLSVDKNLIEVFVKDADKEHVVLLAARASRTENQDAIDAAIVGMLADPKEARAGIREVHFLPFNPVDKRTALTYIDSNGNWHRASKGAPEQILALCNAKEDLKKRVHSIIDKFADRGLRSLAVARQQVPEKTKESAGTPWQFVGLLPLFDPPRHDSAETIRQALHLGVNVKMITGDQLAIAKETGRRLGMGTNMYPSASLLGQDKDASIAALPVEELIEKADGFAGVFPEHKYEIVKKLQERKHICGMTGDGVNDAPALKKADIGIAVADATDAARSASDIVLTEPGLSVIISAVLTSRAIFQRMKNYTIYAVSITIRIVFGFLFIALIWKFDFSPFMVLIIAILNDGTIMTISKDRVKPSPLPDSWKLKEIFATGIVLGGYLALMTVIFFWAMHDTDFFSDKFGVRSLREREHEMMGALYLQVSIVSQALIFVTRSRSWSYAERPGLLLVTAFIIAQLVATLIAVYANWGFARIKGIGWGWAGVIWLYSIVFYVPLDIMKFAIRYILSGKAWLNLLENKTAFTTKKDYGKEEREAQWALAQRTLHGLQPPETSNLFNDKNSYRELSEIAEQAKRRAEVARLRELHTLKGHVESVVKLKGLDIDTIQQHYTV

>GaAHA1

MGNDKGISLEEIKNESVDLERIPIEEVFEQLKCTRGGLTSEEGTNRLQVFGPNKLEEKKESKFLKFLGFMWNPLSWVMEAAALMAIALANGDGRPPDWQDFVGIIALLLINSTISFIEENNAGNAAAALMANLAPKTKVLRDGRWSEQDAAILVPGDIITIKLGDIVPADARLLEGDPLKIDQSALTGESLPVTKNPSDEVFSGSTCKQGEIEAVVIATGVHTFFGKAAHLVDSTNQVGHFQKVLTAIGNFCICSIAVGIIIEIIVMYPIQHRKYRDGIDNLLVLLIGGIPIAMPTVLSVTMAIGSHRLSQQGAITKRMTAIEEMAGMDVLCSDKTGTLTLNKLTVDRNLIEVFAKGVEKEHVILYAARASRTENQDAIDAAIVGMLADPKEARAGIQEIHFLPFNPVDKRTALTYIDAAGNWHRASKGAPEQIITLCNCKEDVKKKVHAVIDKFAERGLRSLAVARQDVPEKSKDGPGAPWQLIGLLPLFDPPRHDSAETIRRALNLGVNVKMITGDQLAIAKETGRRLGMGTNMYPSSSLLGQDKDASIASLPVDELIEKADGFAGVFPEHKYEIVKRLQERKHICGMTGDGVNDAPALKKADIGIAVADATDAARSASDIVLTEPGLSVIISAVLTSRAIFQRMKNYTIYAVSITIRIVFGFMFIALIWKFDFAPFMVLIIAILNDGTIMTISKDRVKPSPQPDSWKLKEIFSTGIVLGGYLALMTVVFFWVMKDTDFFPNMFNVRSLADSPEEMMAALYLQVSIVSQALIFVTRSRSWSYVERPGLLLLTAFVIAQLVATLIAVYANWGFARIKGMGWGWAGVIWLYSVVTYIPLDLIKFAIRYVLSGKAWDNLLENKTAFTTKKDYGKEEREAQWAAAQRTLHGLQPPETSNLFNEKSSYRELSEIAEQAKRRAEVARLRELNTLKGHVESVVKLKGLDIDTIQQHYTV

>GaAHA5

MGAISLEEIKNETVDLEKIPIEEVFEQLKCSREGLSSEEGANRLQIFGPNKLEEKKESKILKFLGFMWNPLSWVMEAAAIMAIALANGDGKPPDWQDFVGIVCLLVINSTISFIEENNAGNAAAALMAGLAPKTKVLRDGKWSEQEAAILVPGDIISIKLGDIIPADARLLEGDPLKIDQSALTGESLPVTKNPGDEVFSGSTCKQGEIEAVVIATGVHTFFGKAAHLVDSTNNVGHFQKVLTAIGNFCICSIAIGMLVEIIVMYPIQHRKYRDGIDNLLVLLIGGIPIAMPTVLSVTMAIGSHRLSQQGAITKRMTAIEEMAGMDVLCSDKTGTLTLNKLSVDRNLIEVFVKDVDKEHVVLLAARASRTENQDAIDAAIVGMLADPKEARAGIREVHFLPFNPVDKRTALTYIDSNGNWHRASKGAPEQILTLCNAKEDVKKKVHAIIDKFADRGLRSLGVARQLVPEKSKDGAGTPWQFVGLLPLFDPPRHDSAETIRRALNLGVNVKMITGDQLAIAKETGRRLGMGTNMYPSASLLGQDKDASIAALPVEELIEKADGFAGVFPEHKYEIVRKLQERKHICGMTGDGVNDAPALKKADIGIAVADATDAARSASDIVLTEPGLSVIISAVLTSRAIFQRMKNYTIYAVSITIRIVFGFMLIALIWKFDFSPFMVLIIAILNDGTIMTISKDRVKPSPLPDSWKLKEIFATGIVLGGYLALMTVVFFWIMHDTDFFSEKFGVRSLRKRDDQMMGALYLQVSIVSQALIFVTRSRSWSYFERPGLLLVTAFIIAQLVATVIAVYANWGFARIQGIGWGWAGVIWLYSVVFYIPLDLMKFAIRYILSGKAWLNLLENKTAFTTKKDYGKEEREAQWALAQRTLHGLQPPEASNLFNDKSSYRELSEIAEQAKRRAEVARLRELHTLKGHVESVVKLKGLDIDTIQQHYTV

>GaAHA6

MGAISLEEIKNETVDLEKIPIEEVFEQLKCSREGLSSEEGANRLQIFGPNKLEEKKESKILKFLGFMWNPLSWVMEAAAIMAIALANGDGKPPDWQDFVGIVCLLVINSTISFIEENNAGNAAAALMAGLAPKTKVLRDGKWSEQEAAILVPGDIISIKLGDIIPADARLLEGDPLKIDQSALTGESLPVTKNPGDEVFSGSTCKQGEIEAVVIATGVHTFFGKAAHLVDSTNNVGHFQKVLTAIGNFCICSIAIGMLVEIIVMYPIQHRKYRDGIDNLLVLLIGGIPIAMPTVLSVTMAIGSHRLSQQGAITKRMTAIEEMAGMDVLCSDKTGTLTLNKLSVDRNLIEVFVKDVDKEHVVLLAARASRTENQDAIDAAIVGMLADPKEARAGIREVHFLPFNPVDKRTALTYIDSNGNWHRASKGAPEQILTLCNAKEDVKKKVHAIIDKFADRGLRSLGVARQLVPEKSKDGAGTPWQFVGLLPLFDPPRHDSAETIRRALNLGVNVKMITGDQLAIAKETGRRLGMGTNMYPSASLLGQDKDASIAALPVEELIEKADGFAGVFPEHKYEIVRKLQERKHICGMTGDGVNDAPALKKADIGIAVADATDAARSASDIVLTEPGLSVIISAVLTSRAIFQRMKNYTIYAVSITIRIVFGFMLIALIWKFDFSPFMVLIIAILNDGTIMTISKDRVKPSPLPDSWKLKEIFATGIVLGGYLALMTVVFFWIMHDTDFFSEKFGVRSLRKRDDQMMGALYLQVSIVSQALIFVTRSRSWSYFERPGLLLVTAFIIAQLVATVIAVYANWGFARIQGIGWGWAGVIWLYSVVFYIPLDLMKFAIRYILSGKAWLNLLENKTAFTTKKDYGKEEREAQWALAQRTLHGLQPPEASNLFNDKSSYRELSEIAEQAKRRAEVARLRELHTLKGHVESVVKLKGLDIDTIQQHYTV

>GaAHA14

MVTVAPRFRVSLNHHERGARQTVAGSREFQPGGDRFGEFNERLPLEEVFEQLRTSRAGLTSEDAEVRVHIFGQNKLEEKPENKFLKFLSFMWNPLSWVMEAAAVMAIVLANGGGEGPDWQDFVGIICLLIINSTISFIEENNAGNAAAALMARLAPKTKVLRDGQWQERDAAILVPGDIISIKLGDIIPADARLLEGDPLKIDQATLTGESLPVTKRTGDEVFSGSTCKHGEIEAVVIATGVHSFFGKAAHLVDSTEVVGHFQQVLTSIGNFCICSIAVGMVLEIIVMFPIQHRSYRDGINNLLVLLIGGIPIAMPTVLSVTLAIGSHRLSQQGAITKRMTAIEEMAGMDVLCSDKTGTLTLNRLTVDRNLVEVFSKNMDKDLIVLLAARASRLENQDAIDAAIINMLADPKEARANIKEVHFLPFNPVDKRTAITYIDSDGNWYRASKGAPEQLSGATNLSSYSSSSSTVYQILNLCLEKDLIAGRVHAIIDKFAERGLRSLGVAFQEIPERTKESPGGPWTFCGLLPLFDPPRHDSAETIRRALNLGVDVKMITGDQLAIAKETGRRLGMGTNMYPSSSLLGREKDENEALPVDELIEKADGFAGVFPEHKYEIVKILQEKKHVVGMTGDGVNDAPALKKADIGIAVADATDAARSAADIVLTEPGLSVIISAVLTSRAIFQRMKNYTIYAVSITIRIVLGFVLLALIWEYDFPPFMVLIIAILNDGTIMTISKDRVRPSPTPDSWKLNEIFATGVVIGTYLALVTVLFYWIVIDTDFFETHFNVRSISDDTEQISSAVYLQVSIISQALIFVTRSRSWSFVERPGVLLMCAFVVAQLVATLIAVYAHISFADISGIGWGWAGVIWLYSLVFYVPLDIIKFTVRYALSGEAWNLLFDRKVSVLFLVSYLIGFKSFLEEAFHGIPVQQTAFTSKKDYGKDDRAAQWILSQRSLQGLMAADLDFNGRRSRSSLIADQARRRAEIARLGELHTLRGHVESVMRLKNLDLNAIKSAHTV

>GaAHA2

MGGEKGISLEEIKNESVDLERIPIEEVFEQLKCSREGLSTEEGNNRLQVFGPNKLEEKKESKVLKFLGFMWNPLSWVMEAAAIMAIALANGDGRPPDWQDFVGIIVLLVINSTISFIEENNAGNAAAALMANLAPKTKVLRDGRWSEQEAAILVPGDIITIKLGDIVPADARLLEGDPLKIDQSALTGESLPVTKNPSDEVFSGSTCKQGEIEAVVIATGVHTFFGKAAHLVDSTNQVGHFQKVLTAIGNFCICSIAVGIIVELIVMYPIQHRKYRDGIDNMLVLLIGGIPIAMPTVLSVTMAIGSHRLSQQGAITKRMTAIEEMAGMDVLCSDKTGTLTLNKLTVDRNLIEVFTKGVEKEHVILYAARASRTENQDAIDAAIVGMLADPKEARAGVREIHFLPFNPVDKRTALTYIDSDGNWHRASKGAPEQIIDLCKCKDDVRKKVHSVIDKFAERGLRSLAVARQEIPEKTKESPGSPWQFIGLLPLFDPPRHDSAETIRRALNLGVNVKMITGDQLAIAKETGRRLGMGTNMYPSSSLLGQDKDASIAALPIDELIEKADGFAGVFPEHKYEIVKRLQERKHICGMTGDGVNDAPALKKADIGIAVADATDAARSASDIVLTEPGLSVIISAVLTSRAIFQRMKNYTIYAVSITIRIVFGFMFIALIWKFDFAPFMVLIIAILNDGTIMTISKDRVKPSPQPDSWKLKEIFSTGIVLGGYLALMTVIFFWAMKDTNFFSNTFNVRSLRHNNIDGEKEMMAALYLQVSIVSQALIFVTRSRSWSYFERPGLLLVSAFLVAQLVATLIAVYADWGFARIKGMGWGWAGVIWLYSVVTYIPLDLIKFAIRYVLSGKAWDNLLENKTAFTTKKDYGKEEREAQWAAAQRTLHGLQPPETTSIFNERSSYRELSEIAEQAKRRAEVARLRELNTLKGHVESVVKLKGLDIDNIQQHYTV

>GaAHA3

MDRLSGSHSGYTFCYYRWRATIKAPPPPFHMAGNKGISLDEIKNESVDLERIPIEEVFEQLKCTRAGLTTQEGNNRLQVFGPNKLEEKKESKFLKFLGFMWNPLSWVMEAAAVMAIALANGGGRPPDWQDFVGIIVLLVINSTISFIEENNAGNAAAALMANLAPKTKVLRDGRWSEQEAAILVPGDIITIKLGDIVPADARLLEGDPLKIDQSALTGESLPVTKNPSDEVFSGSTCKQGEIEAVVIATGVHTFFGKAAHLVDSTNQVGHFQQVLTAIGNFCICSIAVGIVIELIVMYPIQRRKYRDGIDNLLVLLIGGIPIAMPTVLSVTMAIGSHRLSQQGAITKRMTAIEEMAGMDVLCSDKTGTLTLNKLTVDRNLIEVFAKGVEKEQVLLYAARASRTENQDAIDAAIVGMLADPKEARAGIREVHFLPFNPVDKRTALTYIDSDGKWHRASKGAPEQIITLCNCKADVRNKVHAVIDKFAERGLRSLAVARQEVPEKTKESPGAPWQLVGLLPLFDPPRHDSAETITRALNLGVNVKMITGDQLAIAKETGRRLGMGTNMYPSSSLLGQDKDSSVSALPVDELIEKADGFAGVFPEHKYEIVKRLQERKHICGMTGDGVNDAPALKKADIGIAVADATDAARSASDIVLTEPGLSVIISAVLTSRAIFQRMKNYTIYAVSITIRIVFGFLFIALIWKFDFAPFMVLIIAILNDGTIMTISKDRVKPSPQPDSWKLKEIFSTGIVLGGYLALMTVLFFWAMKDTDFFSDKFHVRSLRDRPEQMMAALYLQVSIVSQALIFVTRSRSWSFVERPGLLLVTAFIAAQLVATLIAVYANWRFAKIQGMGWGWAAVIWLYSLVTYIPLDLIKFAIRYVLSGKAWDNLLENKTAFTTKKDYGKEEREAQWAAAQRTLHGLQPPETSNVFNERSSYRELSEIAEQAKRRAEVARLRELNTLKGHVESVVKLKGLDIDTIQQHYTV

>GaECA2

MEERAFPAWSWSVEHCLKEYDVRLDKGLSSYKVEKRREKYGWNELAKEKGKPLWRLVLEQFDDMLVKILLVAAFISFLLAYMHGSESEESGFEAYVEPFVIVLILVLNAIVGVWQETNAEKALEALKEMQCESGKVLRDGFLVPDLPARELVPGDIVELQVGDKVPADMRIAALKTSTLRLEQSALTGEAMPVLKGTSPIFQKECELQAKENMVFAGTTVVNGCCVCIVVCTGMNTEIGKIQKQIHEASLEESDTPLKKKLDEFGSRLTTAIGIVCLIVWLINYKNFLSYDMVEGWPANVRFSFEKCTYYFKIAVALAVAAIPEGLPAVITTSLALGTRKMAQKNAIVRKLPSVETLGCTTVICSDKTGTLTTNQMSVAEFFTLGGKTTTSRIFHVKGTTYDPKDGGIVDWTCYNMDANLQVMAEICAVCNDAGIFCDGRLFRATGLPTEAALKVLAEKMGVPDAKMRNKIRDSELVANYLIDRSTVKLGCCEWWIKRSKRVATLEFDRVRKSSSIIVREAAGQNRLLAKGAVESLLERSTHVQLADGSLAPMDGPCRQLLLSRQTEMSSKGLRCLGLAYKEDLGEFSDYYSENHPAHKKLLDPACYCSIESDLVFVGAVGLRDPPRDEVHKAIEDCKRAGIRVMVITGDNKSTAEAICREIKLFSDGEDLRGKSFTGKDFMALSPSQQIETLSKPGGKVFSRAEPRHKQEIVRMLKEMGEIVAMTGDGVNDAPALKLADIGIAMGITGTEVAKEASDMVLADDNFSTIVSAVAEGRSIYNNMKAFIRQYMISSNVGEVISIFLTAALGLPECMIPVQLLWVNLVTDGPPATALGFNPPDIGIMRKPPRRSDDALINSWVLFRYLIIGSYVGIATVGIFILWYTRASFMGINLVSDGHTLVELSQLRNWGECSTWSNFTVAPYMVGGGQLISFSNPCDYFTAGKVKAMTMSLSVLVAIEMFNSLNALSEDSSLLTLPPWRNPWLLAAMSVSFGLHCLILYVPFFADIFAVAPLSLNEWFLVILVSVPVILIDEILKFVGRGQRYRVKEKTA

>GaECA3

MEKKPFPAWSWSVEQCLKEYNVKLDKGLSSYQVEKQREKYGWNELSKEKGKPLLRLVLEQFDDILVKILLVAAYISFILAYMHGSESEESGFEAYVEPFVIVLILVLNAIVGVWQETNAEKALDALKEMQCESGKVVRDGYIVPDLPARELVPGDIVELQVGDKVPADMRIAALKTSTLRLEQSALTGEAMPVLKGTSPIFPEECELQAKENMVFAGTTVVNGSCVCIVVCTGMNTEIGKIQKQIHEASLEESDTPLKKKLDEFGSRLTTAIGLVCLIVWLINCKNFLSWDMVDGWPANLRFSFEKCTYYFKIAVALAVAAIPEGLPAVITTSLALGTKKMAQKNAIVRKLPSVETLGCTTVICSDKTGTLTTNQMSVAEFFTLGGKTTTSRIFHVEGTTYDPKDGGIVDWTCYNMDANLQVMAEICAVCNDAGIFCDGRLFRATGLPTEPALKVLVEKMGVPDAKMRNEIHDSQLAANYLIDRSTIKLGCWEWWTKRSKRLATLELDTLRKSMSVIVREPTGHNRLLVKGAVESLVERSTHVQLADGSLVPMDESCSQLLLSRNSEMSSKGLRCLGLAYKDDLGEFSDYYSENNPAHKKLLDPASYSSIESDLVFVGVVGLRDPPRDEVDKAIEDCKGAGIRVIVITGDNKSTAEAICHEIKLFSDGEDVRGKSFTGKEFMALSPSQQIETLSKPGGKVFSRAEPRHKQEIVRMLKEMGEIVAMTGDGVNDAPALKLADIGIAMGITGTEVAKEASDMVLADDNFSTIVSAVAEGRSIYNNMKAFIRYMISSNVGEVISIFLTAALGLPECMIPVQLLWVNLVTDGPPATALGFNPPDVGIMRKPPRKSDDTLIDSWVLFRYLTIGSYVGVATVGIFILWYTQASFMGINLVSDGHTLIELSQLRNWGECSTWSNFSVAPYTVGDGHLITFSNPCDYFTIGKVKAMTLSLSVLVAIEMFNSLNALSEDSSLLTLPPGRNPWLLVAMSVSFGLHCLVLYVPFLANMFGVVPLSLNEWFLVILVSIPVILIDETLKFFGRSRRHRVKEKTA

>GaAHA8

MVDIDDTLQEIKNENVDLERIPVEEVFLQLKCTKEGLTTEEGLKRLQIFGPNKLEEKSESKVLKFLGFMWNPLSWVMEIAAIMAIALANGGGKPPDWQDFVGITVLLIINSTISFIEENNAGNAAAALMAGLAPKTKVLRDGKWCEQEAAILVPGDIISIKLGDIIPADARLLEGDALKVDQSALTGESLPVNKNPGDGVYSGSTVKQGELEAVVIATGVHTFFGKAAHLVDSTNNVGHFQKVLTAIGNFCICSIAVGMLVEIVVMYPIQRRKYRDGIDNLLVLLIGGIPIAMPTVLSVTMAIGSHRLSQQGAITKRMTAIEEMAGMDVLCSDKTGTLTLNKLTVDKSMVEVFTKDVDKEMLLLLAARASRVENQDAIDACIVGMLGDPKEAREGITEVHFFPFNPVDKRTAMTYIESDGSWHRVSKGAPEQIISLCDLRDDVKKKAHDIIDKFADRGLRSLGVARQTVPEKTKDSPGSPWEFVGLLPLFDPPRHDSAETIRRALHLGVNVKMITGDQLAIGKETGRRLGMGTNMYPSSALLGHNKDEKVETIDVDELIEKADGFAGVFPEHKYEIVKRLQERNHICGMTGDGVNDAPALKKADIGIAVDDATDAARSASDIVLTEPGLSVIVSAVLTSRAIFQRMKNYTIYAVSITIRIVLGFMLLALIWKFDFSPFMVLIIAILNDGTIMTISKDRVKPSPMPDSWKLKEIFATGIVLGTYLACMTVVFFWAANDSDFFSDKFGVKSIRYSQDELTAAVYLQVSIVSQALIFVTRSRSWSFIERPGLLLVVAFILAQLVATVLAVYANWGFARIKGIGWPWAGVIWLYSIVFYIPLDVLKFLIRYALSGKAWDNLLQNKTAFTTKKDYGREEREAQWATAQRTLHGLQAPGAEEILNEKSSYRELSEIAEQAKKRAEVARLRELHTLKGHVDSVVKLKGLDIETINQNYTV

>GaAHA12

MENKDETLDAVLKEAVDLENVPLEEVFQTLRCNRDGLTTEAAEQRLSIFGYNKLEEKQESKILKFLGFMWNPLSWVMEAAAIMAIALANGGGKPPDWQDFVGIITLLIINSTISFIEENNAGNAAAALMARLAPKAKVFRDGKWSEEEASILVPGDIISIKLGDIIPADARLLDGDPLKIDQSSLTGESLPVTKGPGDSIYSGSTCKQGEIEAVVIATGVHTFFGKAAHLVDSTNQQGHFQKVLTAIGNFCICSIAVGMITEIIVMYPIQDREYRPGIDNLLVLLIGGIPIAMPTVLSVTMAIGSHRLSLQGAITKRMTAIEEMAGMDVLCSDKTGTLTLNKLSVDKNLIEIFAKGVDPDTVVLMAARASRLENQDAIDAAIVGMLADPKEARAGIQEVHFLPFNPTDKRTALTYIDNQGRMHRVSKGAPEQILNLAHNKSELERRVHAVIDKFAERGLRSLAVAYQEVPDGRKESSGGPWQFIGLMPLFDPPRHDSADTIRRALNLGVNVKMITGDQLAIAKETGRRLGMGTNMYPSSSLLGQNKEESIAALPVDELIEKADGFAGVFPEHKYEIVKRLQARKHICGMTGDGVNDAPALKKADIGIAVADATDAARSASDIVLTEPGLSVIISAVLTSRAIFQRMKNYTIYAVSITIRIVLGFMLLALIWKFDFPPFMVLIIAILNDGTIMTISKDRVKPSPLPDSWKLAEIFATGIILGGYLAMMTVIFFWAAYKTDFFPRTFGVKSLQKTDRKDIKMLASAVYLQVSIISQALIFVTRARSWSFLERPGLLLVLAFVVAQLIATLIAVYANWGFAAIEGIGWGWAGVIWLYNLIFYIPLDFIKFFIRYALSGKAWDLVIEQRIAFTRKKDFGKEERELKWAHAQRTLHGLQPPDTKMFGDRTSYNELNQMAEEAKRRAEIARLRELTTLKGHVESVVRLKGLDIDTIQQAYTV

>GaECA4

MGKGGENYGKREFVNSKPTDPDVFPAWAKDIRECEKHYDVSRKSGLSTAEVENRRKIYGNNELEKHEGQSIWSLIMEQFNDTLVRILLLAAVVSFVLAWYDGDEGGEMEITAFVEPLVIFLILIVNAIVGVWQENNAEKALEALKEIQSEQATVIRDGIKIPSLPAKELVPGDIVELKVGDKVPADMRVLELVSSTLRVEQGSLTGESEAVNKTNKSVSEDADIQGKRSMVFAGTTVVNGNCFCLVTQIGMETEIGKVHTQIHVASQSEEDTPLKKKLNEFGEVLTMIIGVICIFVWLINVKYFLSWEYIDGWPRNFKFSFEKCTYYFEIAVALAVAAIPEGLPAVITTCLALGTRKMAQKNALVRKLPSVETLGCTTVICSDKTGTLTTNQMAVAKLVAMGGHASSLRSFRVDGTTYNPSDGKIHDWPSGGMDANLETIAKISAICNDAGVTHSDNKYVAHGMPTEAAIKVLVEKMGLPKGLYSGGAAGDDVLRCCQWWNEYEHRIATLEFDRDRKSMGVIVKSKSGRSSLLVKGAVENLLERSSKMQLLDGSVVPLDQNSRILVSNALQDMSSGALRCLGFAYKDELPEFETYDGSDDHPAHALLLDPSNYSSIESNLTFVGLVGLRDPPRQEVHQAIEDCKAAGIRVMVITGDNKNTAEAICHEIGVFGPMEDISSKSLTGKEFMALSDKKAHLRQSGGLLFSRAEPRHKQEIVRLLKEDGEVVAMTGDGVNDAPALKLADIGIAMGIAGTEVAKEASDMVLADDNFSTIVSAVGEGRSIYDNMKAFIRYMISSNIGEVASIFLTAALGIPEGLIPVQLLWVNLVTDGPPATALGFNPPDKDIMKKPPRRSDDSLITAWILFRYLVIGLYVGVATVGVFIIWYTHRSFLGIDLSGDGHTLVTYPQLANWAQCSSWKNFTVSPFTAGNQVFDFENNPCDYFQGGKVKAMTLSLSVLVAIEMFNSLNALSEDGSLLTMPPWVNPWLLLAMSVSFGLHFLILYVPFLAQVFGIVPLSFNEWLLVLAVAFPVILIDEVLKFVGRLGRRMRSSSQRPLKPKTE

>GaECA5

MGRGGENYGKRENAGAASFKQETFPAWARDVKQCEEKYQVNRELGLSSAEVEKRRQIYGLNELLKHKPTSIFQLLLEQFNDTLVRILLAAAIISFVLAWYDGEGGGEKEITAFVEPLVIFLILIVNAFVGIWQESSAEKALEALKEIQSEHADVIRDGKKVSSLPAKELVPGDIVELRVGDKVPADMRVLNMISSTVRVEQGSLTGESEAVSKTVKVVPENSDIQGKKCMVFAGTTMVNGNCICLVTEIGMNTEIGKVHSQIHEASQSDTDTPLKKKLNEFGEVLTMIIGMICILVWLINVKYFLSWEYVDGWPTNFKFSFEKCTYYFEIAVALAVAAIPEGLPAVITTCLALGTRKMAQKNALVRKLPSVETLGCTTVICSDKTGTLTTNQMAVSKLVAIGSRSGTLRSFDVEGTTYDPFDGKILGWPVDGMDSNLEMIAKISAVCNDAGVEQSGRHYVAIGMPTEAALKVLVEKMELPEKYASSSVPGDPRRCCHVWNKMVQRIATLEFDRDRKSMGVIINSSSGNKSLLVKGAVENLLERSSFIQLLDGSTVELDKYSKDLILQVLREMSTDALRCLGFAYKEELPEFATYNGDEDHPAHQLLLNPSNYSSIESNLIFVGLVGLRDPPRKEVRQAIEDCKAAGIRVMVITGDNKNTAEAICREIGVFGYREDITSRSLTGKEFMDHPDQRNHLRQNGGLLFSRAEPRHKQEIVRLLKQDGEVVAMTGDGVNDAPALKLADIGVAMGITGTEVAKEASDMVLADDNFSTIVAAVGEGRSIYDNMKAFIRYMISSNIGEVASIFLTAALGIPEGMIPVQLLWVNLVTDGPPATALGFNPQDTDIMKKPPRRSNDSLITAWILFRYLVIGSYVGLATVGVFIIWYTHNTFMGIDLSGDGHSLVTYSQLSNWDKCPSWVNFTASPFTAGPQVFNFDTNPCDYFRSGKIKASTLSLSVLVSIEMFNSLNALSEDGSLLTMPPWVNPWLLLAMSISFGLHFLILYVPFLAQVFGIVPLSLNEWLLVLAVAFPVILIDEVLKCIGRCTTGPRYSPATKSIKHKAE

>GaHMA4

MVANKNLQKSYFDVLGLCCSSEVPLIENILKSLEGVKEVSVIVPTRTVIVLHDNLLLSQLQIVKALNQARLEANVRAHGEIKYQKKWPSPFAVVCGLLLLLSFLKYVYHPLQWLAVGAVVIGIYPVLFKGFAAITHFRIDINILILIAVIGSVAMKDYTEAGTIVFLFTTAEWLESRASHKASAVMSSLMRITPQKAVIAENGEEVDADEVKLNTLLAVKAGEVIPIDGIVVDGRCEVDEKSLTGESLPVTKEKDSTVWAGTINLNGYISVKTTAVAEDCVVAKMAKLVEEAQNSKSNTQRFIDKCAQFYTPVIIVVSAAIAVIPAALRVQNLHHWFHLALVVLVSACPCALILSTPVASFCALTKAATSGLLVKGGDYLETLSKIRITAFDKTGTLTRGEFIVTDFQSLSQDISLDTLLYWVSSIESKSSHPMAAALVDYGRSHSIEPNPETVEDYQNFPGEGIYGRVDGRDIYIGSLKISVRAHGTVPTLEGNMMKGKTIGFVYSGATPAGIFSLSDACRTGVPEAVEELKSMRIKVAMLTGDNQSAAIHVQEQLGNRLDVVHAELLPEDKARIIKEFKKEGATAMIGDGVNDAPALATADIGISMGISGSALATETGHVVLMSNDIRKIPKAIKLARKAHRKVIENVILSISTKTAILALAFAGHPLVWAAVLADVGTCLLVICNSMLLLRGKHKDGRKCCKSSAAAHTNKHGCKASHCDSSHKHQDASLDKKVQKACEPPTCSSERCASRCHSGLFKTDSPSNSRGSDKCEDLGRTHDGSVIREAKYCDQGSCHLVNHKIEAQNLPRKCCSGRGSLDLGKEANALHGAKQCHQGHLHQYSSSTPEEEQRETKNDHCHSTHCRENHVEIHGNNLTAFGNLVEHRCLESLNQRAHLDSHEPTHTAIDITMNPDEVHGCANVEKRELGGCCKSYMKECCGKHKHGRFRPGLTDIITE

>GaECA6

MGRGGEDYGKREKVSAASSKVENFPAWAKDVKQCEENFQTNRELGLSSAEVEKRREIYGWNELEKHEGTSIFQLILEQFNDTLVRILLLAAIISFVLAWLDGDEGGEKEITAFVEPLVIFLILIVNAIVGIWQESNAEKALEALKEIQSEQANVVRDGKKVSNLPAKELVPGDIVELRVGDKVPADMRVLTLISSTVRVEQGSLTGESEAVSKTAKVVPENTDIQGKKCMVFAGTTVVNGNCICMVTQIGMNTEIGKVHSQIHEASQSDDDTPLKKKLNEFGEALTMIIGVVCALVWLINVKYFLSWEYVDGWASNFKFSFEKCTYYFEIAVALAVAAIPEGLPAVITTCLALGTRKMAQKNALVRKLPSVETLGCTTVICSDKTGTLTTNQMAVSKLIAIGSRPAILRAFDVEGTTYNPFDGKIRGWAAGEMDANLQMIAKICAVCNDAGVEQSGSHYVATGMPTEAALKVLVEKMGLPEENGSSSDHWDHQRCCQAWNKMEQRIATLEFDRDRKSMGVIVNSSTGQKALLVKGAVENLLERSSFMQLCDGSIIELDQYSKDLILQSLHEMSTDALRCLGFAYKEEPFEFTTYNGDEDHPAHQLLLNPSNYSSIESKLIFSGLVGLRHLDKPMKDPPRKEVRQAIEDCKAAGIRVMVITGDNKNTAEAICREIGVFESSEDISSRSLTGKDFMDHPNQKNHLRQSGGLLFSRAEPRHKQEIVRLLKEDGEVVAMTGDGVNDAPALKLADIGVAMGIAGTEVAKEASDMVLADDNFSTIVAAVGEGRSIYNNMKAFIRYMISSNIGEVASIFLTSALGIPEGMIPVQLLWVNLVTDGPPATALGFNPPDKDIMKKPPRRSDDSLITAWILFRYLVIGLYVGIATVGVFIIWYTHHSFLGIDLSGDGHSLVTYSRLANWAKCDSWEGFSVSPFTAGSQVFKFDSDPCDYFQSGKIKASTLSLSVLVAIEMFNSLNALSEDGSLLTMPPWVNPWLLLAMSVSFGLHFLILYVPFLARVFGIVPLSTNEWLLVIAVAFPVILIDEVLKFIGRRTTRLHYPAVPKSSKQKAE

>GaAHA7

MASNGDISLEQIKNETVDLERIPVEEVFQQLKCTREGLTSEEGEKRLQIFGPNKLEEKEESKFLKFLGFMWNPLSWVMEAAAIMAIVLANGGGKPPDWPDFIGIVSLLFINSTISFIEKNNAGNAAAALMAGLAPKTKVLRDGKWGEQEATILVPGDVINVKLGDIIPADARLLEGDALKFDQSALTGESLPVTKNPGDEVFSGSTCKQGEIEAVVIATGVHTFFRKAAHLVDNTNNVGHFQKVLAAIGNFCICSIAVGMLIEILVMYPIQHRRYRDGIDNLLVLLIGGIPIAMPTILSVTMAIGSHSLSQQGAITKRMTAIEEMAGMDVLCSDKTGTLTLNKLTVDKSLIEVFINDMDADTLVLIAARASRVENQDAIDASIVGMLGDFKEARAGITEVHFLPFNPVDKRTAITYIDRNGEWHRCSKGAPEQVIELCELTGGLRQKAHHVIDSFADRGLRSLGVARQTIPEKTKECAGGPWEFVGLLPLFDPPRHDSAETIRQTLDLGVNVKMITGDQLAIGKETGRRLGMGTNMYPSSSLLGQCKDEAIATIPVDELIETADGFIGVFPEHKYEIVKRLQELKHICGMTGDGVNDAPALKKADIGIAVADATDAARSASDIVLTEPGLSVIVSAVLTSRAIFQRMKNYTIYAVSITIRIVMGFMLIALIWEFDFSPFMVLIIAILNDGTIMTISKDRVKPSPTPDSWKLKEIFITGVVLGAYMAIVSVIFFWLVHDTLFFTEKFGVKPINENANELTSALYLQVSIISQALIFVTRSRSWSFVELPGLLLLGAFLAAQLVATCIAVYANWGFARIEGIGWEWTGVIWPHSQQRRIMNYGKEAREAQWAANQRTLHGLSPPETSWTNEKDHHELSEIAEQAKKRAEIARLRKLHTLKGHVESVVKLKGIDIDTIQQHYSV

>GaHMA2

MESALSVTVPTLALFSIPRALNRHFYYNNCSLIARCIRSRLFPQGRGVTLLASRSYSSPLRSLCAAPVPQRLHRRLECVASSAAYFGAAGGGGVYGGGDGSGSGSGGGGGDGGEGTGDGDLKAKLGAGAVDEPSALSPDIIILDVGGMTCGGCAASVKRILENQPQVSSASVNLTTETAIVWPVSEAKVVPNWQKELGEALARQLTSCGFNSNLRDSGRDNFFKVFERKMDEKRSRLRESGRELAVSWALCAVCLIGHVAHFLGAKASWMHAFHSTGFHLSLSLFTLLGPGRQLIFEGVKNLFKGAPNMNTLVGLGALSSFAVSSLAVLIPKLGWRAFFEEPVMLIAFVLLGRNLEQRAKIKATSDMTGLLSSLPSQARLMVDDSIVEVPCSSLSVGDQIVVLPGDRVPADGIVRAGRSTIDESSFTGEPMPVTKEPGSQVAAGSINLNGTLTIEVRRPGGETAMGDIVRLVEEAQSREAPVQRLADKVAGHFTYGVMALSAATFMFWNLFGARIIPASIYQGSTVSLALQLSCSVLVVACPCALGLATPTAMLVGTSLGATRGLLLRGGNILEKFSMVNVIIFDKTGTLTIGRPVVTKVVTPSGMDHSDSRQHFDGSWSEDDVLKLAAAVESNTIHPVGKAIVEAAQAVKSPNIKVVDGTFVEEPGSGAVAVINDKTVSVGTLEWVQRHGVGDSLLLETDEELRNKSVVYVGVNNKLAGLIYFEDQIREDARHVVDSLYRQGISVYMLSGDKRSTAEYVASIVGIPKDKVLSQVKPDEKRKFVSELQENQNVVAMVGDGINDAAALASAHIGVAMGGGVGAASEVSSIVLMGNRLSQLLDALALSQLTMKTVKQNLWWAFAYNIVGIPIAAGTLLPLTGTMLTPSIAGALMGLSSIGVVTNSLLLRFRFSLQQQQAYRSSLQPPPPPPPPPYAAVDINNDLAMDHSKAKLKKPDSIT

>GaHMA7

MATKLLALACIRKESYGDLSPRPHYPSMPKYPKGVTAQETSLQGSEAKAMFSVMGMTCSACAGSVEKAVKRLPGIKEAVVDVLNNKAQVMFYPSFVNEESIREAIEDAGFQAALIQDETDDKSVQVCRIRINGMTCTSCSPTLENALQAVPGVQKVQVALATEEAQIHHDPKIITYNQLMEKIEETGFGAVLVSTGEDMSKINLRIDGVRTVNSMRMLENSLQALPGVQAVQTSPELKKIAVSYKPDMTGPRNFIKVIDSTGSSRRFKATIYPEGEGAGRESHRKEEIKQYFRSFLWSLIFTTPVFLTSMIFMYIPGIKHGLDTKVVNMLTIGEVIRWVLSTPVQFIIGRRFYTGSYKALRHGSANMDVLIALGTNAAYFYSVYTVIRAASSPDFEGTDFFETSAMLISFILLGKYLEVLAKGKTSEAIAKLMNLAPETAILLSLDEEGNVISEEEIDSRLIQKNDIIKIIPGAKVASDGFVLWGQSHINESMITGEARPVAKRKGDTVIGGTVNENGVLHIKATKVGSESALAQIVRLVESAQMAKAPVQKFADRISKYFVPLVIMLSFSTWLAWFLAGKLHGYPESWIPSSMDSFELALQFGISVMVIACPCALGLATPTAVMVGTGVGASLGVLIKGGQALEGAHKVNCIVFDKTGTLTVGKPVVVNTRLLKNVVLHEFYELVAATEANSEHPLAKAIIEYAKKFREDEENPAWPEARDFVSKTGHGVKAIVRNKEVIVGNKSLMLENNIVIPVDAQDMLTETESMAQTGILVSIDSEVTGVLAISDPVKPGAQEVISILKSMNVRSIMVTGDNWGTASSIASQIGIETVVAEAKPEQKAEKVKELQAEGYAVAMVGDGINDSPALVAADVGMAIGAGTDIAIEAADIVLMKSNLEDVITAIHLSRKTFSRIRLNYIWALGYNILGIPIAAGALFPSTGFRLPPWIAGAAMAASSVSVVCCSLLLKNYERPKKLENLEIGGIQIE

>GaACA13

MSSSGGNQMYDCGTLLFKVTTSGFTTAQKRWRIAYASIYSVRVMLSLAKEIISKRGIEQPSIISDLHPYVALDVEPSSSPHWGEKFSSSSFAPKIDRKRLVETLVETVKEKDLVSLHQLGGVEGIAAALGTNPEKGIRDDDRDVVKRQEMFGTNTYHKPPPKGLLYFVLDAFKDTTILILLVCAALSLGFGIKEHGAAEGWYEGGSIFVAVFLVIVVSALSNFRQETQFDKLSKISNNIKVEVVRGGRRRQVSIFDLVVGDVVFLKIGDQIPADGLFLDGYSLQVDESSMTGESDHMEVDATRNPFLFSGSKVADGYGQMLVASVGMDTTWGEMMSSITSDKNERTPLQERLDRLTSSIGKVGLAVAFLVLVVLLIRYFTGNTEDDNGNTEYIGSKTSVDDILNAVVRIVSAAVTIVVVAIPEGLPLAVTLTLAYSMKRMMADQAMVRKLSACETMGSATIICTDKTGTLTLNQMKVTQFWLGQESIKEDHSNIIDHAVLELFYQGVGLNTTGSVCKPVSGSLPEFCGSPTEKAILSWAVLGLDLDMEKLKQKYSILHVETFNSEKKRSGVSVRRKTDETLHVHWKGAAEIIVAMCSDYYESNGGIRSMDEDQRSRIETIIQSMAASSLRCIAFAHKQVSQKEMECVDDSEKTHQRIKEDGLTLLGIVGLKDPCRPGVKKAVEACKSAGVDIKMITGDNIFTAKAIAAECGILGADYNEESGQAIEGIEFRNYTPEERMEKIGKIKVMARSSPFDKLLMVQCLKQKGDVVAATGDGTNDALALKEADIGLSMGIQGTEVAKESSDIVILDDNFSSVATVLRWGRCVYNNIQKFIQFQLTVNVAALVINFIAAVSAGEVPLTAVQLLWVNLIMDTLGALALATDRPTKELMKKPPVGRTEPLITNVMWRNLLAQAVYQIAILLILQFRGESMFNVPKRVKDTLIFNTFVLCQVFNEFNARKLEKQNVFQGILQNRLFLGIVGITIILQVVMVEFLKKFADTERLELWQWGVCILFAAFSWPIAWVVKLIPVSDKPFFSYLKRSKSSSQLNKSSTTRNLQSAGMELEVQ

>GaACA14

MSSSGGNQMYDCGTLLFKVTTSGFTTAQKRWRIAYASIYSVRVMLSLAKEIISKRGIEQPSIISDLHPYVALDVEPSSSPHWGEKFSSSSFAPKIDRKRLVETVKEKDLVSLHQLGGVEGIAAALGTNPEKGIRDDDRDVVKRQEMFGTNTYHKPPPKGLLYFVLDAFKDTTILILLVCAALSLGFGIKEHGAAEGWYEGGSIFVAVFLVIVVSALSNFRQETQFDKLSKISNNIKVEVVRGGRRRQVSIFDLVVGDVVFLKIGDQIPADGLFLDGYSLQVDESSMTGESDHMEVDATRNPFLFSGSKVADGYGQMLVASVGMDTTWGEMMSSITSDKNERTPLQERLDRLTSSIGKVGLAVAFLVLVVLLIRYFTGNTEDDNGNTEYIGSKTSVDDILNAVVRIVSAAVTIVVVAIPEGLPLAVTLTLAYSMKRMMADQAMVRKLSACETMGSATIICTDKTGTLTLNQMKVTQFWLGQESIKEDHSNIIDHAVLELFYQGVGLNTTGSVCKPVSGSLPEFCGSPTEKAILSWAVLGLDLDMEKLKQKYSILHVETFNSEKKRSGVSVRRKTDETLHVHWKGAAEIIVAMCSDYYESNGGIRSMDEDQRSRIETIIQSMAASSLRCIAFAHKQVSQKEMECVDDSEKTHQRIKEDGLTLLGIVGLKDPCRPGVKKAVEACKSAGVDIKMITGDNIFTAKAIAAECGILGADYNEESGQAIEGIEFRNYTPEERMEKIGKIKVMARSSPFDKLLMVQCLKQKGDVVAATGDGTNDALALKEADIGLSMGIQGTEVAKESSDIVILDDNFSSVATVLRWGRCVYNNIQKFIQFQLTVNVAALVINFIAAVSAGEVPLTAVQLLWVNLIMDTLGALALATDRPTKELMKKPPVGRTEPLITNVMWRNLLAQAVYQIAILLILQFRGESMFNVPKRVKDTLIFNTFVLCQVFNEFNARKLEKQNVFQGILQNRLFLGIVGITIILQVVMVEFLKKFADTERLELWQWGVCILFAAFSWPIAWVVKLIPVSDKPFFSYLKRSKSSSQLNKSSTTRNLQSAGMELEVQ

>GaACA12

MSSSDECKLYDCSTSLLTVKAPGGFTVAQRRWRIAYITIYSARVMLSLADKIISQRATQLPSMTSQQYVTEFDHYVALDIEHKINQKRLVKTVKEKDLVSLNHLGGVDGVVDALCTNSEHGIRDDEQEVIKRQEMFGFNKYHKPPPKGLLYFVLEAFKDTTILILLVCATLSLGFGIKEHGAEEGWYEGGSIFVAVFLVIVVSALSNFRQETQFDKLSKISNNIKVEVVRSGRRQQISIFDLVAGDVVFLKIGDQIPADGLFLDGHSLQVDESSMTGESDHVEVDACRNPFLSSGSKVVDGYARMLVASVGMDTAWGEMMSSITSDKNERTPLQARLDKLTSSIGKVGLAVAFLVLAVLLIRYFTGNTKDDNGQTEYRGSQTDVDDILNAVVRIVAAAVTIVVVAIPEGLPLAVTLTLAYSMKRMMADQAMVRKLSACETMGSATIICTDKTGTLTLNQMKVTQFWLGQESVEEDLAKEIAPSVLELFYQGVGLNTTGSVCKPVSGSLPEFSGSPTEKAILSWAVLGLGMDMEKLKQQYIILHVETFNSEKKRSGVSVQRKADEMVDIHWKGAAEMIVAMCSQYYESNGIIRSMSEDGRERIETIIQSMAASSLRCIAFAHKQVLKGETEDGDDQSGKTNRRLKEDGLTLLGIVGLKDPCRPGVKKAVQACQSAGVGVKMITGDNIFTAKAIATECGILGPDYQQGSGEVVEGTEFRNYAPDERMEKVEKIRVMARSSPFDKLLMVQCLKQKGHVVAVTGDGTNDAPALKEADIGLSMGIQGTEVAKESSDIVILDDNFSSVATVLRWGRCVYNNIQKFIQFQLTVNVAALVINFIAAVSAGEVPLTTVQLLWVNLIMDTLGALALATDRPTNELMEKPPVGRTEPLITNIMWRNLLAQALYQIAILLILQFRGESIVNVPETVKDTLIFNTFVLCQVFNEFNARKLEKQNVFEGILKNRLFLGIIGVTIVLQVVMVEFLKKFADTEQLKLWQWGVCILLAAFSWPIAWFVKLIPVSNTPFFSYLKRSRTIFKRPINHQKP

>GaHMA5

MSPGSRDLQLTSQAAGVWRSTYPSSVRADDPDDMEEGTRLLDSYETGDYKLESIEEGSMRRIQVTVTGMTCAACSNSVEAALKSINGVLRASVALLQNRADVVFDPNLVKDEDIKNAIEDAGFEAEILPEPSNVGTKPRGVLVGQFTIGGMTCAACVNSVEGILRDLPGVSRAVVALATSLGEVEYDPTVISKDDIVNAIEDAGFEASLVQSSEQDKIILGVAGVFNELDVQLIEGILSSLKGVRQFRFDRSSGELEVLFDPEVVSSRSLVDGIEGGSKGKFKLHVMNPYARMTTKDEETSIMFQLFTSSLFLSIPVFLIRVVCPHIPLLDAFLLWRCGPFLMGDWLKWALVSVVQFVIGKRFYVAAGRALRNGSTNMDVLVALGTSASYFYSVGALLYGAITGFWSPTYFETSSMLITFVLLGKYLECLAKGKTSDAIKKLVELAPATALLVVKDNGGNIIGEREVDALLIQPGDILKVLPGAKLPADGVVVWGSSYVNESMVTGESVPVSKEVDSPVIGGTINLHGALHIKATKIGSEAVLSQIISLVETAQMSKAPIQKFADFVASIFVPTVVTLSLITLLGWYAGGAAGAYPQQWLPENGNYFVFALMFSISVVVIACPCALGLATPTAVMVATGVGASNGVLIKGGDALERAQKVQYVIFDKTGTLTQGKAKVTTVKVFSEMDRGEFLTLVASAEASSEHPLAKAIVEYARHFHFFDENSLTEDAQYSSKESPISAWLLDVAEFSAVPGRGIQCFIDGKRVLVGNRKLLTESGVSISAHVEQFVVDLEESARTGILAAYDGNVIGVLGVADPLKREAAVVVQGLQKMGVGPVMVTGDNWRTAQAVAREVGIRDVRAEVMPAGKAEVVRSFQKDGSIVAMVGDGINDSPALAAADVGMAIGAGTDIAIEAADYVLMRNNLEDVITAIDLSRKTFSRIRWNYVFAMAYNVVAIPIAAGVLYPSLGIKLPPWAAGACMALSSVSVVCSSLLLRRYKKPRLTTILEITVE

>GaACA2

MEELLKDFEVPPKNSSEAALRRWRKLVTIVRNPRRRFRMIANLEKRSEAEQQKLKIKEKIRVALIVQKAALQFIDAAGPPDYKITDEVRQAKFGIEPDELASIVHGHDIKRLKSHGGVDGIAEKVTVSLDEGVCSENVSTRQRIYGFNRYTEKPPRNFWMFVWDALQDLTLIILMICAVVSIGVGLATEGWPKGMYDGAGILLSIILVVLVTAISDYRQSLQFRDLDREKKKISVQVTRDGRRQQVSIYDLVVGDVVHLGIGDQVPADGLFISGYSVQIDESSLSGETDPVDIYEQKPFLLSGTKVRDGSAKMLVTAVGMRTEWGKLMETLNEGGEDETPLQVKLNGVATIIGKIGLTFAVLTFLVLTVRFLIEKALHNEFTKWSSTDALTLLDYFAIAVTIIVVAVPEGLPLAVTLSLAFAMKQLMDERALVRHLSACETMGSASCICTDKTGTLTTNHMVVNKIWICEKIRNIGGNENKSIDELEIHESVFSILLRSIFLNSSAEVVKDENGKNSILGTPTETALLEFGLLLSADLDAYRRQFKILKVEPFNSDRKKMSVLVALPEGRIQAFCKGAPEIVLRMCEKVVDSSGEVVLLSEERVRDITEAINGFASDALRTLCVAVKDVGETFNENGIPDSGYTLIAVFGIKDPVRPGVKEAVQTCLAAGITVRMVTGDNINTAKAIAKECGILTAEENAIEGPEFSSKSPDEMKDIIPNIQVMARSKPSDKLNFVINLRNMFGEVVAVTGDGTNDAPALRQSDIGLAMGIAGTEVAKENADVIVMDDNFATIVNVAKWGRAVYINIQKFVQFQLTVNVVALIINFVSACISGSAPLTAVQLLWVNMIMDTLGALALATEPPNDALMKRPPVPRGASFITKPMWRNIIGQSIYQLIVLGVLNFDGKQLLKLTGSDATTVLNTVIFNSFVFCQVFNEINSREIEKINILRGMFSSWIFLGVMASTVAFQVVIVEFLGTFASTVPLSWQLWLLCILIGSVSLIVGVIVKCIPVERAAVKPKHHDGYDALPSGPELA

>GaHMA6

MNINVGDNKMELNGRDDLKRPLLEPSDSVCVTIPEPVDKLEKKRTVMFKIGNIKCASCVTSIESVLGEINGVESVSVSPIHGYAAIEYVPKLVNPKIIKETIEDAGFPVKEFSEQQIAVCRLRIKGMACTSCSESLERALKFLDGVKKAVVGLALEEAKVHFDPNVTDSDRIIEAIEDAGFGADLISSGNEANKVHLKLEGVSSVEDMNTIKSYLESAIGVNHVEMDLEEKMATVNYDPDFTGPRSIIEAVQEVAHGSYKASLYIPPRQRETEQHHEINNYRNQFLLSCLFSVPLFIFSMVLPMLPPFGDWLEYKIYNMFTVGLLLRWVLCTPVQFIVGRRFYKGSYHALRLKSANMDVLVAMGTNAAYFYSVYVAIKSLSSDTFKGQDFFETSAMLISFILLGKYLEVLAKGKTSDALAKLTDLAPDSACLLILDDDGNVVSEVAISTQLIQRNDIIKIIPGEKVPVDGIVTDGQSYVNESMITGEAQPIAKKPGDKVIGGTMNENGCLLVNATHVGSETALSQIVQLVEAAQLARAPIQKIADRISRFFVPAIVLTAFITWLGWLIPGVIGIYPKHWIPKGMDKFELALQFGISVLVVACPCALGLATPTAVMVATGKGASLGVLIKGGNALEKAHKVKAIVFDKTGTLTVGKPEVVNVMLFSSVSMEDFCDVAIAAEANSQHPIAKAFLEHARKLRQKIESNRQSNNQHVTEAKDFEVHPGTGVSGKVGDKMVLVGNKRLMQTYNVTVGPEIEGYISEHEQQARTCVLVSIDGKIAGAFAVTDPVKPEANNVILYLRSMGISSIMVTGDNWATATAIAKEVGIEKVIAETDPIGKADRIKDLQMRGLTVAMVGDGINDSPALVAADVGMAIGAGTDVAIEAADIVLIKSNLEDVVTAIDLSRKTISRIWLNYVWALGYNILGVPVAAGILYPFTGVRLPPWLAGACMAASSLSVVCSSLLLQSYRKSWVFQDTKIGHSHCSKST

>GaACA8

MSLRLRKPSEPTMHRQVEPSKSSVRRWRVAVTAISVTRFLVGLTKKVVEKNAELLRSLSFVTIDVEGSGDERVPILDVDPQGLAKMVKDKSFQSLNDQYGGVKQVATLLQTDFKKGIPGDDNDLALRTKVFGANKYQKQPAKSFFSFVLEAFKDTIIIILLVCAVLSLGFGIKQHGLKEGWYDGGSIIVAVVLVVVVSAVSNYRQSKQFEELSHETNDIRVQVVRNGRYQPVSIFELVVGDIVSLKTGDQIPADGLFVEGHSLKVDESSMTGESDHVEVNEKKNPFLLSGTKVTDGHGYMLVTAVGMNTAWGEMMSSIRRDLNEETPLQARLSKLTSYIGNIGLSVAVLVLLVLLIRYFTGHTKAENGRSAFNGSKTKFDDVMNSVVGIIAAAVTIVVVAIPEGLPLAVTLTLAYSMKRMMRDHAMVRKLSACETMGSATIICTDKTGTLTLNEMKVTEFWLGKEPIDNSMSSEIAPNVLQLLSEGVGLNTTGTVYKPKPTSVPEIYGSPTEKAILSWALNDMGLNIDESKQSCEIIHVEAFNSEKKRSGVLIRRSNNKRVLATHWKGAAEMLLAMCSCYYDKKGVSKFMNEDEREHIGMVIESMAAKSLRCIAFATLDVTVTDGNEENHTKLEERGLTWLGLVGLKDPCRPGVKQAVESCQNAGVSIKMITGDNMHTARAIAFECGILNSESSLHNEAVVEGVQFRNYSEEERMQKIETIRVLARSSPFDKLLMVQCLKQKGHVVAVTGDGTNDAPALKEADIGLSMGIQGTEVAKESSDIVILDDNFTSVATVLRWGRCVYNNIQKFIQFQLTVNVAALVINFIAAVSSGDVPLTAVQLLWVNLIMDTLGALALATEQPTNDLMDKRPVGRTEPLITKVMWRNLTAQALYQVAILLILQFKGKSIFGVSEEVKDTLIFNTFVLCQIFNEFNARNMDKKNIFKGIHKNRLFLAIIGITLVLQAIMVEFLQRFANTERLSWEQWGACIGIAALTWPIGWIVKCIPVDKKVQTRSSAAS

>GaACA3

MEDYLRKNFAVEPKRPSEEALRRWRSAVALVKNRSRRFRMVADLAKRAEADRRRKIIQEKIRVALYVQKAALNFIDAGKQAERKLPEDVREAGFHIGADELASIVRSHDMSSFEEHGGVEGLAKKVSVSLTNGVVPTDISFRKNIYGNNKFDEKPARSFWMFVWEALHDLTLIILIVCAVVSIGVGVATEGWPGGLYDGLGIVLCIFLVVFVTAISDYKQSLQFKDLDKEKKNILVQVTREGCRQKISIYDLVVGDIVHLSIGDQVPADGVLISGYSLSIDESSLSGESEPVKVTQERPFLLSGTKVQDGSGKMLVTTVGMRTEWGRLMVTLSEGGVDETPLQVKLNGVATVIGKIGLVFAVLTFLVLAIRFMVTKAQLGEIEKWGMSDVLSLLNFFAVAVTIIVVAVPEGLPLAVTLSLAFAMKKLMSDKALVRHLSACETMGSATCICTDKTGTLTTNHMVVDKIWTCGRTISIAGDNKRKDVLRSSIAGEVLDLLLQSIFQNTGAEVVKGKDGKNNILGSPTETAILEFGLLLGGEFKKQRKESTILKVEPFNSEKKRMSVLVSLSNGGENRAFCKGASEIILESCNKVINVDGKAEHLSKEQKKFITDVINGFACEALRTLCLAFKDVKDTSDVDSDSIPQENYTLIAVIGIKDPVRPGVRQAVETCLSAGIKVRIVTGDNINTAKAIARECGILTENGLAIEGPEFRDMSPRQMEETIPKLQVLARSLPLDKHKLVTYLRMEFKEVVAVTGDGTNDAPALHEADIGLAMGIAGTEVAKENADVIIMDDNFATIQNVARWGRAVYINIQKFVQFQLTVNIVALMLNFVSACISGSAPLTAVQLLWVNMIMDTLGALALATEPPHEGLMKRPPIGRDVAFISRVMWRNIIGQTIYQLIVLAILKFDGKRLLKISGSNATAILNTLIFNSFVFCQVFNEINSRDMEKINVFRGFFDSWLFIMVMVCTVGFQSIIVELLGTVADTVPLSWELWLTSILLGAGSLIVAVILKCIPVENWREASTTKHHDGYEPLPTGPDMA

>GaHMA3

MAMAGDLLRLTVLNRPKLSLGNGAKLKADRFGLLKRCPRGRFHCQPRSTPGFVLFSSLETRLESEESSIQPVGQKLKDPSVLLDVNGMMCGGCVSRVKSVISSDERVESVVVNLLTETAAIKLKREVMERETAESVAESIAQRVSECGFMAKMRVSGTGIAENMRKWQEMLKKKEELLVKSRNRVAFAWTLVALCCGAHASHILHSLGIHFGHGSFLEILHNSYVKGGLALTALLGPGRDLLVDGLLAFKKGSPNMNSLVGFGSIVAFIISAVSLLNPGLKWDASFFDEPVMLLGFVLLGRSLEEKARIRASSDMNELLSLISTRSRLVITSSDTDSSADSVLSSDAICIEVPSDDIRVGDSVLVLPGETIPVDGKVLTGRSVVDESMLTGESLPVFKEKGLMVSAGTINWDGPLRIEATSTGSNSTIAKIVRMVEDAQGQEAPVQRLADAIAGPFVYSIMTLSAATFAFWYYAGSHIFPDVLLNDIAGPDGDSLLLSLKLAVDVLVVSCPCALGLATPTAILVGTSLGARQGLLIRGGDVLERLANTGTLTEGKPTVSSVSSFTYDESEILQIAAAVERTATHPIAKAIVKKAELLNLLLPETRGQLVEPGFGTLAEVNGCLVAVGKLEWVNERFQIKASPSDLMALEHAVMRQSSSPSNYSKTAIYVGREGEGVIGAIGMSDSLRFDAKSTVSRLQRKGIKTILISGDREEAVATIAKTVGIVHEFVNASLTPQQKSRVISTLQTAGHHIAMVGDGINDAPSLAIADVGIALQTEAQETAASDAASIILLGNRLSQVVDALDLAQATMAKVYQNLSWAVAYNIVAIPIAAGVLLPQYELAMTPSFSGGLMALSSIFVVTNSLLLRLHGSEKSWKNSIAKISQMPAGP

>GaACA9

MSLRHRNTGYLEPAMSDGEDTPIVKCQHRRWRSVFAAIYSARIFVSLYKKIINKKQILRSLSYIALDVHDSDSSDDRLPSLGVDQKTLTEVVREKSLETLSKLGGVKQIAASLETDKKDGISANEADLAHRVNVFGANRYQKPPKKSFFSFVYEAFKDTTIIILLVCAVLSLGFGIKQHGITDGGYDGGSIVIAVFLVVAVSAVSNFKQNRQFEKLSKESSDIKVEVVRDGRRQFISVFEVVVGDVVCLKIGDQIPADGLFLDGHSLKVDESSMTGESDHVEINGSNNPFVLSGTKVTNGFGSMLVTSVGMNTAWGEMMSSINRELDEETPLQARLNKLTSAIGKIGLAVAVLVLAVLLIRYFTGNTKDDQGNKEYIRGKTKFDSMMNSVVEIISAAITIVVVAIPEGLPLAVTLTLAYSMKQMMADHAMVRKLSACETMGSATTICTDKTGTLTLNEMKVMEFWLGKELMGSISSEIAPNVHKLLQQAVALNTTGTVYKPNSRSLPEISGSPTEKAILSWAVSDLGMNLDDPKQNYELIQVEAFNSEKKRSGVLIRRKSESGGATQVHWKGAAEMILAMCSQYYDRSGVVKAIDEEERVEMGKVIEDMAAKSLRCIAFAHTQNPKDNERVLQESGLILLGLVGLKDPCRPGVRKAVEACINAGVNIKIITGDNIFTAKAIATECGILQPNEDLREAVIEGVQFRNYSPEERMAKINKICVMARSSPFDKLLMVQCLKQNGHVVAVTGDGTNDAPALKEADIGLSMGIQGTEVAKESSDIIILDDNFTSVVTVLRWGRCVFNNIQKFIQFQLTVNIAALVINFIAAVSSGEIPLTAVQLLWVNLIMDTFGALALATERPTNDLMTKPPVGRSKPLISNIMWRNLIAQALYQVAVLLTLQFRGKFIFDVDKKVNNTLIFNTFVLCQVFNEFNARKLEKKNIFQGLHKNKLFLGIIAITIILQVVMVEFLKRFANTQRLNWGQWGTCIGIAALSWPLGWLVKWIPA

>GaECA1

MEDAYARSVSEVLDFFGVDSSKGLTDFQVSQHARLYGKNGTPFWKLVFKQFDDLLVKILIAAALVSFLLALINGETGLIAFLEPSVILMILAANAAVGVITETNAEKALEELRAYQADIATVLRNVGCKIPADMRMIEMLSGQLRVDQAILTGESSSVEKDLESTIATNAVYQDKTNILFSGTVVVAGRARAVVIGVGANTAMGSIRDSMLRTDDEATPLKKKLDEFGTFLAKVIAGICVLVWIVNIGHFRDPAHGGFLRGAIHYFKIAVALAVAAIPEGLPAVVTTCLALGTKRMARLNAIVRSLPSVETLGCTTVICSDKTGTLTTNMMSVSKICVVHSIKNGPEVAEFGVSGTTYAPEGFIFDNTGVQLEFPAQLPCLLHIAMCSALCNESLLQYNPDKGNYEKIGESTEVALRVLAEKVGLPGFDSMPSALNMLSKHKRASYCNHYWENQFKKVSVLEFSRDRKMMSVLCNHKQMEIMFSKGAPESIISRCTNILCNNDGSTIPIDATLRAELDSRFNSFDLRRVSSLFSFAGKETLRCLALALKIMPMGQQTLSFDDEKDLTFIGLVGMLDPPREEVRNAMISCMTAGIRVIVVTGDNKSTAESVCRKIGAFDHLVDYVGHSYTAAEFEELPGTQQTMALQRMALLTRVEPSHKRMLVEALQNQNEVVAMTGDGVNDAPALKKADIGIAMGSGTAVAKSASDMVLADDNFATIVAAVAEGRAIYNNTKQFIRYMISSNIGEVVCIFVAAVLGIPDTLAPVQLLWVNLVTDGLPATAIGFNKPDSDVMKAKPRKVSEAVVSGWLFFRYLVIGAYVGLATVAGFIWWFIYSETGPKLPYTELMNFDTCPTRETTYPCSIFEDRHPSTVAMTVLVVVEMFNALNNLSENQSLLVIPPWSNLWLVASIILTMLLHILILYVPPLSTLFSVTSLSWNEWAVILYLSFPVIIIDEVLKFFSRNSHGNAYCIRFNFRFRRYDALPKKELRDK

>GaACA10

MTTILQSNLLCFEYTIQVPSATFTKSRKKWHSLFATIYCSRKFSSLITKTATANDEARVVHRSPSHVSLAVMQENSPFRIDQPTLIELVKEKKIEKLRKHGGVDGVASGLGTDTQVGVSGSAEDIERRHEAFGSNTYKKPPTKGFFHFVVEAFKDLTIMILLGCAALSLGFGIKEHGLKDGWYDGGSIFVAVFLVIGVSAVSNYRQNRQFDKLSKVSNNIQVDVVRGGRRQQISIFDIVVGDIVCLKIGDQVPADGLFTDGHSLQIDESSMTGESDHVEVNGSQNPFLLSGTKVADGYARMLVTSVGMNTTWGQMMCQISRDTNDETPLQARLNKLTSSIGKVGLAVAFLVLVVLLVRYFTGHTTDENGNREFNGSKTKSDDIINAVVGIVAAAVTIVVVAIPEGLPLAVTLTLAYSMKRMMADQAMVRKLSACETMGSATTICTDKTGTLTLNRMKVTKFWLGQESMEEGASSISPFVVDLIHQGVALNTTGSVYRASPGTEYEFSGSPTEKAILSWAVVELKMDMEKTKKCCAVLQVEAFNSQKKRSGVLIGRNDDDTVHVHWKGAAEMILALCSSYYDASGVEKDLDDDERMKFEQIIQGMAASSLRCIAFAHKQVPEEEYQNLKEQKKLKEDNLTLLGLVGIKDPCRPGVKKAVEDCQYAGVNIKMITGDNVFTARAIATECGILKPGQDLSSGAVVEGEEFRNYTPQERMEKVEKIQVMARSSPFDKLLMVQCLKQKGHVVAVTGDGTNDAPALKEADIGLSMGIQGTEVAKESSDIVILDDNFASVATVLRWGRCVYTNIQKFIQFQLTVNVAALCINFVAAVSAGEVPLTAVQLLWVNLIMDTLGALALATERPTKELMEKPPVGRTEPLITNIMWRNLLAQALYQVAILLTLQFSGESILGVTEKVNDTLIFNIFVFCQVFNEFNARKLEKKNVFEGIHKNKLFIGIIGVTILLQVVMVEFLKRFADTERLSWGQWGACIAVAAVSWPLGWVVKCLPVPQKPIFSYLKWWK

>GaACA4

MGTFWNENFDLKPKHSSEEALEKWRKVVGFVKNPKRRFRFTANLSKRYEAAAMRRSNHEKLRIAVLVSKAALQFISGVKPSESDYVVPEEVKAAGFELCAEELGSIVENQDVKKLKIHGGVDGIAEKLSTSTTDGLSSDSGLLNKRQEVYGINKFAEAEAKGFLVFVWEALQDMTLMILGVCALVSLIVGIAMEGWPKGAHDGLGIVASILLVVFVTATSDYRQSLQFKDLDKEKKKITIQVTRNACRQKMSIYDLLPGDIVHLNIGDQVPADGLFVSGFSVLIDGSSLTGESEPVIVNVDNPFMLSGTKLQDGSCKMMVTSVGMRTQWGKLMATLSEGGDDETPLQVKLNGVATIIGKVGLFFAVVTFAVMVQGLFTSKLQEGTIWSWSGDEALKLLEFFAVAVTIVVVAVPEGLPLAVTLSLAFAMKKMMNDKALVRHLAACETMGSATNICSDKTGTLTTNHMTVVKSCICMDVREVGNNNKASLCSEIPESAVKLLLQSIFTNTGGEIVINKDGKREILGTPTETALLEFGLSLGGDSQAERLASKLVKVEPFNSTKKRMGVILELPEGGLRAHTKGASEIVLAGCDKVINSNGEVIPLDAESINHLNATINQFANEALRTLCLAYMELENGFSPDNAIPVSGYTCIGIVGIKDPVRPGVKESVAICRAAGITVRMVTGDNINTAKAIARECGILTDDGIAIEGPDFREKSQEEMLALIPKIQVMARSSPMDKHTLVRQLRSIDEVVAVTGDGTNDAPALHEADIGLAMGISGTEVAKESADVIILDDNFSTIVTVAKWGRSVYINIQKFVQFQLTVNIVALIVNFSSACLTGTAPLTAVQLLWVNMIMDTLGALALATEPPTDELMKRAPVGKKGNFISNVMWRNILGQSFYQFMVIWYLQVKGKGMFSLDGPDSDLKLNTIIFNSFVFCQVFNEISSRNMEEINVFKGILNNYVFVAVLGCTAVFQVIIIEFLGTFASTTPLTCLQWFVSVFIGFLGMPIAAALKTIPV

>GaHMA1

METHSISLTNFSPLTRPLRPSRLGRLNSFHFKPLFFSPLSTRYKSLFLPLNSHTIRIRCVANHEHHHHHEHDHDHDHDHHHHHHHGSGLLNGPQKAVIGFAKAIRWMDLANFLREHLHLCCCATALFIAAAACPYLVPKPAVKPLQNSFLVLAFPLVGVSAALDAITDIAGGKVNIHVLMALAAFASVFMGNALEGGLLLAMFNLAHIAEEFFTSRSMIDVKELKENYPDSALVLNLDDDNLPNVSDLSYQSIPVHDVEVGSYILVTTGEAVPVDCEVFHGSATITIEHLTGEIKPLEAKAGDRIPGGARNLDGRMIVKVLKTWKESTLSRIVQLTEEAQLNKPKLQRWLDEFGEQYSKVVVVLSVAIAVLGPFLFKWPFISTAVCRGSIYRALGLMVAASPCALAVAPLAYATAVSSCARKGILLKGGQVLDALASCHTVAFDKTGTLTTGGLMFKAIEPIYGHIIGNMKTNLTSCCVPSCEVEALAVAAAMEKGTTHPIGRAVVDHSIGKDLPSVSVESFEYFPGKGLIATLNSAESGTRGGKMLKASLGSIEFITSLCKSEVESRKITAAVNASSFGTDFVHAALSVDEKVTLIHLEDRPRPGVLDVISELKDKAKVRVMMLTGDHKLSAWRVANAVGINEVYCSLKPEDKLNHVKRISGDMGGGLIMVGEGINDAPALAAATVGIVLAHRASATAIAVADVLLLQDNISGVPFSIAKARQTTSLVKQNVALALTCIILASLPSVLGFLPLWLTVLLHEGGTLLVCLNSIRALNDPSWSWGQDLRNLIGKLKSKLALLRHNATSSPIQTAPL

>GaACA11

MTTIFEPNLICSEHTIQVPPSLSTSDKAWHSIFASKKFSSLLTKSPTPKEEAKVVRRSPSHVSVNVMQENPLYQIDQRTLIELVKEKKLDRLQEFGGVNGVASNFGTDTQVGISGGADDVARLRNTFGSNTYNKPPTKGFFHFVIEALKEPAVLILLGCAALSLGFKIKAHGHKDSWYEVGSNFAAVFLPIVVSAISNYMQNRQFKELSRTNNKILVDVVRGGQRQQIRMFDVVVGDIVCLKMGDQVPADGLFLDGHSLQVDESSMTGETEHTEVNSSQNPFLLSWTKVAHGDARMLVTSVGSNTTWGQISRETNEQTPLQARLNKLSSSIAKVGLAVAFPVLLVLLVRYFTGHTKDEKGNREFHRSKAKTSDIINSIVGIATTAITTVAEGLPLVVTLTLAYSMKKMVADQAMVRKLSACETMGSTTTICTNKTGTLTLNRMEVTKFWLGQESMEEGASSISPFVVDLIHQGVALNTTGSFYRASPGTEYEFSGSPTEKAILSWAVVELKMDVEKTKKSCAVLQVEAFISQRRRSGVLIERNDDDTVHVHWKGAAEMILAMCSSYYDASGVVKDLDDGERTKFEEIIQGMAASSLRCIAFAHKQVPEEEYQNLKEQKKLKEDSLALLGLVGIKDPCRPGVKKAVEDCQYAGVNIKMITGDNVFTARAIAIECGILKPGQDLSSGAVVEGEEFRKYTLQERMEKVEKIQVMARSSPCDKLLMVQCLKQKGHVVAFTGDGTDDAPALKEADIGLSMGIQGTEAAKESSDIVILDDSFASVATVLRWGRCVYTNIQKFIQFLLTVNVSALCINFIAAVSTGEVPLTTVQLLWVNLYMDTLGALALATELPTKELLEKPPVGRTEPLITNIMWRNLLAQALYQIAVLLTLQFNGESIFGVTKKVNHTLIFNTFMLCQVFNNFNARKLEKKNVFDDIHKNKMFIGIIGVTIVLQVVMVEFLKRFTDTERLNWGQWGACIAIAAASCPLGWAVKCLPVPKKPIFSYQKW

>GaACA5

MSSIFKGSPYRRPNDLEAGSSRSAHSDDEDHESCADPFDITSTKNAPIDRLRRWRQAALVLNASRRFRYTLDLKKEEEKKQILRKIRAHAQAIRAAYLFKQAGEQVNGTTTPHPTPGSDFAFGPEQLASVTRDHNFNALQEYGGVNGLAESLKTNLQKGIPGDDSDLLKRRNAFGSNTYPRKKGRSFWRFVWEACQDLTLMILVVAAVASLALGIKTEGPKEGWYDGGSIAFAVFLVIIVTAISDYRQSLQFQKLDEEKRNIHLEVVRGGRRVEISIYDIVVGDIIPLNIGDQVPADGILISGHSLAIDESSMTGESDIVQKDAKQPFLMSGCKVADGSGTMLVTGVGINTEWGLLMANLSEDTGEETPLQVRLNGVATFIGFVGLSVAFAVLVVLLVRYFTGHTEDSNGRQQFVAGKTSVGNAIDGAIKIVTVAVTIVVVAVPEGLPLAVTLTLAYSMKKMMADKALVRRLSACETMGSATTICSDKTGTLTLNQATYAGGRKNDPPERRSELPDTLVSLLIEGIAGGGDVEVSGSPTEKAILNWGIKLGMDFDAVRSGSSIVHVFPFNSEKKRGGVAIRLLSLRSLFEKFLGRLVTTAAQASNSNPIFISKPDSKVHIHWKGAAEIVLAACTRYLDTNGEAVAMDEEKMAFFEKAIETMAAGSLRCVAIAYRSYDSEKVPTNEEELAKWALPEDDLVLLAVVGIKDPCRPSVKKSVQLCQKAGVKVRMVTGDNLKTARAIALECGILSSDAPESSLIEGKVFRSLSDSEREEVAEKISVMGRSSPNDKLLLVQALRRKGHVVAVTGDGTNDAPALHEADIGLAMGIQGTEVAKESSDIIILDDNFASVVKVVRWGRSVYANIQKFIQFQLTVNVAALVINVVAAVSSGDVPLNAVQLLWVNLIMDTLGALALATEPPTDHLMHRPPVGRREPLITNIMWRNLLIQAIYQVTVLLVLNFDGKKILHLEHESKEHANRVKNTLIFNAFVLCQIFNEFNARKPEEVNIFRGLSKNYLFIGIVAITIILQAIIVEFLGKFAKTEKLSWQLWLVSIGIGFISWPLAILGKFIPVPETPVSRVFSRMFYRRRNQNVSGSQKHEDSNATSIRN

>GaACA6

MSGTSSGNGLLHLNDVEAGLSKDNADLDHHLDPDAGTSDPFDIAHTKNAAPETLKRWRQAALVLNASRRFRYTLDLKKEEEKEQRKRMIRAHAQVIRAALLFKLAGENQIVSSAPVASPSAGDDYKIGLEHLASMTRDHKLSALEQYGGVKGLSGLLRTNLEKGIDEDEADLLNRRNVFGSNTYPRKKGRSFWMFLWEAWQDLTLIILIIAAAVSLGLGIKTEGLKEGWYDGGSIFFAVFLVILVTATSDYRQSLQFQNLNEEKRNIQLEVVRGGRTVKVSIYDLVVGDVVPLKIGDQVPADGVLIAGHSLAIDESSMTGESKIVHKNQNDPFLMSGCKVADGFGTMLVTGVGINTEWGLLMASISEDTGEETPLQVRLNGVATFIGIVGLSVAVSVLAILLARYFTGNTEDPDGATQFIKGRTKFDDAFNDVVKIFTIAVTIVVVAVPEGLPLAVTLTLAYSMRKMMADKALANTDTQVRRLSACETMGSATTICSDKTGTLTLNEMTVVEAFVGKKKINPPADSSQLHLSVVSLLSEGVAQSTMGNVFVSKDGGDVEISGSPTEKAILSWAIELGMKFDAIRSESTILHVFPFNSEKKRGGVALRRDRNKDMNGRNICCPGLVDRSDAEVHIHWKGAAEIVLAACSGYLDSNGCLQSMNEDKEFFEAAIDEMAANCLRCIALAYRLCEQEKVPSNEESFDDWVLPEDNLVLLAIVGIKDPCRPGVKDAVKICTDAGVKVRMVTGDNIQTAQAIALECGILSSAQDVTEPTIIEGRVFRALTEIEREQVARKIMVMGRSSPNDKLLLVQALRKGGDVVAVTGDGTNDAPALHEADIGLSMGIQGTEVAKESSDIIILDDNFASVVKVVRWGRSVYANIQKFIQFQLTVNVAALVINVAAAISSGDVPLNSVQLLWVNLIMDTLGALALATEPPTDNLMHRSPVGRREPLITNIMWRNLLIQALYQVTVLLVLNFRGMTILQLEDDGNREHAYKVKNSLIFNAFVMCQIFNEFNARKPEEVNCFKGVTKNYLFMGIIGFTFILQIIIIEFLGKFTSTVRLDWQLWLVSLGIGIISWPLAIVGKLIPVPKTPVASYFIKPFQQCKRSRDA

>GaACA7

MMGDSTLTSDKFCLNLVRILTWGHKLKSQVVIFVLFLADDLLQAALVLNASRRFRYTLDLRKQEEKEQRKRMIRAHAQVIRAALLFKLAGEKEIVSGTPVTLPGAAGDFAVGLEQLASMTRDHKLSALQQYGGVKGLSDLLKTNLETGIYGDEVDLLNRKTAFGSNTYPRKKGRSFWRFLWEAWQDLTLIILIVAASVSLGLGIKTEGLKEGWYDGGSIFLAVLLVIVVTATSDYRQSLQFQNLNEEKRNIQLEVLRGGRTVKVSIYDVVVGDVVPLKIGDQVPADGVLVTGHSLAIDESSMTGESKIVHKDKKEPFLMSGCKVADGVGTMLVTGVGINTEWGLLMASISEDTGEETPLQVRLNGVATFIGIVGLSVAVSVLVVLLARYFTGHTEDPDGTKQFIKGRTNFDDAFNGVVKIFTIAVTIVVVAVPEGLPLAVTLTRDLQVRRLSACETMGSATTICSDKTGTLTLNEMTVVEAFVGKKKINPPSDSSQLPASVVSLLNEGVAQNSTGNVFVPKADSEVRIHWKGAAEIVLTSCSGYIDSNGCLQSINEDKEFFKAAIDEMAVNSLRCVALAYRLCEKEKVPTDEEGFNGWILPEDNLVLLAIVGIKDPCRPGVKDAVKICMDAGVKVRMVTGDNIQTAKAIALECGILSSAEDATEPTIIEGRVFRELSDKEREQIAKKITVMGRSSPSDKLLLVQALRKGGDVVAVTGDGTNDAPALHEADIGLSMGIQGTEVAKESSDIIILDDNFASVVKVVRWGRSVYANIQKFIQFQLTVNVAALVINVVAAISSGDVPLNSVQLLWVNLIMDTLGALALATEPPTDNLMHRSPVGRREPLITNIMWRNLLLQIFNEFNARKPEEINCFKGVTKNYLFMGIIGFTFVLQIIIIEFLGKFTTTVRLNWKLWLVSLGIGIISWPLAIVGKLIPVPKTPVSGYFTKAFRRCRTDRNA

>GaACA1

MFAGASNIGGCNEHGDEDTLSVSLLNTTANSSRKNWTRLYNFIIFLRKAAQIVRDKDLTSLEEEGGVRRVKSRLQSILAHEIDRSFEEQATAVIVTGTTFDAKFFVVSFVEASKTPTIFPLLVFAALSFTIEMMEAEPKHGWHDGVAILVAVFMLLIFRSVANYRRARRLQKRKKFHVSVLKDGQPKTITISTLVVGHILCLKKGDFVPADGLFVSENGLKLDDKLNPNINRDDNPFLLAGSKIMEGEGHMLVTSVGDNSVLPTVDPDEKSLMEDQIDKTNAYMEYVGLSISLLVSALVLINLLARKMDKNSNIMPEMKGDVSAYRVIKIFARVFLNPRGKVQIFTGVLTVMVTSLQHGMPVVITISLSYWKKKMASGDANVQSLSSCGTIGIVSVICFDENTVVACKEVMGSIVGALKEEEVGCKLMSKDELATAQVMAHEIGILNPDLRDMAIENKDLHELAATAEGMNKIAVIGSCKLKDKIRILQRLKQEGHVVAFIGGMATNDDLALKAADVGITICECSTKMARENSEIVISSRNSLRSLIRCLKMGKCAYGNVRTFTEIQLTATLASLLVTLVTTSILDESPITGIHMLWVNSIICILGGLMMVMESYGHQELMNNQPARRMKSLLTKTMWRNVAIRAASDACHLLVLQFIGQAILQINKDVVKTMVFNGFILCQVLDLFISTIIIFARNEEVSVSVVRFMCSSHWFLMASGGVMAMQVVVVELLQSLAEYERLNVMQWGFCFIYAAWLCGTGLTVKLIADSASEVLWSSRSSGSLFGYLRLRRSSLRLFVIPFSVCVVASFSYCYVNPDIA

>GaALA1

MKRYVYINDNESSHELYCDNRISNRKYTVLNFLPKNLWEQFSRFMNQYFLFIACLQLWSLITPVNPASTWGPLIFIFAVSASKEAWDDYNRYLSDKKANEKLVWVVRQGIRKQIQAQDIHVGNIVWLRENDEVPCDLVLIGTSDPQGLCYVETAALDGETDLKTRVIPSACMGIDFELLHKIKAGVIECPNPDKDITRFDANLRLFPPFIDNDVCPLTIKNTILQSCYLRNTEWACGVAVYTGRNETKLGMSRGIPEPKLTAMDAMIDKLTGAIFVFQIVVVMVLGIAGNVWKDTEARKQWYVQYPIEGPWYELLVIPLRFELLCSIMIPISIKVSLDLVKSLYAKFIDWDAEMIDYETGIPSHATNTAISEDLGQVEYIMTDKTGTLTENRMIFRRCCISGVFYGNESGDALKDTKLLNAVAGGSPDVVQFLTVMAICNTVVPIKSKTGAISYKAQSQDEDALVNAAAQLHMVYANKNANILEIRFNGSVIKYEVLEILEFTSDRKRMSVVVKDCQTGKIVLLSKGADEAILPYAFVGQQTRTFIEAVEQYAQLGLRTLCLAFRELREDEYQEWSLMFKEASSTLVDREWRIAEVCQRLEHDFEVLGVTAIEDRLQDGVPETIETLRKAGINFWMLTGDKQNTAIQIALSCNFISPEPKGQLLLIDGKTEDEVCRSLERVLLTMRITSSEPKDVAFVVDGWALEIALKHYRKAFTELAILSRTAICCRVTPSQKAQLVELLKSCDYRTLAIGDGGNDVRMIQQADIGVGISGREGLQAARAADYSIGKFRFLKRLILVHGRYSYNRTAFLSQYSFYKSLVICFIQIFFSFISGVSGTSLFNSVSLMAYNVFYTSVPVLVSVLDKDLSEGTVMQHPQILFYCQAGRLLNPSTFAGWFGRSLFHAIVVFVITIHAYAYEKSEMEELSMVALSGCIWLQAFVVALETNSFTILQHLAIWGNLVAFYVINWIFSAIPASGMYTIMFRLCRQPSYWITMSLIVAAGMGPVLALKYFRYTYRPSKINTLQQAERMGGPILTLGNIEPHPRPMEKEVVSPLQISQPKNRNPVYEPLLSDSPNSSRRSLGSGAPFDFFQSQSRLSSSYSRNCKDN

>GaALA2

MDSKTPVENLCCIEPALSSSSRRSNFSARSKSLGGSSIREVNFSDVGPKPVRYGSQGAESETYSMSQKEINDEDARLVHINDPVNTNERFKFAGNSIRTAKYSILTFLPRNLFEQFHRVAYVYFLVIAVLNQLPQLAVFGRTASILPLAFVLLVTAVKDAYEDYRRHRADRIENNRLALVLVNNEFQQKKWKNIQVGEIIKIQANETIPCDMVLLSTSEPTGVAYVQTTNLDGESNLKTRYAKQETLQKIPEKENVSGLIKCEKPNRNIYGFQANMEVDGKQVSLGPSNIILRGCELKNTTWAVGVAVYAGSETKAMLNNSGAPSKRSRLETHMNLEIIFLSLFLVALCTVVSVCAAVWLRRHRDELDYLPFYRRKDFSEDEEKNYNYYGWGLEIFFTFLMSVIVFQIMIPISLYISMELVRVGQAYFMIRDAEMYDESSNTRFQCRALNINEDLGQIKYVFSDKTGTLTENKMEFQCASIWGVDYNGGKATSQDQKDGYFVQADGQVLRPKMVVKTDPELLQYVRNGKETKEGSYVHDFFLALAACNTIVPIIVDTPDPTLKLIDYQGESPDEQALVYAAAAYGFMLIERTSGHIVIDIQGERKRFNVLGLHEFDRDRKRMSVILGFPNQSVKVFVKGADTTMFSVIDRSLNTSIIRATEAHLQSYSSIGLRTLVIGMRELSTSEFEEWHSAFEVASTALMGRARLLRKIASNIESNLCILGASGIEDKLQQGVPEAIESLRTAGIKVWVLTGDKQETAISIGYSSKLLTSKMTQVIVNSNSKESCRKSLEDAIIMSKKLTTTSGTTNETGRTLGTGSTPVALIIDGTSLVYILDSELEERLFELACNCSVVLCCRVAPLQKAGIISLVKKRTSDMTLAIGDGANDVSMIQMADVGVGISGQEGRQAVMASDFAMGQFRFLVPLLFVHGHWNYQRMGYMILYNFYRNAVFVLVLFWYVLFTCFTLTTAINEWSSVLYSVIYTSVPTIVVGILDKDLSRLTLLKHPQLYGAGHRDECYNKTLFWITMLDTLYQSVVVFFIPLLAYWGSTIDAASIGDLWTLAVVILVNLHLAMDVIHWNWITHAAIWGSIIATFICVIVIDAIPSLVGYWAIFEIAKTRLFWFCLLAIIVTALIPRFVVKVLYQFYAPCDVQIAREAEKFWAQSQSAAVEVEMSPILDHPRR

>GaALA3

MDSRTSFEDFYSIESAFSSSSRRSNFSVQSKASGGNSIREVNFGDLGTKPVRYGSHGADSETYSISMSQKEINDEDARLVHINDPVQTNERFEFSGNSIRTGKYSILTFLPRNLFEQFHRVAYIYFLLIAVLNQLPQLAVFGRGASILPLAFVLLVTAVKDAYEDYRRHRSDRIENNRLASVLVDDQFQEKKWKNIQVGEIIKIYANETIPCDMVLLSTSDPTGVAYVQTINLDGESNLKTRYAKQETLMKIPENDEVIGLIKCEKPNRNIYGFQANMEVDGKQLSLGPSNIILRGCELKNTAWAVGVAVYTGRETKAMLNSSGAPSKRSRLETHMNLEIIFLSLFLIALCTVVSICAAVWLRHHRKELDYLPFYRRKEFSDGEEENYNYYGWGLEICFTFLMSVIVFQIMIPISLYISMELVRVGQAYFMIRDTRMYDESSNSRFQCRALNINEDLGQIKYVFSDKTGTLTENKMEFQCASIWGVDYSGGNAISLDQNDGYFVKVDGKVLRPKMKVKTDPELLQFARNGKETQEGSHVYDFFLALAACNTIVPLIVDTPDPTVKLIDYQGESPDEQALVYAAASYGFMLIERTSGHIVIDIQGERQRFNVFGLHEFDSDRKRMSVILGFPDRYVKVFVKGADTSMFSVIDRSMDMKVIRTTEAHLHSYSSLGLRTLVVGMRELSTSEFKQWHSTFEAASTALMGRASLLRKVANNIENNLHILGASGIEDKLQQGVPEAIESLRTAGIKVWVLTGDKQETAISIGYSSKLLTSKMTQIIINSKSMESCRKSLEDAIIMSKKLTTTSAISGTTNNTGGTSGAGSTPIALITDGTSLVYILDSELEERLFQLSCNCSVVLCCRVAPLQKAGIVSLVKKRTADMTLAIGDGANDVSMIQMADVGVGISGQEGRQAVMASDFAMGQFRFLVPLLLVHGHWNYQRMGYMILYNFYRNAVFVLVLFWYVLFTSFTLTTAITEWSSVLYSVIYTALPTIVVGILDKDLSRRTLLKYPQLYRAGQKQECYNKKLFWITMIDTFWQSAVTFFIPLLAYWESTIDASSIGDLWTLAVVILVNLHLAMDVNRWNWLTHAAIWGSIIATFICVIVIDALPFLVGYWAIFEIAKTGLFWLCLLAIIVAALIPHFVVKALYQLYAPCDVQIAREAEKFRTLCESGAVEIEMNSILEVPRR

>GaALA4

LYSADCLSRQQFSLECPTTDSGNQVSWGSMELHNNDNTYTSFDISRSSSQAQENLSKSRRIRNKSVDFDDNLPYSENPRLIYINDPRRTNDKYEFTGNEIRTSKYTLITFLPKNLFIQFHRVAYLYFLAIAALNQLPPLAVFGRTVSLFPLLFVLCVTAIKDGYEDWRRHRSDRNENNREALVLQVGEFQRKKWKKIRAGEVVKIHADETIPCDMVLLGTSDPSGLAYIQTMNLDGESNLKTRYARQETASSIFEGCNVSGLIRCEQPNRNIYEFTANMEFNGHKFPLSQSNIVLRGCQLKNTDWIIGVVVYAGQETKAMLNSAVSPSKRSKLEGYMNRETFWLSIFLLVMCSVVAVGMGLWLHRHKDELDTLPYYRKTYIREGRENGKTYRYYGIPMETFFSFLSSVIVFQIMIPISLYITMELVRLGQSYFMIEDKHMYCSNSGSRFQCRSLNINEDLGQIRYVFSDKTGTLTENKMEFRKASVYGKDYRSSNLTDDSLQDNSITDAAVRSRWKLKSEISVDSELMDLLHKDLAGDERIAAHLFFLTLAACNTVIPIVSQDASSSHGSSDSSGEVKTIDYQGESPDEQALVSAASAYGYTLHERTSGHIVIDINGNKLRLDVLGLHEFDSVRKRMSVVIRFPDNTVKVLVKGADSTMFSILADTEKVDQIRQATRSHLTEYSSEGLRTLAVAARDLTDAELEQWQCRYEDASTSLIDRAAKLRQTAALVECNLKLLGATAIEDKLQDGVPEAIESLRQAGIKVWVLTGDKQETAISIGLSCKLLTADMQQIIINGNSEEECRNLLTDAMTRHGVQPANRKKQNSTRRKNSENGYLEIPDDTKSSNVLQQHSGKEEPDVCAPLALIIDGNSLVYILEKDLQSELFDIATSCKVVLCCRVAPLQKAGIVDLIKSRTDDMTLAIGDGANDVSMIQMADVGVGICGQEGRQAVMASDFAMGQFRFLKRLLLVHGHWNYQRVGYLVLYNFYRNAVFVLMLFWYVPFFVVKSELFEFLGCIGQKKGGTY

>GaALA5

MSGWDNIRSSTRSQQGRSHSLNQREPSRTVTLGRVQPQAPAFRTIYCNDRDANFAHRYRGNSVSTTKYNFFTFLPKGLYEQFRRVANLYFLMVSILSATPYSPVHPVTNVVPLSLVLLVSLIKEAFEDWKRFQNDMAINSTPVDVLQDQRWESIPWKKLQVGDIIRVKQDGFFPADMLLLASTNADGVCYIETANLDGETNLKIRKALERTWDYVTPEKACEFKGEVQCEQPNNSLYTFTGNLVIDNQTLPLSPNQILLRGCSLKNTEFVVGVVIFTGHETKVRYKSLTLSLIRIDAILWEFGMSLQVMMNSMNVPSKRSTLERKLDKLILTLFGTLFTMCLVGAIGSGVFIDRKYYFLGLSKSVEDQFNPNRRFLVVLLTMLTLLTLYSTIIPISLYVSIEMVKFIQSTQFINKDLNMYHAETDTPALARTSNLNEELGQVEYIFSDKTGTLTRNLMEFFKCTIGGEIYGTGMTEIERGVAERKGIKVQEVPTSINSVREKGFNFDDVRLMRGAWRNEPNPDACKEFFRCLAICHTVLPEGDESPEKIKYQAASPDEAALVLAAKHFGFFFYRRTPTMIYVRESHVERMGKIQDVSYEILNVLEFNSTRKRQSVVCRYPDGRLVLYCKGADTVIYERLVGGSDDLKKVTREHLENFGSAGLRTLCLAYKDLAPDVYESWNEKFIQAKSSLRDRERKLDEVCLSMWYHFLLEETKYIFFSFVIMIFLSSLHLKKYIDSCIICFDFYFLFIYYFHNNAVMILFTEILALISELIEKDLILIGATAIEDKLQEGVPDCIETLRAGIKMWVLTGDKMETAINIAYACNLLNNEMKQFIISSETDAIREVEERGDQVEIARFIKEEVKKQLKKCLDEAQQYFHSVSGPKLALIIDGKCLMYALDPSLRIMLLNLSLNCSSVVCCRVSPLQKAQVTSLVKKGARKITLSIGDGANDVSMIQAAHIGVGISGLEGMQAVMASDFAIAQFRFLKDLLLVHGRWSYIRLCKVVTYFFYKNLTFTLTQFWFTFYTGFSGQRFYDDWFQSLYNVIFTALPVIIVGLFDKVPQFHLFLRILFPCYKLDLFKTLLDVQDVSSSLSKRYPELYKEGIKNMFFKWRVVAIWAFFAVYQSLVFYYFVTVSSSTSQGSSGKMFGLWDVSTMAFTCVVVTVNLRLIMICNSITRWHYISVGGSIVAWFLFIFLYSGIMTPYDRQENIFWVIYVLMSTFYFYITLLLVPVAALLGDFLYLGVQRWFFPYDYQIVQEIHKDEADDSGRTDLLGIDNQLTPDEARSYALSQLPRELSKHTGFAFDSPGYESFFASQLGVYAPQKAWDVARRASMRSKPK

>GaALA6

MSGWGTPSRTVTLGRVQPQAPALRTIYCNHREANFAHRYKGNSISTTKYNVFTFLPKGLYEQVILLEFYRTETGIWLCDSLGISYDNFLLFRRVANLYFLMVSILSATPYSPVHPITNMVPLSLVLLFSLIKEAFEDWQRFQNDMTINNTLVDVLQAQGWESLQWKKLQVGDIIRVKQDGFFPADLLLLASTNVDGICYIETANLDGETNLKIRKALERTWDYVTPEKACEFKGEVQCEQPNNSLYTFTGNLVMDNQTMPLSPNQILLRGCSLKNTDYIVGTVIFTGHETKVMMNSMNVPSKRSTLERKLDKLILALFSTLFSMCLLGAIGSGIFIDRKYYYLGLSQSVEDQFNPSKRFLVIILTMLTLLTLYSTIIPISLYVSIETIKFIQSSQFINKDLNMYHAESNTPASARTSNLNEELGQVEYIFSDKTGTLTRNLMEFFKCSIGGEMYGTGMTEIEMGVAERKGIKVQEAKTSTNSLREKGFNFDDVRLMQGAWRNEPNHDACKEFFRCLAICHTVLPEGEETPEKIRYQAASPDEAALVLAAKNFGFFFYRRTPTMIYVRESHVEKMGKTQDVSYEILNVLEFNSTRKRQSVVCRYRDGRLVLYCKGADTVIYERLGTGNGDLNKVTRVHLEQFGSAGLRTLCLAYRDLAPDLYESWNEKFITAKSSLRDREKRLDEVAELIEKELILIGATAIEDKLQEGVPNCIQTLSRAGIKIWVLTGDKIETAINIAYACNLLNNEMKQFIISSETDAIREVEERGDQTEIARFMKEEVKKQLKQFLDEAPQYFCSPSGPKLALVIDGKCLMYALEPSLRIMLLTLSLNCSSVVCCRVSPLQKAQVTSLVKKGARKITLSIGDGANDVSMIQAAHIGIGISGLEGMQAVMASDFAIAQFRFLEDLLLVHGRWSYIRLCKVVMYFFYKNLAFTLTQFWFTFGTGFSGQRFYDDWFQSLYNVIFTALPVVIVGLFDQDISSSLSKKYPELYKEGDNMFWVIYVLMSTSYFYVTLLLVPVAALLGDFLYLGVQRWFFPYDYQIVQESHKDDADSINKPDSQETKEHLTPDEARSNEISQLPKEVSKHSGFAFDSPGYESFFATQFGKYVPPKAWDVARRASMKSKSKPK

>GaALA7

MAKGKIRAKIKRSQLYTFACGRKSTQEAVPFVEGAGCSRTVHCNQPHMHRKRPLKYRSNYISTTKYNFLSFLPKSLYEQFHRVANLYFLAAAVLSLTPLSPFSAVSMIAPLAFVVGLSMAKEGLEDWRRFMQDMNVNSRKVKFHKEGGVFEFKQWQQVEVGDVVRVEKDEFFPADLLLLSSSYEDGICYVETMNLDGETNLKVKRAMDVTLSLEEDEDFKNFTGLIKCEDPNPSLYTFVGNLDYDRQTYSLEPAQVLLRDSKLRNTGFVYGVVIFTGFDTKVMQNSTKSPSKRSRIEKKMDYIIYVLFSLLLVISLISSLGFALKTKFHMPNWWYMRPDDTEQFYNPKEPFISGLSHLVTALILYGYLIPISLYVSIEVVKVLQASFINQDLQMYDDDTGIPAQARTSNLNEELGQVDTILSDKTGTLTCNQMDFLKCSIAGTAYGVRASEVERAAAQQMANDLEQDGRQSSVSSKRSKQDIELETVVTSKDGKDNKPPIKFFSFEDDRMTEGNWRKEPNVDMIILFCRILAVCHTAIPELNEETGTYTYEAESPDEGAFLVAAREFGFEFFKRTQTSVFVRERYIGSGQTTEREYKILNLLEFTSKRKRMTVIVRDEEGQIILMCKGADSIIFDRLAKKGKTYLEDTTKHLNIYGEAGLRTLALAYRKLEESEYSAWNNEFQKAKTSIGADRDTNLEKVAEMMEKELTLVGATAVEDKLQNGVPQCIDKLAQAGLKLWVLTGDKMETAINIGFACSLLREGMKQICITTLDSESKEAIKENILTQVNNGSQMIKLEKDPYAAFALIIDGKTLAYALEEDMKLLFLGLAVDCASVICCRVSPKQKALVTRLVKEGTGKVTLGIGDGANDVGMIQEADIGVGISGVEGMQAVMASDFSVSQFRFLERLLVVHGHWCYKRIAQMVCYFFYKNIAFGLTLFYFEAFTGFSGQSVYDDWYMILFNVVLTSLPVISLGVFEQDVSSDVCLEFPTLYQQGPRNLFFDWYRILGWMGNGLYSSLVIFSLNIVIFYDQAYRVEGQTTDMSILGTIMFTCIICALNFQVSLIMTHYTWMQHVLIWFSIVAWFIFLFIYGMLPPKISHSAYQILTEALASAPVYWITTLLVTIACTLPYLAHMSLQRCFNPMDHHVIQEIKYLKKDVSDQVMWRREKTKAREKTKIGFTARVDALIRSLRGKLNKKTHSIESNKSYPPS

>GaALA8

MARVRKRNIHLSKSCSFSCCRRPSQPSSTDEHDRIGQKGYSRVVYCNEPDCEEQIRLKYRGNYVSTTKYTAANFIPKSLFEQFRRVANIYFLVVACVSFSPLAPYSAPSVLVPLIVVIGATMAKEGIEDYRRRQQDVEANNRTVEVYDGNSSFNETKWKNLRVGDIVKVHKDEYFPADLLLLSSEYEDGVCYVETMNLDGETNTKSKHPLEVTSFIRDVETVKGFRAVIKCEDPNEHLYSFVGTLYYECQQYALSPQQILLRGTKLKNTDYINGVVIFTGHDTKVMQNAMDPPSKRTRIERRMDRIIYVLFSALILVSFVGSLLFGIETKKDGGDYGRWYLRPDITTVFFDPRRPSVAAFLHFLTGLMLYGYLIPISLYVSIEICKVLQSIFINQDQAMYDEETKRSAHARTSNLNEELGQVCTILSDKTGTLTCNSMEFVKCSIAGTAYGRGMTEVEIALARKRGETLDEQRDIDTVESREPVKGFNFRDGRIMNRKWVNEPNRDYIEKFFRVLALCHTAVPEVLDPGKIFYEAESPDEAAFVIAAREVGFQFFKRNQTSIQLRELDRSSGEIVDRVYELLHVLEFSSARRRMSVIVRNPERQLLLLAKGADSVIFERLSEEGRMFEAETKEHIERYSEAGLRTLAVAYRELDDDEYKRWEQEYVKAKTSVSADRDDLLDEKADMIERGLILLGATAIEDKLQKGVPDCIDKLAQAGIHIWVLTGDKKGTAINIGYACSLLRHGMKEILVILEKPGIEAIERDKEEFAKAYKIVEKQIDEGISQVTGESNTQFGLIIDGKSLIFALDKNLIIRFMELAMKCATVICCRSSPKQKAIVTRWVKSKTGRTTLAIGDGANDVGMIQEADIGVGITGVEGMQAAMSSDFSIAQFHFLERLLLVHGHWCYRRITMMICYFFYKNITFGFTLFWFEAYSSFSAQPAYNDWYMSSYNVLFTSLPVIALGVFEQDFSARHCLKYPSLYKEGIDNVLFRWIHILGWMFNGVLSSVIIFFLTTNSITGQAFRKDGQVADYSILGLTMYTCVVLAVNCQMALSINYFTWIQHLFIWGSITLWFKFLLVYGSIPPTLSTTAYKVFIEACAPSIRYWLTIILVVIATLLPLFSYRAFQTRFQPMVHDSIQTRRP

>GaALA9

MARIRKRSIQLSKLCSFACCRSSSQDEHAQIGQKGYSRVVYCNEPDSQEQIRLRYRGNYVSTTKYTAVNFIPKSLFEQFRRVANIYFLVVACVSFSPLAPYSAPSIILPLVVVIGATMAKEGVEDWRRNLQDIEANNRKVEVYDKSSCSFRQTKWKDLRVGDLVKVCKDEYFPADLLLLSSSYEDVVCYVETMNLDGETNLKLKHALNVTSFYSSAESLKEFRAVIKCEDPNEHLYSFIGTLYFDCQQYSLSPQQILLRDSKLKNTEYIFGVVIFTGHDTKVMQNATDPPSKRTKIERRMDRIIYVLFSTLILVSFIGSLFFGIETNNDLSGGNYTRWYLRPDKTTVFFDPRRPAVAAFLHCLTGLMLYGYLIPISLYVSIEICKVLQSIFINQDQAMYDEEMDRPAHARTSNLNEELGQVHTILSDKTGTLTCNSMEFVKCSIAGTLYGRGMTEVEIALARKRGEQLTEQAPIDASESKKSIKGFNFRDERIMDGKWVSEPQSDVIQKFFRVLATCHTAVPEVMGPDEIMYEAESPDEAAFVIAAREVGFEFFARNQTSIKLRELDPTSGIRVDRVYELLHVLEFSSARKRMSVIVKNPENQLLLLAKGADSVIFERLSKEGRAYEERTKEHIEKYSEAGLRTMAVAYRELDNDEYKTWEQEFLKAKTSVSADRDVLMDELACQIERDLILLGATAVEDKLQKGVPGCIDKLARAGIGIWVLTGDKRGTAINIGYACSLLRHGMKQIVITLELPEILAQEKEGDKEAIAEASLNSIKLQILKAKSQVTEESSTEFGLIIDGKSLTYAMDKNLVNSFMDLAMRCATVICCRSSPKQKAVITRLVKSVTGRTTLAIGDGANDVGMLQEADIGVGISGVEGMQAVMASDFAIAQFRFLERLLLVHGHWCYRRISMMICYFFYKNITFGFTLFWFEAYTSFSGQPAYNDWYMSCYNVFFTSLPVIALGVFDQDVSARLCLKHPLLYQEGVRNILFNWPRILGWMFNGLLSSIIIYFLTTNSITGPAFRKDGQVADYSVLGVTMYTCVVWTVNCQMALSINYFTWIQHLFIWGSIAFWYIFLVVYGSIPPTLSTTAYKVLVEACAPSILYWLTTLLVVIAALLPLLSYRAFQIRFRPMEHDRIQIQRRRPESTEYDATQRRLSESSEARTSSELPSEVRIRIDNLKASSKRKN

>GaALA10

MTGGRRRKQHFSRIHAFSCGKASFKGDHSLIGGPGFSRVVYCNDPECFEASLRNYAGNSVRSTKYTLATFFPKSLFEQFRRVANFYFLICAILSFTPLSPYSAVSNVLPLVVVIGATMGKEAVEDWRRKKQDTEVNNRKVKMHQSDGIFEPTKWMDLKVGDIVKVEKDEFFPADLILLSSSYEEAICYVETMNLDGETNLKLKQASDVTSSLHDDASFQDFKATIRCEDPNANLYSFVGSLELGDEQYPLSPQQLLLRDSKLRNTDYIFGVVIFTGRDTKVIQNSTEPPSKRSKIEKRMDNIVYFLFAVLVGLSIIGSIFFGIETREDLENGKMRRWYLRPDDTTIYYNPKRAAVAAILQFLTALMLYSYLIPISLYVSIEIVKVLQSIFINQDLHMYHEETDKPAHARTSNLNEELGQVDTILSDKTGTLTCNSMEFIKCSIAGTSYGHGITEVERALAWRKGSPLAREVPEINDQVEEFKKEKPSVKGFNFVDERIMNGNWLKEPHADVIQKFLRLLAICHTAIPEVDEETGRISYEAESPDEAAFVVAARELGFEFYERTQTSISLYEFDLSGKKVERSYKLLNILEFSSSRKRMSVILQNEEGKLLLLCKGADSVMFERLAKNGQEFAEQTKEHIEEYADAGLRTLVLAYREINEEEYVEFNEKFTEAKNIVSADREEMIEEVAESIERDLILLGATAVEDKLQNGVPECIDKLAQAGIKIWVLTGDKMETAINIGFACSLLRQGMKQIIINSDTPEDKALEKSGDKTAAAAAYKASVLQQIAEGRLLLTSSNENSEALALIVDGKSLTYALEDDVKDAFLELAIGCASVICCRSSPKQKALVTRLVKTKTRSTTLAIGDGANDVGMLQEADIGVGISGVEGMQAVMSSDIAIAQFRFLERLLLVHGHWCYRRISSMICYFFYKNIVFGFTLFFYEIYASFSGQAVYNDWFLSFYNVFFTSLPVIALGVFDQDVSSRLCLKFPLLYQEGIQNVLFSWLRIIAWAFNGVLSATVIFFFCIRAVQHQAFRKGGEVVGLEILGTAMYTCVVWVVNCQMALSVSYFTYIQHLFIWGSIILWYIFLMAYGAMDPSISTTAYKVFIESCAPAGMYWLLTLLVLISSLLPYFIYSAIQVRFFPSYHQMIQWIRSDGQSDDPEYCHMVRQRSLRPTTVGYTARLEAKSRSSRKGGEDHQ

>GaALA11

MSGGRRRKVLMSRIYGIACGKASFKEDHSQIGGPGFSRVVYCNEPNSLEAGTRNYSDNYVSTTKYTIATFLPKSLFEQFRRVANFFFLVTGILSFTAIAPYSALSAIVPLIIVIGATMIKEGVEDWRRQQQDIEVNNRKVKVHQGDGNFHHTEWKNLRVGDIVKVEKDEFFPTDLILLASSYEDAVCYVETMNLDGETNLKLKQALEVTSSLHDDYNFRDFKAIVKCEDPNANLYSFVGTMEFEEQQHPLSPQQLLLRDSKLRNTDYIYGAVVFTGHDTKVMQNATDPPSKRSKIEKTMDRVIYLMFFIVFIMGFIGSIFFGIATENDYEGGRIKRRWYLRPDNAEIFFDPERAPVAAIYHFLTALLLYSYFIPISLYVSIEIVKVLQSIFINQDSHMYYEEADKPAHARTSNLNEELGQVDTILSDKTGTLTCNSMEFIKCSIAGTAYGRGVTEVERAIYRKKGSPVVHEPNGLNHIEDSADVNPAIKGFNFKDERIMNGNWVNEPRADVIQKFFRLLAICHTAIPEVDEENGNISYEAESPDEAAFVIAARVLGFEFHNRTQTSISLHELDPVSGKRVNRLFKLLNVLEFDSSRKRMSVIVRDEEGKLLLLCKGADSVMFERLAKGGRDFEEDTREHMNEYADAGLRTLVLAYRELSQNEYEVFNEKMTEAKNSVSADRETLIDEVAEMIERDLILLGATAVEDKLQNGVPDCIDKLAQAGIKLWVLTGDKMETAINIGYACSLLRQGMKQIIINIDTPEIQSLEKTGDKDAVIKASRKSVMEQIVSGKAQVSALSAISEAFALIIDGKSLAYALEDDMKNSFLELAIGCASVICCRSSPKQKALVTRLVKLGTGKTTLAIGDGANDVGMLQEADIGIGISGVEGMQAVMSSDVAIAQFRYLERLLLVHGHWCYRRISSMICYFFYKNIAFGFTIFLYEAYTSFSAQPAYNDWYLTLFNVFFSSLPVIAMGVFDQDVSAWFCLKFPLLYQEGVQNVLFSWRRIVSWMFNGFYSAIIIFFFCSRALEQQAFNDEGKTASKDILGGTMYTCIVWVVNLQMALSISYFTLIQHIVIWGTIAFWYVFQLAYGALPASFSTDAYRVFVEALAPAPSYWFITLFVVIATLTPYFLYSAIQMRFFPMYHEMIQWIRHEGLSDDPLYCEMVRQRSIRPTTVGFTARRAASRRQ

>GaP5

MSRFHVGGKVVDKVDLLRKKHAAWRLDVWPFAMLYLLWLTMVVPSIDFVDAAIVLGGLAVTHILVLLFTTWSVDFKCFVQYSKVNNIRLADVCKVTPAKFCGSKEIASSSSAKDVEEIYFDFRKQCFIYSKEEDTFCKLPYPTKETFGYYLKCSGHGSDAKVLAATEKWGRNVFEYPQPTFQKLMKEHCMEPFFVFQVFCVGLWCLDEYWYYSLFTLFMLFMFESTMAKSRLKTLSELRRVRVDSQTLMVHRCGKWVKLSGTDLLPGDVVSIGRSSGQNEEDKSVPADMLILAGSAIVNEAILTGESTPQWKVSIAGRGIEEKLSAKRDKNHMLFGGTKILQHTADKSFPLRTPDGGCLAVVLRTGFETSQGKLMRTILFSTERVTANSWESGLFILFLVVFAIIAAGYVLKKGLEDPTRSKYKLFLSCSLIITSVIPPELPMELSIAVNTSLIALARRGIFCTEPFRIPFAGKVDICCFDKTGTLTSDDMEFSGVVGLNDSSELESDMTKVPSRTVEILASCHALVFVDNKLVGDPLEKAALKGIDWSYKSDEKAIPKKGSGNPVQIVQRHHFASHLKRMAVVVRVQEDFFSFVKGAPETIQDRLIDLPPTYVETYKKYTRQGSRVLALAYKSLPDMTVSEARSMERDTVECGLTFAGFAVFNCPIRADSSTVLSELKNSSHDLVMITGDQALTACHVAGQVNIVSKPALILVAVKNSKGYEWVSPDETERIPYSENEVEALSETHDLCIGGDCFEMLQQTSAVLRVIPFVKVFARVAPEQKELIMTTFKTVGRLTLMCGDGTNDVGALKQAHVGVALLNAVPPTKSESSSGTSKDENTKSLKSKSKPTVEATGNSEASSKGKVVPRSESSNNATSNRHLNAAEKHRQKLKKMMDELNEEGDGRSAPIVKLGDASMASPFTAKHASVAPTTDIIRQGRSTLVTTLQMFKILGLNCLATAYVLSVMYLDGVKLGDVQATISGVFTAAFFLFISHARPLPTLSAARPHPNIFCSYVFLSLMGQFAMHLLFLISSVKEAEKHMPEECIEPESEFHPNLVNTVSYMVSMMLQVATFAVNYMGHPFNQSIPENKPFLYALGAAAGFFVVITSDIFRDLNDWLSLVPLPVGLRDKLLIWALLMFLCCYAWERLLRWAFPGKIPAWRKRQRVAAASSEKKLV

>GhAHA1

MAETSVGLEDIKNETIDLDSKVLKFLGFMWNPLSWVMEIAAIMAIALANG

GGKPPDWQDFIGIVALLLINSTISFIEENNAGNAAAALMAGLAPKTKVLR

DGKWSEKEAAILVPGDIISIKLGDIVPADARLLEGDPLKIDQSALTGESL

PVNKNAGDEVFSGSTVKQGELEAVVIATGVHTFFGKAAHLVDSTNNVGHF

QQVLTAIGNFCICSIGVGMLIEIVVMYPIQQRRYRDGIDNLLVLLIGGIP

IAMPTVLSVTMAIGSHRLSQQGAITKRMTAIEEMAGMDVLCSDKTGTLTL

NKLTVDKSLVEVFTNDVDKDMVILLAARASRVENQDAIDACIVGMLGDPK

EARAGITEVHFFPFNPVDKRTAMTYIEADGSWHRVSKGAPEQIIDLCNLR

DDVKRRTHDIIANFADRGLRSLAVARQTVKEKNKDAQGEAWEFVSILPLF

DPPRHDSAETIRRALNLGVNVKMITGDQLAIGKETGRRLGMGTNMYPSSA

LLGQNKGDTIDTIGVDELIEKADGFAGVFPEHKYEIVKRLQQRNHICGMT

GDGVNDAPALKKADIGIAVDDATDAARGASDIVLTEPGLSVIVSAVLTSR

AIFQRMKNYTIYAVSITIRIVLGFMLLALIWKFDFSPFMVLIIAILNDGT

IMTISKDRVKPSPMPDSWKLKEIFAMGIVLGTYLACMTVVFFWAANDSNF

FQDKFGVRSIRHNQDELTAAIYLQVSIVSQALIFVTRSRSWSFIERPGLL

LVVAFILAQLIATLLAVYANWGFARIKGIGWGWAGVIWLYSLVFYVPLDV

LKFLIRYSLSGKAWHNLLQNKTAFTTKKDYGKGEREAQWAMAQRTLHGLT

PPEMTQLYNEETNYRELSEIAEQARKRAEVARLRELHTLKGHVESVVKLK

GLDIDTIQQHYTV

>GhAHA3

MADKSISLEEIKNETVDLVSISFPRIQPERIPVEEVFQQLKCTRNGLTSE

EGQKRLQIFGPNKLEEKKAKENKVLKFLGFMWNPFSWVMEFAAIMAIALA

NGGGKPPDWQDFIGIVSLLFINSTISFIEENNAGNAAAALMAGLAPKTKV

LRDGKWNEQEAAILVPGDIISIKLGDIVPADARLLEGDSLKIDQSALTGE

SLPVNKHSGDEVFSGSTVKQGEIEAVVIATGVHTFFGKAAHLVDSTNNVG

HFQQVLTAIGNFCICSIGVGMLIEIVVMYPIQRRKYRDGIDNLLVLLIGG

IPIAMPTVLSVTMAIGSHRLSQQGAITKRMTAIEEMAGMDVLCSDKTGTL

TLNKLTVDKSMIEVFMDNIDKEMVLLLAARASRVENQDAIDACIVGMLGD

PKEARAGVTEVHFFPFNPVDKRTAMTYIEADGSWHRVSKGAPEQIIELCN

LRNDAKRRAHDIITKFADRGLRSLAVAKRKVPEKTKDGQGDPWQFVGLLP

LFDPPRHDSAETIRRALSLGVNVKMITGDQLAIGKETGCRLGMGTNMYPS

SVLFGENKGDALDTIGVDELIEKADGFAGVFPEHKYEIVKRLQQRKHICG

MTGDGVNDAPALKKADTGIAVDDATDAARSASDIDRVKPSPMPDSWKLKE

IFCTGIVLGTYLACMTVVFFWAANDSNFFSDKFGVRSIRHNQDELTAAVY

LQVSIVSQALIFVTRSRSWSFIERPGFLLVIAFILAQLVATVIAVYANWG

FARIKGIGWGWPGVIWIYSVVFYFPLDVIKFLIRYAMSGKAWNNLLQNKT

AFTTKKDYGKGEREAQWALAQRTLHGLTPPEMTEKSNYIELSEIAEQARK

RAEVARLRELHTLKGHVESVVKLKGLDIDTIQQHYTV

>GhAHA2

MAETSVGLEDIKNETIDLESKVLKFLGFMWNPLSWVMEIAAIMAIALANG

GGKPPDWQDFIGIVALLLINSTISFIEENNAGNAAAALMAGLAPKTKVLR

DGKWSEQEAAILVPGDIISIKLGDIVPADARLLEGDPLKIDQSALTGESL

PVNKNAGDEVFSGSTVKQGELEAIVIATGVHTFFGKAAHLVDSTNNVGHF

QQVLTAIGNFCICSIGVGMLIEIVVMYPIQQRRYRDGIDNLLVLLIGGIP

IAMPTVLSVTMAIGSHRLSQQGAITKRMTAIEEMAGMDVLCSDKTGTLTL

NKLTVDKSLVEVFTNDVDKDMVILLAARASRVENQDAIDACIVGMLGDPK

EARAGITEVHFFPFNPVDKRTAMTYIEADGSWHRVSKGAPEQIIDLCNLR

DDVKRRAHDIIANFADRGLRSLAVARQTVKEKNKDAQGEAWEFVGILPLF

DPPRHDSAETICRALNLGVNVKMITGDQLAIGKETGRRLGMGTNMYPSSA

LLGQNKGDTIDTIGVDELIEKADGFAGVFPEHKYEIVKRLQQRNHICGMT

GDGVNDAPALKKADIGIAVDDATDAARGASDIVLTEPGLSVIVSAVLTSR

AIFQRMKNYTIYAVSITIRIVLGFMLIALIWKFDFSPFMVLIIAILNDGT

IMTISKDRVKPSPMPDSWKLKEIFATGIVLGTYLACMTVVFFWAASDSNF

FQDKFGVRSIRHNQDELTAAIYLQVSIVSQALIFVTRSRSWSFIERPGLL

LVVAFILAQLIATLLAVYANWGFARIKGIGWGWAGVIWLYSLVFYVPLDV

LKFLIRYSLSGKAWDNLLQSKTAFTTKKDYGKGEREAQWAMAQRTLHGLT

PPEMTQLYNEETNYRELSEIAEQARKRAEVARLRELHTLKGHVESVVKLK

GLDIDTIQQHYTV

>GhAHA12

MGAISLEEIKNETVDLEKIPIEEVFEQLKCSREGLSSEEGANRLQIFGPN

KLEEKKESKILKFLGFMWNPLSWVMEAAAIMAIALANGDGKPPDWQDFVG

IVCLLVINSTISFIEENNAGNAAAALMAGLAPKTKVLRDGKWSEQEAAIL

VPGDIISIKLGDIIPADARLLEGDPLKIDQSALTGESLPVTKNPGDEVFS

GSTCKQGEIEAVVIATGVHTFFGKAAHLVDSTNNVGHFQKVLTAIGNFCI

CSIAIGMLVEIIVMYPIQHRKYRDGIDNLLVLLIGGIPIAMPTVLSVTMA

IGSHRLSQQGAITKRMTAIEEMAGMDVLCSDKTGTLTLNKLSVDRNLIEV

FVKDVDKEHVVLLAARASRTENQDAIDAAIVGMLADPKEARAGIREVHFL

PFNPVDKRTALTYIDSNGNWHRASKGAPEQILTLCNAKEDVKKKVHAIID

KFADRGLRSLGVARQLVPEKSKDGAGTPWQFVGLLPLFDPPRHDSAETIR

RALNLGVNVKMITGDQLAIAKETGRRLGMGTNMYPSASLLGQDKDASIAA

LPVEELIEKADGFAGVFPEHKYEIVRKLQERKHICGMTGDGVNDAPALKK

ADIGIAVADATDAARSASDIVLTEPGLSVIISAVLTSRAIFQRMKNYTIY

AVSITIRIVFGFMLIALIWKFDFSPFMVLIIAILNDGTIMTISKDRVKPS

PLPDSWKLKEIFATGIVLGGYLALMTVVFFWIMHDTDFFSEKFGVRSLRK

RDDQMMGALYLQVSIVSQALIFVTRSRSWSYFERPGLLLVTAFIIAQLVA

TVIAVYANWGFARIQGIGWGWAGVIWLYSVVFYIPLDLMKFAIRYILSGK

AWLNLLENKTAFTTKKDYGKEEREAQWALAQRTLHGLQPPEASNLFNDKS

SYRELSEIAEQAKRRAEVARLRELHTLKGHVESVVKLKGLDIDTIQQHYT

V

>GhAHA13

MGAISLEEIKNETVDLEKIPIEEVFEQLKCSREGLSSEEGANRLQIFGPN

KLEEKKESKILKFLGFMWNPLSWVMEAAAIMAIALANGDGKPPDWQDFVG

IVCLLVINSTISFIEENNAGNAAAALMAGLAPKTKVLRDGKWSEQEAAIL

VPGDIISIKLGDIIPADARLLEGDPLKIDQSALTGESLPVTKNPGDEVFS

GSTCKQGEIEAVVIATGVHTFFGKAAHLVDSTNNVGHFQKVLTAIGNFCI

CSIAIGMLVEIIVMYPIQHRKYRDGIDNLLVLLIGGIPIAMPTVLSVTMA

IGSHRLSQQGAITKRMTAIEEMAGMDVLCSDKTGTLTLNKLSVDRNLIEV

FVKDVDKEHVVLLAARASRTENQDAIDAAIVGMLADPKEARAGIREVHFL

PFNPVDKRTALTYSDSNGNWHRASKGAPEQILTLCNAKEDVKKKVHAIID

KFADRGLRSLGVARQQVPEKSKDGAGTPWQFVGLLPLFDPPRHDSAETIR

RALNLGVNVKMITGDQLAIAKETGRRLGMGTNMYPSASLLGQDKDASIAA

LPVEELIEKADGFAGVFPEHKYEIVRKLQERKHICGMTGDGVNDAPALKK

ADIGIAVADATDAARSASDIVLTEPGLSVIISAVLTSRAIFQRMKNYTIY

AVSITIRIVFGFMLIALIWKFDFSPFMVLIIAILNDGTIMTISKDRVKPS

PLPDSWKLKEIFATGIVLGCYLALMTVVFFWIMHDTDFFSEKFGVRSLRK

KDDQMMGALYLQVSIVSQALIFVTRSRSWSYFERPGLLLVTAFIIAQLAA

TVIAVYANWGFARIQGIGWGWAGVIWLYSVVFYIPLDLMKFAIRYILSGK

AWLNLLENKTAFTTKKDYGKEEREAQWALAQRTLHGLQPPEASNLFNDKS

SYRELSEIAEQAKRRAEVARLRELHTLKGHVESVVKLKGLDIDTIQQHYT

V

>GhAHA28

MVTVAPGLGSGDIPVSLNHHERGARQTVAGSREFQPRGDRFGEFNERLPL

EEVFEQLRTSRAGLTSEDAEVRVHIFGQNKLEEKPENKFLKFLSFMWNPL

SWVMEAAAVMAIVLANGGGEGPDWQDFVGIICLLILNSTISFIEENNAGN

AAAALMARLAPKTKVLRDGQWQERDAAILVPGDIISIKLGDIIPADARLL

EGDPLKIDQATLTGESLPVTKRTGDEVFSGSTCKHGEIEAVVIATGVHSF

FGKAAHLVDSTEVVGHFQQVLTSIGNFCICSIAVGMVLEIIVMFPIQHRS

YRDGINNLLVLLIGGIPIAMPTVLSVTLAIGSHRLSQQGAITKRMTAIEE

MAGMDVLCSDKTGTLTLNRLTVDRNLVEVFSKNMDKDLIVLLAARASRLE

NQDAIDAAIINMLADPKEARANIKEVHFLPFNPVDKRTAITYIDSDGNWY

RASKGAPEQILNICLEKDLIAGRVHAIIDKFAERGLRSLGVAFQEIPERT

KESPGGPWTFCGLLPLFDPPRHDSAETIRRALNLGVDVKMITGDQLAIAK

ETGRRLGMGTNMYPSSSLLGREKDENEALPVDELIEKADGFAGVFPEHKY

EIVKILQEKKHVVGMTGDGVNDAPALKKADIGIAVADATDAARSAADIVL

TEPGLSVIISAVLTSRAIFQRMKNYTIYAVSITIRIVLGFVLLALIWEYD

FPPFMVLIIAILNDGTIMTISKDRVRPSPTPDSWKLNEIFATGVVIGTYL

ALVTVLFYWIVIDTDFFETHFNVRSISDDIEQISSAVYLQVSIISQALIF

VTRSRSWSFVERPGVLLMCAFVVAQLVATLIAVYAHISFADISGIGWGWA

GVIWLYSLVFYVPLDIIKFTVRYALSGEAWNLLFDRKTAFTSKKDYGKDD

RAAQWILSQRSLQGLMAADLDFNGRRSRSSLIADHARRRAEIARLGELHT

LRGHVESVMRLKNLDLNAIKSAHTV

>GhAHA18

MGNDKGISLEEIKNESVDLERIPIEEVFEQLKCTRGGLTSEEGANRLQVF

GPNKLEEKKESKFLKFLGFMWNPLSWVMEAAALMAIALANGDGRPPDWQD

FVGIIALLLINSSISFIEENNAGNAAAALMANLAPKTKVLRDGRWSEQDA

AILVPGDIITIKLGDIVPADARLLEGDPLKIDQSALTGESLPVTKNPSDE

VFSGSTCKQGEIEAVVIATGVHTFFGKAAHLVDSTNQVGHFQKVLTAIGN

FCICSIAVGIIIEIIVMYPIQHRKYRDGIDNLLVLLIGGIPIAMPTVLSV

TMAIGSHRLSQQGAITKRMTAIEEMAGMDVLCSDKTGTLTLNKLTVDRNL

IEVFAKGVEKEHVILYAARASRTENQDAIDAAIVGMLADPKEARAGIREI

HFLPFNPVDKRTALTYIDAAGNWHRASKGAPEQIITLCNCKEDVKKKVHA

VIDKFAERGLRSLAVARQDVPEKSKDGPGAPWQLIGLLPLFDPPRHDSAE

TIRRALNLGVNVKMITGDQLAIAKETGRRLGMGTNMYPSSSLLGQDKDAS

IASLPVDELIEKADGFAGVFPEHKYEIVKRLQERKHICGMTGDGVNDAPA

LKKADIGIAVADATDAARSASDIVLTEPGLSVIISAVLTSRAIFQRMKNY

TIYAVSITIRIVFGFMFIALIWKFDFAPFMVLIIAILNDGTIMTISKDRV

KPSPQPDSWKLKEIFSTGIVLGGYLALMTVVFFWVMKDTDFFPNMFNVRS

LADSPEEMMAALYLQVSIVSQALIFVTRSRSWSYVERPGLLLLTAFVIAQ

LVATLIAVYANWGFARIKGMGWGWAGVIWLYSVVTYIPLDLIKFAIRYVL

SGKAWDNLLDNKTAFTTKKDYGKEEREAQWAAAQRTLHGLQPPETSNLFN

EKSSYRELSEIAEQAKRRAEVARLRELNTLKGHVESVVKLKGLDIDTIQQ

HYTV

>GhAHA14

MAGISLEEIKNETVDLEKIPIEEVFEQLKCTREGLSSDEGVNRIQIFGPN

KLEEKKESKILKFLGFMWNPLSWVMEAAAIMAIALANGEGKPPDWQDFVG

IVCLLVINSTISFIEENNAGNAAAALMAGLAPKTKVLRDGKWTEQEAAIL

VPGDIISIKLGDIIPADARLLEGDPLKVDQSALTGESLPVTKNPGDEVFS

GSTCKQGEIEAIVIATGVHTFFGKAAHLVDSTNQVGHFQKVLTAIGNFCI

CSIAIGMLVEIVVMYPIQHRKYRDGIDNLLVLLIGGIPIAMPTVLSVTMA

IGSHRLSQQGAITKRMTAIEEMAGMDVLCSDKTGTLTLNKLSVDKNLIEV

FVKDADKEHVVLLAARASRTENQDAIDAAIVGMLADPKEARAGIREVHFL

PFNPVDKRTALTYIDSNGNWHRASKGAPEQILALCNAKEDLKKRVHSIID

KFADRGLRSLAVARQQVPEKTKESAGTPWQFVGLLPLFDPPRHDSAETIR

QALHLGVNVKMITGDQLAIAKETGRRLGMGTNMYPSASLLGQDKDASIAA

LPVEELIEKADGFAGVFPEHKYEIVKKLQERKHICGMTGDGVNDAPALKK

ADIGIAVADATDAARSASDIVLTEPGLSVIISAVLTSRAIFQRMKNYTIY

AVSITIRIVFGFLFIALIWKFDFSPFMVLIIAILNDGTIMTISKDRVKPS

PLPDSWKLKEIFATGIVLGGYLALMTVIFFWAMHDTDFFSDKFGVRSLRE

REHEMMGALYLQVSIVSQALIFVTRSRSWSYAERPGLLLVTAFIIAQLVA

TLIAVYANWGFARIKGIGWGWAGVIWLYSIVFYVPLDIMKFAIRYILSGK

AWLNLLENKTAFTTKKDYGNEEREAQWALAQRTLHGLQPPETSNLFNDRN

SYRELSEIAEQAKRRAEVARLRELHTLKGHVESVVKLKGLDIDTIQQHYT

V

>GhAHA15

MAGISLEEIKNETVDLEKIPIEEVFEQLKCTREGLSSDEGVNRLQIFGPN

KLEEKKESKILKFLGFMWNPLSWVMEAAAIMAIALANGDGKPPDWQDFVG

IVCLLVINSTISFIEENNAGNAAAALMAGLAPKTKVLRDGKWTEQEAAIL

VPGDIISIKLGDIIPADARLLEGDPLKVDQSALTGESLPVTKNPGDEVFS

GSTCKQGEIEAIVIATGVHTFFGKAAHLVDSTNQVGHFQKVLTAIGNFCI

CSIAIGMLVEIVVMYPIQHRKYRDGIDNLLVLLIGGIPIAMPTVLSVTMA

IGSHRLSQQGAITKRMTAIEEMAGMDVLCSDKTGTLTLNKLSVDKNLIEV

FVKDADKEHVVLLAARASRTENQDAIDAAIVGMLADPKEARAGIREVHFL

PFNPVDKRTALTYIDSNGNWHRASKGAPEQILALCNAKEDLKKRVHSIID

KFAERGLRSLAVSRQQVPEKTKESAGTPWQFVGLLPLFDPPRHDSAETIR

QALHLAVKVKMITGDQLAIAKETGRRLGMGTNMYPSASLLGQDKDASIAA

LPVEELIEKADGFAGVFPEHKYEIVRKLQERKHICGMTGDGVNDAPALKK

ADIGIAVADATDAARSASDIVLTEPGLSVIISAVLTSRAIFQRMKNYTIY

AVSITIRIVFGFLFIALIWKFDFSPFMVLIIAILNDGTIMTISKDRVKPS

PLPDSWKLKEIFATGIVLGGYLALMTVIFFWVMHDTDFFSDKFGVRSLRE

RDHEMMGALYLQVSIVSQALIFVTRSRSWSYAERPGLLLVTAFIIAQLVA

TLIAVYANWGFARIKGIGWGWAGVIWLYSIVFYVPLDIMKFAIRYILSGK

AWLNLLENKTAFTTKKDYGKEEREAQWALAQRTLHGLQPPETSNLFNDKN

SYRELSEIAEQAKRRAEASRASYTHILGLHILVESLSYLLTQPPSQTAFT

TKKDYGKEEREAQWALAQRTLHGLQPPETSNLFNDKNSYRELSEIAEQAK

RRAEVARLRELHTLKGHVESVVKLKGLDIDTIQQHYTV

>GhAHA29

MWNPLSWVMEAAAVMAIVLANGGGEGPDWQDFVGIICLLIINSTISFIEE

NNAGNAAAALMARLAPKTKVLRDGQWQERDAAILVPGDIISIKLGDIIPA

DARLLEGDPLKIDQATLTGESLPVTKRTGDEVFSGSTCKHGEIEAVVIAT

GVHSFFGKAAHLVDSTEVVGHFQQVLTSIGNFCICSIAVGMVLEIIVMFP

IQLRSYRDGINNLLVLLIGGIPIAMPTVLSVTLAIGSHRLSQQGAITKRM

TAIEEMAGMDVLCSDKTGTLTLNRLTVDRNLVEVFSKNMDKDLVVLLAAR

ASRLENQDAIDAAIINMLADPKEARANIKEVHFLPFNPVDKRTAITYIDS

DGNWYRASKGAPEQILNLCLEKDEIAGRVHAIIDKFAERGLRSLGVAFQE

VPERTKKSPGGPWTFCGLLPLFDPPRHDSAETIRRALNLGVDVKMITGDQ

LAIAKETGRRLGMGTNMYPSSSLLGREKDESEALPADELIEKADGFAGVF

PEHKYEIVKILQEKKHVVGMTGDGVNDAPALKKADIGIAVADATDAARSA

ADIVLTEPGLSVIISAVLTSRAIFQRMKNYTIYAVSITIRIVLGFVLLAL

IWEYDFPPFMVLIIAILNDGTIMTISKDRVKPSPTPDSWKLNEIFATGVV

IGTYLALVTVLFYWIVIDTDFFETHFNVRSISDNTEQISSAVYLQVSIIS

QALIFVTRSRSWSFVERPGVLLMCAFVVAQLVATLIAVYAHISFADISGI

GWGWAGVIWLYSLVFYVPLDIIKFTVRYALSGEAWNLLFDRKTAFTSKKD

YGKDDRAAQWILSQRSLQGLMAADLDFNGRRSRTSLIADQARRRAEIARL

GELHTLRGHVESVMRLKNLDLNAIKSAHTV

>GhAHA4

MADKSISLEEIKNETVDLERIPVEEVFQQLKCTRNGLGSEEGQKRLQIFG

PNKLEEKKAKENKLLKFLGFMWNPLSWVMEFAAIMAIALANGGGKPPDWQ

DFIGIVSLLFINSTISFIEENNAGNAAAALMAGLAPKTKVLRDGKWNEQE

AAILVPGDIISIKLGDIVPADARLLEGDALKIDQSALTGESLPVNKHSGD

EVFSGSTVKQGEIEAVVIATGVHTFFGKAAHLVDSTNNVGHFQQVLTAIG

NFCICSIGVGMLIEIVVMYPIQRRKYRDGIDNLLVLLIGGIPIAMPTVLS

VTMAIGSHRLSQQGAITKRMTAIEEMAGMDVLCSDKTGTLTLNKLTVDKS

MIEVFMDNIDKEMVLLLAARASRVENQDAIDACIVGMLGDPKEARAGVTE

VHFFPFNPVDKRTAMTYIEADGSWHRASKGAPEQIIELCNLRNDAKRRAH

DIITKFADRGLRSLAVAKQKVPEKTKDGQGDPWQFVGLLPLFDPPRHDSA

ETIRRALSLGVNVKMITGDQLAIGKETGRRLGMGTNMYPSSVLLGENKGD

ALDTIGVDELIEKADGFAGVFPEHKYEIVKRLQQRKHICGMTGDGVNDAP

ALKKADIGIAVDDATDAARSASDIVLTEPGLSVIVSAVLTSRAIFQRMKN

YTIYAVSITIRIVLGFMLLALIWKFDFSPFMVLIIAILNDGTIMTISKDR

VKPSPMPDSWKLKEIFGTGIVLGTYLACMTVVFFWAANDSNFFSDKFGVR

SIRHNQDELTAAVYLQVSIVSQALIFVTRSRSWSFIERPGFLLVIAFILA

QLVATVIAVYANWGFARIKGIGWGWAGVIWIYSVVFYFPLDVIKFLIRYA

MSGKAWNSLLQNKTAFTTKKDYGKGEREAQWALAQRTLHGLTPPEITEKS

NYIELSEIAEQARKRAEVARLRELHTLKGHVESVVKLKGLDIDTIQQHYT

V

>GhAHA19

MGNDRGISLEEIKNESVDLERIPIEEVFEQLKCTRGGLTSEEGANRLQVF

GPNKLEEKKESKFLKFLGFMWNPLSWVMEAAALMAIALANGDGRPPDWQD

FVGIIALLLINSSISFIEENNAGNAAAALMANLAPKTKVLRDGRWSEQDA

AILVPGDIITIKLGDIVPADARLLEGDPLKIDQSALTGESLPVTKNPSDE

VFSGSTCKQGEIEAVVIATGVHTFFGKAAHLVDSTNQVGHFQKVLTAIGN

FCICSIAVGIIIEMIVMYPIQHRKYRDGIDNLLVLLIGGIPIAMPTVLSV

TMAIGSHRLSQQGAITKRMTAIEEMAGMDVLCSDKTGTLTLNKLTVDRNL

IEVFAKGVEKEHVILYAARASRTENQDAIDAAIVGMLADPKEARAGIREI

HFLPFNPVDKRTALTYIDAAGNWHRASKGAPEQIITLCNCKEDVKKKVHA

VIDKFAERGLRSLAVARQEVPEKSKDGPGAPWQLIGLLPLFDPPRHDSAE

TIRRALNLGVNVKMITGDQLAIAKETGRRLGMGTNMYPSSSLLGQDKDAS

IASLPVDELIEKADGFAGVFPEHKYEIVKRLQERKHICGMTGDGVNDAPA

LKKADIGIAVADATDAARSASDIVLTEPGLSVIISAVLTSRAIFQRMKNY

TIYAVSITIRIVFGFMFIALIWKFDFAPFMVLIIAILNDGTIMTISKDRV

KPSPQPDSWKLKEIFSTGIVLGGYLALMTVVFFWAMKDADFFPNMFNVRS

LADSPEEMMAALYLQVSIVSQALIFVTRSRSWSYVERPGLLLLTAFVIAQ

LVATLIAVYANWGFARIKGMGWGWAGVIWLYSVVTYIPLDLIKFAIRYVL

SGKAWDNLLENKTAFTTKKDYGKEEREAQWAAAQRTLHGLQPPETSNLFN

EKSSYRELSEIAEQAKRRAEVARLRELNTLKGHVESVVKLKGLDIDTIQQ

HYTV

>GhAHA20

MGGEKGISLEEIKNESVDLERIPIEEVFEQLKCTREGLSTEEGNNRLQVF

GPNKLEEKKESKVLKFLGFMWNPLSWVMEAAAIMAIALANGDGRPPDWQD

FVGIIVLLVINSTISFIEENNAGNAAAALMANLAPKTKVLRDGRWSEQEA

AILVPGDIITIKLGDIVPADARLLEGDPLKIDQSALTGESLPVTKNPSDE

VFSGSTCKQGEIEAVVIATGVHTFFGKAAHLVDSTNQVGHFQKVLTAIGN

FCICSIAVGIIVELIVMYPIQHRKYRDGIDNMLVLLIGGIPIAMPTVLSV

TMAIGSHRLSQQGAITKRMTAIEEMAGMDVLCSDKTGTLTLNKLTVDRNL

IEVFTKGVEKEHVILYAARASRTENQDAIDAAIVGMLADPKEARAGVREI

HFLPFNPVDKRTALTYIDNDGNWHRASKGAPEQIIDLCKCKDDVRKKVHS

VIDKFAERGLRSLAVARQEIPEKTKESPGSPWQFIGLLPLFDPPRHDSAE

TIRRALNLGVNVKMITGDQLAIAKETGRRLGMGTNMYPSSSLLGQDKDAS

IAALPIDELIEKADGFAGVFPEHKYEIVKRLQERKHICGMTGDGVNDAPA

LKKADIGIAVADATDAARSASDIVLTEPGLSVIISAVLTSRAIFQRMKNY

TIYAVSITIRIVFGFMFIALIWKFDFAPFMVLIIAILNDGTIMTISKDRV

KPSPQPDSWKLKEIFSTGIVLGGYFALMTVIFFWAMKDTNFFSNTFNVRS

LRHSIDGEREMMAALYLQVSIVSQALIFVTRSRSWSYFERPGLLLVSAFL

VAQLVATLIAVYADWGFARIKGMGWGWAGVIWLYSVVTFIPLDFIKFAIR

YVLSGKAWDNLLENKTAFTTKKDYGKEEREAQWAAAQRTLHGLQPPETTS

IFNERSSYRELSEIAEQAKRRAEVARLRELNTLKGHVESVVKLKGLDIDN

IQQHYTV

>GhAHA21

MGGEKGISLEEIKNESVDLERIPIEEVFEQLKCTREGLSTEEGNNRLQVF

GLNKLEEKKESKVLKFLGFMWNPLSWVMEAAAIMAIALANGDGRPPDWQD

FVGIIVLLVINSTISFIEENNAGNAAAALMANLAPKTKVLRDGRWSEQEA

AILVPGDIITIKLGDIVPADARLLEGDPLKIDQSALTGESLPVTKNPSDE

VFSGSTCKQGEIEAVVIATGVHTFFGKAAHLVDSTNQVGHFQKVLTAIGN

FCICSIAVGIIVELIVMYPIQHRKYRDGIDNMLVLLIGGIPIAMPTVLSV

TMAIGSHRLSQQGAITKRMTAIEEMAGMDVLCSDKTGTLTLNKLTVDRNL

IEVFTKGVEKEHVILYAARASRTENQDAIDAAIVGMLADPKEARAGVREI

HFLPFNPVDKRTALTYIDSDGNWHRASKGAPEQIIDLCKCKDDVRKKVHS

VIDKFAERGLRSLAVARQEIPEKTKESPGSPWQFIGLLPLFDPPRHDSAE

TIRRALNLGVNVKMITGDQLAIAKETGRRLGMGTNMYPSSSLLGQDKDAS

IAALPIDELIEKADGFAGVFPEHKYEIVKRLQERKHICGMTGDGVNDAPA

LKKADIGIAVADATDAARSASDIVLTEPGLSVIISAVLTSRAIFQRMKNY

TIYAVSITIRIVFGFMFIALIWKFDFAPFMVLIIAILNDGTIMTISKDRV

KPSPQPDSWKLKEIFSTGIVLGGYLALMTVIFFWAMKDTNFFSNTFNVRS

LRHNNIDGEKEMMAALYLQVSIVSQALIFVTRSRSWSYFERPGLLLVSAF

LVAQLVATLIAVYADWGFARIKGMGWGWAGVIWLYSVVTYIPLDLIKFAI

RYVLSGKAWDNLLENKTAFTTKKDYGKEEREAQWAAAQRTLHGLQPPETT

SIFNERSSYRELSEIAEQAKRRAEVARLRELNTLKGHVESVVKLKGLDID

NIQQHYTV

>GhAHA16

MAGNKGISLDEIKNESVDLERIPIEEVFEQLKCTRAGLTTQEGNNRLQVF

GPNKLEEKKESKFLKFLGFMWNPLSWVMEAAAVMAIALANGGGRPPDWQD

FVGIIVLLVINSTISFIEENNAGNAAAALMANLAPKTKVLRDGRWSEQEA

AILVPGDIITIKLGDIVPADARLLEGDPLKIDQSALTGESLPVTKNPSDE

VFSGSTCKQGEIEAVVIATGVHTFFGKAAHLVDSTNQVGHFQQVLTAIGN

FCICSIAVGIVIELIVMYPIQRRKYRDGIDNLLVLLIGGIPIAMPTVLSV

TMAIGSHRLSQQGAITKRMTAIEEMAGMDVLCSDKTGTLTLNKLTVDRNL

IEVFAKGVEKEQVLLYAARASRTENQDAIDAAIVGMLADPKEARAGIREV

HFLPFNPVDKRTALTYIDSDGKWHRASKGAPEQIITLCNCKADVRNKVHA

VIDKFAERGLRSLAVARQEVPEKTKESPGAPWQLVGLLPLFDPPRHDSAE

TITRALNLGVNVKMITGDQLAIAKETGRRLGMGTNMYPSSSLLGQDKDSS

VSALPVDELIEKADGFAGVFPGDGVNDAPALKKADIGIAVADATDAARSA

SDIVLTEPGLSVIISAVLTSRAIFQRMKNYTIYAVSITIRIVFGFLFIAL

IWKFDFAPFMVLIIAILNDGTIMTISKDRVKPSPQPDSWKLKEIFSTGIV

LGGYLALMTVLFFWAMKDTDFFSDKFHVRSLRDRPEQMMAALYLQVSIVS

QALIFVTRSRSWSFVERPGLLLVTAFIAAQLVATLIAVYANWRFAKIQGM

GWGWAAVIWLYSLVTYIPLDLIKFAIRYVLSGKAWDNLLENKTAFTTKKD

YGKEEREAQWAAAQRTLHGLQPPETSNVFNERSSYRELSEIAEQAKRRAE

VARLRELNTLKGHVESVVKLKGLDIDTIQQHYTV

>GhAHA17

MAGNKGISLDEIKNESVDLERIPIEEVFEQLKCTRAGLTTQEGNNRLQVF

GPNKLEEKKESKFLKFLGFMWNPLSWVMEAAAIMAIALANGGGRPPDWQD

FVGIIVLLVINSTISFIEENNAGNAAAALMANLAPKTKVLRDGRWSEQEA

AILVPGDIITIKLGDIVPADARLLEGDPLKIDQSALTGESLPVTKNPSDE

VFSGSTCKQGEIEAVVIATGVHTFFGKAAHLVDSTNQVGHFQQVLTAIGN

FCICSIAVGIVIELIVMYPIQRRKYRDGIDNLLVLLIGGIPIAMPTVLSV

TMAIGSHRLSQQGAITKRMTAIEEMAGMDVLCSDKTGTLTLNKLTVDRNL

IEVFAKGVEKEQVLLYAARASRTENQDAIDAAIVGMLADPKEARAGIREV

HFLPFNPVDKRTAITYIDCDGKWHRASKGAPEQIITLCNCKADVRNKVHA

VIDKFAERGLRSLAVARQEVPEKTKESPGAPWQLVGLLPLFDPPRHDSAE

TITRALNLGVNVKMITGDQLAIAKETGRRLGMGTNMYPSSSLLGQDKDSS

VSALPVDELIEKADGFAGVFPGDGVNDAPALKKADIGIAVADATDAARSA

SDIVLTEPGLSVIISAVLTSRAIFQRMKNYTIYAVSITIRIVFGFLFIAL

IWKFDFAPFMVLIIAILNDGTIMTISKDRVKPSPQPDSWKLKEIFSTGIV

LGGYLALMTVLFFWAMKDTDFFSDKFHVRSLRDRPEQMMAALYLQVSIVS

QALIFVTRSRSWSFVERPGLLLVTAFIAAQLVATLIAVYANWRFAKIQGM

GWGWAAVIWLYSLVTYIPLDLIKFAIRYVLSGKAWDNLLENKTAFTTKKD

YGKEEREAQWAAAQRTLHGLQPPETSNVFNERSSYRELSEIAEQAKRRAE

VARLRELNTLKGHVESVVKLKGLDIDTIQQHYTV

>GhAHA24

MASNGDISLEQIKNETVDLERIPVEEVFQQLKCTREGLTSAEGEKRLQIF

GPNKLEEKEESKFLKFLGFMWNPLSWVMEAAAIMAIVLANGGGKPPDWPD

FIGIVSLLFINSTISFIEENNAGNAAAALMAGLAPKTKVLRDGKWGEQEA

ATLVPGDVINVKLGDIIPADARLLEGDALKIDQSALTGESLPVTKNPGDE

VFSGSTCKQGEIEAVVIATGVHTFFGKAAHLVDNTNNVGHFQKVLVAIGN

FCICSIAVGMLIEILVMYLIQHRRYRDGIDNLLVLLIGGIPIAMPTVLSV

TMAIGSHSLSQQGAITKRMTAIEEMAGMDVLCSDKTGTLTLNKLPVDKSL

IEVFVSDMDADALVLIAARASRVENQDAIDASIVGMLGDPKEARAGITEV

HFLPFNPVDKRTAITYIDRNGEWHRCSKGAPEQIIELCELTGGLRQKAHH

VIDSFADRGLRSLGVARQTIPEKKKECAGGPWEFVGLLPLFDPPRHDSAE

TIRQALDLGVNVKMITGDQLAIGKETSHRLGMGTNMYPSSSLLGQCKDEA

IATIPVDELIETADGFAGVVPEHKYEIVKRLQELNHICGMTGDGVNDAPA

LKKADIGIAVADATDAARSASDIVLTEPGLSVIVSAVLTSRAIFQRMKNY

TIYAVSITIRIVMGFMLIALIWEFDFSPFMVLIIAILNDGTIMTISKDRV

KPSPTPDSWKLKEIFITGVVLGAYMAIVSVIFFWLVHDTLFFTEKFGVKP

INENANELTSALYLQVSIISQALIFVTRSRSWSFVELPGLLLLGAFLAAQ

LVATCIAVYANWGFARIEGIGWEWAGVIWLFSVITYIPLDILKFIIRYSL

TGKAWDNLLQKKAAFTTKKNYGKETREAQWAADQRTLHGLSPPETSWTNE

KDHHELSEIAEQAKKRAEIARLRELHTLKGHVESVVKLKGIDIDTIQQHY

TV

>GhAHA5

MVDIDDTLQEIKNENVDLLKCTKEGLTTEEGLKRLQIFGPNKLEEKSESK

VLKFLGFMWNPLSWVMEIAAIMAIALANGGGKPPDWQDFVGITVLLIINS

AISFIEENNAGNAAAALMAGLAPKTKVLRDGKWCEQEAAILVPGDIISIK

LGDIIPADARLLEGDALKVDQSALTGESLPVNKNPGDGVYSGSTVKQGEL

EAVVIATGVHTFFGKAAHLVDSTNNVGHFQKVLTAIGNFCICSIAVGMLV

EIVVMYPIQRRKYRDGIDNLLVLLIGGIPIAMPTVLSVTMAIGSHRLSQQ

GAITKRMTAIEEMAGMDVLCSDKTGTLTLNKLTVDKSMVEVFTKDVDKEM

LLLLAARASRVENQDAIDACIVGMLGDPKEAREGITEVHFFPFNPVDKRT

AMTYIESDGSWHRVSKGAPEQIISLCDLRDDVKKKAHDIIDKFADRGLRS

LGVARQTVPEKTKDSPGSPWEFVGLLPLFDPPRHDSAETIRRALHLGVNV

KMITGDQLAIGKETGRRLGMGTNMYPSSALLGHNKDEKVDTIDVDELIEK

ADGFAGVFPEHKYEIVKRLQERNHICGMTGDGVNDAPALKKADIGIAVDD

ATDAARSASDIVLTEPGLSVIVSAVLTSRAIFQRMKNYTIYAVSITIRIV

LGFMLLALIWKFDFSPFMVLIIAILNDGTIMTISKDRVKPSPMPDSWKLK

EIFATGIVLGTYLACMTVVFFWAANDSDFFSDKFGVKSIRYSQDELTAAV

YLQVSIVSQALIFVTRSRSWSFIERPGLLLVVAFILAQLVATVLAVYANW

GFARIKGIGWPWAGVIWLYSIVFYIPLDVLKFLIRYALSGKAWDNLLQNK

TAFTTKKDYGRGEREAQWATAQRTLHGLQAPGAEEILNEKSSYRELSEIA

EQAKKRAEVARLRELHTLKGHVDSVVKLKGLDIETINQNYTV

>GhAHA11

MASNGDISLEQIKNETVDLERIPVEEVFQQLKCTREGLTSAEGEKRLQIF

GPNKLEEKEESKFLKFLGFMWNPLSWVMEAAAIMAIVLANGGGKPPDWPD

FIGIVSLLFINSTISFIEENNAGNAAAALMAGLAPKTKVLRDGKWGEQEA

ATLVPGDVINVKLGDIIPADARLLEGDALKIDQSALTGESLPVTKNPGDE

VFSGSTCKQGEIEAVVIATGVHTFFGKAAHLVDNTNNVGHFQKVLVAIGN

FCICSIAVGMLIEILVMYLIQHRRYRDGIDNLLVLLIGGIPIAMPTVLSV

TMAIGSHSLSQQGAITKRMTAIEEMAGMDVLCSDKTGTLTLNKLPVDKSL

IEVFVSDMDADALVLIAARASRVENQDAIDASIVGMLGDPKEARAGITEV

HFLPFNPVDKRTAITYIDRNGEWHRCSKGAPEQIIELCELTGGLRQKAHH

VIDSFADRGLRSLGVARQTIPEKKKECAGGPWEFVGLLPLFDPPRHDSAE

TIRQALDLGVNVKMITGDQLAIGKETSHRLGMGTNMYPSSSLLGQCKDEA

IATIPVDELIETADGFAGVVPEHKYEIVKRLQELNHICGMTGDGVNDAPA

LKKADIGIAVADATDAARSASDIVLTEPGLSVIVSAVLTSRAIFQRMKNY

TIYAVSITIRIVMGFMLIALIWEFDFSPFMVLIIAILNDGTIMTISKDRV

KPSPTPDSWKLKEIFITGVVLGAYMAIVSVIFFWLVHDTLFFTEKFGVKP

INENANELTSALYLQVATCIAVYANWGFARIEGIGWEWAGVIWLFSVITY

IPLDILKFIIRYSLTGKAWDNLLQKKAAFTTKKNYGKETREAQWAADQRT

LHGLSPPETSWTNEKDHHELSEIAEQAKKRAEIARLRELHTLKGHVESVV

KLKGIDIDTIQQHYTV

>GhAHA6

MVDIDDTLQEIKNENVDLERIPVEEVFLQLKCTKEGLTTEEGLKRLQIFG

PNKLEEKSESKVLKFLGFMWNPLSWVMEIAAIMAIALANGGGKPPDWQDF

VGITVLLIINSTISFIEENNAGNAAAALMAGLAPKTKVLRDGKWCEQEAA

ILVPGDIISIKLGDIIPADARLLEGDALKVDQSALTGESLPVNKNPGDGV

YSGSTVKQGELEAVVIATGVHTFFGKAAHLVDSTNNVGHFQKVLTAIGNF

CICSIAVGMLVEIVVMYPIQRRKYRDGIDNLLVLLIGGIPIAMPTVLSVT

MAIGSHRLSQQGAITKRMTAIEEMAGMDVLCSDKTGTLTLNKLTVDKSMV

EVFTKDVDKEMLLLLAARASRVENQDAIDACIVGMLGDPKEAREGITEVH

FFPFNPVDKRTAMTYIESDGSWHRVSKGAPEQIISLCDLRDDVKKKAHDI

IDKFADRGLRSLGVARQTVPEKTKDSPGSPWEFVGLLPLFDPPRHDSAET

IRRALHLGVNVKMITGDQLAIGKETGRRLGMGTNMYPSSALLGHNKDEKV

ETIDVDELIEKADGFAGVFPEHKYEIVKRLQERNHICGMTGDGVNDAPAL

KKADIGIAVDDATDAARSASDIVLTEPGLSVIVSAVLTSRAIFQRMKNYT

IYAVSITIRIVLGFMLLALIWKFDFSPFMVLIIAILNDGTIMTISKDRVK

PSPMPDSWKLNEIFATGIVLGTYLACMTVVFFWAANDSDFFSDKFRVKSI

RYSQDELTAAVYLQVSIVSQALIFVTRSRSWSFIERPGLLLVVAFILAQL

VATVLAVYANWGFARIKGIGWPWAGVIWLYSIVFYIPLDVLKFLIRYALS

GKAWDNLLQNKTAFTTKKDYGREEREAQWATAQRTLHGLQAPGAEEILNE

KSSYRELSEIAEQAKKRAEVARLRELHTLKGHVDSVVKLKGLDIETINQN

YTV

>GhECA11

MGKGGENYGKREFVNSKPTDPDVFPAWAKDIHECEKHYDVSQKSGLSTAE

VENRRRIYGNNELEKHEGQSIWSLIMEQFNDTLVRILLLAAVVSFVLAWY

DGDEGGEMEITAFVEPLVIFLILIVNAIVGVWQENNAEKALEALKEIQSE

QATVIRDGIKIPSLPAKELVPGDIVELKVGDKVPADMRVLELVSSTLRVE

QGSLTGESEAVNKTNKSVSEDADIQGKRSMVFAGTTVVNGNCFCLVTQIG

METEIGKVHTQIHVASQSEEDTPLKKKLNEFGEVLTMIIGVICIFVWLIN

VKYFLSWEYIDGWPRNFKFSFEKCTYYFEIAVALAVAAIPEGLPAVITTC

LALGTRKMAQKNALVRKLPSVETLGCTTVICSDKTGTLTTNQMAVAKLLA

MGGHASSLRNFRVDGTTYNPSDGKIHGWPSGGMDANLETIAKISAICNDA

GVTHSDNKYVAHGMPTEAAIKVLVEKMGLPKGLYSGGAAGNDVLRCCQWW

NEYEHRIATLEFDRDRKSMGVIVKSKSGRRSLLVKGAVENLLERSSKMQL

LDGSVVPLDQNSRILVSNALQDMSSGALRCLGFAYKDELPEFETYDGSDD

HPAHALLLDPSNYPSIESNLTFVGLVGLRDPPRQEVHQAIEDCKAAGIRV

MVITGDNKNTAEAICREIGVFGPMEDISSKSLTGKEFMALSDKKAHLRQS

GGLLFSRAEPRHKQEIVRLLKEDGEVVAMTGDGVNDAPALKLADIGIAMG

IAGTEVAKEASDMVLADDNFSTIVSAVGEGRSIYDNMKAFIRYMISSNIG

EVASIFLTAALGIPEGLIPVQLLWVNLVTDGPPATALGFNPPDKDIMKKP

PRRSDDSLITAWILFRYLVIGLYVGVATVGVFIIWYTHGSFLGIDLSGDG

HTLVTYPQLANWAQCSSWKNFTVSPFTAGNQVFSFENNPCDYFQGGKVKA

MTLSLSVLVAIEMFNSLNALSEDGSLLTMPPWVNPWLLLAMSVSFGLHFL

ILYVPFLAQVFGIVPLSFNEWLLVLAVAFPVILIDEVLKFVGRLGRRMRS

SSQRPLKPKTE

>GhECA12

MGKGGENYGKREFVNSKPTDPDVFPAWAKDIRECEKHYDVSRKSGLSTAE

VENRRKIYGNNELEKHEGQSIWSLIMEQFNDTLVRILLLAAVVSFVLAWY

DGDEGGEMEITAFVEPLVIFLILIVNAIVGVWQENNAEKALEALKEIQSE

QATVIRDGIKIPSLPAKELVPGDIVELKVGDKVPADMRVLELVSSTLRVE

QGSLTGESEAVNKTNKSVSEDADIQGKRSMVFAGTTVVNGNCFCLVTQIG

METEIGKVHTQIHVASQSEEDTPLKKKLNEFGEVLTMIIGVICIFVWLIN

VKYFLSWEYIDGWPRNFKFSFEKCTYYFEIAVALAVAAIPEGLPAVITTC

LALGTRKMAQKNALVRKLPSVETLGCTTVICSDKTGTLTTNQMAVAKLVA

MGGHASSLRSFRVDGTTYNPSDGKIHDWPSGGMDANLETIAKISAICNDA

GVTHSDNKYVAHGMPTEAAIKVLVEKMGLPKGSYSGGAAGDDVLRCCQWW

NEYEHRIATLEFDRDRKSMGVIVKSKSGRRSLLVKGAVENLLERSSKMQL

LDGSVVPLDQNSRILVSNALQDMSSAALRCLGFAYKDELPEFETYDGSDD

HPAHALLLDPSNYSSIESNLTFVGLVGLRDPPRQEVHQAIEDCKAAGIRV

MVITGDNKNTAEAICHEIGVFGPMEDISSKSLTGKEFMALSDKKAHLRQS

GGLLFSRAEPRHKQEIVRLLKEDGEVVAMTGDGVNDAPALKLADIGIAMG

IAGTEVAKEASDMVLADDNFSTIVSAVGEGRSIYDNMKAFIRYMISSNIG

EVASIFLTAALGIPEGLIPVQLLWVNLVTDGPPATALGFNPPDKDIMKKP

PRRSDDSLITAWILFRYLVIGLYVGVATVGVFIIWYTHSSFLCIDLSGDG

HTLVTYPQLANWAQCSSWKNFTVSPFTAGNQVFYFENNPCDYFQGGKVKA

MTLSLSVLVAIEMFNSLNALSEDGSLLTMPPWVNPWLLLAMSVSFGLHFL

ILYVPFLAQVFGIVPLSLNAWLLVLAVAFPVILIDEVLKFVGRLGRRMRS

SSQRPLKPKTE

>GhECA7

MGRGEEDHGKREKISAASSKVENFPAWAKDVKQCEENFQTNRELGLSSAE

VEKRREIYGWNELEKHEGTSIFQLILEQFNDTLVRILLLAAIISFVLAWL

DGDEGGEKEITAFVEPLVIFLILIVNAIVGIWQESNAEKALEALKEIQSE

QANVVRDGKKVSNLHAKELVPGDIVELRVGDKVPADMRVLTLISSTVRVE

QGSLTGESEAVSKTAKVVPENTDIQGKKCMVFAGTTVVNGNCICMVTQIG

MNTEIGKVHSQIHEASQSDDDTPLKKKLNEFGEVLTMIIGVICALVWLIN

VKYFLSWEYVDGWPSNFKFSFEKCTYYFEIAVALAVAAIPEGLPAVITTC

LALGTRKMAQKNALVRKLPSVETLGCTTVICSDKTGTLTTNQMAVSKLIA

MGSRPGTLRAFDVEGTTYNPFDGKIRGWAAGEMDANLQMIAKICAVCNDA

GVEQSGSHYVATGMPTEAALKVLVEKMGLPEENGSSSGHGDHQRCCQAWN

KLEQRIATLEFDRDRKSMGVIVNSSTGQKALLVKGAVENLLERSSFMQLR

DGSIIELDQYSKDLILQSLHEMSTDALRCLGFAYKEEPFEFTTYNGDEDH

PAHQLLLNPSNYSSIESKLIFAGLVGLRHLDKPMKDPPRKEVRQAIEDCK

AAGIRVMVITGDNKNTAEAICREIGVFGSSEDISSRSLTGKDFMDHPNQK

NHLRQSGGLLFSRAEPRHKQEIVRLLKEDGEVVAMTGDGVNDAPALKLAD

IGVAMGIAGTEVAKEASDMVLADDNFSTIVAAVGEGRSIYNNMKAFIRYM

ISSNIGEVASIFLTSALGIPEGMIPVQLLWVNLVTDGPPATALGFNPPDK

DIMKKPPRRSDDSLITAWILFRYLVIGLYVGIATVGVFIIWYTHHSFLGI

DLSGDGHSLVTYSQLANWGKCDSWEGFSVSPFTAGSQVFKFDYDPCDYFH

SGKIKASTLS

>GhAHA7

MASDGDISLEQIKNESVDLENIPVEEVFKLLKCTKEGLTTAEGETRLSIF

GHNKLEEKKDNKVLKFLGFMWNPLSWVMEAAAIVAIVLANGGGKPPDWPD

FIGIVTLLLINSTISFIEENSAGNAAAALMAGLAPKTKVLRDGKWSEQEA

SILVPGDIISVKLGDIIPADARLLEGDPLKIDQAALTGESLPVTKNPGDL

VFSGSTCKQGELDAIVIATGIHSFFGKAAHLVDNTNNVGHFQQVLTSIGN

FCIFSIGIGMVIEIIVMYPIQHRNYRDGIDNLLVLLIGGIPIAMPTVLSV

TMAIGSHRLSEQGAITKRMTAIEEMAGMDILCSDKTGTLTLNKLTIDKNL

VEVFINDMDVDTLVLLAARASRVENQDAIDACIVGMLSDPKQAREGITEL

HFLPFNPVDKRTAITYTDRKGEWHRCSKGAPEEIIDLCGLTGGLRKKALS

IIDGYANRGLRSLGVARQTIPEKTKESPGGPWEFVGLLPLFDPPRHDSAE

TIKRALELGISVKMITGDQLAIGKETGRRLGMGTDMYPSSALLGECGDED

IAAIPIDDLIEKADGFAGVFPEHKYEIVGRLQERKHICGMTGDGVNDAPA

LKKADIGIAVADSTDAARGASDIVLTEPGLSVIVSAVLTSRSIFQRMKNY

TIYAVSITIRIVLGFMLVALIWKFDFSPFMVLVIAILNDGTIMTISKDRV

RPSPKPDTWKLDEIFATGVVLGTYMAIVTVFFFWLVHDTEFFTRTFGVKP

INDNEDALTTALYLQVSTVSQALIFVTRSRSWSFIELPGPLLLIAFILAQ

LVATLLAVYANWGFARIQGIGWEWAGAIWVFSIITYIPLDVLKFIIRYAL

TGDAWGDVVQSKTAFKGESEVQLTTNYRPLQGVSSPPETWSNDELVPIV

>GhAHA10/GhTT13

MENKDETLDAVLKEAVDLENVPLDEVLQTLRCNRDGLTTEAAEQRLSIFG

YNKLEEKQESKILKFLGFMWNPLSWVMEAAAIMAIALANGGGKPPDWQDF

VGIITLLTINSTISFIEENNAGNAAAALMARLAPKAKVFRDGKWSEEEAS

ILVPGDIISIKLGDIIPADARLLDGDPLKIDQSSLTGESLPVTKGPGDSI

YSGSTCKQGEIEAVVIATGVHTFFGKAAHLVDSTNQQGHFQKVLTAIGNF

CICSIAVGMITEIIVMYPIQDRDYRPGIDNLLVLLIGGIPIAMTTVLSVT

MAIGSHRLSLQGAITKRMTAIEEMAAMDVLCSDKTGTLTLNKLSVDKNLI

EIFAKGVDPDTVVLMAARAARLENQDAIDAAIVGMLADPKEARAGIQEVH

FLPFNPTDKRTALTYIDNQGRMHRVSKGAPEQILNLAHNKSELERRVHAV

IDKFAERGLRSLAVAYQEVPEGRKESSGGPWQFIGLMPLFDPPRHDSADT

IRRALNLGVNVKMITGDQLAIAKETGRRLGMGTNMYPSSALLGQNKEESI

AALPVDELIEKADGFAGVFPEHKYEIVKRLQARKHICGMTGDGVNDAPAL

KKADIGIAVADATDAARSASDIVLTEPGLSVIISAVLTSRAIFQRMKNYT

IYAVSITIRIVLGFMLLALIWKFDFPPFMVLIIAILNDGTIMTISKDRVK

PSPLPDSWKLAEIFATGIILGGYLAMMTVIFFWAAYKTDFFPSTFGVKSL

QKTDRKDIKMLASAVYLQVSIISQALIFVTRARSWSFLERPGFLLVLAFV

VAQLIATLIAVYANWGFAAIEGIGWGWAGVIWLYNLIFYIPLDFIKFFIR

YALSGKAWDLVIEQRIAFTRKKDFGKEERELKWAHAQRTLHGLQPPDTKM

FGDRTSYNELNQMAEEAKRRAEIARLRELTTLKGHVESVVRLKGLDIDTI

QQAYTV

>GhECA9

MGRGGENYGKRENAGAASSKQETFPAWARDVKQCEEKYQVNRELGLPSAE

VEKRRQIYGLNELLKHEPTSIFQLLLEQFNDTLVRVLLAAAIISFVLAWY

DGEGRGEKEITAFVEPLVIFLILIVNAIVGIWQESSAEKALEALKEIQSE

HADVIRDGKKVSSLPAKELVPGDIVELRVGDKVPADMRVLSLISSTVRVE

QGSLTGESEAVSKTVKVVPENSDIQGKKCMVFAGTTMVNGNCICLVTEIG

MNTEIGKVHSQIHEASQSDTDTPLKKKLNEFGEVLTLIIGMICILVWLIN

VKYFLSWEYVDGWPTNFKFSFEKCTYYFEIAVALAVAAIPEGLPAVITTC

LALGTRKMAQKNALVRKLPSVETLGCTTVICSDKTGTLTTNQMAVSKLVA

IGSRSGTLRSFDVEGTTYDPFDGKILGWPVDGMDANLEMIAKISAVCNDA

GVEQSGRHYVAIGMPTEAALKVLVEKMELPEKYASSSAPGDPRRCCQVWN

KMVQRIATLEFDRDRKSMGVIINSSSGNKSLLVKGAVENLLERSSFIQLL

DGSTVELDKYSKDLILQVLREMSTDALRCLGFAYKEELPEFATYNSDEDH

PAHQLLLNPSNYSSIESNLIFVGLVGLRDPPRKEVRQAIEDCKAAGIRVM

VITGDNKNTAEAICREIGVFGYREDITSRSLTGKEFMDHPDQRNHLRQNG

GLLFSRAEPRHKQEIVRLLKQDGEVVAMTGDGVNDAPALKLADIGVAMGI

TGTEVAKEASDMVLADDNFSTIVAAVGEGRSIYDNMKAFIRYMISSNIGE

VASIFLTAALGIPEGMIPVQLLWVNLVTDGPPATALGFNPQDTDIMKKPP

RRSNDSLITAWILFRYLVIGSYVGLATVGVFIIWYTHNTFMGIDLSGDGH

SLVTYSQLSNWDKCPSWVNFTASPFTAGPQVFNFDTNPCDYFRSGKIKAS

TLSLSVLVSIEMFNSLNALSEDGSLLTMPPWVNPWLLLAMSISFGLHFLI

LYVPFLAQVFGIVPLSHNEWLLVLAVAFPVILIDEVLKCIGRCTTGPRYS

PATISIKHKAE

>GhECA10

MGRGGENYGKRENAGAASFKQETFPAWARDVKQCEEKYQVNRELGLSSAE

VEKRRQIYGLNELLKHKPTSIFQLLLEQFNDTLVRILLAAAIISFVLAWY

DGEEGGEKEITAFVEPLVIFLILIVNAFVGIWQESSAEKALEALKEIQSE

HADVIRDGKKVSSLPAKELVPGDIVELRVGDKVPADMRVLNMISSTVRVE

QGSLTGESEAVSKTVKVVPENSDIQGKKCMVFAGTTMVNGNCICLVTEIG

MNTEIGKVHSQIHEASQSDTDTPLKKKLNEFGEVLTMIIGMICILVWLIN

VKYFLSWEYVDGWPTNFKFSFEKCTYYFEIAVALAVAAIPEGLPAVITTC

LALGTRKMAQKNALVRKLPSVETLGCTTVICSDKTGTLTTNQMAVSKLVA

IGSRSGTLRSFDVEGTTYDPFDGKILGWPVDGMDSNLEMIAKISAVCNDA

GVEQSGRHYVAIGMPTEAALKVLVEKMELPEKYASSSAPGDPQPIQGCCH

VWNKMVQRIATLEFDRDRKSMGVILPSSSGNKSLLVKVLREMSTDALRCL

GFAYKEELPEFATYNGDEDHPAHQLLLNPSNYSSIESNLIFVGLVGLRDP

PRKEVRQAIEDCKAAGIRVMVITGDNKNTAEAICREIGVFGYREDITSRS

LTGKEFMDHPDQRNHLRQNGGLLFSRAEPRHKQEIVRLLKQDGEVVAMTG

DGVNDAPALKLADIGVAMGITGTEVAKEASDMVLADDNFSTIVAAVGEGR

SIYDNMKAFIRYMISSNIGEVASIFLTADLGIPEGMIPVQLLWVNVVSDG

RPATALGFNPQDTDIMKKPPRRSNDSLITAWILFRYLVIGSYVGLATVGV

FIIWYTHNTFMGIDLSGDGHSLVTYSQLSNWDKCPSWVNFTASPFTAGPQ

VFNFDTNPCDYFRSGKIKASTLSLSVLVSIEMFNSLNALSEDGSLLTMPP

WVNPWLLLAMSISFGLHFLILYVPFLAQVFGIVPLSLNEWLLVLAVAFPV

ILIDEVLKCIGRCTTGPRYSPATKSIKHKAE

>GhAHA26

MGDKNEVLEAVLKETVDLENIPIEEVFENLRCSREGLTTEAAEERLTIFG

HNKLEEKKESKFLKFLGFMWNPLSWVMEAAAIMAIALANGGGKPPDWQDF

VGIITLLVINSTISFIEENNAGNAAAALMARLAPKAKVLRDGRWNEQDAA

ILVPGDIISIKLGDIIPXXXXXXXXXXXPADARLLEGDPLKIDQSALTGE

SLPVTKGPGDGIYSGSTCKQGEIEAVVIATGVHTFFGKAAHLVDTTNQVG

HFQKVLTAIGNFCICSIAVGMVIEIIVMYPIQDRDYRPGIDNLLVLLIGG

IPIAMPTVLSVTMAIGSHRLSQQGAITKRMTAIEEMAGMDVLCSDKTGTL

TLNKLTVDKNLIEVFAKGVDADTVVLMAARASRTENQDAIDSAIVGMLAD

PKEARAGIREVHFLPFNPTDKRTALTYIDSDGKMHRVSKGAPEQILHLAH

NKTDIERRVHAVIDKFAERGLRSLAVAYQEVPDGRKESPGGPWQFIGLMP

LFDPPRHDSAETIRRALNLGVNVKMITGDQLAIGKETGRRLGMGTNMYPS

SALLGQDKDESIAALPVDELIEKADGFAGVFPEHKYEIVKRLQARKHICG

MTGDGVNDAPALKKADIGIAVADATDAARSASDIVLTEPGLSVIISAVLT

SRAIFQRMKNYTIYAVSITIRIVLGFMLLALIWKFDFPPFMVLIIAILND

GTIMTISKDRVKPSPLPDSWKLAEIFTTGIVLGSYLAVMTVIFFWAAYKT

NFFPRVFGVATLEKTAHDDIKKLASAVYLQVSIISQALIFVTRSRSWSFV

ERPGLLLLAAFVIAQLIATLIAVYANWSFAAIEGIGWGWAGVIWLYNIIF

YIPLDFIKFFIRYALSGRAWDLVIEQRIAFTRQKDFGKEQRELQWAHAQR

TLHGLQAPDTKMFTERTHFTELNQMAEEAKRRAEIARLRELHTLKGHVES

VVRLKNLDIDTIQQAYTV

>GhECA8

MGRGGEDYGKREKVSAASSKVENFPAWAKDVKQCEENFQMNRELGLSSAE

VEKMREIYGWNELEKHEGTSIFQLILEQFNDTLVRILLLAAIISFVLAWL

DGDEGGEKEITAFVEPLVIFLILIVNAIVGIWQESNAEKALEALKEIQSE

QANVVRDGKKVSNLPAKELVPGDIVELRVGDKVPADMRVLTLISSTVRVE

QGSLTGESEAVSKTAKVVPENTDIQGKKCMVFAGTTVVNGNCICMVTQIG

MNTEIGKVHSQIHEASQSDDDTPLKKKLNEFGEALTMIIGVVCALVWLIN

VKYFLSWEYVDGWASNFKFSFEKCTYYFEIAVALAVAAIPEGLPAVITTC

LALGTRKMAQKNALVRKLPSVETLGCTTVICSDKTGTLTTNQMAVSKLIA

IGSRPAILRAFDVEGTTYNPFDGKIRGWAAGEMDANLQMIAKICAVCNDA

GVEQSGSHYVATGMPTEAALKVLVEKMGLPEENGSSSDHWDHQRCCQAWN

KMEQRIVTLEFDRDRKSMGVIVNSSTGQKALLVKGAVENLLERSSFMQLR

DGSIIELDQYSKDLILQSLHEMSTDALRCLGFAYKEEPFEFTTYNGDEDH

PAHQLLLNPSNYSSIESKLIFSGLVGLRDPPRKEVRQAIEDCKAAGIRVM

VITGDNKNTAEAICREIGVFGSSEDISSRSLTGKDFMDHPNQKNHLRQSG

GLLFSRAEPRHKQEIVRLLKEDGEVVAMTGDGVNDAPALKLADIGVAMGI

AGTEVAKEASDMVLADDNFSTIVAAVGEGRSIYNNMKAFIRYMISSNIGE

VASIFLTSALGIPEGMIPVQLLWVNLVTDGPPATALGFNPPDKDIMMKPP

RRSDDSLITAWILFRYLVIGLYVGIATVGVFIIWYTHHSFLGIDLSGDGH

SLVTYSRLANWAKCDSWEGFSVSPFTAGSQVFKFDSDPCDYFQSGKIKAS

TLCLSVLXXXKSSPRLFPSLSW

>GhAHA9

MASNGDISLEQIKNETVDLERIPVEEVFQQLKCTREGLTSEEGEKRLQIF

GPNKLEEKEESKFLKFLGFMWNPLSWVMEAAAIMAIVLANGGGKPPDWPD

FIGIVSLLFINSTISFIEKNNAGNAAAALMAGLAPKTKVLRDGKWGEQEA

AILVPGDVINVKLGDIIPADARLLEGDALKFDQSALTGESLPVTKNPGDE

VFSGSTCKQGEIEAVVIATGVHTFFGKAAHLVDNTNNVGHFQKVLAAIGN

FCICSIAVGMLIEILVMYPIQHRRYRDGIDNLLVLLIGGIPIAMPTILSV

TMAIGSHSLSQQGAITKRMTAIEEMAGMDVLCSDKTGTLTLNKLTVDKSL

IEVFINDMDADTLVLIAAKASRVENQDAIDASIVGMLGDPKEARAGITEV

HFLPFNPVDKRTAITYIDRNGEWHRCSKGAPEQVIELCELTGGLRQKAHH

VIDSFADRGLRSLGVARQTIPEKTKECAGGPWEFVGLLPLFDPPRHDSAE

TIRQTLDLGVNVKMITGDQLAIGKETGRRLGMGTNMYPSSSLLGQCKDEA

IATIPVDELIETTDGFIGVFPEHKYEIVKRLQELKHICGMTGDGVNDAPA

LKKADIGIAVADATDAARSASDIVLTKPGLSVIVSAVLTSRAIFQRMKNY

TIYAVSITIRIVMGFMLIALIWEFDFSPLMVLIIAILNDGTIMTISKDRV

KPSPTPDSWKLKEIFITGVVLGAYMAIVSVIFFWLVHDTLFFTDKFGVKP

INENANELTSALYLQVSIISQALIFVHGQGAGPLLNSLVSCFLEPSSQRS

WPHSQQRRIMNYGKEAREAQWAANQRTLHGLSPPETSWTNEKDHHELSEI

AEQAKKRAEIARLRKLHTLKGHVESVVKLKGIDIDTIQQHYSV

>GhHMA2

MSAGRADVDAISCQVPRHRSDVAAISRKMAHSGKEEFIIKEMVANKNLQK

SYFDVLGLCCSSEVPLIENILKSLEGVKEVSVIVPTRTVIVLHDNLLLSQ

LQIVKALNQARLEANVRAHGEIKYQKKWPSPFAVVCGLLLLLSFLKYVYH

PLQWLAVGAVVIGIYPVLFKGFAAITHFRIDINILILIAVAAIYFTVIGS

VAMKDYTEAGTIVFLFTIAEWLESRASHKASAVMSSLMSITPQKAVIAEN

GEEVDADEVKLNTLLAVKAGEAIPIDGIVVDGRCEVDEKSLTGESLPVTK

EKDSTVWAGTINLNGYISVKTTAVAKDCVVAKMAKLVEEAQNSKSNTQRF

IDKCAQFYTPAIIIVSAAIAVIPTALRVKNIHHWFHLALVVLVSACPCSL

ILSTPVASFCALTKAATSGLLVKGGDYLETLSKIRITAFDKTGTLTRGEF

IVTDFQPLSQDISLDTLLYWVSSIESKSSHPMAAALVDYGRSHSVEPNPE

TVEDYQNFPGEGIYGRVDGRDIYIGSKKISVRAHGTVPTLEGNMMKGKTI

GFVYSGATPAGIFSLSDACRTGVPEAVDELKSMRIKAAMLTGDNQAVAIH

VQEQLGNRLDVVHAELLPEDKARIIKEFKKEGATAMIGDGVNDAPALATA

DIGISMGISGSALATETGHVVLMSNDIRKIPKAIKLAKKAHRKVIENVIL

SISTKTAILALAFAGHPLVWAAVLADVGTCLLVICNSMLLLRGKHKDGRK

CCKSSVAAHTNKHGSKASHCDSFHKHQDASLDKKVQKACEPPTCCSERCA

SRCHSGLFKTDSSSNSRGSDKCEDSRRTHDGFVIREAKYCNQRSCHLVNH

KIEAQNLPRKCCSGHGSLDLGKEANALYGAKQCHQGHLHQYSSSTPKKEQ

QGTKNDHCHSTYCGENHVDIHGNNLTAFENLVEHRCLESLNQTGHLDSHE

PTHTAIDITMNPDEVHGCTSVEKRELGGCCKSYMKECCGKHGRFRPGLTE

IITE

>GhECA6

MEERAFPAWSWSVEHCLKEYDVRLDKGLSSYKVEKQREKYGWNELAKEKG

KPLWLLVLEQFDDMLVKILLVAAFISFLLAYMHGSESEESGFEAYVEPFV

IVLILFLNAIVGVWQETNAEKALEALKEMQCESGKVLRDGFLVPDLPARE

LVPGDIVELQAPADMRIAALKTSTLRLEQSALTGEAMPVLKGTSPIFQKE

CELQAKENIVFAGTTVVNGCCVCIVVCTGMNTEIGKIQRQIHEASLEESD

TPLKKKLDEFGSRLTTAIGIVCLIVWLINYKNFLSYDMVDGWPANFRFSF

EKCTYYFKIAVALAVAAIPEGLPAVITTSLALGTRKMAQKNAIVRKLPSV

ETLGCTTVICSDKTGTLTTNQMSVAEFFTLGGKTTTSRIFHVKGTTYDPK

DGGIVDWTCYNMDANLQVMAEICAVCNDAGIFCDGRLFRATGLPTEAALK

VLAEKMGVPDAKMRSKIRDSELVANYLIDRSTVKLGCCEWWIKRSKRVAT

LEFDRVRKSSSIIVREAAGQNRLLAKGAVESLLERSTHVQLADGSLAPMD

EPCRQLLLSRQTEMSSKGLRCLGLAYKEDLGEFSDYYSENHPAHKKLLDP

ACYCSIENDLVFVGVVGLRDPPRDEVHKAIEDCKGAGIRVMVITGDNKST

AEAICREIKLFSDGEDLRGKSFTGKEFMALSPSQQIETLSKPGGKVFSRA

EPRHKQEIVRMLKEMGEIVAMTGDGVNDAPALKLADIGIAMGITGTEVAK

EASDMVLADDNFSTIVSAVAEGRSIYNNMKAFIRHVD

>GhHMA4

MAANKKKLQKSYFDVLGLCCSSEVPLIENILKPLEGVKQVSVIVPTRTVI

VVHDNLLVSQLQIVKALNQARLEANVRAPGEIKYQKKWPSPFAMACGLLL

LISFFKYVYRPLQWVAVGAVVIGICPILLKGYAAITNFRLDINILMLIAV

IGSIAMKDYTEAATIVFLFTIAEWLESRASHKATAVMSSLMSMSPQKAVI

AESGEEVDVDEVKLNTVLAVKAGEVIPIDGIVVDGNCEVDEKTLTGESLP

VSKQKDSTVWAGTINLNGYISVKTTAVAEDCVVAKMAKLVEEAQNSKSTT

QRFIDKCAQFYTPAIIVVSVAIAVIPAAFRVHNLRHWFHLALVVLVSACP

CALILSTPVASFCALTKAATSGLLVKGGDYLEILSNIKITAFDKTGTLTR

GEFVVTNFRSLCQDISFNSLLYWVSSIESKSSHPMAAALIEYGRSHSIEP

KPETVEDYQNFPGEGIYGRIDGRDIYIGSRKVSVRAHGTAPNVEGNMMEG

KTIGYVFCGATPAGIFSLSDACRTGAAEAVNELKSMGIKTAMLTGDNQAA

AIHVQEQLGNSLDVIHADLLPQDKARIVEEFKKEGPTAMLGDGINDAPAL

ATADIGISMGISGSALATETGHVILMSNDIRKIPKAIRLARKAHRKVIQN

VILSISTKVAILALAFAGHPLVWAAVLADVGTCLLVIFNSMLLLHGTHKH

AGKCSKSSAASHKDKQGCNTSHCHSSHNHEHSSIDKKVQKACEPQKCSSR

SCASRCQPNPSNSDASSNSCGSNKCTESTGTREMKHCDQGSCNIVNHKIE

AHNLPSKCCSSHGKLGEKHCHHSSNQQGTKADHCHSTCGGNHTDRQTLGT

FVEHSCLESPKPEAHPYSNKCFTDYWESPHTAIDIPMNTSHETAQACMSV

EKREMGGCCKSYMKECCGKHGHFRSGLTEIVTE

>GhAHA22

MDTEKKAVSLEAINKEIVDLENIPIEEVLEKLKCTKEGLTTDEVQQRLEL

FGYNKLEEKKENKILKFLGFMWNPLSWVMEAAAVMAIALAHGGKKETDYH

DFVGILALLLINSTISFIEENNAGNAAAALMARLAPKAKVLRDGKWNEED

ASVLVPGDIISIKLGDPLKIDQSALTGESLPVTKHPGDGVYSGSTCKQGE

IEAVVIATGVHTFFGKAAHLVESTTHVGHFQQVLTSIGNFCICSIAIGML

AELIVIYGAQRRSYRTGIDNLLVILIGGIPIAMPTVLSVTMAIGSHRLSQ

QGAITKRMTAIEEMAGMDVLCSDKTGTLTLNKLTVDKNLIEVFVNNVDKD

TVILMAARASRLENQDAIDTAIVAMLADPKEARAGITEVHFLPFNPTDKR

TALTYVDEAGKMHRVSKGAPEQILNLAYNKSEIGKKVHSIIDKYAERGLR

SLAVARQEVPAGTKDSPGGPWEFVGLLPLFDPPRHDSAETIRRALDLGVS

VKMITGDQLAIGKETGRRLGMGTNMYPSSSLLGDHKNEEIGAFSIDELIE

NADGFAGVFPEHKFEIVKRLQAKKHIVGMTGDGVNDAPALKKADIGIAVA

DSTDAARSASDIVLTEPGLSVIISAVLTSRAIFQRMKNYTIYAVSITIRI

VLGFMLLTLIWRLNFPPFMVLIIAILNDGTIMTISKDRVKPSPLPDSWKL

KEIFATGIVLGSYLALMTLIFFWAAYETDFFPDKFGVRSLSKKSFDLTQE

SERRKVNALLSSAVYLQVSTISQALIFVTRSRGWSFTERPGLLLVTAFII

AQLVATVISAQATWSFAGIRAVGWGWCGVIWIYNILTYFLLDPIKFAVRY

ALSGKAWDLVLNQRTAFSTQKDFGKEAREAAWAAEQRTLHGLQSISDAKI

TEKHNFRDISVMAEEARRRAEIARLREIHTLKGKVESFAKLRGLDIDVNP

HYTV

>GhAHA25

MARLAPKAKVFRDGKWSEEEASILVPGDIISIKLGDIIPADARLLDGDPL

KIDQSSLTGESLPVTKGPGDSIYSGSTCKQGEIEAVVIATGVHTFFGKAA

HLVDSTNQQGHFQKVLTAIGNFCICSIAVGMITEIIVMYPIQDREYRPGI

DNLLVLLIGGIPIAMPTVLSVTMAIGSHRLSLQGAITKRMTAIEEMAGMD

VLCSDKTGTLTLNKLSVDKNLIEIFAKGVDPDTVVLMAARASRLENQDAI

DAAIVGMLADPKEARAGIQEVHFLPFNPTDKRTALTYIDNQGRMHRVSKG

APEQILNLAHNKSELERRVHAVIDKFAERGLRSLAVAYQEVPDGRKESSG

GPWQFIGLMPLFDPPRHDSADTIRRALNLGVNVKMITGDQLAIAKETGRR

LGMGTNMYPSSSLLGQNKEESIAALPVDELIEKADGFAGVFPEHKYEIVK

RLQARKHICGMTGDGVNDAPALKKADIGIAVADATDAARSASDIVLTEPG

LSVIISAVLTSRAIFQRMKNYTIYAVSITIRIVLGFMLLALIWKFDFPPF

MVLIIAILNDGTIMTISKDRVKPSPLPDSWKLAEIFATGIILGGYLAMMT

VIFFWAAYKTDFFPRTFGVKSLQKTDRKDIKMLASAVYLQVSIISQALIF

VTRARSWSFLERPGLLLVLAFVVAQLIATLIAVYANWGFAAIEGIGWGWA

GVIWLYNLIFYIPLDFIKFFIRYALSGKAWDLVIEQRIAFTRKKDFGKEE

RELKWAHAQRTLHGLQPPDTKMFGDRTSYNELNQMAEEAKRRAEIARLRE

LTTLKGHVESVVRLKGLDIDTIQQAYTV

>GhHMA5

MESALSVTVPTLALFSIPRALNRHFFYNNSSLIARCIRSRLFPQGRGVTL

LASRSYSSPLRSLCSASVPQRLHRRLECVASSAAYFGAAGGGGVYGGGDG

SGSGGGGGDGGEGTGDGDLKAKLGAGAVDEPSALSPDIIILDVGGMTCGG

CAASVKRILENQPQVSSASVNLTTETAIVWPVSEAKVVPNWQKELGEALA

RQLTSCGFNSNLRDSGRDNFFKVFERKMDEKRSRLKESGRELAVSWALCA

VCLIGHVAHFLGAKASWMHAFHSTGFHLSLSLFTLLGPGRQLIFEGVKNL

FKGAPNMNTLVGLGALSSFAVSSLAVLIPKLGWRAFFEEPVMLIAFVLLG

RNLEQRAKIKATRDMTGLLSSLPSQARLMVDDSIVEVPCSSLSVGDQIVV

LPGDRVPADGIVRAGRSTIDESSFTGEPMPVTKEPGSQVAAGSINLNGTL

TIEVRRPGGETAMGDIVRLVEEAQSREAPVQRLADKVAGHFTYGVMALSA

ATFMFWNLFGARIIPASIYQGSAVSLALQLSCSVLVVACPCALGLATPTA

MLVGTSLGATRGLLLRGGNILEKFSMVNVIIFDKTGTLTIGRPVVTKVVT

PSRMDHSDSRQHFDGSWSEDDVLKLAAAVESNTIHPVGKAIVEAAQAVKS

PNIKVVDGTFVEEPGSGAVAVIDDKTVSVGTLEWVQRHGVGDSLLLETDE

ELRNKSVVYVGVNNKLAGLIYFEDQIREDARHVVDSLHRQGISVYMLSGD

KRSTAEYVASIVGIPKDKVLSQVKPDEKRKFVSELQENQNVVAMVGDGIN

DAAALASAHIGVAMGGGVGAASDVSSIVLMGNRLSQLLDALALSQLTMKT

VKQNLWWAFAYNIVGIPIAAGTLLPLTGTMLTPSIAGALMGLSSIGVVTN

SLLLRFRFSLQQQQAYRSSLQPPPHAAMDINNDLAKDHSRAKLKKPDSIT

>GhHMA6

MESALSVTVPTLALFSIPRALNRHFYYNNCSLIARCIRSRLFPQGRGVTL

LASRSYSSPLRSLCSAPVPQRLHRRLECVASSAAYFGAAGGGGVYGGGDG

SGSGGGGGDGGEGTGDGDLKAKLGAGAVDEPSALSPDIIILDVGGMTCGG

CAASVKRILENQPQVSSASVNLTTETAIVWPVSEAKVVPNWQKELGEALA

RQLTSCGFNSNLRGRDNFFKVFERKMDEKRSRLRESGRELAVSWALCAVC

LIGHVAHFLGAKASWMHAFHSTGFHLSLSLFTLLGPGRQLIFEGVKNLFK

GAPNMNTLVGLGALSSFAVSSLAVLIPKLGWRAFFEEPVMLIAFVLLGRN

LEQRAKIKATSDMTGLLSSLPSQARLMVDDSIVEVPCSSLSVGDQIVVLP

GDRVPADGIVRAGRSTIDESSFTGEPMPVTKEPGSQVAAGSINLNGTLTI

EVRRPGGETAMGDIVRLVEEAQSREAPVQRLADKVAGHFTYGVMALSAAT

FMFWNLFGARIIPASIYQGSTVSLALQLSCSVLVVACPCALGLATPTAML

VGTSLGATRGLLLRGGNILEKFSMVNVIIFDKTGTLTIGRPVVTKVVTPS

GMDHSDSRQHFNGSWSEDDVLKLAAAVESNTIHPVGKAIVEAAQAVKSPN

IKVVDGTFVEEPGSGAVAVINDKTVSVGTLEWVQRHGVGDSLLLETDEEL

RNKSVVYVGVNNKLAGLIYFEDQIREDARHVVDSLYRQGISVYMLSGDKR

STAEYVASIVGIPKDKVLSQVKPDEKRKFVSELQENQNVVAMVGDGINDA

AALASAHIGVAMGGGVGAASEVPSIVLMGNRLSRLLDALALSQLTMKTVK

QNLWWAFAYNIVGIPIAAGTLLPLTGTMLTPSIAGALMGLSSIGVVTNSL

LLRFRFSLQQQQAYRSSLQPPPPPPPYAAVDINNDLAMDHSKAKLKKPDS

IT

>GhHMA12

MATKLLALACIRKESYGDLSPRPHYPSMPKYPKGITAQETSLQGSEAKAM

FSVMGMTCSACAGSVEKAVKRLPGIKEAVVDVLNNKAQVMFYPSFVNEES

ILEAIEDAGFQAALIQDETDDKFVQVCRIRINGMTCTSCSSTLENALQAV

PGVQKVQVALATEEAQIHHDPKIITYNQLMQKIEETGFGAVLVSTGEDMS

KINLRINGVRTVNSMRMLENSLQALPGVQAVQTSLELKKIAVSYKPDMTG

PRNFIKVIDSTGSSRRFKATIYPEGEGAGRESHRKEEIKQYFRSFLWSLI

FTTPVFLTSMVFMYIPGIKHGLDTKVVNMLTIGEVIRWVLSTPVQFIIGR

RFYTGSYKALRHGSANMDVLIALGTNAAYFYSVYTVIRAASSPDFEGTDF

FETSAMLISFILLGKYLEVLAKGKTSEAIAKLMNLAPETAILLSLDEEGN

VISEEEIDSRLIQKNDIIKIIPGAKVASDGFVLWGQSHINESMITGEARP

VAKRKGDTVIGGTVNENGVLHIKATKVGSESALAQIVRLVESAQMAKAPV

QKFADRISKYFVPLVIMLSFSTWLAWFLAGKLHGYPESWIPSSMDSFELA

LQFGISVMVIACPCALGLATPTAVMVGTGVGASLGVLIKGGQALEGAHKV

NCIVFDKTGTLTVGKPVVVNTRLLKNVVLHEFYELVAATEVNSEHPLAKA

IIEYAKKFREDEENPAWPEARDFVSITGHGVKAIVRNKEVIVGNKSLMLE

KNIVIPVDAQDMLTETESMAQTGILVSIDGEVTGVLAISDPVKPGAQEVI

SILKSMNVRSIMVTGDNWGTASSIASQIGIETVVAEAKPEQKAEKVKELQ

AEGYAVAMVGDGINDSPALVAADVGMAIGAGTDIAIEAADIVLMKSNLED

VITAIHLSRKTFSRIRLNYIWALGYNILGIPIAAGALFPSTGFRLPPWIA

GAAMAASSVSVVCCSLLLKNYERPKKLENLEIGGIQIE

>GhECA1

MEDAYARSVSEVLDFFGVDSSKGLTDFQVSQHARLYGKNVLPEEERTPFW

KLVFKQFDDLLVKILIAAALVSFLLALINGETGLIAFLEPSVILMILAAN

AAVGVITETNAEKALEELRAYQADIATVLRNGCFSILPATELVPGDIVEV

SELLFPADMRMIEMLSGQLRVDQAILTGESSSVEKDLESTIATNAVYQDK

TNILFSGTVVVAGRARAVVIGVGANTAMGSIRDSMLRTDDEATPLKRKLD

EFGTFLAKVIAGICVLVWIVNIGHFRDPAHGGFLRGAIQYFEIAVALAVA

AIPEGLPAVVTTCLALGTKRMARLNAIVRSLPSVETLGCTTVICSDKTGT

LTTNMMSVSKICVVHSIKNGPEVAEFGVSGTTYAPEGFIFDNTGVQLEFP

AQLPCLLHIAMCSALCNESLLQYNPDKGNYEKIGESTEVALRVLAEKVGL

PGFDSMPSALNMLSKHERASYCNHYWENQFKKVSVLEFSRDRKMMSVLCN

HKQMEIMFSKGAPESIISRCTNILCNNDGSTIPMDATLRAELDSRFNSFA

GKETLRCLALALKIMPMGQQILSFDDEKDLTFIGLVGMLDPPREEVRNAM

ISCMTAGIRVIVVTGDNKIKFQSTAESVCRKIGAFDHLVDYVGHSYTAAE

FEELPGTQQTMALQRMALLTRVEPSHKRMLVEALQNQNEVVAMTGDGVND

APALKKADIGVAMGSGTAVAKSASDMVLADDNFATIVAAVAEGRAIYNNT

KQFIRYMISSNIGEVVCIFVAAVLGIPDTLAPVQLLWVNLVTDGLPATAI

GFNKPDSDVMKAKPRKVSEAVVSGWLFFRYLVIGAYVGLATVAGFIWWFI

YSETGPKLPYTELMNFDTCPTRETTYPCSIFEDRHPSTVAMTVLVVVEMF

NALNNLSENQSLLVIPPWSNLWLVASIILTMLLHILILYVPSLSTLFSVT

SLSWNEWAVILYLSFPVIIIDEVLKFFSRNSHGIRFNFRFRRYDALPKKE

LRDK

>GhAHA8

MASDGDISLEQIKNEIVDLENIPVEEVFKLLKCTKEGLTTAEGETRLSIF

GHNKLEEKKDNKVLKFLGFMWNPLSWVMEAAAIMAIVLANGGGKPPDWPD

FVGIVLRDGKWSEQEASILVPGDIISVKLGDIIPADARLLEGDPLKIDQA

ALTGESLPVTKNPGDSVFSGSTCKQGELDAIVIATGIHSFFGKAAHLVDN

TNNVGHFQQVLTSIGNFCIFSIGIGMVIEIIVMYPIQHRNYRDGIDNLLV

LLIGGIPIAMPTVLSVTMAIGSHRLSEQGAITKRMTAIEEMAGMDILCSD

KTGTLTLNKLTVDKNLVEVFINDMDVDTLVLLAARASRVENQDAIDACIV

GMLSDPKQAREGITELHFLPFNPVDKRTAITYTDSKGEWHRCSKGAPEEI

IDLCGLTGGLRKKALSIIDGYANRGLRSLGVARQTIPEKTKESPGGPWEF

VGLLPLFDPPRHDSAETIKRALELGISVKMITGDQLAIGKETGRRLGMGT

DMYPSSALLGQCGDEDIAAIPIDDLIEKADGFAGVFPEHKYEIVRRLQER

KHICGMTGDGVNDAPALKKADIGIAVADSTDAARGASDIVLTEPGLSVIV

SAVLTSRSIFQRMKNYTIYAVSITIRIVLGFMLVALIWKFDFSPFMVLVI

AILNDGTIMTISKDRVRPSPKPDTWKLDEIFATGVVLGTYMAIVTVFFFW

LVHDTEFFTRTFGVKPINDNEDALTTALYLQVSTVSQALIFVTRSRRWSF

IELPGPLLLIAFILAQLVATLLAVYANWGFARIQGIGWEWAGAIWVFSII

TYIPLDVLKFFIRYALTGDAWGDVVQSKTAFKGECEVQLIANYRPLPGVS

SPPETWSNDELAPIV

>GhECA5

MEERAFPAWSWSVEHCLKEYDVRLDKGLSSYKVEKRREKYVWNELAKEKG

KPLWRLVLEQFDDMLVKILLVAAFISFLLAYMHGSESEESGFEAYVEPFV

IVLILVLNAIVGVWQETNAEKALEALKEMQCESGKVLRDGFLVPDLPARE

LVPGDIVELQIRAKSTTGEAMPVLKGTSPIFQKECELQAKENMVFAGTTV

VNGCCVCIVVCTGMNTEIGKIQKQIHEASLEESDTPLKKKLDEFGSRLTT

AIGIVCLIVWLINYKNFLSYDMVEGWPANVRFSFEKCTYYFKIAVALAVA

AIPEGLPAVITTSLALGTRKMAQKNAIVRKLPSVETLGCTTVICSDKTGT

LTTNQMSVAEFFTLGGKTTTSQICAVCNDAGIFCDGRLFRATGLPTEAAL

KVLAEKMGVPDAKMRNKIRDSELVANYLIDRSTVKLGCCEWWIKRSKRVA

TLEFDRVRKSSSIIVREAAGQSRLLAKGAVESLLERSTHVQLADGSLAPM

DEPCRQLLLSRQTEMSSKGLRCLGLAYKEDLGEFSDYYSENHPAHKKLLD

PACYCSIESDLVFVGAVGLRDPPRDEVHKAIEDCKRAEIRVMVITGDNKS

TAEAICREIKLFSDGEDLRGKSFTGKEFMALSPSQQIETLSKPGGKVFSC

AEPRHKQEIVRMLKEMGEIVAMTGDGVNDAPALKLADIGIAMGITGTEVA

KEASDMVLADDNFSTIVSAVAEECMIPVQLLWVNLVTDGPPATALGFNPP

DIGIMRKPPRRSDDALINSWVLFRYLIIGSYVGIATVGIFILWYTRASFM

GINLVSDGHTLVELSQLRN

>GhACA18

MSSSSGNQMYDCGTLLFKVTTSGFTTAQKRWRIAYASIYSVRVMLSLAKE

IISKRGIEQPSIISDLHPYVALDVEPSSSPHWGEKFSSSSFAPKIDRXNF

SSSSLAPKIDRKRLVETVKEKDLVSLHQLGGVEGIAAALGTNPEKGIRDD

DRDVVKRQEMFGTNTYHKPPPKGLLYFVLDAFKDTTILILLVCAALSLGF

GIKEHGAAEGWYEGGSIFVAVFLVIVVSALSNFRQETQFDKLSKISNNIK

VEVVRGGRRRQVSIFDLVVGDVVFLKIGDQIPADGLFLDGYSLQVDESSM

TGESDHMEVDATRNPFLFSGSKVADGYGQMLVASVGMDTTWGEMMSSITS

DKNERTPLQERLDRLTSSIGKVGLAVAFLVLVVLLIRYFTGNTEDDNGNT

EYIGSKTSVDDILNAVVRIVSAAVTIVVVAIPEGLPLAVTLTLAYSMKRM

MADQAMVRKLSACETMGSATIICTDKTGTLTLNQMKVTQFWLGQESIKED

HSNIIDHAVLELFYQGVGLNTTGSVCKPVSGSLPEFCGSPTEKAILSWAA

LGLDLDMEKLKQKYSILHVETFNSEKKRSGVSVRRKTDETLHVHWKGAAE

IIVAMCSDYYESNGGIRSMDEDQRSRIETIIQSMAASSLRCIAFAHKQVS

QKEMECVDDSEKTHQRIKEDGLTLLGIVGLKDPCRPGVKKAVEACKSAGV

DIKMITGDNIFTAKAIAAECGILGADYNEESGQAIEGIEFRNYTPEERME

KIGKIKVMARSSPFDKLLMVQCLKQKGDVVAVTGDGTNDALALKEADIGL

SMGIQGTEVAKESSDIVILDDNFSSVATVLRWGRCVYNNIQKFIQFQLTV

NVAALVINFIAAVSAGEVPLTAVQLLWVNLIMDTLGALALATDRPTKELM

KKPPVGRTEPLITNVMWRNLLAQAVYQIAILLILQFRGESMFNVPKRVKD

TLIFNTFVLCQVFNEFNARKLEKQNVFQGILQNRLFLGIVGITIILQVVM

VEFLKKFADTERLELWQWGVCILFAAFSWPIAWVVKLIPVSDKPFFSYLK

RSKSSSQLNKSSTTRNLQSAGMELEVQ

>GhACA9

MEELLKGFEVPPKNSSEAALRRWRKLVTIVRNPRRRFRMIANLEKRSEAE

QQKLKIKEKIRVALIVQKAALQFIDAAGPLDYKITDEVRQANFGIEPDEL

ASIVHGHDIKRLKSHGGVDGIAEKVTVSLDEGVLSENVSTRQRIYGFNRY

TEKPPRTFWMFVWDALQDLTLIILMICAVVSIGVGLATEGWPKGMYDGAG

ILLSIILVVLVTAISDYRQSLQFRDLDREKKKISVQVTRDGRRQQVSIYD

LVVGDVVHLGIGDQVPADGLFISGYSMQIDESSLSGETDPVDIYEQKPFL

LSGTKVRDGSAKMLVTAVGMRTEWGKLMETLNEGGEDETPLQVKLNGVAT

IIGKIGLTFAVLTFLVLTVRFLIEKALHNEFTKWSSTDALTLLDYFAIAV

TIIVVAVPEGLPLAVTLSLAFAMKQLMDERALVRHLSACETMGSASCICT

DKTGTLTTNHMVVNKIWICEKISNIGDNENKNIDELEIHESVFSIFLRSI

FLNSSAEVVKDENGKNSILGTPTETALLEFGLLLSADHDAYRRQFKILKV

EPFNSDRKKMSVLVALPEGRIQAFCKGAPEIVLRMCEKVVDSSGEVVLLS

EERVRDITEAINGFASDALRTLCVAVKDVGETFNENGIPDSGYTLIAVFG

IKDPVRPGVKEAVQTCLAAGITVRMVTGDNINTAKAIAKECGILTAEENA

IEGPEFSSKSPDEMKDIIPNIQVMARSKPSDKLNFVINLRNMFGEVVAVT

GDGTNDAPALRQSDIGLAMGIAGTEVAKENADVIVMDDNFATIVNVAKWG

RAVYINIQKFVQFQLTVNVVALIINFVSACISGSAPLTAVQLLWVNMIMD

TLGALALATEPPNDALMKRPPVPRGASFITKPMWRNIIGQSIYQLIVLGV

LNFDGKQLLRLTGSDATTVLNTVIFNSFVFCQVFNEINSREIEKINILRG

MFSSWVFLGVMASTVAFQVVIVEFLGTFASTVPLSWQLWLLCILIGSVSL

IVGVIVKCIPVERAAVKPKHHDGYDALPSGPELA

>GhHMA9

MELNGRDDLKRPLLEPSDSVCVTIPEPVDKLEKKRTVMFKIGNIKCASCV

TSIESVLGEINGVESVSVSPIHGYAAIEYVPKLVNPKIIKETIEDAGFPV

KEFSEQQIAVCRLRIKGMACTSCSESLERALKFLDGVKKAVVGLALEEAK

VHFDPNVTDSDRIIEAIEDAGFGADLISSGNEANKVHLKLEGVSSVEDMN

TIKSYLESAIGVNHVEMDLEEKMATVNYDPDFTGPRSIIEAVQEVPHGPY

TASLYIPPRQRETEQHHQINNYRNQFLLSCLFSVPLFIFSMVLPMLPPFG

DWLVYKIYNMFTVGLLLRWVLCRPVQFIVGRRFYKGSYHALRLKSANMDV

LVAMGTNAAYFYSVYVAIKSLSSDTFKGQDFFETSAMLISFILLGKYLEV

LAKGKTSDALAKLTDLAPDSACLLILDDDGNVVSEVAISTQLIQRNDVIK

IIPGEKVPVDGIVTDGQSYVNESMITGEAQPIAKKPGDKVIGGTMNENGC

LLVKATHVGSETALSQIVQLVEAAQLARAPIQKIADRISRFFVPAIVLTA

FITWLGWLIPGVIGIYPKHWIPKGMDKFELALQFGISVLVVACPCALGLA

TPTAVMVATGKGASLGVLIKGGNALEKAHKWKVTSSFQITRISASVKAIV

FDKTGTLTVGKPEVVNVMLFSSVSMEDFCDVAIAAEANSQHPIAKAFLEH

ARKLRQKIESNRQSNNQHVTEAKDFEVHPGTGVSGKVGDKMVLVGNKRLM

QTYNVTVGPEIEGYISEHEQQARTCVLVSIDGKIAGAFAVTDPVKPEANN

VILYLRSMGISSIMVTGDNWATATAIAKEVGIEKVIAETDPIGKADRIKD

LQMRGLTVAMVGDGINDSPALVAADVGMAIGAGTDVAIEAADIVLIKSNL

EDVVTAIDLSRKTISRIWLNYVWALGYNILGVPVAAGILYPFTGVRLPPW

LAGACMAASSLSVVCSSLLLQSYRKSWVFQDTKIGHSHCFKST

>GhHMA3

MVANKNLQKSYFDVLGLCCSSEVPLIENILKSLEGVKEVSVIVPTRTVIV

LHDNLLLSQLQIVKALNQARLEANVRAHGEIKYQKKWPSPFAVVCGLLLL

LSFLKYVYHPLQWLAVGTVVIGIYPVLFKGFAAITHFRIDINILILIAVI

GSVAMKDYTEAGTIVFLFTTAEWLESRASHKASAVMSSLMRITPQKAVIA

ENGEEVDADEVKLNTLLAVKAGEVIPIDGIVVDGRCEVDEKSLTGESLPV

TKEKDSTVWAGTINLNGYISVKTTAVAEDCVVAKMAKLVEEAQNSKSNTQ

RFIDKCAQFYTPVIIVVSAAIAVIPAALRAATSGLLVKGGDYLETLSKIR

ITAFDKTGTLTRGEFIVTDFQPLSQDISLDTLLYWVSSIESKSSHPMAAA

LVDYGRSHSIEPNPETVEDYQNFPGEGIYGRVDGRDIYIGSLKISVRAHG

TVPTLEGNMMKGKTIGFVYSGATPAGIFSLSDACRTGVLEAVEELKSMRI

KVAMLTGDNQSAAIHVQEQLGNRLDVVHAELLPEDKARIIKEFKKEGATA

MIGDGVNDAPALATADIGISMGISGSALATETGHVVLMSNDIRKIPKAIK

LARKAHRKVIENVILSISTKTAILALAFAGHPLVWAAVLADVGTCLLVIC

NSMLLLRGKHKDGRKCCKSSAAAHTNKHGCKASHCDSSHKHQDASLDKKV

QKACEPPTCSSERCASRCHSGLFKTDSPSNSRGSDKCEDLGRTHDGSVIR

EAKYCDQGSCHLVNHKIEAQNLPRKCCSGRGSLDLGKEANALHGAKQCHQ

GHLHQYSSSTPEEEQRETKNDHCHSTHCRENHVEIHGNNLTAFGNLVEHR

CLESLNQRAHLDSHEPTHTAIDITMNPDEVHGCANVEKRELGGCCKSYMK

ECCGKHKHGRFRPGLTDIITE

>GhACA10

MEELLKDFEVPPKNSSEAALRRWRKLVTIVRNPRRRFRMIANLEKRSEAE

QQKLKIKEKIRVALIVQKAALQFIDAAGPPDYKITDEVRQAKFGIEPDEL

ASIVHGHDIKRLKSHGGVDGIAEKVTVSLDEGVCSENVSTRQRIYGFNRY

TEKPPRNFWMFVWDALQDLTLIILMICAVVSIGVGLATEGWPKGMYDGAG

ILLSIILVGLVTAISDYRQSLQFRDLDREKKKISVQVTRDGRRQQVSIYD

LVVGDVVHLGIGDQVPADGLFISGYSVQIDESSLSGETDPVDIYEQKPFL

LSGTKVRDGSAKMLVTAVGMRTEWGKLMETLNEGGEDETPLQVKLNGVAT

IIGKIGLTFAVLTFLVLTVRFLIEKALHNEFTKWSSTDALTLLDYFAIAV

TIIVVAVPEGLPLAVTLSLAFAMKQLMDERALVRHLSACETMGSASCICT

DKTGTLTTNHMVVNKIWICEKIRNIGGNENKSIDELEIHESVFSILLRSI

FLNSSAEVVKDENGKNSILGTPTETALLEFGLLLSADLDAYRRQFKILKV

EPFNSDRKKMSVLVALPEGRIQAFCKGAPEIVLRMCEKVVDSSGEVVLLS

EERVRDITEAINGFASDALRTLCVAVKDVGETFNENGIPDSGYTLIAVFG

IKDPVRPGVKEAVQTCLAAGITVRMVTGDNINTAKAIAKECGILTAEENA

IEGPEFSSKSPDEMKDIIPNIQVMARSKPSDKLNFVINLRNMFGEVVAVT

GDGTNDAPALRRSDIGLAMGIAGTEVAKENADVIVMDDNFATIVNVAKWG

RAVYINIQKFVQFQLTVNVVALIINFVSACISGSAPLTAVQLLWVNMIMD

TLGALALATEPPNDALMKRPPVPRGASFITKPMWRNIIGQSIYQLIVLGV

LNFDGKQLLKLTGSDATTVLNTVIFNSFVFCQVFNEINSREIEKINIFRG

MFSSWIFLGVMASTVAFQVVIVEFLGTFASTVPLSWQLWLLCILIGSVSL

IVGVIVKCIPVERAAVKPKHHDGYDALPSGPELA

>GhACA16

MSLRLRKPSEPTMHRQVEPSKSSVRRWRVAVTAISVTRFLVGLTKKVVEK

NAELLRSLSFVTIDVEGSGDERVPILDVDPQGLAKMVKDKSFQSLNDQYG

GVKQVATLLQTDFKKGIPGDDNDLALRTKVFGANKYQKQPAKSFFSFVLE

AFKDTIIIILLVCAVLSLGFGIKQHGLKEGWYDGGSIIVAVVLVVVVSAV

SNYRQSKQFEELSHETNDIRVQVVRNGRYQPVSIFELVVGDIVSLKTGDQ

IPADGLFVEGHSLKVDESSMTGESDHVEVNEKKNPFLLSGTKVTDGHGYM

LVTAVGMNTAWGEMMSSIRRDLNEETPLQARLSKLTSYIGNIGLSVAVLV

LLVLLIRYFTGHTKAENGRSAFNGSKTKFDDVMNSVVGIIAAAVTIVVVA

IPEGLPLAVTLTLAYSMKRMMREHAMVRKLSACETMGSATIICTDKTGTL

TLNEMKVTEFWLGKEPIDNSMSSEIAPNVLQLLSEGVGLNTTGTVYKPKP

TSVPEIYGSPTEKAILSWALNDMGLNIDESKQSCEIIHVEAFNSEKKRSG

VLIRRSNNKRVLATHWKGAAEMLLAMCSCYYDKKGVSKFMNEDEREHIGM

VIESMAAKSLRCIAFATSDVTVTDGNEENHTKLEERGLTWLGLVGLKDPC

RPGVKQAVESCQNAGVSIKMITGDSMHTARAIAFECGILNSESSLHNEAV

VEGVQFRNYSEEERMQKIETIRVLARSSPFDKLLMVQCLKQKGHVVAVTG

DGTNDAPALKEADIGLSMGIQGTEVAKESSDIVILDDNFTSVATVLRWGR

CVYNNIQKFIQFQLTVNVAALVINFIAAVSSGDVPLTAVQLLWVNLIMDT

LGALALATEQPTNDLMDKRPVGRTEPLITKVMWRNLIAQALYQVTILLIL

QFKGKSIFGVSEEVKDTLIFNTFVLCQIFNEFNARNMDKKNIFKGIHKNR

LFLAIIGITLVLQAIMVEFLQRFANTERLSWEQWGACIGIAALTWPIGWI

VKCIPVDKKVQTRSSAAS

>GhACA8

MVADLAKRAEADRRRKIIQEKIRVALYVQKAALNFIDAGKQAERKLPEDV

REAGFHIGADELASIVRSHDMSSFEENGGVEGLAKKVSVSLTNGVVPTDI

SFRKNIYGNNKFDEKPARSFWTFVWEALHDLTLIILIVCAVVSIGVGVAT

EGWPGGLYDGLGIVLCIFLVVFVTAISDYKQSLQFKDLDKEKKNILVQVT

REGCRQKISIYDLVVGDIVHLSIGDQVPADGVLISGYSLSIDESSLSGES

EPVKVTQERPFLLSGTKVQDGSGKMLVTTVGMRTEWGRLMVTLSEGGVDE

TPLQVKLNGVATVIGKIGLVFAVLTFLVLAIRFMVTKAQLGEIEKWVMSD

VLSLLNFFAVAVTIIVVAVPEGLPLAVTLSLAFAMKKLMSDKALVRHLSA

CETMGSATCICTDKTGTLTTNHMVVDKIWTCGRTISIAGDNKREDVLRSS

IAGEVLDLLLQSIFQNTGAEVVKGKDGKNNILGSPTETAILEFGLLLGGE

FKKHRKESTILKVEPFNSEKKRMSVLVSLSNGGENRAFCKGASEIILESC

NKVINVDGKAEHLSKEQKKFITDVINGFACEALRTLCLAFKDVKDTSDVD

SDSIPQENYTLIAVIGIKDPVRPGVRQAVETCLSAGIKVRIVTGDNINTA

KAIARECGILTENGLAIEGPEFRDMSPRQMEETIPKLQVLARSLPLDKHK

LVTYLRMEFKEVVAVTGDGTNDAPALHEADIGLAMGIAGTEVAKENADVI

IMDDTFATIQNVARWGRAVYINIQKFVQFQLTVNIVALMLNFVSACISGS

APLTAVQLLWVNMIMDTLGALALATEPPHEGLMKRPPIGRDVAFITRVMW

RNIIGQTIYQLIVLAILKFDGKRLLKISGSNATAILNTLIFNSFVFCQVF

NEINSRDMEKINVFRGFFDSWLFIMVMVCTVGFQSIIVELLGTVADTVPL

SWELWLISILLGAGSLIVAVILKCIPVENCKEASTTKHHDGYEPLPTGPD

MA

>GhACA19

MSSSDGNQMYDCGTLLFKVTTSGFTTAQKRWRIAYASIYSVRVMLSLAKE

IISKRGIEQPSIISDLHPYVALDVEPSSSPQWGENFSSSSLAPKIDRQET

QFDKLSKISNNIKVEVVRGGRRRQVSIFDLVVGDVVFLKIGDQIPADGLF

LDGYSLQVDESSMTGESDHMEVDVTRNPFLFSGSKVADGYGQMLVASVGI

DTTWGEMMSSITSDKNERTPLQERLDRLTSSIGKVGLAVAFLVLVVLLIR

YFTGNTEDDNGNTEYIGSKTSVDDILNAVVRIVSAAVTIVVVAIPEGLPL

AVTLTLAYSMKRMMADQAMVRKLSACETMGSATIICTDKTGTLTVNQMKV

TQFWLGQESIKEDHSNIIDHTVLELFYQGVGLNTTGSVCKPVSGSLPEFS

GSPTEKAILSWAVLGLDLDMEKLKQKYSILHVETFNSEKKRSGVSVRRKT

DETLHVHWKGAAEIIVAMCSDYYESNGGIRSMDEDQRSRIETIIQSMAAS

SLRCIAFAHKQVSQKEMECVDDSEKTHQRIKEDGLTLLGIVGLKDPCRPG

VKKAVEACKSAGVDIKMITGDNIFTAKAIAAECGILGADYNEESGQAIEG

IEFRNYTPEERMEKIGKIMVMARSSPFDKLLMVQCLKQKGDVVAVTGDGT

NDALALKEADIGLSMGIQGTEVAKESSDIVILDDNFSSVATVLRWGRCVY

NNIQKFIQFQLTVNIAALVINFIAAVSAGEVPLTAVQLLWVNLIMDTLGA

LALATDRPTKELMKKPPVGRTEPLITNVMWRNLLAQAVYQIAILLILQFR

GESMFNVPKRVKDTLIFNTFVLCQVFNEFNARKLEKQNVFQGILQNRLFL

GIVGITIILQVVMVEFLKNFADTERLKLWQWGLCILFAAFSWPIAWVVKL

IPVSDKPFFSYLKRSKPSSQLNKSSTTRNLQSAGMELEVQ

>GhACA17

MSLRHRNTGYLEPAMSDGEDTPIVKCQHRRWRSVFAAIYSARIFVSLYKK

IINKKQILRSLSYIALDVHDSDSCDDRLPFLGVDQKTLTEVVREKSLETL

SKLGGVKQIAASLETDKKDGISANEADLAHRVDVFGANRYQKPPKKSFFS

FVYEAFKDTTIIILLVCAVLSLGFGIKQHGITDGGYDGGSIVIAVFLVVA

VSAVSNFKQNRQFEKLSKESSDIKVEVVRDGRRQFISVFEVVVGDVVCLK

IGDQIPADGLFLDGHSLKVDESSMTGESDHVEINGSNNPFVLSGTKVTNG

FGSMLVTSVGMNTAWGEMMSSINRELDEETPLQARLNKLTSAIGKIGLAV

AVLVLAVLLIRYFTGNTKDDQGNKEYIRGKTKFDSMMNSVVEIISAAITI

VVVAIPEGLPLAVTLTLAYSMKQMMAEHAMVRKLSACETMGSATTICTDK

TEIAPNVHKLLQQAVALNTTGTVYKPNSRSLPEISGSPTEKAILSWAVSD

LGMNLDDPKQNYELIQVEAFNSEKKRSGVLIRRKSESGGATQVHWKGAAE

MILAMCSQYYDRSGVVKAIDEEERVEMGKVIEDMAAKSLRCIAFAHTQNP

KDNERVLQESGLILLGLVGLKDPCRPGVRKAVEACINAGVNIKIITGDNI

FTAKAIATECGILQPNEDLREAVIEGVQFRNYSPEERMAKINKICVMARS

SPFDKLLMVQCLKQNGHVVAVTGDGTNDAPALKEADIGLSMGIQGTEVAK

ESSDIIILDDNFTSVVTVLRWGRCVFNNIQKFIQFQLTVNIAALVINFIA

AVSSGEIPLTAVQLLWVNLIMDTFGALALATERPTNDLMTKPPVGRSKPL

ISNIMWRNLIAQALYQVAVLLTLQFRGKFIFDVDKKVKNTLIFNTFVLCQ

VFNEFNARKLEKKNIFQGLQKNKLFLGIIAITIILQVVMVEFLKRFANTQ

RLNWGQWGTCIGIAALSWPLGWLVKWIPA

>GhECA2

MEDAYARSVSEVLDFFGVDSSKGLTDFQVSQHARLYGKNVLPEEERTPFW

KLVFKQFDDLLVKILIAAALVSFLLALINGETGLIAFLEPSVILMILAAN

AAVGVITETNAEKALEELRAYQADIATVLRNGCFSILPATELVPGDIVEV

SVGCKIPAEMRRIEMLSGQLRVDQAILTGNWTICFCCIIFEVVLKPQNEK

GLLPRIFMYQTVLIVFPCSLRCESSSVEKDLESTIATNAVYQDKTNILFS

GTVVVAGRARAVVIGVGANTAMGSIRDSMLRTDDEATPLKKKLDEFGTFL

AKVIAGICVLVWIVNIGHFRDPAHGGFLRGAIHYFKIAVALAVAAIPEGL

PAVVTTCLALGTKRMARLNAIVRSLPSVETLGCTTVICSDKTGTLTTNMM

SVSKICVVHSIKNGPEVAEFGVSGTTYAPEGFIFDNTGVQLEFPAQLPCL

LHIAMCSALCNESLLQYNPDKGNYEKIGESTEVALRVLAEKVGLPGFDSM

PSALNMLSKHERASYCNHYWENQFKKVSVLEFSRDRKMMSVLCNHKQMEI

MFSKGAPESIISRCTNILCNNDGSTIPIDATLRAELDSRFNSFDLRRVSS

LFSFAGKETLRCLALALKIMPMGQQTLSFDDEKDLTFIGLVGMLDPPREE

VRNAMISCMTAGIRVIVVTGDNKSTAESVCRKIGAFDHLVDYVGHSYTAA

EFEELPGTQQTMALQRMALLTRVEPSHKRMLVEALQNQNEVVAMTGDGVN

DAPALKKADIGIAMGSGTAVAKSASDMVLADDNFATIVAAVAEGRAIYNN

TKQFIRYMISSNIGEVVCIFVAAVLGIPDTLAPVQLLWVNLVTDGLPATA

IGFNKPDSDVMKAKPRKVSEAVVSGWLFFRYLVIGAYVGLATVAGFIWWF

IYSETGPKLPYTELMNFDTCPTRETTYPCSIFEDRHPSTVAMTVLVVVEM

FNALNNLSENQSLLVIPPWSNLWLVASIILTMLLHILILYVPPLSTLFSV

TSLSWNEWAVILYLSFPVIIIDEVLKFFSRNSHGNAYCSFGSVSSDLINN

HVSYICDERIRFNFRFRRYDALPKKELRDK

>GhACA20

MTTILQSNLLCFEYTIQVPSATFTKSRKKWHSLFATIYCSRKFSSLITKT

ATANDEARVVHRSPSHVSLAVMQENSPFRIDQPTLIELVKEKKIEKLRKH

GGVDGVASGLGTDTQVGVSGSAEDIERRREAFGSNTYKKPPTKGFFHFVV

EAFKDLTIMILLGCAALSLGFGIKEHGLKDGWYDGGSIFVAVFLVIAVSA

VSNYRQDRQFDKLSKVSNNIQVDVVRGGRRQQISIFDIVVGDIVCLKIGD

QVPADGLFIDGHSLQIDESSMTGESDHVEVNGNQNPFLLSGTKVADGYAR

MLVTSVGMNTTWGQMMCQISRDTNDETPLQARLNKLTSSIGKVGLAVAFL

VLVVLLVRYFTGHTTDENGNREFNGSKTKSDDIINAVVGIVAAAVTIVVV

AIPEGLPLAVTLTLAYSMKRMMADQAMVRKLSACETMGSATTICTDKTGT

LTLNRMKVTKFWLGQESMEEGASSISPFVVDLIHQGVALNTTGSVYRASP

GTEYEFSGSPTEKAILSWAVVELKMDMEKTKKCYAVLQVEAFNSQKKRSG

VLIGRNDDDTVHVHWKGAAEMILALCSSYYDASGVEKDLDDDERTKLEQI

IQGMAASSLRCIAFAHKQVPEEEYQNLKEQKKLKEDSLALLGLVGIKDPF

RPGVKKAVEDCQYAGVNIKMITGDNVFTARAIATECGILKPGQDLSSGAV

VEGEEFRNYTPHERMEKVEKIQVMARSSPFDKLLMVQCLKQKGHVVAVTG

DGTNDAPALKEADIGLSMGIQGTEVAKESSDIVILDDNFASVATVLRWGR

CVYTNIQKFIQFQLTVNVAALCINFVAAVSAGEVPLTAVQLLWVNLIMDT

LGALALATERPTKELMEKPPVGRTEPLITNIMWRNLLAQALYQIAVLLTL

QFSGESIFGVTEKVNDTLIFNIFVFCQVFNEFNARKLEKKNVFEGIHKNK

LFIGIIGVTILLQVVMVEFLKRFADTERLTWGQWGACIAVAAVSWPLGWV

VKCLPVPQKPIFSYLKWRK

>GhACA21

MTTILQSNLLCFEYTIQVPSATFTKSRKKWHSLFATIYCSRKFSSLITKT

ATANDEARVVHRSPSHVSLAVMQENSPFRIDQPTLIELVKEKKVEKLRKH

GGVDGVASGLGTDTQVGVSGSAEDIERRHEAFGSNTYKKPPTKGFFHFVV

EAFKDLTIMILLGCAALSLGFGIKEHGLKDGWYDGGSIFVAVFLVIGVSA

ISNYRQNRQFDKLSKVSNNIQVDVVRGGRRQQISIFDIVVGDIVCLKIGD

QVPADGLFIDGHSLQIDESSMTGESDHVEVNGSQNPFLLSGTKVADGYAR

MLVTSVGMNTTWGQMMCQISRDTNDETPLQARLNKLTSSIGKVGLAVAFL

VLVVLLVRYFTGHTTDENGNREFNGSKTKSDDIINAVVGIVAAAVTIVVV

AIPEGLPLAVTLTLAYSMKRMMADQAMVRKLSACETMGSATTICTDKTGT

LTLNRMKVTKFWLGQESMEEGASSISPFVVDLIHQGVALNTTGSVYRASP

GTEYEFSGSPTEKAILSWAVVELKMDMEKTKKCCAVLQVEAFNSQKKRSG

VLIGRNDDDTVHVHWKGAAEMILALCSSYYDASGVEKDLDDDERMKFEQI

IQGMAASSLRCIAFAHKQVPEEEYQNLKEQKKLKEDSLTLLGLVGIKDPC

RPGVKKAVEDCQYAGVNIKMITGDNVFTARAIATECGILKPGQDLSSGAV

VEGEEFRNYTPHERMEKVEKIQVMARSSPFDKLLMVQCLKQKGHVVAVTG

DGTNDAPALKEADIGLSMGIQGTEVAKESSDIVILDDNFASVATVLRWGR

CVYTNIQKFIQFQLTVNVAALCINFVAAVSAGEVPLTAVQLLWVNLIMDT

LGALALATERPTKELMEKPPVGRTEPLITNIMWRNLLAQALYQVAILLTL

QFSGESILGVTEKVNDTLIFNIFVFCQVFNEFNARKLEKKNVFEGIHKNK

LFIGIIGVTILLQVVMVEFLKRFADTERLSWGQWGACIVVAAVSWPLGWV

VKCLPVPQKPIFSYLKWWK

>GhACA7

MESYLNENFGDVKPKNSSEEALERWRKLCWIVKNRKRRFRFTANLSKRFE

AEAIRRSNQEKFRVAVLVSQAALQFIHGLNLSSEYDAPEEVKAAGFQICA

DELGSIVEGHDVKKLKIHGGVEDIAAKLSTSIVNGIPTSEHLVNKRKRIY

GINKFTETPPRGFWVFVWEALQDTTLMILAVCALVSLAVGITVEGWPKGA

YDGLGIVLSILLVVFVTATSDYRQYLQFRDLDKEKKKITVQVTRDGLRQK

ISIFDLLPGDIVHLAIGDQVPADGLFISGFSVLINESSLTGESEPVSANA

RNPFLLSGTKVQDGSCKMLVTTVGMRTQWGKLMATLSEGGDDETPLQVKL

NGVATIIGKIGLFFAVVTFAVLVQGLFSRKLQDGTQWIWSGDDAMEMLEF

FAIAVTIVVVAVPEGLPLAVTLSLAFAMKKMMNDKALVRHLAACETMGSS

TSICSDKTGTLTTNHMTVVKTCFCGETKEVSTSNKSNHFRSAVPESAVKI

LIESIFNNTGGEVVNNKENKIEILGTPTETALLEFGLLLGGDFQAERKAS

KIVKVEPFNSAKKRMGVVIEFPEGGLRVHCKGASEIILAACDKVISSNGD

VVPLDEPTTNHLKNTIEQFASEALRTLCLAYMDVGTDFSVDSSLPLQGYT

CIGIVGIKDPVRPGVKESVAICKSAGITVRMVTGDNINTAKAIAREIGIL

TDDGIAIEGPVFREKSEEELYELIPKIQVMARSSPMDKHTLVKHLRTSLG

EVVAVTGDGTNDAPALHEADIGLAMGIAGTEVAKESADVIILDDNFSTIV

TVAKWGRSVYINIQKFVQFQLTVNVVALIVNFTSACLTGNAPLTAVQLLW

VNMIMDTLGALALATEPPNDDLMKRSPVGRKGNFISNTRGKAAFHLDGPD

SDLILNTLIFNSFVFCQVFNEISSREMEKINVLKGILKNHVFVAVLSCTI

IFQIVIVEFLGTFASTSPLTVQQWFVSVCLGFLGMPIAAALKLIPVGSN

>GhACA3

MDSYLNQNFDLKSKHSSDEALEKWRTVVGFVKNPKRRFRFTANLSKRYEA

AAMRRTNQEKLRIAVLVSKAAFQFISGVQPSDYVVPEQVIAAGFQLCADE

LGSIVEGHDVKKLKFHGGVSGIAEKLSTSTNTGLSSDAALLSKRQEIYGI

NKFAEPEAKGFWVFVWEALQDMTLMILGACAIVSLVVGIAMEGWPAGAHD

GLGIVASILLVVFVTATSDYRQSLQFKDLDKEKKKITIQVTRNDCRQKLS

IYDLLPGDIVHLNIGDQVPADGLFVSGFSVLIDESSLTGESEPVVVNDKN

PFMLSGTKLQDGSCKMLVTTVGMRTQWGKLMATLSEGGDDETPLQVKLNG

VATIIGKVGLFFAVVTFAVMVQGLLRSKLQEGTIWSWSGDEALKLLEFFA

VAVTIVVVAVPEGLPLAVTLSLAFAMKKMMNDKALVRHLAACETMGSATG

ICSDKTGTLTTNRMTVVKSCICMSVKEINSTNKASFCSEIHESAIKLLLQ

SIFMNTGGEIVTSKDGRREILGTPTETALLEFGLSLGGDPQTERQASKTV

KVEPFNSTKKRMGVVLELPGGGLRAHTKGASEIVLAGCDKVIDSNGEVVP

LDEKSINHLNATINEFANEALRTLCLAYLELKNGFSSDNAIPVSGYTCLG

IVGIKDPVRPGVKESVAICRTAGITVRMVTGDNINTAKAIARECGILTDD

GIAIEGPDFREKSQEELLKLIPKIQVIARSSPMDKHTLVKQLRTTFDEVV

AVTGDGTNDAPALHEADIGLAMGIAGTEVAKESADVIILDDNFSTILTVA

KWGRSVYINIQKFVQFQLTVNIVALIVNFSSACLTGSAPLTAVQLLWVNM

IMDTLGALALATEPPTDELMKRAPVGRKGNFISNVMWRNIFGQSFYQLMV

IWYLQARGKAMFELDGPDSTLKLNTLIFNSFVFCQVFNEISSRNMEEINV

LSGILNNSVFVAVLGCTAVFQIIIIEFLGTFASTTPLTYSQWGLSVVIGF

FSMPIAAALKLVPV

>GhAHA27

MGDKNEVLEAVLKETVDLENIPIEEVFENLRCSREGLTTEAAEERLTIFG

HNKLEEKKESKFLKFLGFMWNPLSWVMEAAAIMAIALANGGGKPPDWQDF

VGIITLLVINSTISFIEENNAGNAAAALMARLAPKAKVLRDGRWNEQDAA

ILSALTGESLPVTKGPGDGIYSGSTCKQGEIEAVVIATGVHTFFGKAAHL

VDTTNQVGHFQKVLTAIGNFCICSIAVGMVIEIIVMYPIQDRDYRPGIDN

LLVLLIGGIPIAMPTVLSVTMAIGSHRLSQQGAITKRMTAIEEMAGMDVL

CSDKTGTLTLNKLTVDKNLIEVFAKGVDADTVVLMAARASRTENQDAIDS

AIVGMLADPKEARAGIREVHFLPFNPTDKRTALTYIDSDGKMHRVSKGAP

EQILHLAHNKTDIERRVHAVIDKFAERGLRSLAVAYQEVPDGRKESPGGP

WQFIGLMPLFDPPRHDSAETIRRALNLGVNVKMITGDQLAIGKETGRRLG

MGTNMYPSSALLGQDKDESIAALPVDELIEKADGFAGVFPEHKYEIVKRL

QARKHICGMTGDGVNDAPALKKADIGIAVADATDAARSASDIVLTEPGLS

VIISAVLTSRAIFQRMKNYTIYAVSITIRIVLGFMLLALIWKFDFPPFMV

LIIAILNDGTIMTISKDRVKPSPLPDSWKLAEIFTTGIVLGSYLAVMTVI

FFWAAYKTNFFPRVFGVATLEKTAHDDIKKLASAVYLQVSIISQALIFVT

RSRSWSFVERPGLLLLAAFVIAQLIATLIAVYANWSFAAIEGIGWGWAGV

IWLYNIIFYIPLDFIKFFIRYALSGRAWDLVIEQRIAFTRQKDFGKEQRE

LQWAHAQRTLHGLQAPDTKMFTERTHFTELNQMAEEAKRRAEIARLRELH

TLKGHVESVVRLKNLDIDTIQQAYTV

>GhACA5

METFWNESFDLKPKHSSEEALEKWRKVVGFVKNPKRRFRFTANLSKRYEA

AAMRRSNHEKLRIAVLVSKAALQFISGVKPSESDYVVPEEVKAAGFELCA

EELGSIVEGQDVKKLKIHGGVDGIAEKLSTSTTDGLSSDSVLLNKRQEVY

GINKFAEAEAKGFLVFVWEALQDMTLMILGVCALVSLIVGIAMEGWPKGA

HDGLGIVASILLVVFVTATSDYRQSLQFKDLDKEKKKITIQVTRNACRQK

MSIYDLLPGDIVHLNIGDQVPADGLFVSGFSVLIDGSSLTGESEPVMVNA

DNPFMLSGTKLQDGSCKMMVTSVGMRTQWGKLMATLSEGGDDETPLQVKL

NGVATIIGKVGLFFAVVTFAVMVQGLFMSKLQEGTIWSWSGDEALKLLEF

FAVAVTIVVVAVPEGLPLAVTLSLAFAMKKMMNDKALVRHLAACETMGSA

TNICSDKTGTLTTNHMTVVKSCICMDVREVGNNNKASLCSEIPESAVKLL

LQSIFTNTGGEIVINKDGKREILGTPTETALLEFGLSLGGDSRAERLASK

LVKVEPFNSTKKRMGVILELPEGGLRAHTKGASEIVLAGCDKVINSNGEV

VPLDVESINHLNATINQFANEALRTLCLAYMELENDFSPDNAIPLSGYTC

IGIVGIKDPVRPGVKESVAICRSAGITVRMVTGDNINTAKAIARECGILT

DDGIAIEGPDFREKSQEEMLALIPKIQVMARSSPMDKHTLVRQLRSIDEV

IAVTGDGTNDAPALHEADIGLAMGISGTEVAKESADVIILDDNFSTIVTV

AKWGRSVYINIQKFVQFQLTVNIVALIVNFSSACLTGTAPLTAVQLLWVN

MIMDTLGALALATEPPTDELMKRAPVGKKGNFISNVMWRNILGQSFYQFM

VIWYLQVKGKGMFSLEGPDSDLTLNTIIFNSFVFCQVFNEISSRNMEEIN

VFKGILNNYVFVAVLGCTAVFQVIIIEFLGTFASTTPLTCLQWFVSVFIG

FLGMPVAAALKTIPV

>GhACA11

MGGFSSSDGCLQDLEAGPSKDNNDLNTNLDPDADTSDPFDIDQTKNATPQ

TLKRWRQAALVLNASRRFRYTLDLRKQEEKEQRKRMIRAHAQVIRAALLF

KLAGEKEIVSGTPVTLPGAAGDFAVGLEQLASMTRDHKLSALQQYGGVKG

LSDLLKTNLETGIYGDEVDLLNRKTAFGSNTYPRKKGRSFWRFLWEAWQD

LTLIILIVAASVSLGLGIKTEGLKEGWYDGGSIFLAVLLVIVVTATSDYR

QSLQFQNLNEEKRNIQLEVLRGGRTVKVSIYDVVVGDVVPLKIGDQVPAD

GVLVSGHSLAIDESSMTGESKIVHKDKKEPFLMSGCKVADGVGTMLVTGV

GINTEWGLLMASISEDTGEETPLQVRLNGVATFIGIVGLSVAVSVLVVLL

ARYFTGHTEDPDGTKQFIKGRTNFDDAFNDVVKIFTIAVTIVVVAVPEGL

PLAVTLTLAYSMRKMMADKALVRRLSACETMGSATTICSDKTGTLTLNEM

TVVEAFVGKKKINPPSDSSQLPASVVSLLNEGVAQNSTGNVFVPKDGGNI

EISGSPTEKAILSWAVKLGMKFDIIRSDSKILHVFPFNSEKKRGGVALQG

ADSEVRIHWKGAAEIVLTSCSGYIDSNGCLQSINEDKEFFKAAIDEMAVN

SLRCVALAYRLCEKEKVPTDEEGFNGWILPEDNLVLLAIVGIKVHLFFLV

MVRIIYFYPNSIC

>GhHMA1

MDLANFLREHLHLCCCATALFIAAAACPYLVPKPAVKPLQNSFLVLAFPL

VGVSAALDAITDIAGGKVNIHVLMALAAFASVFMGNALEGGLLLAMFNLA

HIAEEFFTSRSMIDVKELKENYPDSALVLNLDDDNLPNVSDLSYQSIPVH

DVEVGSYILVTTGEAVPVDCEVFHGSATITIEHLTGEIKPLEAKAGDRIP

GGARNLDGRMIVKVLKTWKESTLSRIVQLTEEAQLNKPKLQRWLDEFGEQ

YSKVVVVLSVAIAVLGPFLFKWPFISTAVCRGSIYRALGLMVAASPCALA

VAPLAYATAVSSCARKGILLKGGQVLDALASCHTVAFDKTGTLTTGGLMF

KAIEPIYGHIIGNKKTNLTSCCVPSCEVEALAVAAAMEKGTTHPKDLPSV

SVESFEYFPGKGLIATLNSAESGTRGGKMLKASLGSIEFITSLCKSEVES

RKIRAAVNASSFGTDFVHAALSVDEKVTLIHLEDRPRPGVLDVISELKDK

AKVRVMMLTGDHKLSAWRVANAVGINEVYCSLKPEDKLNHVKRISGDMGG

GLIMVGEGINDAPALAAATVGIVLAHRASATAIAVADVLLLQDNISGVPF

SIAKARQTTSLVKQNVALALTCIILASLPSVLGFLPLWLTVLLHEGGTLL

VCLNSIRALNDPSWSWGQDLRNLIGKLKSKLALLRHNATSSPIQTAPL

>GhACA15

MSSIFKGSPYRRPNDLEAGSSRSAHSDDEDHESCADPFDITSTKNAPIDR

LRRWRQAALVLNASRRFRYTLDLKKEEEKKQILRKIRAHAQAIRAAYLFK

QAGEQVNGTTTPHPTPGSDFAFGPEQLASVTRDHNFNALQEYGGVNGLAE

SLKTNLQKGIPGDDSDLLKRRNAFGSNTYPRKKGRSFWRFVWEACQDLTL

MILVVAAVASLALGIKTEGPKEGWYDGGSIAFAVFLVIIVTAISDYRQSL

QFQKLDEEKRNIHLEVVRGGRRVEISIYDIVVGDIIPLNIGDQVPADGIL

ISGHSLAIDESSMTGESDIVQKDAKQPFLMSGCKVADGSGTMLVTGVGIN

TEWGLLMANLSEDTGEETPLQVRLNGVATFIGFVGLSVAFAVLVVLLVRY

FTGHTEDSNGRQQFVAGKTSVGNAIDGAIKIVTVAVTIVVVAVPEGLPLA

VTLTLAYSMKKMMADKALVRRLSACETMGSATTICSDKTGTLTLNQATYA

GGRKNDPPESRSELPDTLVSLLIEGIAGGGDVEVSGSPTEKAILNWGIKL

GMDFDAVRSGSSIVHVFPFNSEKKRGGVAIRLLSLWSLFEKFLGRLVTTA

AQASNSNPIFISKPDSKVHIHWKGAAEIVLAACTRYLDTNGEAVAMDEEK

MAFFEKAIETMAAGSLRCVAIAYRSYDSEKVPTNEEELAKWALPEDDLVL

LAVVGIKIYVLWSVKRVVSSCAGLQNDPCRPSVKKSVQLCQKAGVKVRMV

TGDNLKTARAIALECGILSSDAPESSLIEGKVFRSLSDSEREEVAEKISV

MGRSSPNDKLLLVQALRRKGHVVAVTGDGTNDAPALHEADIGLAMGIQGT

EVAKESSDIIILDDNFASVVKVVRWGRSVYANIQKFIQFQLTVNVAALVI

NVVAAVSSGDVPLNAVQLLWVNLIMDTLGALALATEPPTDHLMHRPPVGR

REPLITNIMWRNLLIQAIYQVTVLLVLNFDGKKILHLEHESREHAKRVKN

TLIFNAFVLCQIFNEFNARKPEEVNIFRGLSKNYLFIGIVAITIILQAII

VEFLGKFAKTEKLSWQLWLVSIGIGFISWPLAILGKFIPVPETPVSRVFS

RMFYRRRNQSSQKHEDSNATSIRN

>GhAHA23

MDTEKKAVALEAINKEIVDLENIPIEEVLEKLKCTKEGLTTDEVQQRLEL

FGYNKLEEKKENKILKFLGFMWNPLSWVMEAAAVMAIALAHGGFHPADAR

LLQGDPLKIDQSALTGESLPVTKHPGDGVYSGSTCKQGEIEAVVIATGVH

TFFGKAAHLVESTTHVGHFQQVLTSIGNFCICSIAIGMLAELIVIYGAQR

RSYRTGIDNLLVILIGGIPIAMPTVLSVTMAIGSHRLSQQGAITKRMTAI

EEMAGMDVLCSDKTGTLTLNKLTVDKNLIEVFVNNVDKDTVILMAARASR

LENQDAIDTAIVAMLADPKEARAGITEVHFLPFNPTDKRTALTYIDEAGK

MHRVSKGAPEQILNLAYNKSEIGKKVHSIIDKYAERGLRSLAVARQEVPA

GTKDSPGGPWEFVGLLPLFDPPRHDSAETIRRALDLGVSVKMITGDQLAI

GKETGRRLGMGTNMYPSSSLLGDHKNEEIGAFSIDELIENADGFAGVFPE

HKFEIVKRLQAKKHIVGMTGDGVNDAPALKKADIGIAVADSTDAARSASD

IVLTEPGLSVIISAVLTSRAIFQRMKNYTIYAVSITIRIVLGFMLLTLIW

RLNFPPFMVLIIAILNDGTIMTISKDRVKPSPVPDSWKLKEIFATGIVLG

SYLALMTVIFFWAAYETDFFPDKFGVRSLSKKSFDLKQESERRKVNALLS

SAVYLQVSTISQALIFVTRSRGWSFTERPGLLLVTAFIIAQLVATVISAQ

ATWSFAGIRAVGWGWCGVIWIYNILTYFLLDPIKFAVRYALSGKAWDLVL

NQRTAFSTQKDFGKEAREAAWAAEQRTLHGLQSTSDAKITEKHNFRDISV

MAEEARRRAEIARLREIHTLKGKVESFAKLRGLEIDVNPHYTV

>GhACA13

MSGTSSGNGLLHLNDVEAGLSKDNADLDHHLDPDAGTSDPFDIAHTKNAA

PETLKRWRQAALVLNASRRFRYTLDLKKEEEKEQRKRMIRAHAQVIRAAL

LFKLAGENQIVSSAPVASPSAGDDYKIGLEHLASMTRDHKLSALEQYGGV

KGLSGLLRTNLEKGIDEDEADLLNRRNAFGSNTYPRKKGRSFWMFLWEAW

QDLTLIILIIAAAVSLGLGIKTEGLKEGWYDGGSIFFAVFLVILVTATSD

YRQSLQFQNLNEEKRNIQLEVVRGGRTVKVSIYDLVVGDLVPLKIGDQVP

AHGVLIAGHSLAIDESSMTGESKIVHKNQNDPFLMSGCKVADGFGTMLVT

GVGINTEWGLLMASISEDTGEETPLQVRLNGVATFIGIVGLSVAVSVLAI

LLARYFTGNTEDPDGATQFIKGRTKFDDAFNDVVKIFTIAVTIVVVAVPE

GLPLAVTLTLAYSMRKMMADKALANTDTQVRRLSACETMGSATTICSDKT

GTLTLNEMTVVEAFVGKKKINPPADSSQLHLSVVSLLSEGVAQSTMGNVF

VSKDGGDVEISGSPTEKAILSWAIELGMKFDAIRSESTILHVLPFNSEKK

RGGVALRRDRNKDMNGRNICCPGLVDRSDAEVHIHWKGAAEIVLAACSGY

LDSNGCLQSMNEDKEFFEAAIDEMAANCLRCIALAYRLCEQEKVPSNEES

FDDWVLPEDNLVLLAIVGIKDPCRPGVKDAVKICTDAGVKVRMVTGDNIQ

TAQAIALECGILSSAQDVTEPTIIEGRVFRALTEIEREQVARKIMVMGRS

SPNDKLLLVQALRKGGDVVAVTGDGTNDAPALHEADIGLSMGIQGTEVAK

ESSDIIILDDNFASVVKVVRWGRSVYANIQKFIQFQLTVNVAALVINVAA

AISSGDVPLNSVQLLWVNLIMDTLGALALATEPPTDNLMHRSPVGRREPL

ITNIMWRNLLIQALYQVTVLLVLNFRGMSILQLEDDGNREHAYKVKNSLI

FNAFVMCQIFNEFNARKPEEVNCFKGVTKNYLFMGIIGFTFILQIIIIEF

LGKFTSTVRLDWQLWLVSLGIGIISWPLAIVGKLIPVPKTPVASYFIKPF

QQCKRSRDA

>GhECA3

MRIAALKTSTLRLEQSALTGEAMPVLKGTSPIFPEECELQAKENMVFAGT

TVVNGSCVCIVVCTGMNTEIGKIQKQIHEASLEESDTPLKKKLDEFGSRL

TTAIGLVCLIVWLINYKNFLSWDMVDGWPANLRFSFEKCTYYFKIAVALA

VAAIPEGLPAVITTSLALGTKKMAQKNAIVRKLPSVETLGCTTVICSDKT

GTLTTNQMSVAEFFTLGGKTTTSRIFHVEGTTYDPKDGGIVDWTCYNMDA

NLQVMAEICAVCNDAGIFCDGRLFRATGLPTEAALKVLVEKMGVPDAKMR

NKIHDSQLAANYLIDRSTIKLGCCEWWTKRSKRLATLELDTVRKSMSVIV

REPTGHNRLLVKGAVESLVERSTHVQLADGSLVPMDESCSQLLLSRNSEM

SSKGLRCLGLAYKDDLGEFSDYYSENHPAHKKLLDPASYSSIESDLVFVG

VVGLRDPPRDEVDKAIEDCKGAGIRVIVITGDNKSTAEAICHEIKLFSDG

EDVRGKSFTGKEFMALSPSQQIETLSKPGGKVFSRAEPRHKQEIVRMLKE

MGEIVAMTGDGVNDAPALKLADIGIAMGITGTEVAKEASDMVLADDNFST

IVSAVAEGRSIYNNMKAFIRYMISSNVGEVISIFLTAALGLPECMIPVQL

LWVNLVTDGPPATALGFNPPDVGIMWKPPRKSDDALIDSWVLFRYLTIGS

YVGVATVGIFILWYTQASFMGINLVSDGHTLIELSQLRNWGECSTWSNFC

VAPYTVGDGHLITFSNPCDYFTIGKVKAMTLSLSVLVAIEMFNSLNALSE

DSSLLTLPPWRNTWLLVAMSVSFGLHCLILYVPFLANMFGVVPLSLNEWF

LVILVSIPVVLIDETLKFFGRSRRHRVKEKTA

>GhECA4

MRIAALKTSTLRLEQSALTGEAMPVLKGTSPIFPEECELQAKENMVFAGT

TVVNGSCVCIVVCTGMNTEIGKIQKQIHEASLEESDTPLKKKLDEFGSRL

TTAIGLVCLIVWLINCKNFLSWDMVDGWPANLRFSFEKCTYYFKIAVALA

VAAIPEGLPAVITTSLALGTKKMAQKNAIVRKLPSVETLGCTTVICSDKT

GTLTTNQMSVAEFFTLGGKTTTSRIFHVEGTTYDPKDGGIVDWTCYNMDA

NLQVMAEICAVCNDAGIFCDGRLFRATGLPTEAALKVLVEKMGVPDAKMR

NEIHDSQLAGNYLIDRSTIKLGCWEWWTKRSKRLATLELDTLRKSMSVIV

REPTGHNRLLVKGAVESLVERSTHVQLADGSLVPMDESCSQLLLSRNSEM

SSKGLRCLGLAYKDDLGEFSDYYSENNPAHKKLLDPASYSSIESDLVFVG

VVGLRDPPRDEVDKAIEDCKGAGIRVIVITGDNKSTAEAICHEIKLFSDG

EDVRGKSFTGKEFMALSPSQQIETLSKPGGKVFSRAEPRHKQEIVRMLKE

MGEIVAMTGDGVNDAPALKLADIGIAMGITGTEVAKEASDMVLADDNFST

IVSAVAEGRSIYNNMKAFIRYMISSNVGEVISIFLTAALGLPECMIPVQL

LWVNLVTDGPPATALGFNPPDVGIMRKPPRKSDDTLIDSWVLFRYLTIGS

YVGVATVGIFILWYTQASFMGINLVSDGHTLIELSQLRNWGECSTWSNFS

VAPYTVGDGHLITFSNPCDYFTIGKVKAMTLSLSVLVAIEMFNSLNALSE

DSSLLTLPPWRNPWLLVAMSVSFGLHCLVLYVPFLANMFGVVPLSLNEWF

LVILVSIPVILIDETLKFFGRSRRHRVKEKTA

>GhACA4

MDSYLNQNFDLKSKHSSDEALEKWRTVVGFVKNPKRRFRFTANLSKRYEA

AAMRRTNQEKLRIAVLVSKAAFQFISGVQPSDYVVPEQVIAAGFQLCADE

LGSIVEGHDVKKLKFHGGVSGIAEKLSTSTNTGLSSDAALLSKRQEIYGI

NKFAEPEAKGFWVFVWEALQDMTLMILGACAIVSLVVGIAMEGWPAGAHD

GLGIVASILLVVFVTATSDYRQSLQFKDLDKEKKKITIQVTRNDCRQKLS

IYDLLPGDIVHLNIGDQVPADGLFVSGFSVLIDESSLTGESEPVVVNDKN

PFMLSGTKLQDGSCKMLVTTVGMRTQWGKLMATLSEGGDDETPLQVKLNG

VATIIGKVGLFFAVVTFAVMVQGLLRSKLQEGTIWSWSGDEALKLLEFFA

QVIARSSPMDKHTLVAKESADVIILDDNFSTILTVAKWGRSVYINIQKFV

QFQLTVNIVALIVNFSSACLTGSAPLTAVQLLWVNMIMDTLGALALATEP

PTDRVLQHADRCCFEAGPSMKPICLDFVKNIKEALMSLFVMYREEFLPAP

GIYMSLASTLFIV

>GhACA12

MSGFSSGDGRLQDLEAGPSKDNNDLNTNLDPDADTSDPFDIDQTKNATPQ

TLKRWRQAALVLNASRRFRYTLDLRKQEEKEQRKRMIRAHAQVIRAALLF

KLAGEKGIVSGTPVTLPGAAGDFAVGLEQLASMTRDHKLSALQQYGGVKG

LSDLLKTNLETGIYGDEVDLLNRKTAFGSNTYPRKKGRSFWRFLWEAWQD

LTLIILIVAASVSLGLGIKTEGLKEGWYDGGSIFLAVLLVIVVTATSDYR

QSLQFQNLNEEKRNIQLEVVRGGRIVKVSIYDVVVGDVVPLKIGDQVPAD

GVLVSGHSLAIDESSMTGESKIVYKDKNEPFLMSGCKVADGVGTMLVTGV

GINTEWGLLMASISEDTGEETPLQVRLNGVATFIGIVGLSVAVSVLVVLL

ARYFTGHTEDPDGTKQFIKGRTNFDDAFNDVVKIFTIAVMADALIWRCIG

LDLPLACLNSASQAAARVPFRCAATDRNVHGLCCLVFNCKLQQWTTIFGN

DVTIVVVAVPEGLPLAVTLTRDLQVRRLSACETMGSATTICSDKTGTLTL

NEMTVVEAFVGKKKINPPSDSSQLPASVVSLLNEGVAQNSTGSVFVPKDG

GNIEISGSPTEKAILSWAVKLGMKFDIIRSDSKILHVFPFNSEKKRGGVA

LQGACLLLYLAGRLGKELAKPWLLYYLHFADSEVRIHWKGAAEIVLTSCS

GYIDSNGCLQSIKEDKEFFKAAIDEMAVNSLRCVALAYRLCEKEKVPTDE

EGFNGWILPEENLVLLAIVGIKDPCRPGVKDAVKICMDAGVKVRMVTGDN

IQTAKAIALECGILSSAEDATEPTIIEGRVFRELSDKEREQISKKITVMG

RSSPSDKLLLVQALRKGGDVVAVTGDGTNDAPALHEADIGLSMGIQGTEV

AKESSDIIILDDNFASVVKVVRWGRSVYANIQKFIQFQLTVNVAALVINV

VAAISSGDVPLNSVQLLWVNLIMDTLVALALATEPPTDNLMHRSPVGRRE

PLITNIMWRNLLLQASYQVTVLLVLNFMGLTILHLKDDHDREHAYDVKNT

LIFNAFVMCQIFNEFNARKPEEINCFKGVTKNYLFMGIIGFTFVLQIIII

EFLGKFTKTVRLYWKLWLVSLGIGIVSWPLAIVGKLIPVPKTPVSGYFTK

AFRRCRTDGNA

>GhHMA13

MTGPRNFIKVIDSTGSSRRFKATIYPEGEGAGRESHRKEEIKQYFRSFLW

SLIFTTPVFLTSMVFMYIPGIKHGLDTKVVNMLTIGEVIRWVLSTPVQFL

IGRRFYTGSYKALRHGSANMDVLIALGTNAAYFYSCNAYLIHFTSAKRNG

DTVIGGTVNENGVLHIKATKVGSESALAQIVRLVESAQMAKAPVQKFADR

ISKYFVPLVIMLSFSTWLAWFLAGKLHGYPESWIPSSMDSFELALQFGIS

VMVIACPCALGLATPTAVMVGTGVGASLGVLIKDGQALEGAHKVNCIVFD

KTGTLTVGKPVVVNTRLLKNMVLHEFYELVAATEVNSEHPLTKAIIEYAK

KFREDEENPAWPEARDFVSITGHGVKAIVRNKEVIVGNKSLMLENNIVIP

VDAQDMLTETESMAQTGILVSIDGEVKGVLAISDPVKPGAQEVISILKSM

NVRSIMVTGDNWRTASSIASQFGIETVVAEAKPEQKAEKVKELQAEGYAV

AMVGDGINDSPALVAADVGMAIGAGTDIAIEAADIVLMKSNLDDVITAIH

LSRKTFSRIRLNYIWALGYNILGIPIAAGALFPSTGFRLPPWIAGAAMAA

SSVSVVCCSLLLKNYERPKKLENLEIGGIQIE

>GhHMA7

MVTGESVPVSKEVDSPVIGGTINLHGALHIKATKIGSEAVLSQIISLVET

AQMSKAPIQKFADFVASIFVPTVVTLSLITLLGWYAGGAAGAYPQQWLPE

NGNYFVFALMFSISVVVIACPCALGLATPTAVMVATGVGASNGVLIKGGD

ALERAQQMHRGEFLTLVASAEASSEHPLAKAIVEYARHFHFFDENSLTED

AEYSSKESPISAWLLDVAEFSAVPGRGIQCFIDGKRVLVGNRKLLTESGV

SISAHVEQFVVDLEESARTGILAAYDGNVIGVLGVADPLKREAAVVVQGL

QKMGVRPVMVTGDNWRTAQAVAREVGIRDVRAEVMPAGKAEVVRSFQKDG

SIVAMVGDGINDSPALAAADVGMAIGAGTDIAIEAADYVLMRNNLEDVIT

AIDLSRKTFSRIRWNYVFAMAYNVVAIPIAAGVLYPSLGIKLPPWAAGAC

MALSSVSVVCSSLLLRRYKKPRLTTILEITVE

>GhAHA30

MGTKTPNFATLPEMIFDVASGNDIRFNALDAFSDWKHEGLPPVEVLSTAQ

WKFRRTSRAGLTSEDAEVRVHIFGQNKLEEKPENKFLKFLSFMWNPLSWV

LETAAVMAIVLANGGGEGPDWQDFVGIICLLIINSTISFIEENNAGNAAA

ALMARLAPKTKVLRDGQWQETDAAILVPGDIISIKLGDIIPADARLLEGD

PLKIDQASALLDSDKSTTLIQCTSFSSKNKLKHPKQHNDKLKYLFYVVYK

SIVCDKTGTLTLNRLTVDRNLVEVFSKNMDKDLVVLLAARASRLENQDAI

DAAIINMLADPKEARANIKEVHFLPFNPVDKRTAITYIDSDEPKGRNFKG

ELRPPLGGLHTAACLCKSMWLTFS

>GhHMA8

MSPGSRDLQLTSQAAGVWRSTYPSSVRAVDPDDMEEGTRLLDSYETGDYK

LESIEEGSMRRIQVTVTGMTCAACSNSVEAALKNINGVLRASVALLQNRA

DVVFDPTLVKDEDIKNAIEDAGFEAEILPEPSNVGTKPRGVLVGQFTIGG

MTCAACVNSVEGILRDLPGVSRAVVALATSLGEVEYDPTVISKDDIVNAI

EDAGFEASLVQSSEQDKIILGVAGVFNELDVQLIEGILSSLKGVRQFRFD

RSSGELEVLFDPEVVSSRSLVDGIEGGSKGKFRLHVMNPYARMTTKDEET

SIMFQLFTSSLFLSIPVFLIRVVCPHIPLLDAFLLWRCGPFLMGDWLKWA

LVSVVQFVIGKRFYVAAGRALRNGSTNMDVLVALGTSASYFYSVGALLYG

AITGFWSPTYFETSSMLITFVLLGKYLECLAKGKTSDAIKKLVELAPATA

LLVVKDNGGNIIGEREVDALLVQPGDILKVLPGAKLPAXPNLFEVDSPVI

GGTINLHGALHIKATKIGSEAVLSQIISLVETAQMSKAPIQKFADFNMKL

KVGINILGAWGDSGREVGELLLEKLVASIFVPTVVTLSLITLLGWYVGGA

AGAYPEQWLPENGNYFVFALMFSISVVVIACPCALGLATPTAVMVATGVG

ASNGVLIKGGDALERAQKVQYVIFDKTGTLTQGKAKVTTVKVFSEMDRGE

FLTLVASAEASSEHPLAKAIVEYARHFHFFDENSLTEDAQYSSKGSPISA

WLLDVAEFSAVPGRGIQCFIDGKQVLVGNRKLLTESGVSISAHVEQFVVD

LEERARTGILAAYDGNVIGVLGVADPLKREAAVVVEGLQKMGVRPVMVTG

DNWRTAQAVAREVGIRDVRAEVMPAGKAEVVRSFQKDGSIVAMVGDGIND

SPALAAADVGMAIGAGTDIAIEAADYVLMRNNLEGVITAIDLSRKTFSRI

QWNYVFAMAYNVVAIPIAAGVLYPSLGIKLPPWAAGACMALSSVSVVCSS

LLLRRYKKPRLTTILEITVE

>GhAHA31

MGTKTPNFATLPEMIFGLTSEDAEVRVHIFGQNKLEEKPENKFLKFLSFM

WNPLSWVMEAAAVMAIVLANGGGEGPDWQDFVGIICLLIINSTISFIEEN

NAGNAAAALMARLAPKTKVLRYGQWQETDAAILVPGDIISIKLRDIIPAD

ARLLEGDPLKIDQASALLDSDKSTTLIQCTSFSSKNKLKHPKQHNDKLKY

LFYVVYKSIICDKTGTLTLNRLTVDRNLVEVFSKNMDKDLVVLLAARASR

LENQDAIDAAIINMLADPKEARANIKEVHFLPFNPVDKRTAITYIDSDEP

KGRNFKGELRPPLGGLHTAACLCKSMWLTFS

>GhACA6

MLSGTKLQDGSCKMMVTSVGMRTQWGKLMATLSEGGDDETPLQVKLNGVA

TIIGKVGLFFAVVTFAVMVQGLFTSKLQEGTIWSWSGDEALKLLEFFAVA

VTIVVVAVPEGLPLAVTLSLAFAMKKMMNDKALVRHLAACETMGSATNIC

SDKTGTLTTNHMTVVKSCICMDVREVGNNNKASLCSEIPESAVKLLLQSI

FTNTGGEIVINKDGKREILGTPTETALLEFGLSLGGDSQAERLASKLVKV

EPFNSTKKRMGVVLELPEGGLRAHTKGASEIVLAGCDKVINCNGEVIPLD

AESINHLNATINQFANEALRTLCLAYMELENGFSPDNAIPVSGYTCIGIV

GIKDPVRPGVKESVAICRAAGITVRMVTGDNINTAKAIARECGILTDDGI

AIEGPDFREKSQEEMLALIPKIQVMARSSPMDKHTLVRQLRSIDEVVAVT

GDGTNDAPALHEADIGLAMGISGTEVAKESADVIILDDNFSTIVTVAKWG

RSVYINIQKFVQFQLTVNIVALIVNFSSACLTGTAPLTAVQLLWVNMIMD

TLGALALATEPPTDELMKRAPVGKKGNFISNVMWRNILGQSFYQFMVIWY

LQVKGKGMFSLDGPDSDLKLNTIIFNSFVFCQVFNEISSRNMEEINVFKG

ILNNYVFVAVLGCTAVFQVIIIEFLGTFASTTPLTCLQWFVSVVIGFLGM

PIAAALKTIPV

>GhACA14

MSGTSSGNGLLHLNDVEAGLSKDNADLDHHLDPDAGTSDPFDIAHTKNAS

PETLKRWRQAALVLNASRRFRYTLDLKKEEEKEQRKRMIRAHAQVIRAAL

LFKLAGENQIVSGAPVASPSAGDDYKIGLEQLASMTRDHKLSALEQYGGV

KGLSGLLRTNLEKGIDEDEADLLNRRNAFGSNTYPRKKGRSFWMFLWEAW

QDLTLIILIIAAAVSLGLGIKTEGLKEGWYDGGSIFFAVFLVILVTATSD

YRQSLQFQNLNEEKRNIQLEVVRGGRTVKVSIYDLVVGDLVPLKIGDQVH

KNQNDPFLMSGCKVADGFGTMLVTGVGINTEWGLLMASISEDTGEETPLQ

VRLNGVATFIGVVGLSVAVSVLAILLARYFTGNTEDPNGATQFIKGRTKF

DDAFNDVVKIFTIAVTIVVVAVPEGLPLAVTLTLAYSMRKMMADKALANT

DTQVRRLSPCEPMASATTICSDKTGTLTLNEMTVVEAFVGKKKINPPAES

SQLHLSVVSLLSEGVAQSTTGNVFVSKLGMKFDAIRSESTILHVFPFNSE

KKRGGVALRRSDAEVHIHWKGAAEIVLAACSGYLDSNGCLQSMNEDKEFF

EAAIDEMAANCLRCIALAYRLCEQEKVPSNEESFDDWVLPEDNLVLLAIV

GIKDPCRPGVKDAVKICTDAGVKVRMVTGDNIQTAQAIALECGILSSAQD

VTEPTIIEGRVFRALTEKEREQVARKIMVMGRSSPNDKLLLVQALRKGGD

VVAVTGDGTNDAPALHEADIGLSMGIQGTEVAKESSDIVILDDNFASVVK

VVRWGRSVYANIQKFIQFQLTVNVAALVINVVAAISTGDVPLNSVQLLWV

NLIMDTLGALALATEPPTDNLMHRSPVGRRNIDVESSRQTVRGFSGEKEK

GFTLLFNVTALYQVTVLLVLNFRGMTILQLEDDGNREHAYKVKNSLIFNA

FVMCQIFNEFNARKPEEINCFKGVTKNYLFMGIIGFTFILQIIIIEFLGK

FTSTVRLDWQLWLVSLGIGIISWPLAIVGKLIPVPKTPVASYFIKPFQQC

KRSRDA

>GhACA22

MTGETGHTEVNRSQNPFLLSWTKVAHGDARMLVTSVGSNTTWGQISRETN

EQTPLQARLNKLSLSIAKVGLAVAFPVLLVLLVRYFTGHTKDEKGNREFH

RSKAKTSDIINAIVGIATTAITTVAEGLPLVVTLTLAYSMKKMVADQAMV

RKLSACETMGSTTTICTNKTGTLTLNRMEVTKFWLGQESMEEGASSISPF

VVDLIHQGVALNTTGSFYRASPGTEYEFSGSPTEKAILSWAVVELKMDVE

KTKKSCAVLQVEAFISQRRRSGVLIERNDDDTVHVHWKGAAEMILAMCSS

YYDASGVVKDLDDGERTKFEEIIQGMAASSLRCIAFAHKQVPEEEYQNLK

EQKKLKEDSLALLGLVGIKDPCRPGVKKAVEDCQYAGVNIKMITGDNVFT

ARAIATECGILKPGQDLSSGAVVEGEEFRKYTLQERMEKVEKIQVMARSS

PFDKLLMVQCLKQKGHVVAFTGDGTDDAPALKEADIGLSMGIQGTEAAKE

SSDIVILDDSFASVATVLRWGRCVYTNIQKFIQFLLTVNVSALCINFIAA

VSTGEVPLTTVQLLWVNLYMDTLGALALATELPTKELLEKPPVGRTEPLI

ANIMWRNLLAQALYQIAVLLTLQFNGESIFGVTKKAYCSSHWGWHKCCTT

TPAHREADICLFMGIQGTEVAKESSNIVILEDDFASVATVLRWGEDVSIA

ITAECEILKLDKDMSSCVVVEGEEFRNYNP

>GhHMA10

MDKAPVQKFADRISKYFVPLVIMLSFSTWLAWVLAGKLHGYPESWIPSSM

DSFELALQFGISVMVIACPCALGLATPTAVMVGTGVGASLGVLIKGGQAL

EGAHKVNCIVFDKTGTLTVGKPVVVNTRLLKNVVLHEFYELVAATEANSE

HPLAKAIIEYAKKFREDEENPAWPEARDFVSITRHGVKAIVRDKEIIVGN

KSLMLENNIVIPVDAQDMLTETESMAQTGILVSIDGEVTGVLAISDPVKP

GAQEVISILKSMNVRSIMVTGDNWGTASSIASQIGIETVVAETKPEQKAE

KVKELQAEGYAVAMIGDGINDLPALEAADVGMAIGAGTDIAIEAADIVLM

KSNLEDVITAILLSKKTFSHICLNYIWAFGYNMLGTPIAAESLFPPTRFR

LPLWIAGAAMVASSVSVVCC

>GhACA1

MFAGASNIGGCNEHGDEDTLSVSLLNTTANSSRKNWTRLYNLFLVVGSRI

KSGREELSIEEDGSVPKSSSNSGSADCCTIDMVPTEIKEFFIEEDGSVPK

SSSTSGSADCSTNDMFAPKIEVFPKEDGSVPSIIFLRKAAQIVRDKDLTS

LEEEGGVRRVKSRLQSILAHEIDRSFEEQATAVIVTGTTFDAKVFVVSFV

EASKTPTIFPLLVFAALSFTIEMMEAEPKHGWHDGVAILVAVFMLLIFRS

VANYRRARRLQKRKKFHVSVLKDGQPKTITISTLVVGHILCLKKGDFVPA

DGLFVSENGLKLDDKLNPNINRDDNPFLLAGSKIMEGEGHMLVTSVGDNS

VLPTVDPDEKSLMEDQIDKTNAYMEYVGLSISLLVSALVLINLLARKMDK

NSNIMPEMKGDVSAYRVIKIFARVFLNPRGKVQIFTGVLTVMVTSLQHGM

PVVITISLSYWKKKMALGDANVQSLSSCGTIGIVSVICFDENTVVACKEV

MGSIVGALKEEEVGCKLMSKDELATAQVMAHEIGILNPDLRDMAIENKDL

HELAATAEGMNKIAVIGSCKLKDKIRILQRLKQEGHVVAFIGGMATNDDL

ALKAADVGITICECSTKMARENSEIVISSRNSLRSLIRCLKMGKCAYGNV

RTFTEIQLTATLASLLVTLVTTSILDESPITGIHMLWVNSIIFILGGLMM

VMESYGHQELMNNQPARRMKSLLTKTMWRNVAIRAASDACHLLVLQFIGQ

AILQINKDVVKTMVFNGFILCQVLDLFISSIIIFARNKEVSVSVVRFMCS

SHWFLMASGGVMAMQVVVVELLQSLAEYERLNVMQWGFCFIYAAWLCGTG

LTVKLIADSASEVLWSSRSSGSLFGYLRLRRSSLRLFVIPFSVCVVACFS

YCYVNPDIA

>GhHMA11

MDKAPVQKFADRISKYFVPLVIMLSFSTWLAWFLAGKLHGYPESWIPSSM

DSFELALQFGISVMVIACPCALGLATPTAVMVGTGVGASLGVLIKGGQAL

EGAHKVNCIVFDKTGTLTVGKPVVVNTRLLKNVVLHEFYELVAATEANSE

HPLAKAIIQYAKFREDEENPAWPEARDFVSITRHGVKAIVRDKEIIVGNK

SLMLENNIVIPVDAQDMLTETESMAQTGILVSIDGEVTGVLAISDPVKPG

AQEVISILKSMNVRSIMVTGDNWGTASSIASQIGIETVVAEAKPEQKAEK

VKELQAEGYAVAMVGDGINDSPALVAADVGMEIGAGIDIAIEAADIVLMK

SNLEDVITAIYLSRKTFSRIRLNYIWAFGYNMLGIPIATGSLFPPTGFRL

PPWIAGAAMAASSVSVVCCSLLLKNYKRPKKLENLDTGGIHIE

>GhACA2

MVPTEIEEFFIEEDGSVPKSSSTSGSADCSTNDMFAPKIEVFPKEDGSVP

SIIFLRKAAQIVRDKDLTSLEEEGGVRRVKSRLQSILAQEIDRSFEEQAT

AVIVTSTTFDAKVFVVSFVEASKTPTIFLLLVFAALSFTIEMMEAEPKHG

WHDGVAILVAVFMLLIFRSVANYRRARRLQKRNKFHVSVLKDGQPKTITI

STLAVGHILCLQKGDFVPADGLFVSENDLKLDDILNPNINRDDNPFLLAG

SKIMEGEGHMLVTSVGDNTVLAIVDPDEKSLMEGQIDKTNACMEYVGLSI

SLLVSALVLINLVVRKMDKNSNTMPEIKGDVSAYRVIKIFARVFLNPRGK

VQILTGVLTVMVTSLQHGIPVVITISLWYWKKKMSSGDANVQNLSSCGTI

GIVSVMCFDENTVVACKEVMESIVGTLKEEEVGCKLMSKDELPTAQAMAR

EIGIQNPDLRDMAIENKDLHELAATAEGMNKIAVIGSCKLEDKIRILQRL

KQEGHVVAFIGGMATNDDLALKAADVGITICECSTKMARENSEIVISSRN

SLRSLIRCLKMGKCAYGNVQTFNQIQLTAILASLLVTLVTTSILDESPIT

GIHMLWVNSIICILGGLMMVMESYGHQELMNNQTVRRMKSLLTKTMWRNV

AIRAASDACHLLLLQFIGQAILQINKDVVKTMVFNGFVLCQVLDLFISTI

IIFARNEEVSVSVVRFMCSRHWFLMASGGVMAMQVVVVELLQSLAEYERL

NVMQWGFCFIYAAWMCGTGLTVKLIADSASEVLWSSRSSGSQFGYLRLRR

SNIRLFVIPFSACVVASFSYYYVNPDNA

>GhACA23

MLVTSVGMNTTWGQISRETNEQTPLQARLNKLSSSIAKVGLAVAFPVLVV

LLVRYFTGHTKDEKGNREFNRSKTKTSDIINAIVGIATTAITTAAEGLPL

VVTLTLAYSKKKMVADQAMVRKLSACETMASTTTICTAKTGTLTLNRMKV

TKFWLGQESMEEGASSISPFVVDLIHQGVALNTTGSFYRASPGTEYELSG

SPTEKAILSWAVVELKMDVEKTKKRCAALQVEAFIPQKRRSGVLIERNDD

DTVHVHWKGAAEMILAMSSSYYDASGVVKDLDDGERMKFEEIIQGMAASS

LRCIAFAHKQVPEEEYQNLKEQKKLKEDSLTLLGLVGIKDPCRPGVKKAA

EDCQYAGVNIKMITGDNVFTAIAIATECGILKPGQDLSSGAVVEGEEFRN

YTSQERMEKVEKIQVMAGFSPFDKLLMVQCLKQKGHVVAVTGDGTDDAPA

LKEADIGLSTGIQGTEAARESSDIVILDDSFASVATVLRWGRCVYTNIQK

FIQFLLTANVATLCINFIAAVSTGEVPLTTVQLFWVNLYMDTLGALALAT

ERPTKELLEKPPVGRTEPLITNIMWRNLLAQALYQIAVLLTLQFNGESIF

GVTKKVNHTLIFNTFMLCQVFNKFNARKLEKKNVFNDMHKNKMFIGITGV

TIVLQVVMVEFLKRFTDTERLNWGQWGACIAIAAASCPLGWAVKCLPVPK

KPILSYLKWNVAAQREADIGLSMGIQGTEVAKESSNIVILEDDFASVATV

LRWGKMSVYQHPKIHSFPAHFKCTTLYKLHSSKVLLDLGVIFRYKSLSNV

DSRPTAECEILKPDKDMSSCVVVEGKEFRNYTPEERMEKVEKIQVMARSS

PSDKLLTVQCLKQKGQVIAVTGDGINDAPALKGADIGLSMGIQGTEVAKE

SSDIIILDDNFASVAIVLWWGKCIYTNIQNFIQFLLTVNVAALCINFIAA

MSAGEIPLTTVQLLWGNLYMNTFAALSLVPERPTKELMAKPPISRTEPLI

SNIVWRTILAQALYQIAPYNSMVNQSLG

>GhALA1

MNQYFLLIACLQLWSLITPVNPASTWGPLIFIFAVSASKEAWDDYNRYLS

DKKANEKLVWVVRQGIRKHIQAQDIHVGNIVWLRENDEVPCDLVLIGTSD

PQGLCYVETAALDGETDLKTRGVIECPNPDKDITRFDANLRLFPPFIDND

FLNYLFHLSGNETKLGMSRGIPEPKLTAMDAMIDKLTGAIFVFQIVVVMV

LGIAGNVWKDTEARKQWYVQYPIEGPWYELLVIPLRFELLCSIMIPISIK

VSLDLVKSLYAKFIDWDAEMIDYETGIPSHATNTAISEDLGQVEYIMTDK

TGTLTENRMIFRRCCISGVFYGNESGDALKDTKLLNAVAGSSPDVVQFLT

VMAICNTVVPIKSKTGAISYKAQSQDEDALVNAAAQLHMVYANKNANILE

IRFNGSVIKYEVLEILEFTSDRKRMSVVVKDCQNGKIVLLSKGADEAILP

FAYVGQQTRTFIEAVEQYAQLGLRTLCLACRELREDEYQEWSLLFKEASS

TLVDREWRIAEVCQRLEHDFEVLGVTAIEDHLQDGVPETIETLRKAGINF

WMLTGDKQNTAIQIALSCNFISPEPKGQLLLIDGKTEDEVCRSLERVLLT

MRITSSEPKDVAFVVDGWALEIALKHYRKAFTELAILSRTAICCRVTPSQ

KAQLVELLKSCDYRTLAIGDGGNDVRMIQQADIGVGISGREGLQAARAAD

YSIGSVSGTSLFNSVSLMAYNVFYTSIPVLVSVLDKDLSEGTVMQHPQIL

FYCQAGRLLNPSTFAGWFGRSLFHVSAILYQSILTDTGCKAIVVFVITIH

AYAYEKSEMEELSMVALSGCIWLQAFVVALETNSFTILQHLAIWGNLVAF

YVINWIFSAIPASGMYTIMFRLCRHPSYWITMSLIVVAGMGPVLALKYFR

YTYRPSKINTLQQAERMGGPILTLGNIEPHPRPMEKEVVSPLQISQPKNR

NPVYEPLLSDSPNSSRRSLGSGTPFDFFQSQSRLSSSYSRNCKDN

>GhALA2

MNQYFLLIACLQLWSLITPVNPASTWGPLIFIFAVSASKEAWDDYNRYLS

DKKANEKLVWVVRQGIRKQIQAQDIHVGNIVWLRENDEVPCDLVLIGTSD

PQGLCYVETAALDGETDLKTRFLNYLFRLSGNETKLGMSRGIPEPKLTAM

DAMIDKLTGAIFVFQIVVVMVLGIAGNVWKDTEARKQWYVKYPIEGPWYE

LLVIPLRFELLCSIMIPISIKVSLDLVKSLYAKFIDWDAEMIDYETGIPS

HATNTAISEDLGQVEYIMTDKTGTLTENRMIFRRCCISGVFYGNESGDAL

KDMKLLNAVAGGSPDVVQFLTVMAICNTVVPIKSKTGAISYKAQSQDEDA

LVNAAAQLHMVYANKNANILEIRFNGSVIKYEVLEILEFTSDRKRMSVVV

KDCQTGKIVLLSKGADEAILPYAFVGQQTRTFIEAVEQYAQLGLRTLCLA

CRELREDEYQEWSLMFKEASSTLVDREWRIAEACQRLEHDFEVLGVTAIE

DRLQDGVPETIETLRKAGINFWMLTGDKQNTAIQIALSCNFISPEPKGQL

LLIDGKTEDEVCRSLERVLLTMRITSSEPKDVAFVVDGWALEIALKHYRK

AFTELAILSRTAICCRVTPSQKAQLVELLKSCDYRTLAIGDGGNDVRMIQ

QADIGVGISGREGLQAARAADYSIGSVSGTSLFNSVSLMAYNVFYTSIPV

LVSVLDKDLSEGTVMQHPQILFYCQAGRLLNPSTFAGWFGRSLFHAIVVF

VISIHAYAYEKSEMEELSLVALSGCIWLQAFVVALETNSFTILQHLAIWG

NLVAFYVINWIFSAIPASGMYTIMFRLCRQPSYWITMSLIVAAGMGPVLA

LKYFRYTYRPSKINTLQQAERMGGPILTLGNIEPHPRPMEKEVVSPLQIS

QPKNRNPVYEPLLSDSPNSSRRSLGSGAPFDFFQSQSRLSSSYSRNCKDN

>GhALA3

MDSRTSFEDFYSIESAFSSSSRRSNFSVQSKASGGNSIREVNFGDLGTKP

VRYGSHGADSETYSISMSQKEINDEDARLVHINDPVQTNERFEFSGNSIR

TGKYSILTFLPRNLFEQFHRVAYIYFLLIAVLNQLPQLAVFGRGASILPL

AFVLLVTAVKDAYEDYRRHRSDRIENNRLASVLVDDQFQEKKWKNIQVGE

IIKIYANETIPCDMVLLSTSDPTGVAYVQTINLDGESNLKTRYAKQETLM

KIPENDEVIGLIKCEKPNRNIYGFQANMEVDGKQLSLGPSNIILRGCELK

NTAWAVGVAVYAGRETKAMLNSSGAPSKRSRLETHMNLEIIFLSLFLIAL

CTVVSICAAAWLRHHRKELDYLPFYRRKEFSDGEEENYNYYGWGLEICFT

FLMSVIVFQIMIPISLYISMELVRVGQAYFMIRDTRMYDESSKSRFQCRA

LNINEDLGQIKYVFSDKTGTLTENKMEFQCASIWGVDYSGGNAISLDQND

GYFVKVDGKVLRPKMKVKTDPELLQFARNGKETQEGSHVYDFFLALAACN

TIVPLIVDTPDPTVKLIDYQGESPDEQALVYAAASYGFMLIERTSGHIVI

DIQGERQRASLLRKVANNIENNLHILGASGIEDKLQQGVPEAIESLRTAG

IKVWVLTGDKQETAISIGYSSKLLTSKMTQIIINSKSMESCRKSLEDAII

MSKKLTTTSAISGTTNNTGGTSGAGSTPIALIIDGTSLVYILDSELEERL

FQLSCNCSVVLCCRVAPLQKAGIVSLVKKRTADMTLAIGDGANDVSMIQM

ADVGVGISGQEGRQAVMASDFAMGQFRFLVPLLLVHGHWNYQRMGYMILY

NFYRNAVFVLVLFWYVLFTSFTLTTAITEWSSVLYSVIYTALPTIVVGIL

DKDLSRRTLLKYPQLYRAGQKQECYNKKLFWITMIDTFWQSTVTFFIPLL

AYWESTIDASSIGDLWTLAVVILVNLHLAMDVNRWNWLTHAAIWGSIIAT

FICVIVIDALPFLVGYWAIFEIAKTGLFWLCLLAIIVAALIPHFVVKALY

QLYAPCDVQITREAEKFRTLCESGAVEIEMNSILEVPRR

>GhALA4

MDSRTSFEDFYSIESAFSSSSRRSNFSVQSKASGGNSIREVNFGDLGTKP

VRYGSHGADSETYSISMSQKEINDEDARLVQINDPVQTNERFEFSGNSIR

TGKYSILTFLPRNLFEQFHRVAYIYFLLIAVLNQLPQLAVFGRGASILPL

AFVLLVTAVKDAYEDYRRHRSDRIENNRLASVLVDDQFQEKKWKNIQVGE

IIKIYANETIPCDMVLLSTSDPTGVAYVQTINLDGESNLKTRYAKQETLM

KIPENDKVIGLIKCEKPNRNIYGFQANMEVDGKQLSLGPSNIILRGCELK

NTAWAVGVAVYAGRETKAMLNSSGAPSKRSRLETHMNLEIIFLSLFLIAL

CTVVSICAAVWLRRHRKQLDYLPFYRRKEFSDGEEENYNYYGWGLEICFT

FLMSVIVFQIMIPISLYISMELVRVGQAYFMIRDTQMYDESSNSRFQCRA

LNINEDLGQIKYVFSDKTGTLTENKMEFQCASIWGVDYSGGNAISLDQND

GYFVKVDGKVLRPQMKVRTDPELLQFARNRKETQEGSHVYDFFLALAACN

TIVPLIVDTPDPTVKLIDYQGESPDEQALVYAAASYGFMLIERTSGHIVI

DIQGERQSIHTYARSMFLVCTSLIVIGSKQWHSTFEAASTALIGRASLLR

KVANNIENNLHILGASGIEDKLQQGVPEAIESLRTAGIKVWVLTGDKQET

AISIGYSSKLLTSKMTQIIINSKSMESCRKSLEDAIIMSKKPTTTSAISG

TTNNTGGTSGAGSTPIALIMDGTSLVYILDSELEERLFQLSCNCSVVLCC

RVAPLQKAGIVSLVKKRTADKTLAIGDGANDVSMIQMADVGVGISGQEGR

QAVMASDFAMGQFRFLVPLLLVHGHWNYQRMGYMILYNFYRNAVFVLVLF

WYVLFTSFTLTTAITEWSSVLYSVIYTALPTIVVGILDNDLSRRTLLKYP

QLYRAGQNQECYNKKLFWITMIDTFWQSAVAFFIPLLAYWGSTIDTSSIG

DLWTLAVVILVNLHLAMDVNRWNWLTHAAIWGSIIATFICVMVIDALPFL

VGYWAIFEIAKTGLFWLCLLAIIVAALIPRFVVKALYQLYAPCDVQIARE

AEKFRTLCESGAVEIEMNSILEVPRR

>GhALA5

MDSKTPVENLYSIEPALSSSSRRSNFSARSKSLGGSSIREVNFSDVGPQP

VRYGSQGAESDTYSMSQKEINDEDARLVHINDPVNTNERFKFAGNSIRTA

KYSILTFLPRNLFEQFHRVAYIYFLVIAVLNQLPQLAVFGRTASILPLAF

VLLVTAVKDAYEDYRRHRADRIENNRLALVLVNNEFQQKKWKNIQVGEII

KIQANETIPCDMVLLSTSEPTGVAYVQTINLDGESNLKTRYAKQETLQKI

PEKENVSGLIKCEKPNRNIYGFQANMEVDGKRVSLGPSNIILRGCELKNT

TWAVGVVVYAGSETKAMLNNSGAPSKRSRLETHMNLEIIFLSLFLVALCT

VVSVCAAVWLRRHRDELDYLPFYRRKDFSEDEEKNYNYHGWGLEIFFTFL

MSVIVFQIMIPISLYISMELVRVGQAYFMIRDAEMYDESSNTRFQCRALN

INEDLGQIKYVFSDKTGTLTENKMEFQCASIWGVDYNGGKATSQDQKDGY

FVQADGQVLRPKMVVKTDPELLQFVRNGKETKEGSYVHDFFLALAACNTI

VPIIVDTPDPTLRLIDYQGESPDEQALVYAAAAYGFMLIERTSGHIVIDI

QGERKSNLCILGASGIEDKLQQGVPEAIESLRTAGIKVWVLTGDKQETAI

SIGYSSKLLTSKMTQVIVNSNSKESCRKSLEDAIIMSKKLTTMSGTTNET

GRTLGSGSTPVALIIDGTSLVYILDSELEERLFELACNCSVVLCCRVAPL

QKAGIVSLVKKRTSDMTLAIGDGANDVSMIQMADVGVGISGQEGRQAVMA

SDFAMGQFRFLVPLLFVHGHWNYQRMGYMILYNFYRNAVFVLVLFWYVLF

TGFTLTTAINEWSSVLYSVIYTSVPTIVVGILDKDLSRLTLLKHPQLYGA

GHRDECYNKTLFWMTMLDTLYQSVVVFFIPLLSYWGSTIDASSIGDLWTL

AVVILVNLHLAMDVIQWNWITHAAIWGSIIATFICVIIIDAIPSLVGYWA

IFEIAKTRLFWCCLLAIIVTALIPRFVVKVLYQFYAPCDVQIAREAEKFW

AQNQSAAVEVEMSPILDHQRR

>GhALA6

MEGKLYCLHLILPLWWCITKVLLLAASCLPLAASAATDFLYLEDQQEELY

SADCLSRQQFSLECPAKDSGNQVSWGSMELHNNDHTYTSFDISRSSSQAQ

ENLSKSRRIRNKSVDFDDNLPYSENPRLIYINDPRRTNDKYEFTGNEIRT

SKYTLITFLPKNLFIQFHRVAYLYFLAIAALNQLPPLAVFGRTVSLFPLL

FVLCVTAIKDGYEDWRRHRSDRNENNREALVLQVGEFQMKKWKKIRAGEV

VKIHADETIPCDMVLLGTSDPSGLAYIQTMNLDGESNLKTRYARQETASS

IFEGCNVSGLIRCEQPNRNIYEFTANMEFNGHKFPLSQSNIVLRGCQLKN

TDWIIGVVVYAGQETKAMLNSAVSPSKRSKLEGYMNRETFWLSIFLLVMC

SVVAVGMGLWLHRQKDELDTLPYYRKTYIREGRENGKTYRYYGIPMETFF

SFLSSVIVFQIMIPISLYITMELVRLGQSYFMIEDKHMYCSNSGSRFQCR

SLNINEDLGQIRYVFSDKTGTLTENKMEFRKASVYGKDYRSSNLTDDSVQ

DNSITDAAVPSRWKLKSEISVDSELMDLLHKDLAGDERIAAHLFFLTLAA

CNTVIPIVSQDASSSHGSSDSWGEIKAIDYQGESPDEQALVSAASAYGYT

LHERTSGHIVIDINGDKLRLDVLGLHEFDSVRKRMSVVIRFPDNTVKVLV

KGADSTMFSILADTEKVDQIRQATRSHLTEYSSEGLRTLVVAARDLTDAE

LEQWQCRYEDASTSLIDRAAKLRQTAALVECNLKLLGATAIEDKLQDGVP

EAIESLRQAGIKVWVLTGDKQETAISIGLSCKLLTADMQQIIINGNSEEE

CRNLLTDAMTRHGVQPANRKKQNSKRRKDSENGYLEIPDDTKTSNVLQRC

SGKEEPDVCAPLALIIDGNSLVYILEKDLQSELFDIATSCKVVLCCRVAP

LQKAGIVDLIKSRTDDMTLAIGDGANDVSMIQMADVGVGICGQEGRQAVM

ASDFAMGQFRFLKRLLLVHGHWNYQRVGYLVLYNFYRNAVFVLMLFWYIL

CTAFSTTSALTDWSSVFYSVIYTSVPTIVIGILDKDLSHKTLLEYPKLYG

VGHRHEAYNLQLFWITMIDTLWQSLVLFYIPLFTYKESTIDIWSMGSLWT

IAVVILVNIHLAMDIQRWVFITHAAVWGSIIITYACMVVLDSIPVFPNYW

TIYHLVKSPTYWLTILLIIIVALLPRFLFKVIHQIFWPSDIQIAREAEIL

RKVTPNLRSKPDEDSS

>GhALA7

MELHNNDNTYTSFDISRSSSQAQENLSKSRRIRNKSVDFDDNLPYSENPR

LIYINDPRRTNDKYEFTGNEIRTSKYTLITFLPKNLFIQFHRVAYLYFLA

IAALNQLPPLAVFGRTVSLFPLLFVLCVTAIKDGYEDWRRHRSDRNENNR

EALVLQVGEFQRKKWKKIRAGEVVKIHADETIPCDMVLLGTSDPSGLAYI

QTMNLDGESNLKTRYARQETASSIFEGCNVSGLIRCEQPNRNIYEFTANM

EFNGHKFPLSQSNIVLRGCQLKNTDWIIGVVVYAGQETKAMLNSAVSPSK

RSKLEGYMNRETFWLSIFLLVMCSVVAVGMGLWLHRHKDELDTLPYYRKT

YIREGRENVRLGQSYFMIEDKHMYCSNSGSRFQCRSLNINEDLGQIRYVF

SDKTGTLTENKMEFRKASVYGKDYRSSNLTDDSLQDNSITDAAVRSRWKL

KSEISVDSELMDLLHKDLAGDERIAAHLFFLTLAACNTVIPIVSQDASSS

HGSSDSSGEVKTIDYQGESPDEQALVSAASAYGYTLHERTSGHIVIDING

NKLRLDVLGLHEFDSVRKRMSVVIRFPDNTVKVLVKGADSTMFSILADTE

KVDQIRQATRSHLTEYSSEGLRTLVVAARDLTDAELEQWQCRYEDASTSL

IDRAAKLRQTAALVECNLKLLGATAIEDKLQDGVPEAIESLRQAGIKVWV

LTGDKQETAISIGLSCKLLTADMQQIIINGNSEEECRNLLTDAMTRHGVQ

PANRKKQNSTRRKNSENGYLEIPDDTKSSNVLQQHSGKEEPDVCAPLALI

IDGNSLVYILEKDLQSELFDIATSCKVVLCCRVAPLQKAGIVDLIKSRTD

DMTLAIGDGANDVSMIQMADVGVGICGQEGRQAVMASDFAMGQFRFLKRL

LLVHGHWNYQRVGYLVLYNFYRNAVFVLMLFWYILCTAFSTTSALTDWSS

VFYSVIYTSVPTIVVGILDKDLSHKTLLEYPKLYGVGHRHEAYNLQLFWI

TMIDTLWQSLVLFYIPLFTYKESTIDIWSMGSLWTIAVVILVNIHLAMDI

RRWVFITHAAVWGSIIITYACMVVLDSIPVFPNYWTIYHLVKSPTYWLTI

LLIIIVALLPRFLFKVIHQIFWPSDIQIAREAEILRKVAPNLRSKPDEDS

S

>GhALA8

MSGWGTPSRTVTLGRVQPQAPALRTIYCNHREANFAHRYKGNSISTTKYN

VFTFLPKGLYEQFRRVANLYFLMVSILSATPYSPVHPITNMVPLSLVLLF

SLIKEAFEDWQRFQNDMTINNTLVDVLQAQGWESLQWKKLQVGDIIRVKQ

DGFFPADLLLLASTNVDGICYIETANLDGETNLKIRKALERTWDYVTPEK

ACEFKGEVQCEQPNNSLYTFTGNLVMDNQTMPLSPNQILLRGCSLKNTDY

IVGTVIFTGHETKVMMNSMNVPSKRSTLERKLDKLILALFSTLFSMCLLG

AIGSGIFIDRKYYYLGLSQSVEDQFNPSKRFLVIILTMLTLLTLYSTIIP

ISLYVSIETIKFIQSSQFINKDLNMYHAESNTPASARTSNLNEELGQVEY

IFSDKTGTLTRNLMEFFKCSIGGEMYGTGMTEIEMGVAERKGIKVQEAKT

STNSLREKGFNFDDVRLMQGAWRNEPNHDACKEFFRCLAICHTVLPEGEE

TPEKIRYQAASPDEAALVFAAKNFGFFFYRRTPTMIYVRESHVEKMGKTQ

DVSYEILNVLEFNSTRKRQSVVCRYRDGRLVLFCKGADTVIYERLGTGNG

DLNKVTRVHLEQFGSAGLRTLCLAYRDLAPDLYESWNEKFITAKSSLRDR

EKRLDEVAELIEKELILIGATAIEDKLQEGVPNCIQTLSRAGIKIWVLTG

DKIETAINIAYACNLLNNEMKQFIISSETDAIREVEERGDQTEIARFMKE

EVKKQLKQFLDEAPQYFCSPSGPKLALVIDGKCLMYALEPSLRIMLLTLS

LNCSSVVCCRVSPLQKAQVTSLVKKGARKITLSIGDGANDVSMIQAAHIG

IGISGLEGMQAVMASDFAIAQFRFLEDLLLVHGRWSYIRLCKVVMYFFYK

NLAFTLTQFWFTFGTGFSGQRFYDDWFQSLYNVIFTALPVVIVGLFDQDI

SSSLSKKYPELYKEGVRNMFFNRRVVAIWACFAVYQSLVFFYFVSLSSST

SRDSDGKMFGLWDVSTMAFTCVVVTVNLRLLMMCNSITRWHYISVGGSII

VWFVFIFIYSGIMTRFDRNDNMFWVIYVLMSTSYFYVTLLLVPVAALLGD

FLYLGVQRWFFTYDYQIVQESHKDDADSINKPDSQETKEHLTPDEARSNE

ISQLPKEVSKHSGFAFDSPGYESFFATQFGKYVPPKAWDVARRASMKSKS

KPK

>GhALA9

MSGWGTPSRTVTLGRVQPQAPALRTIYCNHREANFAHRYKGNSISTTKYN

VFTFLPKGLYEQFRRVANLYFLMVSILSATPYSPVHPITNMVPLSLVLLF

SLIKEAFEDWQRFQNDMTINNTLVDVLQAQGWESLQWKKLQVGDIIRVKQ

DGFFPADLLLLASTNVDGICYIETANLDGETNLKIRKALERTWDYVTPEK

ACEFKGEVQCEQPNNSLYTFTGNLVMDNQTMPLSPNQILLRGCSLKNTDY

IVGTVIFTGHETKVMMNSMNVPSKRSTLERKLDKLILALFSTLFSMCLLG

AIGSGIFIDRKYYYLGLSQSVEDQFNPSKRFLVIILTMLTLLTLYSTIIP

ISLYVSIETIKFIQSSQFINKDLNMYHAESNTPASARTSNLNEELGQVEY

IFSDKTGTLTRNLMEFFKCSIGGEMYGTGMTEIEMGVAERKGIKVQEAKT

STNSLREKGFNFDDVRLMQGAWRNEPNHDACKEFFRCLAICHTVLPEGEE

TPEKIRYQAASPDEAALVLAAKNFGFFFYRRTPTMIYVRESHVEKMGKTQ

DVSYEILNVLEFNSTRKRQSVVCRYRDGRLVLFCKGADTVIYERLGTGNG

DLNKVTRVHLEQFGSAGLRTLCLAYRDLAPDLYESWNEKFITAKSSLRDR

EKRLDEVAELIEKELILIGATAIEDKLQEGVPNCIQTLSRAGIKIWVLTG

DKIETAINIAYACNLLNNEMKQFIISSETDAIREVEERGDQTEIARFMKE

EVKKQLKQFLDEAPQYFCSPSGPKLALVIDGKCLMYALEPSLRIMLLTLS

LNCSSVVCCRVSPLQKAQVTSLVKKGARKITLSIGDGANDVSMIQAAHIG

IGISGLEGMQAVMASDFAIAQFRFLEDLLLVHGRWSYIRLCKVVMYFFYK

NLAFTLTQFWFTFGTGFSGQRFYDDWFQSLYNVIFTALPVVIVGLFDQDI

SSSLSKKYPELYKEGVRNMFFNRRVVAIWACFAVYQSLVFFYFVSLSSST

SRDSDGKMFGLWDVSTMAFTCVVVTVNLRLLMMCNSITRWHYISVGGSII

VWFVFIFIYSGIMTRFDRNDNMFWVIYVLMSTSYFYVTLLLVPVAALLGD

FLYLGVQRWFFPYDYQIVQESHKDDADSINKPDSQETKEHLTPDEARSNE

ISQLPKEVSKHSGFAFDSPGYESFFATQFGKYVPPKAWDVARRASMKSKS

KPK

>GhALA10

MAKGKIRAKIKRSQLYTFACGRKSTQEAVPFVEGAGCSRTVHCNQPHMHR

KRPLKYRSNYISTTKYNFLSFLPKSLYEQFHRVANLYFLAAAVLSLTPLS

PFSAVSMIAPLAFVVGLSMAKEGLEDWRRFMQDMNVNSRKVKFHKEGGVF

EFKQWQQVEVGDVVRVEKDEFFPADLLLLSSSYEDGICYVETMNLDGETN

LKVKRAMDVTLSLEEDEDFKNFTGLIKCEDPNPSLYTFVGNLDYDRQTYS

LEPAQVLLRDSKLRNTGFVYGVVIFTGFDTKVMQNSTKSPSKRSRIEKKM

DYIIYVLFSLLLVISLISSLGFALKTKFHMPNWWYMRPDDTEQFYNPKEP

FISGLSHLVTALILYGYLIPISLYVSIEVVKVLQASFINQDLQMYDDDTG

IPAQARTSNLNEELGQVDTILSDKTGTLTCNQMDFLKCSIAGTAYGVRAS

EVERAAAQQMANDLEQDGRQSSVSSKRSKQDIELETVVTSKDGKDNKPPI

KFFSFEDDRMTEGNWRKEPNVDMIILFCRILAVCHTAIPELNEETGTYTY

EAESPDEGAFLVAAREFGFEFFKRTQTSVFVRERYIGSGQTTEREYKILN

LLEFTSKRKRMTVIVRDEEGQIILMCKGADSIIFDRLAKKGKTYLEDTTK

HLNIYGEAGLRTLALAYRKLEESEYSAWNNEFQKAKTSIGADRDTNLEKV

AEMMEKELTLVGATAVEDKLQKGVPQCIDKLAQAGLKLWVLTGDKMETAI

NIGFACSLLREGMKQICITTLDSESKEAIKENILTQVNNGSQMIKLEKDP

YAAFALIIDGKTLAYALEEDMKLLFLGLAVDCASVICCRVSPKQKALVTR

LVKEGTGKVTLGIGDGANDVGMIQEADIGVGISGVEGMQAVMASDFSVSQ

FRFLERLLVVHGHWCYKRIAQMVCYFFYKNIAFGLTLFYFEAFTGFSGQS

VYDDWYMILFNVVLTSLPVISLGVFEQDVSSDVCLEFPTLYQQGPRNLFF

DWYRILGWMGNGLYSSLVIFSLNIVIFYDQAYRVEGQTTDMSILGTIMFT

CIICALNFQVSLIMTHYTWMQHVLIWFSIVAWFIFLFIYGMLPPKISHSA

YQILTEALASAPVYWITTLLVTIACTLPYLAHMSLQRCFNPMDHHVIQEI

KYLKKDVSDQVMWRREKTKAREKTKIGFTARVDALIRSLRGKLNKKTHSI

ESNKSYPPS

>GhALA11

MARVRKRNIHLSKLCSFSCCRRPSQPSSTDEYDRIGQKGYSRVVYCNEPD

CEEQIRLKYRRNYVSTTKYTAANFIPKSLFEQFRRVANIYFLVVACVSFS

PLAPYSAPSVLVPLIVVIGATMAKEGIEDYRRKQQDVEANNRTVEVYDRN

SSFNETKWKNLRVGDIVKVHKDEYFPADLLLLSSDYEDGVCYVETMNLDG

ETNLKSKHPLEVTSFIRDVETVKGFRAVIKCEDPNEHLYKFVGTLYYECQ

QYALSPQQILLRGTKLKGPDYINGVVIFTGHDTKVMQNAMDPPSKRTRIE

RRMDKIIYVLFSALILVSFIGSLLFGIETKKDGGDYGRWYLRPDITTVFF

DPRRPSVAAFLHFLTGLMLYGYLIPISLYVSIEICKVLQSIFINQDQAMY

DEETKRSAHARTSNLNEELGQVFTILSDKTGTLTCNKMEFVKCSIAGTAY

GFGMTEVEIALARKRGETLDEQRDIDTVESREPVKGFNFRDGRIMNRKWV

HEPYGDYIEKFFRVLALCHTAVPEVLDPGKIFYEAESPDEAAFVIAAREV

GFQFVKRNQTSIQLRELDRSSGEIVDRVYELLHVLEFSSTRRRMSVIVRN

PERQLLLLAKGADSVIFERLSEQGRMFEAETKEHIERYSEAGLRTLAVAY

RELDDDEYKRWEQEYVKAKTSVSANRDDLLDVMAELIERDLILLGATAIE

DKLQKGVPDCIDKLAQAGINIWVLTGDKKGTAINIGYACSLLRHGMKEIL

VILENPGIAAVERDTEDFAKASEIVEKQIDEGISQVTGGSSTQFGLIIDG

QSLIFALDEKLIMRFMELAMKCATVICCRSSPKQKAIVTRWVKSVTGRTT

LAIGDGANDVGMIQEADIGVGITGVEGMQAAMSSDFSIAQFRFLERLLLV

HGHWCYRRITMMICYFFYKNITFGFTLFWFEAYSSFSAQPAYNDWYMSSY

NVLFTSLPVIALGVFDQDFSARHCLEYPSLYKEGIDDVLFRWTHILGWMF

NGVLSSVIIFFLTANSITGQAFRKDGQVADYSVLGLTMYTCVVLAVNCQM

ALCINYFTWIQHLFIWGSITLWFKFLLVYGSIPPTLSTTAYKVFIEACAP

SIQYWLTIILVVIATLLPWFSYRAFQTRFRSMVRDCIQT

>GhALA12

MVGGRRKKQHFSRIHAFTCGKAYFRGDHSLIGGPGFSRVVYCNDPECFEA

SLLNYGGNYVRSTKYTLATFFPKSLFEQFRRVANFYFLICAILSFTPLSP

YSSISNVLPLVVVIGATMGKEAVEDWKRKKQEVAYSSYLAGETEKGAGRA

KRVADDIEMNNRKVKVHQGDGRFEHTKWMDLKVGDIVKVEKDEFFPADLI

LLSSSYEEAICYVETMNLDGETNLKLKQALEATSSLHEDSSFQNFKAVIR

CEDPNANLYSFVGSLEFRKEQYPLSPQQLLLRDSKLRNTDYIFGAVIFTG

HDTKVIQNSTEPPSKRSKIERRMDKIVYVLFALLVLLSVIGSIFFGIATR

EDLENGKMTRWYLRPDETTIYYDPERATVAAILQFLTALMLYSYLIPISL

YVSIEVVKVLQSIFINQDLHMYYEEADKPARARTSNLNEELGQVDTILSD

KTGTLTCNSMEFIKCSVAGTSYGRGITEVEKALAWRKVSPLAQDVTEEEG

QVEEFKKEKPSVKGFNFLDERIMNGNWIKEPRADVIQKFLLLLAVCHTAI

PEVDEEAGRTSYEAESPDEAAFVVAARELGFEFYERTQTRISFYEFDPLS

GKKVERSYNLLNILEFSSSRKRMSVIVRNEEGKLLLLCKGADRTSFYLQD

LKG

>GhALA13

MAGGRRKKQHFSRIHAFTCGKASFRGDHSLIGGPGFSRVVYCNDPECFEA

SLLNYGGNYVRSTKYTLATFFPKSLFEQFRRVANFYFLICAILSFTPLSP

YSSISNVLPLVVVIGATMGKEAVEDWKRKKQEVAYSGYLAGETEKGAGRA

KRVADVLEICQFRCSGSIVHLVEDIEMNNRKVKVHQGDGGFEHTKWMDLK

VGDIVKVEKDEFFPADLILLSSSYEEAICYVETMNLDGETNLKLKQALEA

TSSLHEDSSFQNFKAVIRCEDPNANLYSFVGSLEFRKEQYPLSPQQLLLR

DSKLRNTDYIFGAVIFTGHDTKVIQNSTEPPSKRSKIERRMDKIVYVLFA

LLVLLSVIGSIFFGIATREDLENGKKTRWYLRPDETTVYYDPERATVAAI

LQFLTALMLYSYLIPISLYVSIEVVKVLQSIFIDQDLHMYYEEADKPARA

RTSNLNEELGQVDTILSDKTGTLTCNSMEFIKCSVAGTSYGRGVTEVERA

LAWRKGSPLAQDVTEEEGQVEEFKKEKPSVKGFNFVDERMMNGNWIKEPR

ADVIQKFLRLLAVCHTAIPEVDEEAGRTSYEAESPDEAAFVVAARELGFE

FYERTQTSISFYEFDPLSGEKVERSYNLLNILEFSSSRKRMSVIVRNEEG

KLLLLCKGADSVMFERLAKNGQEFAEQTKEHIAEYADAGLRTLVIAYREI

DEQEYVEFNEQFTEAKNLVSADREEMIEEVAGKIERDLILLGATAVEDKL

QNGVPECIDKLAQAGIKIWVLTGDKMETAINIGFACSLLRQGMKQIVINP

ETPENKALEKSDDKSAAAAAFKASVLQQIAEGKRLLSSSTKNSKAVALIV

DGKSLTYALEDDVKDNFLELAIGCASVICCRSSPKQKALVTRLVKSKTGS

TTLAIGDGANDVGMLQEADIGVGISGVEGMQAVMSSDIAIAQFRFLERLL

LVHGHWCYRRISSMICYFFYKNIAFGFTIFFYEIYASFSGQAVYNDWFLS

LYNVFFTSLPVIALGVFDQDVSSRLCLKFPPLYQEGIQNVLFSWLRILAW

LFNGVLSATIIFLFCIRAMQHQAFRKGGEVVGLEILGATMYTCVVWVVNC

QMALSISYFTYIQHLFIWGGVVFWYIFLIAYGAIDPDISTSAYQVFIEAC

APAGSYWLLTLLVLIASLLPYFTYSAIQMRFFPLYHQMIQWIRSDGQTND

PEYCHMVRQRSLSHTTVGYTARFEAKSKSSKERARDH

>GhALA14

MTGGRRRKQHFSRIHAFSCGKASFRGDHSLIGGPGFSRVVYCNDPECFEA

SLRNYAGNYVRSTKYTLATFFPKSLFEQFRRVANFYFLICAILSFTPLSP

YSAVSNVLPLVVVIGATMGKEAVEDWRRKKQDTEVNNRKVKMHQSDGIFE

PTKWMDLKVGDIVKVEKDEFFPADLILLSSSYEEAICYVETMNLDGETNL

KLKGASDVTSSLHDDASFQDFKATIRCEDPNANLYSFVGSLELGDEQYPL

SPQQLLLRDSKLRNTDYIFGVVIFTGRDTKVIQNSTEPPSKRSKIEKRMD

NIVYFLFAVLVGLSIIGSIFFGIETREDLENGKMRRWYLRPDDTTIYYNP

KRAAVAAILQFLTALMLYSYLIPISLYVSIEIVKVLQSIFINQDLHMYHE

ETDKPAHARTSNLNEELGQVDTILSDKTGTLTCNSMEFIKCSIAGTSYGH

GITEVERALVWRKGSPLAREVPEINGQVEEFKKEKPLVKGFNFVDERIMN

SNWLNEPHADVIQKFLRLLAICHTAIPEVDEETGRISYEAESPDEAAFVV

AARELGFEFYERTQTSISLYEFDLSGKKVKRSYKLLNILEFSSSRKRMSV

ILQNEEGKLLLLCKGADSVMFERLAKNGIEFAEQTKEHIEEYADAGLRTL

VLAYREINEEEYVEFNEKFMEAKNIVSADREEMIEEVAESIERDLILLGA

TAVEDKLQNGVPECIDKLAQAGIKIWVLTGDKMETAINIGFACSLLRQGM

KQIIINSDTPENKALEKSGDKTAAAAAYKASVLQQIAEGRQLLTSSNENS

EALALIVDGKSLTYALEDDVKDAFLELTIGCASVICCRSSPKQKALVTRL

VKTKTGSTTLAIGDGANDVGMLQEADIGVGISGVEGMQAVMSSDIAIAQF

RFLERLLLVHGHWCYRRISSMICYFFYKNIVFGFTLFFYEIYASFTGQAV

YNDWFLSFYNVFFTSLPVITLGVFDQDVSSRLCLKFPLLYQEGIQNVLFS

WLRIVAWAFNGVLSATVIFFFCIRATQHQAFRKGGEVVGLEILGTTMYTC

VVWVVNCQMTLSISYFTYIQHLFIWGSIILWYIFLMAYGAMATSISTTAY

KVFIESCAPAGMYWLLTLLVLISSLLPYFIYSAIQVRFFPSYHQMIQWIR

SDGQSDDPEYCHMVRQRSLRPTTVGYTARLEAKSRSSRKGGEDHQ

>GhALA15

MTGGRRRKQHFSRIHAFSCGKASFKGDHSLIGGPGFSRVVYCNDPECFEA

SLRNYAGNYVRSTKYTLATFFPKSLFEQFRRVANFYFLICAILSFTPLSP

YSAVSNVLPLVVVIGATMGKEAVEDWRRKKQDTEVNNRKVKMHQSDGIFE

PTKWMDLKVGDIVKVEKDEFFPADLILLSSSYEEAICYVETMNLDGETNL

KLKQASDVTSSLHDDASFQDFKATIRCEDPNANLYSFVGSLELGDEQYPL

SPQQLLLRDSKLRNTDYIFGVVIFTGRDTKVIQNSTEPPSKRSKIEKRMD

NIVYFLFAVLVGLSIIGSIFFGIETREDLENGKMRRWYLRPDDTTIYYNP

KRAAVAAILQFLTALMLYSYLIPISLYVSIEIVKVLQSIFINQDLHMYHE

ETDKPAHARTSNLNEELGQVDTILSDKTGTLTCNSMEFIKCSIAGTSYGH

GITEVERALAWRKGSPLAREVPEINDQVEEFKKEKPSVKGFNFVDERIMN

GNWLKEPHADVIQKFLRLLAICHTAIPEVDEETGRISYEAESPDEAAFVV

AARELGFEFYETTQTSISLYEFDLSGKKVERSYKLLNILEFSSSRKRMSV

ILQNEEGKLLLLCKGADSVMFERLAKNGQEFAEQAKEHIEEYADAGLRTL

VLAYREINEEEYVEFNEKFTEAKNIVSADREEMIEEVAESIERDLILLGA

TAVEDKLQNGVPECIDKLAQAGIKIWVLTGDKMETAINIGFACSLLRQGM

KQIIINSDTPEDKALEKSGDKTAAAAAYKASVLQQIAEGRLLLTSSNENS

EALALIVDGKSLTYALEDDVKDAFLELAIGCASVICCRSSPKQKALVTRL

VKTKTGSTTLAIGDGANDVGMLQEADIGVGISGVEGMQAVMSSDIAIAQF

RFLERLLLVHGHWCDRRISSMICYFFYKNIVFGFTLFFYEIYASFSGQAV

YNDWFLSFYNVFFTSLPVIALGVFDQDVSSRLCLKFPLLYQEGIQNVLFS

WLRIIAWAFNGVLSATVIFFFCIRAVQHQAFRKGGEVVGLEILGTAMYTC

VVWVVNCQMALSVSYFTYIQHLFIWGSIILWYIFLMAYGAMDPSISTTAY

KVFIESCAPAGMYWLLTLLVLISSLLPYFIYSAIQVRFFPSYHQMIQWIR

SDGQSDDPEYCHMVRQRSLRPTTVGYTARLEAKSRSSRKGGEDHQ

>GhALA16

MSGGRRRKVLMSRIYGVACGKASFKEDHSQIGGPGFSRIVYCNEPNSLEA

GTRNYSDNYVSTTKYTVATFLPKSLFEQFRRVANFFFLVTGILSFTAIAP

YSALSAIVPLIIVIGATMIKEGVEDWRRQQQDIEVNNRKVKVHQGDGNFH

HTEWKNLRVGDIVKVEKDEFFPTDLILLASSYEDAVCYVETMNLDGETNL

KLKQALEVTSSLHNDYNFRDFKAIVKCEDPNANLYSFVGTMEFEEQQHPL

SPQQLLLRDSKLRNTDYIYGAVVFTGHDTKVMQNATDPPSKRSKIEKTMD

RVIYLMFFIVFIMGFVGSIFFGIATENDYEGGRIKRRWYLRPDNAEIFFD

PERAPVAAIYHFLTALLLYSYFIPISLYVSIEIVKVLQSIFINQDSHMYY

EEADKPAHARTSNLNEELGQVDTILSDKTGTLTCNSMEFIKCSIAGTAYG

RGVTEVERAIYRKKGSPVVHEPNGLNHIEDSADANPAIKGFNFKDERIMN

GNWVNEPRADVIQKFFRLLAICHTAIPEVDEENGNISYEAESPDEAAFVI

AARVLGFEFHNRTQTSISLHELDPVPGKRVNRLYKLLNVLEFDSTRKRMS

VIVRDEEGKLLLLCKGADSVMFERLAKGGRDFEEDTREHMNEYADAGLRT

LVLAYRELSENEYEVFNEKMTEAKNSVSADRETLIDGVAEMIERDLILLG

ATAVEDKLQNGVPDCIDKLAQAGIKLWVLTGDKMETAINIGYACSLLRQG

MKQIIINLDTPEIQSLEKTGDKDAVIKASRKSVMEQIVSGKSQVSALSAI

SEAFALIIDGKSLAYALEDDMKNIFLELAIGCASVICCRSSPKQKALVTR

LVKLGTGKTTLAIGDGANDVGMLQEADIGIGISGVEGMQAVMSSDVAIAQ

FRYLERLLLVHGHWCYRRISSMICYFFYKNIAFGFTIFLYEAYTSFSAQP

AYNDWYLTLFNVFFSSLPVIAMGVFDQDVSARFCLKFPLLYQEGVQNVLF

SWRRIVSWMFNGFYSAIIIFFFCSRALEQQAFNDEGKTASKDILGGTMYT

CIVWVVNLQMALSISYFTLIQHIVIWGTIAFWYVFQLAYGALPASFSTDA

YRVFVEALAPAPSYWFITLFVVIATLTPYFLYSAIQMRFFPMYHEMIQWI

RHEGLSDDPLYCEMVRQRSIRPTTVGFTARRAASRRQ

>GhALA17

MSGGRRRKVLMSRIYGIACGKASFKEDHSQIGGPGFSRVVYCNEPNSLEA

GTRNYSDNYVSTTKYTIATFLPKSLFEQFRRVANFFFLVTGILSFTAIAP

YSALSAIVPLIIVIGASMIKEGVEDWRRQQQDIEVNNRKVKVHQGDGNFH

HTEWKNLRVGDIVKVEKDEFFPTDLILLASSYEDAVCYVETMNLDGETNL

KLKQALEVTSSLHDDYNFRDFKAIVKCEDPNANLYSFVGTMEFEEQQHPL

SPQQLLLRDSKLRNTDYIYGAVVFTGHDTKVMQNATDPPSKRSKIEKTMD

RVIYLMFFIVFIMGFVGSIFFGIATENDYEGGRIKRRWYLRPDNAEIFFD

PERAPVAAIYHFLTALLLYSYFIPISLYVSIEIVKVLQSIFINQDSHMYY

EEADKPAHARTSNLNEELGQVDTILSDKTGTLTCNSMEFIKCSIAGTAYG

RGVTEVERAIYRKKGSPVVHEPNGLNHIEDSAGVNPAIKGFNFKDERILN

GNWVNEPRADVIQKFFRLLAICHTAIPEVDEENGNISYEAESPDEAAFVI

AARVLGFEFHNRTQTSISLHELDPVSGKRVNRLFKLLNVLEFDSSRKRMS

VIVRDEEGKLLLLCKGADSVMFERLAKGGRDFEEDTREHMNEYADAGLRT

LVLAYRELSENEYEVFNEKMTEAKNSVSADRETLIDEVAEMIERDLILLG

ATAVEDKLQNGVPDCIDKLAQAGIKLWVLTGDKMETAINIGYACSLLRQG

MKQIIINIDTPEIQSLEKTGDKDAVIKASRKSVMEQIVSGKAQVSALSAI

SEAFALIIDGKSLAYALEDDMKNSFLELAIGCASVICCRSSPKQKALVTR

LVKLGTGKTTLAIGDGANDVGMLQEADIGIGISGVEGMQAVMSSDVAIAQ

FRYLERLLLVHGHWCYRRISSMICYFFYKNIAFGFTIFLYEAYTSFSAQP

AYNDWYLTLFNVFFSSLPVIAMGVFDQDVSARFCLKFPLLYQEGVQNVLF

SWRRIVSWMFNGFYSAIIIFFFCSRALEQQAFNDEGKTASKDILGGTMYT

CIVWVVNLQMALSISYFTLIQHIVIWGTIAFWYVFQLAYGALPASFSTDA

YRVFVEALAPAPSYWFITLFVVIATLTPYFLYSAIQMRFFPMYHEMIQWI

RHEGLSDDPLYCEMVRQRSIRPTTVGFTARRAASRRQ

>GhP5-1

MSRFHVGGKVVDKVDLLRKKHAAWRLDVWPFAMLYLLWLTMVVPSIDFVD

AAIVLGGLAVTHILVLLFTTWSVDFKCFVQYSKVNNIRLADVCKVTPAKF

CGSKEIASSSSAKDVEEIYFDFRKQCFIYSKEEDTFCKLPYPTKETFGYY

LKCSGHGSDAKVLAATEKWGRNVFEYPQPTFQKLMKEHCMEPFFVFQVFC

VGLWCLDEYWYYSLFTLFMLFMFESTMAKSRLKTLSELRRVRVDSQTLMV

HRCGKWVKLSGTDLLPGDVVSIGRSSGQNEEDKSVPADMLILAGSAIVNE

AILTGESTPQWKVSIAGRGIEEKLSAKRDKNHMLFGGTKILQHTADKSFP

VRTPDGGCLAVVLRTGFETSQGKLMRTILFSTERVTANSWESGLFILFLV

VFAIIAAGYVLKKGLEDPTRSKYKLFLSCSLIITSVIPPELPMELSIAVN

TSLIALARRGIFCTEPFRIPFAGKVDICCFDKTGTLTSDDMEFSGVVGLN

DSSELESDMTKVPSRTVEILASCHALVFVDNKLVGDPLEKAALKGIDWSY

KSDEKAIPKKGSGNPVQIAQRHHFASHLKRMAVVVRVQEDFFSFVKGAPE

TIQDRLIDLPPTYVETYKKYTRQGSRVLALAYKSLPDMTVSEARSMERDT

VECGLTFAGFAVFNCPIRADSLTVLSELKNSSHDLVMITGDQALTACHVA

GQVNIVSKLALILVAVKNSKGYEWVSPDETERIPYSENEVEALSETHDLC

IGGDCFEMLQQTSAVLRVIPFVKVFARVAPEQKELIMTTFKTVGRLTLMC

GDGTNDVGALKQVHCAAIMFKAFQAHVGVALLNAVPPTKSESSSGTSKDE

NTKSLKSKKSKPTVEATGNSEASSKGKVVPRSESSNNATSNRHLNAAEKH

RQKLKKMMDELNEEGDGRSAPIVKLGDASMASPFTAKHASVAPTTDIIRQ

GRSTLVTTLQMFKILGLNCLATAYVLSVMYLDGVKLGDVQATISGVFTAA

FFLFISHARPLPTLSAARPHPNIFCSYVFLSLMGQFAMHLLFLISSVKEA

EKHMPEECIEPESEFHPNLVNTVSYMVSMMLQVATFAVNYMGHPFNQSIP

ENKPFLYALGAAAGFFVVITSDIFRDLNDWLSLVPLPVGLRDKLLIWALL

MFLCCYAWERLLRWAFPGKIPAWRKRQRVAAASSEKKLV

>GhP5-2

MSRFHVDGKVVDKVDLLRKKHAAWRLDVWPFAMLYLLWLTMVVPSIDFVD

AAIVLGGLAVTHILVLLFTTWSVDFKCFVQYSKVNNIRLADVCKVTPAKF

CGSKEIASSSSAKDVEEIYFDFRKQCFIYSEEEDTFCKLPYPTKETFGYY

LKCSGHGSDAKVLAATEKWGRNVFEYPQPTFQKLMKEHCMEPFFVFQVFC

VGLWCLDEYWYYSLFTLFMLFMFESTMAKSRLKTLSELRRVRVDSQTLMV

HRCGKWVKLSGTDLLPGDVVSIGRSSGQNEEDKSVPADMLILAGSAIVNE

AILTGESTPQWKVSIAGRGIEEKLSAKRDKNHMLFGGTKILQHTADKSFP

LRTPDGGCLAVVLRTGFETSQGKLMRTILFSTERVTANSWESGLFILFLV

VFAIIAAGYVLKKGLEDPTRSKYKLFLSCSLIITSVIPPELPMELSIAVN

TSLIALARRGIFCTEPFRIPFAGKVDICCFDKTGTLTSDDMEFSGVVGLN

DSSELESDMTKVPSRTVEILASCHALVFVDNKLVGDPLEKAALKGIDWSY

KSDEKAIPKKGSGNPVQIVQRHHFASHLKRMAVVVRVQEDFFAFVKGAPE

TIQDRLIDLPPTYVETYKKYTRQGSRVLALAYKSLPDMTVSEARSMERDT

VECGLTFAGFAVFNCPIRADSSTVLSELKNSSHDLVMITGDQALTACHVA

GQVNIVSKPALILVAVKNSKGYEWVSPDETERIPYSENEVEALSETHDLC

IGGDCFEMLQQTSAVLRVIPFVKVFARVAPEQKELIMTTFKTVGRLTLMC

GDGTNDVGALKQVYCAAIMFKAFQAHVGVALLNAVPPTKSESSSGTSKDE

NTKALKSKKSKPTVEATGNSEASSKGKVVPRSESSNNATSNRHLNAAEKH

RQKLKKMMDELNEEGDGRSAPIVKLGDASMASPFTAKHASVAPTTDIIRQ

GRSTLVTTLQMFKILGLNCLATAYVLSVMYLDGVKLGDVQATISGVFTAA

FFLFISHARPLPTLSAARPHPNIFCSYVFLSLMGQFAMHLLFLISSVKEA

EKHMPEECIEPESEFHPNLVNTVSYMVSMMLQVATFAVNYMGHPFNQSIP

ENKPFLYALGAAAGFFVVITSDLFRDLNDWLSLIPLPVGLRDKLLLWALL

MFLCCYAWERLLRWAFPGKIPAWRKRQRVAAASSEKKLV

>GrAHA11

MDTEKKAVSLEAINKEIVDLENIPIEEVLEKLKCTKEGLTTDEVQQRLELFGYNKLEEKKENKILKFLGFMWNPLSWVMEAAAVMAIALAHGGKKETDYHDFVGILALLLINSTISFIEENNAGNAAAALMARLAPKAKVLRDGKWNEEDASVLVPGDIISIKLGDIIPADARLLQGDPLKIDQSALTGESLPVTKHPGDGVYSGSTCKQGEIEAVVIATGVHTFFGKAAHLVESTTHVGHFQQVLTSIGNFCICSIAIGMLAELIVIYGAQRRSYRTGIDNLLVILIGGIPIAMPTVLSVTMAIGSHRLSQQGAITKRMTAIEEMAGMDVLCSDKTGTLTLNKLTVDKNLIEVFVNNVDKDTVILMAARASRLENQDAIDTAIVAMLADPKEARAGITEVHFLPFNPTDKRTALTYVDEAGKMHRVSKGAPEQILNLAYNKSEIGKKVHSIIDKYAERGLRSLAVARQEVPAGTKDSPGGPWEFVGLLPLFDPPRHDSAETIRRALDLGVSVKMITGDQLAIGKETGRRLGMGTNMYPSSSLLGDHKNEEIGAFSIDELIENADGFAGVFPEHKFEIVKRLQAKKHIVGMTGDGVNDAPALKKADIGIAVADSTDAARSASDIVLTEPGLSVIISAVLTSRAIFQRMKNYTIYAVSITIRIVLGFMLLTLIWRLNFPPFMVLIIAILNDGTIMTISKDRVKPSPLPDSWKLKEIFATGIVLGSYLALMTLIFFWAAYETDFFPDKFGVRSLSKKSFDLTQESERRKVNALLSSAVYLQVSTISQALIFVTRSRGWSFTERPGLLLVTAFIIAQLVATVISAQATWSFAGIRAVGWGWCGVIWIYNILTYFLLDPIKFAVRYALSGKAWDLVLNQRTAFSTQKDFGKEAREAAWAAEQRTLHGLQSISDAKITEKHNFRDISVMAEEARRRAEIARLREIHTLKGKVESFAKLRGLDIDVNPHYTV

>GrECA3

MDKRPFPAWSWSVEQCLKEYNAKLDKGLSSYQVEKQREKYGWNELAKEKGKPLLRLVLEQFDDMLVKILLVAAFISFILAYMHGSDSEESGFEAYVEPFVIVLILVLNAIVGVWQETNAEKALEALKEMQCESGKVLRDGYIVPDLPARELVPGDIVELQVGDKVPADMRIAALKTSTLRLEQSALTGEAMPVLKGTSPIFPEECELQAKENMVFAGTTVVNGSCVCIVVCTGMNTEIGKIQKQIHEASLEESDTPLKKKLDEFGSRLTTAIGLVCLIVWLINYKNFLSWDMVDGWPANLRFSFEKCTYYFKIAVALAVAAIPEGLPAVITTSLALGTRKMAQKNAIVRKLPSVETLGCTTVICSDKTGTLTTNQMSVAEFFTLGGKTTTSRMFHVEGTTYDPKDGGIVDWTCYNMDANLQVMAEICAVCNDAGIFCDGRLFRATGLPTEAALKVLVEKMGVPDAKMRNKIHDSQLAANYLIDRSTIKLGCCEWWTKRSKRLATLELDTVRKSMSVIVREPTGHNRLLVKGAVESLVERSTHVQLADGSLVPMDESCSQLLLSRNSEMSSKGLRCLGLAYKDDLGEFSDYYSENHPAHKKLLDPASYSSIESDLVFVGVVGLRDPPRDEVDKAIEDCKAAGIRVMVITGDNKSTAEAICHEIKLFSDGEDVRGKSFTGKEFMALSPSQQIETLSKPGGKVFSRAEPRHKQEIVRMLKEMGEIVAMTGDGVNDAPALKLADIGIAMGITGTEVAKEASDMVLADDNFSTIVSAVAEGRSIYNNMKAFIRYMISSNVGEVISIFLTAALGLPECMIPVQLLWVNLVTDGPPATALGFNPPDVGIMWKPPRKSDDALIDSWVLFRYLTIGSYVGVATVGIFILWYTQASFMGINLVSDGHTLIELSQLRNWGECSTWSNFSVAPYTVGDGHLITFSNPCDYFTIGKVKAMTLSLSVLVAIEMFNSLNALSEDSSLLTLPPWRNTWLLVAMSVSFGLHCLILYVPFLANMFGVVPLSLNEWFLVILVSIPVVLIDETLKFFGRSRRHRVKEKTA

>GrAHA13

MGDKNEVLEAVLKETVDLENIPIEEVFENLRCSREGLTTEAAEERLTIFGHNKLEEKKESKFLKFLGFMWNPLSWVMEAAAIMAIALANGGGKPPDWQDFVGIITLLVINSTISFIEENNAGNAAAALMARLAPKAKVLRDGRWNEQDAAILVPGDIISIKLGDIIPADARLLEGDPLKIDQSALTGESLPVTKGPGDGIYSGSTCKQGEIEAVVIATGVHTFFGKAAHLVDTTNQVGHFQKVLTAIGNFCICSIAVGMVIEIIVMYPIQDRDYRPGIDNLLVLLIGGIPIAMPTVLSVTMAIGSHRLSQQGAITKRMTAIEEMAGMDVLCSDKTGTLTLNKLTVDKNLIEVFAKGVDADTVVLMAARASRTENQDAIDSAIVGMLADPKEARAGIREVHFLPFNPTDKRTALTYIDSDGKMHRVSKGAPEQILHLAHNKADIERRVHAVIDKFAERGLRSLAVAYQEVPDGRKESPGGPWQFIGLMPLFDPPRHDSAETIRRALNLGVNVKMITGDQLAIGKETGRRLGMGTNMYPSSALLGQDKDESIAALPVDELIEKADGFAGVFPEHKYEIVKRLQARKHICGMTGDGVNDAPALKKADIGIAVADATDAARSASDIVLTEPGLSVIISAVLTSRAIFQRMKNYTIYAVSITIRIVLGFMLLALIWKFDFPPFMVLIIAILNDGTIMTISKDRVKPSPLPDSWKLAEIFTTGIVLGSYLAVMTVIFFWAAYKTNFFPRVFGVATLEKTAHDDIKKLASAVYLQVSIISQALIFVTRSRSWSFVERPGLLLLAAFVIAQLIATLIAVYANWSFAAIEGIGWGWAGVIWLYNIIFYIPLDFIKFFIRYALSGRAWDLVIEQRVCIPLSYRVHCYCCCLIRHIGLSDEFLFDAQIAFTRQKDFGKEQRELQWAHAQRTLHGLQAPDTKMFTERTHFTELNQMAEEAKRRAEIARLRELHTLKGHVESVVRLKNLDIDTIQQAYTV

>GrAHA6

MAETSVGLEDIKNETIDLERIPVHEVFQKLKCTRDGLSSEEGQKRLHIFGPNKLEEKQESKVLKFLGFMWNPLSWVMEIAAIMAIALANGGGKPPDWQDFIGIVALLFINSTISFIEENNAGNAAAALMAGLAPKTKVLRDGKWSEQEAATLVPGDIISIKLGDIVPADARLLEGDPLKIDQSALTGESLPVNKNAGDEVFSGSTVKQGELEAVVIATGVHTFFGKAAHLVDSTNNVGHFQQVLTAIGNFCICSIGVGMLIEIVVMYPIQQRRYRDGIDNLLVLLIGGIPIAMPTVLSVTMAIGSHRLSQQGAITKRMTAIEEMAGMDVLCSDKTGTLTLNKLTVDKSLVEVFTNDVDKDMVILLAARASRVENQDAIDACIVGMLGDPKEARAGITEVHFFPFNPVDKRTAMTYIEADGSWHRVSKGAPEQIIDLCNLRDDVKRRTHDIIANFADRGLRSLAVARQTVKEKNKDAQGEAWEFVGILPLFDPPRHDSAETIRRALNLGVNVKMITGDQLAIGKETGRRLGMGTNMYPSSALLGQNKGDTIDTVGVDELIEKADGFAGVFPEHKYEIVKRLQQRNHICGMTGDGVNDAPALKKADIGIAVDDATDAARGASDIVLTEPGLSVIVSAVLTSRAIFQRMKNYTIYAVSITIRIVLGFMLLALIWKFDFSPFMVLIIAILNDGTIMTISKDRVKPSPMPDSWKLKEIFAMGIVLGTYLACMTVVFFWAANDSNFFQDKFGVRSIRHNQDELTAAIYLQVSIVSQALIFVTRSRSWSFIERPGLLLVVAFILAQLIATLLAVYANWGFARIKGIGWGWAGVIWLYSLVFYVPLDVLKFLIRYSLSGKAWDNLLQNKTAFTTKKDYGKGEREAQWAMAQRTLHGLTPPEMTQLYNEETNYRELSEIAEQARKRAEVARLRELHTLKGHVESVVKLKGLDIDTIQQHYTV

>GrAHA14

MSEELDKPLLDPENFNREGIDLERLPLEEVFEQLRTSRAGLTSEDAEVRVHIFGQNKLEEKPENKFLKFLSFMWNPLSWVMEAAAVMAIVLANGGGEGPDWQDFVGIICLLIINSTISFIEENNAGNAAAALMARLAPKTKVLRDGQWQERDAAILVPGDIISIKLGDIIPADARLLEGDPLKIDQATLTGESLPVTKRTGDEVFSGSTCKHGEIEAVVIATGVHSFFGKAAHLVDSTEVVGHFQQVLTSIGNFCICSIAVGMVLEIIVMFPIQLRSYRDGINNLLVLLIGGIPIAMPTVLSVTLAIGSHRLSQQGAITKRMTAIEEMAGMDVLCSDKTGTLTLNRLTVDRNLVEVFSKNMDKDLVVLLAARASRLENQDAIDAAIINMLADPKEARANIKEVHFLPFNPVDKRTAITYIDSDGNWYRASKGAPEQILNLCLEKDEIAGRVHAIIDKFAERGLRSLGVAFQEVPERTKESPGGPWTFCGLLPLFDPPRHDSAETIRRALNLGVDVKMITGDQLAIAKETGRRLGMGTNMYPSSSLLGREKDESEALPADELIEKADGFAGVFPEHKYEIVKILQEKKHVVGMTGDGVNDAPALKKADIGIAVADATDAARSAADIVLTEPGLSVIISAVLTSRAIFQRMKNYTIYAVSITIRIVLGFVLLALIWEYDFPPFMVLIIAILNDGTIMTISKDRVKPSPTPDSWKLNEIFATGVVIGTYLALVTVLFYWIVIDTDFFETHFNVRSISDNTEQISSAVYLQVSIISQALIFVTRSRSWSFVERPGVLLMCAFVVAQLVATLIAVYAHMSFADISGIGWGWAGVIWLYSLVFYVPLDIIKFTVRYALSGEAWNLLFDRKTAFTSKKDYGKDDRAAQWILSQRSLQGLMAADLDFNGRRSRTSLIADQARRRAEIARLGELHTLRGHVESVMRLKNLDLNAIKSAHTV

>GrAHA5

MWNPLSWVMEAAAIMAIALANGDGKPPDWQDFVGIVCLLVINSTISFIEENNAGNAAAALMAGLAPKTKVLRDGKWTEQEAAILVPGDIISIKLGDIIPADARLLEGDPLKVDQSALTGESLPVTKNPGDEVFSGSTCKQGEIEAIVIATGVHTFFGKAAHLVDSTNQVGHFQKVLTAIGNFCICSIAIGMLVEIVVMYPIQHRKYRDGIDNLLVLLIGGIPIAMPTVLSVTMAIGSHRLSQQGAITKRMTAIEEMAGMDVLCSDKTGTLTLNKLSVDKNLIEVFVKDADKEHVVLLAARASRTENQDAIDAAIVGMLADPKEARAGIREVHFLPFNPVDKRTALTYIDSNGNWHRASKGAPEQILALCNAKEDLKKRVHSIIDKFAERGLRSLAVSRQQVPEKTKESAGTPWQFVGLLPLFDPPRHDSAETIRQALHLGVNVKMITGDQLAIAKETGRRLGMGTNMYPSASLLGQDKDASIAALPVEELIEKADGFAGVFPEHKYEIVRKLQERKHICGMTGDGVNDAPALKKADIGIAVADATDAARSASDIVLTEPGLSVIISAVLTSRAIFQRMKNYTIYAVSITIRIVFGFLFIALIWKFDFSPFMVLIIAILNDGTIMTISKDRVKPSPLPDSWKLKEIFATGIVLGGYLALMTVIFFWVMHDTDFFSDKFGVRSLRERDHEMMGALYLQVSIVSQALIFVTRSRSWSYAERPGLLLVTAFIIAQLVATLIAVYANWGFARIKGIGWGWAGVIWLYSIVFYVPLDIMKFAIRYILSGKAWLNLLENKTAFTTKKDYGKEEREAQWALAQRTLHGLQPPETSNLFNDKNSYRELSEIAEQAKRRAEVARLRELHTLKGHVESVVKLKGLDIDTIQQHYTV

>GrAHA1

MGNDRGISLEEIKNESVDLERIPIEEVFEQLKCTRGGLTSEEGANRLQVFGPNKLEEKKESKFLKFLGFMWNPLSWVMEAAALMAIALANGDGRPPDWQDFVGIIALLLINSTISFIEENNAGNAAAALMANLAPKTKVLRDGRWSEQDAAILVPGDIITIKLGDIVPADARLLEGDPLKIDQSALTGESLPVTKNPSDEVFSGSTCKQGEIEAVVIATGVHTFFGKAAHLVDSTNQVGHFQKVLTAIGNFCICSIAVGIIIEMIVMYPIQHRKYRDGIDNLLVLLIGGIPIAMPTVLSVTMAIGSHRLSQQGAITKRMTAIEEMAGMDVLCSDKTGTLTLNKLTVDRNLIEVFAKGVEKEHVILYAARASRTENQDAIDAAIVGMLADPKEARAGIREIHFLPFNPVDKRTALTYIDAAGNWHRASKGAPEQIITLCNCKEDVKKKVHAVIDKFAERGLRSLAVARQEVPEKSKDGPGAPWQLIGLLPLFDPPRHDSAETIRRALNLGVNVKMITGDQLAIAKETGRRLGMGTNMYPSSSLLGQDKDASIASLPVDELIEKADGFAGVFPEHKYEIVKRLQERKHICGMTGDGVNDAPALKKADIGIAVADATDAARSASDIVLTEPGLSVIISAVLTSRAIFQRMKNYTIYAVSITIRIVFGFMFIALIWKFDFAPFMVLIIAILNDGTIMTISKDRVKPSPQPDSWKLKEIFSTGIVLGGYLALMTVVFFWAMKDADFFPNMFNVRSLADSPEEMMAALYLQVSIVSQALIFVTRSRSWSYVERPGLLLLTAFVIAQLVATLIAVYANWGFARIKGMGWGWAGVIWLYSVVTYIPLDLIKFAIRYVLSGKAWDNLLENKTAFTTKKDYGKEEREAQWAAAQRTLHGLQPPETSNLFNEKSSYRELSEIAEQAKRRAEVARLRELNTLKGHVESVVKLKGLDIDTIQQHYTV

>GrAHA7

MADKSISLEEIKNETVDLERIPVEEVFQQLKCTRNGLTSEEGQKRLQIFGPNKLEEKKENKLLKFLGFMWNPLSWVMEFAAIMAIALANGGGKPPDWQDFIGIVSLLFINSTISFIEENNAGNAAAALMAGLAPKTKVLRDGKWNEQEAAILVPGDIISIKLGDIVPADARLLEGDALKIDQSALTGESLPVNKHSGDEVFSGSTVKQGEIEAVVIATGVHTFFGKAAHLVDSTNNIGHFQQVLTAIGNFCICSIGVGMLIEIVVMYPIQRRKYRDGIDNLLVLLIGGIPIAMPTVLSVTMAIGSHRLSQQGAITKRMTAIEEMAGMDVLCSDKTGTLTLNKLTVDKSMIEVFMDNIDKEMVLLLAARASRVENQDAIDACIVGMLGDPKEARAGVTEVHFFPFNPVDKRTAMTYIEADGSWHRASKGAPEQIIELCNLRNDAKRRAHDIITKFADRGLRSLAVAKQKVPEKTKDGQGDPWQFVGLLPLFDPPRHDSAETIRRALSLGVNVKMITGDQLAIGKETGRRLGMGTNMYPSSVLLGENKGDALDTIGVDELIEKADGFAGVFPEHKYEIVKRLQQRKHICGMTGDGVNDAPALKKADIGIAVDDATDAARSASDIVLTEPGLSVIVSAVLTSRAIFQRMKNYTIYAVSITIRIVLGFMLLALIWKFDFSPFMVLIIAILNDGTIMTISKDRVKPSPMPDSWKLKEIFGTGIVLGTYLACMTVVFFWAANDSNFFSDKFGVRSIRHNQDELTAAVYLQVSIVSQALIFVTRSRSWSFIERPGFLLVIAFILAQLVATVIAVYANWGFARIKGIGWGWAGVIWIYSVVFYFPLDVFKFLIRYAMSGKAWNNLLQNKTAFTTKKDYGKGEREAQWALAQRTLHGLTPPEITEKSNYIELSEIAEQARKRAEVARLRELHTLKGHVESVVKLKGLDIDTIQQHYTV

>GrAHA2

MGGEKGISLEEIKNESVDLERIPIEEVFEQLKCTREGLSTEEGNNRLQVFGPNKLEEKKESKVLKFLGFMWNPLSWVMEAAAIMAIALANGDGRPPDWQDFVGIIVLLVINSTISFIEENNAGNAAAALMANLAPKTKVLRDGRWSEQEAAILVPGDIITIKLGDIVPADARLLEGDPLKIDQSALTGESLPVTKNPSDEVFSGSTCKQGEIEAVVIATGVHTFFGKAAHLVDSTNQVGHFQKVLTAIGNFCICSIAVGIIVELIVMYPIQHRKYRDGIDNMLVLLIGGIPIAMPTVLSVTMAIGSHRLSQQGAITKRMTAIEEMAGMDVLCSDKTGTLTLNKLTVDRNLIEVFTKGVEKEHVILYATRASRTENQDAIDAAIVGMLADPKEARAGVREIHFLPFNPVDKRTALTYIDSDGNWLRASKGAPEQIIDLCKCKDDVRKKVHSVIDKFAERGLRSLAVARQEIPEKTKESPGSPWQFIGLLPLFDPPRHDSAETIRRALNLGVNVKMITGDQLAIAKETGRRLGMGTNMYPSSSLLGQDKDASIAALPIDELIEKADGFAGVFPEHKYEIVKRLQERKHICGMTGDGVNDAPALKKADIGIAVADATDAARSASDIVLTEPGLSVIISAVLTSRAIFQRMKNYTIYAVSITIRIVFGFMFIALIWKFDFAPFMVLIIAILNDGTIMTISKDRVKPSPQPDSWKLKEIFSTGIVLGGYLALMTVIFFWAMKDTNFFSNTFNVRSLRHSIDGEREMMAALYLQVSIVSQALIFVTRSRSWSYFERPGLLLVSAFLVAQLVATLIAVYADWGFARIKGMGWGWAGVIWLYSVVTYIPLDFIKFAIRYVLSGKAWDNLLENKTAFTTKKDYGKEEREAQWAAAQRTLHGLQPPETTSIFNERSSYRELSEIAEQAKRRAEVARLRELNTLKGHVESVVKLKGLDIDNIQQHYTV

>GrAHA3

MAGNKGISLDEIKNESVDLERIPIEEVFEQLKCTREGLSTQEGNNRLQVFGPNKLEEKKESKFLKFLGFMWNPLSWVMEAAAIMAIALANGGGRPPDWQDFVGIIVLLVINSTISFIEENNAGNAAAALMANLAPKTKVLRDGRWSEQEAAILVPGDIITIKLGDIVPADARLLEGDPLKIDQSALTGESLPVTKNPSDEVFSGSTCKQGEIEAVVIATGVHTFFGKAAHLVDSTNQVGHFQQVLTAIGNFCICSIAVGIVIELIVMYPIQRRKYRDGIDNLLVLLIGGIPIAMPTVLSVTMAIGSHRLSQQGAITKRMTAIEEMAGMDVLCSDKTGTLTLNKLTVDRNLIEVFAKGVEKEQVLLYAARASRTENQDAIDAAIVGMLADPKEARAGIREVHFLPFNPVDKRTALTYIDSDGKWHRASKGAPEQIITLCNCKADVRNKVHAVIDKFAERGLRSLAVARQEVPEKTKESPGAPWQLVGLLPLFDPPRHDSAETITRALNLGVNVKMITGDQLAIAKETGRRLGMGTNMYPSSSLLGQDKDSSVSALPVDELIEKADGFAGVFPEHKYEIVKRLQERKHICGMTGDGVNDAPALKKADIGIAVADATDAARSASDIVLTEPGLSVIISAVLTSRAIFQRMKNYTIYAVSITIRIVFGFLFIALIWKFDFAPFMVLIIAILNDGTIMTISKDRVKPSPQPDSWKLKEIFSTGIVLGGYLALMTVLFFWAMKDTDFFSVSTAFAMSPFMCYFL

>GrECA2

MEERAFPAWSWSVEHCLKEYDVRLDKGLSSYKVEKQREKYGWNELAKEKGKPLWRLVLEQFDDMLVKILLVAAFISFLLAYMHGSESEESGFEAYVEPFVIVLILFLNAIVGVWQETNAEKALEALKEMQCESGKVLRDGFLVPDLPARELVPGDIVELQVGDKVPADMRIAALKTSTLRLEQSALTGEAMPVLKGTSPIFQKECELQAKENIVFAGTTVVNGCCVCIVVCTGMNTEIGKIQRQIHEASLEESDTPLKKKLDEFGSRLTTAIGIVCLIVWLINYKNFLSYDMVDGWPANFRFSFEKCTYYFKIAVALAVAAIPEGLPAVITTSLALGTRKMAQKNAIVRKLPSVETLGCTTVICSDKTGTLTTNQMSVAEFFTLGGKTTTSRIFHVKGTTYDPKDGGIVDWTCYNMDANLQVMAEICAVCNDAGIFCDGRLFRATGLPTEAALKVLAEKMGVPDAKMRNKIRDSELVANYLIDRSTVKLGCCEWWIKRSKRVATLEFDRVRKSSSIIVREAAGQNRLLAKGAVESLLERSTHVQLADGSLAPMDEPCRQLLLSRQTEMSSKGLRCLGLAYKEDLGEFSDYYSENHPAHKKLLDPACYCSIENDLVFVGVVGLRDPPRDEVHKAIEDCKGAGIRVMVITGDNKSTAEAICREIKLFSDGEDLRGKSFTGKEFMALSPSQQIETLSKPGGKVFSRAEPRHKQEIVRMLKEMGEIVAMTGDGVNDAPALKLADIGIAMGITGTEVAKEASDMVLADDNFSTIVSAVAEGRSIYNNMKAFIRYMISSNVGEVISIFLTAALGLPECMIPVQLLWVNLVTDGPPATALGFNPPDIGIMHKPPRRSDDALINSWVLFRYLIIGSYVGIATVGIFILWYTRASFMGINLVSDGHTLVELSQLHNWGECSTWSNFTVAPYMVGGGQLITFSNPCDYFTAGKVKAMTLSLSVLVAIEMFNSLNALSEDSSLLTLPPWRNPWLLVAMSVSFGLHCLILYVPFLADIFAVAPLSLNEWFLVILVSVPVILIDEILKFVGRSQRYRVKEKTA

>GrAHA8

MVDIDDTLQEIKNENVDLERIPVEEVFLQLKCTKEGLTTEEGLKRLQIFGPNKLEEKSESKVLKFLGFMWNPLSWVMEIAAIMAIALANGGGKPPDWQDFVGITVLLIINSTISFIEENNAGNAAAALMAGLAPKTKVLRDGKWCEQEAAILVPGDIISIKLGDIIPADARLLEGDALKVDQSALTGESLPVNKNPGDGVYSGSTVKQGELEAVVIATGVHTFFGKAAHLVDSTNNVGHFQKVLTAIGNFCICSIAVGMLVEIVVMYPIQRRKYRDGIDNLLVLLIGGIPIAMPTVLSVTMAIGSHRLSQQGAITKRMTAIEEMAGMDVLCSDKTGTLTLNKLTVDKSMVEVFTKDVDKEMLLLLAARASRVENQDAIDACIVGMLGDPKEAREGITEVHFFPFNPVDKRTAMTYIESDGSWHRVSKGAPEQIISLCDLRDDVKKKAHDIIDKFADRGLRSLGVARQTVPEKTKDSPGSPWEFVGLLPLFDPPRHDSAETIRRALHLGVNVKMITGDQLAIGKETGRRLGMGTNMYPSSALLGHNKDEKVDTIDVDELIEKADGFAGVFPEHKYEIVKRLQERNHICGMTGDGVNDAPALKKADIGIAVDDATDAARSASDIVLTEPGLSVIVSAVLTSRAIFQRMKNYTIYAVSITIRIVLGFMLLALIWKFDFSPFMVLIIAILNDGTIMTISKDRVKPSPMPDSWKLKEIFATGIVLGTYLACMTVVFFWAANDSDFFSDKFGVKSIRYSQDELTAAVYLQVSIVSQALIFVTRSRSWSFIERPGLLLVVAFILAQLVATVLAVYANWGFARIKGIGWPWAGVIWLYSIVFYIPLDVLKFLIRYALSGKAWDNLLQNKTAFTTKKDYGREEREAQWATAQRTLHGLQAPGAEEILNEKSSYRELSEIAEQAKKRAEVARLRELHTLKGHVDSVVKLKGLDIETINQNYTV

>GrAHA12

MENKDETMDAVLKEAVDLENVPLEEVFQTLRCNRDGLTTEAAEQRLSIFGYNKLEEKQESKILKFLGFMWNPLSWVMEAAAIMAIALANGGGKPPDWQDFVGIITLLTINSTISFIEENNAGNAAAALMARLAPKAKVFRDGKWSEEEASILVPGDIISIKLGDIIPADARLLDGDPLKIDQSSLTGESLPVTKGPGDSIYSGSTCKQGEIEAVVIATGVHTFFGKAAHLVDSTNQQGHFQKVLTAIGNFCICSIAVGMITEIIVMYPIQDRDYRPGIDNLLVLLIGGIPIAMPTVLSVTMAIGSHRLSLQGAITKRMTAIEEMAGMDVLCSDKTGTLTLNKLSVDKNLIEIFAKGVDPDTVVLMAARAARLENQDAIDAAIVGMLADPKEARAGIQEVHFLPFNPTDKRTALTYIDNQGRMHRVSKGAPEQILNLAHNKSELERRVHAVIDKFAERGLRSLAVAYQEVPEGRKESSGGPWQFIGLMPLFDPPRHDSADTIRRALNLGVNVKMITGDQLAIAKETGRRLGMGTNMYPSSALLGQNKEESIAALPVDELIEKADGFAGVFPEHKYEIVKRLQARKHICGMTGDGVNDAPALKKADIGIAVADATDAARSASDIVLTEPGLSVIISAVLTSRAIFQRMKNYTIYAVSITIRIVLGFMLLALIWKFDFPPFMVLIIAILNDGTIMTISKDRVKPSPLPDSWKLAEIFATGIILGGYLAMMTVIFFWAAYKTDFFPSTFGVKSLQKTDRKDIKMLASAVYLQVSIISQALIFVTRARSWSFLERPGFLLVLAFVVAQLIATLIAVYANWGFAAIEGIGWGWAGVIWLYNLIFYIPLDFIKFFIRYALSGKAWDLVIEQRVCILFN

>GrECA4

MGKGGENHGKREFVNSKPTDPDVFPAWAKDIHECEKHYDVSQKSGLSTAEVENRRRIYGNNELEKHEGQSIWSLIMEQFNDTLVRILLLAAVVSFVLAWYDGDEGGEMEITAFVEPLVIFLILIVNAIVGVWQENNAEKALEALKEIQSEQATVIRDGIKIPSLPAKELVPGDIVELKVGDKVPADMRVLELVSSTLRVEQGSLTGESEAVNKTNKSVSEDADIQGKRSMVFAGTTVVNGNCFCLVTQIGMETEIGKVHTQIHVASQSEEDTPLKKKLNEFAEVLTMIIGVICIFVWLINVKYFLSWEYIDGWPRNFKFSFEKCTYYFEIAVALAVAAIPEGLPAVITTCLALGTRKMAQKNALVRKLPSVETLGCTTVICSDKTGTLTTNQMAVAKLVAMGGHASSLRNFRVDGTTYNPSDGKIHGWPSGGMDANLETIAKISAICNDAGVTHSDNKYVAHGMPTEAAIKVLVEKMGLPKGLYSGGAAGNDVLRCCQWWNEYEHRIATLEFDRDRKSMGVIVKSKSGRRSLLVKGAVENLLERSSKMQLLDGSVVPLDQNSRILVSNALQDMSSGALRCLGFAYKDELPEFETYDGSDDHPAHALLLDPSNYPSIESNLTFVGLVGLRDPPRQEVHQAIEDCKAAGIRVMVITGDNKNTAEAICREIGVFGPMEDISSKSLTGKEFMALSDQKAHLRQSGGLLFSRAEPRHKQEIVRLLKEDGEVVAMTGDGVNDAPALKLADIGIAMGIAGTEVAKEASDMVLADDNFSTIVSAVGEGRSIYDNMKAFIRYMISSNIGEVASIFLTAALGIPEGLIPVQLLWVNLVTDGPPATALGFNPPDKDIMKKPPRRSDDSLITAWILFRYLVIGLYVGVATVGVFIIWYTHGSFLGIDLSGDGHTLVTYPQLANWAQCSSWKNFTVSPFTAGNQVFSFENNPCDYFQGGKVKAMTLSLSVLVAIEMFNSLNALSEDGSLLTMPPWVNPWLLLAMSVSFGLHFLILYVPFLAQVFGIVPLSFNEWLLVLAVAFPVILIDEVLKFVGRLGRRMRSSSQRPLKPKTE

>GrAHA9

MASNGDISLEQIKNETVDLERIPVEEVFQQLKCTREGLTSEEGEKRLQIFGPNKLEEKEESKFLKFLGFMWNPLSWVMEAAAIMAIVLANGGGKPPDWPDFIGIVSLLFINSTISFIEENNAGNAAAALMAGLAPKTKVLRDGKWGEQEAATLVPGDVINVKLGDIIPADARLLEGDALKIDQSALTGESLPVTKNPGDEVFSGSTCKQGEIEAVVIATGVHTFFGKAAHLVDNTDNVGHFQKVLAAIGNFCICSIAVGMLIEILVMYPIQHRRYRDGIDNLLVLLIGGIPIAMPTVLSVTMAIGSHSLSQQGAITKRMTAIEEMAGMDVLCSDKTGTLTLNKLTVDKSLIEVFVSDMDADALVLIAARASRVENQDAIDASIVGMLGDPKEARAGITEVHFLPFNPVDKRTAITYIDRNGEWHRCSKGAPEQIIELCELTGGLRQKAHHVIDSFAGRGLRSLGVARQTIPEKKKECAGGPWEFVGLLPLFDPPRHDSAETIRQALDLGVNVKMITGDQLAIGKETSRRLGMGTNMYPSSSLLGQCKDEAIATIPVDELIETADGFAGVFPEHKYEIVKRLQELKHICGMTGDGVNDAPALKKADIGIAVADATDAARSASDIVLTEPGLSVIVSAVLTSRAIFQRMKNYTIYAVSITIRIVMGFMLIALIWEFDFSPFMVLIIAILNDGTIMTISKDRVKPSPTPDSWKLKEIFITGVVLGAYMAIVSVIFFWLVHDTLFFTEKFGVKPINENANELTSALYLQVSIISQALIFVTRSRSWSFVELPGLLLLGAFLAAQLVATCIAVYANWGFARIEGIGWEWAGVIWLFSVITYIPLDILKFIIRYSLTGKAWDNLLQKKAAFTTKKNYGKEAREAQWAADQRTLHGLSPPETSWTNEKDHHELSEIAEQAKKRAEIARLRELHTLKGHVESVVKLKGIDIDTIQQHYTV

>GrAHA10

MASDGDISLEQIKNEIVDLENIPVEEVFKLLKCTKEGLTTAEGETRLSIFGHNKLEEKKDNKVLKFLGFMWNPLSWVMEAAAIMAIVLANGGGKPPDWPDFVGIVTLLLINSTISFIEENSAGNAAAALMAGLAPKTKVLRDGKWSEQEASILVPGDIISVKLGDIIPADARLLEGDPLKIDQAALTGESLPVTKNPGDSVFSGSTCKQGELDAIVIATGIHSFFGKAAHLVDNTNNVGHFQQVLTSIGNFCIFSIGIGMVIEIIVMYPIQHRNYRDGIDNLLVLLIGGIPIAMPTVLSVTMAIGSHRLSEQGAITKRMTAIEEMAGMDILCSDKTGTLTLNKLTVDKNLVEVFINDMDVDTLVLLAARASRVENQDAIDACIVGMLSDPKQAREGITELHFLPFNPVDKRTAITYTDSKGEWHRCSKGAPEEIIDLCGLTGGLRKKALSIIDGYANRGLRSLGVARQTIPEKTKESPGGPWEFVGLLPLFDPPRHDSAETIKRALELGISVKMITGDQLAIGKETGRRLGMGTDMYPSSALLGQCGDEDIAAIPIDELIEKADGFAGVFPEHKYEIVRRLQERKHICGMTGDGVNDAPALKKADIGIAVADSTDAARGASDIVLTEPGLSVIVSAVLTSRSIFQRMKNYTIYAVSITIRIVLGFMLVALIWKFDFSPFMVLVIAILNDGTIMTISKDRVRPSPKPDTWKLDEIFATGVVLGTYMAIVTVFFFWLVHDTEFFTRTFGVKPINDNEDALTTALYLQVSTVSQALIFVTRSRSWSFIELPGPLLLIAFILAQLVATLLAVYANWGFARIQGIGWEWAGAIWVFSIITYIPLDVLKFFIRYALTGDAWGDVVQSKTAFKGECEVQLIANYRPLQGVSSPPETWSNDELAPIV

>GrECA5

MGRGEEDHGKREKISAASSKVENFPAWAKDVKQCEENFQTNRELGLSSAEVEKRREIYGWNELEKHEGTSIFQLILEQFNDTLVRILLLAAIISFVLAWLDGDEGGEKEITAFVEPLVIFLILIVNAIVGIWQESNAEKALEALKEIQSEQANVVRDGKKVSNLHAKELVPGDIVELRVGDKVPADMRVLTLISSTVRVEQGSLTGESEAVSKTAKVVPENTDIQGKKCMVFAGTTVVNGNCICMVTQIGMNTEIGKVHSQIHEASQSDDDTPLKKKLNEFGEVLTMIIGVICALVWLINVKYFLSWEYVDGWPSNFKFSFEKCTYYFEIAVALAVAAIPEGLPAVITTCLALGTRKMAQKNALVRKLPSVETLGCTTVICSDKTGTLTTNQMAVSKLIAMGSRPGTLRAFDVEGTTYNPFDGKIRGWAAGEMDANLQMIAKICAVCNDAGVEQSGSHYVATGMPTEAALKVLVEKMGLPEENGSSSGHGDHQRCCQAWNKLEQRIATLEFDRDRKSMGVIVNSSTGQKALLVKGAVENLLERSSFMQLRDGSIIELDQYSKDLILQSLHEMSTDALRCLGFAYKEEPFEFTTYNGDEDHPAHQLLLNPSNYSSIESKLIFAGLVGLRDPPRKEVRQAIEDCKAAGIRVMVITGDNKNTAEAICREIGVFGSSEDISSRSLTGKNFMDHPNQKNHLRQSGGLLFSRAEPRHKQEIVRLLKEDGEVVAMTGDGVNDAPALKLADIGVAMGIVGTEVAKEASDMVLADDNFSTIVAAVGEGRSIYNNMKAFIRYMISSNIGEVASIFLTSALGIPEGMIPVQLLWVNLVTDGPPATALGFNPPDKDIMKKPPRRSDDSLITAWILFRYLVIGLYVGIATVGVFIIWYTHHSFLGIDLSGDGHSLVTYSQLANWGKCDSWEGFSVSPFTAGSQVFKFDSDPCDYFHSGKIKASTLSLSVLVAIEMFNSLNALSEDGSLLTMPPWVNPWLLLAMSVSFGLHFLILYVPFLARVFGIVPLSTNEWLLVIAVAFPVILIDELLKFIGRRTTKLRYPAVPKSSKQKAE

>GrECA6

MGRGGENYGKRENAGAASSKQETFPAWARDVKQCEEKYQVNRELGLPSAEVEKRRQIYGLNELLKHEPTSIFQLLLEQFNDTLVRILLAAAIISFVLAWYDGEGRGEKEITAFVEPLVIFLILIVNAIVGIWQESSAEKALEALKEIQSEHADVIRDGKKVSSLPAKELVPGDIVELRVGDKVPADMRVLSLISSTVRVEQGSLTGESEAVSKTAKVVPENSDIQGKKCMVFAGTTMVNGNCICLVTEIGMNTEIGKVHSQIHEASQSDTDTPLKKKLNEFGEVLTLIIGMICILVWLINVKYFLSWEYVDGWPTNFKFSFEKCTYYFEIAVALAVAAIPEGLPAVITTCLALGTRKMAQKNALVRKLPSVETLGCTTVICSDKTGTLTTNQMAVSKLVAIGSRSGTLRSFDVEGTTYDPFDGKILGWPVDGMDANLEMIAKISAVCNDAGVEQSGRHYVAIGMPTEAALKVLVEKMELPEKYASSSAPGDPRRCCQVWNKMVQRIATLEFDRDRKSMGVIINSSSGNKSLLVKGAVENLLERSSFIQLLDGSTVELDKYSKDLILQVLREMSTDALRCLGFAYKEELPEFATYNSDEDHPAHQLLLNPSNYSSIESNLIFVGLVGLRDPPRKEVRQAIEDCKAAGIRVMVITGDNKNTAEAICREIGVFGYREDITSRSLTGKEFMDHPDQRNHLRQNGGLLFSRAEPRHKQEIVRLLKQDGEVVAMTGDGVNDAPALKLADIGVAMGITGTEVAKEASDMVLADDNFSTIVAAVGEGRSIYDNMKAFIRYMISSNIGEVASIFLTAALGIPEGMIPVQLLWVNLVTDGPPATALGFNPQDTDIMKKPPRRSNDSLITAWILFRYLVIGSLLNVVNACA

>GrHMA3

MVANKNLQKSYFDVLGLCCSSEVPLIENILKSLEGVKEVSVIVPTRTVIVLHDNLLLSQLQIVKALNQARLEANVRAHGEIKYQKKWPSPFAVVCGLLLLLSFLKYVYHPLQWLAVGAVVIGIYPVLFKGFAAITHFRIDINILILIAVIGSVAMKDYTEAGTIVFLFTIAEWLESRASHKASAVMSSLMSITPQKAVIAENGEEVDVDEVKLNTLLAVKAGEAIPIDGIVVDGRCEVDEKSLTGESLPVTKEKDSTVWAGTINLNGYISVKTTAVAEDCVVAKMAKLVEEAQNSKSNTQRFIDKCAQFYTPAIIIVSAAIAVIPTALRVKNIHHWFHLALVVLVSACPCSLILSTPVASFCALTKAATSGLLVKGGDYLETLSKIRITAFDKTGTLTRGEFIVTDFQPLSQDISLDTLLYWVSSIESKSSHPMAAALVDYGRSHSIEPNPETVEDYQNFPGEGIYGRVDGRDIYIGSKKISVRAHGTVPTLEGNMMKGKTIGFVYSGATPAGIFSLSDACRTGVPEAVDELKSMRIKVAMLTGDNQAVAIHVQEQLGNRLD

>GrAHA4

MAGLAPKTKVLRDGKWSEQEAAILVPGDIISIKLGDIIPADARLLEGDPLKIDQSALTGESLPVTKNPGDEVFSGSTCKQGEIEAVVIATGVHTFFGKAAHLVDSTNNVGHFQKVLTAIGNFCICSIAIGMLIEIIVMYPIQHRKYRDGIDNLLVLLIGGIPIAMPTVLSVTMAIGSHRLSQQGAITKRMTAIEEMAGMDVLCSDKTGTLTLNKLSVDRNLIEVFVKDVDKEHVVLLAARASRTENQDAIDAAIVGMLADPKEARAGIREVHFLPFNPVDKRTALTYIDSNGNWHRASKGAPEQILTLCNAKEDVKKKVHAIIDKFADRGLRSLGVARQQVPEKSKDGAGTPWQFVGLLPLFDPPRHDSAETIRRALNLGVNVKMITGDQLAIAKETGRRLGMGTNMYPSASLLGQDKDASIAALPVEELIEKADGFAGVFPEHKYEIVRKLQERKHICGMTGDGVNDAPALKKADIGIAVADATDAARSASDIVLTEPGLSVIISAVLTSRAIFQRMKNYTIYAVSITIRIVFGFMLIALIWKFDFSPFMVLIIAILNDGTIMTISKDRVKPSPLPDSWKLKEIFATGIVLGGYLALMTVVFFWIMHDTDFFSEKFGVRSLRKKDDQMMGALYLQVSIVSQALIFVTRSRSWSYFERPGLLLVTAFIIAQLAATVIAVYANWGFARIQGIGWGWAGVIWLYSVVFYIPLDLMKFAIRYILSGKAWLNLLENKTAFTTKKDYGKEEREAQWALAQRTLHGLQPPEASNLFNDKSSYRELSEIAEQAKRRAEVARLRELHTLKGHVESVVKLKGLDIDTIQQHYTV

>GrHMA2

MAANKKKLQRSYFDVLGLCCSSEVPLIENILKPLEGVKQVSVIVPTRTVIVVHDNLLVSQLQIVKALNQARLEANVRAHGEIKYQKKWPSPFAMACGLLLLISFFKYVYRPLQWVAVGAVVIGICPILLKGYAAITNFRLDINILMLIAVIGSIAMKDYTEAATIVFLFTIAEWLESRASHKATAVMSSLMSMSPQKAVIAESGEEVDVDEVKLNTVLAVKAGEVIPIDGIVVDGNCEVDEKTLTGESLPVSKQKDSTVWAGTINLNGYISVKTTAVAEDCVVAKMAKLVEEAQNSKSTTQRFIDKCAQFYTPAIIVVSVAIAVIPAAFRVHNLRHWFHLALVVLVSACPCALILSTPVASFCTLTKAATSGLLVKGGDYLEILSNIKITAFDKTGTLTRGEFVVTNFRSLCQDISFNSLLYWVSSIESKSSHPMAAALIEYGRSHSIEPKPETVEDYQNFPGEGIYGRIDGRDIYIGSRKVSVRAHGTAPNVEGNMMEGKTIGYVFCGATPAGIFSLSDACRTGAAEAVNELKSMGIKTAMLTGDNQAAAIHVQEQLGNSLDVIHADLLPQDKARIVEEFKKEGPTAMLGDGINDAPALATADIGISMGISGSALATETGHVILMSNDIRKIPKAIRLARKAHRKVIQNVILSISTKVAILALAFAGHPLVWAAVLADVGTCLLVIFNSMLLLHGTHKHAGKCSKSSAASHKDKQGCNTSHCHSSHNHEHSSIDKKVQKACEPQKCSSRSCASRCQSNPSNSDASSNSCGSNKCTESTGTREMKHCDQGSCNIVNHKIEAHNLPSKCCSSHGKLGEKHCHHSSNQQGTKADQCHSTCGGNHTDRQTLGTFVEHSCLESPKPEAHPYSNKCFTDYWESPHTAIDIPMNTSHETAQACMSVEKREMGGCCKSYMKECCGKHGHFRSGLTEIVTE

>GrECA1

MEDAYARSVSEVLDFFGVDSSKGLTDFQVSQHARLYGKNVLPEEERTPFWKLVFKQFDDLLVKILIAAALVSFLLALINGETGLIAFLEPSVILMILAANAAVGVITETNAEKALEELRAYQADIATVLRNGCFSILPATELVPGDIVEVSVGCKIPADMRMIEMLSGQLRVDQAILTGESSSVEKDLESTIATNAVYQDKTNILFSGTVVVAGRARAVVIGVGANTAMGSIRDSMLRTDDEATPLKKKLDEFGTFLAKVIAGICVLVWIVNIGHFRDPAHGGFLRGAIHYFKIAVALAVAAIPEGLPAVVTTCLALGTKRMARLNAIVRSLPSVETLGCTTVICSDKTGTLTTNMMSVSKICVVHSIKNGPEVAEFGVSGTTYAPEGFIFDNTGVQLEFPAQLPCLLHLAMCSALCNESLLQYNPDKGNYEKIGESTEVALRVLAEKVGLPGFDSMPSALNMLSKHERASYCNHYWENQFKKVSVLEFSRDRKMMSVLCNHKQMEIMFSKGAPESIISRCTNILCNNDGSTIPMDATLRAELDSRFNSFAGKETLRCLALALKIMPMGQQILSFDDEKDLTFIGLVGMLDPPREEVRNAMISCMTAGIRVIVVTGDNKSTAESVCRKIGAFDHLVDYVGHSYTAAEFEELPGTQQTMALQRMALLTRVEPSHKRMLVEALQNQNEVVAMTGDGVNDAPALKKADIGVAMGSGTAVAKSASDMVLADDNFATIVAAVAEGRAIYNNTKQFIRYMISSNIGEVVCIFVAAVLGIPDTLAPVQLLWVNLVTDGLPATAIGFNKPDSDVMKAKPRKVSEAVVSGWLFFRYLVIGAYVGLATVAGFIWWFIYSETGPKLPYTELMNFDTCPTRETTYPCSIFEDRHPSTVAMTVLVVVEMFNALNNLSENQSLLVIPPWSNLWLVASIILTMLLHILILYVPPLSTLFSVTSLSWNEWAVILYLSFPVIIIDEVLKFFSRNSHGNAYCSFGSVSADLINNHVSYICDEHTIYCIMFLIIFKRSAATIKYSFVYFDLL

>GrHMA8

MGTKLLALACIRKESYGDLSPRPHYPSMPKYPKGITAQETSLQGSEAKAMFSVMGMTCSACAGSVEKAVKRLPGIKEAVVDVLNNKAQVMFYPSFVNEESILEAIEDAGFQAALIQDETDDKSVQVCRIRINGMTCTSCSTTLENALQPVPGVQKVQVALATEEAQIHHDPKIITYNQLMQKIEETGFGAVLVSTGEDMSKINLRIDGVRTVNSMRMLENSLQALPGVQAVQTSPELKKIAVSYKPDMTGPRNFIKVIDSTGSSRRFKATIYPEGEGAGRESHRKEEIKQYFRSFLWSLIFTTPVFLTSMVFMYIPGIKHGLDTKVVNMLTIGEVIRWVLSTPVQFIIGRRFYTGSYKALRHGSANMDVLIALGTNAAYFYSVYTVIRAASSPDFEGTDFFETSAMLISFILLGKYLEVLAKGKTSEAIAKLMNLAPETAILLSLDEEGNVISEEEIDSRLIQKNDIIKIIPGAKVASDGFVLWGQSHINESMITGEARPVAKRKGDTVIGGTVNENGVLHIKATKVGSESALAQIVRLVESAQMAKAPVQKFADRISKYFVPLVIMLSFSTWLAWFLAGKLHGYPESWIPSSMDSFELALQFGISVMVIACPCALGLATPTAVMVGTGVGASLGVLIKGGQALEGAHKVNCIVFDKTGTLTVGKPVVVNTRLLKNMVLHEFYELVAATEVNSEHPLAKAIIEYAKKFREDEENPAWPEARDFVSITGHGVKAIVRNKEVIVGNKSLMLENNIVIPVDAQDMLTETELMAQTGILVSIDGEVTGVLAISDPVKPGAQEVISILKSMNVRSIMVTGDNWGTASSIASQIGIETVVAEAKPEQKAEKVKELQAEGYAVAMVGDGINDSPALVAADVGMAIGAGTDIAIEAADIVLMKSNLEDVITAIHLSKKTFSRIRLNYIWALGYNILGIPIAAGALFPSTGFRLPPWIAGAAMAASSVSVVCCSLLLKNYERPKKLENLEIGGIQIE

>GrACA6

MSSSDECKLYDCSTSLLNVTAPSGFTVAQRRWRIAYIRIYSSRVMLSLADKIISQRDTQLPSMTSQQFDHYVAEFDHYVALDIDHKINQKRLVKTVKEKDLVSLNHLRGVDGVVDALCTNSEHGIRDDEQEVIKRQEMFGFNKYHKPPPKGLLYFVLEAFKDTTILILLVCAALSLGFGIKEHGAEEGWYEGGSIFVAVFLVIVVSALSNFRQETQFDKLSKISNNIKVEVVRSGRRQQISIFDLVAGDVVFLKIGDQIPADGLFLDGHSLQVDESSMTGESDHVEVDACRNPFLSSGSKVVDGYARMLVASVGMDTAWGEMMSSITSDKNERTPLQARLDKLTSSIGKVGLVVAFLVLAVLLIRYFTGNTKDDNGQTEYHGSETDVDDILNAVVRIVAAAVTIVVVAIPEGLPLAVTLTLAYSMKRMMADQAMVRKLSACETMGSATIICTDKTGTLTLNQMKVTQFWLGQESVEEDLAKKIAPSVLELFYQGVGLNTTGSVCIPLSGSLPEFSGSPTEKAILSWAVLGLGMNIEKLKQQYSILHVETFNSEKKRSGVSVQRKADEMVHIHWKGAAEMIVAMCSQYYESNGIIRSMNEDGRDRIETIIQSMAASSLRCIAFAHKQVLKGETEDGDDQSRKTNRGIKEDGLTLLGIVGLKDPCRPGVKKAVQACQSAGVSIKMITGDNIFTAKAIATECGILGPDYQQGSGEVVEGIEFRNYAPDERMEKVEKIRVMARSSPFDKLLMVQCLKQKGHVVAVTGDGTNDAPALKEADIGLSMGIQGTEVAKESSDIVILDDNFSSVATVLRWGRCVYNNIQKFIQFQLTVNVAALVINFIAAVSAGEVPLTTVQLLWVNLIMDTLGALALATDRPTNELMEKPPVGRTEPLITNIMWRNLLAQALYQIAILLILQFRGESIVNVPETVKDTLIFNTFVLCQVFNEFNARKLEKQNVFEGILKNRLFLGIIGVTIVLQVVMVEFLKKFADTEQLKLWQWGVCILLAAFSWPIAWFVKLIPVSNTPFFSYLKRLRIIYTIVKRPINHQKP

>GrHMA6

MSPGSRDLQLTSQAAGVWRSTYPSSVRAVDPDDMEEGTRLLDSYETGDYKLESIEEGSMRRIQVTVTGMTCAACSNSVEAALKNINGVLRASVALLQNRADVVFDPTLVKDEDIKNAIEDAGFEAEILPEPSNVGTKPRGVLVGQFTIGGMTCAACVNSVEGILRDLPGVSRAVVALATSLGEVEYDPTVISKDDIVNAIEDAGFEASLVQSSEQDKIILGVAGVFNELDVQLIEGILSSLKGVRQFRFDRSSGELEVLFDPEVVSSRSLVDGIEGGSKGKFRLHVMNPYARMTTKDEETSIMFQLFTSSLFLSIPVFLIRVVCPHIPLLDAFLLWRCGPFLMGDWLKWALVSVVQFVIGKRFYVAAGRALRNGSTNMDVLVALGTSASYFYSVGALLYGAITGFWSPTYFETSSMLITFVLLGKYLECLAKGKTSDAIKKLVELAPATALLVVKDNGGNIIGEREVDALLIQPGDILKVLPGAKLPADGVVVWGSSYVNEGMVTGESVPVSKEVDSPVIGGTINLHGALHIKATKIGSEAVLSQIISLVETAQMSKAPIQKFADFVASIFVPTVVTLSLITLLGWYVGGAARAYPEQWLPENGNYFVFALMFSISVVVIACPCALGLATPTAVMVATGVGASNGVLIKGGDALERAQKVQYVIFDKTGTLTQGKAKVTTVKVFSEMDRGEFLTLVASAEASSEHPLAKAIVEYARHFHFFDENSLTEDAQYSSKGSPISAWLLDVAEFSAVPGRGIQCFIDGKQVLVGNRKLLTESGVSISAHVEQFVVDLEERARTGILAAYDGNVIGVLGVADPLKREAAVVVEGLQKMGVRPVMVTGDNWRTAQAVAREVGIRDVRAEVMPAGKAEVVRSFQKDGSIVAMVGDGINDSPALAAADVGMAIGAGTDIAIEAADYVLMRNNLEDVITAIDLSRKTFSRIRWNYVFAMAYNVVAIPIAAGVLYPSLGIKLPPWAAGACMALSSVSVVCSSLLLRRYKKPRLTTILEITVE

>GrHMA7

MTFSFVGAMNINVGDNKMELNGRDDLKRPLLEPSDSVCVTIPEPVDKLEKKRTVMFKIGNIKCASCVTSIESVLGEINGVESVSVSPIHGHAAIEYVPKLVNPKLIKETIEDAGFPVKEFSEQQIAVCRLRIKGMACTSCSESLERALKFLDGVKKAVVGLALEEAKVHFDPNITDSDRIIEAIEDAGFGADLISSGNEANKVHLKLEGVSSVEDMNTIKSYLESAIGVNHVEMDLEEKRATVNYDPDFTGPRSIIEAVQEVAHGSYKASLYIPPRQRETEQHHEINNYRNQFLLSCLFSVPLFIFSMVLPMLPPFGDWLEYKIYNMFTVGLLLRWVLCTPVQFIVGRRFYKGSYHALRLKSANMDVLVAMGTNAAYFYSVYVAIKSLSSDTFKGQDFFETSAMLISFILLGKYLEVLAKGKTSDALAKLTDLAPDSACLLILDDDGNVVSEVAISTQLIQRNDIIKIIPGEKVPVDGIVIDGQSYVNESMITGEAQPIAKKPGDKVIGGTMNENGCLLVKATHVGSETALSQIVQLVEAAQLARAPIQKIADRISRFFVPAIVLTAFITWLGWLIPGVIGIYPKHWIPKGMDKFELALQFGISVLVVACPCALGLATPTAVMVATGKGASLGVLIKGGNALEKAHKVKAIVFDKTGTLTVGKPEVVNVMLFSSVSMEDFCDVAIAAEANSQHPIAKAFLEHARKLRQKIESNRQSNNQHVTEAKDFEVHPGTGVSGKVGDKMVLVGNKRLMQTYNVTVGPEIEGYISEHEQQARTCVLVSIDGKIAGAFAVTDPVKPEAKNVILYLHSMGISSIMVTGDNWATATAIAKEVGIEKVIAEMDPIGKADRIKDLQMRGLTVAMVGDGINDSPALVAADVGMAIGAGTDVAIEAADIVLIKSNLEDVVTAIDLSRKTISRIWLNYVWALGYNILGVPVAAGILYPFTGVRLPPWLAGACMAASSLSVVCSSLLLQSYRKSWVFQDTKSGHSHCSKST

>GrACA4

MEELLKGFEVPPKNSSEAALRRWRKLVTIVRNPRRRFRMIANLEKRSEAEQQKLKIKEKIRVALIVQKAALQFIDAAGPLDYKITDEVRQANFGIEPDELASIVHGHDIKRLKSHGGVDGIAEKVTVSLDEGVLSENVSTRQRIYGFNQYTEKPPRTFWMFVWDALQDLTLIILMICAVVSIGVGLATEGWPKGMYDGAGILLSIILVVLVTAISDYRQSLQFRDLDREKKKISVQVTRDGRRQQVSIYDLVVGDVVHLGIGDQVPADGLFISGYSMQIDESSLSGEADPVDIYEQKPFLLSGTKVRDGSAKMLVTAVGMRTEWGKLMETLNEGGEDETPLQVKLNGVATIIGKIGLTFAVLTFLVLTVRFLIEKALHNEFTKWSSTDALTLLDYFAIAVTIIVVAVPEGLPLAVTLSLAFAMKQLMDERALVRHLSACETMGSASCICTDKTGTLTTNHMVVNKIWICEKISNIGGNENKNIDELEIHESVFSIFLRSIFLNSSAEVVKDENGKNSILGTPTETALLEFGLLLSADHDAYRRQFKILKVEPFNSDRKKMSVLVALPEGRIQAFCKGAPEIVLRMCEKVVDSSGEVVLLSEERVRDITEAINGFASDALRTLCVAVKDVGETFNENGIPDSGYTLIAVFGIKDPVRPGVKEAVQTCLAAGITVRMVTGDNINTAKAIAKECGILTAEENAIEGPEFSSKSPDEMKDIIPNIQVMARSKPSDKLNFVTNLRNMFGEVVAVTGDGTNDAPALRQSDIGLAMGIAGTEVAKENADVIVMDDNFATIVNVAKWGRAVYINIQKFVQFQLTVNVVALIINFVSACISGSAPLTAVQLLWVNMIMDTLGALALATEPPNDALMKRPPVPRGASFITKPMWRNIIGQSIYQLIVLGVLNFDGKQLLRLTGSDATTVLNTVIFNSFVFCQVFNEINSREIEKINILRGMFSSWVFLGVMASTVAFQVVIVEFLGTFASTVPLSWQLWLLCILIGSVSLIVGVIVKCIPVERAAVKPKHHDGYDALPSGLA

>GrHMA4

MTGLLSSLPSQARLMVDDSIVEVPCSSLSVGDQIVVLPGDRVPADGIVRAGRSTIDESSFTGEPMPVTKEPGSQVAAGSINLNGTLTIEVRRPGGETAMGDIVRLVEEAQSREAPVQRLADKVAGHFTYGVMALSAATFMFWNLFGARIIPASIYQGSAVSLALQLSCSVLVVACPCALGLATPTAMLVGTSLGATRGLLLRGGNILEKFSMVNVIIFDKTGTLTIGRPVVTKVVTPSRMDHSDSRQHFDGSWSEDDVLKLAAAVESNTIHPVGKAIVEAAQAVKSPNIKVVDGTFVEEPGSGAVAVIDDKTVSVGTLEWVQRHGVGDSLLLETDEELRNKSVVYVGVNNKLAGLIYFEDQIREDARHVVDSLHRQGISVYMLSGDKRSTAEYVASIVGIPKDKVLSQVKPDEKRKFVSELQENQNVVAMVGDGINDAAALASAHIGVAMGGGVGAASEVSSIVLMGNRLSQLLDALALSQLTMKTVKQNLWWAFAYNIVGIPIAAGTLLPLTGTMLTPSIAGALMGLSSIGVVTNSLLLRFRFSLQQQQAYRSSLQPPPHAAMDINNDLAKDHSRAKLKKPDSIT

>GrACA5

MEDYLRKNFAVEPKRPSEEALRRWRSAVALVKNRSRRFRMVADLAKRAEADRRRKIIQEKIRVALYVQKAALNFIDAGKQAESKLPEDVRKAGFHIGADELASIVRSHDMSSFEEHGGVEGLAKKVSVSLTNGVVPTDISFRQNIYGNNKFDEKPARSFWMFVWEALHDLTLIILIVCAVVSIGVGVATEGWPGGLYDGLGIVLCIFLVVFVTAISDYKQSLQFKDLDKEKKNILVQVTREGCRQKISIYDLVVGDIVHLSIGDQVPADGVLISGYSLSIDESSLSGESEPVKVTQERPFLLSGTKVQDGSGKMLVTTVGMRTEWGRLMVTLSEGGVDETPLQVKLNGVATVIGKIGLVFAVLTFLVLTIRFMVTKAQLGEIEKWGMSDVLVLLNFFAVAVTIIVVAVPEGLPLAVTLSLAFAMKKLMSDKALVRHLSACETMGSATCICTDKTGTLTTNHMVVDKIWTCGRTISIAGDNKREDVLRSSIAGEVLDLLLQSIFQNTGAEVVKGKDGKNNILGSPTETAILEFGLLLGGEFKKYRKESTILKVEPFNSEKKRMSVLVSLSNGGENRAFCKGASEIILESCNKVINVDGKAEHLSKEQKKYITDVINGFACEALRTLCLAFKDVKDTSDVHSDSIPQENYTLIAVIGIKDPVRPGVRQAVETCLSAGIKVRIVTGDNINTAKAIARECGILTENGLAIEGPEFRDMSPRQMEETIPKLQVLARSLPLDKHKLVTYLRKEFKEVVAVTGDGTNDAPALHEADIGLAMGIAGTEVAKENADVIIMDDNFATIQNVARWGRAVYINIQKFVQFQLTVNIVALMLNFVSACISVIFRFCPSDGCSATLGEHDHGHSRCIGIGHRTSS

>GrHMA5

MAMAGDLLRLSVLNRPKLSFSNGAKLKADRFGLLKRCPRGRFHCRPRSTPGFVLFSSLETRLESEESSIQPVGQKLKDPSVLLDVNGMMCGGCVSRVKSVISSDERVESVVVNLLTETAAIKLKREVMERETVESVAESIAQRVSECGFMAKRRVSGIGIAENMRKWKEMLKKKEELLVKSRNRVAFAWTLVALCCGAHASHILHSLGIHFGHGSFLEVLHNSYVKGGLALTALLGPGRDLLVDGLLAFKKGSPNMNSLVGFGSIAAFIISAVSLLNPGLEWDASFFDEPVMLLGFVLLGRSLEEKARIRASSDMNELLSLISTRSRLVITSSDTDSSADSVLSSDAICIEVPSDDIRVGDSVLVLPGETIPVDGKVLTGRSVVDESMLTGESLPVFKEKGLTVSAGTINWDGPLRIGATSTGSNSTIAKIVRMVEDAQGQEAPVQRLADAIAGPFVYSIMTLSAATFAFWYYAGSHIFPDVLLNDIAGPDGDPLLLSLKLAVDVLVVSCPCALGLATPTAILVGTSLGARQGLLIRGGDVLERLANVDRIAFDKTGTLTEGKPTVSSVSSFTYDESEILQIAAAVERTAIHPIAQAIVKKAELLNLVLPETRGQLVEPGFGTLAEVNGRLVAVGKLEWVNERFQIKASPSDLMALEHAVMRQSSSPSNYSKTAIYVGREGEGVIGAIGMSDSLRFDAESTVSRLQRKGIKTILISGDREEAVATIAKTVGIEHEFVNASLTPQQKSRVISTLQTAGHHIAMVGDGINDAPSLALADVGIALQTEAQETAASDAASIILLGNRLSQVVDALDLAQATMAKVYQNLSWAVAYNIVAIPIAAGVLLPQYDLAMTPSFSGGLMALSSIFVVTNSLLLRLHGSEKSWKNSIAKISQMPAGPG

>GrACA10

MSLRHRNTGYPEPAMSDGEDTPIVKCQHRRWRSVFAAIYSARIFVSLYKKIINKKQILRSLSYIALDVHDSDSSDDRLPSLSVDQKTLTEVVREKSLGTLSKLGGVKQIAASLETDEKDGISTNEADLAHRVDVFGANRYQKPPKKSFFSFVYEAFKDTTIIILLVCAVLSLGFGIKQHGITDGGYDGGSIIIAVFLVVAVSAVSNFKQNRQFEKLSKESRDIKVEVVRDGRRQFISVFEVVVGDVVCLKIGDQIPADGLFLDGHSLKVDESSMTGESDHVEINGSNNPFVLSGTKVTNGFGSMLVTSVGMNTAWGKMMSSINSELDEETPLQARLNKLTSAIGKIGLTVAVLVLAVLLIRYFTGNTKDDQGNKEYIRGKTKFDSMMNSVVEIISAAITIVVVAIPEGLPLAVTLTLAYSMKQMMADQAMVRKLSACETMGSATTICTDKTGTLTLNEMKVTEFWLGKELMGSISSEIAPNVHKLLQQAVALNTTGTVYKPNSRSLPEISGSPTEKAILSWAVSDLGMNLDDPKQNYELIQVEAFNSEKKRSGVLIRRNSENGGATQVHWKGAAEMILAMCSQYYDRSGAVKAIDEEERVEMGKIIEDMAAKSLRCIAFAHTKYPIDNERVLQESGLILLGLVGLKDPCRPGVRTAVEACINAGVNIKMITGDNIFTAKAIATECGILQPNEDMSEAVIEGVQFRNYSPEERMAKINRICVMARSSPFDKLLMVQCLKQNGHVVAVTGDGTNDAPALKEADIGLSMGIQGTEVAKESSDIIILDDNFTSVVTVLRWGRCVFNNIQKFIQFQLTVNIAALVINFIAAISSGEVPLTAVQLLWVNLIMDTFGALALATERPTNDLMTKPPVGRSKPLISNIMWRNLIAQALYQVAVLLTLQFRGKFIFDVDEKVKNTLIFNTFVLCQVVNEFNARKLEKKNIFQGLHKNKLFLGIIAITIILQVVMVEFLKRFANTQRLNWGQWGTCIGIAALSWPLGWLVKWIPA

>GrACA9

MSLRLRKPSEPTMHRQVEPSKSSVRRWRVAVTAISVTRFLVGLTKKVAEKNAELLRSLSFVTIDVEGSGDERVPILDVDPQGLAKMVKDKSFQSLNDQYGGVKQVATLLQTDFKTGIPGDDNDLALRTKVFGANKYQKQPAKSFFSFVLEAFKDTIIIILLVCAVLSLAFGIKQHGLKEGWYDGGSIIVAVVLVVVVSAVSNYRQSKQFEELSHETNDIRVQVVRNRRYQPVSIFELVVGDIVSLKTGDQIPADGLFVEGHSLKVDESSMTGESDHVEVNEKKNPFLLSGTKVTDGHGYMLVTAVGMNTAWGEMMSSIRRDLNEETPLQVRLSKLTSYIGNIGLSVAVLVLLVLLIRYFTGHTKAENGRSAFNGSRTKFDDVMNSVVSIIAAAVTIVVVAIPEGLPLAVTLTLAYSMKRMMRDHAMVRKLSACETMGSATIICTDKTGTLTLNEMKVTEFWLGKEPIDNSMSSEIAPNVLQLLSEGVGLNTTGTVYKPEPTSVPEIYGSPTEKAILSWALNDMGLNIDESKQSCEIIHVEAFNSEKKRSGVLIRRSNNKRVLATHWKGAAEMLLAMCSCYCDKKGVLKFMNEDERAHIGMVIESMAAKSLRCIAFATSDVTVTDGNEENHTKLEETGLTWLGLVGLKDPCRPGVKQAVESCKKAGVSIKMITGDNMHTARAIAFECGILNSESSLHNEAVVEGVQFRNYSEEERRQKIETIRVMARSSPFDKLLMVQCLKQKGHVVAVTGDGTNDAPALKEADIGLSMGIQGTEVAKESSDIVILDDNFTSVATVLRWGRCVYNNIQKFIQFQLTVNVAALVINFIAAVSSGDVPLTAVQLLWVNLIMDTLGALALATEQPTNDLMDKRPVGRTEPLITKVMWRNLIAQALYQVAILLILQFKGKSIFGVPEEVKDTLIFNTFVLCQIFNEFNARNMDKKNIFKGIHKNRLFLAIIGITLVLQAIMVEFLQRFANTERLSWEQWGACIGIAALTWPIGWIVKCIPVDKKVQTRSSAAS

>GrACA7

MTTILQSNLLCFEYTIQVPSATFPKSRKKWHSLFATIYCSRKFSSLITKTATANDEARVVHRSPSHVSLAVMQENSPFRIDQPTLIELVKEKKIEKLRKHGGVDGVASGLGTDTQVGVSGSAEDIERRREAFGSNTYKKPPTKGFFHFVVEAFKDLTIMILLGCAALSLGFGIKEHGLKDGWYDGGSIFVAVFLVIAVSAVSNYRQDRQFDKLSKVSNNIQVDVVRGGRRQQISIFDIVVGDIVCLKIGDQVPADGLFIDGHSLQIDESSMTGESDHVEVNGNQNPFLLSGTKVADGYARMLVTSVGMNTTWGQMMCQISRDTNDETPLQARLNKLTSSIGKVGLAVAFLVLVVLLVRYFTGHTTDENGNREFNGSKTKSDDIINAVVGIVAAAVTIVVVAIPEGLPLAVTLTLAYSMKRMMADQAMVRKLSACETMGSATTICTDKTGTLTLNRMKVTKFWLGQESMEEGASSISPFVVDLIHQGVALNTTGSVYRASPGTEYEFSGSPTEKAILSWAVVELKMDMEKTKKCYAVLQVEAFNSQKKRSGVLIGRNDDDTVHVHWKGAAEMILALCSSYYDASGVEKDLDDDERTKFEQIIQGMAASSLRCIAFAHKQVPEEEYQNLKEQKKLKEDNLTLLGLVGIKDPCRPGVKKAVEDCQYAGVNIKMITGDNVFTARAIATECGILKPGQDLSSGAVVEGEEFRSYTPHERMEKVEKIQVMARSSPFDKLLMVQCLKQKGHVVAVTGDGTNDAPALKEADIGLSMGIQGTEVAKESSDIVILDDNFASVATVLRWGRCVYTNIQKFIQFQLTVNVAALCINFVAAVSAGEVPLTAVQLLWVNLIMDTLGALALATERPTKELMEKPPVGRTEPLITNIMWRNLLAQALYQIAVLLTLQFSGESIFGVTEKVNDTLIFNIFVFCQVFNEFNARKLEKKNVFEGIHKNKLFIGIIGVTILLQVVMVEFLKRFADTERLTWGQWGACIAVAAVSWPLGWVVKCLPVPQKPIFSYLKWRK

>GrACA3

MESYLNENFGDVKPKNSSEEALERWRKLCWIVKNRKRRFRFTANLSKRFEAEAIRRSNQEKFRVAVLVSQAALQFIHGLNLSSEYDAPEEVKAAGFQICADELGSIVEGHDVKKLKIHGGVEDIAAKLSTSIVNGIPTSEHLVNERKRIYGINKFTETPPRGFWVFVWEALQDTTLMILAVCALVSLAVGITVEGWPKGAYDGLGIVLSILLVVFVTATSDYRQSLQFRDLDKEKKKITVQVTRDGLRQKISIFDLLPGDIVHLAIGDQVPADGLFISGFSVLINESSLTGESEPVSVNSRNPFLLSGTKVQDGSCKMLVTTVGMRTQWGKLMATLSEGGDDETPLQVKLNGVATIIGKIGLFFAVVTFAVLVQGLFSRKLQDGTQWIWSGDDAMEMLEFFAIAVTIVVVAVPEGLPLAVTLSLAFAMKKMMNDKALVRHLAACETMGSSTSICSDKTGTLTTNHMTVVKTCFCGQIKEVSTSNKNNHFRSAVPESAAKILIESIFNNTGGEVVNNKENKIEILGTPTETALLEFGLLLGGDFQAERKASKIVKVEPFNSAKKRMGVVIEFPEGGLRVHCKGASEIILAACDKVISSNGDVLPLDEPTTNHLKNTIEQFASEALRTLCLAYMDVGTNFSGDSSLPLQGYTCIGIVGIKDPVRPGVKESVAICKSAGITVRMVTGDNINTAKAIAREIGILTDDGIAIEGPVFREKSEEELYELIPKIQVMARSSPMDKHTLVKHLRTSLGEVVAVTGDGTNDAPALHEADIGLAMGIAGTEVAKESADVIILDDNFSTIVTVAKWGRSVYINIQKFVQFQLTVNVVALIVNFASACLTGNAPLTAVQLLWVNMIMDTLGALALATEPPNDDLMKRSPVGRKGNFISNVMWRNILGQSLYQFVIIWILQTRGKAAFHLDGPDSDLILNTLIFNSFVFCQVFNEISSREMEKINVLKGLLKNHVFVAVISCTIIFQIVIVEFLGTFASTSPLTVQQWFVSVCLGFLGMPIAAALKLIPVGSN

>GrACA2

MDSYLNQNFDLKSKHSSDEALEKWRTVVGFVKNPKRRFRFTANLSKRYEAAAMRRTNQEKLRIAVLVSKAAFQFISGVQPSDYVVPEQVIAAGFQLCADELGSIVEGHDVKKLKFHGGVSGIAEKLSTSTNTGLSSDAALLSKRQEIYGINKFAEPEAKGFWVFVWEALQDMTLMILGACAIVSLVVGIAMEGWPAGAHDGLGIVASILLVVFVTATSDYRQSLQFKDLDKEKKKITIQVTRNDCRQKLSIYDLLPGDIVHLNIGDQVPADGLFVSGFSVLIDESSLTGESEPVVVNDKNPFMLSGTKLQDGSCKMLVTTVGMRTQWGKLMATLSEGGDDETPLQVKLNGVATIIGKVGLFFAVVTFAVMVQGLLRSKLQEGTIWSWSGDEALKLLEFFAVAVTIVVVAVPEGLPLAVTLSLAFAMKKMMNDKALVRHLAACETMGSATGICSDKTGTLTTNRMTVVKSCICMSVKEINSTNKASFCSEIHESAIKLLLQSIFMNTGGEIVTSKDGRREILGTPTETALLEFGLSLGGDPQTERQASKTVKVEPFNSTKKRMGVVLELPGGGLRAHTKGASEIVLAGCDKVIDSNGEVVPLDEKSINHLNATINEFANEALRTLCLAYLELKNGFSSDNAIPVSGYTCLGIVGIKDPVRPGVKESVAICRTAGITVRMVTGDNINTAKAIARECGILTDDGIAIEGPDFREKSQEELLKLIPKIQVIARSSPMDKHTLVKQLRTTFDEVVAVTGDGTNDAPALHEADIGLAMGIAGTEVAKESADVIILDDNFSTILTVAKWGRSVYINIQKFVQFQLTVNIVALIVNFSSACLTGSAPLTAVQLLWVNMIMDTLGALALATEPPTDELMKRAPVGRKGNFISNVMWRNIFGQSFYQLMVIWYLQARGKAMFELDGPDSTLKLNTLIFNSFVFCQVFNEISSRNMEEINVLSGILNNSVFVAVLGCTAVFQIIIIEFLGTFASTTPLTYSQWGLSVVIGFFSMPIAAALKLVSV

>GrACA1

METFWNESFDLKPKHSSEEALEKWRKVVGFVKNPKRRFRFTANLSKRYEAAAMRRSNHEKLRIAVLVSKAALQFISGVKPSESDYVVPEEVKAAGFELCAEELGSIVEGQDVKKLKIHGGVDGIAEKLSTSTTDGLSSDSVLLNKRQEVYGINKFAEAEAKGFLVFVWEALQDMTLMILGVCALVSLIVGIAMEGWPKGAHDGLGIVASILLVVFVTATSDYRQSLQFKDLDKEKKKITIQVTRNACRQKMSIYDLLPGDIVHLNIGDQVPADGLFVSGFSVLIDGSSLTGESEPVMVNADNPFMLSGTKLQDGSCKMMVTSVGMRTQWGKLMATLSEGGDDETPLQVKLNGVATIIGKVGLFFAVVTFAVMVQGLFMSKLQEGTIWSWSGDEALKLLEFFAVAVTIVVVAVPEGLPLAVTLSLAFAMKKMMNDKALVRHLAACETMGSATNICSDKTGTLTTNHMTVVKSCICMDVREVGNNNKASLCSEIPESAVKLLLQSIFTNTGGEIVINKDGKREILGTPTETALLEFGLSLGGDSRAERLASKLVKVEPFNSTKKRMGVILELPEGGLRAHTKGASEIVLAGCDKVINSNGEVVPLDVESINHLNATINQFANEALRTLCLAYMELENDFSPDNAIPLSGYTCIGIVGIKDPVRPGVKESVAICRSAGITVRMVTGDNINTAKAIARECGILTDDGIAIEGPDFREKSQEEMLALIPKIQVMARSSPMDKHTLVRQLRSIDEVVAVTGDGTNDAPALHEADIGLAMGISGTEVAKESADVIILDDNFSTIVTVAKWGRSVYINIQKFVQFQLTVNIVALIVNFSSACLTGTAPLTAVQLLWVNMIMDTLGALALATEPPTDELMKRAPVGKKGNFISNVMWRNILGQSFYQFMVIWYLQVKGKGMFSLEGPDSDLTLNTIIFNSFVFCQVTFYYEHLDTAGKAYIVISMKLPYKTMLRNFPQVFNEISSRNMEEINVFKGILNNYVFVAVLGCTAVFQVIIIEFLGTFASTTPLTCLQWFVSVFIGFLGMPVAAALKTIPV

>GrACA12

MSGFSSGDGRLQDLEAGPSKDNNDLNTNLDPDADTSDPFDIDQTKNATPQTLKRWRQAALVLNASRRFRYTLDLRKQEEKEQRKRMIRAHAQVIRAALLFKLAGEKGIVSGTPVTLPGAAGDFAVGLERLALMTRDHKLSALQQYGGVKGLSDLLKTNLETGIYGDEVDLLNRKTAFGSNTYPRKKGRSFWRFLWEAWQDLTLIILIVAASVSLGLGIKTEGLKEGWYDGGSIFLAVLLVIVVTATSDYRQSLQFQNLNEEKRNIQLEVVRGGRIVKVSIYDVVVGDVVPLKIGDQVPADGVLVNGHSLAIDESSMTGESKIVYKDKNEPFLMSGCKVADGVGTMLVTGVGINTEWGLLMASISEDTGEETPLQVRLNGVATFIGIVGLSVAVSVLVVLLARYFTGHTEDPDGTKQFIKGRTNFDDAFNDVVKIFTIAVTIVVVAVPEGLPLAVTLTLAYSMRKMMADKALVRRLSACETMGSATTICSDKTGTLTLNEMTVVEAFVGKKKINPPSDSSQLPASVVSLLNEGVAQNSTGNVFVPKDGGNIEISGSPTEKAILSWAVKLGMKFDIIRSDSKILHVFPFNSEKKRGGVALQGADSEVRIHWKGAAEIVLTSCSGYIDSNGCLQSINEDKEFFKAAIDEMAVNSLRCVALAYRLCEKEKVPTDEEGFNGWILPEDNLVLLAIVGIKDPCRPGVKDAVKICMDAGVKVRMVTGDNIQTAKAIALECGILSSAEDATEPTIIEGRVFRELSDKEREQIAKKITVMGRSSPSDKLLLVQALRKGGDVVAVTGDGTNDAPALHEADIGLSMGIQGTEVAKESSDIIILDDNFASVVKVVRWGRSVYANIQKFIQFQLTVNVAALVINVVAAISSGDVPLNSVQLLWVNLIMDTLGALALATEPPTDNLMHRSPVGRREPLITNIMWRNLLLQASYQVTVLLVLNFMGLTILHLKDDHDREHAYDVKNTLIFNAFVMCQIFNEFNARKPEEINCFKGVTKNYLFMGIIGFTFVLQIIIIEFLGKFTKTVRLNWKLWLVSLGIGIISWPLAIVGKLIPVPKTPVSGYFTKAFRRCRTDRNA

>GrHMA1

METHSISLTNFSPLTRPLRPSRLRRLNSFHFKPLFFSPLSTRYKSLFLPLNSHTIRIRCVANHGHHHHHEHDLDHGHGHDHDHDHHHHHHHHHHHHGSGQLNGPQKAVIGFAKAIRWMDLANFLREHLHLCCCATALFIAAAAFPYLVPKPAVKPLQNSFLVLAFPLVGVSAALDAITDIAGGKVNIHVLMALAAFASVFMGNALEGGLLLAMFNLAHIAEEFFTSRSMIDVKELKENYPDSALVLNLDDDNLPNVSDLSYRSIPVHDVEVGSYILVTTGEAVPVDCEVFHGSATITIEHLTGEIKPLEAKAGDRIPGGARNLDGRMIVKVLKTWKESTLSRIVQLTEEAQLNKPKLQRWLDEFGEQYSKVVVVLSVAIAVLGPFLFKWPFISTAVCRGSIYRALGLMVAASPCALAVAPLAYATAVSSCARKGILLKGGQVLDALASCHTVAFDKTGTLTTGGLMFKAIEPIYGHIIGNKKTNFTSCCVPNCEVEALAVAAAMEKGTTHPIGRAVVDHSIGKDLPSVSVESFEYFPGKGLIATLNSAESGTRGGKMLKASLGSIEFITSLCKSEVKSRMIRAAVNASSYGTDFVHAALSVDEKVTLIHLEDRPRPGVLDVISELKDKAKVRVMMLTGDHKLSAWRVANAVGINEVYCSLKPEDKLNHVKRISGDMGGGLIMVGEGINDAPALAAATVGIVLAHRASATAIAVADVLLLQDNISGVPFSIAKARQTTSLVKQNVALALTCIILASLPSVLGFLPLWLTVLLHEGGTLLVCLNSIRALNDPSWSWGQDLRNLIGKLKSKLALLRHNATSSTIQTAPL

>GrACA11

MSSIFKGSPYRRPNDLEAGSSRSAHSDDEDHESFADPFDITSTKNAPIDRLRRWRQAALVLNASRRFRYTLDLKKEEEKKQILRKIRAHAQAIRAAYLFKQAGEQVNGTTTPHPTPGSDFAFGPEQLASVTRDHNFNALQEYGGVNGLAESLKTNLEKGIPGDDSDLLKRRNAFGSNTYPRKKGRSFWRFVWEACQDLTLMILVVAAVASLALGIKTEGPKEGWYDGGSIAFAVFLVIIVTAISDYRQSLQFQKLDEEKRNIHLEVVRGGRRVEISIYDIVVGDVVPLNIGDQVPADGILISGHSFAIDESSMTGESDIVQKDAKQPFLMSGCKVADGSGTMLVTGVGINTEWGLLMANLSEDTGEETPLQVRLNGVATFIGFVGLSVAFAVLVVLLVRYFTGHTEDSNGRQQFVAGKTSVGNAIDGAIKIVTVAVTIVVVAVPEGLPLAVTLTLAYSMKKMMADKALVRRLSACETMGSATTICSDKTGTLTLNQMTVVEAYAGGRKNDPPESRSELPDTLVSLLIEGIAVNANGSVFTSEGGGDVEVSGSPTEKAILIWGIKLGMDFDAVRSGSSIVHVFPFNSEKKRGGVAIRLPDSKVHIHWKGAAEIVLAACTWYLDTNGEAVAMDEEKVAFFEKAIETMAAGSLRCVAIAYRSYESEKVPTNEEELAKWALPEDDLVLLAIVGIKDPCRPSVKDSVQLCQKAGVKVRMVTGDNLKTARAIALECGILSSDAPESSLIEGKVFRSLSDSEREEVAEKISVMGRSSPNDKLLLVQALRRKGHVVAVTGDGTNDAPALHEADIGLAMGIQGTEVAKESSDIIILDDNFASVVKVVRWGRSVYANIQKFIQFQLTVNVAALVINVVAAVSSGDVPLNAVQLLWVNLIMDTLGALALATEPPTDHLMHRPPVGRREPLITNIMWRNLLIQAIYQVTVLLVLNFDGKKILNLEHESKEHANRVKNTLIFNAFVLSQIFNEFNARKPDEVNIFRGLSKNYLFIGIVAITIILQAIIVEFLGKFAKTEKLSWQLWLVSIGIGFISWPLATLGKFIPVPETPVSRVFSRMFYRRRNQSSQKHEDSNATSIRN

>GrACA8

MTTIFEPNLICSEHTIQDPPTPSTSEKAWHSIFASKKFSSLLTMSPTPKEEAKVVHRSPSHVSVNVMQENPPYQIDQTTLIELVKEKKLERLQEFGGVNGVASTFGTDTQVGISGGADDVARLRNTFGSNTYNKPPTKGFFHFVIEALKEPAVMILLGCAALSLGFKIKAHGHKDSWHEVGSNFAAVFLPIVVSAISNYMQNRQFEELSRANNNILVDVVRGGQRQQLPMFDVVVGDIVCLKMGDQVPADGLFLDGHSLQVDESSMTGATEHTEVNSSQNPFLLSWTKVAHGDARMLVTSVGMNTTWGQISRETNEQTPLQARLNKLSSSIAKVGLAVAFPVLVVLLVRYFTGHTKDEKGNREFNRSKTKTSDIINAIVGIATTAITTAAEGLPLVVTLTLAYSKKKMVADQAMVRKLSACETMASTTTICTAKTGTLTLNRMKVTKFWLEYELSGSPTEKAILSWAVVELKMDVEKTKKRCAALQVEAFIPQKRRSGVLIERNDDDTVHVHWKGAAEMILAMSSSYYDASGVVKDLDDGERMKFEEIIQGMAASSLRCIAFAHKQVPEEEYQNLKEQKKLKEDSLTLLGLVGIKDPCRPGVKKAVEDCQYAGVNIKMITGDNVFTAIAIATECGILKPVVEGEEFRNYTSQERMEKVEKIQVMAGFSPFDKLLMVQCLKQKGHVVAVTGDGTDDAPALKEADIGLSTGIQGTEAARESSDIVILDDSFASVATVLRWGRCVYTNIQKFIQFLLTANVATLCINFIAAVSTGEVPLTTVQLFWVNLYMDTLGALALATERPTKELLEKPPVGRTEPLITNIMWRNLLAQALYQIAVLLTLHFNGESIFGVTKKVNHTLIFNTFMLCQVFNKFNARKLEKKNVFNDMHKNKMFIGITGVTIVLQVVMVEFLKRFTDTERLNWGQWGACIAIAAASCPLGWAVKCLPVPKKPIFSYLKWNVAGLDQNS

>GrALA1

MKRYVYINDNESSHELYCDNRISNRKYTVLNFLPKNLWEQFSRFMNQYFLLIACLQLWSLITPVNPASTWGPLIFIFAVSASKEAWDDYNRYLSDKKANEKLVWVVRQGIRKHIQAQDIHVGNIVWLRENDEVPCDLVLIGTSDPQGLCYVETAALDGETDLKTRVIPSACMGIDFELLHKIKGVIECPNPDKDITRFDANLRLFPPFIDNDVCPLTIKNTILQSCYLRNTEWACGVAVYTGNETKLGMSRGIPEPKLTAMDAMIDKLTGAIFVFQIVVVMVLGIAGNVWKDTEARKQWYVQYPIEGPWYELLVIPLRFELLCSIMIPISIKVSLDLVKSLYAKFIDWDTEMIDYETGIPSHATNTAISEDLGQVEYIMTDKTGTLTENRMIFRRCCISGVFYGNESGDALKDTKLLNAVAGSSPDVVQFLTVMAICNTVVPIKSKTGAISYKAQSQDEDALVNAAAQLHMVYANKTANILEIRFNGSVIKYEVLEILEFTSDRKRMSVVVKDCQNGKIVLLSKGADEAILPYAYVGQQTRTFIEAVEQYAQLGLRTLCLACRELREDEYQEWSLLFKEASSTLVDREWRIAEVCQRLEHDFEVLGVTAIEDRLQDGVPETIETLRKAGINFWMLTGDKQNTAIQIALSCNFISPEPKGQLLLIDGKTEDEVCRSLERVLLTMRITSSEPKDVAFVVDGWALEIALKHYRKAFTELAILSRTAICCRVTPSQKAQLVELLKSCDYRTLAIGDGGNDVRMIQQADIGVGISGREGLQAARAADYSIGKFRFLKRLILVHGRYSYNRTAFLSQYSFYKSLVICFIQIFFSFISGVSGTSLFNSVSLMAYNVFYTSVPVLVSVLDKDLSEGTVMQHPQILFYCQAGRLLNPSTFAGWFGRSLFHAIVVFVITIHAYAYEKSEMEELSMVALSGCIWLQAFVVALETNSFTILQHLAIWGNLVAFYVINWIFSAIPASGMYTIMFRLCRQLSYWITMSLIVAAGMGPVLALKYFRYTYRPSKINTLQQAERMGGPILTLGNIEPHPRPMEKEVVSPLQISQPKNRNPVYEPLLSDSPNSSRRSLGSGTPFDFFQSQSRLSSSYSRNCKDN

>GrALA2
[truncated: 12,030 more chars]
