# Supplementary material for: Genomic Comparison of the P-ATPase Gene Family in Four Cotton Species and Their Expression Patterns in Gossypium hirsutum
Source: Molecules. 2018 May 5;23(5):1092. doi: 10.3390/molecules23051092 (PMC6102550; doi:10.3390/molecules23051092)
Supplement: Supplementary file 1 [file molecules-23-01092-s001.zip › Supplementary files/Table S1.docx]

|  |  | Chro- | | Protein | Isoelectric | Molecular | |
| --- | --- | --- | --- | --- | --- | --- | --- |
| Gene | Protein ID | | msome | Length  (aa) | Point | | Weight  (kDa) |
|  |  | |  |  |  | |  |
| *GrAHA1* | Gorai.009G068100.1 | | 9 | 956 | 6.8 | | 105.17 |
| *GrAHA2* | Gorai.010G123000.1 | | 10 | 957 | 6.8 | | 105.79 |
| *GrAHA3* | Gorai.013G212200.1 | | 13 | 954 | 7.38 | | 105.21 |
| *GrAHA4* | Gorai.009G004200.1 | | 9 | 825 | 8.81 | | 90.86 |
| *GrAHA5* | Gorai.004G027300.1 | | 4 | 951 | 6.93 | | 104.86 |
| *GrAHA6* | Gorai.011G089100.1 | | 11 | 953 | 6.47 | | 105.06 |
| *GrAHA7* | Gorai.010G136300.1 | | 10 | 949 | 7.24 | | 104.68 |
| *GrAHA8* | Gorai.009G125200.1 | | 9 | 953 | 6.06 | | 104.99 |
| *GrAHA9* | Gorai.009G053500.1 | | 9 | 952 | 5.49 | | 104.28 |
| *GrAHA10* | Gorai.013G217900.1 | | 13 | 899 | 4.85 | | 97.97 |
| *GrAHA11* | Gorai.008G232800.1 | | 8 | 966 | 7.33 | | 106.08 |
| *GrAHA12* | Gorai.006G023600.1 | | 6 | 956 | 6.64 | | 105.31 |
| *GrAHA13* | Gorai.003G094300.1 | | 3 | 987 | 6.63 | | 108.9 |
| *GrAHA14* | Gorai.011G053900.1 | | 11 | 980 | 5.35 | | 108.03 |
| *GrECA1* | Gorai.009G220900.1 | | 9 | 1033 | 5.19 | | 112.97 |
| *GrECA2* | Gorai.007G110700.1 | | 7 | 1050 | 5.67 | | 115.97 |
| *GrECA3* | Gorai.001G152900.1 | | 1 | 1050 | 5.26 | | 115.7 |
| *GrECA4* | Gorai.002G252900.1 | | 2 | 1061 | 5.24 | | 116.17 |
| *GrECA5* | Gorai.001G106800.1 | | 1 | 1061 | 5.26 | | 116.41 |
| *GrECA6* | Gorai.003G137900.1 | | 3 | 880 | 5.07 | | 96.45 |
| *GrHMA1* | Gorai.007G218200.1 | | 7 | 826 | 8.37 | | 89.28 |
| *GrHMA2* | Gorai.009G003000.1 | | 9 | 933 | 7.82 | | 100.77 |
| *GrHMA3* | Gorai.004G098400.1 | | 4 | 956 | 7.66 | | 103.84 |
| *GrHMA4* | Gorai.005G220300.1 | | 5 | 586 | 6.2 | | 62.06 |
| *GrHMA5* | Gorai.010G096400.1 | | 10 | 898 | 6.52 | | 95.71 |
| *GrHMA6* | Gorai.008G049700.1 | | 8 | 1011 | 4.82 | | 108.22 |
| *GrHMA7* | Gorai.009G072500.1 | | 9 | 995 | 6.27 | | 107.87 |
| *GrHMA8* | Gorai.004G210800.1 | | 4 | 988 | 6.19 | | 106.9 |
| *GrACA1* | Gorai.003G029900.1 | | 3 | 1048 | 5.66 | | 114.08 |
| *GrACA2* | Gorai.011G108000.1 | | 11 | 1014 | 6.19 | | 109.87 |
| *GrACA3* | Gorai.010G025500.1 | | 10 | 1020 | 6.46 | | 111.16 |
| *GrACA4* | Gorai.006G136000.1 | | 6 | 1032 | 5.58 | | 113.2 |
| *GrACA5* | Gorai.011G295500.1 | | 11 | 1041 | 6.98 | | 114.2 |
| *GrACA6* | Gorai.004G056600.1 | | 4 | 1047 | 6.51 | | 115.9 |
| *GrACA7* | Gorai.006G243500.1 | | 6 | 1019 | 7.44 | | 112.12 |
| *GrACA8* | Gorai.006G244700.1 | | 6 | 966 | 7.89 | | 106.56 |
| *GrACA9* | Gorai.007G277500.1 | | 7 | 1018 | 8.02 | | 112.23 |
| *GrACA10* | Gorai.007G277600.1 | | 7 | 1004 | 7.91 | | 110.46 |
| *GrACA11* | Gorai.004G024800.1 | | 4 | 1092 | 6.7 | | 119.25 |
| *GrACA12* | Gorai.009G118300.1 | | 9 | 1082 | 6.88 | | 118.22 |
| *GrACA13* | Gorai.001G221000.1 | | 1 | 923 | 5.92 | | 101.13 |
| *GrALA1* | Gorai.008G154700.1 | | 8 | 1106 | 6.407 | | 124.89 |
| *GrALA2* | Gorai.011G226200.1 | | 11 | 1187 | 6.952 | | 133.51 |
| *GrALA3* | Gorai.012G023200.1 | | 12 | 1182 | 6.691 | | 132.87 |
| *GrALA4* | Gorai.005G003800.1 | | 5 | 1189 | 6.851 | | 135.11 |
| *GrALA5* | Gorai.009G346300.1 | | 9 | 1203 | 7.627 | | 136.95 |
| *GrALA6* | Gorai.002G166300.1 | | 2 | 1227 | 8.167 | | 139.92 |
| *GrALA7* | Gorai.011G051000.1 | | 11 | 1236 | 5.508 | | 141.19 |
| *GrALA8* | Gorai.001G262700.1 | | 1 | 1139 | 6.831 | | 129.91 |
| *GrALA9* | Gorai.003G106100.1 | | 3 | 1195 | 5.588 | | 135.19 |
| *GrALA10* | Gorai.008G188500.1 | | 8 | 1187 | 5.866 | | 134.3 |
| *GrP5* | Gorai.013G132500.1 | | 13 | 1186 | 8.297 | | 131.73 |
| *GhAHA10* | CotAD_13369 | | At6 | 956 | 6.64 | | 105.26 |
| *GhAHA11* | CotAD_00185 | | Dt9 | 916 | 5.4 | | 100.36 |
| *GhAHA12* | CotAD_43378 | | Dt9 | 951 | 7.11 | | 104.71 |
| *GhAHA13* | CotAD_23393 | | Dt9 | 951 | 7.11 | | 104.6 |
| *GhAHA14* | CotAD_07237 | | sca | 951 | 6.83 | | 104.81 |
| *GhAHA15* | CotAD_06154 | | Dt13 | 1038 | 7.16 | | 114.8 |
| *GhAHA16* | CotAD_06914 | | At13 | 934 | 7.13 | | 102.67 |
| *GhAHA17* | CotAD_10988 | | At6 | 934 | 7.12 | | 102.7 |
| *GhAHA18* | CotAD_43487 | | sca | 954 | 6.8 | | 104.95 |
| *GhAHA19* | CotAD_00034 | | Dt9 | 954 | 6.8 | | 104.97 |
| *GhAHA20* | CotAD_48883 | | Dt10 | 957 | 6.83 | | 105.83 |
| *GhAHA21* | CotAD_58127 | | At10 | 958 | 6.83 | | 105.88 |
| *GhAHA22* | CotAD_68122 | | Dt8 | 954 | 7.51 | | 104.81 |
| *GhAHA23* | CotAD_34203 | | At5 | 893 | 8.14 | | 98.29 |
| *GhAHA24* | CotAD_70985 | | sca | 952 | 5.487 | | 104.26 |
| *GhAHA25* | CotAD_51484 | | Dt6 | 828 | 8.764 | | 91.24 |
| *GhAHA26* | CotAD_45144 | | At5 | 968 | 6.65 | | 106.736 |
| *GhAHA27* | CotAD_02056 | | Dt3 | 926 | 6.93 | | 102.16 |
| *GhAHA28* | CotAD_68334 | | At11 | 975 | 6.11 | | 107.272 |
| *GhAHA29* | CotAD_52482 | | Dt11 | 880 | 6.15 | | 96.422 |
| *GhAHA30* | CotAD_25690 | | Dt9 | 374 | 6.52 | | 41.48 |
| *GhAHA31* | CotAD_58663 | | At11 | 331 | 7.02 | | 36.76 |
| *GhECA1* | CotAD_07584 | | At9 | 1004 | 5.2 | | 110.14 |
| *GhECA2* | CotAD_36012 | | Dt9 | 1080 | 5.81 | | 118.79 |
| *GhECA3* | CotAD_54647 | | Dt1 | 882 | 5.97 | | 96.63 |
| *GhECA4* | CotAD_42632 | | Dt1 | 882 | 5.74 | | 96.61 |
| *GhECA5* | CotAD_57400 | | At7 | 819 | 5.69 | | 90.44 |
| *GhECA6* | CotAD_16975 | | At7 | 787 | 5.56 | | 86.88 |
| *GhECA7* | CotAD_49932 | | Dt1 | 960 | 5.14 | | 105.23 |
| *GhECA8* | CotAD_65826 | | sca | 972 | 4.97 | | 106.6 |
| *GhECA9* | CotAD_29550 | | Dt3 | 1061 | 5.21 | | 116.57 |
| *GhECA10* | CotAD_60181 | | At5 | 1031 | 5.31 | | 113.28 |
| *GhECA11* | CotAD_18316 | | Dt9 | 1061 | 5.22 | | 116.2 |
| *GhECA12* | CotAD_03446 | | At9 | 1061 | 5.25 | | 116.26 |
| *GhACA1* | CotAD_50427 | | Dt1 | 909 | 7.12 | | 99.9 |
| *GhACA2* | CotAD_47945 | | Dt1 | 828 | 6.51 | | 91.58 |
| *GhACA3* | CotAD_19392 | | Dt11 | 1014 | 6.19 | | 109.88 |
| *GhACA4* | CotAD_69371 | | sca | 563 | 6.41 | | 61.79 |
| *GhACA5* | CotAD_33582 | | sca | 1015 | 5.37 | | 110.17 |
| *GhACA6* | CotAD_76165 | | sca | 711 | 5.11 | | 76.52 |
| *GhACA7* | CotAD_25589 | | sca | 999 | 6.36 | | 108.64 |
| *GhACA8* | CotAD_43422 | | sca | 1002 | 6.12 | | 109.36 |
| *GhACA9* | CotAD_20564 | | Dt6 | 1034 | 5.48 | | 113.55 |
| *GhACA10* | CotAD_43310 | | At6 | 1034 | 5.71 | | 113.51 |
| *GhACA11* | CotAD_71374 | | At9 | 713 | 5.8 | | 77.63 |
| *GhACA12* | CotAD_01556 | | Dt9 | 1161 | 6.94 | | 126.96 |
| *GhACA13* | CotAD_56251 | | At11 | 1109 | 5.82 | | 121.18 |
| *GhACA14* | CotAD_15582 | | Dt11 | 1056 | 6.31 | | 115.66 |
| *GhACA15* | CotAD_07218 | | sca | 1124 | 8.16 | | 122.99 |
| *GhACA16* | CotAD_12200 | | At1 | 1018 | 7.93 | | 112.18 |
| *GhACA17* | CotAD_12201 | | At1 | 979 | 8.4 | | 107.89 |
| *GhACA18* | CotAD_72332 | | At4 | 1077 | 6.7 | | 118.7 |
| *GhACA19* | CotAD_40831 | | Dt4 | 940 | 6.13 | | 103.83 |
| *GhACA20* | CotAD_10596 | | sca | 1019 | 7.45 | | 112.1 |
| *GhACA21* | CotAD_10245 | | sca | 1019 | 7.31 | | 112.04 |
| *GhACA22* | CotAD_10233 | | sca | 730 | 5.59 | | 80.05 |
| *GhACA23* | CotAD_10608 | | sca | 1028 | 8 | | 113.13 |
| *GhHMA1* | CotAD_19592 | | At1 | 698 | 7.34 | | 74.26 |
| *GhHMA2* | CotAD_48170 | | Dt1 | 1004 | 7.91 | | 109.03 |
| *GhHMA3* | CotAD_74424 | | At4 | 921 | 7.47 | | 100.07 |
| *GhHMA4* | CotAD_23379 | | Dt9 | 933 | 7.82 | | 100.69 |
| *GhHMA5* | CotAD_37715 | | Dt5 | 950 | 8.17 | | 100.64 |
| *GhHMA6* | CotAD_55074 | | At5 | 952 | 8.24 | | 100.8 |
| *GhHMA7* | CotAD_39986 | | At8 | 482 | 5.47 | | 51.07 |
| *GhHMA8* | CotAD_14206 | | Dt8 | 1020 | 4.85 | | 109.19 |
| *GhHMA9* | CotAD_12489 | | At9 | 993 | 6.42 | | 107.79 |
| *GhHMA10* | CotAD_72129 | | sca | 420 | 4.77 | | 44.9 |
| *GhHMA11* | CotAD_25454 | | sca | 443 | 5.15 | | 47.53 |
| *GhHMA12* | CotAD_51877 | | sca | 988 | 6.41 | | 106.93 |
| *GhHMA13* | CotAD_51879 | | sca | 632 | 7.87 | | 68.52 |
| *GhALA1* | CotAD_12771.1 | | Dt8 | 995 | 5.276 | | 111.45 |
| *GhALA2* | CotAD_53650.1 | | sca | 950 | 5.442 | | 106.35 |
| *GhALA3* | CotAD_21325.1 | | At11 | 1089 | 6.367 | | 122.4 |
| *GhALA4* | CotAD_26822.1 | | Dt11 | 1126 | 6.734 | | 126.41 |
| *GhALA5* | CotAD_27250.1 | | sca | 1071 | 6.366 | | 120.42 |
| *GhALA6* | CotAD_14616.1 | | At1 | 1266 | 6.331 | | 143.75 |
| *GhALA7* | CotAD_33704.1 | | sca | 1151 | 6.831 | | 130.64 |
| *GhALA8* | CotAD_57727.1 | | At9 | 1203 | 7.829 | | 137.01 |
| *GhALA9* | CotAD_56216.1 | | At5 | 1203 | 7.829 | | 136.97 |
| *GhALA10* | CotAD_42255.1 | | At6 | 1209 | 6.968 | | 137.34 |
| *GhALA11* | CotAD_07952.1 | | sca | 1139 | 6.648 | | 129.99 |
| *GhALA12* | CotAD_01909.1 | | Dt3 | 653 | 6.45 | | 74.33 |
| *GhALA13* | CotAD_65530.1 | | At11 | 1237 | 5.749 | | 139.46 |
| *GhALA14* | CotAD_31046.1 | | Dt1 | 1195 | 5.735 | | 135.18 |
| *GhALA15* | CotAD_17891.1 | | sca | 1195 | 5.327 | | 134.95 |
| *GhALA16* | CotAD_22000.1 | | Dt8 | 1187 | 5.866 | | 134.34 |
| *GhALA17* | CotAD_57644.1 | | sca | 1187 | 5.785 | | 134.28 |
| *GhP5-1* | CotAD_62923.1 | | At3 | 1189 | 8.33 | | 132.04 |
| *GhP5-2* | CotAD_17989.1 | | Dt3 | 1189 | 8.159 | | 132.11 |
| *GaECA5* | Cotton_A_02222 | | 13 | 1061 | 5.32 | | 116.65 |
| *GaECA6* | Cotton_A_24664 | | 1 | 1068 | 5.12 | | 117.34 |
| *GaHMA1* | Cotton_A_32785 | | 4 | 815 | 7.87 | | 87.73 |
| *GaHMA2* | Cotton_A_24167 | | 5 | 958 | 7.85 | | 101.29 |
| *GaHMA3* | Cotton_A_39885 | | 10 | 889 | 6.63 | | 94.63 |
| *GaHMA4* | Cotton_A_38943 | | 12 | 958 | 7.54 | | 104 |
| *GaHMA5* | Cotton_A_17683 | | 6 | 1011 | 4.75 | | 108.03 |
| *GaHMA6* | Cotton_A_01072 | | 4 | 987 | 5.97 | | 106.99 |
| *GaHMA7* | Cotton_A_25987 | | 3 | 988 | 6.31 | | 106.91 |
| *GaACA1* | Cotton_A_20877 | | 3 | 819 | 8.05 | | 90.43 |
| *GaACA2* | Cotton_A_01905 | | 6 | 1034 | 5.59 | | 113.49 |
| *GaACA3* | Cotton_A_10107 | | 2 | 1041 | 7.25 | | 114.26 |
| *GaACA4* | Cotton_A_09766 | | 11 | 1015 | 5.55 | | 110 |
| *GaACA5* | Cotton_A_26619 | | 3 | 1108 | 8.07 | | 121.15 |
| *GaACA6* | Cotton_A_31403 | | 9 | 1109 | 5.64 | | 121.22 |
| *GaACA7* | Cotton_A_15074 | | 10 | 957 | 7.17 | | 104.4 |
| *GaACA8* | Cotton_A_09309 | | 4 | 1018 | 7.93 | | 112.18 |
| *GaACA9* | Cotton_A_09312 | | 4 | 1004 | 8.35 | | 110.52 |
| *GaACA10* | Cotton_A_16483 | | 10 | 1019 | 7.29 | | 112.02 |
| *GaACA11* | Cotton_A_16470 | | 10 | 1004 | 7.85 | | 110.7 |
| *GaACA12* | Cotton_A_03218 | | 3 | 1041 | 6.35 | | 114.93 |
| *GaACA13* | Cotton_A_25993 | | 3 | 1067 | 6.47 | | 117.6 |
| *GaACA14* | Cotton_A_25991 | | 3 | 1063 | 6.58 | | 117.15 |
| *GaALA1* | Cotton_A_25036 | | 11 | 1176 | 8.24 | | 130.57 |
| *GaALA1* | Cotton_A_04473.1 | | 6 | 1108 | 6.457 | | 125.09 |
| *GaALA2* | Cotton_A_20278.1 | | 12 | 1182 | 6.778 | | 132.84 |
| *GaALA3* | Cotton_A_24709.1 | | 9 | 1187 | 6.64 | | 133.55 |
| *GaALA4* | Cotton_A_04315.1 | | 5 | 1025 | 6.637 | | 115.81 |
| *GaALA5* | Cotton_A_37085.1 | | 8 | 1339 | 7.876 | | 153.64 |
| *GaALA6* | Cotton_A_33194.1 | | 10 | 1127 | 6.848 | | 128.13 |
| *GaALA7* | Cotton_A_25371.1 | | 11 | 1209 | 6.87 | | 137.33 |
| *GaALA8* | Cotton_A_11524.1 | | 1 | 1142 | 7.212 | | 130.42 |
| *GaALA9* | Cotton_A_23434.1 | | 12 | 1189 | 7.469 | | 135.09 |
| *GaALA10* | Cotton_A_18600.1 | | 1 | 1195 | 5.51 | | 135.11 |
| *GaALA11* | Cotton_A_25785.1 | | 6 | 1187 | 5.703 | | 134.42 |
| *GaP5* | Cotton_A_25036.1 | | 11 | 1176 | 8.239 | | 130.56 |
| *GbAHA12* | Gbscaffold9694.3.0 | | sca | 899 | 5.06 | | 97.91 |
| *GbAHA13* | Gbscaffold6881.8.0 | | sca | 508 | 5.03 | | 55.09 |
| *GbAHA14* | Gbscaffold8903.14.1 | | sca | 980 | 5.46 | | 108.07 |
| *GbAHA15* | Gbscaffold8903.14.0 | | sca | 952 | 5.47 | | 104.92 |
| *GbAHA16* | Gbscaffold7145.1.0 | | sca | 956 | 6.24 | | 105.34 |
| *GbAHA17* | Gbscaffold17456.1.0 | | sca | 956 | 6.24 | | 105.31 |
| *GbAHA18* | Gbscaffold3841.1.2 | | sca | 956 | 6.23 | | 105.25 |
| *GbAHA19* | Gbscaffold3035.2.2 | | sca | 956 | 6.3 | | 105.19 |
| *GbAHA20* | Gbscaffold39450.1.0 | | sca | 1006 | 6.24 | | 110.55 |
| *GbAHA21* | Gbscaffold3628.4.0 | | sca | 896 | 8.29 | | 97.87 |
| *GbAHA22* | Gbscaffold8492.3.0 | | sca | 1255 | 6.64 | | 140.18 |
| *GbAHA23* | Gbscaffold2214.20.0 | | sca | 1646 | 6.45 | | 183.48 |
| *GbECA1* | Gbscaffold4556.29.0 | | sca | 1001 | 5.53 | | 109.65 |
| *GbECA2* | Gbscaffold13763.19.0 | | sca | 1001 | 5.53 | | 109.62 |
| *GbECA3* | Gbscaffold4556.29.1 | | sca | 971 | 5.76 | | 106.37 |
| *GbECA4* | Gbscaffold3618.5.0 | | sca | 1050 | 5.39 | | 115.6 |
| *GbECA5* | Gbscaffold30689.1.0 | | sca | 1050 | 5.39 | | 115.66 |
| *GbECA6* | Gbscaffold9789.11.0 | | sca | 1015 | 5.65 | | 112.15 |
| *GbECA7* | Gbscaffold2981.1.0 | | sca | 1002 | 5.46 | | 110.73 |
| *GbECA8* | Gbscaffold4553.12.0 | | sca | 1061 | 5.34 | | 116.48 |
| *GbECA9* | Gbscaffold9697.2.0 | | sca | 798 | 5.32 | | 87.84 |
| *GbECA10* | Gbscaffold24414.3.1 | | sca | 1061 | 5.43 | | 116.22 |
| *GbECA11* | Gbscaffold24414.3.0 | | sca | 1085 | 5.3 | | 118.64 |
| *GbECA12* | Gbscaffold15606.40.0 | | sca | 1446 | 5.32 | | 158.41 |
| *GbHMA1* | Gbscaffold6239.2.0 | | sca | 958 | 7.23 | | 104.03 |
| *GbHMA2* | Gbscaffold1088.7.0 | | sca | 956 | 8.05 | | 103.77 |
| *GbHMA3* | Gbscaffold15300.4.0 | | sca | 933 | 8.12 | | 100.85 |
| *GbHMA4* | Gbscaffold3761.11.0 | | sca | 933 | 8.37 | | 100.85 |
| *GbHMA5* | Gbscaffold11661.1.0 | | sca | 822 | 8.46 | | 88.79 |
| *GbHMA6* | Gbscaffold17001.1.0 | | sca | 885 | 5.97 | | 94.41 |
| *GbHMA7* | Gbscaffold17001.1.1 | | sca | 898 | 5.97 | | 95.68 |
| *GbHMA8* | Gbscaffold9346.8.0 | | sca | 898 | 5.97 | | 95.71 |
| *GbHMA9* | Gbscaffold9346.8.1 | | sca | 838 | 5.93 | | 89.57 |
| *GbHMA10* | Gbscaffold13132.6.0 | | sca | 1199 | 6.33 | | 127.34 |
| *GbHMA11* | Gbscaffold10194.12.0 | | sca | 1098 | 7.01 | | 115.17 |
| *GbHMA12* | Gbscaffold14361.3.0 | | sca | 1011 | 5.08 | | 108.18 |
| *GbHMA13* | Gbscaffold14361.3.1 | | sca | 869 | 5.1 | | 93.28 |
| *GbHMA14* | Gbscaffold30041.2.0 | | sca | 1011 | 5.05 | | 108.13 |
| *GbHMA15* | Gbscaffold14361.3.2 | | sca | 807 | 5.75 | | 86.29 |
| *GbHMA16* | Gbscaffold34293.2.0 | | sca | 552 | 6.65 | | 58.82 |
| *GbHMA17* | Gbscaffold8747.2.0 | | sca | 843 | 5.83 | | 90.26 |
| *GbHMA18* | Gbscaffold2628.6.0 | | sca | 889 | 5.55 | | 96.15 |
| *GbHMA19* | Gbscaffold2628.4.0 | | sca | 988 | 5.86 | | 106.9 |
| *GbHMA20* | Gbscaffold2628.10.0 | | sca | 916 | 5.7 | | 99.65 |
| *GbACA1* | Gbscaffold20356.5.0 | | sca | 1568 | 6.85 | | 172.21 |
| *GbACA2* | Gbscaffold1153.13.0 | | sca | 1214 | 5.56 | | 133.63 |
| *GbACA3* | Gbscaffold1153.12.0 | | sca | 905 | 5.8 | | 99.63 |
| *GbACA4* | Gbscaffold18188.5.0 | | sca | 1015 | 5.56 | | 110.1 |
| *GbACA5* | Gbscaffold12200.4.0 | | sca | 1015 | 5.61 | | 109.9 |
| *GbACA6* | Gbscaffold3399.1.0 | | sca | 1516 | 5.89 | | 163.53 |
| *GbACA7* | Gbscaffold4005.16.1 | | sca | 844 | 5.58 | | 91.05 |
| *GbACA8* | Gbscaffold4005.16.3 | | sca | 844 | 5.58 | | 90.98 |
| *GbACA9* | Gbscaffold3896.21.1 | | sca | 844 | 5.52 | | 90.98 |
| *GbACA10* | Gbscaffold4005.16.0 | | sca | 1020 | 6.03 | | 111.18 |
| *GbACA11* | Gbscaffold3896.21.2 | | sca | 1020 | 6.11 | | 111.1 |
| *GbACA12* | Gbscaffold12484.6.0 | | sca | 1064 | 6.56 | | 116.89 |
| *GbACA13* | Gbscaffold22979.5.0 | | sca | 841 | 6.06 | | 91.67 |
| *GbACA14* | Gbscaffold15225.1.0 | | sca | 830 | 5.36 | | 91.43 |
| *GbACA15* | Gbscaffold10295.26.0 | | sca | 1034 | 5.54 | | 113.52 |
| *GbACA16* | Gbscaffold15272.6.0 | | sca | 1191 | 8.79 | | 130.37 |
| *GbACA17* | Gbscaffold12432.13.0 | | sca | 1124 | 8.19 | | 122.37 |
| *GbACA18* | Gbscaffold51968.1.1 | | sca | 437 | 5.42 | | 47.6 |
| *GbACA19* | Gbscaffold28144.4.1 | | sca | 1217 | 7.85 | | 133.51 |
| *GbACA20* | Gbscaffold12872.7.1 | | sca | 1298 | 6.56 | | 142.13 |
| *GbACA21* | Gbscaffold7001.1.0 | | sca | 1037 | 8 | | 114.34 |
| *GbACA22* | Gbscaffold11313.11.0 | | sca | 1018 | 8.01 | | 112.03 |
| *GbACA23* | Gbscaffold15457.1.0 | | sca | 1004 | 8.49 | | 110.63 |
| *GbACA24* | Gbscaffold11313.12.0 | | sca | 1031 | 8.52 | | 113.91 |
| *GbACA25* | Gbscaffold16813.9.0 | | sca | 929 | 7.54 | | 101.87 |
| *GbACA26* | Gbscaffold5564.2.0 | | sca | 680 | 7.15 | | 74.72 |
| *GbACA27* | Gbscaffold7585.9.0 | | sca | 1155 | 5.93 | | 126.65 |
| *GbACA28* | Gbscaffold2659.3.0 | | sca | 1055 | 6.32 | | 116.29 |
| *GbACA29* | Gbscaffold6455.3.0 | | sca | 1108 | 5.96 | | 121.39 |
| *GbACA30* | Gbscaffold18051.1.0 | | sca | 702 | 5.5 | | 76.94 |
| *GbACA31* | Gbscaffold987.20.0 | | sca | 1039 | 6.35 | | 114.92 |
| *GbACA32* | Gbscaffold18051.2.0 | | sca | 1024 | 6.19 | | 113.46 |
| *GbACA33* | Gbscaffold987.21.0 | | sca | 996 | 6.22 | | 110.26 |
| *GbALA1* | Gbscaffold41642.2.0 | | sca | 1181 | 6.21 | | 132.65 |
| *GbALA2* | Gbscaffold136.1.0 | | sca | 1215 | 7.85 | | 138.57 |
| *GbALA3* | Gbscaffold2759.13.0 | | sca | 1202 | 8.38 | | 136.92 |
| *GbALA4* | Gbscaffold12601.33.0 | | sca | 1139 | 6.22 | | 129.89 |
| *GbALA5* | Gbscaffold8554.2.0 | | sca | 1142 | 6.87 | | 130.52 |
| *GbALA6* | Gbscaffold2256.14.0 | | sca | 1840 | 5.23 | | 208.07 |
| *GbALA7* | Gbscaffold959.23.0 | | sca | 1195 | 5.56 | | 134.98 |
| *GbALA8* | Gbscaffold21539.4.0 | | sca | 1438 | 5.7 | | 163 |
| *GbALA9* | Gbscaffold9700.12.0 | | sca | 1187 | 5.67 | | 134.34 |
| *GbP5-1* | Gbscaffold6055.1.0 | | sca | 1251 | 8.59 | | 139.06 |
| *GbP5-2* | Gbscaffold961.5.0 | | sca | 1186 | 8.51 | | 131.79 |

**Table S1** Characteristics of 250 *P-ATPase* genes in *G. raimondii*, *G. hirsutum*, *G. arboreum* and *G. barbadense.* The “At” and “Dt” represent Group A and Group D chromosomes in *G. hirsutum*, respectively. The “sca” indicates that there is no information on chromosomal location for the gene.
