# Supplementary material for: Genomic Comparison of the P-ATPase Gene Family in Four Cotton Species and Their Expression Patterns in Gossypium hirsutum
Source: Molecules. 2018 May 5;23(5):1092. doi: 10.3390/molecules23051092 (PMC6102550; doi:10.3390/molecules23051092)
Supplement: Supplementary file 1 [file molecules-23-01092-s001.zip › Supplementary files/Table S2.docx]

|  | P_1B_-ATPase | P_2A_-ATPase | P_2B_-ATPase | P_3A_-ATPase | P_4_-ATPase | P_5_-ATPase |
| --- | --- | --- | --- | --- | --- | --- |
| Arabidopsis | 8 | 4 | 10 | 11 | 12 | 1 |
| *G. raimondii* | 8 | 6 | 12 | 14 | 10 | 1 |
| *G. arboretum* | 7 | 6 | 14 | 14 | 11 | 1 |
| *G. barbadense* | 20 | 12 | 33 | 23 | 9 | 2 |
| *G. hirsutum* | 13 | 12 | 23 | 31 | 17 | 2 |

**Table S2**The gene numbers of 6 P-ATPase subfamilies in Arabidopsis, *G. raimondii*, *G. hirsutum*, *G. arboreum* and *G. barbadense.*
