# Supplementary material for: Genomic Comparison of the P-ATPase Gene Family in Four Cotton Species and Their Expression Patterns in Gossypium hirsutum
Source: Molecules. 2018 May 5;23(5):1092. doi: 10.3390/molecules23051092 (PMC6102550; doi:10.3390/molecules23051092)
Supplement: Supplementary file 1 [file molecules-23-01092-s001.zip › Supplementary files/Table S5.docx]

| \| Gene name \| Forward primer (5'–3') \| Reverse primer (5'–3') \| \| --- \| --- \| --- \| |
| --- | --- | --- | --- |
|  |
| \| *GhAHA1* \| TATGTGGAATCCTCTGTCA \| AATGAAGTCTTGCCAGTC \| \| --- \| --- \| --- \| \| *GhAHA2* \| GAGATTGCAGCTATTATG \| CACAATACCAATGAAGTC \| \| *GhAHA3* \| TGGCGGATAAGAGCATCA \| GGCTGAATACGAGGGAAAG \| \| *GhAHA4* \| GAGACTGTTGATCTTGAG \| TTGTTTTCTTTTGCCTTTT \| \| *GhAHA5* \| TATCAACTCCGCTATCAG \| CATCTCTTAGGACCTTGG \| \| *GhAHA6* \| CTTATTATCAACTCCACTATCAG \| CAAGACCAGCCATAAGTG \| \| *GhAHA7* \| GATTTCATTGGTATCGTTACTTTG \| CAGCCATTAAAGCAGCAG \| \| *GhAHA8* \| GGTATCGTGCTAAGAGAT \| TCTCCCAATTTGACACTA \| \| *GhAHA9* \| AGAACGAGACTGTTGATC \| CTCTCCTTCTTCTGATGTC \| \| *GhAHA10* \| TTTGGATGCTGTGCTTAA \| TCTGTTACACCTCAAAGTTT \| \| *GhAHA11* \| CTACTTGTATTGCGGTGTA \| TAGCGGATGATGAACTTG \| \| *GhAHA12* \| ATGGGTGCTATCAGTCTT \| ACTCAATCCTTCTCTGCTA \| \| *GhAHA13* \| GAGATCAAGAACGAGACTGT \| CTCCTCCGAACTCAATCC \| \| *GhAHA14* \| GGTATTAGTCTTGAAGAGA \| AAATCTGAATCCTGTTGA \| \| *GhAHA15* \| CTTCTAATCTCTTCAATGAC \| CCAAGTATGTGAGTGTAT \| \| *GhAHA16* \| GTAACAATCGCCTTCAAG \| TGACAAAGGATTCCACAT \| \| *GhAHA17* \| CAAGTGCTTACTGCCATT \| TTGTCAATCCCATCCCTAT \| \| *GhAHA18* \| GTTAGCATAGTGAGTCAG \| AAGCAGTGAGTAGAAGAA \| \| *GhAHA19* \| CAAGTTAGCATAGTGAGT \| CAGTGAGTAGAAGAAGTC \| \| *GhAHA20* \| AGGCATAGTATTGATGGTGAGAG \| CTGCGAGACCTTGTGACA \| \| *GhAHA21* \| CTAGTCAGTGCCTTTCTA \| AACCCATACCTTTAATCCT \| \| *GhAHA22* \| ACTGCTGCTTATCAACTC \| CATCTTCCTCGTTCCATT \| \| *GhAHA23* \| TGATGAAGTCCAGCAGAG \| AGAGCGATAGCCATAACAG \| \| *GhAHA24* \| TCATATTCGTGACAAGGT \| AATACAAGTAGCCACCAA \| \| *GhAHA25* \| ATTCAACAAATCAACAAGGTCACT \| CGACTGCGATGGAACATATAC \| \| *GhAHA26* \| TGATGCCCGATTACTTGA \| TTAGTTACAGGAAGGGACTC \| \| *GhAHA27* \| CAATAAGACAGACATTGAGAG \| CACTGCAAGAGATCGTAA \| \| *GhAHA28* \| TCAGGTGATATACCAGTC \| TAAGCGTTCATTAAACTCA \| \| *GhAHA29* \| CCTTAATGATGGAACTAT \| AATCAGTATCAATTACAATC \| \| *GhAHA30* \| CAAGCGGCAACGATATTC \| GCAGTAGAAAGAACTTCAACA \| \| *GhAHA31* \| ATGATATTCGGACTTACA \| CCATAACCCATGATAATG \|   *GhUBQ7*  GAAGGCATTCCACCTGACCAAC CTTGACCTTCTTCTTCTTGTGCTTG |

**Table S5** Primers used in qRT-PCR
