# Supplementary material for: Genomic Comparison of the P-ATPase Gene Family in Four Cotton Species and Their Expression Patterns in Gossypium hirsutum
Source: Molecules. 2018 May 5;23(5):1092. doi: 10.3390/molecules23051092 (PMC6102550; doi:10.3390/molecules23051092)
Supplement: Supplementary file 1 [file molecules-23-01092-s001.zip › Supplementary files/Response to Referees.docx]

Dear Editors,

Thank you for give me the opportunity to revise my article entitled “Genomic Comparison of the P-ATPase Gene Family in Four Cotton Species and their Expression Patterns in *Gossypium hirsutum*” (molecules-285009). We greatly appreciate the concerns and suggestions provided by editors and two reviewers, and have tried to make our manuscript more clearly by careful correction. We hope that the revised manuscript now is suitable for the journal. Revised portion are marked in red in the “Revised Manuscript with Track Changes”.

Thank you very much for your consideration. In the case of any questions, please feel free to contact with me.

Sincerely yours

Junshan Gao

Reviewers' Comments to Author:

Reviewer: 1

1.In plants, there are three proton pumps, the vacuolar-type ATPase, the vacuolar proton translocating pyrophosphatase and the P-type proton pumps. Enzymes of the F-type are considered as ATP synthases, located at the inner mitochondrial membrane or the thylakoids. The introduction should be revised accordingly.

**Reply:** According to the reviewer’s suggestion, we have modified the introduction in the revised manuscript accordingly. As follows: The representative H^+^-ATPases are currently divided into three types according to their structure, namely the vacuolar-type H^+^-ATPases (V-ATPases), H^+^-pyrophosphatases (H^+^-PPases) and P type H^+^-ATPases (P-ATPases). The H^+^-PPases is located in vacuolar or endomembranes.(lines:27-29)

2.By the way, not all P-type pumps have protons as substrate. It’s confusing that all were analyzed in silico while only proton pumps were addressed by the experiments and mentioned throughout the discussion section, focusing on proton pumps in the entire manuscript might help.

**Reply:** Thanks for the reviewer’s reminding. We have made corresponding changes in the revised version, As follows: As a P-type pump, P-ATPase can generate and maintain the electrochemical gradient of H^+^ on both sides of the cell membrane by decomposing intracellular……To date, 10 subfamilies of P-ATPase have been identified according to their transport of various ions, heavy metals and possibly lipids.( (lines: 30,37)

3.The proanthocyanidin transport is mediated by the antiporter TT12 in Arabidopsis seeds. The P3A-type ATPase creates the required proton motif force at the tonoplast of a defined cell layer. In other cells V-ATPase and V-PPase take over, and P-type proton pumps localize to the plasma membrane. The expression pattern is of less interest here, the subcellular localization matters. The conclusions are therefore highly speculative and not justified from my point of view.

**Reply:** We knew that the N-terminal motif of AtAHA10 homologues with conserved amino acids (EDLDKPLL) is critical for localizing in the vacuolar membrane, which is described in the paper entitled “Evolution of tonoplast P-ATPase transporters involved in vacuolar acidification” , so we looked at the N-terminal sequenced of P3A-ATPases in upland cotton, but it is regrettable that the similar amino acids motif didn’t been found in the P3A-ATPase genes. We performed subcellular localization prediction of P3A gene using WoLF PSORT online software, all genes were predicted to be located on the plasma membrane.

4. References for the expression pattern of P-type pumps are from 1994 to 2003, since then a vast amount of array data and RNAseq-data were collected and deposited in databases, why was all this information ignored? Some references are missing, for instance for the statement that some members of the P-type ATPase family are missing in Arabidopsis (lines 35-39). I am also puzzled by the statement that a difference of 300 amino acids is “little”.

**Reply:** Many thanks for this comment, we have modified this in the paper accordingly. We re-cited references for the P-type pump expression model from 1994 to 2016 (line 210-235), and the missing references have been re-cited (line 44). The statement that a difference of 300 amino acids is “little” had been changed as follow: this result suggested that the lengths of all P-ATPase proteins differed large, ranging from 800 to 1301aa (lines 74,7 5).

5.The figures need improvement. Why is the fourth dataset not displayed in figure 1? Figure 2 is too small, colored dots are not explained. The color does not correspond to different subfamilies as in the outer rim. Figure 3 is too small, even with higher magnification it is impossible to read the text. Figure 4 might be split in two parts, separating duplication within species from duplication between species. Figure 6: labeling of the axis needs improvement, at least “procyanidians” should be “proanthocyanidins” and there should be a spacing between “content” and the following bracket. It is not clear why data is given for soluble and insoluble PAs, neither the main text nor the legend refer to the difference at all.

**Reply:** Many thanks for this suggestion, because there is no chromosome assembly information in the *G. barbadense* genome database, we cannot get the position of the genes on the chromosome. In Figure 2, the green, orange, blue, red and purple dots indicate Arabidopsis, *G. arboretum*, *G. raimondii*, *G. barbadense* and *G. hirsutum*, respectively, we added it to the legend (lines 119,120). According to the reviewers’ recommendation, we have modified Figure 4 to split in two parts, and the error in Figure 6 has been modified. The original image of Figure 2 and Figure 3 will been provided in the supplementary materials for reviewing. There are some differences in the extraction methods of soluble PAs and insoluble PAs, their difference has no effect on this study, so we ignored it. In different plants, the PAs metabolic pathway may be somewhat different. In brown cotton, is there a P-type ATPase responsible for the accumulation of PAs? and if so, how does it work? How does it differ from TT13 function in Arabidopsis thaliana? This may be the focus of our next work.

6. Figure legends do not contribute much to the understanding of the figures, what is shown in figure 6 and 7, mean and SD? Mean and SE? What’s the number of experiments? Even the methods section is not of help. There is no further information about the number of experiments for the measurement of the PA-content. In case of the PCR, it is stated that three replicates were executed for higher accuracy of experimental results. That would mean that three technical replicates were performed, but no biological replicates. Without further information the experimental data is preliminary and unconfirmed. I also miss additional reference genes.

**Reply:** Many thanks for this comment. We performed 3 biological replicates for PAs measurements and PCR, and we have modified clearly the methods in the Materials and Methods (lines 342,342,349,350). The shown in figure 6 and 7 is mean and SE, we reinterpreted it in the legend. We also added reference genes to the method, as follows: The UBQ7 gene was used as an internal reference gene[41] (line 342).

7.All data is given by number of genes without explaining and considering the function of the proteins, there is not much information provided presenting the data this way. The discussion simply lists what is published about other species with almost no linkage to the obtained data.

**Reply:** Many thanks for this comment. Following the reviewer’s comment, we made corresponding changes in the discussion section. (lines 210-245)

Reviewer: 2

Comments to the Author(s)

1.In the present work a phylogenetic analysis of the P-Type ATPase family in cotton is performed. The authors also uses RNASeq data from the Cotton functional genomics database to assign a function to the respective genes. Finally the have an hypothesis that an AtAHA10 homologue must be responsible for the brown cotton fiber due to accumulation of PAs.

In my opinion this study does not represent a throughly investigation of ATPases in cotton. No members of the P5 family is identified, if this is the case this is the forst plant without a P5 pump. Also only 1 member of the P4 family is found, this is surprisingly low compared to the 12 members found in Arabidopsis (Table S2).P-type ATPases only have a few motifs in common and can therefore be difficult to identify, I will suggest a second search for P4 and P5ATPases.

**Reply:** Many thanks for this suggestion. According to the reviewer’s suggestion, We researched *P4* and *P5* ATPases in the genome database, and found indeed 10, 11, 9, 17 *P4* ATPases and 1, 1, 2, 2 *P5* ATPases in G. raimondii, G. arboretum, G. barbadense and G. hirsutum respectively. We have made corresponding changes in the revised article.

2.AHA10 in arabidopis is unique as it localize to the vacuolar membrane instead of the plasma membrane. If the authors want to find a similar isoform the can start to look at the N-terminal di-leucune motif rather than making qPCT on cotton fibers.

All together I find this study rather descriptive.

**Reply:** This is a very good suggestion. We also knew that the N-terminal motif of AtAHA10 homologues with conserved amino acids (EDLDKPLL) is critical for localizing in the vacuolar membrane, which is described in the paper entitled “Evolution of tonoplast P-ATPase transporters involved in vacuolar acidification” , so we looked at the N-terminal sequenced of P3A-ATPases in upland cotton, but it is regrettable that the similar amino acids motif didn’t been found in the P3A-ATPase genes. In different plants, the PAs metabolic pathway may be somewhat different. In brown cotton, is there a P-type ATPase responsible for the accumulation of PAs? and if so, how does it work? How does it differ from TT13 function in Arabidopsis thaliana? This may be the focus of our next work.

Reviewer: 3

Comments to the Author(s)

1.The manuscript by Chen and colleagues documents the bioinformatic analyses of the P-ATPase gene families in Gossypium hirsutum, G. barbadense, G. raimondii and G. arboretum. The expression of the G. hirsutum gene family is also displayed as a heat map derived from publicly available RNAseq expression data and qRT-PCR is utilized to determine the relative expression levels of the P3A-ATPase genes. The analysis identified a large family of 41 or more genes in the four species and the genes were distributed throughout most of the chromosomes. Figure 1 image was quite small and difficult to read. Also the G. hirsutum chromosomes were not presented in a logical (chronological) order with the At set on the top row and the Dt set on the second row. Also, two chromosomes in the figure were labeled as “Dt-Chr1” presumable the second instance should be “Dt-Chr12”. AtChr1 (at the end of the second row should be shown on the left of the upper row and Dt-Chr1 (at the end of the upper row) should be listed at the left of the second row. There is a typo in the text (p2 lines 84-85) states, “… were all tagged on 10 chromosomes (Fig. 1).”  Actually “12” chromosomes are shown in Figure 1.

**Reply:** Many thanks for this suggestion. we have modified the arrangement of chromosomes in Figure 1. The typo in the text has aslo been modified.( lines 86-87), and the original image of Figure 1 was provided in supplementary material for reading.

2.A phylogenetic analysis of the P-ATPase genes from the 4 Gossypium species (and Arabidopsis) was also performed and shown in Figures 2 and 3. Both of these figures (as included in the manuscript text and printed) are too small and of two low resolution to easily read. This is especially true for the image in Fig. 3 (when magnified 400% on the computer screen) the gene labels are entirely illegible. This must be improved prior to publication. Also, since a significant portion of the paper is focused on this bioinformatic analyses, the protein sequences of the family members identified from the analysis should be provided as supplementary material. For example, this information could be added to Table S1. If this data was provided as a tab delimited text file (or a MicroSoft Excel file), it would be much more user friendly to the reader, and could accommodate inclusion of the predicted protein sequences for the 250 P-ATPase genes.

**Reply:** Thanks for this suggestion. Following the reviewer’s suggestion, the original images of Figure 2 and Figure 3 were provided in supplementary material for reading. The protein sequences for P-ATPase genes of four cotton species and Arabidopsis were provided as supplementary materials.

3.The expression patterns of 74 of the G. hirsutum P-ATPase gene family members are also analyzed from publicly available RNAseq expression data (although this is not made clear in the Results section of the manuscript text). The data is presented as a red-white-blue heat map in Fig. 5, but a clear description of how the data was analyzed and displayed is not provided. The Methods section 5.6 simply states on line 315 that “R software was used to visualize the expression patterns”. This is not sufficient detail to fully interpret the apparent relative expression data shown in Fig. 5. The figure shows a color scale from 3 to -3 (with no description of what that expression range represents) and the legend states that “Different colors represent expression level: red indicates high expression, and blue indicates low expression.” What are the ‘high’ and ‘low’ expressions relative too? This is important because statements like “highly expressed” (p1 line 19), and “… highly expressed in the petal…” (p6 line 162), and “A few genes had higher expression at the three developmental stages…” (p6 lines 163-164) and “… a few genes were highly expressed…” (p6 lines 165-166) all are quantitative judgments. Making statements on the displayed relative expression values (that appear to be relative to themselves i.e. each row appears to go from red to blue (there are no all blue or all red gene expression patterns) – so what is displayed is the variation of the gene transcript abundance (from RNAseq) in the tissue samples relative to itself. This means that almost every gene has a red sample and a blue sample in the heat map. Also, this doesn’t say anything quantitatively about the actually transcript abundance relative to other genes in the heat map (i.e. a highly expressed gene and a gene with very low expression could essentially have the same colors display in the heat map). This type of heat map display can be particularly misleading to the reader – especially when quantitative expression level statements are also made in the text. The authors need to either modify the way the text refers to the gene expression or actually present the data in a quantitative way (which is possible with normalized RNAseq data and a heat map). Also, the normalized RNAseq gene expression data for the G. hirsutum P-ATPase family should be provided as supplementary material as well. This will allow the reader to analyze the data themselves (and can be much more informative than just a heat map alone). On a related note, the Zhang et al., 2015 citation mentioned in the Methods is not included in the Reference list.

**Reply:** We also realized that the figure does not clearly indicate the level of gene expression. So we normalized the RNAseq data and redraw the heat map. In the new figure, the gene expression level can be clearly shown, and the RNAseq gene expression data was provided as a supplementary material for reader to analyze. The citation mentioned (Zhang et al., 2015) has been removed.

4.Quantitative RT-PCR analyses were performed on 31 of the P3A-ATPase genes during cotton fiber development. The UBQ7 gene was used as internal reference gene, but no citation or primer sequences were provided demonstrating this was an appropriate reference for the expression analysis conducted. The authors should add this information.

**Reply:** We have added the primer sequences of UBQ7 in the Table S5, and the reference was also added in the method.

5. Part of the first paragraph of the Discussion section (p9 -10 lines 207-222) are just a long list of published results characterizing the expression of P-ATPase genes. It is not clear why this is brought up in the Discussion (this seems more like just a review of the literature). Making it clear why this information is included and relevant to the results presented in the manuscript is necessary, or the information should be removed from the manuscript or moved to the Introduction.

**Reply:** In discussion section, we listed the expression of P-ATPase genes in other plants to compare the P-ATPase genes in upland cotton whether there were similar expressions. According to the expression patterns, we can inferr preliminarily the potential functions of these genes in upland cotton. It may not be described clearly, and we have modified this part. (line 209-244)
